# Radical Strategy to the Boron-to-Copper Transmetalation Problem: *N*‑Alkylation with Alkylboronic Esters

**DOI:** 10.1021/jacs.5c07856

**Published:** 2025-06-23

**Authors:** Ruocheng Sang, Jason E. Gestwicki

**Affiliations:** Department of Pharmaceutical Chemistry and Institute for Neurodegenerative Diseases, 8785 University of California San Francisco, San Francisco, California 94158, United States

## Abstract

Organoboron compounds represent highly versatile reagents in organic synthesis, but their application to forge C­(sp^3^)–heteroatom bonds, particularly C­(sp^3^)–N coupling, remains underdeveloped. While the integration of alkylboron reagents with copper catalysis offers a promising approach, the inherently sluggish boron-to-copper transmetalation presents a significant challenge. Here, we develop a radical strategy to circumvent the two-electron transmetalation problem via an alkyl radical-capture mechanism, wherein the alkyl radical is generated through an aminyl radical-mediated boron abstraction. Leveraging a reductively activated boron-group transfer reagent, this mechanistically distinct approach enables a general *N*-alkylation using a diverse array of readily available *N*-nucleophiles and alkylboronic esters. This transformation differs fundamentally from previous approaches to provide a greater number of *N*-derivatives of complex small molecules with excellent regioselectivity. This method’s operational simplicity, broad functionality compatibility, and scalability enable streamlined access to *N*-alkylated architectures, addressing unmet needs in medicinal and process chemistry programs.

## Graphical abstract

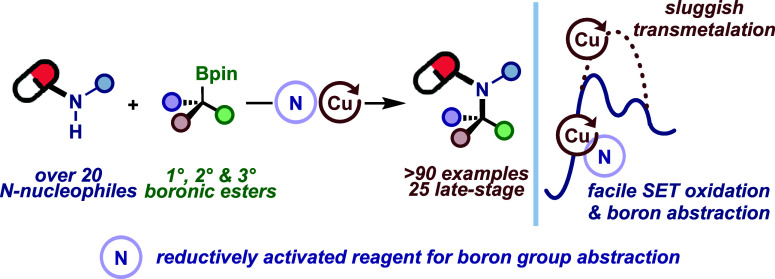

## Introduction

Transition-metal-catalyzed cross-coupling reactions using organoboron reagents are indispensable in modern synthetic chemistry, enabling efficient access to structurally complex organic molecules.^1^ While couplings of aryl organoborons have seen transformative progress in recent decades,^2^ the functionalization of alkylboronic derivatives remains underdeveloped (Figure 1a). This limitation arises from inherent kinetic and mechanistic issues in C­(sp^3^)-hybridized systems, especially sluggish transmetalation, competing β-hydride elimination and protodemetalation of alkylmetal intermediates.^3^ To address these issues, rational ligand design^4,5^ and metallophotoredox catalysis^5–8^ have been employed to expand access to sterically hindered and unactivated alkyl substrates, but these strategies remain largely restricted to C­(sp^3^)–C­(sp^2^) coupling via nickel catalysis. Moreover, while boronate groups arguably can be converted to virtually any functional groups, their general application to forge C­(sp^3^)–heteroatom bonds within practical scopes of N- and O-coupling partners remains constrained.^9^ To achieve fragment coupling with these challenging nucleophilic partners, the strategic incorporation of alkyl organoboron reagents into copper catalysis represents a promising frontier. Central to this approach is the prerequisite transmetalation step prior to accessing high-valent Cu­(III) intermediates, which exhibit exceptional proficiency in facilitating facile product-forming reductive elimination and superior performance in comparison to palladium and nickel catalysis paradigms.^10^ However, no solution exists to address the notoriously sluggish alkylboron-to-copper transmetalation problem. This critical barrier underscores the need for mechanistic development to decouple two-electron transmetalation from the catalytic cycle for C­(sp^3^)–heteroatom bond formation, particularly C–N coupling.

**Figure 1:**
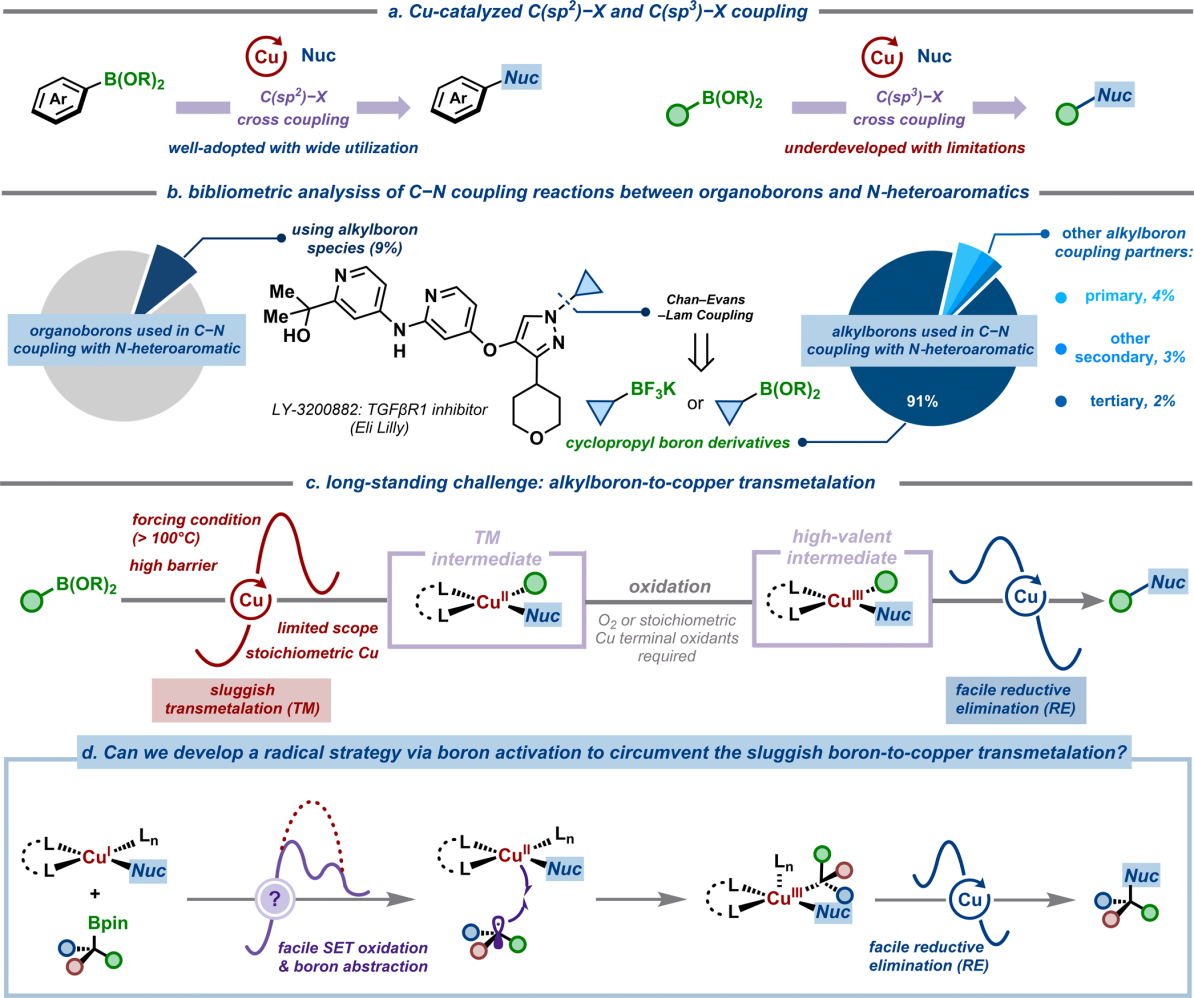
Copper-catalyzed cross-couplings between organoboron reagents and various nucleophiles. (a) Copper-catalyzed C­(sp^3^)–X cross-couplings: an untapped potential. (b) Bibliometric analysis of organoboron–*N*-heteroaromatics coupling reaction. (c) Boron-to-copper transmetalation: A long-standing challenge. (d) This work: A radical strategy to circumvent the sluggish boron-to-copper transmetalation.

C–N bond formation represents a cornerstone of chemical synthesis. In 2024, a vast majority (84%) of FDA-approved drugs featured *N*-heterocyclic frameworks,^11^ while C–N installation reactions constituted an estimated 18% of synthetic transformations executed at the drug discovery stage.^12^ Yet, the methods for producing these bonds, such as classical S_N_2/S_N_1 alkylation, are only effective for primary alkyl halides, faltering dramatically with secondary or tertiary substrates^13,14^ because of competitive elimination, poor regioselectivity, and overalkylation. Alternative approaches such as reductive amination,^15^ Mitsunobu substitution,^16^ and olefin hydroamination^17^ offer partial solutions for alkylamine couplings but struggle with secondary or tertiary C­(sp^3^)–electrophiles and other *N*-nucleophiles like azoles, amides, and carbamates. Transition-metal catalysis, such as copper-catalyzed Chan–Evans–Lam (CEL) coupling, has emerged as potential solutions.^18–20^ CEL coupling proceeds under mild conditions, thereby accommodating sensitive functionalities and complex substrates by bypassing the demanding oxidative addition of organohalides. However, it is predominantly limited to sp^2^-hybridized organoboron reagents,^20^ with the extension to C­(sp^3^)–N couplings proving particularly difficult. Although oxidative amination methods, such as alkyl variants of CEL couplings, have extended the scope of organoboron compounds beyond early successes with cyclopropyl^21,22^ and methyl boron reagents^23^ to include unactivated primary and benzylic organoborons,^19,24–27^ they still suffer from poor efficiency with unactivated secondary organoborons due to the persistent challenge of alkylboron-to-copper transmetalation. Additionally, the scope of the *N*-coupling partners remains narrow, particularly with heteroaromatic substrates. Beyond copper-catalyzed methods, alternative C­(sp^3^)–heteroatom bond-forming strategies have emerged under transition-metal-free conditions—for example, stereospecific amination of organoboron reagents via 1,2-metalate rearrangement mediated by aminating reagents.^28–31^ However, these approaches are largely limited to the synthesis of alkyl amines. Electrochemical functionalization offers a complementary strategy for universal C­(sp^3^)–heteroatom bond construction but is generally restricted to benzylic or tertiary organoboron substrates.^32^

To quantify these limitations, we performed a bibliometric analysis of *N*-heteroaromatic–organoboron cross-coupling reactions, which reveals a stark disparity: 91% of reported examples employ sp^2^-hybridized organoboron species, while only 9% utilize alkyl organoborons (Figure 1b). We hypothesize that this disparity mainly stems from inherent problems in transmetalation, wherein reactivity inversely correlates with C–B bond strength. Notably, the strained cyclopropyl rings present an exception,^22,21^ as their enhanced reactivity stems from strain-induced nucleophilicity. This unique property enables effective C–N coupling, exemplified in the synthesis of the TGFβR1 inhibitor LY-3200882. Conversely, unactivated secondary alkylborons appear in <3% of reported cases, reflecting persistent reactivity barriers. Thus, we aim to establish a general synthetic platform that is able to accommodate a wide range of both *N*-coupling partners and alkyl organoboron compounds.

The challenges associated with alkylboron compounds are their inherently low reactivity due to the lower *s*-character of sp^3^-hybridized carbon. This property diminishes their capacity to stabilize the transient charge buildup during the transmetalation process, resulting in slower reaction rates.^33^ Computational studies illuminated this limitation, identifying the formation of the [C–Cu­(II)] transmetalation intermediate as a high-energy, rate-determining step in conventional two-electron pathways (Figure 1c).^34^ While silver additives facilitate transmetalation in some C–C couplings of activated benzylic boronic esters^35^ or primary trifluoroborate salts,^36^ their mechanistic role is undefined. Leveraging neighboring boronate groups^37^ or preactivating the boronate moiety^38–40^ can accelerate boron-to-copper transmetalation. Recent advances have even enabled stereospecific, copper-catalyzed amination of organoboronic esters with *N*-electrophiles such as hydroxylamine derivatives to furnish secondary and tertiary amines.^38–40^ Nonetheless, these solutions demand specific substrate geometries or a stoichiometric strong base. To overcome the boron-to-copper transmetalation problem, we develop a distinct strategy that circumvents the prohibitive energy barrier of the conventional two-electron process (Figure 1d). Our approach leverages a combination of single-electron copper oxidation/aminyl radical-mediated boron abstraction/radical capture mechanism to allow the functionalization of alkylboronic derivatives in a general format. Moreover, this catalytic cross-coupling has been exploited to establish a general platform filling the long-standing gap in C­(sp^3^)–N cross-coupling, enabling transformations between various alkylboronic esters and *N*-nucleophiles that have remained elusive with classic transition-metal catalysis.

We envisioned the integration of single-electron transfer (SET) with copper catalysis to provide a powerful tool for C­(sp^3^)–N coupling (Figure 2), relying on photochemical activation to generate alkyl radicals. The proposed, unified catalytic cycle generates alkyl radicals and [Cu­(II)–amido] species,^41–50^ facilitating product formation through reductive elimination from Cu­(III) obtained from alkyl radical-to-Cu­(II) oxidative ligation. Fu and Peters pioneered this approach by employing photochemistry to engage unactivated alkyl and aryl halides in C–N couplings,^41–43^ which demonstrated that photoexcitation of the transient [Cu­(I)–amido] complex generates highly reducing species capable of SET to organic halides. These seminal contributions established that merging open-shell radical processes with copper catalysis enables C­(sp^3^)–N couplings that are inaccessible by classical ionic substitution pathways.^41–47^ An alternative strategy involves merging copper catalysis with visible-light photoredox catalysis using alkyl radical precursors. While carboxylic acids and their activated derivatives (*E*_red_ > −1.5 V vs SCE) excel as alkyl radical precursors under photoredox catalysis,^48–50^ alkyl halides (*E*_red_ < −2.0 V vs SCE) pose challenges due to their resistant reduction potentials. To address this limitation, Leonori and MacMillan developed elegant solutions using radical halogen-atom transfer by leveraging α-aminoalkyl and silyl radicals to abstract halogens under mild conditions,^51,52^ thereby bypassing direct substrate reduction to achieve radical-mediated C­(sp^3^)–N couplings. In a similar vein, alkyl organoborons represent another class of electrochemically inert substrates (*E*_red_ < −2.0 V vs SCE).^53^ This thermodynamic constraint motivated our investigation into a reductively activated boron abstraction strategy synergized with copper catalysis to generate Cu­(III) intermediates for this C–N fragment coupling. This strategy would benefit from radical deboronation occurring independently of the copper catalysis cycle and the specific nature of *N*-nucleophiles while also eliminating the need for additional photocatalysis.

**Figure 2:**
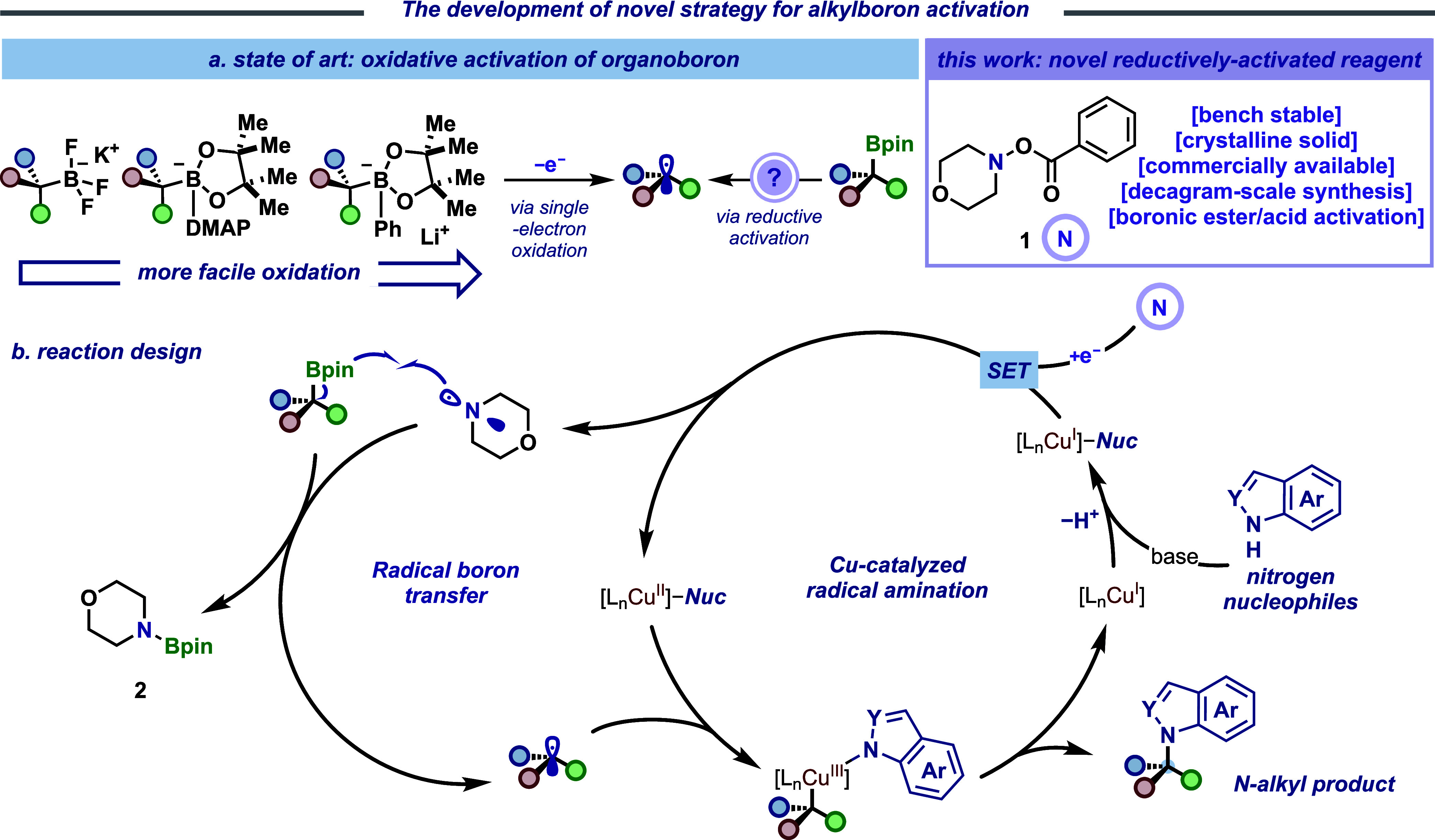
Development of coupling reactions between alkylboronic esters and *N*-nucleophiles by merging boron-group transfer and copper catalysis. (a) Boron activation via a single-electron oxidation process. (b) Reaction design with a novel reductively activated reagent **1**.

Previous studies have shown that single-electron deboronation typically requires reactive four-coordinate “ate” complexes as boron-based radical precursors. These include trifluoroborate salts,^6,7^ *N*- and *P*-based Lewis base complexes,^54,55^ and electron-rich arylboronate complexes^53,56^ (Figure 2a). Several of these methods demand strong bases, and the resulting alkyl radicals are limited to C­(sp^3^)–C­(sp^2^) coupling reactions. However, the requirement for single-electron approaches fundamentally differs from our proposed copper-catalyzed process, which relies on a reductively activated pathway (Figure 2b). Given these redox constraints, it represents an unmet challenge to merge aliphatic organoborons with copper catalysis. In pursuit of a redox-compatible pathway, we identified the reductive boron activation as crucial to our desired transformation, yet no reductively activated analogue has yet been reported. Morpholino benzoate **1** (*E*_red_ ≈ 1.1 V vs SCE)^57^ was recognized as a key dual-function-activated reagent as an oxidant for Cu­(I)-to-Cu­(II) oxidation (*E*_1/2_[Cu­(II)/Cu­(I)] = 0.47 V vs SCE)^58^ and as a source of an *N*-centered radical capable of promoting boron abstraction. Mechanistically, the known ground-state SET between [Cu­(I)–amido] (formed with deprotonated *N*-nucleophile and a Cu­(I) precatalyst) and **1** can afford a [Cu­(II)–amido] complex and induce simultaneous N–O bond homolytic cleavage,^59^ generating a benzoate anion and a nucleophilic aminyl radical (ω ≈ 1.3 eV).^60^ This nucleophilic aminyl radical demonstrates appropriate philicity and reactivity for a polarity-matched interaction with the vacant p-orbital of the boronic ester moiety,^61^ leading to rapid C–B bond scission. This process results in the corresponding alkyl radical concurrent with the formation of morpholinoboronic ester **2**. This mechanism parallels established pathways in metallaphotoredox-catalyzed C­(sp^3^)–C­(sp^2^) cross-coupling8 through single-electron oxidation of amines and visible-light-induced homolytic cleavage of N–N bonds in *N-*nitrosamines.^62^ Subsequently, the Cu­(II) species is captured by the resulting alkyl radical, forming a high-valent [alkyl–Cu­(III)–amido] species that undergoes facile reductive elimination to form the C–N bond and regenerates the Cu­(I) catalyst.^63^

## Results and Discussion

### Reaction Development

Guided by this mechanistic design, we initiated our investigations using *N*-nucleophile **4** as a model along with cyclohexylboronic ester **3** as the alkylating reagent (Figure 3). Under optimized conditions, the reaction employed Cu­(MeCN)_4_PF_6_ as the catalyst, d­(OMe)-Phen (4,7-dimethoxy-1,10-phenanthroline) as the ligand, BTMG (2-*tert*-butyl-1,1,3,3-tetramethyl-guanidine, Barton’s base) as the base, and activated reagent **1** in MeCN, successfully affording *N*-alkylated indazole **5** in 95% yield (Entry 1). Control experiments displayed the essential roles of the copper catalyst and BTMG (Entries 2, 3). Cyclohexylboronic acid proved equally effective (entry 4), whereas trifluoroborate salts were completely ineffective. The modest impact of d­(OMe)-Phen was evidenced by a still-substantial 59% yield in its absence (Entry 5). Reduced reaction performance at room temperature presumably reflects **1**’s diminished capacity for N–O bond scission under ambient conditions (Entry 6). Further investigation of *N*- and *O*-centered radical precursors revealed the decisive impact of the precursor structure and radical polarity on *N*-alkylation efficiency (Figure 3b). While peroxides (DTBP **1c** and BPO **1d**) exhibited superior Cu­(I) oxidation capacity and were employed as oxidants in alkyl variants of CEL couplings,^24,25^ their resultant electrophilic *O*-radicals proved polarity-incompatible with boron-group transfer in our proposed single-electron strategy. Compared to peroxides, O-benzoylhydroxylamines (**1**, **1b**) are weaker oxidants; however, they undergo single-electron transfer with Cu­(I) to generate nucleophilic aminyl radicals that can engage in a polarity-matched interaction with the vacant p-orbital of the boronic ester moiety,^61^ enabling efficient boron-group abstraction. Among them, reagent **1** delivered a high yield of 95%, whereas **1b** gave a yield of only 39%. The critical role of **1** was also conclusively established, whereas reactions under previously reported CEL conditions without **1** failed to yield the product, thereby ruling out background CEL reactions. The traditional ionic substitution approaches with alternative coupling partners were also evaluated, exhibiting significantly lower efficiency (Figure 3c, see Supporting Information 2.6 for details). Notably, the optimal reagent **1** is a commercially available, shelf-stable crystalline solid synthesizable on a decagram scale through direct oxidation of morpholine.^64^ It represents the first example of a reductively activated boron-group transfer reagent, enabling the direct conversion of alkyl organoborons into radical species, without prefunctionalized or electronically activated substrates. The sensitivity assessment confirmed the method’s robustness, maintaining high effectiveness across broad ranges of catalyst loading (2.0–20 mol %), concentration (0.05–0.2 M), and moisture levels (0–25 μL H_2_O), with a slight yield reduction under air (Figure 3d, see Supporting Information 2.3 for details). When the reaction was scaled up to 3.0 mmol, it produced nearly identical isolated yields.

**Figure 3:**
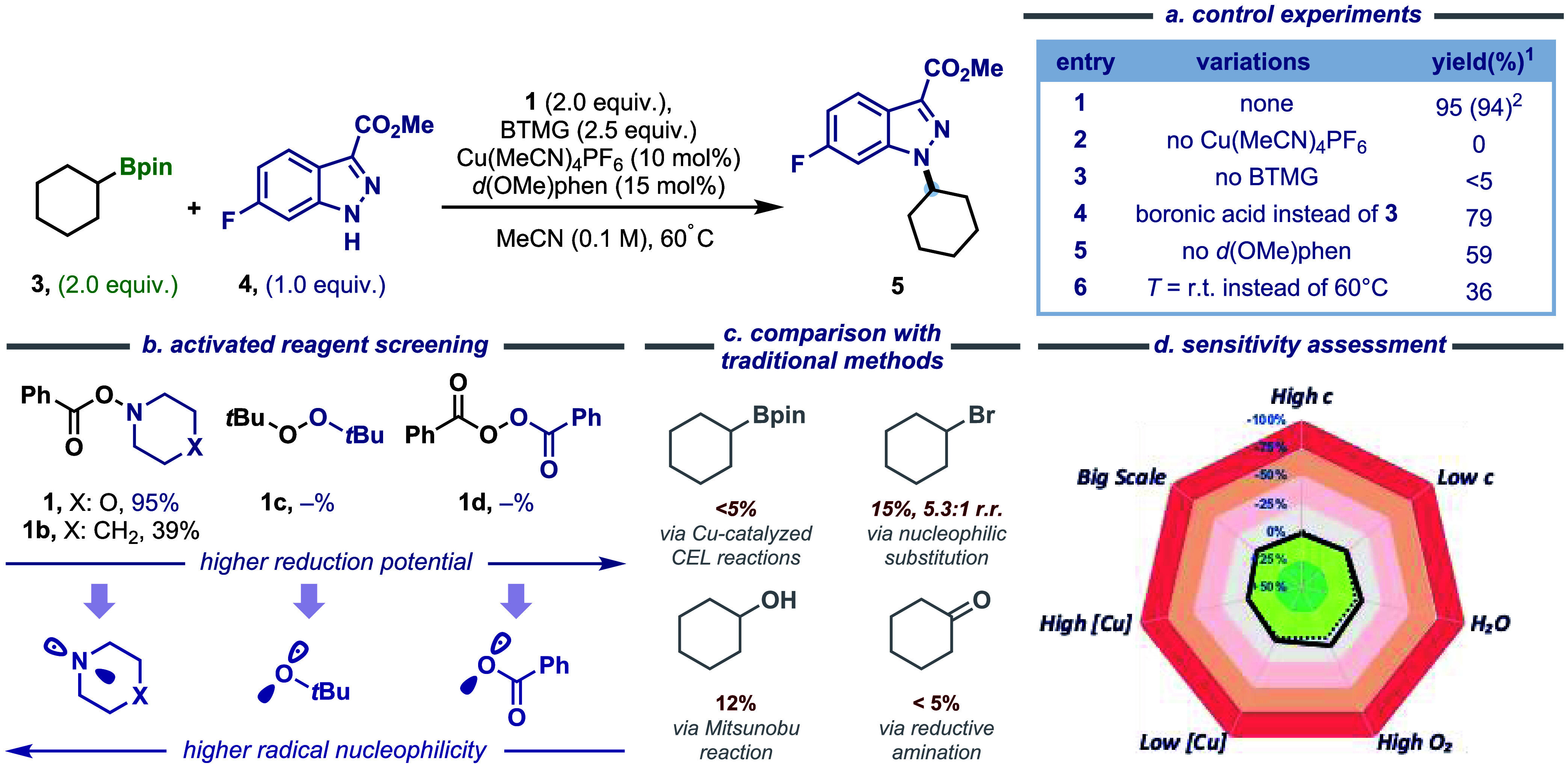
Reaction optimization. (a) Control experiments. (b) Screening of activated reagents. (c) Comparison with traditional methods. (d) Reaction producibility assessment; see the Supporting Information for experimental details. ^1^yield was determined by ^19^F NMR integration relative to methyl 4-fluorobenzoate (0.200 mmol) as the internal standard; ^2^isolated yield.

### Substrate Scope

Having established optimal reaction conditions, we explored the versatility of the reaction with respect to *N*-nucleophiles (Figure 4). This protocol demonstrated high generality across more than 20 classes of medicinally relevant *N-*nucleophiles. Cyclohexylboronic ester **3** was coupled successfully with diverse *N*-heteroaromatics, including indazoles (**5**–**9**), azaindoles (**10**–**12**), indoles (**13**–**16**), azaindoles (**17**–**19**), pyrazoles (**20**–**24**), triazoles (**25**–**27**), imidazoles (**28**,**29**), pyrrole (**30**), and carbazoles (**31**, **32**), to afford corresponding *N*-alkylated products in good to excellent yields. The platform extended to other diverse *N*-nucleophiles: benzamides (**33**, **34**), amides (**35**, **36**), both electron-rich and -poor anilines (**37**, **38**), and the less nucleophilic *N*-pyridyl secondary aniline (**39**). Furthermore, sulfonamide (**40**), sulfoximine (**41**), imine (**42**), carbamate (**43**), dihydroquinolo (**44**), and oxindole (**45**) were also suitable coupling partners. Notably, common challenges with primary anilines and sulfonamides, such as overalkylation, were not observed under these conditions. This approach further achieves a direct synthesis of the tertiary alkylamine (**46**), a transformation previously problematic via metallaphotoredox catalysis due to undesired oxidation of amines by photocatalysts. Finally, pharmaceutically relevant heterocycles were successfully incorporated, including theophylline (**47**) and 2-aminopyrrolo­[2,1-*f*]­[1,2,4]­triazine (**48**), a structural motif in antiviral agents such as remdesivir.

**Figure 4:**
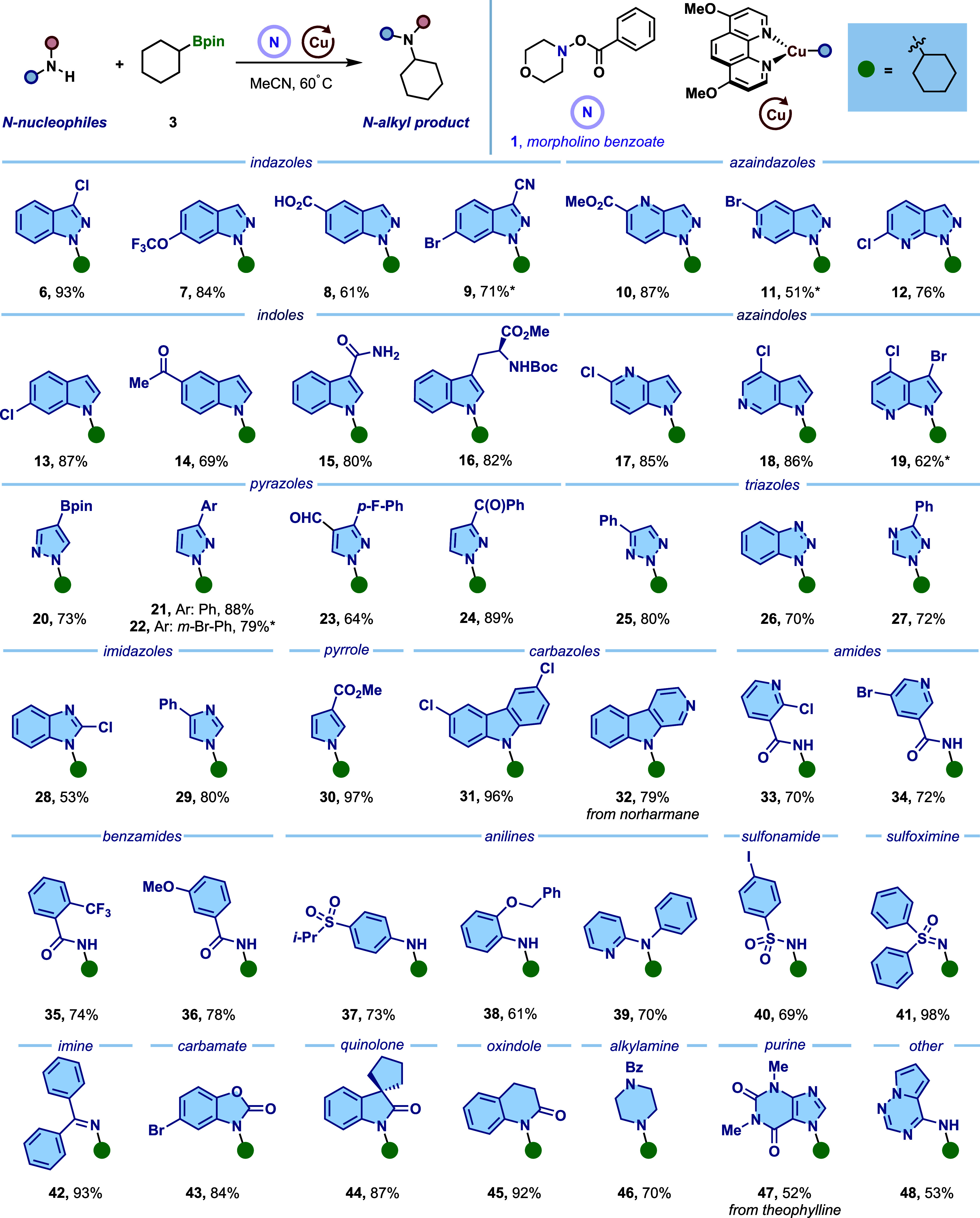
Scope of *N*-nucleophiles. Standard reaction conditions: *N*-nucleophile (0.20 mmol, 1.0 equiv), [Cu­(MeCN)_4_]­PF_6_ (10 mol %), d­(OMe)-Phen (15 mol %), btm (2.5 equiv), alkylboronic ester **3** (2.0 equiv), morpholino benzoate **1** (2.0 equiv), MeCN (*c* = 0.050 or 0.10 M), 16 h, 60 °C. *using CuTC (10 mol %) instead of [Cu­(MeCN)_4_]­PF_6_.

In our scope evaluation, a wide range of functional groups were well-tolerated, including ethers (**7**, **36**, **38**), esters (**5**, **16**, **30**), nitrile (**9**), ketones (**14**, **24**), Boc-protected amine (**10**), and sulfone (**37**). Remarkably, valuable synthetic handles including aryl halides (chlorides: **6**, **12**, **13**, **18**, **19**, **28**, **31**, bromides: **11**, **19**, **22**, **43**, and iodide: **40**), aldehyde (**23**), and free carboxylic acid (**8**) could also be retained. In contrast to traditional ionic substitution, which often encounters selectivity issues,^13,65^ this protocol presents excellent chemoselectivity and exclusive regioselectivity. For instance, indole substrate **15** underwent exclusive alkylation at the indole nitrogen over a competing primary amide, seemingly governed by N–H acidity, while substrate **20** demonstrated selective alkylboron abstraction while preserving arylboronic ester functionality. Critically, substrates containing multiple potential *N*-alkylation sites consistently afforded single constitutional isomers in all cases, with regioselectivity dictated by the steric preferences of ligated [Cu­(I)–amido] species for less hindered positions, aligning with established metallaphotoredox studies.^49–51^

We next shifted our focus to evaluating organoboron coupling partners as alkylating reagents (Figure 5). Primary alkylboronic esters (**49**–**54**) bearing diverse functionalities, nitrile (**50**), protected amine (**51**), terminal olefin (**52**), ester (**53**), and benzyl group (**54**), were coupled efficiently. Secondary boronic esters (**55**–**68**) showed comparable reactivity, encompassing acyclic substrate (**55**) and various cyclic systems ranging from four- to seven-membered rings (**56**–**68**) as well as heterocyclic scaffolds (tetrahydropyran **60**, piperidines **62**, **66**, **67**). Complex architectures such as spirocyclics (**57**, **63**) and deoxy-nortropine alkaloid-derived (**67**) and norbornene-based (**68**) frameworks were alkylated with excellent diastereoselectivity favoring sterically accessible positions. Nevertheless, in contrast to recently reported stereospecific amination reactions of chiral alkylboronic esters,^28–31,38,39^ our radical-based method is not stereospecific. This is expected, as the generation of free alkyl radicals typically results in racemization due to rapid scrambling of configuration prior to bond formation.^13^ Finally, tertiary alkyl groups were directly installed to provide heteroaryl *N*-tertiary alkyl derivatives (**69**–**72**). This capability is exemplified by an *N*-substituted bicyclo[1.1.1]­pentane (**70**), a valuable benzene bioisostere.

**Figure 5:**
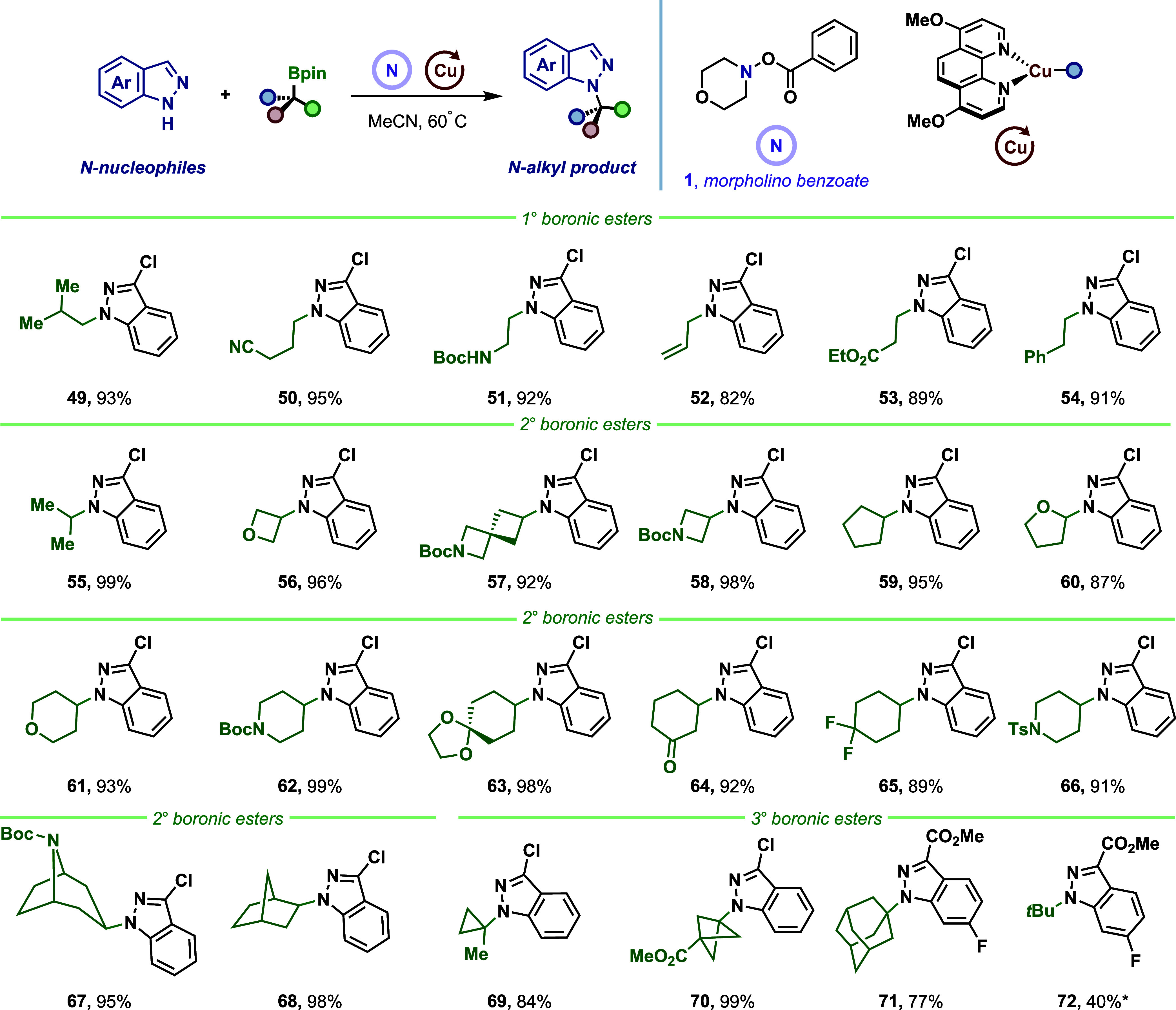
Scope of the boronic ester. Standard reaction conditions: *N*-nucleophile (0.20 mmol, 1.0 equiv), [Cu­(MeCN)_4_]­PF_6_ (10 mol %), d­(OMe)-Phen (15 mol %), BTMG (2.5 equiv,), alkylboronic ester (2.0 equiv), morpholino benzoate **1** (2.0 equiv), MeCN (*c* = 0.10 M), 16 h, 60 °C. *using 5 equiv. *tert*-butylboronic ester.

To highlight the practical impact, this C–N-coupling platform was applied to late-stage *N*-alkylation of numerous densely functionalized complex bioactive molecules and pharmaceuticals (Figure 6). This approach consistently afforded single regioisomers with exceptional selectivity across diverse substrates (**73**–**94**). Importantly, the protocol directly accommodated carboxylic acid (**74**), alcohol (**76**), and secondary amines (**76**, **80**, **92**) in a protecting-group-free manner, eliminating protection/deprotection sequences commonly required in medicinal chemistry workflows. Furthermore, broad tolerance to a vast array of complementary functional groups was exhibited, including acetal (**77**), cyclopropyl (**81**), isoxazole (**82**), thiazole (**88**), internal alkene (**94**), and thioester (**94**). Notably, tertiary amines (**85**, **90**), which are usually incompatible in photoredox catalysis due to competitive oxidation, proved to be compatible.

**Figure 6:**
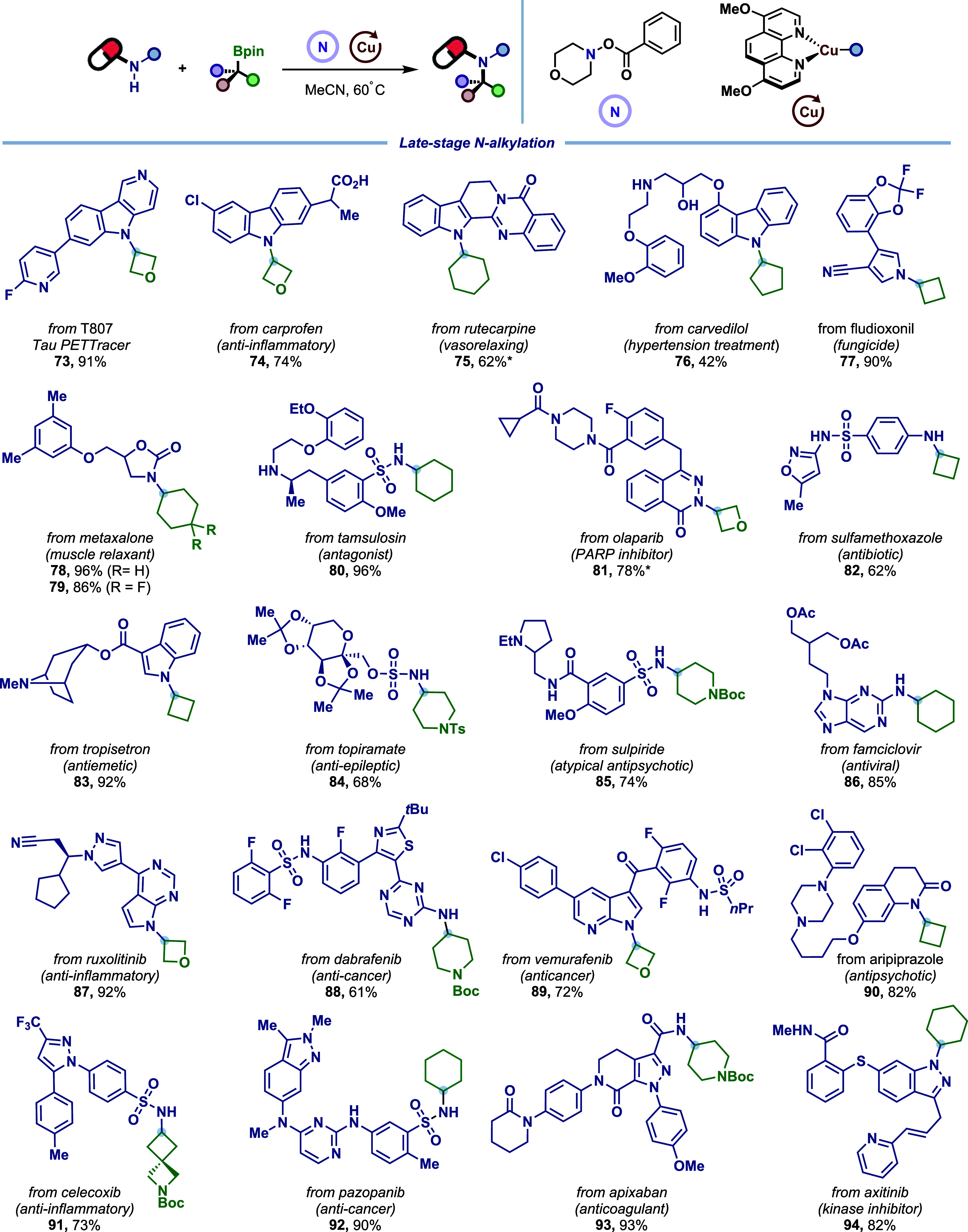
Late-stage *N*-alkylation of complex and biologically active materials. Reaction conditions: *N*-nucleophile (0.20 mmol, 1.0 equiv), [Cu­(MeCN)_4_]­PF_6_ (10 mol %), d­(OMe)-Phen (15 mol %), BTMG (2.5 equiv,), alkylboronic ester (2.0 equiv), morpholino benzoate **1** (2.0 equiv), anhydrous MeCN (*c* = 0.050 or 0.10 M), 16 h, 60 °C. *without d­(OMe)-Phen.

To further explore the utility of this method, we applied a one-pot borylation–amination sequence to olefin and alkane substrates (Figure 7a). The initial conversion of these compounds to boronic esters via iridium-catalyzed hydroboration^66^ and C–H borylation^67^ followed by C–N coupling afforded *N*-alkylated celecoxib (**95**, 78% yield) and a tryptophan derivative (**96**, 80% yield) without intermediate purification. Gram-scale synthesis of pharmaceutical analogues (**97** from metaxalone, **98** from fludioxonil) demonstrates robust scalability at both 1- and 3-g scales (Figure 7b). Thus, a key advantage of this method is its inherent robustness, which does not necessitate the use of electrochemical/photochemical equipment while enabling challenging or previously inaccessible transformations, positioning it as a versatile platform for process-driven synthesis.

**Figure 7:**
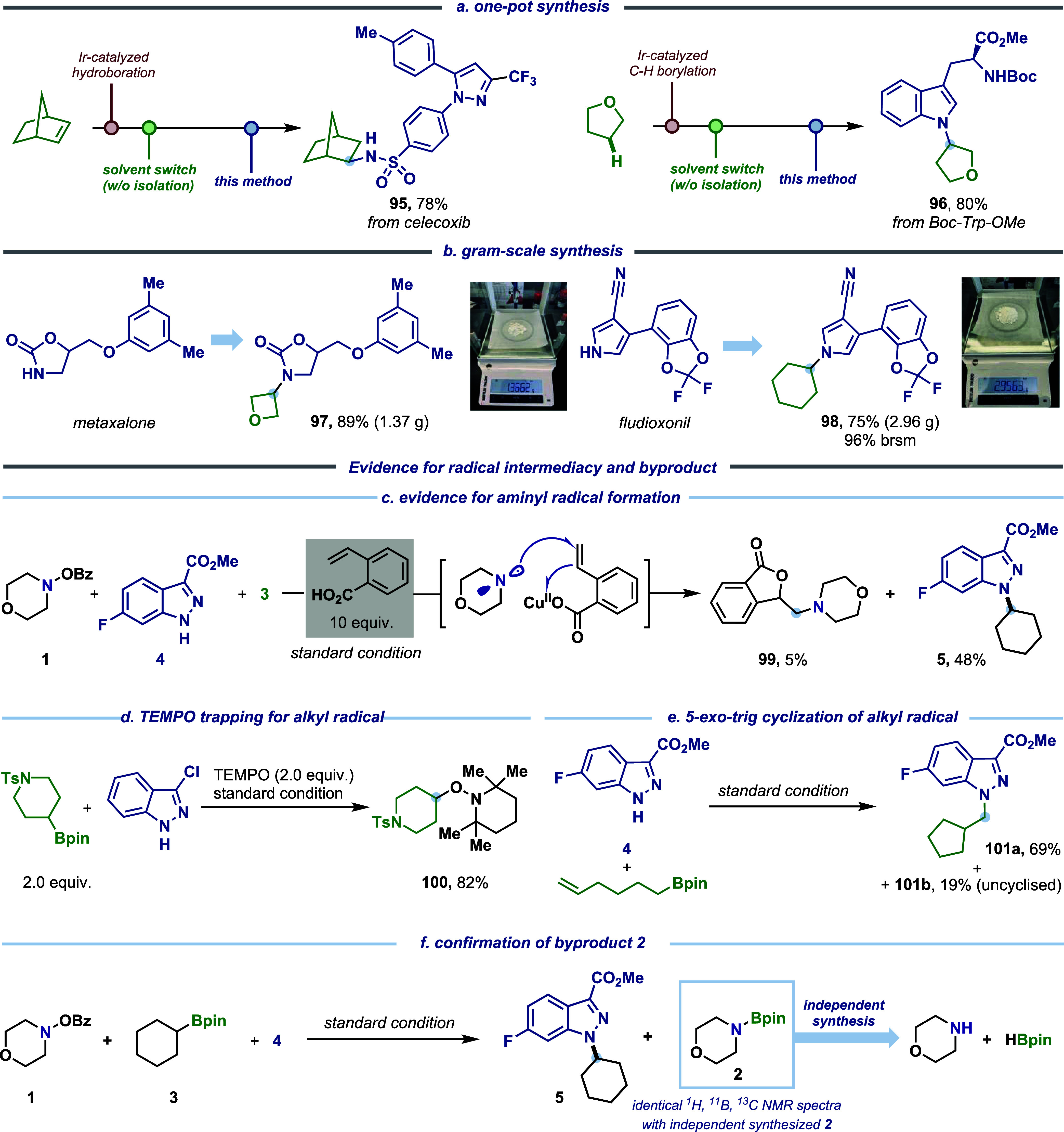
(a) One-pot synthesis of *N*-alkylated pharmaceutical analogues (**95**, **96**). (b) Application to late-stage gram-scale synthesis (**97**, **98**). (c) Evidence for aminyl radical formation. (d) TEMPO trapping experiment. (e) 5-*exo*-trig cyclization experiment. (f) Confirmation of byproduct **2**.

### Mechanistic Study

Mechanistic investigations provided compelling evidence for radical intermediacy and the byproduct of this transformation. To probe for the aminyl radical, addition of 2-vinylbenzoic acid under standard conditions afforded an aminolactonization product (**99**) (Figure 7c). As proposed, the single-electron oxidation of [Cu­(I)–amido] with **1** yields the corresponding Cu­(II) complex while concurrently generating the aminyl radical. This radical then participates in electrophilic amination, ultimately leading to a copper-catalyzed aminolactonization process.^59^ The marked yield difference between the *N*-alkyl product (**5**) and aminolactonization product (**99**) (48 vs 5%) indicates that the aminyl radical predominantly engages in boron-group transfer over direct radical alkene addition. Further experiments substantiated an alkyl radical intermediate. In the presence of 2,2,6,6-tetramethylpiperidin-1-oxyl (TEMPO), an alkyl-trapped TEMPO adduct (**100**) was isolated in 82% yield (Figure 7d), while a radical clock experiment produced cyclopentylmethyl indazole (**101a**) and a linear derivative (**101b**) in a 3.6:1 ratio via 5-*exo*-trig–amination. To confirm the formation of morpholinoboronic ester **2** alongside the alkyl radical, the compound was synthesized independently by dehydrogenating pinacolborane (HBPin) with morpholine.^68^ NMR analyses of both the reaction-derived and independently synthesized samples revealed identical spectroscopic features, verifying the identity of byproduct **2** (Figure 7f, see Supporting Information 3.3 for details).

## Conclusions

We overcome the inherent limitations of sluggish boron-to-copper transmetalation through an alkyl radical-capture strategy, utilizing the first reductively activated boron transfer reagent, to achieve a general, user-friendly *N*-alkylation platform for nearly all classes of medicinally relevant *N*-nucleophiles with alkylboronic esters. This protocol exhibits great utility for medicinal chemistry applications, particularly in late-stage functionalization of complex pharmaceuticals, and demonstrates scalability for large-scale synthesis. By addressing this long-standing bottleneck, it paves the way for previously inaccessible reactions between alkyl organoborons and diverse coupling partners, thereby broadening the horizons of synthetic cross-coupling chemistry.

## Supplementary Material



## Notes

Additional experimental details, including procedures, photographs of the experimental setup, characterization data, and spectra (PDF)
