# Supplementary material for: Radical Strategy to the Boron-to-Copper Transmetalation Problem: N‑Alkylation with Alkylboronic Esters
Source: J Am Chem Soc. 2025 Jun 23;147(26):23259–69. doi: 10.1021/jacs.5c07856 (PMC12232318; doi:10.1021/jacs.5c07856)
Supplement: Supplementary file 1 [file ja5c07856_si_001.pdf]

SUPPLEMENTARY INFORMATION

Radical Strategy to the Boron-to-Copper Transmetalation Problem: *N*-alkylation with Alkylboronic Esters

Ruocheng Sang*, Jason E. Gestwicki*

Institute for Neurodegenerative Diseases and Department of Pharmaceutical Chemistry, University of California San Francisco, San Francisco, CA 94158, USA

*e-mail: ruocheng.sang@ucsf.edu; jason.gestwicki@ucsf.edu

TABLE OF CONTENTS

LIST OF SUPPLEMENTARY SCHEMES, FIGURES AND TABLES 3

LIST OF CHARACTERIZED PRODUCTS 4

1. MATERIALS AND GENERAL METHODS 7

1.1. Glassware, Solvents and Reagents 7

1.2. Chromatography and Instrumentation 7

1.3. Naming of Compounds 7

2. EXPERIMENTAL DATA 8

2.1. General Procedures 8

2.1.1. General Procedure A: Coupling of *N*-nucleophiles with cyclohexylboronic ester **3** 8

2.1.2. General Procedure B: Coupling of 3-chloro1*H*-indazole with alkyl boronic esters 8

2.1.3. General Procedure C: Late-stage alkylation 9

2.2. Reaction Optimization 10

2.2.1. Standard procedure for reaction optimization 10

2.2.2 Ligand screening 10

2.2.3 Copper catalyst screening 10

2.2.3 Base screening 11

2.2.4 Solvent concentration screening 11

2.2.5 Temperature screening 12

2.2.6 Stoichiometry of BTMG screening 12

2.2.7 Stoichiometry of **1** and **3** screening 13

2.2.8 Activation reagent screening 13

2.2.9 Boron species screening 14

2.2.10 Control experiments 14

2.3 Sensitivity Screening 15

2.4. Substrate Scope 16

2.5. Unsuccessful Substrates 67

2.6. Comparison with Previously Reported *N*-alkylation Methods 68

2.7. Literature Analysis of *N*-heteroaromatic–organoboron Coupling Reactions 69

3. MECHANISTIC STUDIES 70

3.1. [L_n–Cu(I)–amido] Formation by the Coordination of **S6** to [L_n–Cu(I)] 70

3.2 Single-electron-oxidation of [L_n–Cu(I)–amido] to [L_n–Cu(I)–amido] by Reagent **1** 74

3.2.1 Probe the aminyl-radical trapping adduct **99** 79

3.3 Probing the Formation of Side Product **2** 80

3.4 Evidence for Alkyl Radical Intermediacy 85

3.4.1 TEMPO trapping experiment for alkyl radical trapping adduct **100** 85

3.4.2 5-*exo*-trig cyclization experiment for alkyl radical trapping product **101a** and **101b** 86

3.5 Probing Radical Capture by [Cu(II)–amido] Species as C–N Bond Forming Step 87

6. SPECTROSCOPIC DATA 88

7. REFERENCES 206

LIST OF SUPPLEMENTARY SCHEMES, FIGURES AND TABLES

Table S1. Ligand screening 10
Table S2. Catalyst screening 11
Table S3. Base screening 11
Table S4. Solvent concentration screening 12
Table S5. Temperature screening 12
Table S6. Temperature screening 13
Table S7. Stoichiometry of **1** and **3** screening 13
Figure S1. Activation reagent screening 13
Table S8. Boron species screening 14
Table S9. Control experiment 14
Table S10. Sensitivity screening 15
Figure S2: Radar diagram representation of sensitivity screen 15
Figure S3: Unsuccessful substrates 67
Figure S4: Comparison with previously reported *N*-alkylation methods; 68
Figure S5: Literature analysis of *N*-heteroaromatic–organoboron coupling reactions 69
Figure S6: Proposed mechanism 70
Figure S7: Studies towards the formation of [L_n–Cu(I)–amido] from indazole **S6** 70
Figure S8: ^{1}H NMR spectroscopy studies towards the formation of [L_n–Cu(I)–amido] from indazole **S6** 71
Figure S9: ^{1}H NMR spectroscopy studies towards the coordination of $Cu(MeCN)_4PF_6$ to BTMG 71
Figure S10: UV/Vis absorption spectroscopy studies towards the coordination of $Cu(MeCN)_4PF_6$ to the ligand, *d*(OMe)Phen 72
Figure S11: UV/Vis absorption spectroscopy studies towards the coordination of $Cu(MeCN)_4PF_6$ to the ligand *d*(OMe)Phen, and BTMG 72
Figure S12: UV/Vis absorption spectroscopy studies towards the formation of [L_n–Cu(I)–amido] from indazole **S6** with ligand *d*(OMe)Phen, and BTMG 73
Figure S13: UV/Vis absorption spectroscopy studies towards the interaction between [L_n–Cu(I)–amido] and cyclohexylboronic acid pinacol ester **3** 74
Figure S14: ^{1}H NMR spectroscopy studies towards the oxidation of $Cu(MeCN)_4PF_6$ enabled by reagent **1** 75
Figure S15: ^{1}H NMR spectroscopy studies towards the oxidation of [L_n–Cu(I)–amido] enabled by reagent **1** with BTMG ligand 76
Figure S16: ^{1}H NMR spectroscopy studies towards the oxidation of [L_n–Cu(I)–amido] enabled by reagent **1** with *d*(OMe)Phen and BTMG ligands 77
Figure S17: UV/Vis absorption spectroscopy studies towards the oxidation of [L_n–Cu(I)–amido] enabled by reagent **1** with *d*(OMe)Phen and BTMG ligands 78
Figure S18: studies towards the oxidation of [L_n–Cu(I)–amido] by reagent **1** 78
Figure S19: Probe the aminyl radical intermediacy by electrophilic amination 79
Figure S20: ^{1}H NMR spectroscopy studies towards the interaction between **1** and **3** 80
Figure S21: ^{11}B NMR spectroscopy studies towards the interaction between **1** and **3** 81
Figure S22: ^{1}H NMR spectroscopy comparison standard reaction formed and independent synthetic **2** 82
Figure S23: ^{13}C NMR spectroscopy comparison standard reaction formed and independent synthetic **2** 83
Figure S24: ^{11}B NMR spectroscopy comparison standard reaction formed and independent synthetic **2** 84
Figure S25: TEMPO trapping for alkyl radical 85
Figure S26: 5-*exo*-trig cyclization of alkyl radical 86
Figure S27: Probing radical capture by [Cu(II)–amido] species as C–N bond forming step 87

LIST OF CHARACTERIZED PRODUCTS

Methyl 1-cyclohexyl-6-fluoro-1*H*-indazole-3-carboxylate (**5**) 16
3-Chloro-1-cyclohexyl-1*H*-indazole (**6**) 16
1-Cyclohexyl-5-(trifluoromethoxy)-1*H*-indazole (**7**) 17
1-Cyclohexyl-1*H*-indazole-5-carboxylic acid (**8**) 17
6-Bromo-1-cyclohexyl-1*H*-indazole-3-carbonitrile (**9**) 18
Methyl 1-cyclohexyl-1*H*-pyrazolo[4,3-b]pyridine-5-carboxylate (**10**) 18
5-Bromo-1-cyclohexyl-1*H*-pyrazolo[3,4-c]pyridine (**11**) 19
6-Chloro-1-cyclohexyl-1*H*-pyrazolo[3,4-b]pyridine (**12**) 19
6-Chloro-1-cyclohexyl-1*H*-indole (**13**) 20
1-(1-Cyclohexyl-1*H*-indol-6-yl)ethan-1-one (**14**) 20
1-Cyclohexyl-1*H*-indole-3-carboxamide (**15**) 21
Methyl *N*-(tert-butoxycarbonyl)-1-cyclohexyl-*L*-tryptophanate (**16**) 21
5-Chloro-1-cyclohexyl-1*H*-pyrrolo[3,2-b]pyridine (**17**) 22
4-Chloro-1-cyclohexyl-1*H*-pyrrolo[2,3-c]pyridine (**18**) 22
3-Bromo-4-chloro-1-cyclohexyl-1*H*-pyrrolo[2,3-*b*]pyridine (**19**) 23
1-Cyclohexyl-4-(4,4,5,5-tetramethyl-1,3,2-dioxaborolan-2-yl)-1*H*-pyrazole (**20**) 23
1-Cyclohexyl-3-phenyl-1*H*-pyrazole (**21**) 24
3-(3-Bromophenyl)-1-cyclohexyl-1*H*-pyrazole (**22**) 24
1-Cyclohexyl-3-(4-fluorophenyl)-1*H*-pyrazole-4-carbaldehyde (**23**) 25
(1-Cyclohexyl-1*H*-pyrazol-3-yl)(phenyl)methanone (**24**) 25
2-Cyclohexyl-4-phenyl-2*H*-1,2,3-triazole (**25**) 26
1-Cyclohexyl-1*H*-benzo[*d*][1,2,3]triazole (**26**) 26
1-Cyclohexyl-3-phenyl-1*H*-1,2,4-triazole (**27**) 27
2-Chloro-1-cyclohexyl-1*H*-benzo[d]imidazole (**28**) 27
1-Cyclohexyl-4-phenyl-1*H*-imidazole (**29**) 28
Methyl 1-cyclohexyl-1*H*-pyrrole-3-carboxylate (**30**) 28
3,6-Dichloro-9-cyclohexyl-9*H*-carbazole (**31**) 29
9-Cyclohexyl-9*H*-pyrido[3,4-b]indole (**32**) 29
2-Chloro-*N*-cyclohexylnicotinamide (**33**) 30
5-Bromo-*N*-cyclohexylnicotinamide (**34**) 30
N-Cyclohexyl-2-(trifluoromethyl)benzamide (**35**) 30
N-Cyclohexyl-3-methoxybenzamide (**36**) 31
4-(Isopropylsulfonyl)-*N*-cyclohexylaniline (**37**) 31
2-(Benzyloxy)-*N*-cyclohexylaniline (**38**) 32
N-Cyclohexyl-*N*-phenylpyridin-2-amine (**39**) 32
N-Cyclohexyl-4-iodobenzenesulfonamide (**40**) 33
(Cyclohexylimino)diphenyl-l6-sulfanone (**41**) 33
N-Cyclohexyl-1,1-diphenylmethanimine (**42**) 34
5-Bromo-3-cyclohexylbenzo[*d*]oxazol-2(3*H*)-one (**43**) 34
1'-Cyclohexylspiro[cyclopentane-1,3'-indolin]-2'-one (**44**) 35
1-Cyclohexyl-3,4-dihydroquinolin-2(1*H*)-one (**45**) 35
(4-Cyclohexylpiperazin-1-yl)(phenyl)methanone (**46**) 36
9-Cyclohexyl-1,3-dimethyl-3,9-dihydro-1*H*-purine-2,6-dione (**47**) 36

N-Cyclohexylpyrrolo[2,1-*f*][1,2,4]triazin-4-amine (**48**) ... 37
3-Chloro-1-isobutyl-1*H*-indazole (**49**) ... 37
4-(3-Chloro-1*H*-indazol-1-yl)butanenitrile (**50**) ... 38
tert-Butyl (2-(3-chloro-1*H*-indazol-1-yl)ethyl)carbamate (**51**) ... 38
1-Allyl-3-chloro-1*H*-indazole (**52**) ... 39
Ethyl 3-(3-chloro-1*H*-indazol-1-yl)propanoate (**53**) ... 39
3-Chloro-1-phenethyl-1*H*-indazole (**54**) ... 40
3-Chloro-1-isopropyl-1*H*-indazole (**55**) ... 40
3-Chloro-1-(oxetan-3-yl)-1*H*-indazole (**56**) ... 41
tert-butyl 6-(4,4,5,5-tetramethyl-1,3,2-dioxaborolan-2-yl)-2-azaspiro[3.3]heptane-2-carboxylate (**S57**) ... 41
tert-Butyl 6-(3-chloro-1*H*-indazol-1-yl)-2-azaspiro[3.3]heptane-2-carboxylate (**57**) ... 42
tert-Butyl 3-(3-chloro-1*H*-indazol-1-yl)azetidine-1-carboxylate (**58**) ... 42
3-Chloro-1-cyclopentyl-1*H*-indazole (**59**) ... 43
3-Chloro-1-(tetrahydrofuran-2-yl)-1*H*-indazole (**60**) ... 43
3-Chloro-1-(tetrahydro-2*H*-pyran-4-yl)-1*H*-indazole (**61**) ... 44
tert-Butyl 4-(3-chloro-1*H*-indazol-1-yl)piperidine-1-carboxylate (**62**) ... 44
3-Chloro-1-(1,4-dioxaspiro[4.5]decan-8-yl)-1*H*-indazole (**63**) ... 45
3-(3-Chloro-1*H*-indazol-1-yl)cyclohexan-1-one (**64**) ... 45
3-Chloro-1-(4,4-difluorocyclohexyl)-1*H*-indazole (**65**) ... 46
3-Chloro-1-(1-tosylpiperidin-4-yl)-1*H*-indazole (**66**) ... 46
tert-Butyl 3-(3-chloro-1*H*-indazol-1-yl)-8-azabicyclo[3.2.1]octane-8-carboxylate (**67**) ... 47
1-(Bicyclo[2.2.1]heptan-2-yl)-3-chloro-1*H*-indazole (**68**) ... 47
3-Chloro-1-(1-methylcyclopropyl)-1*H*-indazole (**69**) ... 48
Methyl 3-(3-chloro-1*H*-indazol-1-yl)bicyclo[1.1.1]pentane-1-carboxylate (**70**) ... 48
Methyl 1-((3*s*,5*s*,7*s*)-adamantan-1-yl)-6-fluoro-1*H*-indazole-3-carboxylate (**71**) ... 49
Methyl 1-(*tert*-butyl)-6-fluoro-1*H*-indazole-3-carboxylate (**72**) ... 49
N-Alkylated T807 (**73**) ... 50
N-Alkylated carprofen (**74**) ... 50
N-Alkylated rutecarpine (**75**) ... 51
N-Alkylated carvedilol (**76**) ... 51
N-Alkylated fludioxonil (**77**) ... 52
N-Alkylated metaxalone (**78**) ... 52
N-Alkylated metaxalone (**79**) ... 53
N-Alkylated tamsulosin (**80**) ... 54
N-Alkylated olaparib (**81**) ... 54
N-Alkylated sulfamethoxazole (**82**) ... 55
N-Alkylated tropisetron (**83**) ... 55
N-Alkylated topiramate (**84**) ... 56
N-Alkylated sulpiride (**85**) ... 56
N-Alkylated famciclovir (**86**) ... 57
N-Alkylated ruxolitinib (**87**) ... 57
N-Alkylated dabrafenib (**88**) ... 58
N-Alkylated vemurafenib (**89**) ... 58
N-Alkylated aripiprazole (**90**) ... 59
N-Alkylated celecoxib (**91**) ... 60

N-Alkylated pazopanib (**92**) 60
N-Alkylated apixaban (**93**) 61
N-Alkylated axitinib (**94**) 61
One-pot synthesis of *N*-alkylated celecoxib (**95**) 62
One-pot synthesis of *N*-alkylated Boc-Trp-OMe (**96**) 63
Large-scale synthesis of **5** 64
Large-scale synthesis of *N*-alkylated metaxalone **97** 65
Large-scale synthesis of *N*-alkylated fludioxonil **98** 65

1. MATERIALS AND GENERAL METHODS

1.1. Glassware, Solvents and Reagents

All manipulations were performed with oven-dried (130°C for a minimum of 12 h) or flame-dried glassware using standard Schlenk techniques under an atmosphere of nitrogen, unless otherwise stated. All anhydrous solvents were commercially supplied from Sigma-Aldrich® with further purification. Acetonitrile (99.8%, 100 mL, anhydrous, w/o molecular sieves) was purchased from Sigma-Aldrich and used as received inside a glovebox. Morpholino benzoate **1** regent was purchased from Combi-Blocks, Inc., and BTMG was obtained from Ambeed Inc.

1.2. Chromatography and Instrumentation

Liquid chromatography–mass spectrometry (LCMS) analysis was performed using an Agilent® Technologies G6100 Series LC/MSD SingleQuad system to monitor the reactions.

Flash column chromatography (FCC) was carried out on a CombiFlash® R_f purification system using RediSep R_f Gold®silica gel (20−40 μm) purchased from Teledyne Isco, Inc.

NMR spectra were recorded at various field strengths, as indicated, using Bruker® 400 MHz, for ^{1}H, ^{11}B, ^{13}C and ^{19}F acquisitions. All NMR spectra were recorder at 25°C unless otherwise stated. Chemical shifts (δ) are reported in parts per million (ppm) and referenced $CDCl_3$ (^{1}H: 7.26 ppm; ^{13}C: 77.16 ppm), CD_3OD (^{1}H: 3.31 ppm; ^{13}C: 49.00 ppm), acetic acid-d_4 (^{1}H: 2.04 ppm; ^{13}C: 20.00 ppm), CD_3CN (^{1}H: 1.94 ppm; ^{13}C: 1.32 ppm) or DMSO-d_6 (^{1}H: 2.50 ppm; ^{13}C: 39.52 ppm). Coupling constants (*J*) are given in Hertz (Hz) and refer to apparent multiplicities (s = singlet, d = doublet, t = triplet, q = quartet, quin = quintet, hex = hextet, h = heptet, m = multiplet, br = broad signal, dd = doublet of doublets, etc.). The ^{1}H NMR spectra are reported as follows: chemical shift (multiplicity, coupling constants, number of protons).

High resolution mass spectrometry (HRMS) was performed by the University of Illinois Mass Spectrometry Laboratory. Electrospray ionization (ESI^+) spectra were performed using a time-off-light (TOF) mass analyzer.

Preparatory high-performance liquid chromatography (HPLC) separation was executed on a CombiFlash® EZ preparative HPLC system with a Gemini 5 μm NX-C18 110 Å, LC column.

UV-Visible Spectroscopy were obtained using an SpectraMax® M5 and 10 mm High-quality fluorometer Quartz Cuvettes (Cells) from MSE Supplies.

1.3. Naming of Compounds

Compound names are those generated by PerkinElmer Signals electronic lab notebook, following the IUPAC nomenclature.

2. EXPERIMENTAL DATA

2.1. General Procedures

2.1.1. General Procedure A: Coupling of *N*-nucleophiles with cyclohexylboronic ester 3

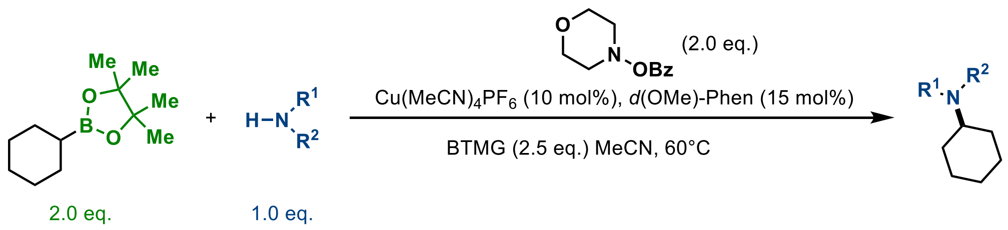

Under an ambient atmosphere, a flame-dried 6 mL reaction vial equipped with a magnetic stir bar was charged with [Cu(MeCN)$_4$]PF$_6$ (7.5 mg, 20 µmol, 10 mol%), 4,7-dimethoxy-1,10-phenanthroline (*d*(OMe)Phen, 7.2 mg, 30 µmol, 15 mol%), the *N*-nucleophile (0.20 mmol, 1.0 equiv.), and morpholino benzoate **1** (83 mg, 0.40 mmol, 2.0 equiv.). The vial was then transferred into an anhydrous, N$_2$-filled glovebox where anhydrous MeCN (2.0 – 4.0 mL, *c* = 0.050 – 0.10 M) was added followed by 2-*tert*-butyl-1,1,3,3-tetramethylguanidine (BTMG) (86 mg, 0.10 mL, 0.50 mmol, 2.5 equiv.) and cyclohexylboronic acid pinacol ester **3** (84 mg, 0.40 mmol, 2.0 equiv.). After sealing vial with a cap with septum, it was removed from the glovebox and stirred at 60°C for 16 hours. The reaction mixture was diluted with EtOAc (2 × 3 mL), filtered through a pad of Celite®, and concentrated *in vacuo.* The residue was finally adsorbed on Celite® and purified by CombiFlash® R$_f$ purification system using RediSep R$_f$ Gold® silica gel (20–40 µm).

2.1.2. General Procedure B: Coupling of 3-chloro1*H*-indazole with alkyl boronic esters

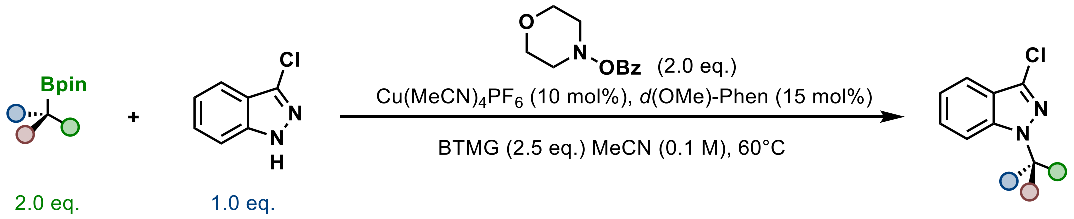

Under an ambient atmosphere, a flame-dried 6 mL reaction vial equipped with a magnetic stir bar was charged with [Cu(MeCN)$_4$]PF$_6$ (7.5 mg, 20 µmol, 10 mol%), 4,7-dimethoxy-1,10-phenanthroline (*d*(OMe)Phen, 7.2 mg, 30 µmol, 15 mol%), 3-chloro-1*H*-indazole (**S6**, 0.20 mmol, 1.0 equiv.) or 6-fluoro-1*H*-indazole-3-carboxylate (**4**, 0.20 mmol, 1.0 equiv.), and morpholino benzoate **1** (83 mg, 0.40 mmol, 2.0 equiv.). The vial was then transferred into an anhydrous, N$_2$-filled glovebox where anhydrous MeCN (2.0 – 4.0 mL, *c* = 0.050 – 0.10 M) was added followed by 2-*tert*-butyl-1,1,3,3-tetramethylguanidine (BTMG) (86 mg, 0.10 mL, 0.50 mmol, 2.5 equiv.) and the alkyl boronic ester (0.40 mmol, 2.0 equiv.). After sealing vial with a cap with septum, it was removed from the glovebox and stirred at 60°C for 16 hours. The reaction mixture was diluted with EtOAc (2 × 3 mL), filtered through a pad of Celite®, and concentrated *in vacuo.* The residue was finally adsorbed on Celite® and purified by CombiFlash® R$_f$ purification system using RediSep R$_f$ Gold® silica gel (20–40 µm).

2.1.3. General Procedure C: Late-stage alkylation

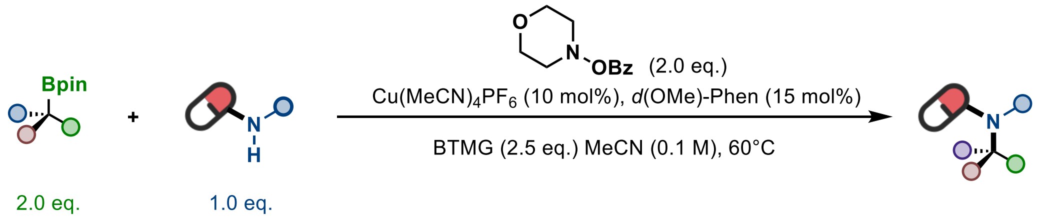

Under an ambient atmosphere, a flame-dried 6 mL reaction vial equipped with a magnetic stir bar was charged with [Cu(MeCN)$_4$]PF$_6$ (7.5 mg, 20 µmol, 10 mol%), 4,7-dimethoxy-1,10-phenanthroline (*d*(OMe)Phen, 7.2 mg, 30 µmol, 15 mol%), the complex *N*-nucleophile (0.20 mmol, 1.0 equiv.), and morpholino benzoate **1** (83 mg, 0.40 mmol, 2.0 equiv.). The vial was then transferred into an anhydrous, N_2-filled glovebox where anhydrous MeCN (4.0 mL, *c* = 0.050 M) was added followed by 2-*tert*-butyl-1,1,3,3-tetramethylguanidine (BTMG) (86 mg, 0.10 mL, 0.50 mmol, 2.5 equiv.) and the alkyl boronic ester (0.40 mmol, 2.0 equiv.). After sealing vial with a cap with septum, it was removed from the glovebox and stirred at 60°C for 16 hours. The reaction mixture was diluted with EtOAc (2 × 3 mL), filtered through a pad of Celite®, and concentrated *in vacuo.* The residue was finally adsorbed on Celite® and purified by CombiFlash® R$_f$ purification system using RediSep R$_f$ Gold® silica gel (20–40 µm).

2.2. Reaction Optimization

2.2.1. Standard procedure for reaction optimization

Under an ambient atmosphere, a flame dried 6 mL reaction vial equipped with a magnetic stir bar was charged with a copper catalyst, a ligand, 6-fluoro-1*H*-indazole-3-carboxylate (40.5 mg, 200 μmol, 1.00 equiv., 96% purity), and morpholino benzoate **1**. The vial was transferred into an anhydrous, N_2-filled glovebox where anhydrous MeCN was added followed by the base and cyclohexylboronic acid pinacol ester **3**. The vial was sealed with a cap with septum, removed from the glovebox. After stirring for 16 hours at 60°C, and methyl 4-flurobenzoate (31.4 mg, 0.200 mmol, 1.00 equiv., 98% purity) as the internal standard was added. After vigorously shaking for 2 min, the yield was determined by ^{19}F NMR integration relative to methyl 4-fluorobenzoate, as the internal standard.

2.2.2 Ligand screening

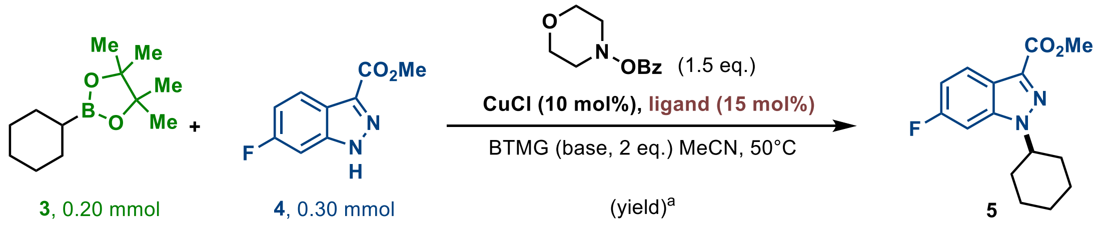

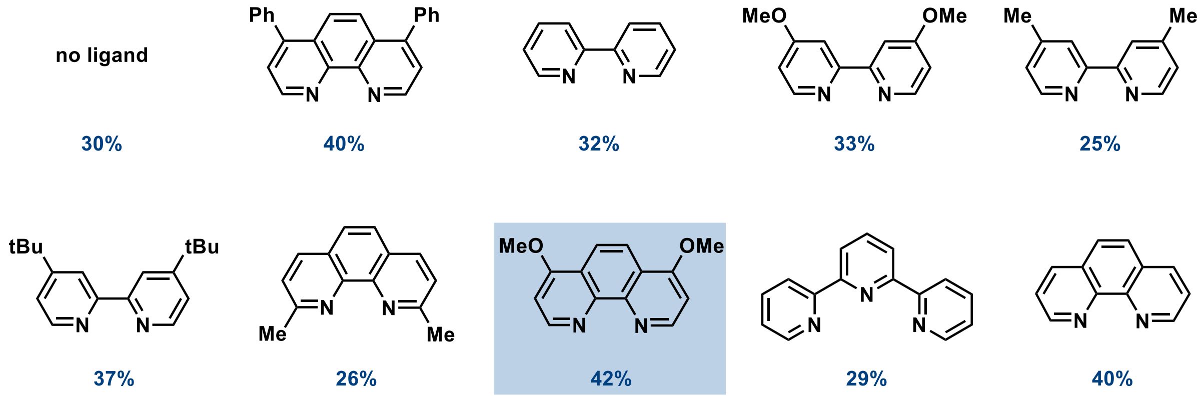

Table S1. Ligand screening

[a] Yield was determined by ^{19}F NMR integration relative to methyl 4-fluorobenzoate (30.8 mg, 0.200 mmol, 1.00 equiv.) as the internal standard.

2.2.3 Copper catalyst screening

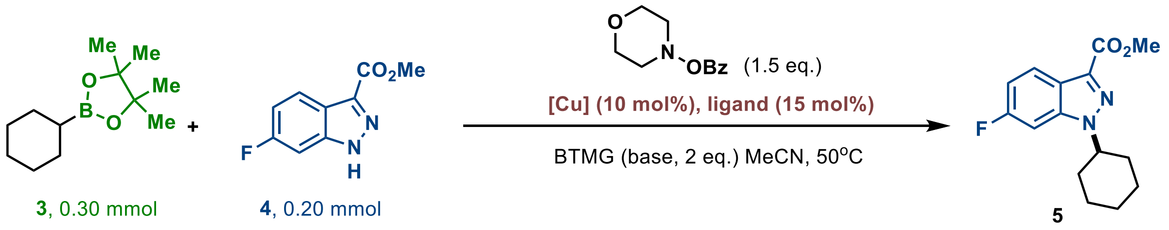

Entry	[Cu] cat.	ligand	NMR yield of 5 (%)

1	CuCl	-	43
2	CuCl	*d*(OMe)-phen	62
3	$CuCl_2$	*d*(OMe)-phen	62
4	$(CuOTf)_2$-PhMe	*d*(OMe)-phen	60
5	$Cu(OTf)_2$	*d*(OMe)-phen	54
**6**	**$Cu(MeCN)_4PF_6$**	***d*(OMe)-phen**	**70**
7	CuI	*d*(OMe)-phen	54
8	$Cu(acac)_2$	*d*(OMe)-phen	28
9	CuTC	*d*(OMe)-phen	64
10	$CuBr_2$-phen (premade catalyst)	*d*(OMe)-phen	49

Table S2. Catalyst screening

[a] Yield was determined by ^{19}F NMR integration relative to methyl 4-fluorobenzoate (30.8 mg, 0.200 mmol, 1.00 equiv.) as the internal standard.

2.2.3 Base screening

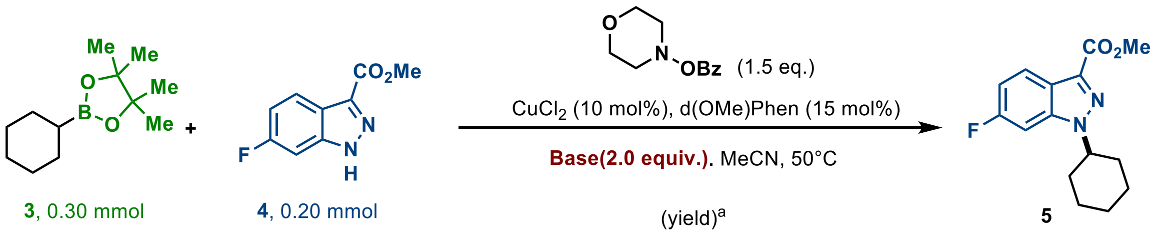

Entry	Base	NMR yield of 5 (%)
**1**	**BTMG**	**62**
2	Cs_2CO_3	56
3	BTTP	40
4	TMG	53

Table S3. Base screening

[a] Yield was determined by ^{19}F NMR integration relative to methyl 4-fluorobenzoate (30.8 mg, 0.200 mmol, 1.00 equiv.) as the internal standard.

2.2.4 Solvent concentration screening

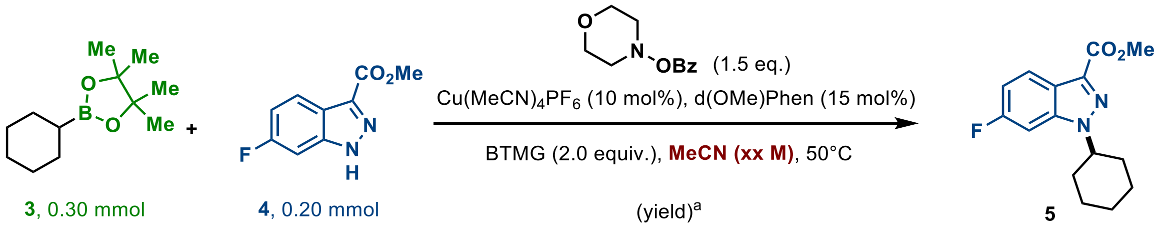

Entry	Conc.	NMR yield of 5 (%)
1	0.20 M	70
**2**	**0.10 M**	**76**
**3**	**0.05 M**	**78**

Table S4. Solvent concentration screening

[a] Yield was determined by ^{19}F NMR integration relative to methyl 4-fluorobenzoate (30.8 mg, 0.200 mmol, 1.00 equiv.) as the internal standard.

2.2.5 Temperature screening

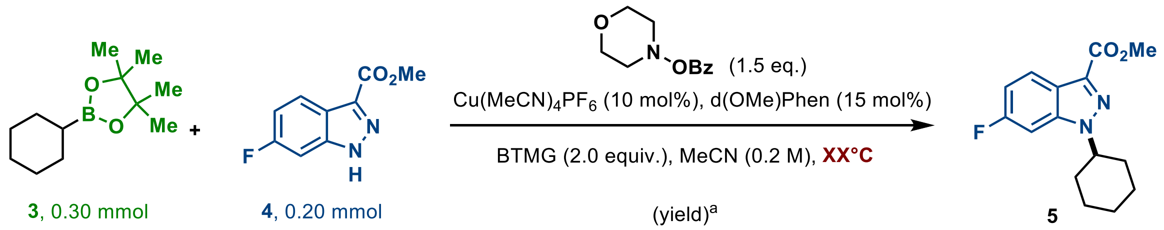

Entry	Temp. (°C)	NMR yield of 5 (%)
1	r.t.	22
2	40	54
3	50	70
**4**	**60**	**74**
**5**	**70**	**74**
6	80	49

Table S5. Temperature screening

[a] Yield was determined by ^{19}F NMR integration relative to methyl 4-fluorobenzoate (30.8 mg, 0.200 mmol, 1.00 equiv.) as the internal standard.

2.2.6 Stoichiometry of BTMG screening

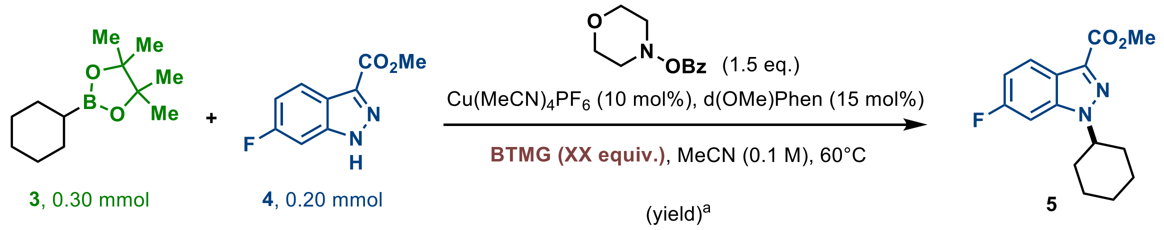

Entry	BTMG (xx equiv.)	NMR yield of 5(%)
1	2.0	76
**2**	**2.5**	**85**
3	3.0	82

Table S6. Temperature screening

[a] Yield was determined by ^{19}F NMR integration relative to methyl 4-fluorobenzoate (30.8 mg, 0.200 mmol, 1.00 equiv.) as the internal standard.

2.2.7 Stoichiometry of 1 and 3 screening

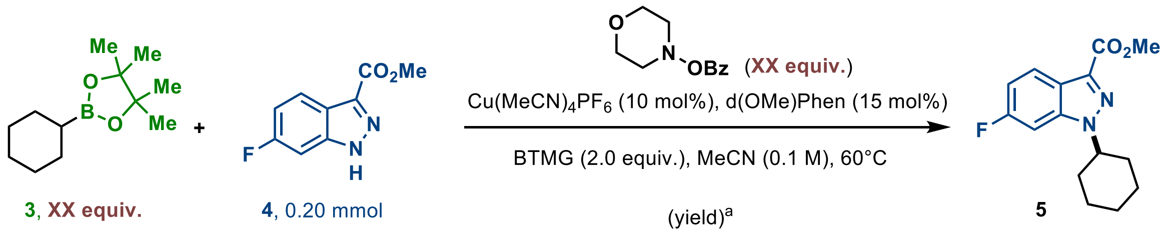

Entry	3 (xx equiv.)	1 (xx equiv.)	NMR yield of 5 (%)
1	1.5	1.5	76
2	1.5	2.0	77
3	1.5	2.5	78
4	2.0	1.5	80
**5**	**2.0**	**2.0**	**87**
**6**	**2.0**	**2.5**	**87**

Table S7. Stoichiometry of 1 and 3 screening

[a] Yield was determined by ^{19}F NMR integration relative to methyl 4-fluorobenzoate (30.8 mg, 0.200 mmol, 1.00 equiv.) as the internal standard.

2.2.8 Activation reagent screening

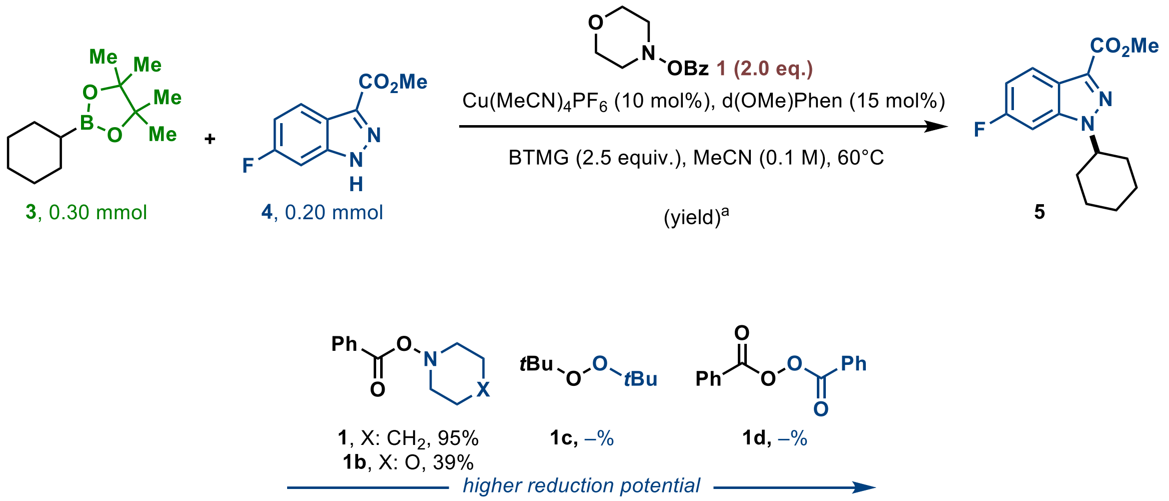

Figure S1. Activation reagent screening

[a] Yield was determined by ^{19}F NMR integration relative to methyl 4-fluorobenzoate (30.8 mg, 0.200 mmol, 1.00 equiv.) as the internal standard.

2.2.9 Boron species screening

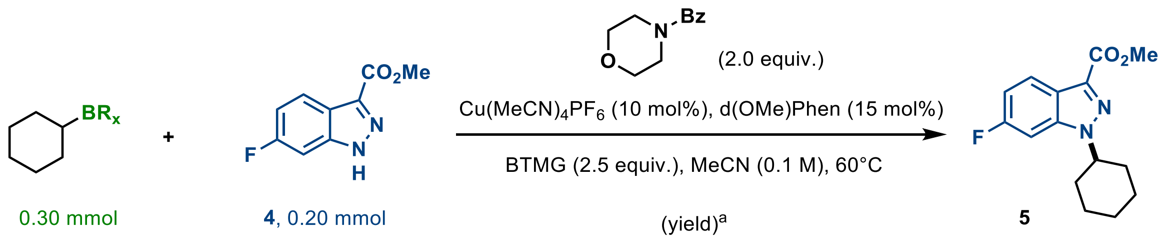

Entry	BRx	NMR yield of 5 (%)
1	$B(OH)_2$[b]	79
**2**	**Bpin**	**95**
3	BF_3K	<5%

Table S8. Boron species screening

[a] Yield was determined by ^{19}F NMR integration relative to methyl 4-fluorobenzoate (30.8 mg, 0.200 mmol, 1.00 equiv.) as the internal standard. [b] performed with cyclohexylboronic acid (2.0 equiv.) and BTMG (5.0 equiv.) instead of **3**.

2.2.10 Control experiments

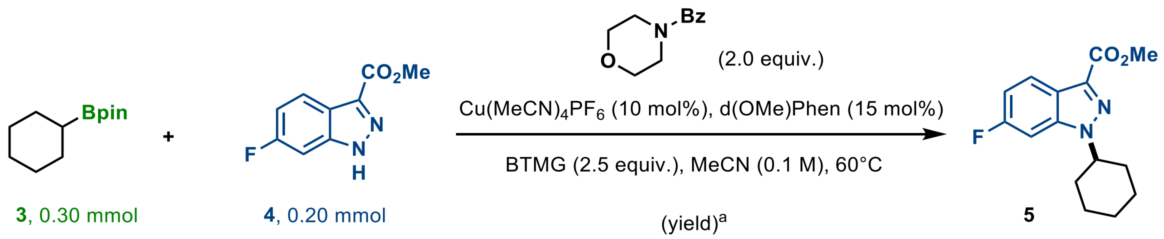

Entry	variations	NMR yield of 5 (%)[a]
1	None	95 (94%)[b]
2	no BTMG	<5
3	no *d*(OMe)Phen	59
4	T = *r.t.* instead of 60°C	36

Table S9. Control experiment

[a] Yield was determined by ^{19}F NMR integration relative to methyl 4-fluorobenzoate (30.8 mg, 0.200 mmol, 1.00 equiv.) as the internal standard. [b] isolated yield.

2.3 Sensitivity Screening

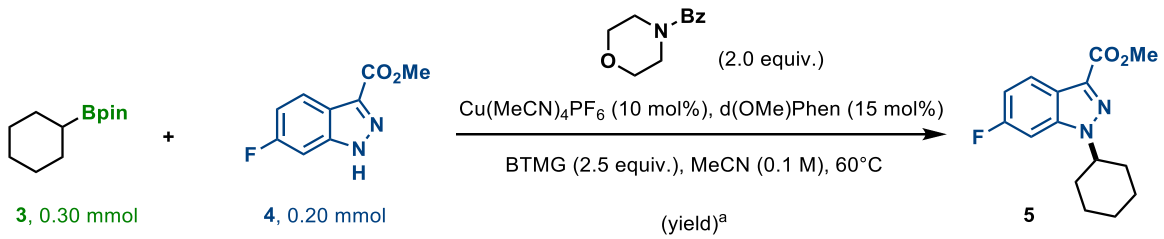

Entry	modification	Deviation from standard conditions	NMR yield of 5 (%)	Deviation from benchmark (%)
1	none	none	95	0
2	high concentration	1 mL MeCN	97	2
3	low concentration	4 mL MeCN	93	-2
4	high H_2O	H_2O (25 µL)	89	-6
5	high O_2	air	86	-9
6	low Cu loading	$Cu(MeCN)_4PF_6$ (2 mol%), *d*(OMe)Phen (5 mol%)	92	3
7	high Cu loading	$Cu(MeCN)_4PF_6$ (20 mol%), *d*(OMe)Phen (25 mol%)	95	0
8	large scale	standard scale x 15 (3.0 mmol)	94 (92)[b]	-1

Table S10. Sensitivity screening

[a] Yield was determined by ^{19}F NMR integration relative to methyl 4-fluorobenzoate (30.8 mg, 0.200 mmol, 1.00 equiv.) as the internal standard. [b] isolated yield.

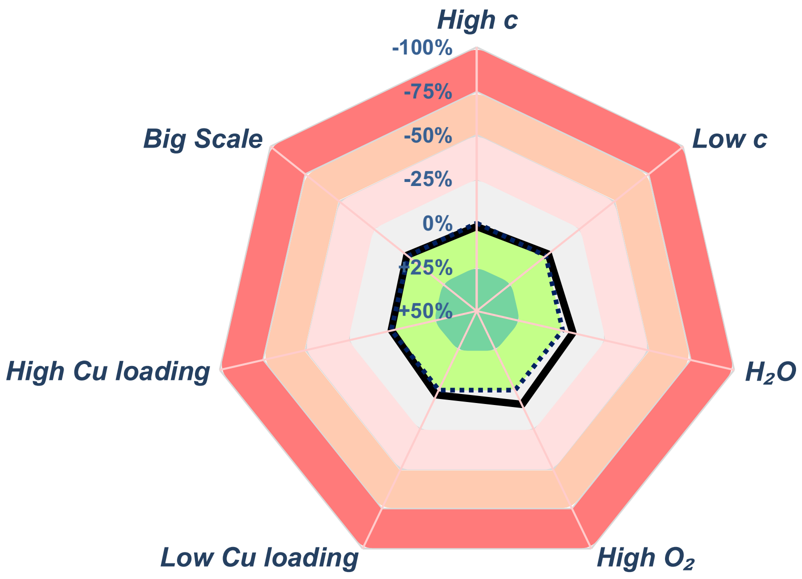

Figure S2: Radar diagram representation of sensitivity screen

2.4. Substrate Scope

Methyl 1-cyclohexyl-6-fluoro-1*H*-indazole-3-carboxylate (5)

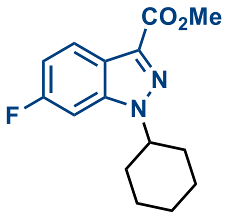

Prepared following **General Procedure A**, using methyl 6-fluoro-1*H*-indazole-3-carboxylate (40.5 mg, 200 μmol, 1.00 equiv., 96% purity), and cyclohexylboronic acid pinacol ester **3** (84 mg, 0.40 mmol, 2.0 equiv.) in MeCN (2.0 mL, *c* = 0.10 M). Purification by CombiFlash® R_f purification system, eluting with EtOAc/heptane (0 – 20%, v/v), gave **5** (52.0 mg, 94%) as a colorless oil.

NMR Spectroscopy (<u>see spectra</u>):

^{1}**H NMR** (400 MHz, $CDCl_3$): δ_H 8.15 (dd, *J* = 8.9, 5.3 Hz, 1H), 7.15 (dd, *J* = 9.1, 2.1 Hz, 1H), 7.04 (td, *J* = 9.0, 2.2 Hz, 1H), 4.38 (tt, *J* = 10.8, 5.1 Hz, 1H), 4.00 (s, 3H), 2.07 (ddt, *J* = 14.5, 10.1, 4.9 Hz, 4H), 1.94 (dt, *J* = 12.7, 3.2 Hz, 2H), 1.75 (ddd, *J* = 13.0, 5.1, 2.6 Hz, 1H), 1.51 – 1.38 (m, 2H), 1.33 (tt, *J* = 12.9, 3.3 Hz, 1H) ppm.

^{13}**C NMR** (101 MHz, $CDCl_3$): δ_C 163.0,162.0 (d, *J* = 246.4 Hz), 140.1 (d, *J* = 12.1 Hz), 134.6, 123.9 (d, *J* = 10.7 Hz), 120.8, 113.1 (d, *J* = 25.8 Hz), 95.6 (d, *J* = 26.5 Hz), 59.7, 52.1, 32.2, 25.7, 25.2 ppm.

^{19}**F NMR** (376 MHz, $CDCl_3$): δ_F –113.73 (d, *J* = 5.4 Hz) ppm.

HRMS (ESI) m/z calc'd for $C_{15}H_{17}FN_2O_2$ $[M+H]^+$, 277.1352; found, 277.1353.

3-Chloro-1-cyclohexyl-1*H*-indazole (6)

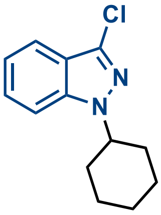

Prepared following **General Procedure A**, using 3-chloro-1*H*-indazole (32.1 mg, 200 μmol, 1.00 equiv., 95% purity), and cyclohexylboronic acid pinacol ester **3** (84 mg, 0.40 mmol, 2.0 equiv.) in MeCN (2.0 mL, *c* = 0.10 M). Purification by CombiFlash® R_f purification system, eluting with EtOAc/heptane (0 – 20%, v/v), gave **6** (43.8 mg, 93%) as a colorless oil.

NMR Spectroscopy (<u>see spectra</u>):

^{1}**H NMR** (400 MHz, $CDCl_3$): δ_H 7.66 (d, *J* = 8.2 Hz, 1H), 7.46 – 7.35 (m, 2H), 7.17 (ddd, *J* = 7.9, 6.2, 1.4 Hz, 1H), 4.35 (ddd, *J* = 15.7, 8.5, 6.0 Hz, 1H), 2.03 (dt, *J* = 9.7, 4.8 Hz, 4H), 1.95 (dt, *J* = 12.4, 2.9 Hz, 2H), 1.76 (dt, *J* = 13.1, 3.3 Hz, 1H), 1.46 (m, 2H), 1.38 – 1.23 (m, 1H) ppm.

^{13}**C NMR** (101 MHz, $CDCl_3$): δ_C 140.1, 132.3, 127.1, 121.12, 121.06, 119.9, 109.5, 58.6, 32.6, 25.9, 25.4

ppm.

All recorded spectroscopic data matched those previously reported in the literature.[1]

1-Cyclohexyl-5-(trifluoromethoxy)-1*H*-indazole (7)

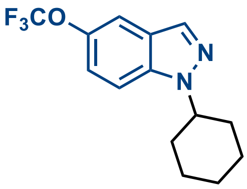

Prepared following **General Procedure A**, using 5-(trifluoromethoxy)-1*H*-indazole (42.1 mg, 200 μmol, 1.00 equiv., 96% purity), and cyclohexylboronic acid pinacol ester **3** (84 mg, 0.40 mmol, 2.0 equiv.) in MeCN (2.0 mL, *c* = 0.10 M). Purification by CombiFlash® R_f purification system, eluting with EtOAc/heptane (0 – 20%, v/v), gave **7** (47.8 mg, 84%) as a colorless oil.

NMR Spectroscopy (*see spectra*):

^{1}H NMR (400 MHz, $CDCl_3$): δ_H 8.00 (s, 1H), 7.62 – 7.53 (m, 1H), 7.45 (d, *J* = 9.1 Hz, 1H), 7.23 (dd, *J* = 9.1, 2.2 Hz, 1H), 4.39 (dt, *J* = 10.8, 5.6 Hz, 1H), 2.10 – 1.92 (m, 6H), 1.78 (dt, J = 12.7, 3.4 Hz, 1H), 1.56 – 1.40 (m, 2H), 1.39 – 1.26 (m, 1H) ppm.

^{13}C NMR (101 MHz, $CDCl_3$): δ_C 143.2, 137.1, 132.8, 123.8, 120.9 (d, *J* = 257.6 Hz, 1H), 120.5, 113.2, 110.2, 58.5, 32.7, 25.9, 25.5 ppm.

^{19}F NMR (376 MHz, $CDCl_3$): δ_F –63.62 (s) ppm.

HRMS (ESI) m/z calc'd for $C_{14}H_{16}N_2OF_3$ $[M+H]^+$, 285.1215; found, 285.1215.

1-Cyclohexyl-1*H*-indazole-5-carboxylic acid (8)

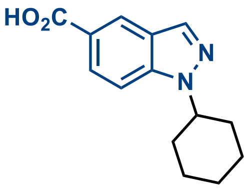

Prepared following **General Procedure A**, using 1*H*-Indazole-5-carboxylic acid (45.8 mg, 200 μmol, 1.00 equiv., 97% purity), and cyclohexylboronic acid pinacol ester **3** (84 mg, 0.40 mmol, 2.0 equiv.) in MeCN (2.0 mL, *c* = 0.10 M). Purification by CombiFlash® R_f purification system, eluting with DCM/MeOH (0 – 50%, v/v), gave **8** (29.9 mg, 61%) as a colorless solid.

NMR Spectroscopy (*see spectra*):

^{1}H NMR (400 MHz, $CDCl_3$): δ_H 8.61 (s, 1H), 8.15 (s, 1H), 8.10 (dd, *J* = 8.8, 1.5 Hz, 1H), 7.50 (d, *J* = 8.9 Hz, 1H), 4.44 (p, *J* = 8.1 Hz, 1H), 2.10 – 1.95 (m, 5H), 1.84 – 1.75 (m, 1H), 1.58 – 1.42 (m, 2H), 1.42 – 1.31 (m, 1H) ppm.

^{13}C NMR (101 MHz, $CDCl_3$): δ_C 171.9, 140.8, 134.7, 127.1, 126.0, 123.8, 121.8, 109.1, 58.5, 32.7, 25.9,

25.5 ppm.

HRMS (ESI) m/z calc'd for $C_{14}H_{17}N_2O_2$ [M+H]$^+$, 245.1290; found, 245.1285.

6-Bromo-1-cyclohexyl-1*H*-indazole-3-carbonitrile (9)

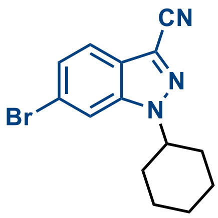

Prepared following **General Procedure A**, using ***copper(I) thiophene-2-carboxylate*** (3.8 mg, 20 µmol, 10 mol%), 6-bromo-1*H*-indazole-3-carbonitrile (45.8 mg, 200 µmol, 1.00 equiv., 97% purity), and cyclohexylboronic acid pinacol ester **3** (84 mg, 0.40 mmol, 2.0 equiv.) in MeCN (4.0 mL, *c* = 0.050 M). Purification by CombiFlash® R_f purification system, eluting with EtOAc/heptane (0 – 20%, v/v), gave **9** (43.2 mg, 71%) as a colorless oil.

NMR Spectroscopy (<u>see spectra</u>):

^{1}H NMR (400 MHz, $CDCl_3$): δ_H 7.75 (s, 1H), 7.69 (d, *J* = 7.2 Hz, 1H), 7.43 (dd, *J* = 8.6, 1.5 Hz, 1H), 4.41 (dt, *J* = 10.8, 5.6 Hz, 1H), 2.17 – 1.89 (m, 6H), 1.80 (dd, *J* = 12.5, 3.8 Hz, 1H), 1.56 – 1.40 (m, 2H), 1.34 (dt, *J* = 12.8, 3.4 Hz, 1H) ppm.

^{13}C NMR (101 MHz, $CDCl_3$): δ_C 139.7, 127.3, 124.3, 122.0, 121.0, 117.8, 113.5, 113.3, 59.8, 32.6, 25.6, 25.2 ppm.

HRMS (ESI) m/z calc'd for $C_{14}H_{15}N_3Br$ [M+H]$^+$, 304.0449; found, 304.0447.

Methyl 1-cyclohexyl-1*H*-pyrazolo[4,3-b]pyridine-5-carboxylate (10)

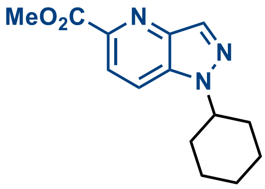

Prepared following **General Procedure A**, using methyl 1*H*-pyrazolo[4,3-b]pyridine-5-carboxylate (36.5 mg, 200 µmol, 1.00 equiv., 97% purity), and cyclohexylboronic acid pinacol ester **3** (84 mg, 0.40 mmol, 2.0 equiv.) in MeCN (2.0 mL, *c* = 0.10 M). Purification by CombiFlash® R_f purification system, eluting with EtOAc/heptane (0 – 40%, v/v), gave **10** (45.3 mg, 87%) as a colorless oil.

NMR Spectroscopy (<u>see spectra</u>):

^{1}H NMR (400 MHz, $CDCl_3$): δ_H 8.35 (s, 1H), 8.13 (d, *J* = 8.9 Hz, 1H), 7.87 (dd, *J* = 8.9, 1.0 Hz, 1H), 4.40 (dt, *J* = 10.8, 5.6 Hz, 1H), 4.02 (s, 3H), 2.07 – 2.03 (m, 2H), 2.03 – 1.90 (m, 4H), 1.76 (dt, *J* = 12.9, 3.4 Hz, 1H), 1.53 – 1.19 (m, 3H) ppm.

^{13}C NMR (101 MHz, $CDCl_3$): δ_C 166.1, 143.3, 141.8, 135.1, 132.2, 121.5, 117.3, 59.2, 53.1, 32.5, 25.7, 25.3 ppm.

HRMS (ESI) m/z calc'd for $C_{14}H_{18}N_3O_2$ $[M+H]^+$, 260.1399; found, 260.1394.

5-Bromo-1-cyclohexyl-1*H*-pyrazolo[3,4-c]pyridine (11)

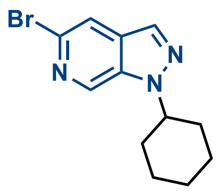

Prepared following **General Procedure A**, using ***copper(I) thiophene-2-carboxylate*** (3.8 mg, 20 μmol, 10 mol%), 5-bromo-1*H*-pyrazolo[3,4-c]pyridine (40.8 mg, 200 μmol, 1.00 equiv., 97% purity), and cyclohexylboronic acid pinacol ester **3** (84 mg, 0.40 mmol, 2.0 equiv.) in MeCN (4.0 mL, *c* = 0.050 M). Purification by CombiFlash® R_f purification system, eluting with EtOAc/heptane (0 – 60%, v/v), gave **11** (28.3 mg, 51%) as a colorless oil.

NMR Spectroscopy (*see spectra*):

^{1}**H NMR** (400 MHz, $CDCl_3$): δ_H 8.75 (s, 1H), 7.96 (s, 1H), 7.78 (d, *J* = 1.2 Hz, 1H), 4.47 (dt, *J* = 10.8, 5.6 Hz, 1H), 2.21 – 1.91 (m, 6H), 1.78 (td, *J* = 7.1, 3.6 Hz, 1H), 1.50 (qt, *J* = 12.8, 4.1 Hz, 2H), 1.34 (tt, *J* = 12.7, 3.5 Hz, 1H) ppm.

^{13}**C NMR** (101 MHz, $CDCl_3$): δ_C 135.2, 133.4, 131.3, 130.8, 129.7, 118.4, 59.5, 32.8, 25.7, 25.4 ppm.

HRMS (ESI) m/z calc'd for $C_{12}H_{15}N_3Br$ $[M+H]^+$, 280.0449; found, 280.0447.

6-Chloro-1-cyclohexyl-1*H*-pyrazolo[3,4-b]pyridine (12)

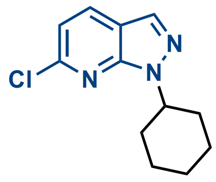

Prepared following **General Procedure A**, using methyl 6-chloro-1*H*-pyrazolo[3,4-b]pyridine (32.0 mg, 200 μmol, 1.00 equiv., 96% purity), and cyclohexylboronic acid pinacol ester **3** (84 mg, 0.40 mmol, 2.0 equiv.) in MeCN (2.0 mL, *c* = 0.10 M). Purification by CombiFlash® R_f purification system, eluting with EtOAc/heptane (0 – 40%, v/v), gave **12** (35.9 mg, 76%) as a colorless oil.

NMR Spectroscopy (*see spectra*):

^{1}**H NMR** (400 MHz, $CDCl_3$): δ_H 7.98 (s, 1H). 7.96 (m, 1H), 7.10 (d, J = 8.3 Hz, 1H), 4.85 (dt, *J* = 10.8, 5.6 Hz, 1H), 2.02 (td, J = 9.7, 3.6 Hz, 4H), 1.97 – 1.87 (m, 2H), 1.76 (dt, J = 13.1, 3.4 Hz, 1H), 1.53 (dqd, J = 13.1, 8.6, 4.4 Hz, 1H), 1.41 – 1.24 (m, 2H) ppm.

^{13}**C NMR** (101 MHz, $CDCl_3$): δ_C 150.2, 148.8, 132.1, 131.9, 117.4, 114.2, 56.0, 32.6, 25.7, 25.5 ppm.

HRMS (ESI) m/z calc'd for $C_{12}H_{15}N_3Cl$ $[M+H]^+$, 236.0955; found, 236.0951.

6-Chloro-1-cyclohexyl-1*H*-indole (13)

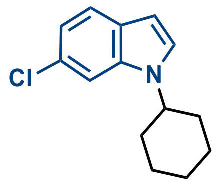

Prepared following **General Procedure A**, using 6-chloro-1*H*-indole (31.6 mg, 200 µmol, 1.00 equiv., 96% purity), and cyclohexylboronic acid pinacol ester **3** (84 mg, 0.40 mmol, 2.0 equiv.) in MeCN (2.0 mL, *c* = 0.10 M). Purification by CombiFlash® R_f purification system, eluting with EtOAc/heptane (0 – 20%, v/v), gave **13** (40.5 mg, 87%) as a colorless oil.

NMR Spectroscopy (*see spectra*):

^{1}**H NMR** (400 MHz, $CDCl_3$): δ_H 7.54 (d, *J* = 8.4 Hz, 1H), 7.39 (d, *J* = 1.8 Hz, 1H), 7.22 (d, *J* = 3.3 Hz, 1H), 7.08 (dd, *J* = 8.4, 1.9 Hz, 1H), 6.49 (d, *J* = 3.2 Hz, 1H), 4.15 (tt, *J* = 11.8, 3.6 Hz, 1H), 2.20 – 2.06 (m, 2H), 1.96 (dt, J = 13.5, 3.4 Hz, 2H), 1.82 (ddt, J = 11.3, 5.6, 1.9 Hz, 1H), 1.70 (qd, J = 12.5, 3.4 Hz, 2H), 1.60 – 1.44 (m, 2H), 1.38 – 1.25 (m, 1H).

^{13}**C NMR** (101 MHz, $CDCl_3$): δ_C 136.1, 127.3, 127.1, 124.9, 121.8, 120.0, 109.6, 101.4, 55.4, 33.6, 26.0, 25.7 ppm.

All recorded spectroscopic data matched those previously reported in the literature.[1]

1-(1-Cyclohexyl-1*H*-indol-6-yl)ethan-1-one (14)

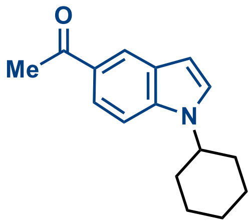

Prepared following **General Procedure A**, using 1-(1*H*-indol-6-yl)ethan-1-one (32.5 mg, 200 µmol, 1.00 equiv., 98% purity), and cyclohexylboronic acid pinacol ester **3** (84 mg, 0.40 mmol, 2.0 equiv.) in MeCN (2.0 mL, *c* = 0.10 M). Purification by CombiFlash® R_f purification system, eluting with EtOAc/heptane (0 – 30%, v/v), gave **14** (33.1 mg, 69%) as a colorless solid.

NMR Spectroscopy (*see spectra*):

^{1}**H NMR** (400 MHz, $CDCl_3$): δ_H 8.11 (d, *J* = 1.4 Hz, 1H), 7.75 – 7.61 (m, 2H), 7.40 (d, *J* = 3.2 Hz, 1H), 6.55 (dd, *J* = 3.2, 0.9 Hz, 1H), 4.35 (dt, *J* = 11.8, 3.7 Hz, 1H), 2.69 (s, 3H), 2.19 – 2.08 (m, 2H), 1.95 (dt, *J* = 13.5, 3.4 Hz, 2H), 1.87 – 1.64 (m, 3H), 1.54 (qt, *J* = 13.2, 3.4 Hz, 2H), 1.38 – 1.23 (m, 1H) ppm.

^{13}**C NMR** (101 MHz, $CDCl_3$): δ_C 198.8, 135.3, 132.4, 130.7, 128.2, 120.5, 120.1, 110.2, 101.6, 55.2, 33.9, 27.1, 26.0, 25.7 ppm.

HRMS (ESI) m/z calc'd for $C_{16}H_{20}NO$ $[M+H]^+$, 242.1545; found, 242.1544.

1-Cyclohexyl-1*H*-indole-3-carboxamide (15)

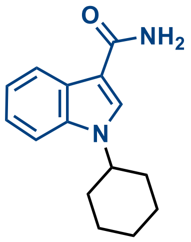

Prepared following **General Procedure A**, using 1H-indole-3-carboxamide (32.4 mg, 200 µmol, 1.00 equiv., 99% purity), and cyclohexylboronic acid pinacol ester **3** (84 mg, 0.40 mmol, 2.0 equiv.) in MeCN (2.0 mL, *c* = 0.10 M). Purification by CombiFlash® R_f purification system, eluting with MeOH/DCM (0 – 10%, v/v), gave **15** (38.6 mg, 80%) as a colorless solid.

NMR Spectroscopy (*see spectra*):

^{1}H NMR (400 MHz, $CDCl_3$): δ_H 7.95 (brs, 2H), 7.46 (dt, *J* = 4.6, 2.9 Hz, 1H), 7.33 – 7.27 (m, 2H), 5.93 (brs, 2H), 4.27 (tt, *J* = 11.8, 3.6 Hz, 1H), 2.27 – 2.13 (m, 2H), 2.02 – 1.94 (m, 3H), 1.84 (dd, J = 13.1, 3.9 Hz, 1H), 1.73 (qd, J = 12.4, 3.4 Hz, 2H), 1.54 (ddt, J = 16.5, 13.2, 6.6 Hz, 2H), 1.39 – 1.24 (m, 1H) ppm.

^{13}C NMR (101 MHz, $CDCl_3$): δ_C 167.6, 136.5, 129.6, 125.6, 122.5, 121.9, 120.4, 110.6, 109.6, 55.7, 33.6, 25.9, 25.6 ppm.

HRMS (ESI) m/z calc'd for $C_{13}H_{16}N_2Cl$ $[M+H]^+$, 235.1002; found, 235.1001.

Methyl *N*-(tert-butoxycarbonyl)-1-cyclohexyl-*L*-tryptophanate (16)

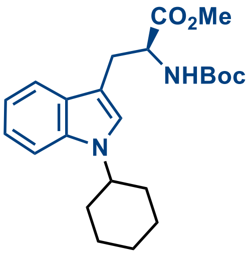

Prepared following **General Procedure A**, using methyl *N*-Boc-*L*-tryptophan methyl ester (65.6 mg, 200 µmol, 1.00 equiv., 97% purity), and cyclohexylboronic acid pinacol ester **3** (84 mg, 0.40 mmol, 2.0 equiv.) in MeCN (4.0 mL, *c* = 0.050 M). Purification by CombiFlash® R_f purification system, eluting with EtOAc/heptane (0 – 60%, v/v), gave **16** (66.0 mg, 82%) as a colorless solid.

NMR Spectroscopy (*see spectra*):

^{1}H NMR (400 MHz, $CDCl_3$): δ_H 7.55 (d, *J* = 7.9 Hz, 1H), 7.35 (d, *J* = 8.2 Hz, 1H), 7.20 (ddd, *J* = 8.2, 6.9, 1.2 Hz, 1H), 7.15 – 7.07 (m, 1H), 7.03 (s, 1H), 5.06 (d, *J* = 8.4 Hz, 1H), 4.66 (dd, *J* = 8.4, 5.2 Hz, 1H), 4.18 (dt, *J* = 11.8, 3.7 Hz, 1H), 3.69 (s, 3H), 3.28 (t, *J* = 5.3 Hz, 2H), 2.19 – 2.05 (m, 2H), 2.00 – 1.90 (m, 2H), 1.86 – 1.76 (m, 1H), 1.68 (qd, *J* = 12.1, 4.3 Hz, 2H), 1.56 – 1.23 (m, 4H), 1.44 (s, 9H) ppm.

^{13}C NMR (101 MHz, $CDCl_3$): δ_C 173.0, 155.3, 135.9, 128.2, 122.9, 121.4, 119.2, 119.0, 109.6, 108.8, 79.8, 55.1, 54.4, 52.3, 33.7, 33.6, 28.5, 28.3, 26.0, 25.7 ppm.

HRMS (ESI) m/z calc'd for $C_{23}H_{33}N_2O_4$ [M+H]$^+$, 401.2440; found, 401.2434.

5-Chloro-1-cyclohexyl-1*H*-pyrrolo[3,2-b]pyridine (17)

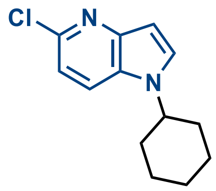

Prepared following **General Procedure A**, using 5-chloro-1*H*-pyrrolo[3,2-b]pyridine (30.8 mg, 200 µmol, 1.00 equiv., 99% purity), and cyclohexylboronic acid pinacol ester **3** (84 mg, 0.40 mmol, 2.0 equiv.) in MeCN (2.0 mL, *c* = 0.10 M). Purification by CombiFlash® R_f purification system, eluting with EtOAc/heptane (0 – 60%, v/v), gave **17** (39.8 mg, 85%) as a colorless oil.

NMR Spectroscopy (see spectra):

^{1}**H NMR** (400 MHz, $CDCl_3$): δ_H 7.61 (dd, *J* = 8.6, 0.8 Hz, 1H), 7.44 (d, *J* = 3.4 Hz, 1H), 7.07 (d, *J* = 8.6 Hz, 1H), 6.61 (dd, *J* = 3.3, 0.8 Hz, 1H), 4.13 (tt, *J* = 11.9, 3.7 Hz, 1H), 2.09 (dtd, *J* = 12.1, 3.8, 2.0 Hz, 2H), 1.94 (dt, *J* = 13.6, 3.5 Hz, 2H), 1.84 – 1.63 (m, 3H), 1.48 (qt, *J* = 13.1, 3.4 Hz, 2H), 1.29 (tt, *J* = 13.0, 3.6 Hz, 1H).

^{13}**C NMR** (101 MHz, $CDCl_3$): δ_C 146.3, 143.9, 128.7, 127.3, 119.4, 116.0, 102.0, 56.1, 33.6, 25.9, 25.5 ppm.

HRMS (ESI) m/z calc'd for $C_{13}H_{16}N_2Cl$ [M+H]$^+$, 235.1002; found, 235.1001.

4-Chloro-1-cyclohexyl-1*H*-pyrrolo[2,3-c]pyridine (18)

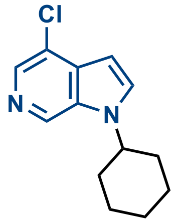

Prepared following **General Procedure A**, using 4-chloro-1*H*-pyrrolo[2,3-c]pyridine (31.5 mg, 200 µmol, 1.00 equiv., 97% purity), and cyclohexylboronic acid pinacol ester **3** (84 mg, 0.40 mmol, 2.0 equiv.) in MeCN (2.0 mL, *c* = 0.10 M). Purification by CombiFlash® R_f purification system, eluting with EtOAc/heptane (0 – 60%, v/v), gave **18** (40.5 mg, 86%) as a colorless oil.

NMR Spectroscopy (see spectra):

^{1}**H NMR** (400 MHz, $CDCl_3$): δ_H 8.70 (brs, 1H), 8.21 (brs, 1H), 7.39 (d, *J* = 3.2 Hz, 1H), 6.60 (d, *J* = 3.1 Hz, 1H), 4.28 (tt, *J* = 11.9, 3.6 Hz, 1H), 2.20 – 2.11 (m, 2H), 2.01 – 1.91 (m, 2H), 1.86 – 1.66 (m, 3H), 1.59 – 1.44 (m, 2H), 1.36 – 1.24 (m, 1H) ppm.

^{13}**C NMR** (101 MHz, $CDCl_3$): δ_C 136.9, 132.1, 131.3, 128.6, 99.6, 56.6, 33.8, 25.9, 25.5 ppm.

HRMS (ESI) m/z calc'd for $C_{13}H_{16}N_2Cl$ [M+H]$^+$, 235.1002; found, 235.1004.

3-Bromo-4-chloro-1-cyclohexyl-1*H*-pyrrolo[2,3-*b*]pyridine (19)

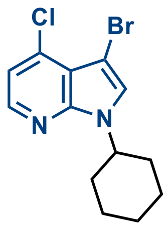

Prepared following **General Procedure A**, using ***copper(I) thiophene-2-carboxylate*** (3.8 mg, 20 µmol, 10 mol%), 3-bromo-4-chloro-1*H*-pyrrolo[2,3-b]pyridine (48.2 mg, 200 µmol, 1.00 equiv., 96% purity), and cyclohexylboronic acid pinacol ester **3** (84 mg, 0.40 mmol, 2.0 equiv.) in MeCN (4.0 mL, *c* = 0.050 M). Purification by CombiFlash® R_f purification system, eluting with EtOAc/heptane (0 – 60%, v/v), gave **19** (38.9 mg, 62%) as a colorless oil.

NMR Spectroscopy (see spectra):

^{1}H NMR (400 MHz, $CDCl_3$): δ_H 8.17 (d, *J* = 5.1 Hz, 1H), 7.35 (s, 1H), 7.07 (d, *J* = 5.1 Hz, 1H), 4.78 (tt, *J* = 11.9, 3.6 Hz, 1H), 2.13 – 2.03 (m, 2H), 1.91 (dt, *J* = 13.5, 3.4 Hz, 2H), 1.78 (dtt, *J* = 11.6, 3.3, 1.6 Hz, 1H), 1.70 – 1.59 (m, 4H), 1.33 – 1.21 (m, 1H) ppm.

^{13}C NMR (101 MHz, $CDCl_3$): δ_C 146.9, 143.5, 136.4, 125.9, 117.7, 116.3, 86.2, 53.9, 33.7, 25.8, 25.6 ppm.

HRMS (ESI) m/z calc'd for $C_{13}H_{15}N_2ClBr$ $[M+H]^+$, 313.0107; found, 313.0106.

1-Cyclohexyl-4-(4,4,5,5-tetramethyl-1,3,2-dioxaborolan-2-yl)-1*H*-pyrazole (20)

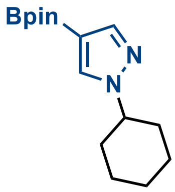

Prepared following **General Procedure A**, using 4-pyrazoleboronic acid pinacol ester (40.0 mg, 200 µmol, 1.00 equiv., 97% purity), and cyclohexylboronic acid pinacol ester **3** (84 mg, 0.40 mmol, 2.0 equiv.) in MeCN (4.0 mL, *c* = 0.050 M). Purification by CombiFlash® R_f purification system, eluting with EtOAc/heptane (0 – 40%, v/v), gave **20** (40.3 mg, 73%) as a colorless oil.

NMR Spectroscopy (see spectra):

^{1}H NMR (400 MHz, $CDCl_3$): δ_H 7.77 (s, 1H), 7.73 (s, 1H), 4.11 (tt, *J* = 11.9, 3.6 Hz, 1H), 2.24 – 2.11 (m, 2H), 1.87 (dt, *J* = 13.5, 3.5 Hz, 2H), 1.71 (td, *J* = 12.1, 3.7 Hz, 4H), 1.48 – 1.17 (m, 2H), 1.31 (s, 12H) ppm.

^{13}C NMR (101 MHz, $CDCl_3$): δ_C 144.9, 133.5, 83.3, 61.2, 33.6, 25.5, 25.5, 24.9 ppm.

^{11}B NMR (128 MHz, $CDCl_3$): δ_C 28.71 (brs) ppm.

All recorded spectroscopic data matched those previously reported in the literature.[2]

1-Cyclohexyl-3-phenyl-1*H*-pyrazole (21)

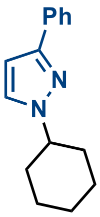

Prepared following **General Procedure A**, using 3-phenyl-1*H*-pyrazole (29.4 mg, 200 µmol, 1.00 equiv., 98% purity), and cyclohexylboronic acid pinacol ester **3** (84 mg, 0.40 mmol, 2.0 equiv.) in MeCN (2.0 mL, *c* = 0.10 M). Purification by CombiFlash® R_f purification system, eluting with EtOAc/heptane (0 – 20%, v/v), gave **21** (39.8 mg, 88%) as a colorless oil.

NMR Spectroscopy (*see spectra*):

^{1}**H NMR** (400 MHz, $CDCl_3$): δ_H 7.81 – 7.76 (m, 2H), 7.44 – 7.33 (m, 3H), 7.31 – 7.22 (m, 1H), 6.52 (d, *J* = 2.3 Hz, 1H), 4.15 (tt, *J* = 11.8, 3.6 Hz, 1H), 2.26 – 2.16 (m, 2H), 1.90 (dt, *J* = 13.5, 3.5 Hz, 2H), 1.78 – 1.66 (m, 3H), 1.43 (qt, *J* = 12.9, 3.4 Hz, 2H), 1.27 (ddd, *J* = 16.4, 8.3, 3.6 Hz, 1H) ppm.

^{13}**C NMR** (101 MHz, $CDCl_3$): δ_C 150.6, 134.0, 128.6, 127.6, 127.4, 125.7, 102.3, 61.4, 33.8, 25.53, 25.52 ppm.

All recorded spectroscopic data matched those previously reported in the literature.[1]

3-(3-Bromophenyl)-1-cyclohexyl-1*H*-pyrazole (22)

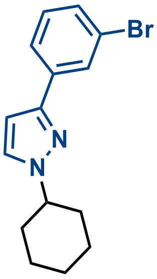

Prepared following **General Procedure A**, using ***copper(I) thiophene-2-carboxylate*** (3.8 mg, 20 µmol, 0.10 equiv.), 3-(3-bromophenyl)-1H-pyrazole (45.5 mg, 200 µmol, 1.00 equiv., 98% purity), and cyclohexylboronic acid pinacol ester **3** (84 mg, 0.40 mmol, 2.0 equiv.) in MeCN (4.0 mL, *c* = 0.050 M). Purification by CombiFlash® R_f purification system, eluting with EtOAc/heptane (0 – 40%, v/v), gave **22** (48.1 mg, 79%) as a colorless oil.

NMR Spectroscopy (*see spectra*):

^{1}**H NMR** (400 MHz, $CDCl_3$): δ_H 7.98 (s, 1H), 7.71 (dt, *J* = 7.8, 1.3 Hz, 1H), 7.47 – 7.36 (m, 2H), 7.24 (t, *J* = 7.9 Hz, 1H), 6.51 (d, *J* = 2.4 Hz, 1H), 4.15 (tt, *J* = 11.8, 7.9 Hz, 1H), 2.26 – 2.16 (m, 2H), 1.91 (dq, *J* = 10.3, 3.5 Hz, 2H), 1.81 – 1.66 (m, 3H), 1.53 – 1.38 (m, 2H), 1.36 – 1.22 (m, 1H) ppm.

^{13}**C NMR** (101 MHz, $CDCl_3$): δ_C 149.2, 136.1, 130.3, 130.2, 128.6, 127.9, 124.2, 122.9, 102.5, 61.6, 33.7, 25.51, 25.50 ppm.

HRMS (ESI) m/z calc'd for $C_{15}H_{18}N_2Br$ $[M+H]^+$, 305.0653; found, 305.0650.

1-Cyclohexyl-3-(4-fluorophenyl)-1*H*-pyrazole-4-carbaldehyde (23)

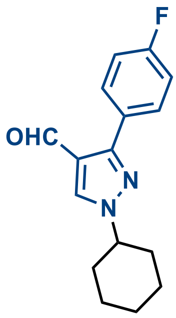

Prepared following **General Procedure A**, using 3-(4-fluorophenyl)-1*H*-pyrazole-4-carbaldehyde (38.8 mg, 200 µmol, 1.00 equiv., 98% purity), and cyclohexylboronic acid pinacol ester **3** (84 mg, 0.40 mmol, 2.0 equiv.) in MeCN (2.0 mL, *c* = 0.10 M). Purification by CombiFlash® R_f purification system, eluting with EtOAc/heptane (0 – 20%, v/v), gave **23** (34.7 mg, 64%) as a colorless oil.

NMR Spectroscopy (*see spectra*):

^{1}**H NMR** (400 MHz, $CDCl_3$): δ_H 9.90 (s, 1H, CHO), 8.04 (s, 1H), 7.79 – 7.71 (m, 2H), 7.14 (t, *J* = 8.7 Hz, 2H), 4.16 (tt, *J* = 11.7, 3.8 Hz, 1H), 2.29 – 2.18 (m, 2H), 1.93 (dt, *J* = 13.5, 3.6 Hz, 2H), 1.81 – 1.66 (m, 3H), 1.45 (qt, *J* = 13.0, 3.4 Hz, 2H), 1.28 (ddt, *J* = 16.5, 12.8, 6.3 Hz, 1H) ppm.

^{13}**C NMR** (101 MHz, $CDCl_3$): δ_C 184.8, 163.4 (d, *J* = 248.4 Hz), 152.3, 131.9, 130.7, 128.2 (d, *J* = 3.3 Hz), 120.5, 115.7 (d, *J* = 21.6 Hz), 62.1, 33.4, 25.3, 25.2 ppm.

^{19}**F NMR** (376 MHz, $CDCl_3$): δ_F –112.63 (s) ppm.

HRMS (ESI) m/z calc'd for $C_{16}H_{18}N_2OF$ $[M+H]^+$, 273.1403; found, 273.1396.

(1-Cyclohexyl-1*H*-pyrazol-3-yl)(phenyl)methanone (24)

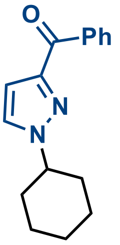

Prepared following **General Procedure A**, using phenyl(1*H*-pyrazol-3-yl)methanone (36.3 mg, 200 µmol, 1.00 equiv., 95% purity), and cyclohexylboronic acid pinacol ester **3** (84 mg, 0.40 mmol, 2.0 equiv.) in MeCN (2.0 mL, *c* = 0.10 M). Purification by CombiFlash® R_f purification system, eluting with EtOAc/heptane (0 – 20%, v/v), gave **24** (45.3 mg, 89%) as a colorless oil.

NMR Spectroscopy (*see spectra*):

^{1}**H NMR** (400 MHz, $CDCl_3$): δ_H 8.27 – 8.22 (m, 2H), 7.56 (s, 1H), 7.51 – 7.43 (m, 3H), 6.93 (d, *J* = 2.4 Hz, 1H), 4.23 (ddd, *J* = 11.7, 7.9, 3.8 Hz, 1H), 2.22 (ddt, *J* = 13.2, 3.9, 1.9 Hz, 2H), 1.92 (dt, *J* = 13.3, 3.5 Hz, 2H), 1.75 (d, *J* = 4.0 Hz, 3H), 1.52 – 1.37 (m, 2H), 1.36 – 1.23 (m, 1H).

^{13}C NMR (101 MHz, $CDCl_3$): δ_C 188.1, 150.1, 137.8, 132.5, 130.7, 128.2, 127.7, 109.1, 62.2, 33.6, 25.4, 25.4 ppm.

HRMS (ESI) m/z calc'd for $C_{16}H_{19}N_2O$ $[M+H]^+$, 255.1497; found, 255.1492.

2-Cyclohexyl-4-phenyl-2*H*-1,2,3-triazole (25)

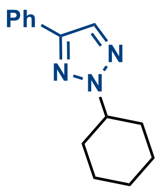

Prepared following **General Procedure A**, using 4-phenyl-2*H*-1,2,3-triazole (30.2 mg, 200 µmol, 1.00 equiv., 96% purity), and cyclohexylboronic acid pinacol ester **3** (84 mg, 0.40 mmol, 2.0 equiv.) in MeCN (2.0 mL, *c* = 0.10 M). Purification by CombiFlash® R_f purification system, eluting with EtOAc/heptane (0 – 50%, v/v), gave **25** (36.3 mg, 80%) as a colorless oil.

NMR Spectroscopy (*see spectra*):

^{1}H NMR (400 MHz, $CDCl_3$): δ_H 7.83 – 7.77 (m, 3H), 7.42 (dd, *J* = 8.4, 6.8 Hz, 2H), 7.38 – 7.29 (m, 1H), 4.49 (tt, *J* = 11.3, 3.9 Hz, 1H), 2.31 – 2.15 (m, 2H), 2.07 – 1.82 (m, 4H), 1.82 – 1.69 (m, 1H), 1.45 (dddd, *J* = 15.8, 12.5, 7.3, 3.4 Hz, 2H), 1.36 – 1.26 (m, 1H) ppm.

^{13}C NMR (101 MHz, $CDCl_3$): δ_C 147.1, 130.9, 130.4, 128.9, 128.3, 126.0, 64.4, 32.9, 25.4, 25.3 ppm.

All recorded spectroscopic data matched those previously reported in the literature.[1]

1-Cyclohexyl-1*H*-benzo[*d*][1,2,3]triazole (26)

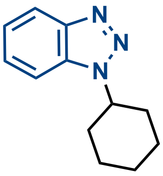

Prepared following **General Procedure A**, using 1*H*-benzotriazole (24.8 mg, 200 µmol, 1.00 equiv., 96% purity), and cyclohexylboronic acid pinacol ester **3** (84 mg, 0.40 mmol, 2.0 equiv.) in MeCN (2.0 mL, *c* = 0.10 M). Purification by CombiFlash® R_f purification system, eluting with EtOAc/heptane (0 – 30%, v/v), gave **26** (28.3 mg, 70%) as a colorless oil.

NMR Spectroscopy (*see spectra*):

^{1}H NMR (400 MHz, $CDCl_3$): δ_H 8.05 (d, *J* = 8.4 Hz, 1H), 7.57 (d, *J* = 8.3 Hz, 1H), 7.51 – 7.40 (m, 1H), 7.34 (dd, *J* = 8.2, 7.0 Hz, 1H), 4.66 (tt, *J* = 10.4, 5.3 Hz, 1H), 2.27 – 2.10 (m, 4H), 2.00 (dt, *J* = 12.6, 3.6 Hz, 2H), 1.82 (dp, *J* = 13.7, 3.2 Hz, 1H), 1.63 – 1.45 (m, 2H), 1.40 (tt, *J* = 12.7, 3.3 Hz, 1H) ppm.

^{13}C NMR (101 MHz, $CDCl_3$): δ_C 146.2, 132.3, 126.9, 123.8, 120.2, 109.9, 59.2, 32.7, 25.7, 25.4 ppm.

All recorded spectroscopic data matched those previously reported in the literature.[1]

1-Cyclohexyl-3-phenyl-1*H*-1,2,4-triazole (27)

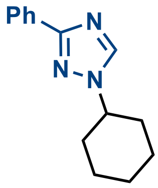

Prepared following **General Procedure A**, using 3-phenyl-4*H*-(1,2,4)triazole (29.9 mg, 200 μmol, 1.00 equiv., 97% purity), and cyclohexylboronic acid pinacol ester **3** (84 mg, 0.40 mmol, 2.0 equiv.) in MeCN (2.0 mL, *c* = 0.10 M). Purification by CombiFlash® R_f purification system, eluting with EtOAc/heptane (0 – 30%, v/v), gave **27** (32.8 mg, 72%) as a colorless oil.

NMR Spectroscopy (*see spectra*):

^{1}H NMR (400 MHz, $CDCl_3$): δ_H 8.11 – 8.08 (m, 3H), 7.45 – 7.35 (m, 3H), 4.19 (tt, *J* = 11.6, 3.8 Hz, 1H), 2.30 – 2.17 (m, 2H), 1.93 (dp, *J* = 10.3, 3.5 Hz, 2H), 1.88 – 1.70 (m, 3H), 1.45 (qt, *J* = 12.8, 3.4 Hz, 2H), 1.37 – 1.21 (m, 1H) ppm.

^{13}C NMR (101 MHz, $CDCl_3$): δ_C 162.1, 141.5, 131.4, 129.1, 128.7, 126.4, 59.7, 33.3, 25.3, 25.2 ppm.

All recorded spectroscopic data matched those previously reported in the literature.[1]

2-Chloro-1-cyclohexyl-1*H*-benzo[d]imidazole (28)

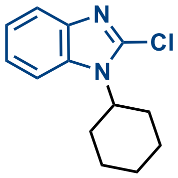

Prepared following **General Procedure A**, using 2-chloro-1*H*-benzo[*d*]imidazole (31.8 mg, 200 μmol, 1.00 equiv., 96% purity), and cyclohexylboronic acid pinacol ester **3** (84 mg, 0.40 mmol, 2.0 equiv.) in MeCN (2.0 mL, *c* = 0.10 M). Purification by CombiFlash® R_f purification system, eluting with EtOAc/heptane (0 – 30%, v/v), gave **28** (24.7 mg, 53%) as a colorless oil.

NMR Spectroscopy (*see spectra*):

^{1}H NMR (400 MHz, $CDCl_3$): δ_H 7.69 (dt, *J* = 7.3, 3.7 Hz, 1H), 7.55 (brs, 1H), 7.25 (dt, *J* = 6.1, 3.7 Hz, 2H), 4.46 (tt, *J* = 12.5, 3.8 Hz, 1H), 2.30 – 2.15 (m, 2H), 1.98 (brs, 4H), 1.83 (d, *J* = 13.2 Hz, 1H), 1.50 (qt, *J* = 13.9, 3.8 Hz, 2H), 1.33 (qt, *J* = 13.2, 3.4 Hz, 1H) ppm.

^{13}C NMR (101 MHz, $CDCl_3$): δ_C 142.1, 140.2, 134.0, 122.8, 122.5, 119.7, 111.5, 57.5, 31.2, 26.1, 25.4 ppm.

All recorded spectroscopic data matched those previously reported in the literature.[1]

1-Cyclohexyl-4-phenyl-1*H*-imidazole (29)

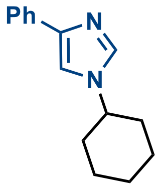

Prepared following **General Procedure A**, using 4-phenyl-1*H*-imidazole (30.0 mg, 200 µmol, 1.00 equiv., 96% purity), and cyclohexylboronic acid pinacol ester **3** (84 mg, 0.40 mmol, 2.0 equiv.) in MeCN (2.0 mL, *c* = 0.10 M). Purification by CombiFlash® R_f purification system, eluting with EtOAc/heptane (0 – 30%, v/v), gave **29** (36.2 mg, 80%) as a colorless oil.

NMR Spectroscopy (*see spectra*):

^{1}H NMR (400 MHz, $CDCl_3$): δ_H 7.77 – 7.71 (m, 2H), 7.57 (s, 1H), 7.35 (t, *J* = 7.6 Hz, 2H), 7.29 – 7.16 (m, 2H), 3.91 (tt, *J* = 11.8, 3.9 Hz, 1H), 2.22 – 2.08 (m, 2H), 1.90 (dt, *J* = 13.4, 3.5 Hz, 2H), 1.69 (dtd, *J* = 37.1, 12.7, 3.8 Hz, 3H), 1.40 (tdd, *J* = 15.0, 12.2, 5.9 Hz, 2H), 1.26 (tt, *J* = 12.7, 3.4 Hz, 1H) ppm.

^{13}C NMR (101 MHz, $CDCl_3$): δ_C 135.6, 134.4, 128.7, 126.7, 124.8, 113.1, 57.2, 34.5, 25.5, 25.3 ppm.

All recorded spectroscopic data matched those previously reported in the literature.[1]

Methyl 1-cyclohexyl-1*H*-pyrrole-3-carboxylate (30)

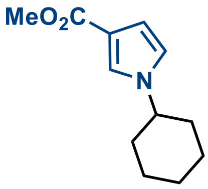

Prepared following **General Procedure A**, using 1H-pyrrole-3-carboxylic acid methyl ester (25.8 mg, 200 µmol, 1.00 equiv., 97% purity), and cyclohexylboronic acid pinacol ester **3** (84 mg, 0.40 mmol, 2.0 equiv.) in MeCN (2.0 mL, *c* = 0.10 M). Purification by CombiFlash® R_f purification system, eluting with EtOAc/heptane (0 – 30%, v/v), gave **30** (40.10 Mg, 97%) as a colorless oil.

NMR Spectroscopy (*see spectra*):

^{1}H NMR (400 MHz, $CDCl_3$): δ_H 7.36 (t, *J* = 2.0 Hz, 1H), 6.65 (t, *J* = 2.6 Hz, 1H), 6.55 (dd, *J* = 2.9, 1.7 Hz, 1H), 3.82 – 3.73 (m, 1H), 3.78 (s, 3H), 2.08 (dtd, *J* = 15.0, 3.9, 2.2 Hz, 2H), 1.88 (dt, *J* = 13.8, 3.5 Hz, 2H), 1.78 – 1.68 (m, 1H), 1.60 (qd, *J* = 12.4, 3.4 Hz, 2H), 1.45 – 1.30 (m, 2H), 1.23 (tt, J = 12.7, 3.6 Hz, 1H) ppm.

^{13}C NMR (101 MHz, $CDCl_3$): δ_C 165.6, 124.0, 119.9, 115.2, 109.6, 59.4, 51.0, 34.5, 25.6, 25.4 ppm.

All recorded spectroscopic data matched those previously reported in the literature.[1]

3,6-Dichloro-9-cyclohexyl-9*H*-carbazole (31)

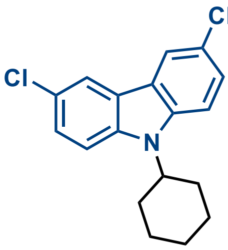

Prepared following **General Procedure A**, using 3,6-dichloro-9*H*-carbazole (48.7 mg, 200 µmol, 1.00 equiv., 97% purity), and cyclohexylboronic acid pinacol ester **3** (84 mg, 0.40 mmol, 2.0 equiv.) in MeCN (2.0 mL, *c* = 0.10 M). Purification by CombiFlash® R_f purification system, eluting with EtOAc/heptane (0 – 20%, v/v), gave **31** (61.1 mg, 96%) as a colorless solid.

NMR Spectroscopy (<u>see spectra</u>):

^{1}**H NMR** (400 MHz, $CDCl_3$): δ_H 7.99 (d, *J* = 2.1 Hz, 2H), 7.48 – 7.38 (m, 4H), 4.41 (tt, *J* = 12.5, 3.9 Hz, 1H), 2.30 (qd, *J* = 12.6, 3.5 Hz, 2H), 2.10 – 1.77 (m, 5H), 1.53 (qt, *J* = 12.8, 3.4 Hz, 2H), 1.38 (qt, *J* = 12.8, 3.2 Hz, 1H) ppm.

^{13}**C NMR** (101 MHz, $CDCl_3$): δ_C 138.6, 126.1, 124.5, 123.6, 120.2, 111.6, 55.9, 30.9, 26.5, 25.7 ppm.

HRMS (ESI) m/z calc'd for $C_{18}H_{17}NCl_2$ $[M+H]^+$, 317.0738; found, 317.0739.

9-Cyclohexyl-9*H*-pyrido[3,4-b]indole (32)

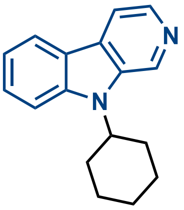

Prepared following **General Procedure A**, using norharmane (34.3 mg, 200 µmol, 1.00 equiv., 98% purity), and cyclohexylboronic acid pinacol ester **3** (84 mg, 0.40 mmol, 2.0 equiv.) in MeCN (2.0 mL, *c* = 0.10 M). Purification by CombiFlash® R_f purification system, eluting with EtOAc/heptane (0 – 60%, v/v), gave **32** (39.4 mg, 79%) as a brown solid.

NMR Spectroscopy (<u>see spectra</u>):

^{1}**H NMR** (400 MHz, $CDCl_3$): δ_H 9.07 (s, 1H), 8.42 (d, *J* = 5.3 Hz, 1H), 8.14 (d, *J* = 7.9 Hz, 1H), 7.98 (d, *J* = 5.3 Hz, 1H), 7.57 (d, *J* = 6.5 Hz, 2H), 7.26 (t, *J* = 7.4 Hz, 1H), 4.53 (tt, *J* = 12.5, 3.8 Hz, 1H), 2.35 (qd, *J* = 12.9, 3.9 Hz, 2H), 2.12 – 1.93 (m, 4H), 1.85 (d, *J* = 13.0 Hz, 1H), 1.62 – 1.31 (m, 3H) ppm.

^{13}**C NMR** (101 MHz, $CDCl_3$): δ_C 140.9, 137.6, 135.9, 132.9, 129.3, 129.0, 128.4, 122.0, 121.3, 119.6, 114.7, 110.9, 55.9, 31.4, 31.2, 26.4, 25.6, 25.0 ppm.

HRMS (ESI) m/z calc'd for $C_{17}H_{19}N_2$ $[M+H]^+$, 251.1548; found, 251.1544.

2-Chloro-*N*-cyclohexylnicotinamide (33)

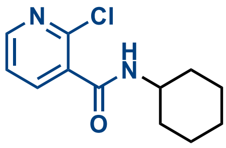

Prepared following **General Procedure A**, using 2-chloronicotinamide (41.4 mg, 200 µmol, 1.00 equiv., 97% purity), and cyclohexylboronic acid pinacol ester **3** (84 mg, 0.40 mmol, 2.0 equiv.) in MeCN (2.0 mL, *c* = 0.10 M). Purification by CombiFlash® R_f purification system, eluting with EtOAc/heptane (0 – 80%, v/v), gave **33** (33.2 mg, 70%) as a colorless solid.

NMR Spectroscopy (*see spectra*):

^{1}**H NMR** (400 MHz, $CDCl_3$): δ_H 8.44 (dd, *J* = 4.8, 2.1 Hz, 1H), 8.07 (dd, *J* = 7.6, 2.0 Hz, 1H), 7.33 (dd, *J* = 7.7, 4.8 Hz, 1H), 6.32 (brs, 1H, NH), 4.07 – 3.95 (m, 1H), 2.08 – 1.99 (m, 2H), 1.75 (dt, *J* = 13.5, 4.0 Hz, 2H), 1.68 – 1.63 (m, 1H), 1.43 (ddt, *J* = 11.3, 9.8, 5.7 Hz, 2H), 1.32 – 1.27 (m, 3H) ppm.

^{13}**C NMR** (101 MHz, $CDCl_3$): δ_C 163.8, 150.9, 147.2, 139.9, 131.8, 122.9, 49.3, 32.9, 25.6, 24.8 ppm.

All recorded spectroscopic data matched those previously reported in the literature.[1]

5-Bromo-*N*-cyclohexylnicotinamide (34)

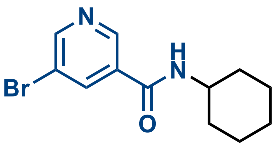

Prepared following **General Procedure A**, using 5-bromonicotinamide (31.3 mg, 200 µmol, 1.00 equiv., 99% purity), and cyclohexylboronic acid pinacol ester **3** (84 mg, 0.40 mmol, 2.0 equiv.) in MeCN (2.0 mL, *c* = 0.10 M). Purification by CombiFlash® R_f purification system, eluting with EtOAc/heptane (0 – 80%, v/v), gave **34** (40.9 mg, 72%) as a colorless solid.

NMR Spectroscopy (*see spectra*):

^{1}**H NMR** (400 MHz, $CDCl_3$): δ_H 8.84 (d, *J* = 2.0 Hz, 1H), 8.75 (d, *J* = 2.3 Hz, 1H), 8.23 (t, *J* = 2.1 Hz, 1H), 6.12 (brs, 1H), 4.02 – 3.91 (m, 1H), 2.19 – 1.98 (m, 2H), 1.76 (dp, *J* = 11.5, 3.8 Hz, 2H), 1.66 (dt, *J* = 13.1, 3.8 Hz, 1H), 1.50 – 1.33 (m, 2H), 1.32 – 1.13 (m, 3H) ppm.

^{13}**C NMR** (101 MHz, $CDCl_3$): δ_C 163.4, 153.1, 145.8, 138.0, 132.2, 121.1, 49.4, 33.2, 25.6, 25.0 ppm.

All recorded spectroscopic data matched those previously reported in the literature.[3]

***N*-Cyclohexyl-2-(trifluoromethyl)benzamide (35)**

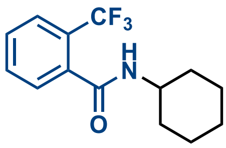

Prepared following **General Procedure A**, using 2-(trifluoromethyl)benzamide (37.8 mg, 200 µmol, 1.00 equiv.), and cyclohexylboronic acid pinacol ester **3** (84 mg, 0.40 mmol, 2.0 equiv.) in MeCN (2.0 mL, *c* = 0.10 M). Purification by CombiFlash® R_f purification system, eluting with EtOAc/heptane (0 – 30%, v/v), gave **35** (40.0 mg, 74%) as a colorless solid.

NMR Spectroscopy (*see spectra*):

^{1}**H NMR** (400 MHz, $CDCl_3$): δ_H 7.99 (d, *J* = 1.9 Hz, 1H), 7.93 (d, *J* = 7.8 Hz, 1H), 7.71 (d, *J* = 7.7 Hz, 1H), 7.53 (t, *J* = 7.8 Hz, 1H), 6.36 – 6.08 (brs, 1H, NH), 4.05 – 3.86 (m, 1H), 2.02 (dq, *J* = 12.2, 3.8 Hz, 2H), 1.84 – 1.70 (m, 2H), 1.65 (dt, *J* = 13.0, 3.7 Hz, 1H), 1.49 – 1.31 (m, 2H), 1.31 – 1.09 (m, 3H) ppm.

^{13}**C NMR** (101 MHz, $CDCl_3$): δ_C 165.4, 136.0, 131.2 (q, *J* = 33.3 Hz), 130.3, 129.2, 127.9 (d, *J* = 3.7 Hz), 124.0 (d, *J* = 3.9 Hz), 123.9 (d, *J* = 273.7 Hz), 49.2, 33.3, 25.6, 25.1 ppm.

^{19}**F NMR** (376 MHz, $CDCl_3$): δ_F –62.71 ppm.

All recorded spectroscopic data matched those previously reported in the literature.[1]

N-Cyclohexyl-3-methoxybenzamide (36)

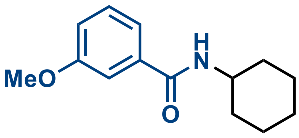

Prepared following **General Procedure A**, using 3-methoxybenzamide (31.2 mg, 200 µmol, 1.00 equiv., 97% purity), and cyclohexylboronic acid pinacol ester **3** (84 mg, 0.40 mmol, 2.0 equiv.) in MeCN (2.0 mL, *c* = 0.10 M). Purification by CombiFlash® R_f purification system, eluting with EtOAc/heptane (0 – 30%, v/v), gave **36** (36.5 mg, 78%) as a colorless solid.

NMR Spectroscopy (*see spectra*):

^{1}**H NMR** (400 MHz, $CDCl_3$): δ_H 7.37 – 7.28 (m, 3H), 7.09 – 6.98 (m, 1H), 6.02 (brs, 1H, NH), 3.99 (tdq, *J* = 11.1, 7.8, 3.4 Hz, 1H), 3.86 (d, *J* = 1.2 Hz, 3H), 2.12 – 1.96 (m, 2H), 1.77 (dp, *J* = 11.9, 4.0 Hz, 2H), 1.67 (dt, *J* = 12.9, 3.9 Hz, 1H), 1.45 (dt, *J* = 16.2, 10.5 Hz, 2H), 1.34 – 1.14 (m, 3H) ppm.

^{13}**C NMR** (101 MHz, $CDCl_3$): δ_C 166.6, 159.9, 136.8, 129.6, 118.7, 117.5, 112.5, 55.6, 48.8, 33.3, 25.7, 25.0 ppm.

All recorded spectroscopic data matched those previously reported in the literature.[4]

4-(Isopropylsulfonyl)-*N*-cyclohexylaniline (37)

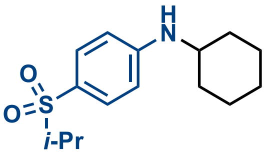

Prepared following **General Procedure A**, using 4-(isopropylsulfonyl)aniline (40.7 mg, 200 µmol, 1.00 equiv., 98% purity), and cyclohexylboronic acid pinacol ester **3** (84 mg, 0.40 mmol, 2.0 equiv.) in MeCN (2.0 mL, *c* =

0.10 M). Purification by CombiFlash® R_f purification system, eluting with EtOAc/heptane (0 – 100%, v/v) with 1% Et_3N, gave **37** (40.8 mg, 73%) as a colorless solid.

NMR Spectroscopy (see spectra):

^{1}**H NMR** (400 MHz, $CDCl_3$): δ_H 7.60 – 7.57 (m, 2H), 6.59 – 6.56 (m, 2H), 4.18 (brs, 1H), 3.31 (brs, 1H), 3.10 (p, *J* = 6.9 Hz, 1H), 2.10 – 1.98 (m, 2H), 1.78 (dt, J = 13.4, 3.9 Hz, 2H), 1.72 – 1.59 (m, 1H), 1.47 – 1.32 (m, 2H), 1.32 – 1.12 (m, 3H), 1.26 (d, *J* = 8.0 Hz, 6H) ppm.

^{13}**C NMR** (101 MHz, $CDCl_3$): δ_C 151.5, 131.2, 122.6, 111.9, 55.9, 51.4, 33.1, 25.8, 24.9, 16.1 ppm.

HRMS (ESI) m/z calc'd for $C_{15}H_{24}NO_2S$ $[M+H]^+$, 282.1528; found, 282.1525.

2-(Benzyloxy)-*N*-cyclohexylaniline (38)

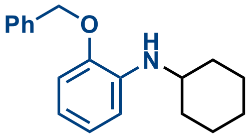

Prepared following **General Procedure A**, using 2-(benzyloxy)aniline (41.1 mg, 200 μmol, 1.00 equiv., 97% purity), and cyclohexylboronic acid pinacol ester **3** (84 mg, 0.40 mmol, 2.0 equiv.) in MeCN (2.0 mL, *c* = 0.10 M). Purification by CombiFlash® R_f purification system, eluting with EtOAc/heptane (0 – 100%, v/v) with 1% Et_3N, gave **38** (34.3 mg, 61%) as a yellow solid.

NMR Spectroscopy (see spectra):

^{1}**H NMR** (400 MHz, $CDCl_3$): δ_H 7.46 – 7.29 (m, 5H), 6.90 – 6.78 (m, 2H), 6.72 – 6.54 (m, 2H), 5.08 (s, 2H), 4.26 (brs, 1H), 3.27 (tt, *J* = 10.1, 3.9 Hz, 1H), 2.06 (dq, *J* = 12.2, 3.8 Hz, 2H), 1.76 (dp, *J* = 11.9, 4.0 Hz, 2H), 1.65 (dt, *J* = 12.8, 3.9 Hz, 1H), 1.46 – 1.30 (m, 2H), 1.30 – 1.14 (m, 3H) ppm.

^{13}**C NMR** (101 MHz, $CDCl_3$): δ_C 146.1, 137.5, 129.7, 128.7, 128.0, 127.6, 121.9, 116.0, 111.8, 110.9, 70.7, 51.7, 33.5, 26.1, 25.2 ppm.

HRMS (ESI) m/z calc'd for $C_{19}H_{24}NO$ $[M+H]^+$, 282.1858; found, 282.1856.

***N*-Cyclohexyl-*N*-phenylpyridin-2-amine (39)**

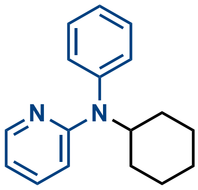

Prepared following **General Procedure A**, using N-phenylpyridin-2-amine (35.1 mg, 200 μmol, 1.00 equiv., 97% purity), and cyclohexylboronic acid pinacol ester **3** (84 mg, 0.40 mmol, 2.0 equiv.) in MeCN (2.0 mL, *c* = 0.10 M). Purification by CombiFlash® R_f purification system, eluting with EtOAc/heptane (0 – 30%, v/v), gave **39** (35.1 mg, 70%) as a brown oil.

NMR Spectroscopy (see spectra):

^{1}H NMR (400 MHz, $CDCl_3$): δ_H 8.17 (dd, *J* = 5.0, 2.0 Hz, 1H), 7.44 (t, *J* = 7.5 Hz, 2H), 7.40 – 7.30 (m, 1H), 7.22 – 7.10 (m, 3H), 6.49 (dd, *J* = 7.0, 5.0 Hz, 1H), 5.89 (d, *J* = 8.7 Hz, 1H), 4.85 (tt, *J* = 12.0, 3.6 Hz, 1H), 1.99 – 1.92 (m, 2H), 1.80 – 1.70 (m, 2H), 1.64 – 1.60 (m, 1H), 1.53 – 1.40 (m, 2H), 1.30 – 1.21 (m, 1H), 1.19 – 1.06 (m, 2H) ppm.

^{13}C NMR (101 MHz, $CDCl_3$): δ_C 158.9, 147.4, 141.6, 136.7, 131.9, 129.8, 127.4, 112.0, 109.1, 54.1, 32.1, 26.2, 25.8 ppm.

HRMS (ESI) m/z calc'd for $C_{17}H_{21}N_2$ $[M+H]^+$, 253.1705; found, 253.1700.

_N_-Cyclohexyl-4-iodobenzenesulfonamide (40)

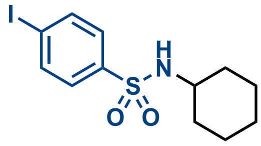

Prepared following **General Procedure A**, using 4-iodobenzenesulfonamide (56.6 mg, 200 μmol, 1.00 equiv., >99% purity), and cyclohexylboronic acid pinacol ester **3** (84 mg, 0.40 mmol, 2.0 equiv.) in MeCN (4.0 mL, *c* = 0.050 M). Purification by CombiFlash® R_f purification system, eluting with EtOAc/heptane (0 – 30%, v/v), gave **40** (50.4 mg, 69%) as a colorless solid.

NMR Spectroscopy (*see spectra*):

^{1}H NMR (400 MHz, $CDCl_3$): δ_H 7.85 (d, *J* = 8.2 Hz, 2H), 7.60 (d, *J* = 8.4 Hz, 2H), 4.85 (d, *J* = 7.6 Hz, 1H, NH), 3.23 – 3.02 (m, 1H), 1.74 (dd, *J* = 11.1, 5.8 Hz, 2H), 1.63 (dq, *J* = 13.1, 4.0 Hz, 2H), 1.51 (dt, *J* = 13.1, 3.9 Hz, 1H), 1.30 – 1.08 (m, 5H) ppm.

^{13}C NMR (101 MHz, $CDCl_3$): δ_C 141.4, 138.4, 128.5, 99.8, 52.9, 33.9, 25.2, 24.7 ppm.

HRMS (ESI) m/z calc'd for $C_{12}H_{17}NO_2SI$ $[M+H]^+$, 366.0025; found, 366.0029.

(Cyclohexylimino)diphenyl-l6-sulfanone (41)

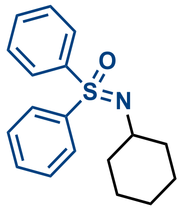

Prepared following **General Procedure A**, using (phenylsulfonimidoyl)benzene (44.8 mg, 200 μmol, 1.00 equiv., 97% purity), and cyclohexylboronic acid pinacol ester **3** (84 mg, 0.40 mmol, 2.0 equiv.) in MeCN (2.0 mL, *c* = 0.10 M). Purification by CombiFlash® R_f purification system, eluting with EtOAc/heptane (0 – 10%, v/v), gave **41** (58.6 mg, 98%) as a colorless solid.

NMR Spectroscopy (*see spectra*):

^{1}H NMR (400 MHz, $CDCl_3$): δ_H 7.98 – 7.96 (m, 4H), 7.47 – 7.42 (m, 6H), 3.10 – 2.96 (m, 1H), 1.96 – 1.85 (m, 2H), 1.75 – 1.67 (m, 2H), 1.56 – 1.44 (m, 3H), 1.24 – 1.13 (m, 2H) ppm.

^{13}C NMR (101 MHz, $CDCl_3$): δ_C 141.7, 132.2, 129.1, 128.6, 54.2, 37.2, 25.8, 25.4 ppm.

All recorded spectroscopic data matched those previously reported in the literature.[5]

_N_-Cyclohexyl-1,1-diphenylmethanimine (42)

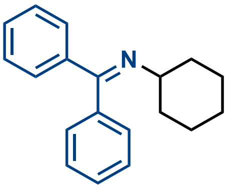

Prepared following **General Procedure A**, using benzophenone imine (36.6 mg, 200 μmol, 1.00 equiv., 99% purity), and cyclohexylboronic acid pinacol ester **3** (84 mg, 0.40 mmol, 2.0 equiv.) in MeCN (2.0 mL, *c* = 0.10 M). Purification by CombiFlash® R_f purification system, eluting with EtOAc/heptane (0 – 80%, v/v), gave **42** (48.8 mg, 93%) as a colorless solid.

NMR Spectroscopy (<u>*see spectra*</u>):

^{1}H NMR (400 MHz, $CDCl_3$): δ_H 7.63 – 7.54 (m, 2H), 7.48 – 7.28 (m, 6H), 7.16 (dd, J = 7.3, 2.0 Hz, 2H), 3.22 (h, *J* = 6.1 Hz, 1H), 1.74 (dt, *J* = 12.8, 3.6 Hz, 3H), 1.68 – 1.56 (m, 5H), 1.33 – 1.15 (m, 2H) ppm.

^{13}C NMR (101 MHz, $CDCl_3$): δ_C 159.0, 138.3, 135.7, 129.7, 129.5, 128.8, 128.6, 128.5, 128.3, 128.2, 128.0, 127.9, 61.6, 34.0, 25.8, 24.6 ppm.

All recorded spectroscopic data matched those previously reported in the literature.[1]

5-Bromo-3-cyclohexylbenzo[*d*]oxazol-2(3*H*)-one (43)

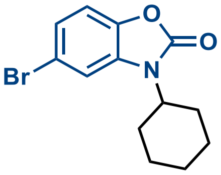

Prepared following **General Procedure A**, using 5-bromo-2,3-dihydro-1,3-benzoxazol-2-one (44.1 mg, 200 μmol, 1.00 equiv., 97% purity), and cyclohexylboronic acid pinacol ester **3** (84 mg, 0.40 mmol, 2.0 equiv.) in MeCN (2.0 mL, *c* = 0.10 M). Purification by CombiFlash® R_f purification system, eluting with EtOAc/heptane (0 – 20%, v/v), gave **43** (50.0 mg, 84%) as a colorless solid.

NMR Spectroscopy (<u>*see spectra*</u>):

^{1}H NMR (400 MHz, $CDCl_3$): δ_H 7.26 – 7.18 (m, 2H), 7.06 (d, *J* = 8.4 Hz, 1H), 4.07 (tt, *J* = 12.3, 4.0 Hz, 1H), 2.10 – 1.84 (m, 6H), 1.80 – 1.71 (m, 1H), 1.51– 1.21 (m, 3H) ppm.

^{13}C NMR (101 MHz, $CDCl_3$): δ_C 153.7, 141.8, 131.9, 124.9, 116.2, 112.8, 111.4, 54.8, 29.9, 25.8, 25.2 ppm.

HRMS (ESI) m/z calc'd for $C_{13}H_{15}NO_2Br$ $[M+H]^+$, 296.0286; found, 296.0282.

1'-Cyclohexylspiro[cyclopentane-1,3'-indolin]-2'-one (44)

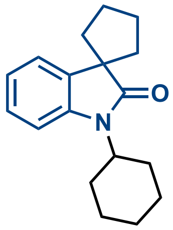

Prepared following **General Procedure A**, using spiro[cyclopentane-1,3'-indolin]-2'-one (37.5 mg, 200 µmol, 1.00 equiv., 99% purity), and cyclohexylboronic acid pinacol ester **3** (84 mg, 0.40 mmol, 2.0 equiv.) in MeCN (2.0 mL, *c* = 0.10 M). Purification by CombiFlash® R_f purification system, eluting with EtOAc/heptane (0 – 20%, v/v), gave **44** (46.8 mg, 87%) as a colorless solid.

NMR Spectroscopy (*see spectra*):

^{1}**H NMR** (400 MHz, $CDCl_3$): δ_H 7.09 (t, *J* = 6.8 Hz, 2H), 6.94 – 6.88 (m, 2H), 4.09 (tt, *J* = 12.6, 4.0 Hz, 1H), 2.10 – 1.71 (m, 16H), 1.44 (qt, *J* = 13.0, 3.6 Hz, 2H), 1.29 (tt, *J* = 13.2, 3.5 Hz, 1H) ppm.

^{13}**C NMR** (101 MHz, $CDCl_3$): δ_C 181.9, 142.0, 137.5, 127.0, 122.5, 121.9, 109.8, 53.6, 52.0, 38.6, 29.2, 26.8, 26.1, 25.5 ppm.

HRMS (ESI) m/z calc'd for $C_{18}H_{24}NO$ $[M+H]^+$, 270.1858; found, 270.1856.

1-Cyclohexyl-3,4-dihydroquinolin-2(1*H*)-one (45)

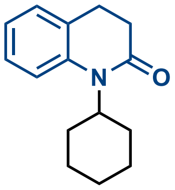

Prepared following **General Procedure A**, using hydrocarbostyril (30.0 mg, 200 µmol, 1.00 equiv., 98% purity), and cyclohexylboronic acid pinacol ester **3** (84 mg, 0.40 mmol, 2.0 equiv.) in MeCN (2.0 mL, *c* = 0.10 M). Purification by CombiFlash® R_f purification system, eluting with EtOAc/heptane (0 – 20%, v/v), gave **45** (42.2 mg, 92%) as a colorless solid.

NMR Spectroscopy (*see spectra*):

^{1}**H NMR** (400 MHz, $CDCl_3$): δ_H 7.22 (td, *J* = 7.8, 1.6 Hz, 1H), 7.14 (d, *J* = 7.8 Hz, 2H), 6.99 (td, *J* = 7.3, 1.1 Hz, 1H), 4.09 (tt, *J* = 12.3, 3.8 Hz, 1H), 2.80 (dd, *J* = 8.5, 5.7 Hz, 2H), 2.61 – 2.49 (m, 2H), 2.40 (tt, *J* = 12.5, 6.0 Hz, 2H), 1.90 –1.58 (m, 6H), 1.41 – 1.18 (m, 3H) ppm.

^{13}**C NMR** (101 MHz, $CDCl_3$): δ_C 171.6, 141.2, 128.8, 127.7, 127.1, 123.0, 116.7, 58.4, 33.9, 29.9, 26.7, 26.0, 25.7 ppm.

All recorded spectroscopic data matched those previously reported in the literature.[6]

(4-Cyclohexylpiperazin-1-yl)(phenyl)methanone (46)

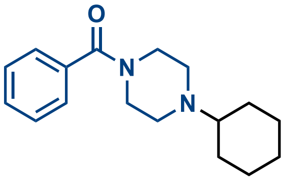

Prepared following **General Procedure A**, using phenyl(piperazin-1-yl)methanone (38.4 mg, 200 μmol, 1.00 equiv., 98% purity), and cyclohexylboronic acid pinacol ester **3** (84 mg, 0.40 mmol, 2.0 equiv.) in MeCN (2.0 mL, *c* = 0.10 M). Purification by CombiFlash® R_f purification system, eluting with MeOH/DCM (0 – 10%, v/v), gave **46** (70.3 mg, 70%) as a colorless oil.

NMR Spectroscopy (see spectra):

^{1}H NMR (400 MHz, $CDCl_3$): δ_H 7.41 – 7.37 (m, 5H), 3.79 (brs, 2H), 3.42 (brs, 2H), 2.65 (brs, 2H), 2.53 – 2.48 (m, 2H), 2.31 (brs, 1H), 1.82 (s, 3H), 1.87 – 1.57 (m, 5H), 1.26 – 1.16 (m, 4H) ppm.

^{13}C NMR (101 MHz, $CDCl_3$): δ_C 170.3, 136.0, 129.7, 128.6, 127.2, 63.7, 49.5, 48.8 48.2, 42.6, 28.9, 26.3, 25.9 ppm.

All recorded spectroscopic data matched those previously reported in the literature.[7]

9-Cyclohexyl-1,3-dimethyl-3,9-dihydro-1*H*-purine-2,6-dione (47)

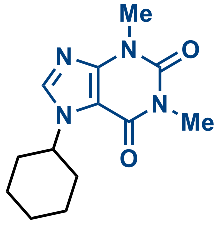

Prepared following **General Procedure A**, using 3,7-dihydro-1,3-dimethyl-1H-purine-2,6-dione (36.4 mg, 200 μmol, 1.00 equiv., 99% purity), and cyclohexylboronic acid pinacol ester **3** (84 mg, 0.40 mmol, 2.0 equiv.) in MeCN (2.0 mL, *c* = 0.10 M). Purification by CombiFlash® R_f purification system, eluting with EtOAc/heptane (0 – 50%, v/v), gave **47** (27.1 mg, 52%) as a colorless solid.

NMR Spectroscopy (see spectra):

^{1}H NMR (400 MHz, $CDCl_3$): δ_H 7.65 (s, 1H), 4.64 (t, J = 12.2 Hz, 1H), 3.59 (s, 3H), 3.41 (s, 3H), 2.20 (d, J = 11.8 Hz, 2H), 1.92 (d, J = 13.4 Hz, 2H), 1.78 – 1.66 (m, 2H), 1.49 (q, J = 13.3 Hz, 2H), 1.34 – 1.21 (m, 1H) ppm.

^{13}C NMR (101 MHz, $CDCl_3$): δ_C 155.2, 151.7, 148.8, 138.2, 106.9, 57.4, 33.9, 30.0, 28.2, 25.6, 25.3 ppm.

All recorded spectroscopic data matched those previously reported in the literature.[1]

_N_-Cyclohexylpyrrolo[2,1-_f_][1,2,4]triazin-4-amine (48)

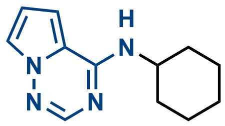

Prepared following **General Procedure A**, using 4-amino-pyrrolo[2,1-*f*][1,2,4]triazine (27.4 mg, 200 µmol, 1.00 equiv., 98% purity), and cyclohexylboronic acid pinacol ester **3** (84 mg, 0.40 mmol, 2.0 equiv.) in MeCN (2.0 mL, *c* = 0.10 M). Purification by CombiFlash® R_f purification system, eluting with EtOAc/heptane (0 – 90%, v/v), gave **48** (22.8 mg, 53%) as a colorless solid.

NMR Spectroscopy (see spectra):

^{1}H NMR (400 MHz, $CDCl_3$): δ_H 7.92 (s, 1H), 7.52 (s, 1H), 6.61 (s, 1H), 6.53 (m, 1H), 5.18 (brs, 1H), 4.16 (tt, *J* = 11.3, 4.1 Hz, 1H), 2.11 (dq, *J* = 12.3, 3.7 Hz, 2H), 1.92 – 1.61 (m, 4H), 1.56 – 1.37 (m, 2H), 1.34 – 1.19 (m, 4H) ppm.

^{13}C NMR (101 MHz, $CDCl_3$): δ_C 153.5, 147.7, 119.0, 114.8, 110.4, 98.7, 49.4, 33.4, 25.7, 25.0 ppm.

HRMS (ESI) m/z calc'd for $C_{12}H_{17}N_4$ $[M+H]^+$, 217.1453; found, 217.1449.

3-Chloro-1-isobutyl-1*H*-indazole (49)

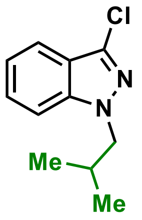

Prepared following **General Procedure B**, using 3-chloro-1*H*-indazole (32.1 mg, 200 µmol, 1.00 equiv., 95% purity), and 2-isobutyl-4,4,5,5-tetramethyl-1,3,2-dioxaborolane (73.6 mg, 400 µmol, 2.00 equiv.) in MeCN (2.0 mL, *c* = 0.10 M). Purification by CombiFlash® R_f purification system, eluting with EtOAc/heptane (0 – 20%, v/v), gave **49** (38.9 mg, 93%) as a colorless oil.

NMR Spectroscopy (see spectra):

^{1}H NMR (400 MHz, $CDCl_3$): δ_H 7.66 (d, *J* = 8.2 Hz, 1H), 7.43 – 7.33 (m, 2H), 7.18 (ddd, *J* = 7.9, 6.5, 1.2 Hz, 1H), 4.11 (d, *J* = 7.3 Hz, 2H), 2.45 – 2.24 (m, 1H), 0.93 (d, *J* = 6.8 Hz, 6H) ppm.

^{13}C NMR (101 MHz, $CDCl_3$): δ_C 141.3, 132.6, 127.4, 121.1, 121.0, 119.9, 109.6, 56.7, 29.7, 20.3 ppm.

HRMS (ESI) m/z calc'd for $C_{11}H_{14}N_2Cl$ $[M+H]^+$, 209.0846; found, 209.0845.

4-(3-Chloro-1*H*-indazol-1-yl)butanenitrile (50)

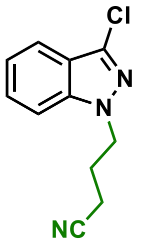

Prepared following **General Procedure B**, using 3-chloro-1*H*-indazole (32.1 mg, 200 µmol, 1.00 equiv., 95% purity), and 4-(4,4,5,5-tetramethyl-1,3,2-dioxaborolan-2-yl)butanenitrile (78.0 mg, 400 µmol, 2.00 equiv.) in MeCN (2.0 mL, *c* = 0.10 M). Purification by CombiFlash® R_f purification system, eluting with EtOAc/heptane (0 – 30%, v/v), gave **50** (41.6 mg, 95%) as a colorless oil.

NMR Spectroscopy (*see spectra*):

^{1}H NMR (400 MHz, $CDCl_3$): δ_H 7.71 (d, *J* = 8.2 Hz, 1H), 7.54 – 7.43 (m, 2H), 7.31 – 7.22 (m, 1H), 4.49 (t, *J* = 5.8 Hz, 2H), 2.43 – 2.29 (m, J = 3.8 Hz, 4H) ppm.

^{13}C NMR (101 MHz, $CDCl_3$): δ_C 141.3, 134.0, 128.2, 121.8, 121.3, 120.1, 118.9, 109.1, 46.9, 25.7, 14.9 ppm.

All recorded spectroscopic data matched those previously reported in the literature.[1]

***tert*-Butyl (2-(3-chloro-1*H*-indazol-1-yl)ethyl)carbamate (51)**

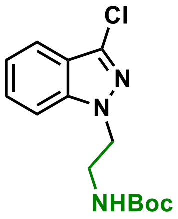

Prepared following **General Procedure B**, using 3-chloro-1*H*-indazole (32.1 mg, 200 µmol, 1.00 equiv., 95% purity), and tert-butyl (2-(4,4,5,5-tetramethyl-1,3,2-dioxaborolan-2-yl)ethyl)carbamate (108.0 mg, 400 µmol, 2.00 equiv.) in MeCN (2.0 mL, *c* = 0.10 M). Purification by CombiFlash® R_f purification system, eluting with EtOAc/heptane (0 – 30%, v/v), gave **51** (54.3 mg, 92%) as a colorless oil.

NMR Spectroscopy (*see spectra*):

^{1}H NMR (400 MHz, $CDCl_3$): δ_H 7.66 (dt, *J* = 8.2, 1.0 Hz, 1H), 7.44 – 7.38 (m, 2H), 7.20 (ddd, *J* = 7.9, 5.1, 2.5 Hz, 1H), 4.76 (brs, 1H, NH), 4.45 (t, *J* = 5.7 Hz, 2H), 3.61 (q, *J* = 5.9 Hz, 2H), 1.40 (s, 9H) ppm.

^{13}C NMR (101 MHz, $CDCl_3$): δ_C 156.0, 141.8, 133.5, 133.3, 129.8, 128.5, 127.8, 121.5, 121.1, 119.8, 109.6, 79.8, 48.4, 40.6, 28.5 ppm.

HRMS (ESI) m/z calc'd for $C_{14}H_{18}N_3O_2NaCl$ $[M+Na]^+$, 318.0985; found, 318.0994.

1-Allyl-3-chloro-1*H*-indazole (52)

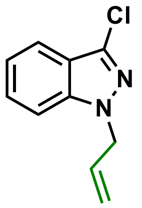

Prepared following **General Procedure B**, using 3-chloro-1*H*-indazole (32.1 mg, 200 µmol, 1.00 equiv., 95% purity), and 2-allyl-4,4,5,5-tetramethyl-1,3,2-dioxaborolane (67.2 mg, 400 µmol, 2.00 equiv.) in MeCN (2.0 mL, *c* = 0.10 M). Purification by CombiFlash® R_f purification system, eluting with EtOAc/heptane (0 – 20%, v/v), gave **52** (31.5 mg, 82%) as a colorless oil.

NMR Spectroscopy (*see spectra*):

^{1}**H NMR** (400 MHz, $CDCl_3$): δ_H 7.66 (dd, *J* = 8.8, 0.9 Hz, 1H), 7.56 (dt, *J* = 8.5, 1.1 Hz, 1H), 7.29 – 7.20 (m, 1H), 7.12 (ddd, *J* = 8.5, 6.6, 0.8 Hz, 1H), 6.06 (ddt, *J* = 17.1, 10.2, 5.7 Hz, 1H), 5.31 (dq, *J* = 10.3, 1.3 Hz, 1H), 5.20 (dq, *J* = 17.1, 1.4 Hz, 1H), 5.07 (dt, *J* = 5.8, 1.6 Hz, 2H) ppm.

^{13}**C NMR** (101 MHz, $CDCl_3$): δ_C 148.1, 131.4, 127.1, 122.4, 119.5, 119.1, 118.8, 118.1, 53.1 ppm.

HRMS (ESI) m/z calc'd for $C_{10}H_{10}N_2Cl$ $[M+H]^+$, 193.0533; found, 193.0537.

Ethyl 3-(3-chloro-1*H*-indazol-1-yl)propanoate (53)

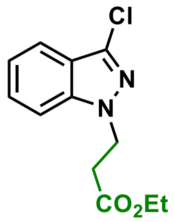

Prepared following **General Procedure B**, using 3-chloro-1*H*-indazole (32.1 mg, 200 µmol, 1.00 equiv., 95% purity), and ethyl 3-(4,4,5,5-tetramethyl-1,3,2-dioxaborolan-2-yl)propanoate (91.2 mg, 400 µmol, 2.00 equiv.) in MeCN (2.0 mL, *c* = 0.10 M). Purification by CombiFlash® R_f purification system, eluting with EtOAc/heptane (0 – 30%, v/v), gave **53** (45.0 mg, 89%) as a colorless oil.

NMR Spectroscopy (*see spectra*):

^{1}**H NMR** (400 MHz, $CDCl_3$): δ_H 7.64 (dt, *J* = 8.2, 1.0 Hz, 1H), 7.49 – 7.41 (m, 2H), 7.19 (ddd, *J* = 8.0, 6.6, 1.2 Hz, 1H), 4.60 (t, *J* = 6.7 Hz, 2H), 4.07 (q, *J* = 7.2 Hz, 2H), 2.96 (t, *J* = 6.8 Hz, 2H), 1.17 (t, *J* = 7.1 Hz, 3H) ppm.

^{13}**C NMR** (101 MHz, $CDCl_3$): δ_C 171.2, 141.1, 133.5, 127.7, 121.4, 121.1, 119.8, 109.7, 61.0, 44.6, 34.5, 14.2 ppm.

HRMS (ESI) m/z calc'd for $C_{12}H_{14}N_2O_2Cl$ $[M+H]^+$, 253.0744; found, 253.0739.

3-Chloro-1-phenethyl-1*H*-indazole (54)

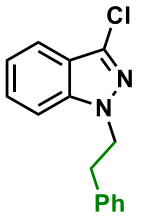

Prepared following **General Procedure B**, using 3-chloro-1*H*-indazole (32.1 mg, 200 µmol, 1.00 equiv., 95% purity), and 4,4,5,5-tetramethyl-2-phenethyl-1,3,2-dioxaborolane (92.9 mg, 400 µmol, 2.00 equiv.) in MeCN (4.0 mL, *c* = 0.050 M). Purification by CombiFlash® R_f purification system, eluting with EtOAc/heptane (0 – 20%, v/v), gave **54** (46.5 mg, 91%) as a colorless oil.

NMR Spectroscopy (*see spectra*):

^{1}**H NMR** (400 MHz, $CDCl_3$): δ_H 7.75 – 7.73 (m, 1H), 7.40 (ddd, *J* = 7.9, 6.8, 1.1 Hz, 1H), 7.33 – 7.19 (m, 7H), 4.65 – 4.58 (m, 2H), 3.29 (dd, *J* = 8.4, 6.7 Hz, 2H) ppm.

^{13}**C NMR** (101 MHz, $CDCl_3$): δ_C 141.1, 138.2, 133.0, 128.9, 128.8, 127.5, 126.9, 121.2, 121.0, 119.8, 109.3, 50.9, 36.5 ppm.

HRMS (ESI) m/z calc'd for $C_{15}H_{14}N_2Cl$ $[M+H]^+$, 257.0846; found, 257.0839.

3-Chloro-1-isopropyl-1*H*-indazole (55)

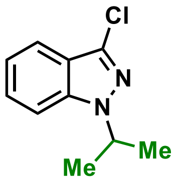

Prepared following **General Procedure B**, using 3-chloro-1*H*-indazole (32.1 mg, 200 µmol, 1.00 equiv., 95% purity), and 2-isopropyl-4,4,5,5-tetramethyl-1,3,2-dioxaborolane (68.0 mg, 400 µmol, 2.00 equiv.) in MeCN (2.0 mL, *c* = 0.10 M). Purification by CombiFlash® R_f purification system, eluting with EtOAc/heptane (0 – 20%, v/v), gave **55** (38.5 mg, 99%) as a colorless oil.

NMR Spectroscopy (*see spectra*):

^{1}**H NMR** (400 MHz, $CDCl_3$): δ_H 7.67 (d, *J* = 8.2 Hz, 1H), 7.43 – 7.35 (m, 2H), 7.19 (dq, *J* = 8.0, 4.5 Hz, 1H), 4.79 (hept, *J* = 6.6 Hz, 1H), 1.57 (d, *J* = 5.2 Hz, 6H) ppm.

^{13}**C NMR** (101 MHz, $CDCl_3$): δ_C 140.1, 132.4, 127.2, 121.2, 121.2, 119.9, 109.5, 50.9, 22.2 ppm.

All recorded spectroscopic data matched those previously reported in the literature.[1]

3-Chloro-1-(oxetan-3-yl)-1*H*-indazole (56)

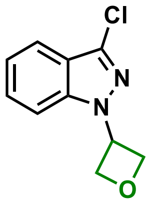

Prepared following **General Procedure B**, using 3-chloro-1*H*-indazole (32.1 mg, 200 µmol, 1.00 equiv., 95% purity), and 4,4,5,5-tetramethyl-2-(oxetan-3-yl)-1,3,2-dioxaborolane (73.6 mg, 400 µmol, 2.00 equiv.) in MeCN (2.0 mL, *c* = 0.10 M). Purification by CombiFlash® R_f purification system, eluting with EtOAc/heptane (0 – 20%, v/v), gave **56** (40.0 mg, 96%) as a colorless solid.

NMR Spectroscopy (*see spectra*):

^{1}**H NMR** (400 MHz, $CDCl_3$): δ_H 7.71 (d, *J* = 8.2 Hz, 1H), 7.49 (d, *J* = 6.7 Hz, 2H), 7.26 (t, *J* = 7.3 Hz, 1H), 5.83 – 5.63 (m, 1H), 5.30 (t, *J* = 6.6 Hz, 2H), 5.12 (t, *J* = 7.3 Hz, 2H) ppm.

^{13}**C NMR** (101 MHz, $CDCl_3$): δ_C 140.5, 134.2, 128.0, 121.9, 121.8, 120.2, 109.3, 53.0 ppm.

All recorded spectroscopic data matched those previously reported in the literature.[8]

***tert*-butyl 6-(4,4,5,5-tetramethyl-1,3,2-dioxaborolan-2-yl)-2-azaspiro[3.3]heptane-2-carboxylate (S57)**

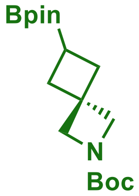

Copper chloride (20.8 mg, 0.155 mmol, 5.00 mol%), bis(pinacolato)diboron (1.18 g, 4.64 mmol, 1.50 equiv.), and Xantphos (107 mg, 0.186 mmol, 6.00 mol%) were placed in an oven-dried reaction flask. The vial was sealed with a screw cap containing a Teflon-coated rubber septum. The vial was connected to a vacuum/nitrogen manifold through a needle, evacuated and backfilled with nitrogen. THF (15 mL, c = 0.21 M) and KO*t*Bu (1.00 M in THF, 4.64 mL, 4.64 mmol, 1.50 equiv.) were added to the vial through the rubber septum. *t*ert-Butyl 6-iodo-2-azaspiro[3.3]heptane-2-carboxylate (1.00 g, 3.09 mmol, 1.00 equiv.) was added. After the reaction was complete, the reaction mixture was passed through a short silica column eluting with ethyl acetate/hexane (10:90). The crude mixture was further purified by CombiFlash® R_f purification system, eluting with EtOAc/heptane (0 – 20%, v/v), gave **S57** (741 mg, 74%) as a colorless solid.

NMR Spectroscopy (*see spectra*):

^{1}**H NMR** (400 MHz, $CDCl_3$): δ_H 3.84 (d, *J* = 9.4 Hz, 4H), 2.24 (dd, *J* = 11.7, 9.5 Hz, 2H), 2.17 – 2.10 (m, 2H), 1.68 (tt, *J* = 9.5, 7.2 Hz, 1H), 1.42 (s, 9H, Bpin), 1.23 (s, 12H, Boc) ppm.

^{13}**C NMR** (101 MHz, $CDCl_3$): δ_C 156.5, 83.4, 79.2, 62.0, 61.7, 37.8, 34.6, 28.6, 24.8 ppm.

^{11}**B NMR** (128 MHz, $CDCl_3$): δ_C 34.30 (brs) ppm.

HRMS (ESI) m/z calc'd for $C_{17}H_{31}NO_4B$ $[M+H]^+$, 324.2346; found, 324.2350.

tert-Butyl 6-(3-chloro-1_H_-indazol-1-yl)-2-azaspiro[3.3]heptane-2-carboxylate (57)

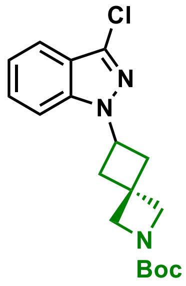

Prepared following **General Procedure B**, using 3-chloro-1*H*-indazole (32.1 mg, 200 μmol, 1.00 equiv., 95% purity), and *tert*-butyl 6-(4,4,5,5-tetramethyl-1,3,2-dioxaborolan-2-yl)-2-azaspiro[3.3]heptane-2-carboxylate (129 mg, 400 μmol, 2.00 equiv.) in MeCN (4.0 mL, *c* = 0.050 M). Purification by CombiFlash® R_f purification system, eluting with EtOAc/heptane (0 – 40%, v/v), gave **57** (63.9 mg, 92%) as a colorless solid.

NMR Spectroscopy (<u>see spectra</u>):

^{1}**H NMR** (400 MHz, $CDCl_3$): δ_H 7.64 (d, *J* = 8.2 Hz, 1H), 7.44 – 7.37 (m, 1H), 7.34 (d, *J* = 8.5 Hz, 1H), 7.22 – 7.13 (m, 1H), 4.97 – 4.81 (m, 1H), 4.08 (s, 2H), 3.98 (s, 2H), 2.98 – 2.88 (m, 2H), 2.73 (ddd, *J* = 10.4, 7.7, 3.0 Hz, 2H), 1.44 (s, 9H) ppm.

^{13}**C NMR** (101 MHz, $CDCl_3$): δ_C 156.2, 140.5, 133.2, 127.5, 121.5, 121.3, 119.9, 109.3, 79.6, 61.7, 60.3, 47.8, 40.6, 31.8, 28.5 ppm.

All recorded spectroscopic data matched those previously reported in the literature.[8]

tert-Butyl 3-(3-chloro-1_H_-indazol-1-yl)azetidine-1-carboxylate (58)

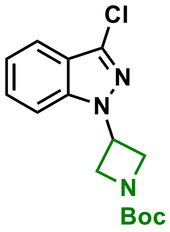

Prepared following **General Procedure B**, using 3-chloro-1*H*-indazole (32.1 mg, 200 μmol, 1.00 equiv., 95% purity), and *tert*-butyl 3-(4,4,5,5-tetramethyl-1,3,2-dioxaborolan-2-yl)azetidine-1-carboxylate (113 mg, 400 μmol, 2.00 equiv.) in MeCN (4.0 mL, *c* = 0.050 M). Purification by CombiFlash® R_f purification system, eluting with EtOAc/heptane (0 – 40%, v/v), gave **58** (60.4 mg, 98%) as a colorless solid.

NMR Spectroscopy (<u>see spectra</u>):

^{1}**H NMR** (400 MHz, $CDCl_3$): δ_H 7.67 (d, *J* = 8.2 Hz, 1H), 7.48 – 7.36 (m, 2H), 7.22 (ddd, *J* = 7.9, 6.4, 1.2 Hz, 1H), 5.32 (tt, *J* = 8.0, 5.6 Hz, 1H), 4.50 (dd, *J* = 9.2, 5.6 Hz, 2H), 4.41 (t, *J* = 8.6 Hz, 2H), 1.47 (s, 9H) ppm.

^{13}**C NMR** (101 MHz, $CDCl_3$): δ_C 156.2, 140.7, 134.2, 128.0, 121.9, 121.8, 120.2, 109.2, 80.1, 56.0, 47.5, 28.5, 28.5 ppm.

All recorded spectroscopic data matched those previously reported in the literature.[8]

3-Chloro-1-cyclopentyl-1*H*-indazole (59)

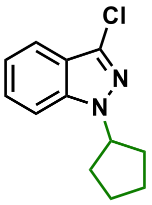

Prepared following **General Procedure B**, using 3-chloro-1*H*-indazole (32.1 mg, 200 μmol, 1.00 equiv., 95% purity), and 2-cyclopentyl-4,4,5,5-tetramethyl-1,3,2-dioxaborolane (78.4 mg, 400 μmol, 2.00 equiv.) in MeCN (2.0 mL, *c* = 0.10 M). Purification by CombiFlash® R_f purification system, eluting with EtOAc/heptane (0 – 20%, v/v), gave **59** (41.8 mg, 95%) as a colorless oil.

NMR Spectroscopy (see spectra):

^{1}H NMR (400 MHz, $CDCl_3$): δ_H 7.66 (dt, *J* = 8.1, 1.0 Hz, 1H), 7.48 – 7.36 (m, 2H), 7.18 (ddd, *J* = 7.9, 6.0, 1.6 Hz, 1H), 5.01 – 4.87 (m, 1H), 2.16 (tdd, *J* = 7.5, 4.9, 1.9 Hz, 4H), 1.98 (ddd, *J* = 10.4, 7.5, 4.9 Hz, 2H), 1.80 – 1.66 (m, 2H) ppm.

^{13}C NMR (101 MHz, $CDCl_3$): δ_C 140.7, 132.2, 127.1, 121.3, 121.1, 119.9, 109.7, 60.0, 32.4, 24.7 ppm.

All recorded spectroscopic data matched those previously reported in the literature.[1]

3-Chloro-1-(tetrahydrofuran-2-yl)-1*H*-indazole (60)

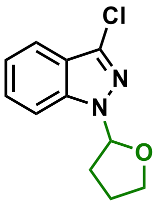

Prepared following **General Procedure B**, using 3-chloro-1*H*-indazole (32.1 mg, 200 μmol, 1.00 equiv., 95% purity), and 4,4,5,5-tetramethyl-2-(tetrahydrofuran-2-yl)-1,3,2-dioxaborolane (79.2 mg, 400 μmol, 2.00 equiv.) in MeCN (2.0 mL, *c* = 0.10 M). Purification by CombiFlash® R_f purification system, eluting with EtOAc/heptane (0 – 20%, v/v), gave **60** (38.9 mg, 87%) as a colorless oil.

NMR Spectroscopy (see spectra):

^{1}H NMR (400 MHz, $CDCl_3$): δ_H 7.66 (d, *J* = 8.1 Hz, 1H), 7.58 (d, *J* = 8.5 Hz, 1H), 7.44 (ddd, *J* = 8.4, 6.9, 1.1 Hz, 1H), 7.22 (t, *J* = 7.5 Hz, 1H), 6.31 (t, *J* = 6.6 Hz, 1H), 4.01 (dtd, *J* = 21.7, 8.2, 6.1 Hz, 2H), 2.98 – 2.84 (m, 1H), 2.39 (tdt, *J* = 14.4, 10.9, 7.4 Hz, 2H), 2.16 – 2.00 (m, 1H) ppm.

^{13}C NMR (101 MHz, $CDCl_3$): δ_C 141.3, 134.3, 127.9, 122.0, 121.9, 119.8, 110.2, 87.3, 69.0, 30.4, 25.1 ppm.

All recorded spectroscopic data matched those previously reported in the literature.[9]

3-Chloro-1-(tetrahydro-2*H*-pyran-4-yl)-1*H*-indazole (61)

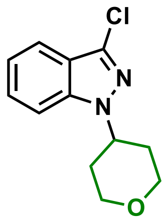

Prepared following **General Procedure B**, using 3-chloro-1*H*-indazole (32.1 mg, 200 μmol, 1.00 equiv., 95% purity), and 4,4,5,5-tetramethyl-2-(tetrahydro-2H-pyran-4-yl)-1,3,2-dioxaborolane (84.8 mg, 400 μmol, 2.00 equiv.) in MeCN (4.0 mL, *c* = 0.050 M). Purification by CombiFlash® R_f purification system, eluting with EtOAc/heptane (0 – 20%, v/v), gave **61** (43.9 mg, 93%) as a colorless oil.

NMR Spectroscopy (*see spectra*):

^{1}H NMR (400 MHz, $CDCl_3$): δ_H 7.68 (dd, *J* = 8.2, 1.1 Hz, 1H), 7.48 – 7.37 (m, 2H), 7.20 (ddd, *J* = 7.9, 5.8, 1.9 Hz, 1H), 4.59 (tt, *J* = 11.5, 4.2 Hz, 1H), 4.20 – 4.11 (m, 2H), 3.59 (td, *J* = 12.0, 2.0 Hz, 2H), 2.39 (dtd, *J* = 13.2, 11.8, 4.6 Hz, 2H), 1.96 (ddq, *J* = 12.9, 4.3, 2.2 Hz, 2H) ppm.

^{13}C NMR (101 MHz, $CDCl_3$): δ_C 140.1, 132.9, 127.4, 121.4, 121.4, 120.1, 109.3, 67.3, 55.8, 32.6, 32.5 ppm.

All recorded spectroscopic data matched those previously reported in the literature.[8]

***tert*-Butyl 4-(3-chloro-1*H*-indazol-1-yl)piperidine-1-carboxylate (62)**

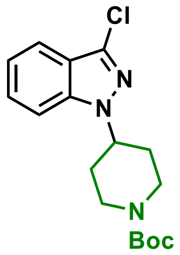

Prepared following **General Procedure B**, using 3-chloro-1*H*-indazole (32.1 mg, 200 μmol, 1.00 equiv., 95% purity), and *tert*-butyl 4-(4,4,5,5-tetramethyl-1,3,2-dioxaborolan-2-yl)piperidine-1-carboxylate (124 mg, 400 μmol, 2.00 equiv.) in MeCN (4.0 mL, *c* = 0.050 M). Purification by CombiFlash® R_f purification system, eluting with EtOAc/heptane (0 – 20%, v/v), gave **62** (66.3 mg, 99%) as a colorless oil.

NMR Spectroscopy (*see spectra*):

^{1}H NMR (400 MHz, $CDCl_3$): δ_H 7.67 (d, *J* = 8.2 Hz, 1H), 7.41 (d, *J* = 3.8 Hz, 2H), 7.20 (dq, *J* = 8.0, 4.6 Hz, 1H), 4.49 (ddt, *J* = 11.5, 8.4, 4.1 Hz, 1H), 4.30 (d, *J* = 13.0 Hz, 2H), 2.93 (t, *J* = 13.1 Hz, 2H), 2.19 (td, *J* = 12.0, 3.8 Hz, 2H), 1.98 (dd, *J* = 13.1, 3.7 Hz, 2H), 1.48 (s, 9H) ppm.

^{13}C NMR (101 MHz, $CDCl_3$): δ_C 154.6, 140.2, 132.9, 127.5, 121.5, 121.3, 120.1, 109.3, 80.0, 56.7, 43.1, 31.6, 28.6, 24.9 ppm.

All recorded spectroscopic data matched those previously reported in the literature.[8]

3-Chloro-1-(1,4-dioxaspiro[4.5]decan-8-yl)-1*H*-indazole (63)

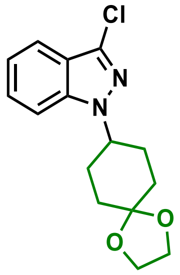

Prepared following **General Procedure B**, using 3-chloro-1*H*-indazole (32.1 mg, 200 µmol, 1.00 equiv., 95% purity), and 4,4,5,5-tetramethyl-2-(1,4-dioxaspiro[4.5]decan-8-yl)-1,3,2-dioxaborolane (107 mg, 400 µmol, 2.00 equiv.) in MeCN (4.0 mL, *c* = 0.050 M). Purification by CombiFlash® R_f purification system, eluting with EtOAc/heptane (0 – 20%, v/v), gave **63** (57.3 mg, 98%) as a colorless oil.

NMR Spectroscopy (see spectra):

^{1}**H NMR** (400 MHz, $CDCl_3$): δ_H 7.65 (d, *J* = 8.2 Hz, 1H), 7.47 – 7.34 (m, 2H), 7.17 (ddd, *J* = 7.9, 6.6, 1.1 Hz, 1H), 4.51 – 4.37 (m, 1H), 3.98 (m, 4H), 2.40 (qd, *J* = 13.0, 3.8 Hz, 2H), 2.06 – 1.90 (m, 4H), 1.77 (td, *J* = 13.5, 4.2 Hz, 2H) ppm.

^{13}**C NMR** (101 MHz, $CDCl_3$): δ_C 140.1, 132.4, 127.2, 121.3, 121.2, 119.9, 109.6, 107.7, 64.6, 64.5, 57.4, 33.8, 29.3 ppm.

All recorded spectroscopic data matched those previously reported in the literature.[8]

3-(3-Chloro-1*H*-indazol-1-yl)cyclohexan-1-one (64)

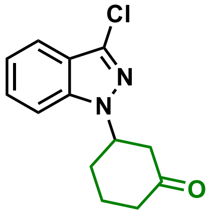

Prepared following **General Procedure B**, using 3-chloro-1*H*-indazole (32.1 mg, 200 µmol, 1.00 equiv., 95% purity), and 3-(4,4,5,5-tetramethyl-1,3,2-dioxaborolan-2-yl)cyclohexan-1-one (89.6 mg, 400 µmol, 2.00 equiv.) in MeCN (4.0 mL, *c* = 0.050 M). Purification by CombiFlash® R_f purification system, eluting with EtOAc/heptane (0 – 20%, v/v), gave **64** (45.6 mg, 92%) as a colorless oil.

NMR Spectroscopy (see spectra):

^{1}**H NMR** (400 MHz, $CDCl_3$): δ_H 7.68 (d, *J* = 8.2 Hz, 1H), 7.44 (ddd, *J* = 8.0, 6.8, 1.2 Hz, 1H), 7.37 (d, *J* = 8.5 Hz, 1H), 7.26 – 7.18 (m, 1H), 4.76 (tt, *J* = 11.1, 4.3 Hz, 1H), 3.17 (dd, *J* = 14.3, 11.2 Hz, 1H), 2.78 (ddt, *J* = 14.3, 3.6, 1.7 Hz, 1H), 2.55 – 2.42 (m, 2H), 2.42 – 2.11 (m, 3H), 1.75 (dddt, *J* = 20.3, 10.2, 6.4, 4.2 Hz, 1H) ppm.

^{13}**C NMR** (101 MHz, $CDCl_3$): δ_C 208.1, 140.1, 133.6, 127.8, 121.8, 121.7, 121.3, 120.1, 119.7, 109.0, 56.9, 47.6, 40.8, 31.1, 22.2 ppm.

HRMS (ESI) m/z calc'd for $C_{13}H_{14}N_2OCl$ $[M+H]^+$, 249.0795; found, 249.0794.

3-Chloro-1-(4,4-difluorocyclohexyl)-1*H*-indazole (65)

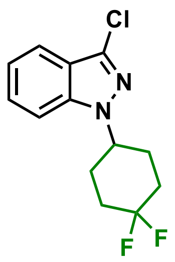

Prepared following **General Procedure B**, using 3-chloro-1*H*-indazole (32.1 mg, 200 μmol, 1.00 equiv., 95% purity), and 2-(4,4-difluorocyclohexyl)-4,4,5,5-tetramethyl-1,3,2-dioxaborolane (98.4 mg, 400 μmol, 2.00 equiv.) in MeCN (2.0 mL, *c* = 0.10 M). Purification by CombiFlash® R_f purification system, eluting with EtOAc/heptane (0 – 40%, v/v), gave **65** (48.3 mg, 89%) as a colorless solid.

NMR Spectroscopy (*see spectra*):

^{1}H NMR (400 MHz, $CDCl_3$): δ_H 7.69 (d, *J* = 8.1 Hz, 1H), 7.48 – 7.37 (m, 2H), 7.21 (t, *J* = 7.3 Hz, 1H), 4.57–4.45 (m, 1H), 2.53 – 2.26 (m, 4H), 2.19 – 1.88 (m, 4H) ppm.

^{13}C NMR (101 MHz, $CDCl_3$): δ_C 140.3, 133.0, 127.6, 122.3 (t, *J* = 241.4 Hz), 121.5, 121.4, 120.2, 109.3, 55.8, 32.7 (t, *J* = 25.0 Hz), 28.2 (d, *J* = 9.1 Hz) ppm.

^{19}F NMR (376 MHz, $CDCl_3$): δ_F –94.9 (d, *J* = 238.3 Hz, 1F), –100.6 (dt, *J* = 233.6 Hz, 1F) ppm.

All recorded spectroscopic data matched those previously reported in the literature.[8]

3-Chloro-1-(1-tosylpiperidin-4-yl)-1*H*-indazole (66)

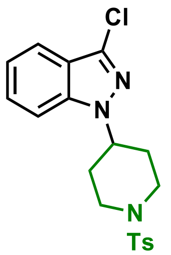

Prepared following **General Procedure B**, using 3-chloro-1*H*-indazole (32.1 mg, 200 μmol, 1.00 equiv., 95% purity), and 4-(4,4,5,5-tetramethyl-1,3,2-dioxaborolan-2-yl)-1-tosylpiperidine (146 mg, 400 μmol, 2.00 equiv.) in MeCN (4.0 mL, *c* = 0.050 M). Purification by CombiFlash® R_f purification system, eluting with EtOAc/heptane (0 – 40%, v/v), gave **66** (70.8 mg, 91%) as a colorless solid.

NMR Spectroscopy (*see spectra*):

^{1}H NMR (400 MHz, $CDCl_3$): δ_H 7.68 (d, *J* = 7.8 Hz, 2H), 7.64 (d, *J* = 8.2 Hz, 1H), 7.36 (t, *J* = 6.3 Hz, 3H), 7.30 (d, *J* = 8.6 Hz, 1H), 7.18 (t, *J* = 7.5 Hz, 1H), 4.31 (tt, *J* = 11.3, 4.3 Hz, 1H), 3.93 (d, *J* = 11.8 Hz, 2H), 2.57 (t, *J* = 11.9 Hz, 2H), 2.46 – 2.36 (m, 2H), 2.45 (s, 3H), 2.05 (d, *J* = 12.9 Hz, 2H) ppm.

^{13}C NMR (101 MHz, $CDCl_3$): δ_C 143.9, 140.1, 133.3, 133.0, 129.9, 127.8, 127.5, 121.5, 121.4, 120.1, 109.1, 55.6, 45.6, 30.8, 21.7 ppm.

HRMS (ESI) m/z calc'd for $C_{19}H_{21}N_3O_2SCl$ $[M+H]^+$, 390.1043; found, 390.1038.

***tert*-Butyl 3-(3-chloro-1*H*-indazol-1-yl)-8-azabicyclo[3.2.1]octane-8-carboxylate (67)**

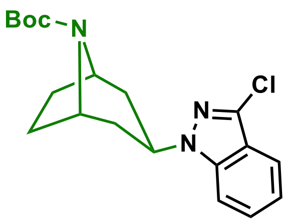

Prepared following **General Procedure B**, using 3-chloro-1*H*-indazole (32.1 mg, 200 µmol, 1.00 equiv., 95% purity), and 8-Boc-3-(4,4,5,5-tetramethyl-[1,3,2]dioxaborolan-2-yl)-8-aza-bicyclo[3.2.1]octane (135 mg, 400 µmol, 2.00 equiv.) in MeCN (4.0 mL, *c* = 0.050 M). Purification by CombiFlash® R_f purification system, eluting with EtOAc/heptane (0 – 40%, v/v), gave **67** (68.8 mg, 95%) as a colorless solid.

NMR Spectroscopy (*see spectra*):

^{1}H NMR (400 MHz, $CDCl_3$): δ_H 7.66 (dt, *J* = 8.2, 1.1 Hz, 1H), 7.39 (m, 2H), 7.19 (dd, *J* = 8.0, 4.4 Hz, 1H), 4.95 (tt, *J* = 11.8, 5.7 Hz, 1H), 4.45 (m, 1H), 4.37 (m, 1H), 2.58–2.36 (m, 2H), 2.16–2.08 (m, 2H), 1.97–1.90 (m, 2H), 1.89–1.79 (m, 2H), 1.53 (s, 9H) ppm.

^{13}C NMR (101 MHz, $CDCl_3$): δ_C 153.3, 139.7, 132.5, 127.1, 121.6, 121.2, 120.1, 109.5, 79.7, 53.3, 52.4, 36.6, 36.3, 28.5, 28.4, 27.4 ppm.

All recorded spectroscopic data matched those previously reported in the literature.[8]

1-(Bicyclo[2.2.1]heptan-2-yl)-3-chloro-1*H*-indazole (68)

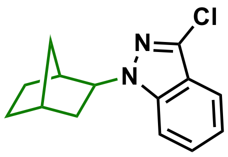

Prepared following **General Procedure B**, using 3-chloro-1*H*-indazole (32.1 mg, 200 µmol, 1.00 equiv., 95% purity), and 2-(bicyclo[2.2.1]heptan-2-yl)-4,4,5,5-tetramethyl-1,3,2-dioxaborolane (98.4 mg, 400 µmol, 2.00 equiv.) in MeCN (4.0 mL, *c* = 0.050 M). Purification by CombiFlash® R_f purification system, eluting with EtOAc/heptane (0 – 20%, v/v), gave **68** (48.3 mg, 98%) as a colorless oil.

NMR Spectroscopy (*see spectra*):

^{1}H NMR (400 MHz, $CDCl_3$): δ_H 7.66 (d, *J* = 8.2 Hz, 1H), 7.40 (d, *J* = 5.6 Hz, 2H), 7.18 (ddd, *J* = 7.8, 5.7, 1.9 Hz, 1H), 4.41 (dd, *J* = 8.4, 3.3 Hz, 1H), 2.53 – 2.43 (m, 3H), 2.05 – 1.97 (m, 1H), 1.88 (ddd, J = 11.5, 8.1, 2.3 Hz, 1H), 1.64 (td, J = 11.5, 3.5 Hz, 2H), 1.37– 1.21 (m, 3H) ppm.

^{13}C NMR (101 MHz, $CDCl_3$): δ_C 140.6, 131.7, 127.0, 121.4, 121.2, 119.8, 109.8, 61.6, 43.1, 37.3, 36.0, 28.8, 27.4 ppm.

All recorded spectroscopic data matched those previously reported in the literature.[10]

3-Chloro-1-(1-methylcyclopropyl)-1*H*-indazole (69)

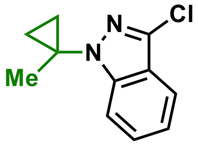

Prepared following **General Procedure B**, using 3-chloro-1*H*-indazole (32.1 mg, 200 μmol, 1.00 equiv., 95% purity), and 4,4,5,5-tetramethyl-2-(1-methylcyclopropyl)-1,3,2-dioxaborolane (72.8 mg, 400 μmol, 2.00 equiv.) in MeCN (4.0 mL, *c* = 0.050 M). Purification by CombiFlash® R_f purification system, eluting with EtOAc/heptane (0 – 10%, v/v), gave **69** (34.9 mg, 84%) as a colorless oil.

NMR Spectroscopy (*see spectra*):

^{1}**H NMR** (400 MHz, $CDCl_3$): δ_H 7.66 (d, *J* = 8.1 Hz, 1H), 7.58 (d, *J* = 8.5 Hz, 1H), 7.43 (ddd, *J* = 8.4, 6.8, 1.1 Hz, 1H), 7.20 (dd, *J* = 8.0, 6.9 Hz, 1H), 1.58 (s, 3H), 1.39 – 1.33 (m, 2H), 1.06 (d, *J* = 2.0 Hz, 2H) ppm.

^{13}**C NMR** (101 MHz, $CDCl_3$): δ_C 140.9, 132.8, 127.5, 121.6, 121.5, 120.1, 110.4, 36.9, 23.1, 14.2 ppm.

HRMS (ESI) m/z calc'd for $C_{11}H_{12}N_2Cl$ $[M+H]^+$, 207.0689; found, 207.0686.

Methyl 3-(3-chloro-1*H*-indazol-1-yl)bicyclo[1.1.1]pentane-1-carboxylate (70)

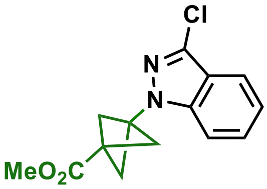

Prepared following **General Procedure B**, using 3-chloro-1*H*-indazole (32.1 mg, 200 μmol, 1.00 equiv., 95% purity), and 2-((1*r*,5*R*,7*S*)-adamantan-2-yl)-4,4,5,5-tetramethyl-1,3,2-dioxaborolane (101 mg, 400 μmol, 2.00 equiv.) in MeCN (4.0 mL, *c* = 0.050 M). Purification by CombiFlash® R_f purification system, eluting with EtOAc/heptane (0 – 10%, v/v), gave **70** (54.9 mg, 99%) as a colorless oil.

NMR Spectroscopy (*see spectra*):

^{1}**H NMR** (400 MHz, $CDCl_3$): δ_H 7.66 (dd, *J* = 8.2, 1.2 Hz, 1H), 7.53 – 7.39 (m, 2H), 7.28 – 7.18 (m, 1H), 3.76 (s, 3H), 2.75 (s, 6H) ppm.

^{13}**C NMR** (101 MHz, $CDCl_3$): δ_C 169.6, 140.6, 134.3, 128.1, 121.8, 121.8, 120.1, 110.2, 55.1, 52.2, 50.9, 35.4 ppm.

All recorded spectroscopic data matched those previously reported in the literature.[1]

Methyl 1-((3*s*,5*s*,7*s*)-adamantan-1-yl)-6-fluoro-1*H*-indazole-3-carboxylate (71)

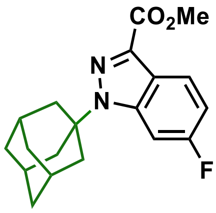

Prepared following **General Procedure B**, using methyl 6-fluoro-1*H*-indazole-3-carboxylate (40.5 mg, 200 μmol, 1.00 equiv., 96% purity), and methyl 3-(4,4,5,5-tetramethyl-1,3,2-dioxaborolan-2-yl)-1l5,3l5-tricyclo[1.1.1.01,3]-pentane-1-carboxylate (105 mg, 400 μmol, 2.00 equiv.) in MeCN (4.0 mL, *c* = 0.050 M). Purification by CombiFlash® R_f purification system, eluting with EtOAc/heptane (0 – 10%, v/v), gave **71** (50.9 mg, 77%) as a colorless solid.

NMR Spectroscopy (see spectra):

^{1}**H NMR** (400 MHz, $CDCl_3$): δ_H 8.21 (dd, *J* = 9.0, 5.6 Hz, 1H), 7.47 (dd, *J* = 10.2, 2.1 Hz, 1H), 7.05 (td, *J* = 8.9, 2.1 Hz, 1H), 4.01 (s, 3H), 2.44 (d, *J* = 3.0 Hz, 6H), 2.31 (s, 3H), 1.82 (s, 6H) ppm.

^{13}**C NMR** (101 MHz, $CDCl_3$): δ_C 163.3,162.1 (d, *J* = 246.4 Hz), 139.2 (d, *J* = 12.0 Hz), 133.5, 123.3 (d, *J* = 10.8 Hz), 120.8, 112.6 (d, *J* = 25.8 Hz), 99.0 (d, *J* = 27.6 Hz), 62.3, 52.2, 41.9, 36.2, 29.8 ppm.

^{19}**F NMR** (376 MHz, $CDCl_3$): δ_F –114.29 (d, *J* = 6.0 Hz) ppm.

HRMS (ESI) m/z calc'd for $C_{19}H_{22}N_2O_2F$ $[M+H]^+$, 329.1665; found, 329.1664.

Methyl 1-(*tert*-butyl)-6-fluoro-1*H*-indazole-3-carboxylate (72)

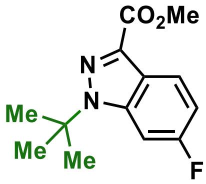

Prepared following **General Procedure B**, using methyl 6-fluoro-1*H*-indazole-3-carboxylate (40.5 mg, 200 μmol, 1.00 equiv., 96% purity), BTMG (171 mg, 1.00 mmol, 0.200 mL, 5.00 equiv.) and 2-(*tert*-butyl)-4,4,5,5-tetramethyl-1,3,2-dioxaborolane (184 mg, 1.00 mmol, 5.00 equiv.) in MeCN (4.0 mL, *c* = 0.050 M). Purification by CombiFlash® R_f purification system, eluting with EtOAc/heptane (0 – 20%, v/v), gave **71** (19.8 mg, 40%) as a colorless solid.

NMR Spectroscopy (see spectra):

^{1}**H NMR** (400 MHz, $CDCl_3$): δ_H 8.22 (dd, *J* = 9.0, 5.5 Hz, 1H), 7.39 (dd, *J* = 10.0, 2.1 Hz, 1H), 7.07 (td, *J* = 8.9, 2.1 Hz, 1H), 4.02 (s, 3H), 1.80 (s, 9H) ppm.

^{13}**C NMR** (101 MHz, $CDCl_3$): δ_C 163.3, 161.4 (d, *J* = 244.8 Hz), 139.4 (d, *J* = 12.0 Hz), 133.4, 124.0 (d, *J* = 10.8 Hz), 122.2, 112.6 (d, *J* = 25.6 Hz), 98.8 (d, *J* = 27.4 Hz), 61.4, 52.2, 29.6 ppm.

^{19}**F NMR** (376 MHz, $CDCl_3$): δ_F –114.04 (d, *J* = 6.0 Hz) ppm.

HRMS (ESI) m/z calc'd for $C_{19}H_{22}N_2O_2F$ $[M+H]^+$, 251.1196; found, 251.1193.

***N*-Alkylated T807 (73)**

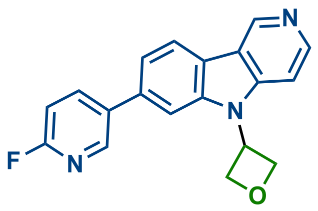

Prepared following **General Procedure C**, using T807 (54.3 mg, 200 µmol, 1.00 equiv., 97% purity), and 4,4,5,5-tetramethyl-2-(oxetan-3-yl)-1,3,2-dioxaborolane (74 mg, 0.40 mmol, 2.0 equiv.). Purification by CombiFlash® R_f purification system, eluting with DCM/MeOH (0 – 15%, v/v), gave **73** (58.3 mg, 91%) as a brown solid.

NMR Spectroscopy (<u>*see spectra*</u>):

^{1}**H NMR** (400 MHz, *d*$_6$-DMSO): δ_H 9.46 (s, 1H), 8.70 (d, *J* = 2.7 Hz, 1H), 8.56 (s, 1H), 8.43 (dd, *J* = 11.3, 8.0 Hz, 2H), 8.21 (s, 1H), 7.89 (d, *J* = 5.6 Hz, 1H), 7.70 (d, *J* = 8.1 Hz, 1H), 7.35 (dd, *J* = 8.6, 2.9 Hz, 1H), 6.21 (td, *J* = 7.7, 3.7 Hz, 1H), 5.33 – 5.13 (m , 4H) ppm.

^{13}**C NMR** (101 MHz, *d*$_6$-DMSO): δ_C 162.7 (d, *J* = 234.0 Hz), 145.5 (d, *J* = 15.3 Hz), 145.2, 143.6, 143.3, 140.4 (d, *J* = 8.0 Hz), 140.7, 134.8 (d, *J* = 4.4 Hz), 134.7, 121.6, 120.9, 119.9, 119.0, 109.5, 109.1, 105.8 75.1, 49.0 ppm.

^{13}**C NMR** (101 MHz, $CDCl_3$): δ_C 163.3 (d, *J* = 239.8 Hz), 146.2 (d, *J* = 14.9 Hz), 145.6, 144.2, 143.3, 140.2 (d, *J* = 7.9 Hz), 139.9, 136.0, 135.1 (d, *J* = 4.7 Hz), 121.9, 121.7, 120.6, 109.7 (d, *J* = 37.5 Hz), 108.6, 75.7, 49.6 ppm.

^{19}**F NMR** (376 MHz, *d*$_6$-DMSO): δ_F –71.15 (d, *J* = 8.1 Hz, 1F) ppm.

HRMS (ESI) m/z calc'd for $C_{19}H_{15}N_3OF$ $[M+H]^+$, 320.1199; found, 320.1202.

***N*-Alkylated carprofen (74)**

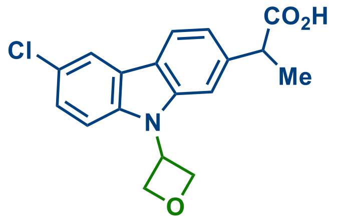

Prepared following **General Procedure C**, using carprofen (55.9 mg, 200 µmol, 1.00 equiv., 98% purity), BTMG (137 mg, 0.800 mmol, 0.160 mL, 4.00 equiv.) and 4,4,5,5-tetramethyl-2-(oxetan-3-yl)-1,3,2-dioxaborolane (110 mg, 0.60 mmol, 3.0 equiv.). Purification by CombiFlash® R_f purification system, eluting with DCM/MeOH (0 – 20%, v/v), gave **74** (48.9 mg, 74%) as a colorless solid.

NMR Spectroscopy (<u>*see spectra*</u>):

^{1}**H NMR** (400 MHz, $CDCl_3$): δ_H 8.04 – 7.92 (m, 2H), 7.75 (d, *J* = 8.7 Hz, 1H), 7.58 (s, 1H), 7.42 (dd, *J* = 8.7, 2.2 Hz, 1H), 7.23 (d, *J* = 8.1 Hz, 1H), 5.87 – 5.74 (m, 1H), 5.39 (t, *J* = 6.6 Hz, 2H), 5.26 (t, *J* = 7.7 Hz, 2H), 3.95 (q, *J* = 7.1 Hz, 1H), 1.64 (s, 3H) ppm.

^{13}**C NMR** (101 MHz, $CDCl_3$): δ_C 180.3, 140.4, 138.8, 137.8, 126.2, 125.5, 124.7, 121.9, 121.1, 120.4, 119.8, 111.0, 108.7, 49.4, 46.0, 18.7 ppm.

HRMS (ESI) m/z calc'd for $C_{18}H_{17}NO_3Cl$ $[M+H]^+$, 330.0897; found, 330.0901.

N-Alkylated rutecarpine (75)

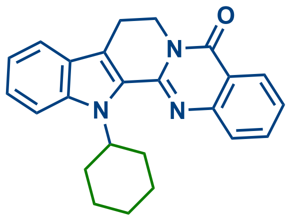

Prepared following **General Procedure B**, using rutecarpine (60.0 mg, 200 µmol, 1.00 equiv., 96% purity), and cyclohexylboronic acid pinacol ester (84 mg, 0.40 mmol, 2.0 equiv.) ***without d(OMe)Phen***. Purification by CombiFlash® R_f purification system, eluting with DCM/MeOH (0 – 5%, v/v), gave **75** (45.6 mg, 62%) as a colorless solid.

NMR Spectroscopy (*see spectra*):

^{1}**H NMR** (400 MHz, $CDCl_3$): δ_H 8.33 (dd, *J* = 8.0, 1.6 Hz, 1H), 7.73 (td, *J* = 7.5, 1.5 Hz, 2H), 7.68 – 7.59 (m, 2H), 7.48 – 7.40 (m, 1H), 7.31 (ddd, *J* = 8.5, 6.9, 1.3 Hz, 1H), 7.15 (t, *J* = 7.5 Hz, 1H), 5.97 (brs, 1H), 4.54 (t, *J* = 6.6 Hz, 2H), 3.12 (t, *J* = 6.6 Hz, 2H), 2.43 (qd, *J* = 12.6, 3.5 Hz, 2H), 2.14 – 1.97 (m, 4H), 1.90 – 1.80 (m, 1H), 1.57 (qt, J = 13.0, 3.5 Hz, 2H), 1.41 (tt, J = 13.1, 3.4 Hz, 1H) ppm.

^{13}**C NMR** (101 MHz, $CDCl_3$): δ_C 161.9, 147.4, 145.5, 139.2, 134.3, 134.2, 130.1, 128.8, 127.4, 127.2, 127.1, 126.3, 125.0, 124.5, 120.8, 120.3, 119.92, 119.89, 113.9, 56.9, 48.9, 40.6, 31.4, 26.7, 25.9, 20.0 ppm.

HRMS (ESI) m/z calc'd for $C_{24}H_{24}N_3O$ $[M+H]^+$, 370.1919; found, 370.1918.

N-Alkylated carvedilol (76)

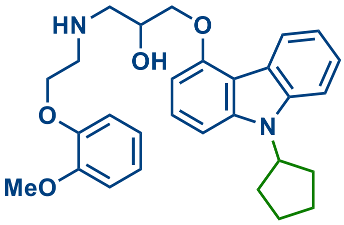

Prepared following **General Procedure C**, using carvedilol (81.3 mg, 200 µmol, 1.00 equiv., 99.9% purity), BTMG (137 mg, 0.800 mmol, 0.160 mL, 4.00 equiv.), and 2-cyclopentyl-4,4,5,5-tetramethyl-1,3,2-dioxaborolane (118 mg, 0.600 mmol, 3.00 equiv.). Purification by CombiFlash® R_f purification system, eluting with DCM/MeOH (0 – 15%, v/v), gave **76** (39.9 mg, 42%) as a colorless solid.

NMR Spectroscopy (see spectra):

^{1}**H NMR** (400 MHz, CD_3OD): δ_H 8.36 (d, J = 7.7 Hz, 1H), 7.50 (d, J = 8.3 Hz, 1H), 7.37 – 7.30 (m, 2H), 7.18 – 7.07 (m, 2H), 6.95 – 6.91 (m, 2H), 6.86 (dd, J = 6.0, 3.1 Hz, 1H), 6.72 (d, J = 8.0 Hz, 1H), 5.22 (p, J = 8.9 Hz, 1H), 4.32 (dt, J = 8.8, 4.6 Hz, 1H), 4.25 (qd, J = 9.7, 5.3 Hz, 2H), 4.20 – 4.08 (m, 2H), 3.75 (s, 3H), 3.15 – 3.02 (m, 3H), 2.96 (dd, J = 12.2, 8.1 Hz, 1H), 2.42 – 2.23 (m, 2H), 2.16 – 2.04 (m, 4H), 1.94 – 1.79 (m, 2H) ppm.

^{13}**C NMR** (101 MHz, CD_3OD): δ_C 156.8, 151.2, 149.5, 142.5, 140.2, 127.2, 125.4, 124.4, 123.8, 123.0, 122.1, 119.8, 115.7, 113.6, 113.3, 110.4, 104.3, 101.7, 71.6, 70.0, 69.5, 57.0, 56.3, 53.5, 29.9, 26.3 ppm.

HRMS (ESI) m/z calc'd for $C_{29}H_{35}N_2O_4$ $[M+H]^+$, 475.2597; found, 475.2591.

***N*-Alkylated fludioxonil (77)**

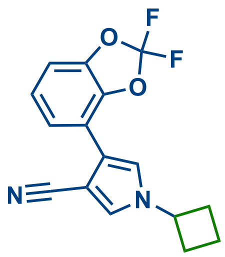

Prepared following **General Procedure C**, using fludioxonil (50.7 mg, 200 μmol, 1.00 equiv., 98% purity), and 2-cyclobutyl-4,4,5,5-tetramethyl-1,3,2-dioxaborolane (72.8 mg, 0.400 mmol, 2.00 equiv.). Purification by CombiFlash® R_f purification system, eluting with EtOAc/heptane (0 – 40%, v/v), gave **77** (54.2 mg, 90%) as a colorless solid.

NMR Spectroscopy (see spectra):

^{1}**H NMR** (400 MHz, $CDCl_3$): δ_H 7.69 (d, J = 8.2 Hz, 1H), 7.32 (d, J = 2.4 Hz, 1H), 7.21 (d, J = 2.4 Hz, 1H), 7.12 (t, J = 8.1 Hz, 1H), 7.02 – 6.87 (m, 1H), 4.57 (p, J = 8.4 Hz, 1H), 2.53 (dtq, J = 10.1, 7.8, 2.5 Hz, 2H), 2.38 (pd, J = 9.5, 2.8 Hz, 2H), 2.01 – 1.80 (m, 2H) ppm.

^{13}**C NMR** (101 MHz, $CDCl_3$): δ_C 143.9, 139.8, 131.5, 131.48 (d, J = 509.4 Hz), 127.8, 124.2, 121.4, 120.6, 118.5, 116.7 (d, J = 2.0 Hz), 107.8, 53.4, 31.0, 14.7 ppm.

^{19}**F NMR** (376 MHz, $CDCl_3$): δ_F –47.90 (s) ppm.

HRMS (ESI) m/z calc'd for $C_{16}H_{12}N_2O_2F_2Na$ $[M+Na]^+$, 325.0765; found, 325.0761.

***N*-Alkylated metaxalone (78)**

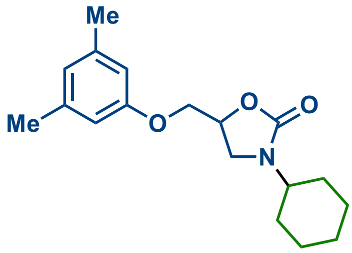

Prepared following **General Procedure C**, using metaxalone (44.7 mg, 200 µmol, 1.00 equiv., 99% purity), and cyclohexylboronic acid pinacol ester (84 mg, 0.40 mmol, 2.0 equiv.). Purification by CombiFlash® R_f purification system, eluting with EtOAc/heptane (0 – 20%, v/v), gave **78** (58.2 mg, 96%) as a colorless solid.

NMR Spectroscopy (*see spectra*):

^{1}**H NMR** (400 MHz, $CDCl_3$): δ_H 6.63 (s, 1H), 6.51 (s, 2H), 4.82 – 4.74 (m, 1H), 4.13 – 3.99 (m, 2H), 3.67 (dt, *J* = 17.6, 6.2 Hz, 2H), 3.48 (dd, *J* = 8.7, 5.7 Hz, 1H), 2.28 (s, 6H), 1.82 (d, *J* = 8.0 Hz, 4H), 1.72 – 1.63 (m, 1H), 1.36 (ddd, *J* = 11.8, 6.8, 2.6 Hz, 4H), 1.13 – 1.04 (m, 1H) ppm.

^{13}**C NMR** (101 MHz, $CDCl_3$): δ_C 158.3, 157.0, 139.5, 123.4, 112.4, 70.97, 68.1, 52.6, 43.0, 30.4, 30.3, 25.4, 25.4, 25.4, 21.5 ppm.

HRMS (ESI) m/z calc'd for $C_{18}H_{26}NO_3$ $[M+H]^+$, 304.1913; found, 304.1914.

N-Alkylated metaxalone (79)

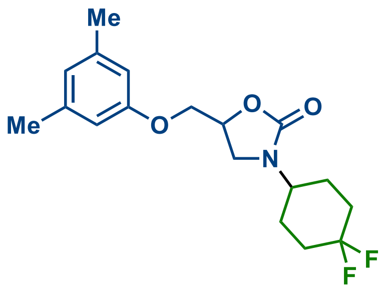

Prepared following **General Procedure C**, using metaxalone (44.7 mg, 200 µmol, 1.00 equiv., 99% purity), and 2-(4,4-difluorocyclohexyl)-4,4,5,5-tetramethyl-1,3,2-dioxaborolane (98 mg, 0.40 mmol, 2.0 equiv.). Purification by CombiFlash® R_f purification system, eluting with EtOAc/heptane (0 – 20%, v/v), gave **79** (58.2 mg, 86%) as a colorless oil.

NMR Spectroscopy (*see spectra*):

^{1}**H NMR** (400 MHz, $CDCl_3$): δ_H 6.63 (s, 1H), 6.51 (s, 2H), 4.82 – 4.74 (m, 1H), 4.13 – 3.99 (m, 2H), 3.67 (dt, *J* = 17.6, 6.2 Hz, 2H), 3.48 (dd, *J* = 8.7, 5.7 Hz, 1H), 2.28 (s, 6H), 1.82 (d, *J* = 8.0 Hz, 4H), 1.72 – 1.63 (m, 1H), 1.36 (ddd, *J* = 11.8, 6.8, 2.6 Hz, 4H), 1.13 – 1.04 (m, 1H) ppm.

^{13}**C NMR** (101 MHz, $CDCl_3$): δ_C 158.2, 157.1, 139.6, 123.5, 122.3 (t, *J* = 241.6 Hz), 112.4, 71.2, 68.0, 50.5, 42.7, 32.7 (t, *J* = 24.8 Hz), 26.0 (dd, *J* = 17.2 Hz) 21.5 ppm.

^{19}**F NMR** (376 MHz, $CDCl_3$): δ_F –94.02 (d, *J* = 238.8 Hz, 1F), –102.69 (dt, *J* = 233.6 Hz, 1F) ppm.

HRMS (ESI) m/z calc'd for $C_{18}H_{24}NO_3F_2$ $[M+H]^+$, 340.1724; found, 340.1724.

N-Alkylated tamsulosin (80)

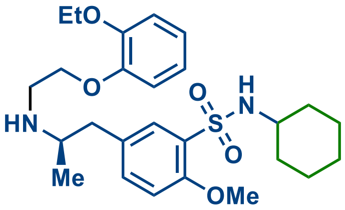

Prepared following **General Procedure C**, using tamsulosin (83.4 mg, 200 µmol, 1.00 equiv., 98% purity), and cyclohexylboronic acid pinacol ester (84 mg, 0.40 mmol, 2.0 equiv.). Purification by CombiFlash® R_f purification system, eluting with DCM/MeOH with 2% Et_3N (0 – 20%, v/v), gave **80** (93.9 mg, 96%) as a colorless oil.

NMR Spectroscopy (<u>see spectra</u>):

> **^{1}H NMR** (400 MHz, $CDCl_3$): δ_H 7.70 (d, J = 2.3 Hz, 1H), 7.37 (dd, J = 8.4, 2.3 Hz, 1H), 6.95 – 6.81 (m, 5H), 5.00 (d, J = 7.4 Hz, 1H), 4.16 (d, J = 5.3 Hz, 2H), 4.02 (q, J = 7.0 Hz, 2H), 3.91 (s, 3H), 3.17 (tq, J = 13.0, 6.3 Hz, 3H), 3.09 – 2.98 (m, 2H), 2.63 (dd, J = 13.4, 8.6 Hz, 1H), 1.63 (d, J = 10.3 Hz, 4H), 1.47 – 1.33 (m, 4H), 1.21 – 1.04 (m, 8H) ppm.

> **^{13}C NMR** (101 MHz, $CDCl_3$): δ_C 154.8, 149.0, 147.9, 135.3, 130.6, 130.3, 128.6, 122.2, 121.2, 120.8, 115.1, 113.6, 112.4, 75.0, 67.7, 64.5, 56.3, 55.1, 52.9, 45.7, 40.8, 33.6, 25.2, 24.9, 24.5, 18.2, 14.9 ppm.

HRMS (ESI) m/z calc'd for $C_{26}H_{39}N_2O_5S$ $[M+H]^+$, 491.2580; found, 491.2581.

N-Alkylated olaparib (81)

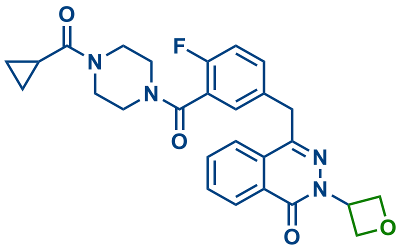

Prepared following **General Procedure C**, using olaparib (88.7 mg, 200 µmol, 1.00 equiv., 99% purity), and 2 4,4,5,5-tetramethyl-2-(oxetan-3-yl)-1,3,2-dioxaborolane (74 mg, 0.40 mmol, 2.0 equiv.). Purification by CombiFlash® R_f purification system, eluting with DCM/MeOH (0 – 10%, v/v), gave **81** (76.7 mg, 78%) as a colorless solid.

NMR Spectroscopy (<u>see spectra</u>):

> **^{1}H NMR** (400 MHz, $CDCl_3$): δ_H 8.43 (dd, J = 7.3, 2.3 Hz, 1H), 7.81 – 7.69 (m, 3H), 7.33 (dd, J = 10.2, 4.6 Hz, 2H), 7.06 (t, J = 8.9 Hz, 1H), 6.13 (p, J = 7.1 Hz, 1H), 5.01 (dt, J = 14.3, 7.4 Hz, 4H), 4.37 (s, 2H), 3.90 – 3.55 (m, 6H), 3.45 – 3.23 (d, J = 20.8 Hz, 2H), 1.79 – 1.68 (m, 1H), 0.99 (dt, J = 6.7, 3.3 Hz, 2H), 0.83 (d, J = 28.6 Hz, 2H) ppm.

> **^{13}C NMR** (101 MHz, $CDCl_3$): δ_C 172.4, 165.3, 159.1, 157.2 (d, J= 248.0 Hz), 145.1, 134.5 (d, J= 3.2 Hz), 133.6, 131.9 (d, J= 8.0 Hz), 131.8, 129.4 (bs), 129.3, 128.8, 127.9, 127.7,124.9, 123.9 (d, J= 16.8 Hz), 116.2 (d, J= 22.4 Hz), 51.6, 46.9, 45.3, 42.4, 38.0, 11.1, 7.9 ppm.

^{19}**F NMR** (376 MHz, $CDCl_3$): δ_F –117.47 ppm.

HRMS (ESI) m/z calc'd for $C_{27}H_{28}N_4O_4F$ $[M+H]^+$, 491.2095; found, 491.2097.

N-Alkylated sulfamethoxazole (82)

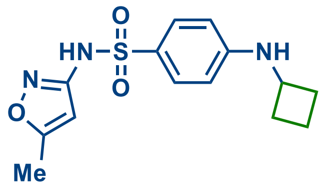

Prepared following **General Procedure C**, using sulfamethoxazole (51.7 mg, 200 μmol, 1.00 equiv., 98% purity), and 2-cyclobutyl-4,4,5,5-tetramethyl-1,3,2-dioxaborolane (73 mg, 0.40 mmol, 2.0 equiv.). Purification by CombiFlash® R_f purification system, eluting with DCM/MeOH (0 – 5%, v/v), gave **82** (38.1 mg, 62%) as a brown oil.

NMR Spectroscopy (<u>*see spectra*</u>):

^{1}**H NMR** (400 MHz, CD_3OD): δ_H 7.58 – 7.55 (m, 2H), 6.55 – 6.52 (m, 2H), 6.09 (d, *J* = 1.1 Hz, 1H), 3.92 (p, *J* = 7.4 Hz, 1H), 2.45 – 2.34 (m, 2H), 2.30 (d, *J* = 0.9 Hz, 3H), 1.95 – 1.74 (m, 4H) ppm.

^{13}**C NMR** (101 MHz, CD_3OD): δ_C 171.7, 159.6, 153.3, 130.2, 125.8, 112.4, 96.4, 31.4, 16.1, 12.3 ppm.

HRMS (ESI) m/z calc'd for $C_{14}H_{18}N_3O_3S$ $[M+H]^+$, 308.1069; found, 308.1066.

N-Alkylated tropisetron (83)

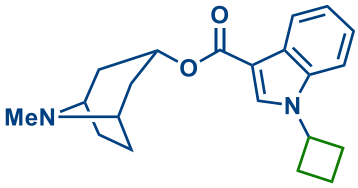

Prepared following **General Procedure C**, using tropisetron-HCl salt (65.0 mg, 200 μmol, 1.00 equiv., 99% purity), and 2-cyclobutyl-4,4,5,5-tetramethyl-1,3,2-dioxaborolane (73 mg, 0.40 mmol, 2.0 equiv.). Purification by CombiFlash® R_f purification system, eluting with DCM/MeOH with 2% Et_3N (0 – 5%, v/v), gave **83** (62.4 mg, 92%) as a colorless solid.

NMR Spectroscopy (<u>*see spectra*</u>):

^{1}**H NMR** (400 MHz, $CDCl_3$): δ_H 8.10 (dt, *J* = 7.9, 2.8 Hz, 1H), 7.87 (s, 1H), 7.39 (dt, *J* = 8.1, 2.7 Hz, 1H), 7.34 – 7.21 (m, 2H), 5.41 – 5.29 (m, 1H), 4.87 (ddd, *J* = 16.6, 9.2, 7.5 Hz, 1H), 3.83 (dt, *J* = 5.5, 2.7 Hz, 2H), 3.08 (s, 2H), 2.80 (s, 3H), 2.64 (ddtd, *J* = 15.5, 8.3, 4.9, 2.6 Hz, 2H), 2.58 – 2.40 (m, 4H), 2.35 (s, 2H), 2.25 (d, *J* = 15.9 Hz, 2H), 2.01 (ttd, *J* = 10.0, 6.8, 4.2 Hz, 2H) ppm.

^{13}**C NMR** (101 MHz, $CDCl_3$): δ_C 163.9, 136.3, 131.3, 126.7, 123.0, 122.3, 121.1, 110.8, 106.6, 64.0, 50.8, 31.0, 30.1, 24.9, 15.3 ppm.

HRMS (ESI) m/z calc'd for $C_{21}H_{27}N_2O_2$ $[M+H]^+$, 339.2073; found, 339.2072.

N-Alkylated topiramate (84)

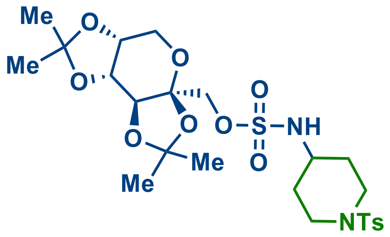

Prepared following **General Procedure C**, using topiramate (68.6 mg, 200 μmol, 1.00 equiv., 99% purity), and 4-(4,4,5,5-tetramethyl-1,3,2-dioxaborolan-2-yl)-1-tosylpiperidine (146 mg, 0.400 mmol, 2.00 equiv.). Purification by CombiFlash® R_f purification system, eluting with DCM/MeOH with 2% Et_3N (0 – 5%, v/v), gave **84** (78.8 mg, 68%) as a colorless solid.

NMR Spectroscopy (see spectra):

^{1}H NMR (400 MHz, $CDCl_3$): δ_H 7.61 (d, J = 8.0 Hz, 2H), 7.33 (d, J = 8.0 Hz, 2H), 5.11 (s, 1H, NH), 4.88 (d, J = 7.4 Hz, 1H), 4.62 – 4.57 (m, 1H), 4.32 – 4.21 (m, 3H), 4.14 (d, J = 10.4 Hz, 1H), 4.07 (d, J = 10.4 Hz, 1H), 3.92 – 3.85 (m, 1H), 3.75 (dd, J = 19.6, 13.0 Hz, 1H), 3.61 (d, J = 11.9 Hz, 2H), 3.27 (dq, J = 10.4, 3.1 Hz, 1H), 2.46 – 2.40 (m, 1H), 2.43 (s, 3H), 2.08 (dd, J = 13.3, 3.8 Hz, 2H), 1.69 – 1.41 (m, 8H), 1.37 – 1.31 (m, 6H) ppm.

^{13}C NMR (101 MHz, $CDCl_3$): δ_C 144.0, 132.7, 129.9, 127.7, 109.3, 109.3, 100.9, 71.0, 70.6, 70.4, 69.9, 61.3, 50.9, 44.9, 31.9, 26.6, 25.9, 25.3, 24.1, 21.7 ppm.

HRMS (ESI) m/z calc'd for $C_{24}H_{37}N_2O_{10}S_2$ $[M+H]^+$, 577.1890; found, 577.1888.

N-Alkylated sulpiride (85)

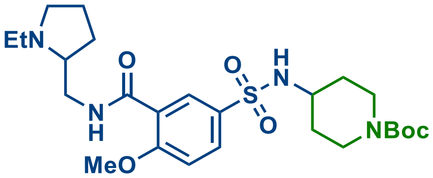

Prepared following **General Procedure C**, using sulpiride (69.7 mg, 200 μmol, 1.00 equiv., 98% purity), and *tert*-butyl 4-(4,4,5,5-tetramethyl-1,3,2-dioxaborolan-2-yl)piperidine-1-carboxylate (124 mg, 0.400 mmol, 2.00 equiv.). Purification by CombiFlash® R_f purification system, eluting with DCM/MeOH with 2% Et_3N (0 – 10%, v/v), gave **85** (77.4 mg, 74%) as a yellow solid.

NMR Spectroscopy (see spectra):

^{1}H NMR (400 MHz, $CDCl_3$): δ_H 8.72 (d, J = 2.5 Hz, 1H), 8.40 (d, J = 7.0 Hz, 1H), 7.98 (dd, J = 8.7, 2.5 Hz, 1H), 7.06 (d, J = 8.7 Hz, 1H), 5.73 (brs, 1H, NH), 4.01 (s, 3H), 3.87 (d, J = 12.1 Hz, 2H), 3.72 (ddd, J = 14.2, 7.2, 2.7 Hz, 1H), 3.33 (d, J = 13.8 Hz, 1H), 3.21 (d, J = 9.9 Hz, 2H), 2.91 – 2.63 (m, 4H), 2.23 (h, J = 7.5 Hz, 2H), 1.91 (dd, J = 11.9, 8.0 Hz, 1H), 1.77 – 1.58 (m, 5H), 1.41 – 1.35 (m, 1H), 1.39 (s, 9H), 1.12 (t, J = 7.2 Hz, 3H) ppm.

^{13}C NMR (101 MHz, $CDCl_3$): δ_C 164.0, 160.4, 154.6, 134.2, 131.6, 131.4, 122.8, 111.8, 79.7, 62.3, 56.4,

53.7, 51.0, 48.1, 42.2, 41.5, 32.8, 28.5, 23.0, 14.2 ppm.

HRMS (ESI) m/z calc'd for $C_{25}H_{14}N_4O_6S$ $[M+H]^+$, 525.2747; found, 525.2747.

N-Alkylated famciclovir (86)

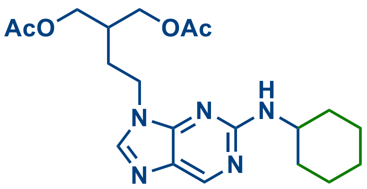

Prepared following **General Procedure C**, using famciclovir (64.9 mg, 200 μmol, 1.00 equiv., 99% purity), and 2-cyclohexyl-4,4,5,5-tetramethyl-1,3,2-dioxaborolane (84 mg, 0.400 mmol, 2.00 equiv.). Purification by CombiFlash® R_f purification system, eluting with DCM/MeOH (0 – 10%, v/v), gave **86** (68.9 mg, 85%) as a colorless oil.

NMR Spectroscopy (see spectra):

^{1}**H NMR** (400 MHz, $CDCl_3$): δ_H 8.62 (s, 1H), 7.67 (s, 1H), 5.29 – 5.13 (m, 1H), 4.26 – 4.00 (m, 6H), 3.86 – 3.74 (m, 1H), 2.01 (s, 6H), 2.00 – 1.83 (m, 5H), 1.72 (dp, *J* = 12.1, 4.1 Hz, 2H), 1.61 (dt, *J* = 12.8, 4.0 Hz, 1H), 1.45 – 1.33 (m, 2H), 1.27 – 1.14 (m, 3H) ppm.

^{13}**C NMR** (101 MHz, $CDCl_3$): δ_C 170.9, 159.2, 153.4, 149.9, 141.4, 127.3, 63.6, 50.1, 40.6, 34.8, 33.2, 28.8, 25.8, 24.9, 20.9 ppm.

HRMS (ESI) m/z calc'd for $C_{20}H_{30}N_5O_4$ $[M+H]^+$, 404.2298; found, 404.2296.

N-Alkylated ruxolitinib (87)

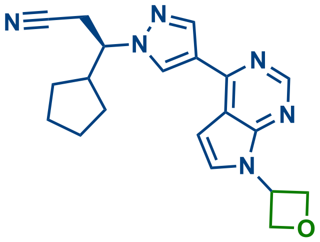

Prepared following **General Procedure C**, using ruxolitinib (62.5 mg, 200 μmol, 1.00 equiv., 98% purity), and 4,4,5,5-tetramethyl-2-(oxetan-3-yl)-1,3,2-dioxaborolane (74 mg, 0.400 mmol, 2.00 equiv.). Purification by CombiFlash® R_f purification system, eluting with DCM/MeOH (0 – 10%, v/v), gave **87** (66.8 mg, 92%) as a brown solid.

NMR Spectroscopy (see spectra):

^{1}**H NMR** (400 MHz, CD_3CN): δ_H 8.67 (s, 1H), 8.41 (s, 1H), 8.28 (s, 1H), 7.79 (d, *J* = 3.8 Hz, 1H), 6.91 (d, *J* = 3.8 Hz, 1H), 5.96 (ddd, *J* = 7.7, 6.4, 1.3 Hz, 1H), 5.09 – 4.92 (m, 4H), 4.35 (td, *J* = 9.8, 3.9 Hz, 1H), 3.10 (dd, *J* = 17.2, 9.6 Hz, 1H), 2.97 (dd, *J* = 17.1, 3.9 Hz, 1H), 2.54 – 2.39 (m, 1H), 2.13 (s, 2H), 1.90 (h, *J* = 2.5 Hz, 2H), 1.70 – 1.40 (m, 5H), 1.40 – 1.11 (m, 4H) ppm.

^{13}**C NMR** (101 MHz, CD_3CN): δ_C 152.4, 152.1, 151.7, 140.5, 131.6, 127.4, 121.8, 114.8, 101.2, 78.3, 64.5, 49.4, 45.5, 30.4, 30.3, 26.0, 25.4, 23.9 ppm.

HRMS (ESI) m/z calc'd for $C_{20}H_{23}N_6O$ $[M+H]^+$, 363.1933; found, 363.1929.

N-Alkylated dabrafenib (88)

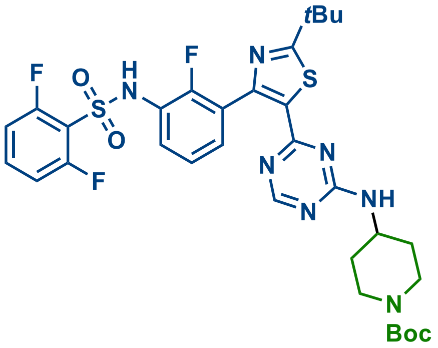

Prepared following **General Procedure C**, using dabrafenib (106 mg, 200 µmol, 1.00 equiv., 99% purity), and *tert*-butyl 4-(4,4,5,5-tetramethyl-1,3,2-dioxaborolan-2-yl)piperidine-1-carboxylate (124 mg, 0.400 mmol, 2.00 equiv.). Purification by CombiFlash® R_f purification system, eluting with DCM/MeOH (0 – 10%, v/v), gave **88** (85.1 mg, 61%) as a slightly yellow solid.

NMR Spectroscopy (*see spectra***):**

^{1}**H NMR** (400 MHz, CD_3CN): δ_H 7.97 (d, *J* = 5.2 Hz, 1H), 7.55 (tdd, *J* = 7.8, 5.1, 2.2 Hz, 2H), 7.34 (ddd, *J* = 8.1, 6.4, 1.8 Hz, 1H), 7.24 (t, *J* = 7.9 Hz, 1H), 7.03 (t, *J* = 8.9 Hz, 2H), 5.79 (d, *J* = 7.7 Hz, 1H), 3.93 (d, *J* = 13.4 Hz, 2H), 1.41 (s, *J* = 1.3 Hz, 18H), 1.39 – 1.17 (m, 4H) ppm.

^{13}**C NMR** (101 MHz, CD_3CN): δ_C 182.9, 162.3, 159.90 (dd, J = 257.7, 3.6 Hz), 158.9, 155.2, 154.3, 152.3 (d, *J* = 250.5 Hz), 136.8 (t, *J* = 11.0 Hz), 134.9, 129.9, 127.0, 125.6, 125.6, 124.9 (d, *J* = 14.4 Hz), 117.76 (t, *J* = 15.7 Hz), 114.1 (dd, *J* = 22.9, 3.7 Hz), 113.86, 107.2, 79.6, 48.9, 38.5, 32.2, 30.7, 28.4 ppm.117.73 (t, *J* = 15.7 Hz), 112.93 (dd, *J* = 22.9, 3.7 Hz), 107.11, 37.86, 30.60 ppm.

^{19}**F NMR** (376 MHz, $CDCl_3$): δ_F –106.90, –130.09 ppm.

HRMS (ESI) m/z calc'd for $C_{33}H_{38}N_6O4S_2F_3$ $[M+H]^+$, 703.2348; found, 703.2350.

N-Alkylated vemurafenib (89)

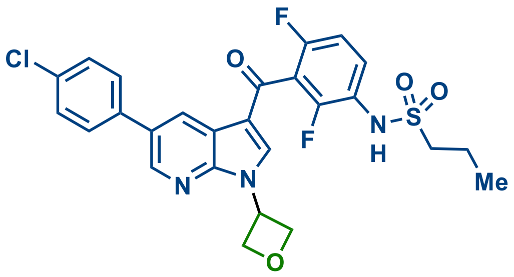

Prepared following **General Procedure B**, using vemurafenib (100 mg, 200 µmol, 1.00 equiv., 99% purity), and 4,4,5,5-tetramethyl-2-(oxetan-3-yl)-1,3,2-dioxaborolane (74 mg, 0.40 mmol, 2.0 equiv.). Purification by

CombiFlash® R_f purification system, eluting with EtOAc/heptane (0 – 60%, v/v), gave **89** (78.9 mg, 72%) as a colorless solid.

NMR Spectroscopy (see spectra):

^{1}H NMR (400 MHz, d_6-DMSO): δ_H 9.80 (s, 1H), 8.84 – 8.58 (m, 3H), 7.79 (d, *J* = 8.0 Hz, 2H), 7.67 – 7.51 (m, 3H), 7.31 (t, *J* = 8.8 Hz, 1H), 6.00 (t, *J* = 7.2 Hz, 1H), 5.10 (t, *J* = 6.8 Hz, 2H), 4.99 (t, *J* = 7.4 Hz, 2H), 3.15 (dd, *J* = 8.9, 6.5 Hz, 2H), 1.75 (q, *J* = 7.6 Hz, 2H), 0.96 (t, *J* = 7.4 Hz, 3H) ppm.

^{13}C NMR (101 MHz, d_6-DMSO): δ_C 180.7, 156.1 (d, *J* = 252.5 Hz), 155.0, 149.5 (d, *J* = 252.5 Hz), 143.8, 139.0, 136.7, 132.7, 131.0, 129.1 (d, *J* = 13.1 Hz), 129.0, 122.0 (d, *J* = 13.9 Hz), 118.2, 117.9 (d, *J* = 23.1 Hz), 115.4, 112.5 (d, *J* = 22.4 Hz), 75.9, 53.4, 49.3, 16.8, 12.6 ppm.

^{19}F NMR (376 MHz, d_6-DMSO): δ_F –117.00 (d, *J* = 8.3 Hz, 1F), –122.24 (d, *J* = 8.9 Hz, 1F) ppm.

HRMS (ESI) m/z calc'd for $C_{26}H_{23}N_3O_4SClF_2$ $[M+H]^+$, 546.1066; found, 546.1058.

***N*-Alkylated aripiprazole (90)**

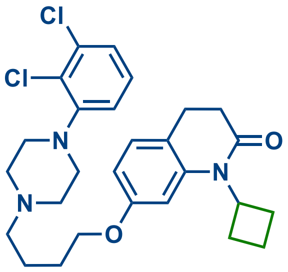

Prepared following **General Procedure C**, using aripiprazole (91.5 mg, 200 µmol, 1.00 equiv., 98% purity), and 2-cyclobutyl-4,4,5,5-tetramethyl-1,3,2-dioxaborolane (73 mg, 0.400 mmol, 2.00 equiv.). Purification by CombiFlash® R_f purification system, eluting with DCM/MeOH (0 – 10%, v/v), gave **90** (82.1 mg, 82%) as a colorless solid.

NMR Spectroscopy (see spectra):

^{1}H NMR (400 MHz, $CDCl_3$): δ_H 7.15 – 7.03 (m, 2H), 7.00 (d, *J* = 8.2 Hz, 1H), 6.94 (dd, *J* = 6.3, 3.2 Hz, 1H), 6.50 (dd, *J* = 8.1, 2.3 Hz, 1H), 6.34 (d, *J* = 2.3 Hz, 1H), 4.37 (p, *J* = 8.1 Hz, 1H), 3.95 (t, *J* = 6.2 Hz, 2H), 3.08 (brs, 4H), 2.83 – 2.70 (m, 6H), 2.62 – 2.41 (m, 6H), 2.22 (pd, *J* = 10.0, 2.6 Hz, 2H), 1.77 (dh, *J* = 28.8, 7.0 Hz, 6H) ppm.

^{13}C NMR (101 MHz, $CDCl_3$): δ_C 172.0, 158.1, 151.2, 140.3, 134.1, 127.7, 127.5, 127.5, 124.7, 120.5, 118.7, 107.6, 104.9, 67.9, 58.2, 53.3, 51.5, 51.2, 34.0, 30.3, 27.3, 24.8, 23.4, 15.6 ppm.

HRMS (ESI) m/z calc'd for $C_{27}H_{34}N_3O_2Cl_2$ $[M+H]^+$, 502.2028; found, 502.2030.

_N_-Alkylated celecoxib (91)

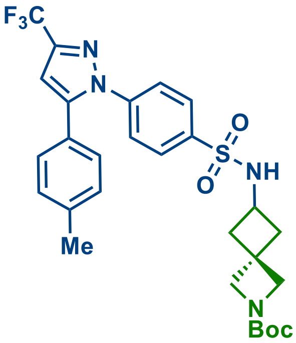

Prepared following **General Procedure C**, using celecoxib (77 mg, 0.20 mmol, 1.0 equiv., 99% purity), **S57** (129 mg, 0.400 mmol, 2.00 equiv.). Purification by CombiFlash® R_f purification system, eluting with EtOAc/heptane (0 – 40%, v/v), gave **91** (84.3 mg, 73%) as a colorless solid.

NMR Spectroscopy (_see spectra_):

^{1}**H NMR** (400 MHz, $CDCl_3$): δ_H 7.82 (d, *J* = 8.5 Hz, 2H), 7.44 (d, *J* = 8.5 Hz, 2H), 7.15 (d, *J* = 7.9 Hz, 2H), 7.08 (d, *J* = 7.9 Hz, 2H), 6.74 (brs, 1H, NH), 5.66 (brs, 1H), 3.85 (s, 2H), 3.75 (s, 2H), 3.64 (q, *J* = 8.1 Hz, 1H), 2.39 – 2.28 (m, 2H), 2.37 (s, 3H), 1.95 (td, *J* = 9.1, 3.2 Hz, 2H), 1.39 (s, 9H, Boc) ppm.

^{13}**C NMR** (101 MHz, $CDCl_3$): δ_C 156.2, 145.4, 144.2 (q, J = 38.0 Hz), 142.6, 140.21, 139.93, 129.83, 128.12, 125.8, 121.1 (q, *J* = 269.2 Hz), 106.4, 79.8, 43.3, 41.6, 32.0, 31.8, 29.1, 28.4, 22.8, 21.4, 14.2 ppm.

^{19}**F NMR** (376 MHz, $CDCl_3$): δ_F –62.38 (s) ppm.

HRMS (ESI) m/z calc'd for $C_{28}H_{32}N_4O_4F_3S$ $[M+H]^+$, 577.2096; found, 577.2106.

_N_-Alkylated pazopanib (92)

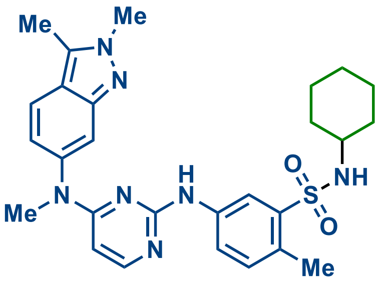

Prepared following **General Procedure C**, using pazopanib (88.4 mg, 200 μmol, 1.00 equiv., 99% purity), and cyclohexylboronic acid pinacol ester (84 mg, 0.40 mmol, 2.0 equiv.). Purification by CombiFlash® R_f purification system, eluting with DCM/MeOH (0 – 20%, v/v), gave **92** (93.1 mg, 90%) as a colorless solid.

NMR Spectroscopy (_see spectra_):

^{1}**H NMR** (400 MHz, Acetic): δ_H δ 9.00 (s, 1H), 7.84 (d, *J* = 8.7 Hz, 2H), 7.72 (d, *J* = 1.7 Hz, 1H), 7.63 (s, 1H), 7.36 (d, *J* = 8.0 Hz, 1H), 7.04 (dd, *J* = 8.8, 1.8 Hz, 1H), 5.96 (d, *J* = 6.3 Hz, 1H), 4.17 (s, 3H), 3.73 (s, 3H), 3.17 – 3.06 (brs, 1H), 2.68 (s, 3H), 2.62 (s, 3H), 1.74 (dt, *J* = 8.3, 4.5 Hz, 2H), 1.71 – 1.61 (m, 2H), 1.50 (d, *J* = 11.3 Hz, 1H), 1.34 – 1.25 (m, 2H), 1.25 – 1.12 (m, 5H) ppm.

^{13}C NMR (101 MHz, Acetic): δ_C 164.0, 153.0, 147.5, 143.7, 142.5, 140.3, 137.0, 135.5, 133.9, 133.3, 125.6, 123.7, 122.5, 121.3, 120.3, 115.1, 98.1, 53.3, 40.6, 37.5, 34.5, 30.6, 26.0, 25.6, 9.7 ppm.

HRMS (ESI) m/z calc'd for $C_{27}H_{34}N_7O_2S$ $[M+H]^+$, 520.2495; found, 520.2484.

***N*-Alkylated apixaban (93)**

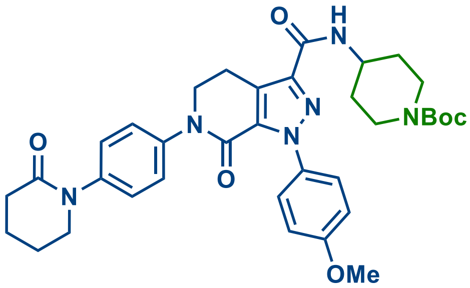

Prepared following **General Procedure C**, using apixaban (94.7 mg, 0.200 mmol, 1.00 equiv.), and *tert*-butyl 4-(4,4,5,5-tetramethyl-1,3,2-dioxaborolan-2-yl)piperidine-1-carboxylate (124 mg, 0.400 mmol, 2.00 equiv.). Purification by CombiFlash® R_f purification system, eluting with DCM/method (0 – 10%, v/v), gave **93** (118.9 mg, 93%) as a colorless solid.

NMR Spectroscopy (*see spectra*):

^{1}H NMR (400 MHz, $CDCl_3$): δ_H 7.42 (d, *J* = 8.5 Hz, 2H), 7.30 (d, *J* = 8.4 Hz, 2H), 7.21 (d, *J* = 8.3 Hz, 2H), 6.90 (dd, *J* = 8.5, 5.0 Hz, 3H), 4.07 (p, *J* = 11.0 Hz, 5H), 3.77 (s, 3H), 3.55 (d, *J* = 5.6 Hz, 2H), 3.33 (t, *J* = 6.6 Hz, 2H), 2.86 (t, *J* = 12.7 Hz, 2H), 2.50 (d, *J* = 5.5 Hz, 2H), 1.90 (s, 6H), 1.43 (s, 9H) ppm.

^{13}C NMR (101 MHz, $CDCl_3$): δ_C 170.1, 161.1, 159.8, 157.3, 154.7, 141.3, 141.1, 139.9, 133.3, 132.4, 126.8, 126.7, 126.1, 125.4, 113.7, 79.6, 58.5, 51.5, 51.1, 46.4, 42.7, 32.8, 32.0, 28.4, 23.4, 21.3, 21.2 ppm.

HRMS (ESI) m/z calc'd for $C_{35}H_{43}N_6O_6$ $[M+H]^+$, 643.3244; found, 643.3243.

***N*-Alkylated axitinib (94)**

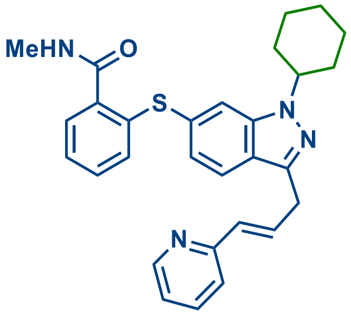

Prepared following **General Procedure C**, using axitinib (77.3 mg, 0.200 mmol, 1.00 equiv.), and cyclohexylboronic acid pinacol ester (84 mg, 0.40 mmol, 2.0 equiv.). Purification by CombiFlash® R_f purification system, eluting with DCM/method (0 – 20%, v/v), gave **94** (76.4 mg, 82%) as a brown solid.

NMR Spectroscopy (*see spectra*):

^{1}H NMR (400 MHz, $CDCl_3$): δ_H 8.54 (d, *J* = 4.9 Hz, 1H), 8.03 – 7.80 (m, 2H), 7.69 – 7.43 (m, 5H), 7.23 –

7.02 (m, 5H), 6.64 (q, J = 4.9 Hz, 1H), 4.32 (p, J = 8.2 Hz, 1H), 2.93 (d, J = 4.8 Hz, 3H), 2.10 – 1.84 (m, 6H), 1.73 (d, J = 12.6 Hz, 1H), 1.51 – 1.23 (m, 3H) ppm.

^{13}C NMR (101 MHz, $CDCl_3$): δ_C 168.7, 155.5, 149.0, 141.2, 140.5, 137.1, 136.2, 135.7, 132.0, 130.7, 130.5, 128.8, 128.3, 126.3, 126.0, 124.5, 122.2, 122.0, 122.0, 121.7, 114.3, 58.5, 32.5, 26.8, 25.7, 25.3 ppm.

All recorded spectroscopic data matched those previously reported in the literature.[1]

One-pot synthesis of *N*-alkylated celecoxib (95)

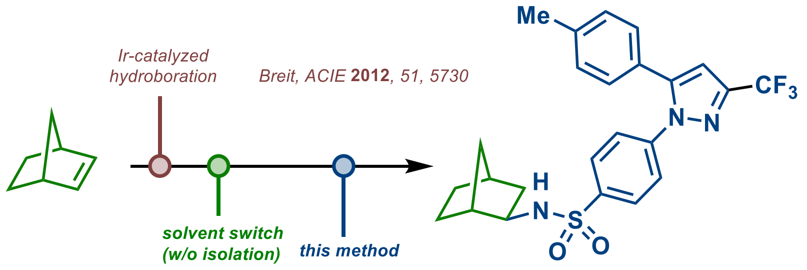

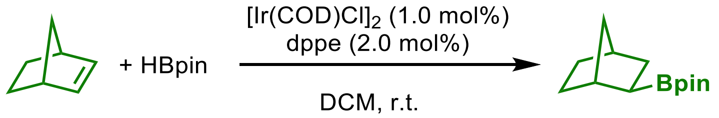

To a solution of $[Ir(COD)Cl]_2$ (4.0 mg, 6.0 μmol, 1.0 mol%), and 1,2-bis(diphenylphosphino)ethane (4.8 mg, 12 μmol, 2.0 mol%) in DCM (3.0 mL) was added norbornene (56.5 mg, 0.600 mmol, 1.00 equiv.) followed by dropwise addition of pinacolborane (92.1 mg, 0.720 mmol, 0.104 ml, 1.2 equiv.) at ambient temperature. After stirring for 24 h at room temparutre., the reaction mixture was quenched with methanol (2.0 mL) and the solvents were removed *in vacuo.* The residue compund **S95** was used directly withour purification for the next step.

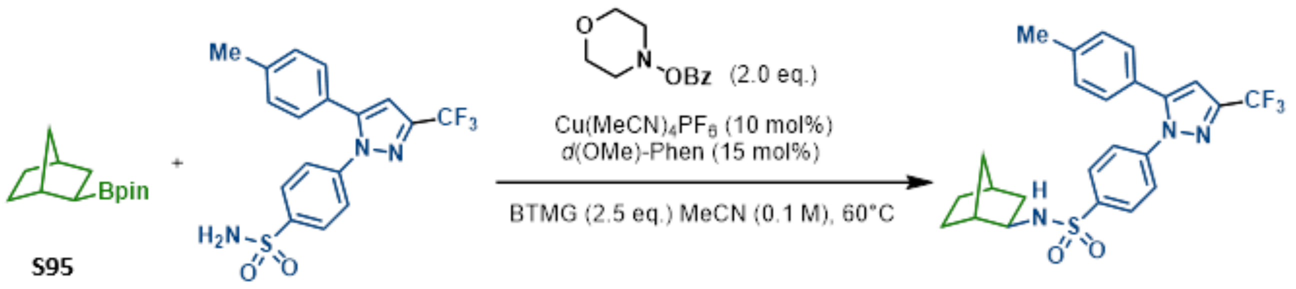

Under an ambient atmosphere, a flame dried 6 mL reaction vial equipped with a magnetic stir bar was charged with $[Cu(MeCN)_4]PF_6$ (7.5 mg, 20 μmol, 10 mol%), 4,7-dimethoxy-1,10-phenanthroline (7.2 mg, 30 μmol, 15 mol%), celecoxib (77 mg, 0.20 mol, 1.0 equiv., 99% purity),and morpholino benzoate **1** (83 mg, 0.40 mmol, 2.0 equiv.). The vial was transferred into an anhydrous, N_2-filled glovebox where anhydrous MeCN was added (2.0 – 4.0 mL, c = 0.050 – 0.10 M) followed by 2-*tert*-butyl-1,1,3,3-tetramethylguanidine (BTMG) (86 mg, 0.10 mL, 0.50 mmol, 2.5 equiv.) and crude boronic ester **S95**. The vial was sealed with a cap with septum, removed from the glovebox. After stirring for 16 hours at 60°C, and the reaction mixture was diluted with EtOAc (2 × 3 mL), filtered through a pad of Celite®, and concentrated *in vacuo.* The residue was adsorbed on Celite® and purified by CombiFlash® R_f purification system using RediSep R_f Gold® silica gel (40 μm), eluting with EtOAc/heptane (0 – 40%, v/v), gave **95** (73.9 mg, 78%) as a colorless solid.

NMR Spectroscopy (see spectra):

^{1}**H NMR** (400 MHz, $CDCl_3$): δ_H 7.89 – 7.82 (m, 2H), 7.51 – 7.43 (m, 2H), 7.16 (d, *J* = 7.9 Hz, 2H), 7.09 (d, *J* = 8.2 Hz, 2H), 6.75 (s, 1H), 4.61 (d, *J* = 7.4 Hz, 1H), 3.14 (td, *J* = 7.8, 3.3 Hz, 1H), 2.37 (s, 3H), 2.21 (t, *J* = 3.8 Hz, 1H), 2.07 (d, *J* = 4.0 Hz, 1H), 1.65 – 1.52 (m, 1H), 1.50 – 1.34 (m, 2H), 1.28 (ddd, *J* = 10.8, 5.4, 3.4 Hz, 2H), 1.19 – 1.08 (m, 2H), 1.08 – 0.97 (m, 2H), 0.88 (t, *J* = 6.8 Hz, 1H) ppm.

^{13}**C NMR** (101 MHz, $CDCl_3$): δ_C 145.4, 144.2 (q, *J* = 38.0 Hz), 143.6, 142.5, 140.6, 139.9, 129.8, 128.9, 128.2, 121.6 (q, *J* = 269.0 Hz), 106.3, 56.9, 42.6, 41.0, 35.7, 35.3, 28.1, 26.4, 21.5 ppm.

^{19}**F NMR** (376 MHz, $CDCl_3$): δ_F –62.40 (s) ppm.

HRMS (ESI) m/z calc'd for $C_{24}H_{25}N_3O_2SF_3$ $[M+H]^+$, 476.1620; found, 476.1622.

One-pot synthesis of *N*-alkylated Boc-Trp-OMe (96)

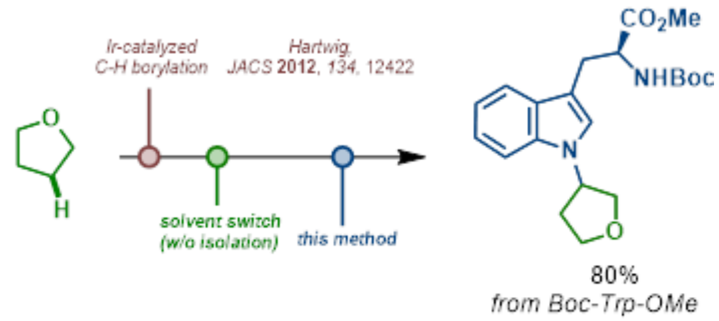

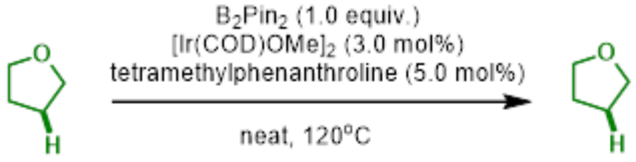

S96

In a N_2-filled glove box, B_2pin_2 (25.4 mg, 0.100 mmol), $[Ir(COD)OMe]_2$ (4.3 mg, 18 μmol, 3.0 mol %), tetramethylphenanthroline (20 mg, 30 μmol, 5.0 mol%) and THF (0.5 mL) were combined in a 4-mL vial with a stirbar. The reaction was heated to 120°C for 16 h. and the solvent was removed *in vacuo.* The residue compund **S96** was used directly without purification for the next step.

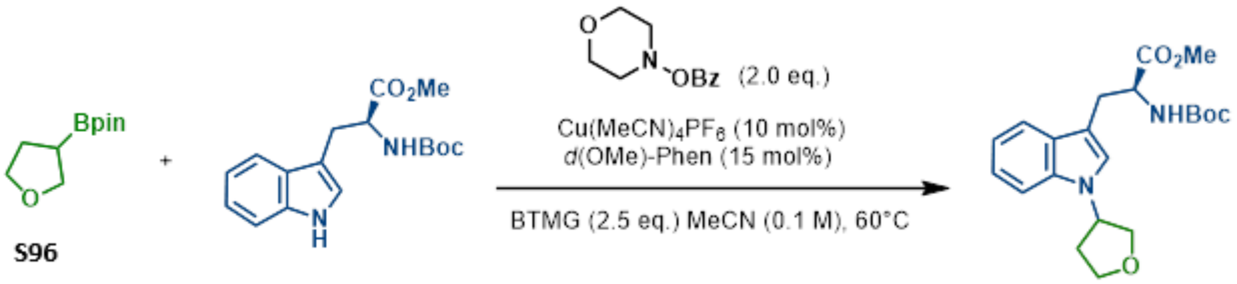

Under an ambient atmosphere, a flame dried 6 mL reaction vial equipped with a magnetic stir bar was charged with $[Cu(MeCN)_4]PF_6$ (7.5 mg, 20 μmol, 10 mol%), 4,7-dimethoxy-1,10-phenanthroline (7.2 mg, 30 μmol, 15 mol%), Boc-Trp-OMe (66 mg, 0.20 mmol, 1.0 equiv., 97% purity),and morpholino benzoate **1** (83 mg, 0.40 mmol,

2.0 equiv.). The vial was transferred into an anhydrous, N_2-filled glovebox where anhydrous MeCN was added (2.0 – 4.0 mL, *c* = 0.050 – 0.10 M) followed by 2-*tert*-butyl-1,1,3,3-tetramethylguanidine (BTMG) (86 mg, 0.10 mL, 0.50 mmol, 2.5 equiv.) and crude boronic ester **S96**. The vial was sealed with a cap with septum, removed from the glovebox. After stirring for 16 hours at 60°C, and the reaction mixture was diluted with EtOAc (2 × 3 mL), filtered through a pad of Celite®, and concentrated *in vacuo.* The residue was adsorbed on Celite® and purified by CombiFlash® R_f purification system using RediSep R_f Gold® silica gel (40 μm), eluting with EtOAc/heptane (0 – 40%, v/v), gave **96** (77.7 mg, 80%) as a colorless solid.

NMR Spectroscopy (see Spectre):

^{1}H NMR (400 MHz, $CDCl_3$): δ_H 7.55 (d, *J* = 7.9 Hz, 1H), 7.37 (d, *J* = 8.2 Hz, 1H), 7.22 (t, *J* = 7.6 Hz, 1H), 7.13 (t, *J* = 7.5 Hz, 1H), 7.05 (s, 1H), 5.13 – 5.00 (m, 2H), 4.64 (dt, *J* = 9.0, 5.4 Hz, 1H), 4.19 – 4.00 (m, 3H), 3.94 (td, *J* = 8.6, 5.9 Hz, 1H), 3.70 (s, 3H), 3.36 – 3.20 (m, 2H), 2.48 (tq, *J* = 14.6, 6.9 Hz, 1H), 2.15 (dtdd, *J* = 17.9, 11.8, 6.5, 3.2 Hz, 1H), 1.45 (s, 9H) ppm.

^{13}C NMR (101 MHz, $CDCl_3$): δ_C 172.7, 155.2, 136.1, 136.1, 128.5, 128.5, 123.5, 121.9, 119.5, 119.2, 109.9, 109.8, 109.4, 79.8, 72.8, 72.6, 67.6, 67.5, 55.6, 54.4, 52.3, 52.2, 33.4, 33.4, 29.8, 28.4, 28.1, 28.0 ppm.

HRMS (ESI) m/z calc'd for $C_{21}H_{28}N_2O_5Na$ $[M+Na]^+$, 411.1896; found, 411.1895.

Large-scale synthesis of 5

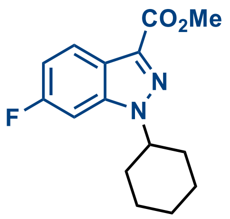

Under an ambient atmosphere, a flame dried 100 mL round-bottom flask equipped with a magnetic stir bar was charged with $[Cu(MeCN)_4]PF_6$ (112 mg, 0.300 mmol, 10.0 mol%), 4,7-dimethoxy-1,10-phenanthroline (108 mg, 0.450 mmol, 20.0 mol%), **4** (0.61 g, 3.0 mmol, 1.0 equiv., 96% purity),and morpholino benzoate **1** (1.24 g, 6.00 mmol, 2.0 equiv.). The vial was transferred into an anhydrous, N_2-filled glovebox where anhydrous MeCN was added (60 mL, *c* = 0.050 M) followed by 2-*tert*-butyl-1,1,3,3-tetramethylguanidine (BTMG) (1.24 g, 13.7 mmol, 2.80 mL, 2.5 equiv.) and 4,4,5,5-tetramethyl-2-(oxetan-3-yl)-1,3,2-dioxaborolane (1.28 g, 1.5 mL, 7.50 mmol, 2.50 equiv.). The flask was sealed with a septum, removed from the glovebox. After stirring the reaction mixture at 60°C for 24 hours, EtOAc (80 mL) was added, and the mixture was filtered through a pad of Celite®. Sat. NH_4Cl solution (60 mL) was then added, and the layers were separated. The organic phase was washed sequentially with 0.5 M HCl (2 × 60 mL), water (60 mL), and brine (60 mL). The organic phase was dried with $MgSO_4$, filtered, and concentrated *in vacuo.* The residue was adsorbed on Celite® and purified by CombiFlash® R_f purification system using RediSep R_f Gold® silica gel (120 μm), eluting with EtOAc/heptane (0 – 70%, v/v), gave **5** (774 mg, 93%) as a colorless oil.

Large-scale synthesis of *N*-alkylated metaxalone 97

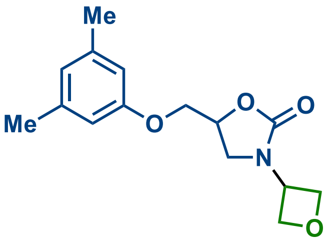

Under an ambient atmosphere, a flame dried 100 mL round-bottom flask equipped with a magnetic stir bar was charged with $[Cu(MeCN)_4]PF_6$ (0.41 g, 1.1 mmol, 20 mol%), 4,7-dimethoxy-1,10-phenanthroline (396 mg, 1.65 mmol, 25 mol%), metaxalone (1.23 g, 5.5 mmol, 1.0 equiv., 99.3% purity),and morpholino benzoate **1** (2.28 g, 11.0 mmol, 2.0 equiv.). The vial was transferred into an anhydrous, N_2-filled glovebox where anhydrous MeCN was added (55 mL, *c* = 0.10 M) followed by 2-*tert*-butyl-1,1,3,3-tetramethylguanidine (BTMG) (2.36 g, 13.7 mmol, 2.80 mL, 2.5 equiv.) and 4,4,5,5-tetramethyl-2-(oxetan-3-yl)-1,3,2-dioxaborolane (2.53 g, 13.7 mmol, 2.5 equiv.). The flask was sealed with a septum, removed from the glovebox. After stirring the reaction mixture at 60°C for 24 hours, EtOAc (80 mL) was added, and the mixture was filtered through a pad of Celite®. Sat. NH_4Cl solution (80 mL) was then added, and the layers were separated. The organic phase was washed sequentially with 0.5 M HCl (2 × 80 mL), water (80 mL), and brine (80 mL). The organic phase was dried with $MgSO_4$, filtered, and concentrated *in vacuo.* The residue was adsorbed on Celite® and purified by CombiFlash® R_f purification system using RediSep R_f Gold® silica gel (120 μm), eluting with EtOAc/heptane (0 – 70%, v/v), gave **97** (1.37 g, 89%) as a colorless solid.

NMR Spectroscopy (*see spectra*):

^{1}**H NMR** (400 MHz, $CDCl_3$): δ_H 6.61 (s, 1H), 6.49 (s, 2H), 5.07 (p, *J* = 6.9 Hz, 1H), 4.87 – 4.63 (m, *J* = 17.9, 5.4 Hz, 5H), 4.09 (h, *J* = 5.7 Hz, 2H), 3.90 (t, *J* = 8.8 Hz, 1H), 3.77 (t, *J* = 7.3 Hz, 1H), 2.26 (s, 6H) ppm.

^{13}**C NMR** (101 MHz, $CDCl_3$): δ_C 157.9, 156.4, 139.2, 123.2, 112.1, 74.4, 74.3, 71.3, 67.7, 48.1, 42.7, 24.6, 21.2 ppm.

HRMS (ESI) m/z calc'd for $C_{15}H_{20}NO_4$ $[M+H]^+$, 278.1392; found, 278.1394.

Large-scale synthesis of *N*-alkylated fludioxonil 98

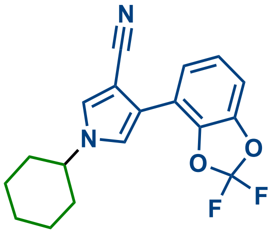

Under an ambient atmosphere, a flame dried 250 mL round-bottom flask equipped with a magnetic stir bar was charged with $[Cu(MeCN)_4]PF_6$ (0.89 g, 2.4 mmol, 20 mol%), 4,7-dimethoxy-1,10-phenanthroline (865 mg, 3.60 mmol, 25 mol%), fludioxonil (3.07 g, 12.0 mmol, 1.00 equiv., 97% purity),and morpholino benzoate **1** (4.97 g, 24.0 mmol, 2.00 equiv.). The vial was transferred into an anhydrous, N_2-filled glovebox where anhydrous MeCN

was added (120 mL, *c* = 0.10 M) followed by 2-*tert*-butyl-1,1,3,3-tetramethylguanidine (BTMG) (5.14 g, 30.0 mmol, 6.00 mL, 2.5 equiv.) and 2-cyclohexyl-4,4,5,5-tetramethyl-1,3,2-dioxaborolane (6.3 g, 30 mmol, 2.5 equiv.). The flask was sealed with a septum, removed from the glovebox. After stirring the reaction mixture at 60°C for 40 hours, EtOAc (150 mL) was added, and the mixture was filtered through a pad of Celite®. Sat. NH_4Cl solution (150 mL) was then added, and the layers were separated. The organic phase was washed sequentially with 0.5 M HCl (2 × 150 mL), water (150 mL), and brine (150 mL). The organic phase was dried with $MgSO_4$, filtered, and concentrated *in vacuo.* The residue was adsorbed on Celite® and purified by CombiFlash® R_f purification system using RediSep R_f Gold® silica gel (120 μm), eluting with EtOAc/heptane (0 – 30%, v/v), gave **98** (2.96 g, 75%) as a colorless solid and unreacted fludioxonil (0.66 g, 22%) was collected. The BRSM (based on recovered starting material) yield for compound **98** was 96%.

NMR Spectroscopy (<u>*see spectra*</u>):

^{1}H NMR (400 MHz, $CDCl_3$): δ_H 7.70 (dd, *J* = 8.3, 1.1 Hz, 1H), 7.30 (d, *J* = 2.4 Hz, 1H), 7.21 (d, *J* = 2.4 Hz, 1H), 7.11 (t, *J* = 8.1 Hz, 1H), 6.94 (dd, *J* = 8.0, 1.1 Hz, 1H), 3.95 – 3.82 (m, 1H), 2.22 – 2.04 (m, 2H), 1.91 (dt, *J* = 13.5, 3.3 Hz, 2H), 1.83 – 1.69 (m, 1H), 1.63 (qd, *J* = 12.4, 3.5 Hz, 2H), 1.41 (qt, *J* = 13.2, 3.5 Hz, 2H), 1.31 – 1.21 (m, 1H) ppm.

^{13}C NMR (101 MHz, $CDCl_3$): δ_C 143.8, 139.7, 131.4 (t, *J* = 254.6 Hz), 127.7, 124.1, 121.2, 120.8, 118.0, 116.8, 107.6, 90.5, 60.0 ppm.

^{19}F NMR (376 MHz, $CDCl_3$): δ_F –49.39 (s) ppm.

HRMS (ESI) m/z calc'd for $C_{18}H_{17}N_2O_2F_2$ $[M+H]^+$, 331.1258; found, 331.1259.

2.5. Unsuccessful Substrates

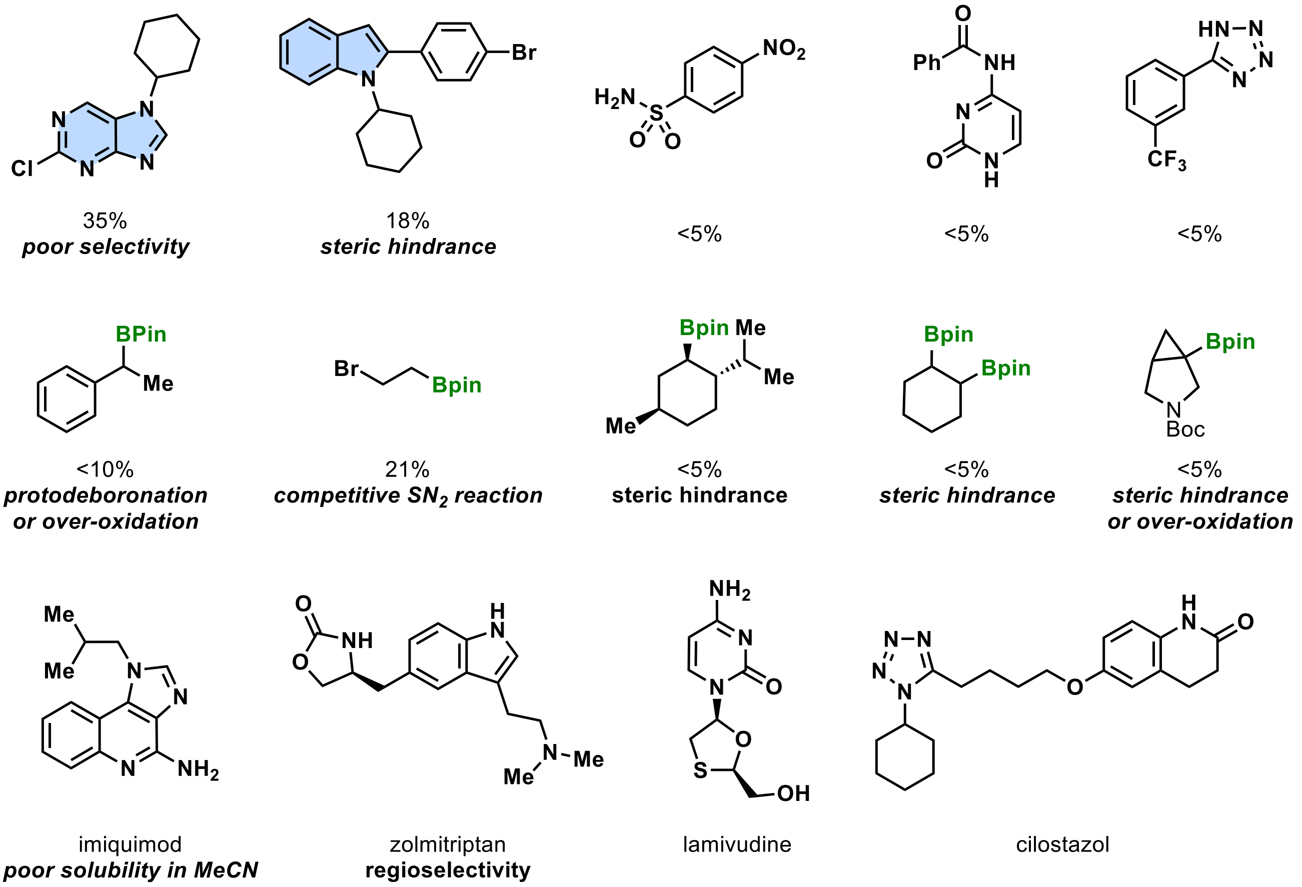

Figure S3: Unsuccessful substrates

2.6. Comparison with Previously Reported *N*-alkylation Methods

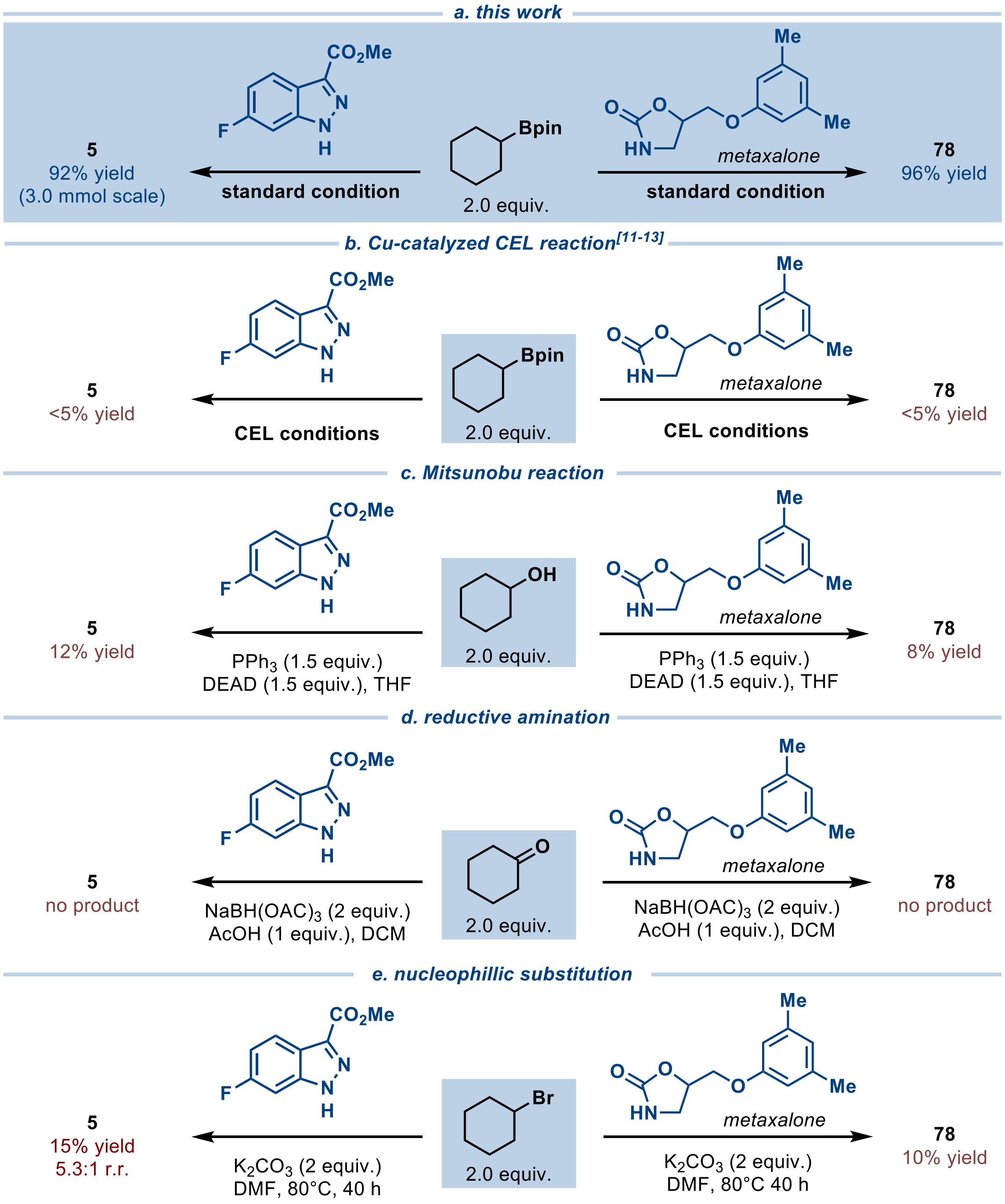

Figure S4: Comparison with previously reported N-alkylation methods;

Note: see CEL reaction conditions in reference 11-13.

2.7. Literature Analysis of *N*-heteroaromatic–organoboron Coupling Reactions

The frequency of reactions between *N*-heteroaromatics and organoborons were determined by analysis using SciFinder and duplicates were removed.

couplings between sp^2 organoborons and N-heteroaromatics

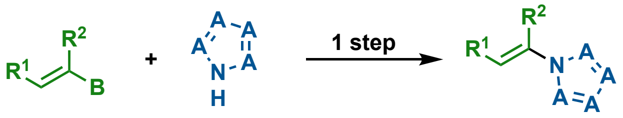

residues	hits
sp^2	*8508*
sp^3	*865*
total	*9373*

couplings between alkyl organoborons and N-heteroaromatics

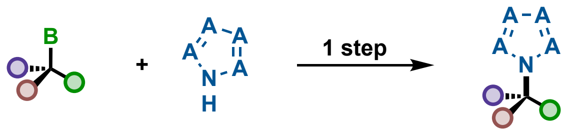

A = any atom except H

alkyl residues	hits
methyl	*4*
primary	*35*
cyclopropyl	*795*
other secondary	*15*
tertiary	*16*
total	*865*

Figure S5: Literature analysis of *N*-heteroaromatic–organoboron coupling reactions

3. MECHANISTIC STUDIES

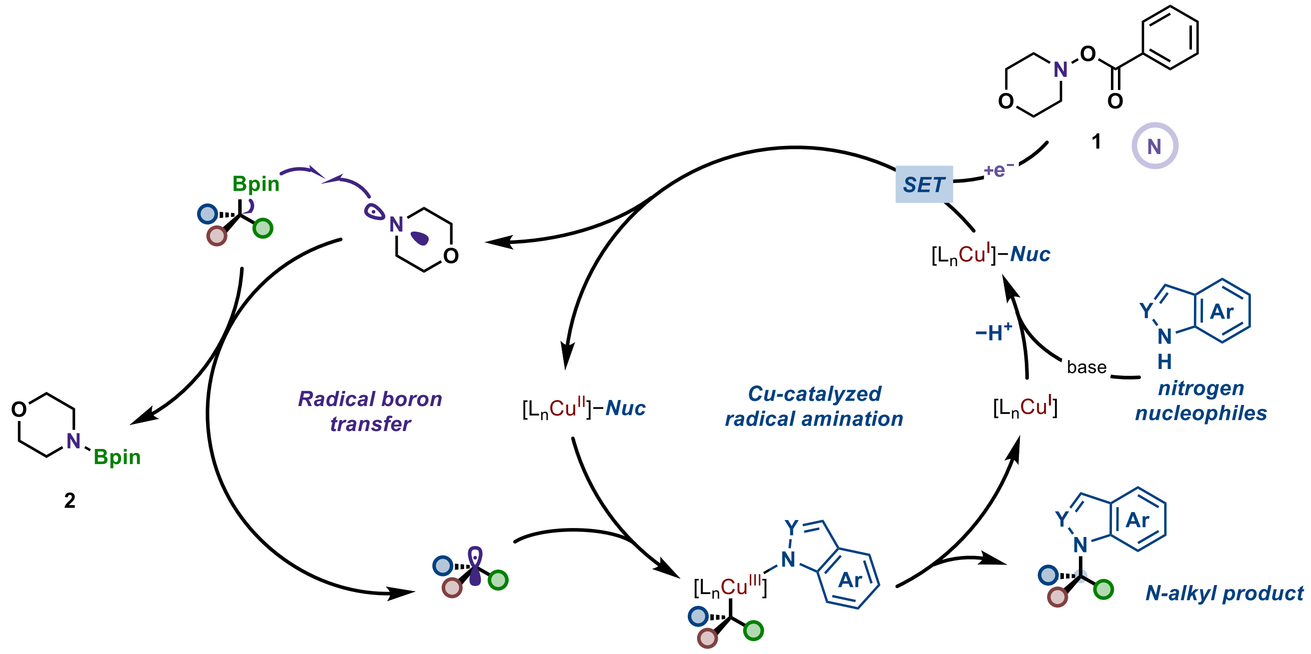

Figure S6: Proposed mechanism

3.1. [L_n–Cu(I)–amido] Formation by the Coordination of S6 to [L_n–Cu(I)]

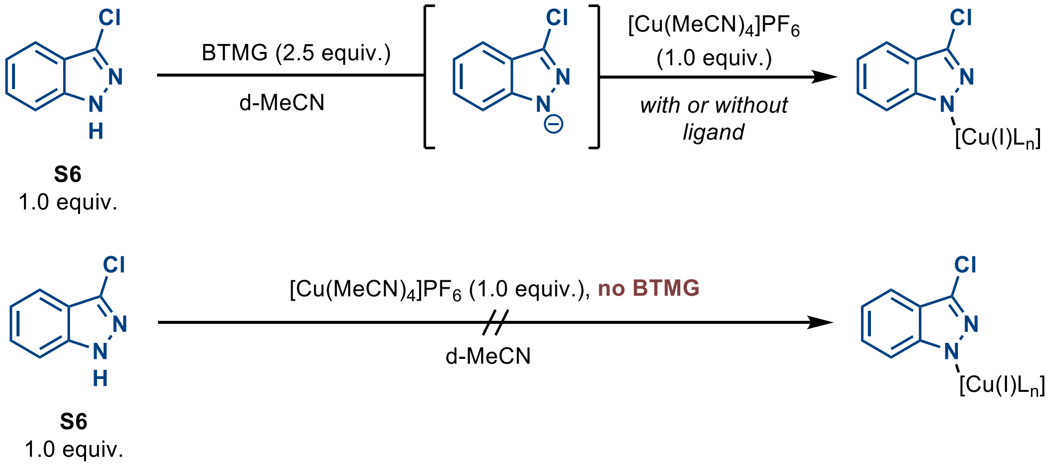

Figure S7: Studies towards the formation of [L_n–Cu(I)–amido] from indazole S6

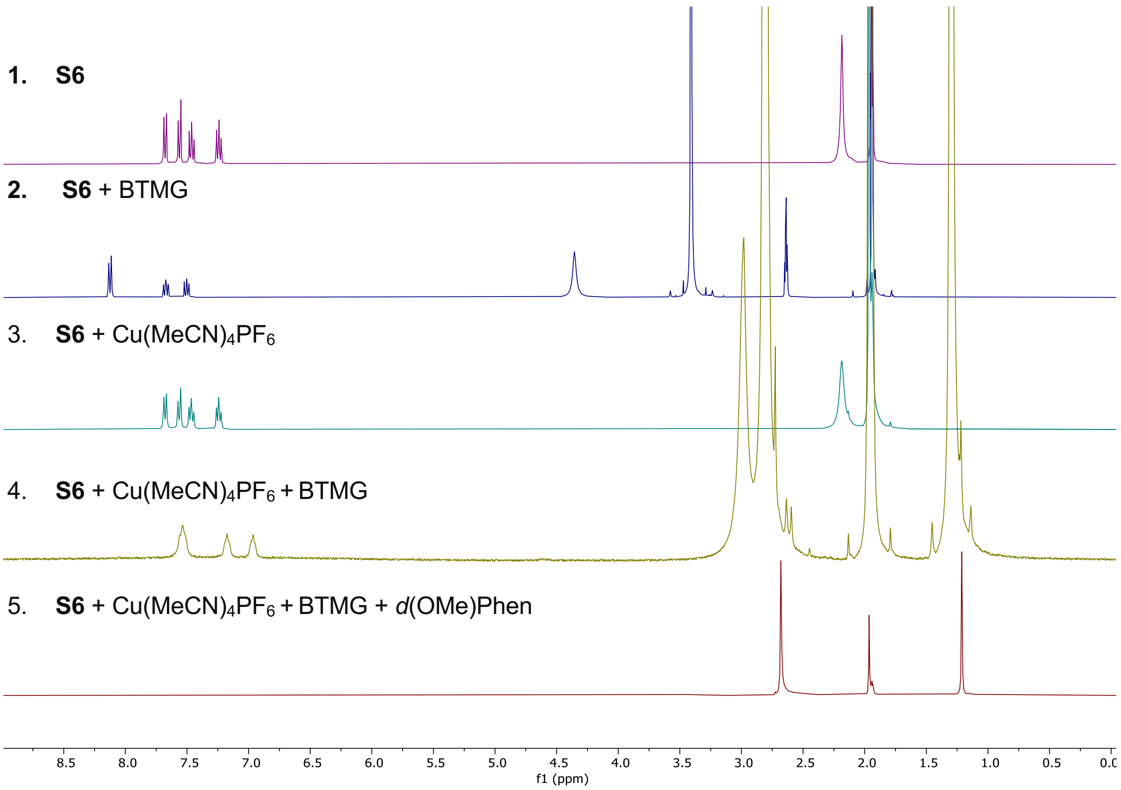

Figure S8: ^{1}H NMR spectroscopy studies towards the formation of [L_n–Cu(I)–amido] from indazole S6

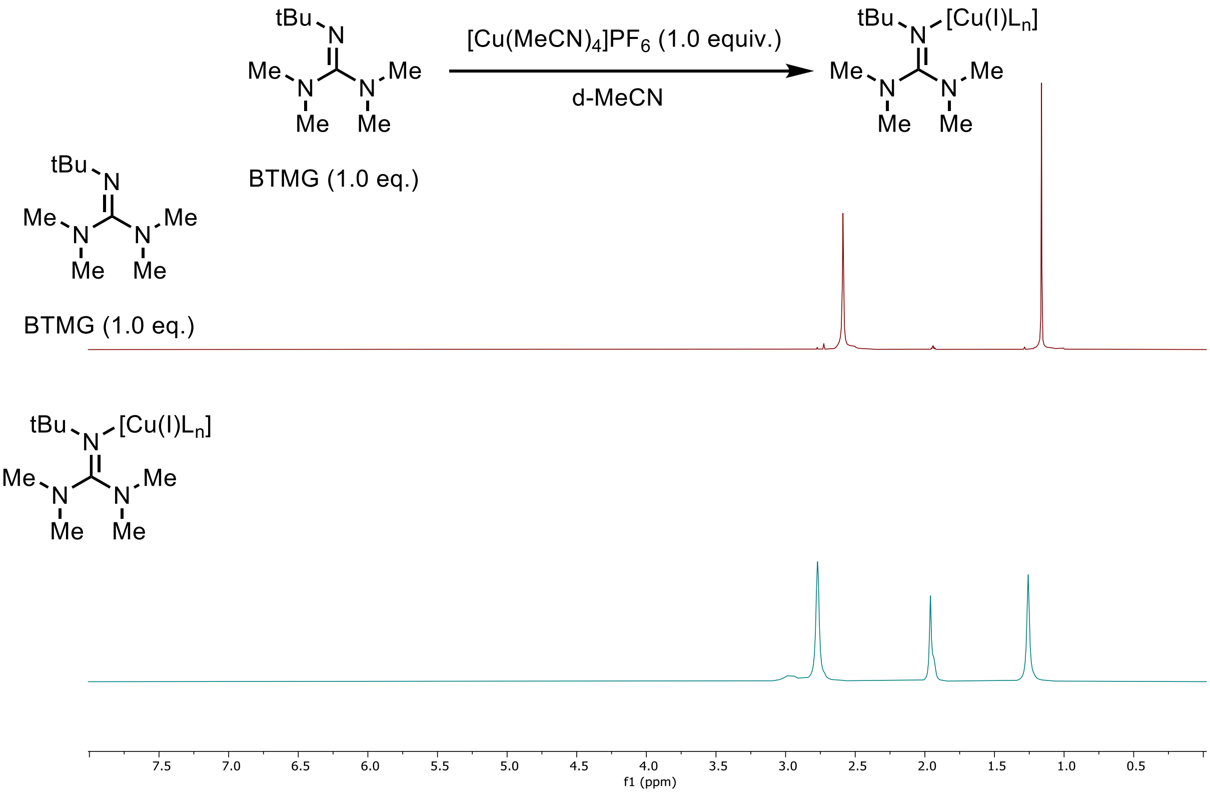

Figure S9: ^{1}H NMR spectroscopy studies towards the coordination of $Cu(MeCN)_4PF_6$ to BTMG

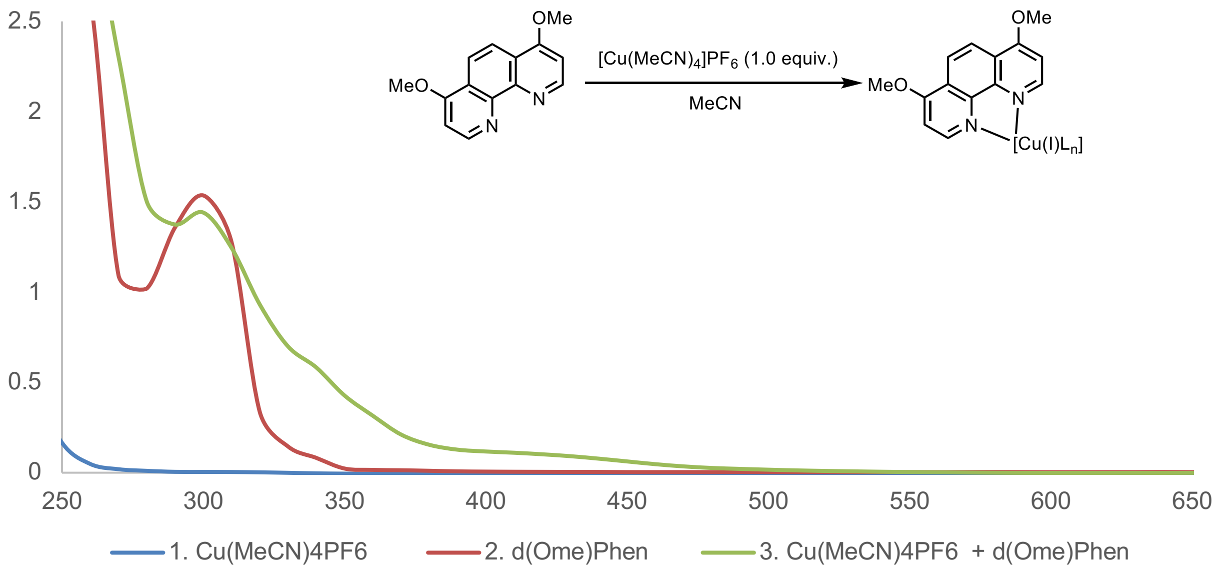

Figure S10: UV/Vis absorption spectroscopy studies towards the coordination of $Cu(MeCN)_4PF_6$ to the ligand, *d*(OMe)Phen

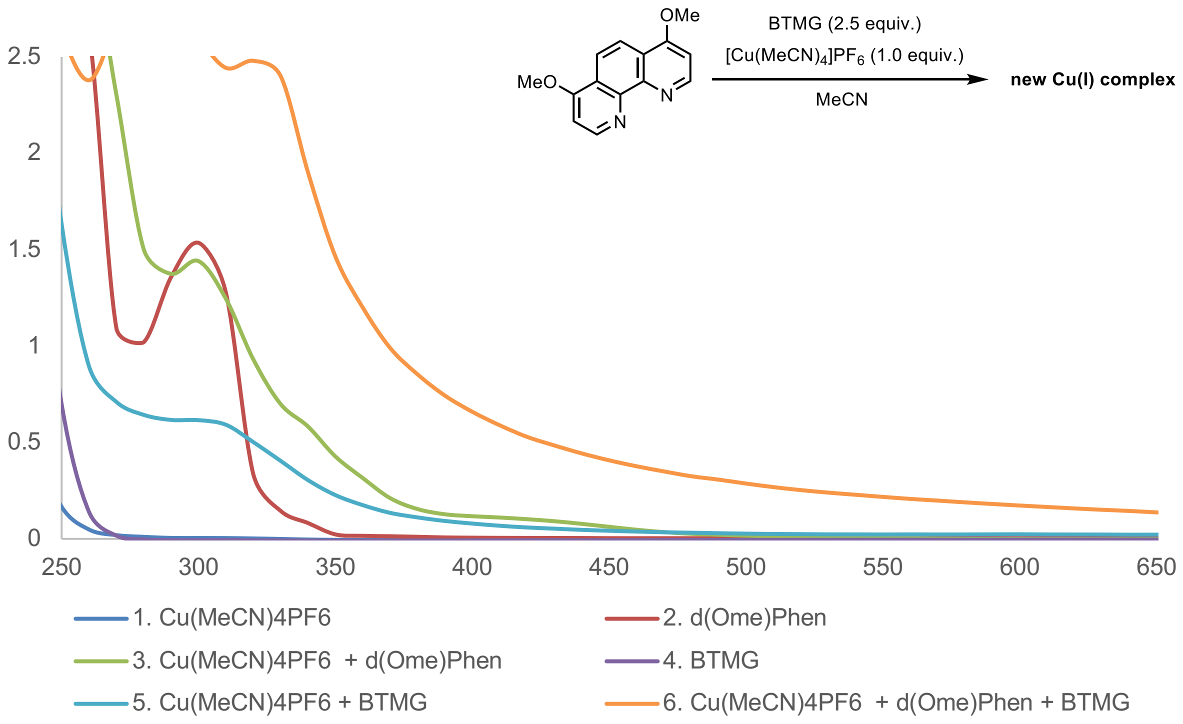

Figure S11: UV/Vis absorption spectroscopy studies towards the coordination of $Cu(MeCN)_4PF_6$ to the ligand *d*(OMe)Phen, and BTMG

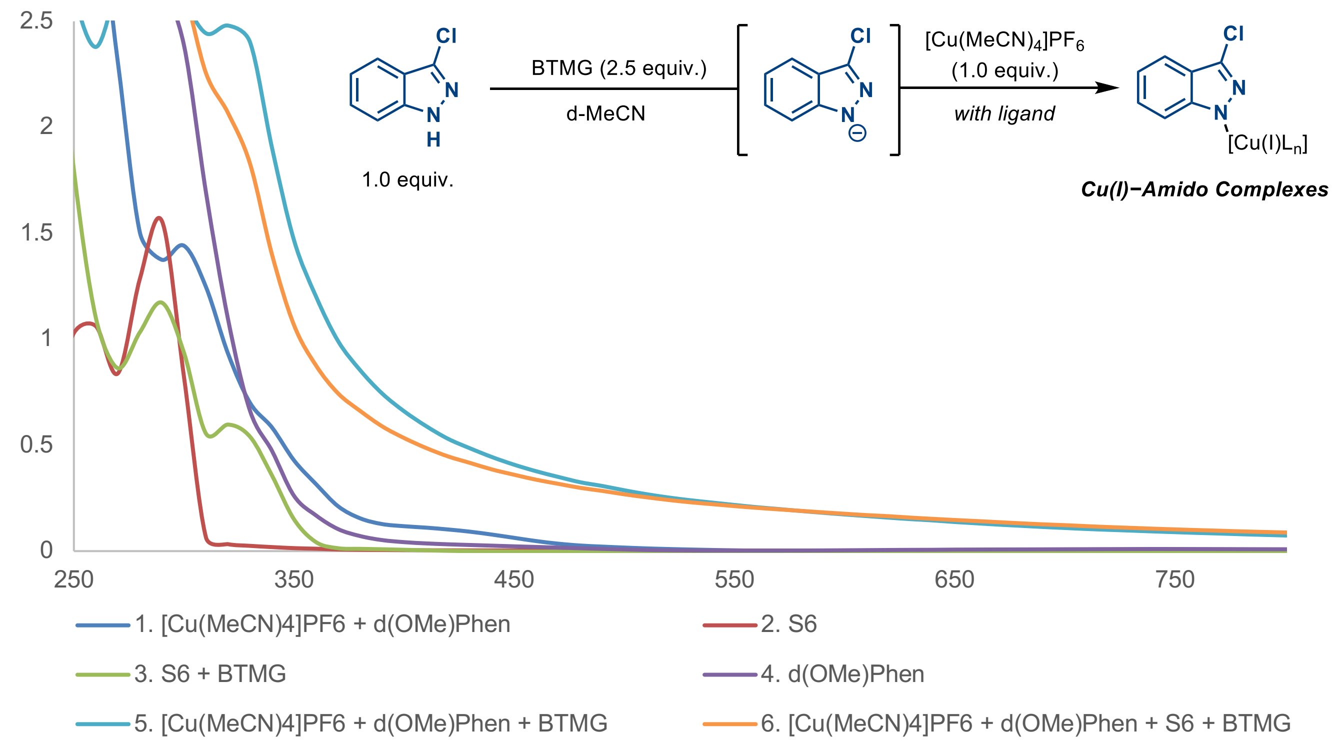

Figure S12: UV/Vis absorption spectroscopy studies towards the formation of [L_n–Cu(I)–amido] from indazole S6 with ligand *d*(OMe)Phen, and BTMG

In a typical procedure, 3-chloro-1*H*-indazole **S6** (7.86 mg, 50.0 µmol, 1.00 equiv.), [Cu(MeCN)4]PF6 (18.6 mg, 50.0 µmol, 1.00 equiv.), *d*(OMe)Phen (12.0 mg, 50.0 µmol, 1.00 equiv.), and BTMG (21.4 mg, 125 µmol, 2.50 equiv.) were dissolved in MeCN (5 mL, *c* = 10 mM of **S6**). Subsequently, 200 µL of this solution was diluted with MeCN to a final volume of 5 mL (*c* = 0.4 mM of **S6**). The absorbance of the resulting 0.4 mM solution was measured using a UV-Vis spectrometer in a cuvette (l = 1.0 cm).

In control experiment (Table S9), it shows the BTMG base is required to give *N*-alkyl product. According to the ^{1}H NMR studies, indazole **S6** is not able to undergo complexation to $[Cu(MeCN)_4]PF_6$ unless BTMG is present (Figure S8). As BTMG leads to deprotonation of **S6**, we believe **S6** in the neutral form is not reactive enough for coordination to [L_n–Cu(I)] to generate [L_n–Cu(I)–amido] complex, hence the need for a base. As a ligand, *d*(OMe)Phen is also able to coordinate [L_n–Cu(I)], but the ^{1}H NMR of this formed complex is different to the one obtained in the presence of **S6**, presumably due to the poor solubility in MeCN.

Our UV/Vis and ^{1}H NMR studies (Figure S9–S11) reveal, as ligands, both *d*(OMe)Phen and BTMG are able to coordinate $[Cu(MeCN)_4]PF_6$, UV/Vis profile of this species is different to the ones with single coordinate with *d*(OMe)Phen or BTMG. As a result, this species can further coordinate with deprotonated indazole **S6** in presence of excess BTMG base to give *d*(OMe)Phen-and-BTMG-ligated [L_n–Cu(I)–amido] complex (Figure S12).

3.2 Single-electron-oxidation of [L_n–Cu(I)–amido] to [L_n–Cu(I)–amido] by Reagent 1

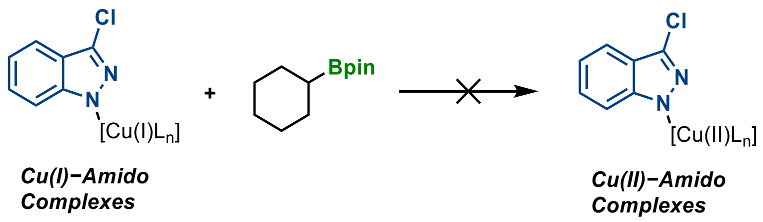

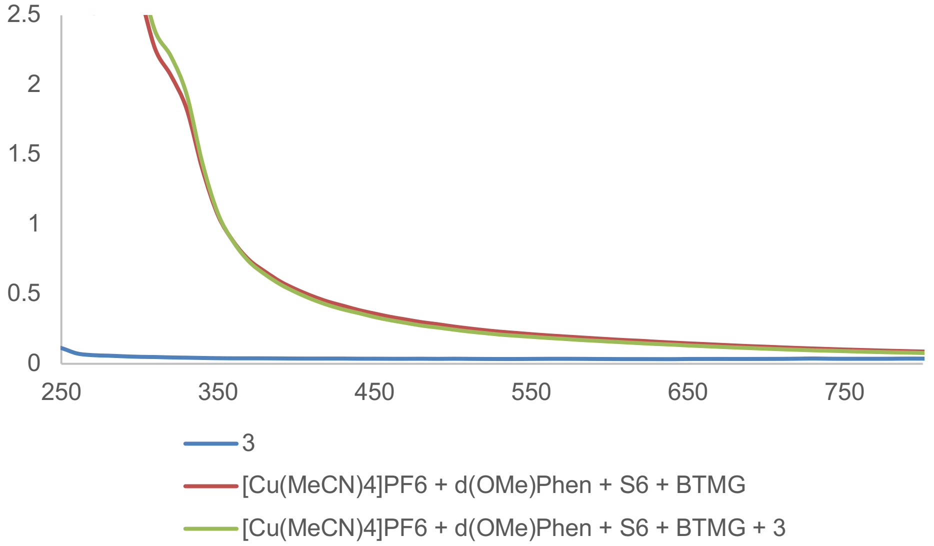

Figure S13: UV/Vis absorption spectroscopy studies towards the interaction between [L_n–Cu(I)–amido] and cyclohexylboronic acid pinacol ester 3

In a typical procedure, 3-chloro-1*H*-indazole **S6** (7.86 mg, 50.0 µmol, 1.00 equiv.), [Cu(MeCN)4]PF6 (18.6 mg, 50.0 µmol, 1.00 equiv.), *d*(OMe)Phen (12.0 mg, 50.0 µmol, 1.00 equiv.), cyclohexylboronic acid pinacol ester **3** (21 mg, 0.10 mmol, 2.0 equiv.), and BTMG (21.4 mg, 125 µmol, 2.50 equiv.) were dissolved in MeCN (5 mL, *c* = 10 mM of **S6**). After stirring at 60°C for 6 h, 200 µL of this solution was diluted with MeCN to a final volume of 5 mL (*c* = 0.4 mM of **S6**). The absorbance of the resulting 0.4 mM solution was measured using a UV-Vis spectrometer in a cuvette (l = 1.0 cm).

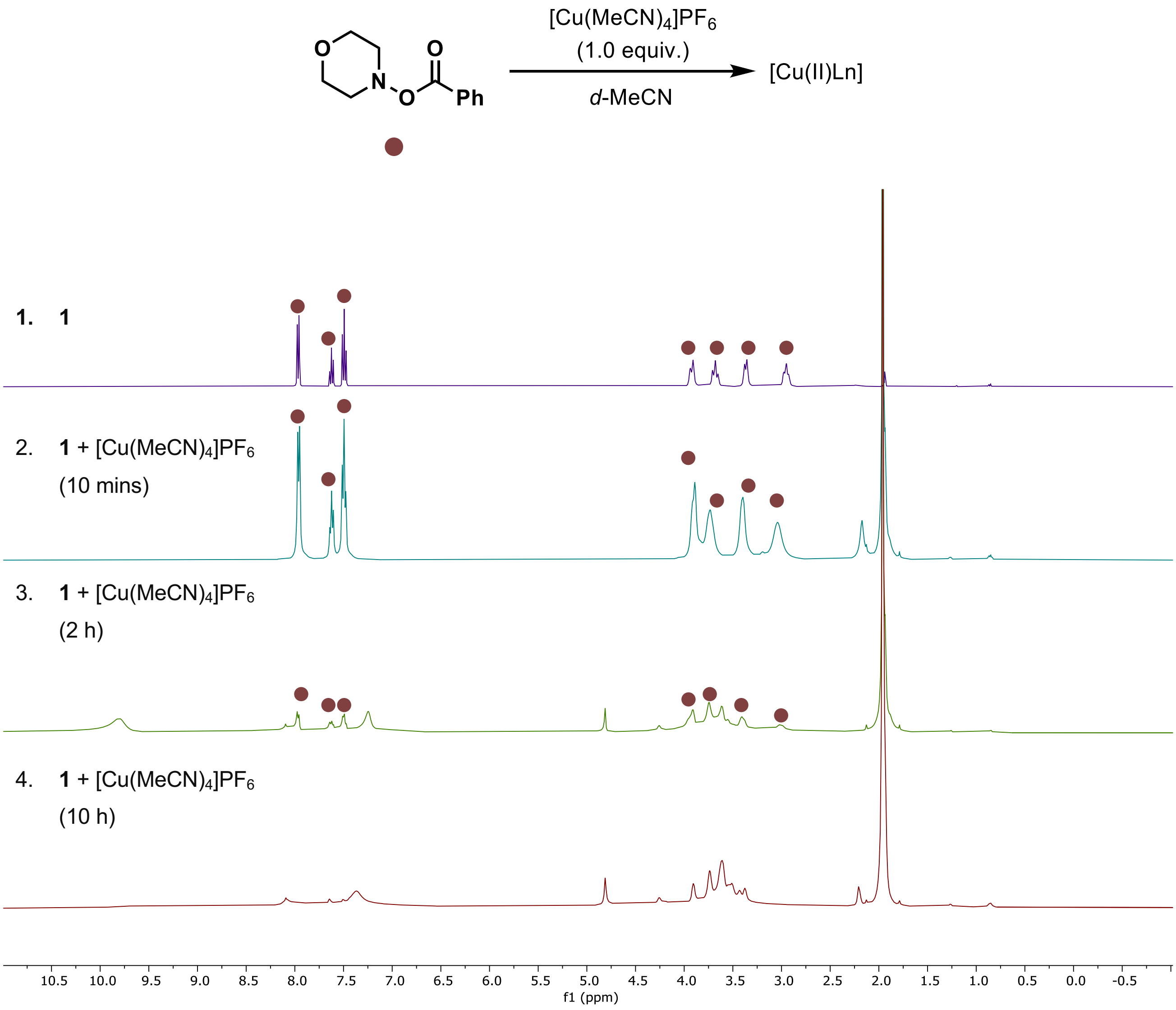

Figure S14: ^{1}H NMR spectroscopy studies towards the oxidation of $Cu(MeCN)_4PF_6$ enabled by reagent 1

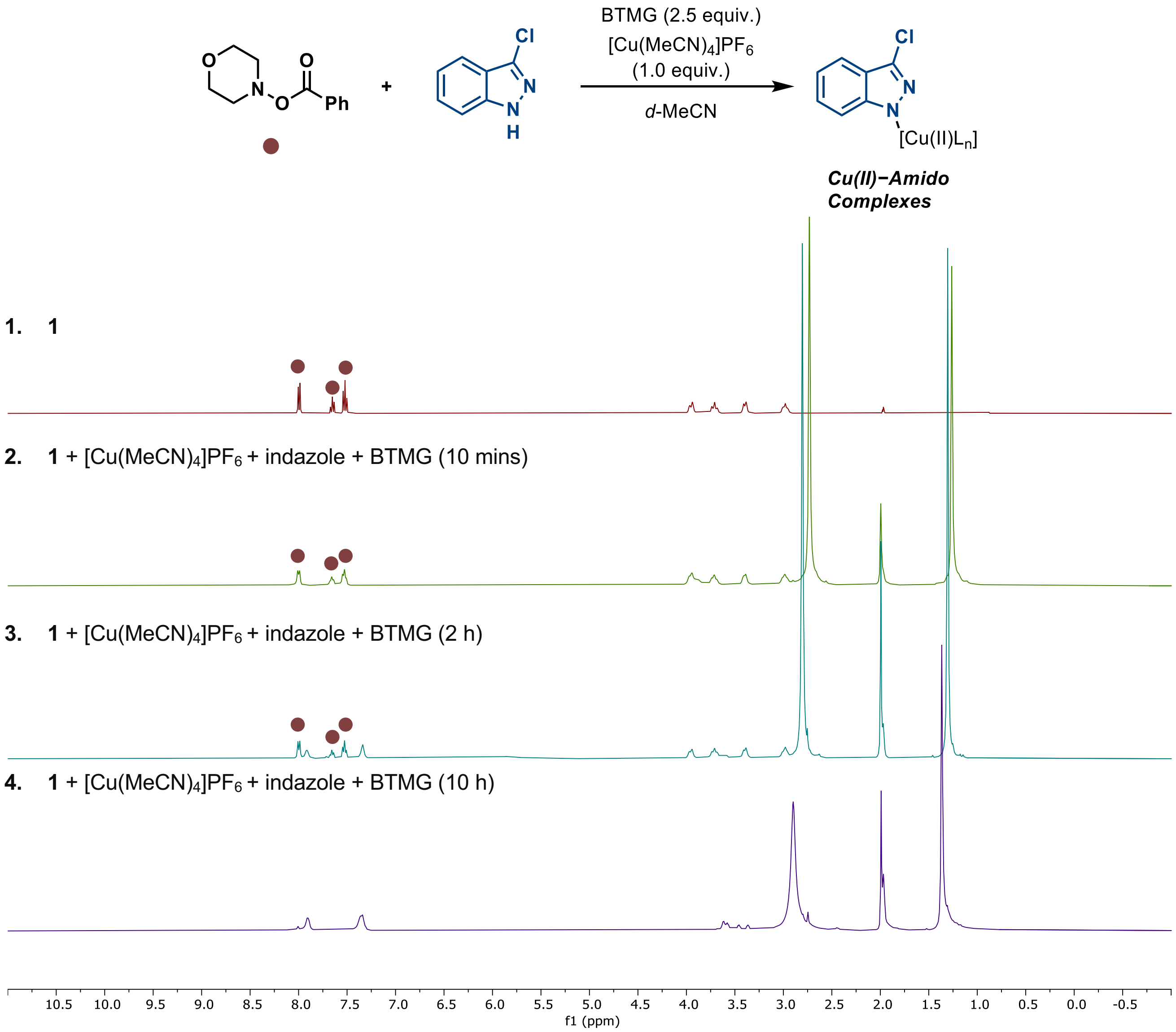

Figure S15: ^{1}H NMR spectroscopy studies towards the oxidation of [L_n–Cu(I)–amido] enabled by reagent 1 with BTMG ligand

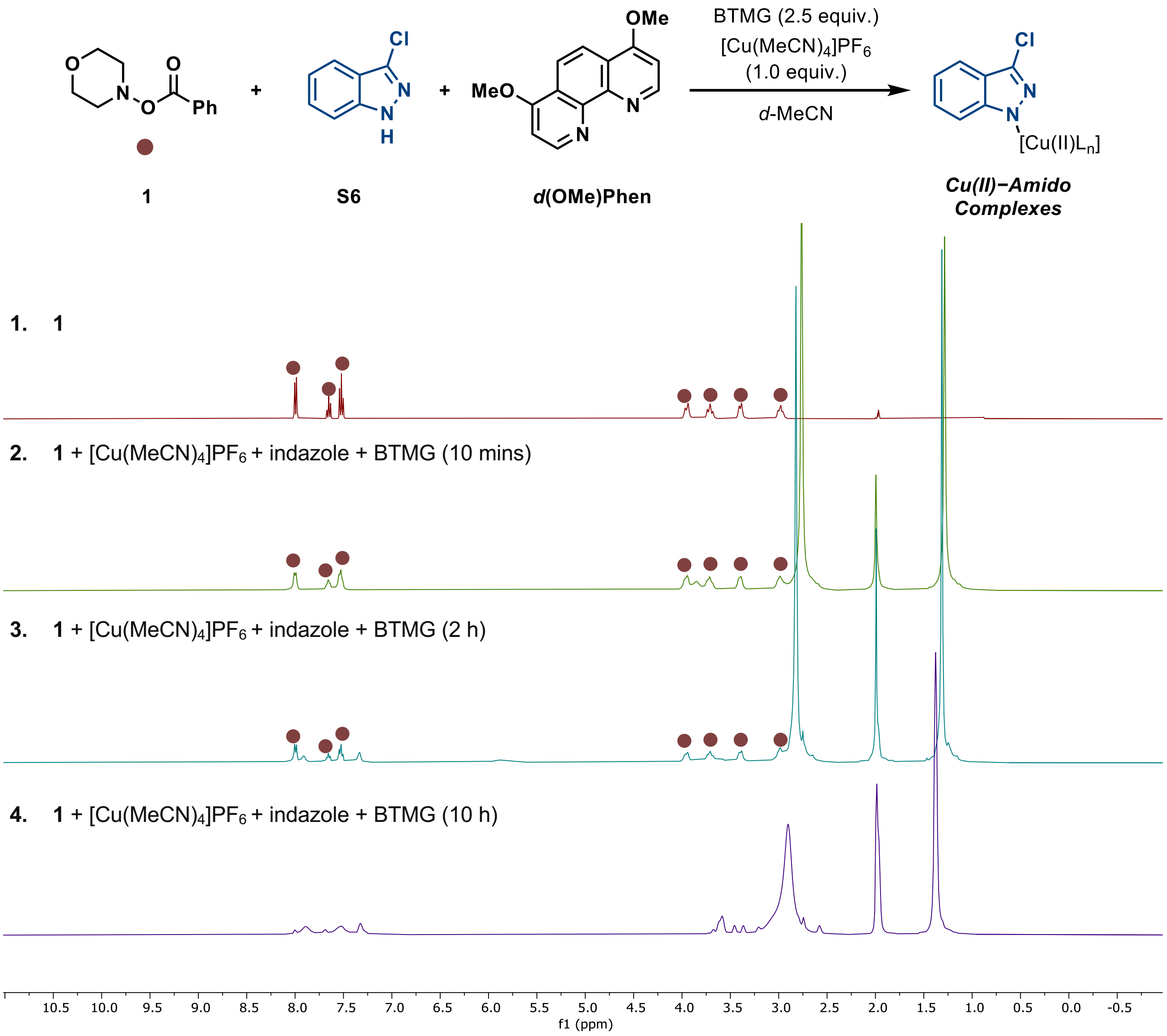

Figure S16: ^{1}H NMR spectroscopy studies towards the oxidation of [L_n–Cu(I)–amido] enabled by reagent 1 with *d*(OMe)Phen and BTMG ligands

In a typical procedure, a flame-dried 6 mL reaction vial equipped with a magnetic stir bar was charged with [$Cu(MeCN)_4$]PF_6 (7.5 mg, 20 μmol, 10 mol%), 4,7-dimethoxy-1,10-phenanthroline (*d*(OMe)Phen, 7.2 mg, 30 μmol, 15 mol%), 3-chloro-1*H*-indazole **S6** (7.86 mg, 50.0 μmol, 1.00 equiv.), and morpholino benzoate **1** (83 mg, 0.40 mmol, 2.0 equiv.) under an ambient atmosphere. The vial was then transferred into an anhydrous, N_2-filled glovebox where anhydrous d_3-MeCN (2.0 mL, *c* = 0.10 M) was added followed by 2-*tert*-butyl-1,1,3,3-tetramethylguanidine (BTMG) (86 mg, 0.10 mL, 0.50 mmol, 2.5 equiv.). After sealing vial with a cap with septum, it was removed from the glovebox and stirred at 60°C for the specific time. Subsequently, the reaction mixture was transferred to an NMR tube for analysis.

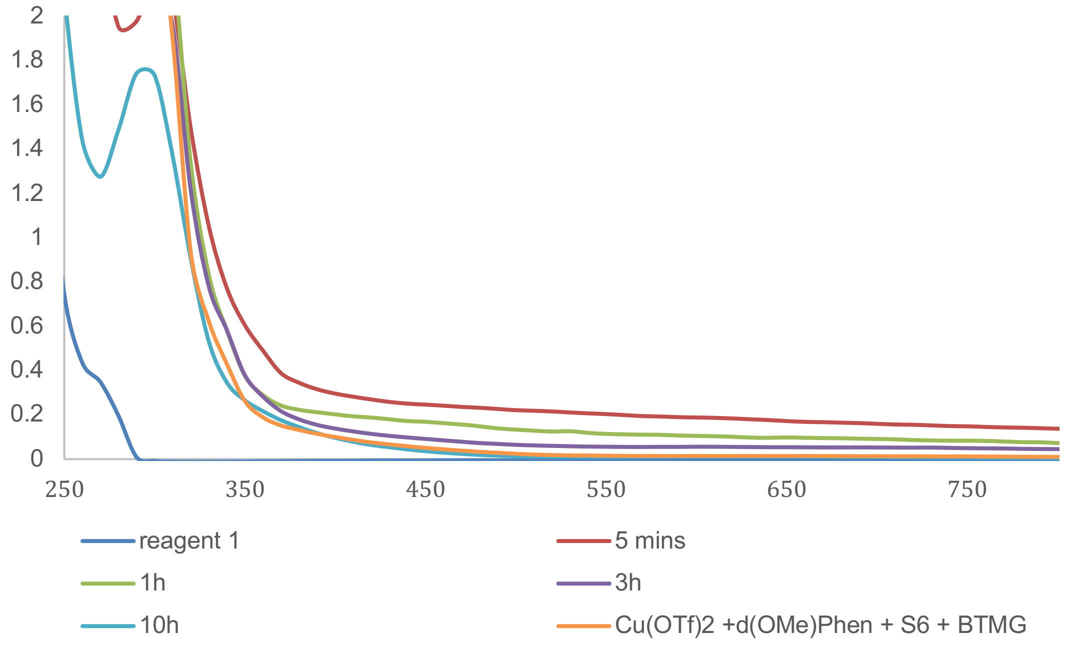

Figure S17: UV/Vis absorption spectroscopy studies towards the oxidation of [L_n–Cu(I)–amido] enabled by reagent 1 with *d*(OMe)Phen and BTMG ligands

In a typical procedure, a flame-dried 6 mL reaction vial equipped with a magnetic stir bar was charged with [$Cu(MeCN)_4$]PF_6 (7.5 mg, 20 μmol, 10 mol%), 4,7-dimethoxy-1,10-phenanthroline (*d*(OMe)Phen, 7.2 mg, 30 μmol, 15 mol%), 3-chloro-1*H*-indazole **S6** (7.86 mg, 50.0 μmol, 1.00 equiv.), and morpholino benzoate **1** (83 mg, 0.40 mmol, 2.0 equiv.) under an ambient atmosphere. The vial was then transferred into an anhydrous, N_2-filled glovebox where anhydrous MeCN (2.0 mL, *c* = 0.10 M) was added followed by 2-*tert*-butyl-1,1,3,3-tetramethylguanidine (BTMG) (86 mg, 0.10 mL, 0.50 mmol, 2.5 equiv.) and cyclohexylboronic acid pinacol ester **3** (84 mg, 0.40 mmol, 2.0 equiv.). After sealing vial with a cap with septum, it was removed from the glovebox and stirred at 60°C for the specific time. Subsequently, this solution was diluted with MeCN to a final volume of 5 mL (*c* = 0.4 mM of **S6**). The absorbance of the resulting 0.4 mM solution was measured using a UV-Vis spectrometer in a cuvette (l = 1.0 cm).

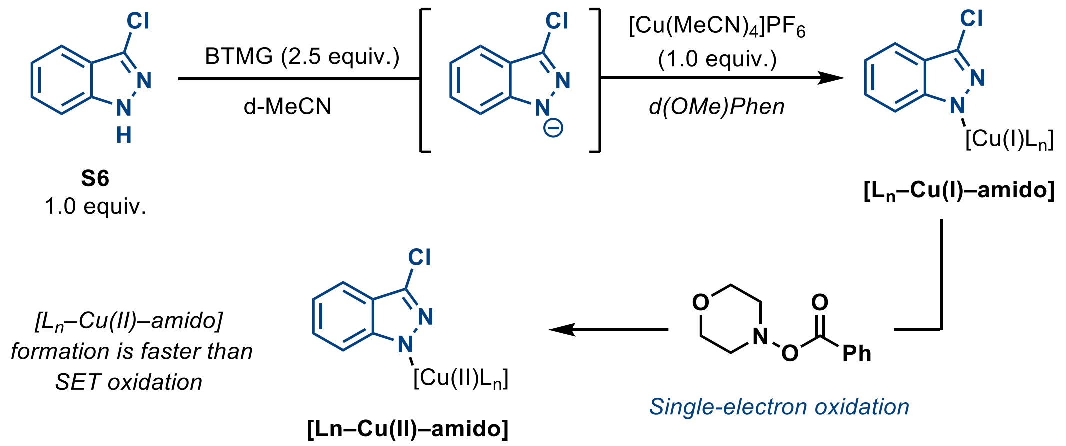

Figure S18: studies towards the oxidation of [L_n–Cu(I)–amido] by reagent 1

3.2.1 Probe the aminyl-radical trapping adduct 99

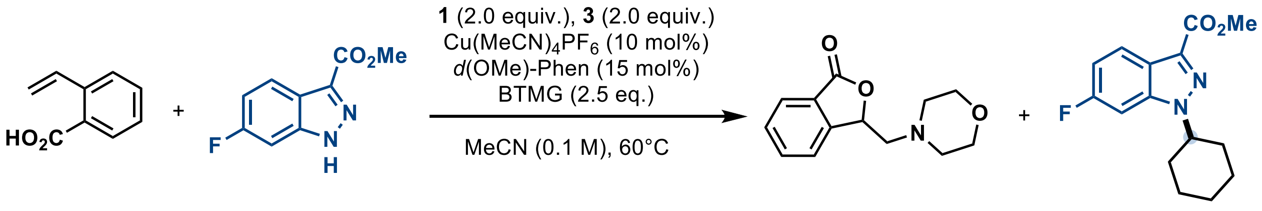

Figure S19: Probe the aminyl radical intermediacy by electrophilic amination

Under an ambient atmosphere, a flame dried 4 mL reaction vial equipped with a magnetic stir bar was charged with [Cu(MeCN)$_4$]PF$_6$ (7.5 mg, 20 µmol, 10 mol%), 4,7-dimethoxy-1,10-phenanthroline (7.2 mg, 30 µmol, 15 mol%), 6-fluoro-1H-indazole-3-carboxylate (40.5 mg, 200 µmol, 1.00 equiv., 96% purity), 2-vinylbenzoic acid (296 mg, 2.00 mmol, 10.0 equiv.), and morpholino benzoate (83 mg, 0.40 mmol, 2.0 equiv.). The vial was transferred into an anhydrous, N$_2$-filled glovebox where anhydrous MeCN was added (2.0 mL, *c* = 0.10 M) followed by 2-*tert*-butyl-1,1,3,3-tetramethylguanidine (BTMG) (86 mg, 0.10 mL, 0.50 mmol, 2.5 equiv.), and cyclohexylboronic acid pinacol ester **3** (84 mg, 0.40 mmol, 2.0 equiv.). The vial was sealed with a cap with septum, removed from the glovebox. After stirring for 16 hours at 60°C, and the reaction mixture was diluted with EtOAc (2 × 3 mL), filtered through a pad of Celite®, and concentrated *in vacuo.* The residue was purified by HPLC (CombiFlash® EZ preparative HPLC system with a Gemini 5 µm NX-C18 110 Å, LC column, MeCN/TFA in water (1/1000, v/v), prewash with 3 mL DMSO) to give **99** (5.0 mg, 5%, retention time: 1.0 min) as a colorless solid and **5** (26.3 mg, 48%, retention time: 28.2 min) as a colorless oil.

NMR Spectroscopy (see spectra):

^{1}**H NMR** (400 MHz, CDCl$_3$): δ_H 7.83 (d, *J* = 7.6 Hz, 1H), 7.62 (d, *J* = 7.4 Hz, 1H), 7.58 – 7.47 (m, 2H), 5.65 – 5.54 (m, 1H), 3.75 – 3.64 (m, 4H), 2.98 (dd, *J* = 55.7, 5.6 Hz, 3H), 2.70 (dd, *J* = 13.3, 6.9 Hz, 3H) ppm.

All recorded spectroscopic data matched those previously reported in the literature.[14]

Cyclohexylboronic acid pinacol ester **3** has a low reduction potential (E_{red} < –2.0 V vs SCE in MeCN), and therefore radical generation by ground-state SET with [Ln–Cu(I)–amido] should not be possible. This is supported by UV/Vis absorption spectroscopy studies (**Figure S13**) where no changes are detected upon treatment of [Ln–Cu(I)–amido] with cyclohexylboronic acid pinacol ester **3**.

A series of ^{1}H NMR studies (**Figures S14–16**) were conducted with [Ln–Cu(I)–amido] (**Figure S14**: Cu(MeCN)$_4$PF$_6$ without any other components, **Figure S15**: Cu(MeCN)$_4$PF$_6$ with BTMG, **Figure S16**: Cu(MeCN)$_4$PF$_6$ with BTMG and *d*(OMe)Phen) treated by reagent **1**. These studies reveal that reagent **1** can directly oxidize Cu(MeCN)$_4$PF$_6$, and its reaction with the [Ln–Cu(I)–amido]-BTMG complex leads to complete oxidation in 10 hours. The addition of *d*(OMe)Phen ligand decreases the rate of this oxidation process, where reagent **1** was not fully consumed in 10 hours. Crucially, combined with the ^{1}H NMR studies of [Ln–Cu(I)–amido] formation showing immediate coordination of deprotonated indazole **S6** to [Ln–Cu(I)] (Figure S7), we believe single-electron oxidation favors [Ln–Cu(I)–amido] over [Ln–Cu(I)]. However, we cannot completely rule out a minor process involving oxidation of [Ln–Cu(I)] by reagent **1**.

Additionally, we monitored the oxidation reaction between [L_n–Cu(I)–amido] and reagent **1**, a diminished

absorbance in the 450–800 nm region by the timeline (5 min – 10 h). Notably, an almost identical profile (10 h) was obtained by reaction of **S6**, BTMG and *d*(OMe)Phen with $Cu(OTf)_2$ to support the formation of [L_n–Cu(**I**)–amido] (**Figure S17**). As the product through sing-electron oxidation of [Ln–Cu(I)–amido] to [Ln–Cu(I)–amido] by reagent **1**, to probe the aminyl radical intermediacy (**Figure S19**), we introduced 2-vinylbenzoic acid under standard conditions to afford amiolactonization product **99**.[14]

3.3 Probing the Formation of Side Product 2

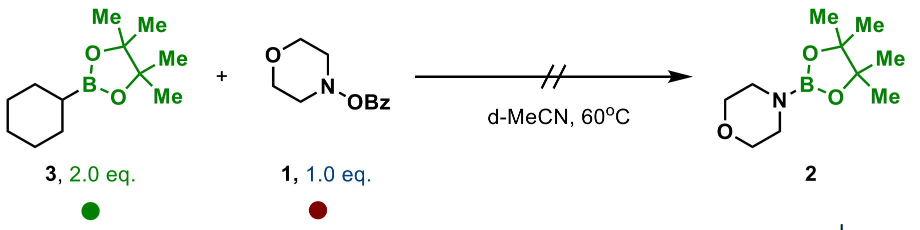

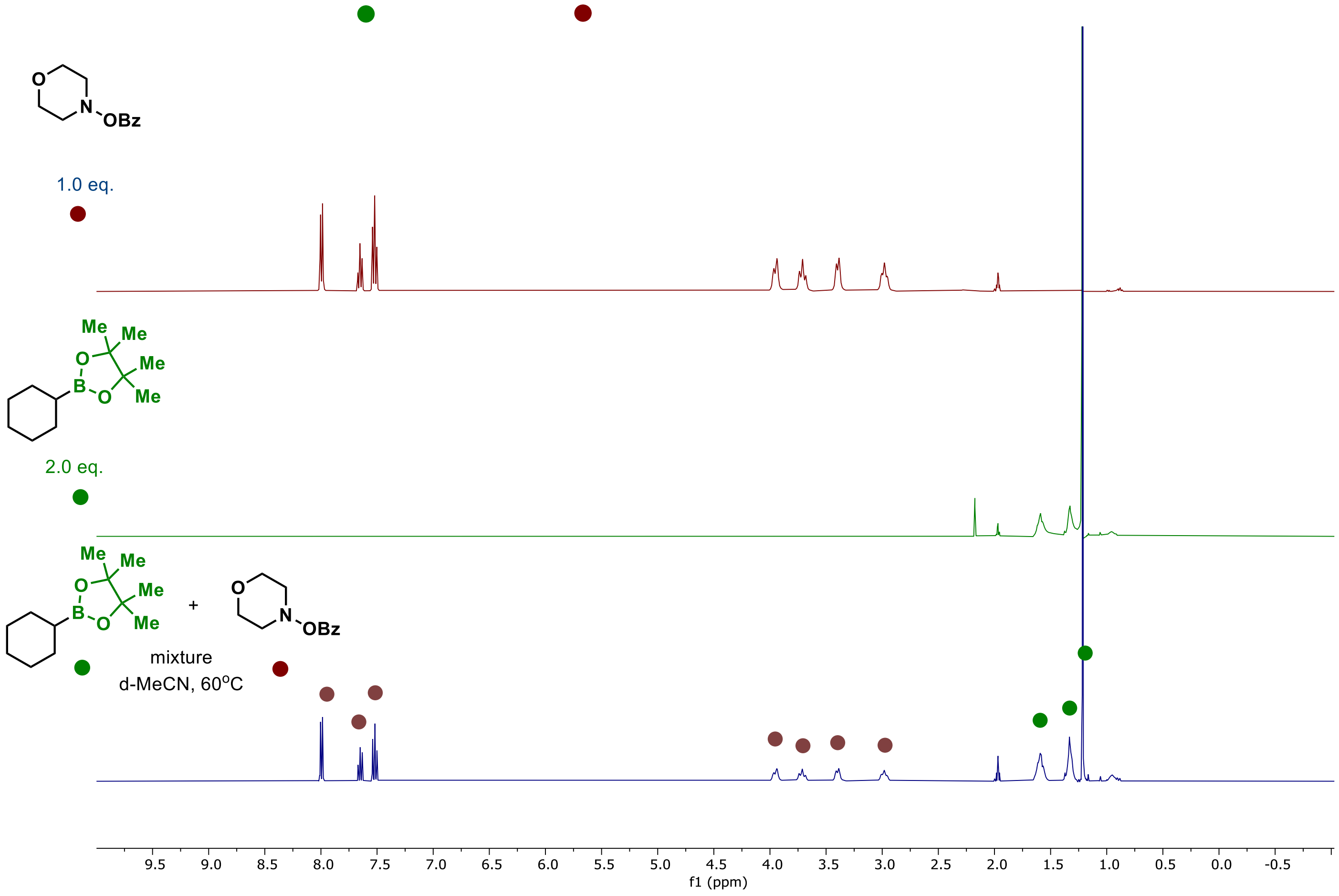

Figure S20: ^{1}H NMR spectroscopy studies towards the interaction between 1 and 3

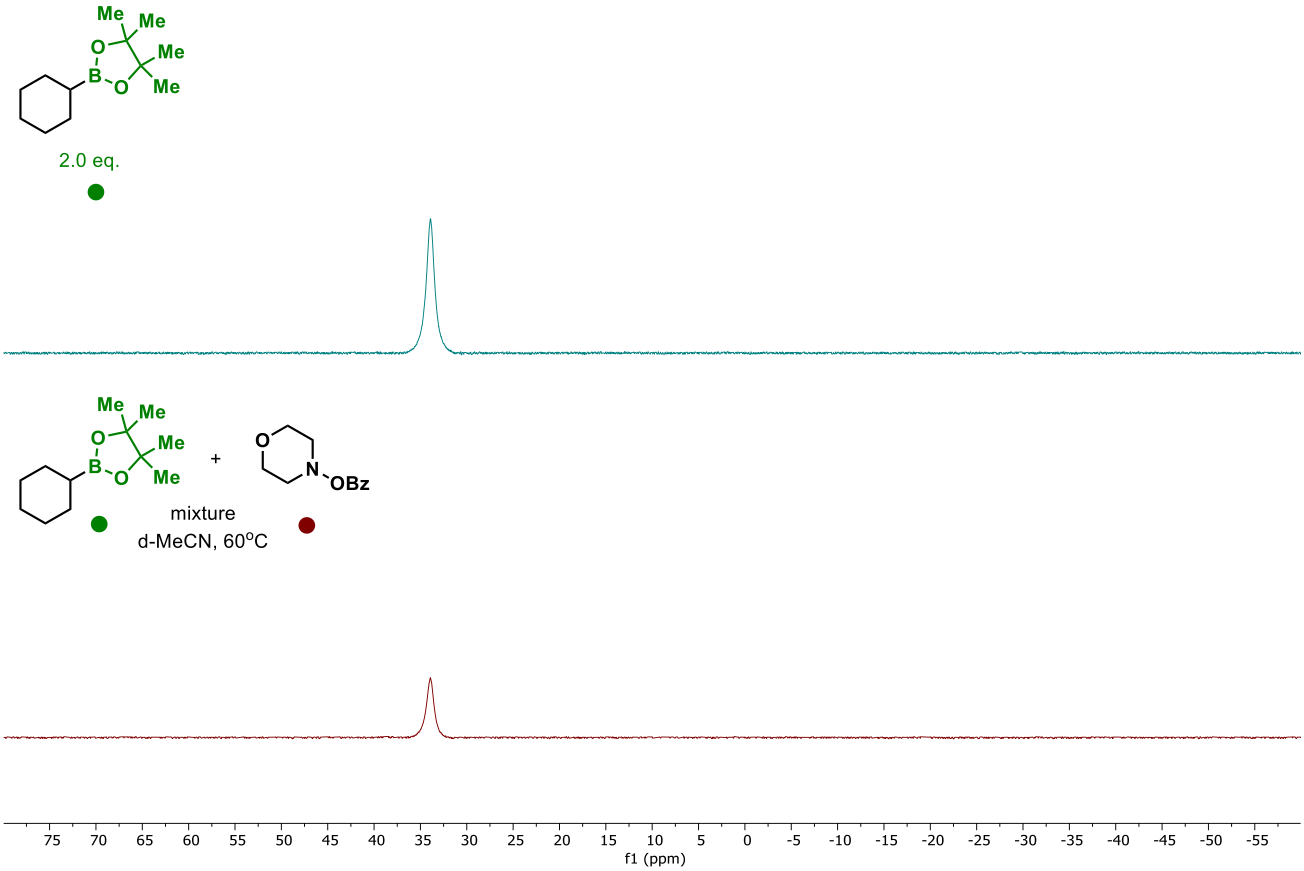

Figure S21: ^{11}B NMR spectroscopy studies towards the interaction between 1 and 3

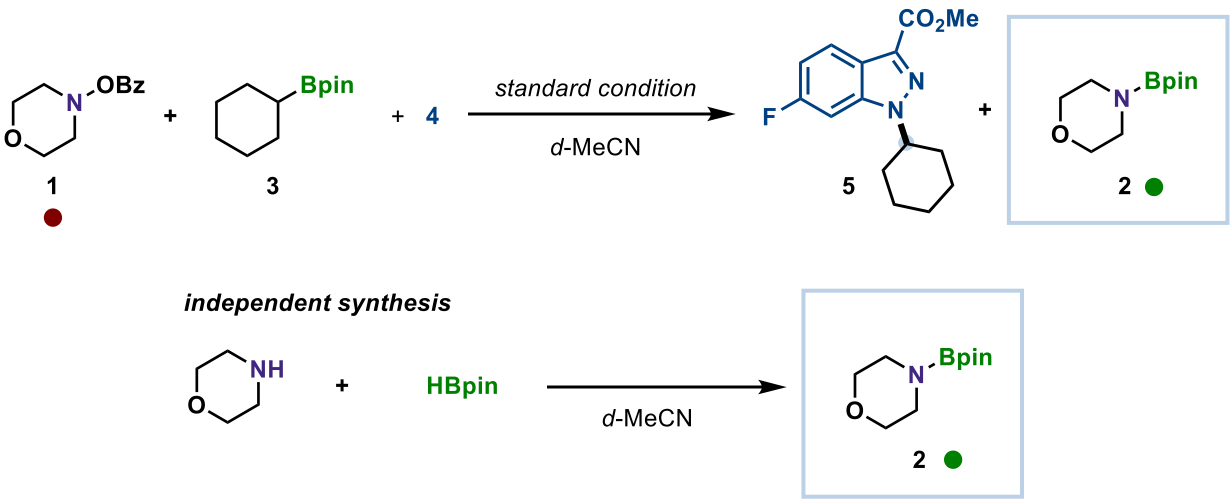

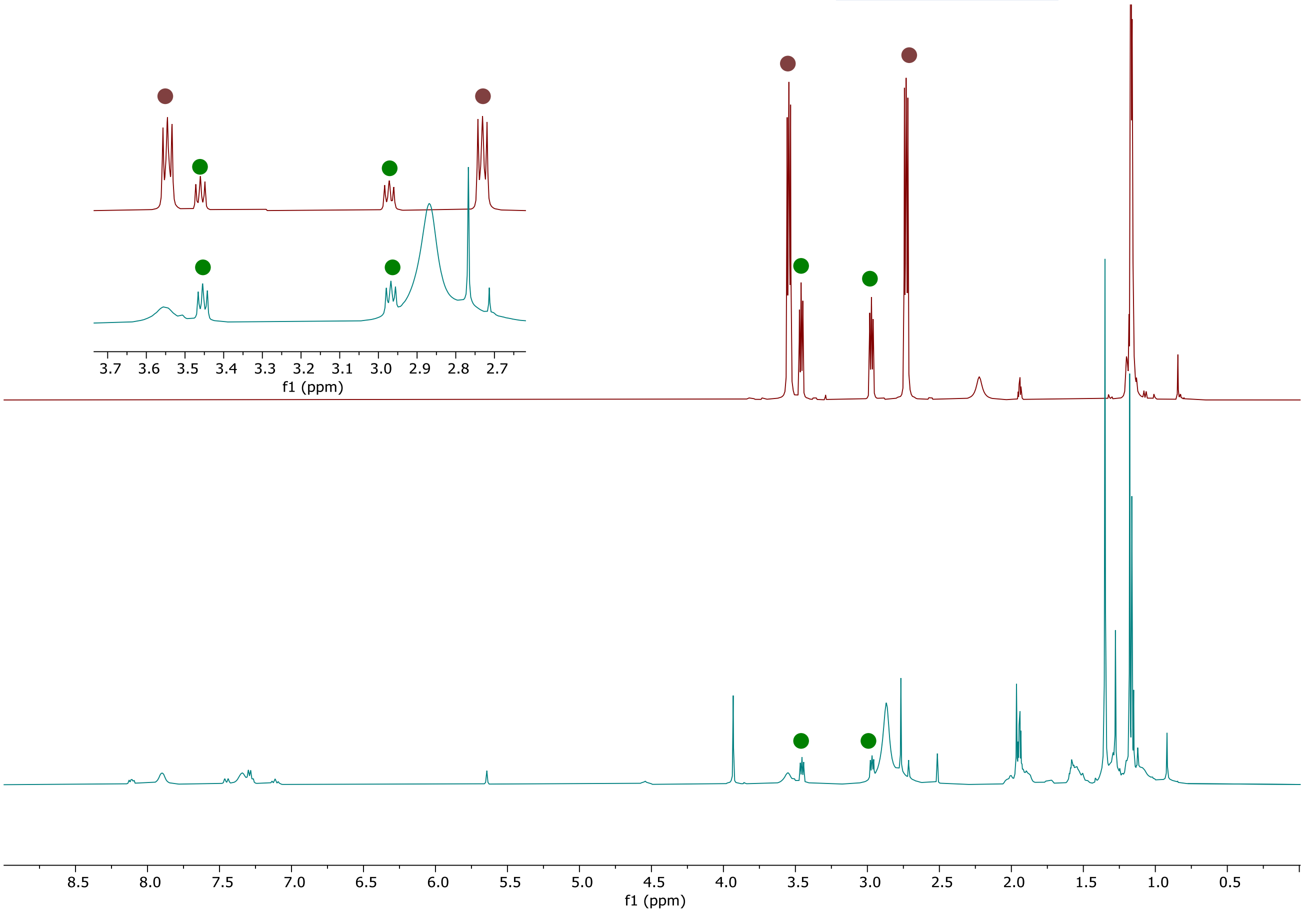

Figure S22: ^{1}H NMR spectroscopy comparison standard reaction formed and independent synthetic 2

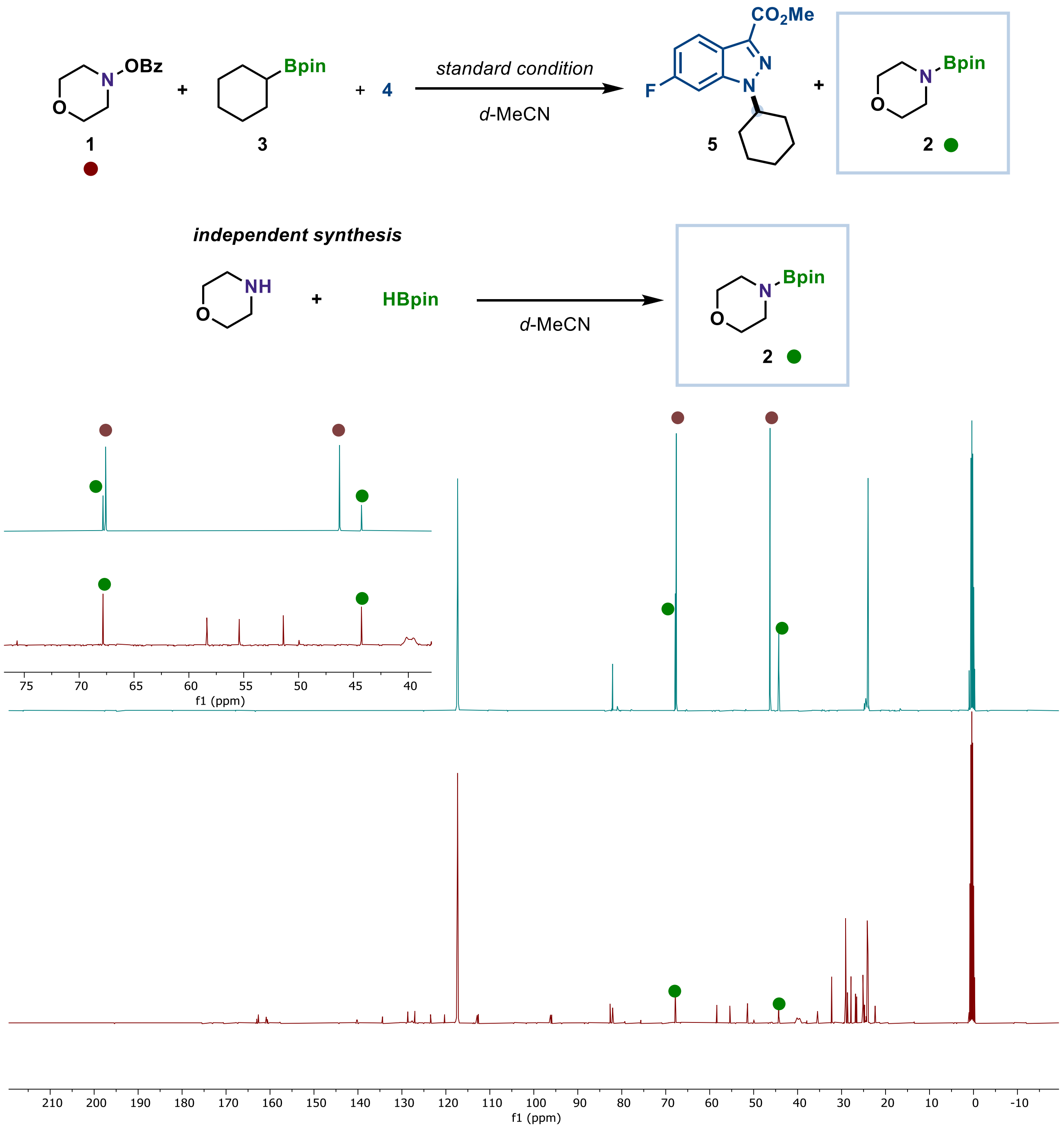

Figure S23: ^{13}C NMR spectroscopy comparison standard reaction formed and independent synthetic 2

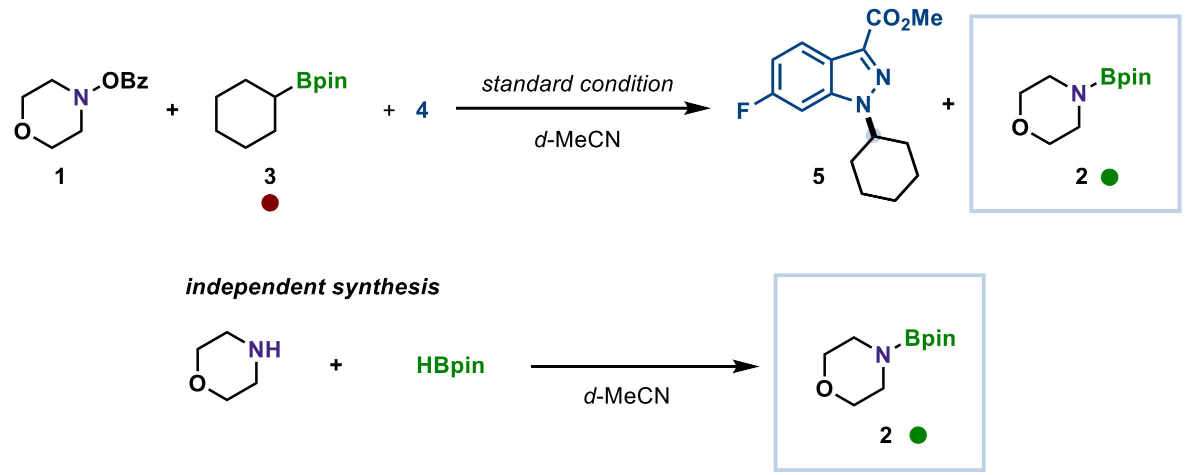

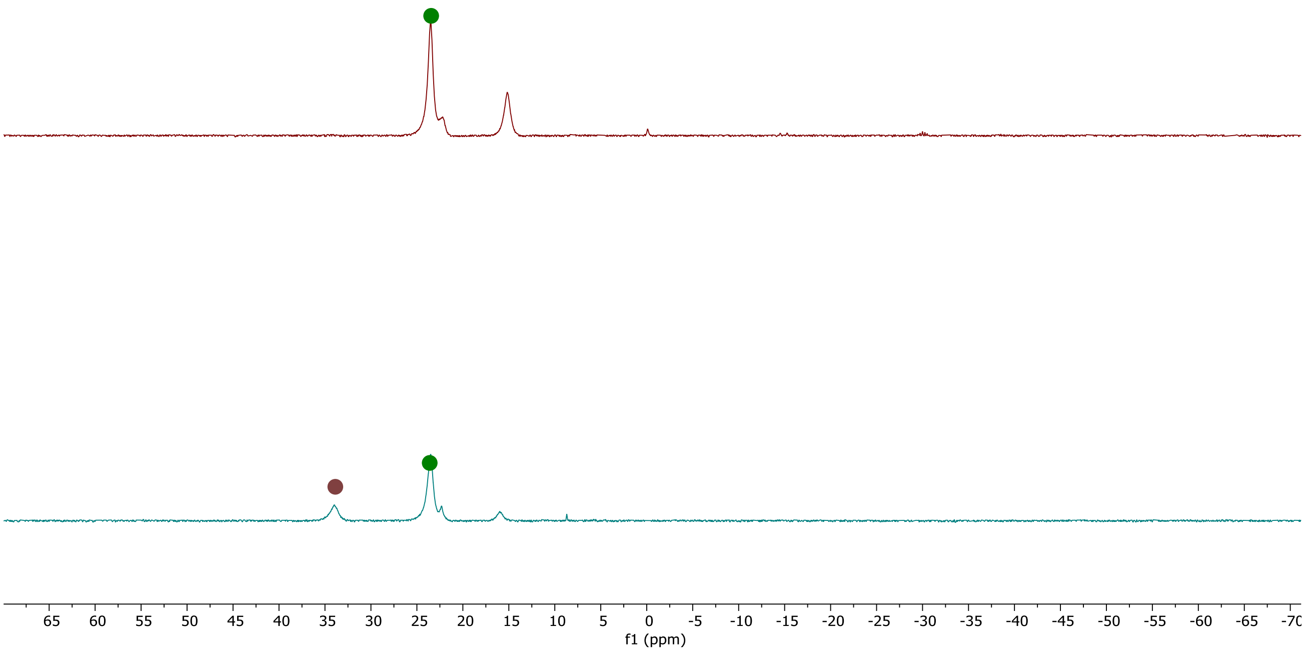

Figure S24: ^{11}B NMR spectroscopy comparison standard reaction formed and independent synthetic 2

Procedure for the NMR analysis of crude reaction mixture: In a general procedure, a flame-dried 6 mL reaction vial equipped with a magnetic stir bar was charged with $[Cu(MeCN)_4]PF_6$ (7.5 mg, 20 µmol, 10 mol%), 4,7-dimethoxy-1,10-phenanthroline (*d*(OMe)Phen, 7.2 mg, 30 µmol, 15 mol%), 3-chloro-1*H*-indazole **S6** (7.86 mg, 50.0 µmol, 1.00 equiv.), morpholino benzoate **1** (83 mg, 0.40 mmol, 2.0 equiv.) and cyclohexylboronic acid pinacol ester **3** (84 mg, 0.40 mmol, 2.0 equiv.) under an ambient atmosphere. The vial was then transferred into an anhydrous, N_2-filled glovebox where anhydrous *d*3-MeCN (2.0 mL, *c* = 0.10 M) was added followed by 2-*tert*-butyl-1,1,3,3-tetramethylguanidine (BTMG) (86 mg, 0.10 mL, 0.50 mmol, 2.5 equiv.). After sealing vial with a cap with septum, it was removed from the glovebox and stirred at 60°C for 16 h. Subsequently, the crude reaction mixture was transferred to an NMR tube for analysis.

Procedure for the NMR analysis of independent synthetic 2: In an N_2-filled glovebox, a flame-dried 6 mL reaction vial equipped with a magnetic stir bar was charged with pinacolatoborane (25.6 mg, 29.0 µL, 0.200 mmol, 1.00 equiv.) and morpholine (34.8 mg, 34.5 µL, 0.400 mmol, 2.00 equiv.) in MeCN (2.0 mL, *c* = 0.10 M). After sealing the vial with a septum cap, it was removed from the glovebox and stirred at room temperature for 4 h. Subsequently, the crude reaction mixture was transferred to an NMR tube for analysis.

^{1}H and ^{11}B NMR studies demonstrate there was no interaction between **1** and **3** to give byproduct **2** (**Figures S21–22**). To verify the byproduct morpholinoboronic ester **2**, we synthesized it independently by dehydrogenating pinacoborane (HBPin) with morpholine.[15] A series of NMR spectra of **2** obtained under this standard condition were compared with those of independently synthesized **2** (**Figures S22–24**). The outcome reveals identical spectroscopic features, confirming that **2** is indeed the byproduct in our reaction.

3.4 Evidence for Alkyl Radical Intermediacy

3.4.1 TEMPO trapping experiment for alkyl radical trapping adduct 100

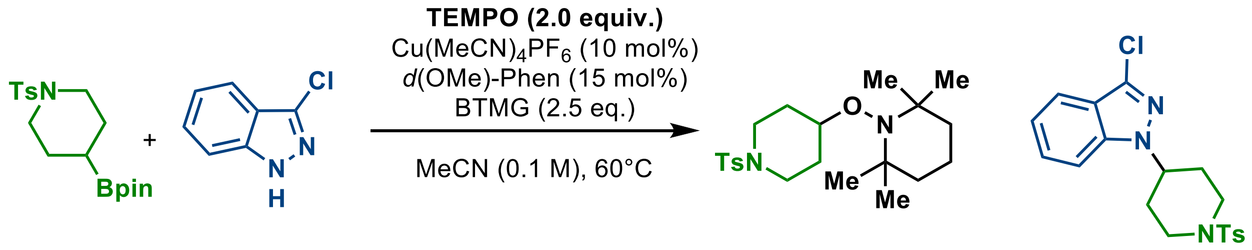

2.0 equiv. **100,** 82% **66**, 4%

Figure S25: TEMPO trapping for alkyl radical

Under an ambient atmosphere, a flame dried 4 mL reaction vial equipped with a magnetic stir bar was charged with $[Cu(MeCN)_4]PF_6$ (7.5 mg, 20 µmol, 10 mol%), 4,7-dimethoxy-1,10-phenanthroline (7.2 mg, 30 µmol, 15 mol%), 3-chloro-1*H*-indazole (31.8 mg, 200 µmol, 1.00 equiv., 96% purity), TEMPO (62.5 mg, 400 µmol, 2.00 equiv.), and morpholino benzoate **1** (83 mg, 0.40 mmol, 2.0 equiv.). The vial was transferred into an anhydrous, N_2-filled glovebox where anhydrous MeCN was added (4.0 mL, *c* = 0.050 M) followed by 2-*tert*-butyl-1,1,3,3-tetramethylguanidine (BTMG) (86 mg, 0.10 mL, 0.50 mmol, 2.5 equiv.), and 4-(4,4,5,5-tetramethyl-1,3,2-dioxaborolan-2-yl)-1-tosylpiperidine (146 mg, 0.400 mmol, 2.00 equiv.). The vial was sealed with a cap with septum, removed from the glovebox. After stirring for 16 hours at 60°C, and the reaction mixture was diluted with EtOAc (2 × 3 mL), filtered through a pad of Celite®, and concentrated *in vacuo.* The residue was adsorbed on Celite® and purified by CombiFlash® R_f purification system using RediSep R_f Gold® silica gel (40 µm), eluting with EtOAc/heptane (0 – 30%, v/v), giving **100** (129 mg, 82%) as a colorless solid and **66** (3.5 mg, 4%) as a colorless oil.

NMR Spectroscopy (*<u>see spectra</u>*):

^{1}H NMR (400 MHz, $CDCl_3$): δ_H 7.63 (d, *J* = 7.9 Hz, 2H), 7.32 (d, *J* = 7.8 Hz, 2H), 3.71 – 3.48 (m, 3H), 2.49 -2.40 (m, 5H), 2.11 – 1.97 (m, 2H), 1.71 – 1.55 (m, 2H), 1.41 (d, *J* = 4.8 Hz, 6H), 1.04 (s, 12H) ppm.

^{13}C NMR (101 MHz, $CDCl_3$): δ_C 143.5, 133.2, 129.6, 127.7, 78.5, 59.7, 45.0, 40.2, 34.4, 31.2, 21.6, 20.2, 17.2 ppm.

All recorded spectroscopic data matched those previously reported in the literature.[16]

3.4.2 5-*exo*-trig cyclization experiment for alkyl radical trapping product 101a and 101b

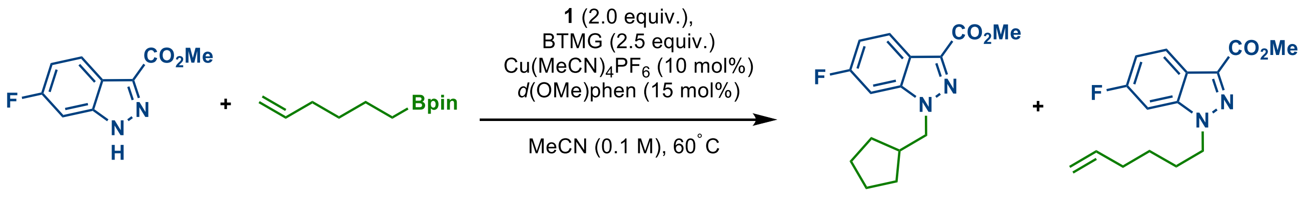

Figure S26: 5-*exo*-trig cyclization of alkyl radical

Under an ambient atmosphere, a flame dried 4 mL reaction vial equipped with a magnetic stir bar was charged with [$Cu(MeCN)_4$]PF_6 (7.5 mg, 20 µmol, 10 mol%), 4,7-dimethoxy-1,10-phenanthroline (7.2 mg, 30 µmol, 15 mol%), methyl 6-fluoro-1*H*-indazole-3-carboxylate (40.5 mg, 200 µmol, 1.00 equiv., 97% purity), and morpholino benzoate **1** (83 mg, 0.40 mmol, 2.0 equiv.). The vial was transferred into an anhydrous, N_2-filled glovebox where anhydrous MeCN was added (4.0 mL, *c* = 0.050 M) followed by 2-*tert*-butyl-1,1,3,3-tetramethylguanidine (BTMG) (86 mg, 0.10 mL, 0.50 mmol, 2.5 equiv.), and 2-(hex-5-en-1-yl)-4,4,5,5-tetramethyl-1,3,2-dioxaborolane (84 mg, 0.40 mmol, 2.0 equiv.). The vial was sealed with a cap with septum, removed from the glovebox. After stirring for 16 hours at 60°C, and the reaction mixture was diluted with EtOAc (2 × 3 mL), filtered through a pad of Celite®, and concentrated *in vacuo.* The residue was adsorbed on Celite® and purified by CombiFlash® R_f purification system using RediSep R_f Gold® silica gel (40 µm), eluting with EtOAc/heptane (0 – 30%, v/v), giving a mixture of **101a** and **101b** (47.8 mg, 88% (69% + 19%), 3.6:1 determined by ^{19}F NMR) as a colorless oil.

NMR Spectroscopy of 101a (*see spectra*):

^{1}H NMR (400 MHz, $CDCl_3$): δ_H 8.18 (dd, *J* = 8.9, 5.2 Hz, 1H), 7.15 – 7.03 (m, 2H), 4.33 (d, *J* = 7.6 Hz, 2H), 4.03 (s, 3H), 2.59 (hept, *J* = 7.6 Hz, 1H), 1.75 – 1.59 (m, 4H), 1.57 (s, 2H), 1.37 – 1.22 (m, 2H) ppm.

^{13}C NMR (101 MHz, $CDCl_3$): δ_C 163.0, 162.3 (d, *J* = 246.4 Hz), 141.1 (d, *J* = 12.2 Hz), 134.8, 124.0 (d, *J* = 10.7 Hz), 120.6, 113.2 (d, *J* = 25.8 Hz), 95.6 (d, *J* = 26.5 Hz), 54.8, 52.3, 40.6, 30.5, 25.1 ppm.

^{19}F NMR (376 MHz, $CDCl_3$): δ_F –113.27 (td, *J* = 9.3, 5.3 Hz) ppm.

HRMS (ESI) m/z calc'd for $C_{15}H_{18}N_2O_2F$ [M+H]$^+$, 277.1352; found, 277.1349.

NMR Spectroscopy of 101b (*see spectra*):

^{1}H NMR (400 MHz, $CDCl_3$): δ_H 8.18 (dd, J = 8.9, 5.2 Hz, 1H), 7.15 – 7.03 (m, 2H), 5.85 – 5.62 (m, 1H), 5.03 – 4.91 (m, 2H), 4.40 (t, J = 7.3 Hz, 2H), 4.03 (s, 3H), 2.06 (p, J = 6.8 Hz, 3H), 1.95 (dd, J = 8.9, 6.5 Hz, 3H), 1.42 (dd, J = 9.1, 6.4 Hz, 3H) ppm.

^{13}C NMR (101 MHz, $CDCl_3$): δ_C 163.0, 162.3 (d, *J* = 246.4 Hz), 141.1 (d, *J* = 12.2 Hz), 137.9, 134.8, 124.0 (d, *J* = 10.7 Hz), 120.6, 115.2, 113.2 (d, *J* = 25.8 Hz), 95.6 (d, *J* = 26.5 Hz), 50.0, 33.2, 29.1, 26.0 ppm.

^{19}F NMR (376 MHz, $CDCl_3$): δ_F –113.05 (td, *J* = 8.9, 5.2 Hz) ppm.

HRMS (ESI) m/z calc'd for $C_{15}H_{18}N_2O_2F$ [M+H]$^+$, 277.1352; found, 277.1349.

To probe the alkyl radical intermediacy, TEMPO trapping and 5-*exo*-trig cyclization experiments were conducted. In the presence of 2,2,6,6-tetramethylpiperidin-1-*oxyl* (TEMPO), the alkyl-trapped TEMPO product **100** was isolated in 87% yield. A radical clock experiment offered complementary evidence: a 5-*exo*-trig-amination gave cyclopentylmethyl indazole **101a**, along with a linear derivative **101b** in a 3.6:1 ratio (**101a**:**101b**). Both experiments provide support for alkyl radical intermediacy.

3.5 Probing Radical Capture by [Cu(II)–amido] Species as C–N Bond Forming Step

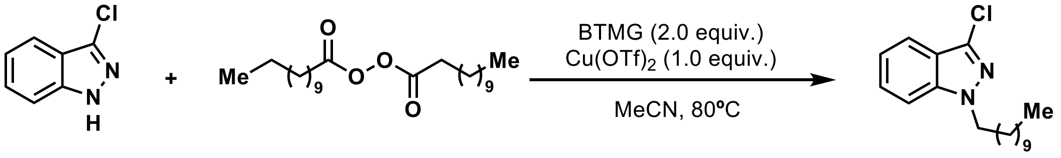

B. Górski, A.-L. Barthelemy, J. J. Douglas, F. Juliá, D. Leonori, *Nat. Catal.* **2021**, *4*, 623–630.

Figure S27: Probing radical capture by [Cu(II)–amido] species as C–N bond forming step

To obtain more information supporting the radical capture of the alkyl radical by the [Cu(II)–amido] species followed by reductive elimination, the reaction between a pre-formed [LnCu(II)]–**S6** complex and lauroyl peroxide was supported by previous studies[8].As lauroyl peroxide leads to alkyl radical formation by thermal O–O bond homolysis[17,18], the alkyl radical was generated without single-electron transfer. The desired *N*-alkylated product was observed in 65% yield, as previously reported[8].

6. SPECTROSCOPIC DATA

^{1}H NMR (400 MHz, $CDCl_3$) of **5** (*see procedure*)

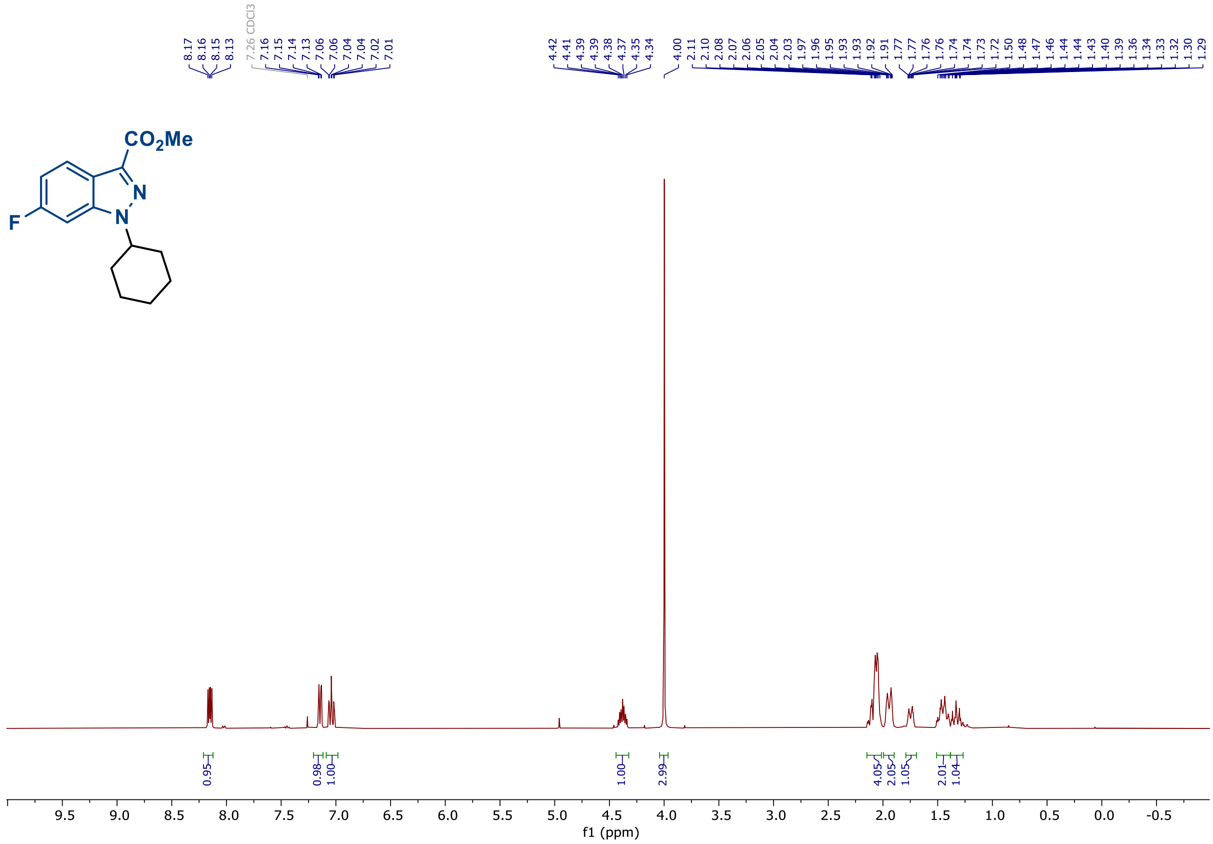

^{13}C NMR (101 MHz, $CDCl_3$) of **5**

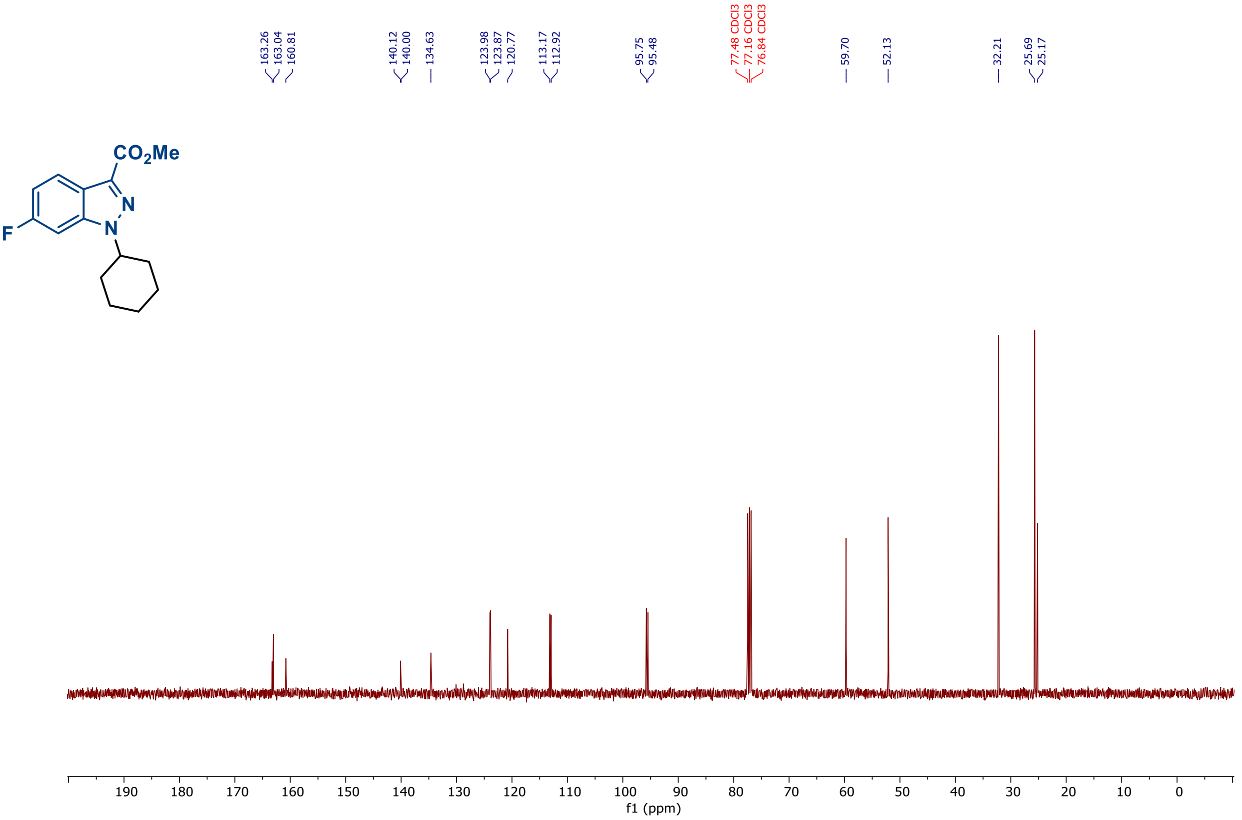

^{19}F NMR (376 MHz, $CDCl_3$) of **5**

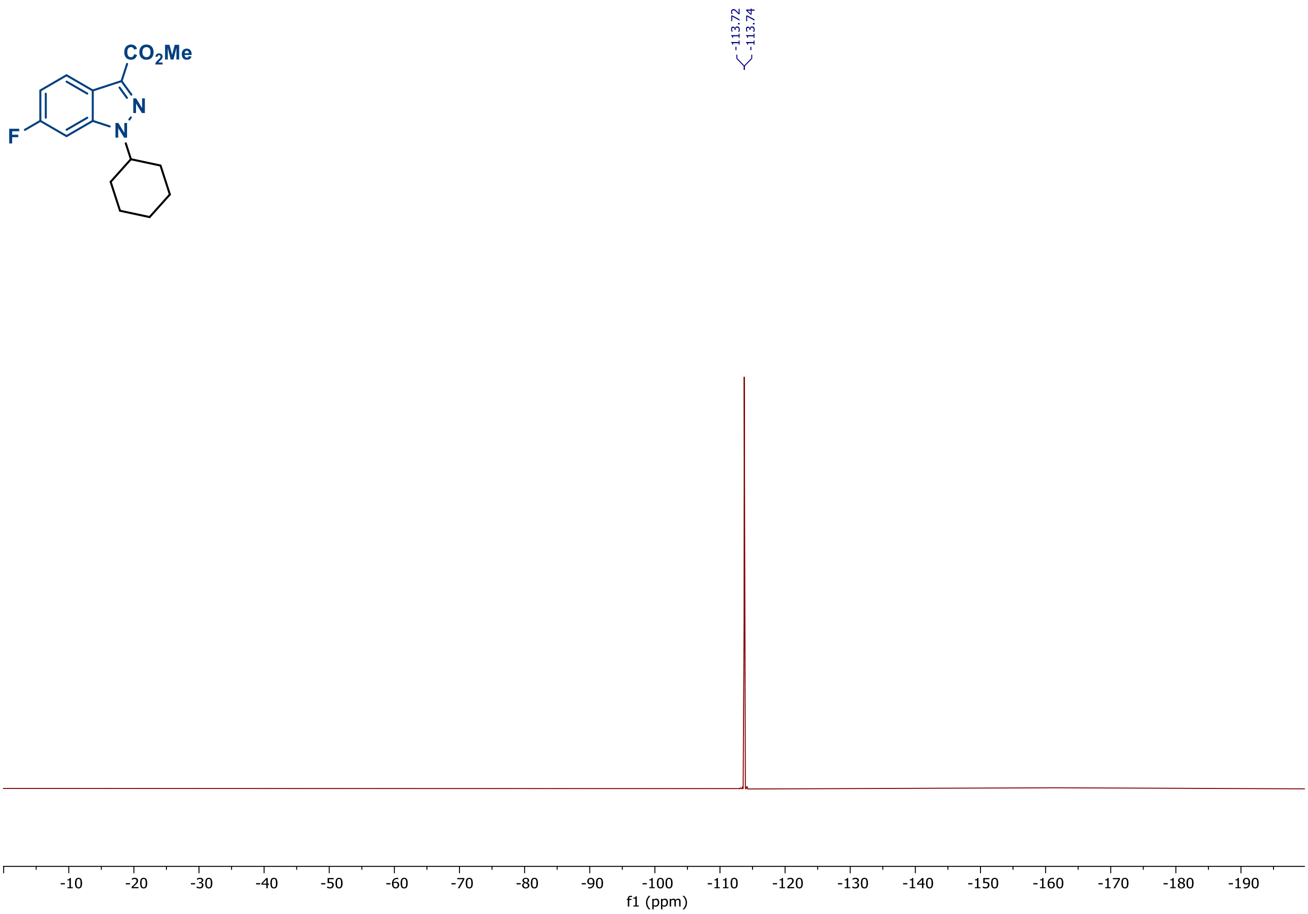

^{1}H NMR (400 MHz, $CDCl_3$) of **6** (*see procedure*)

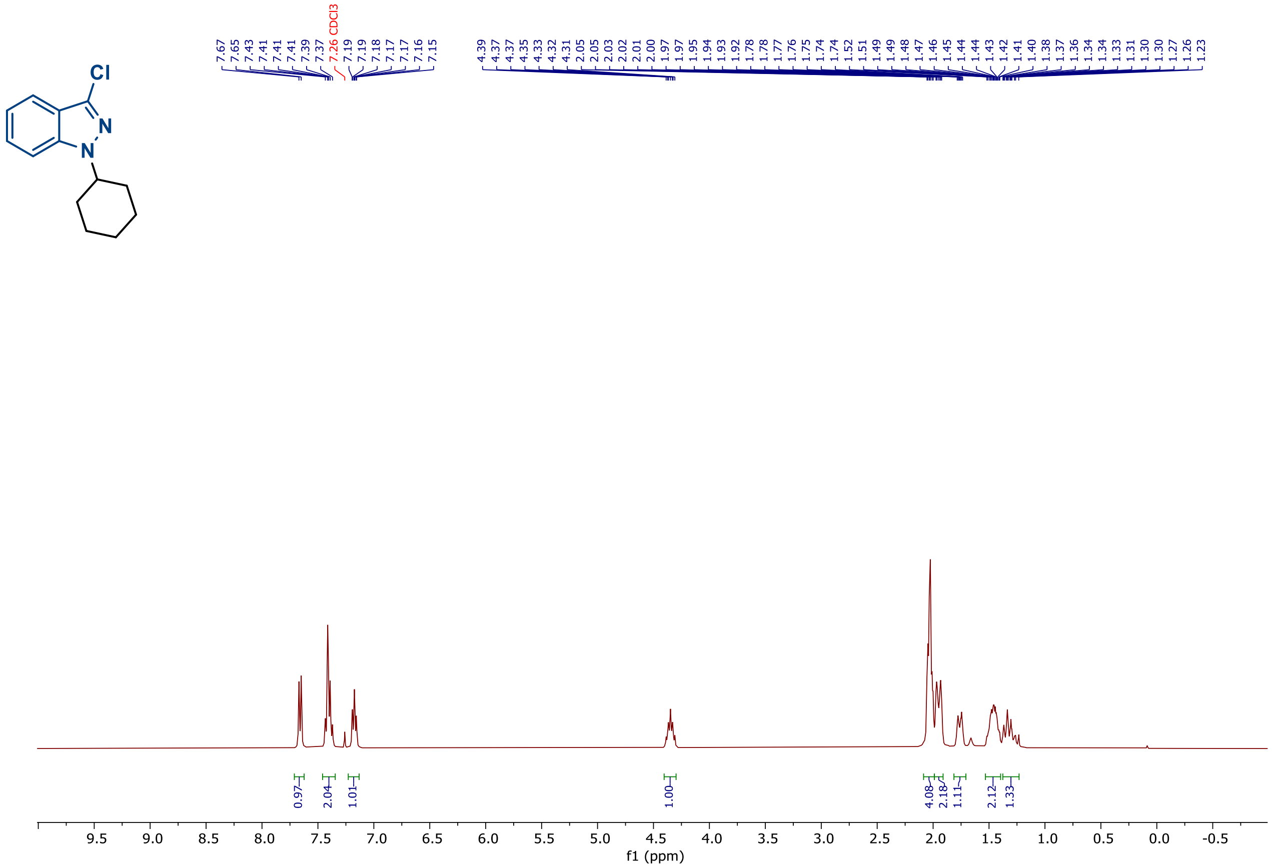

^{13}C NMR (101 MHz, $CDCl_3$) of **6**

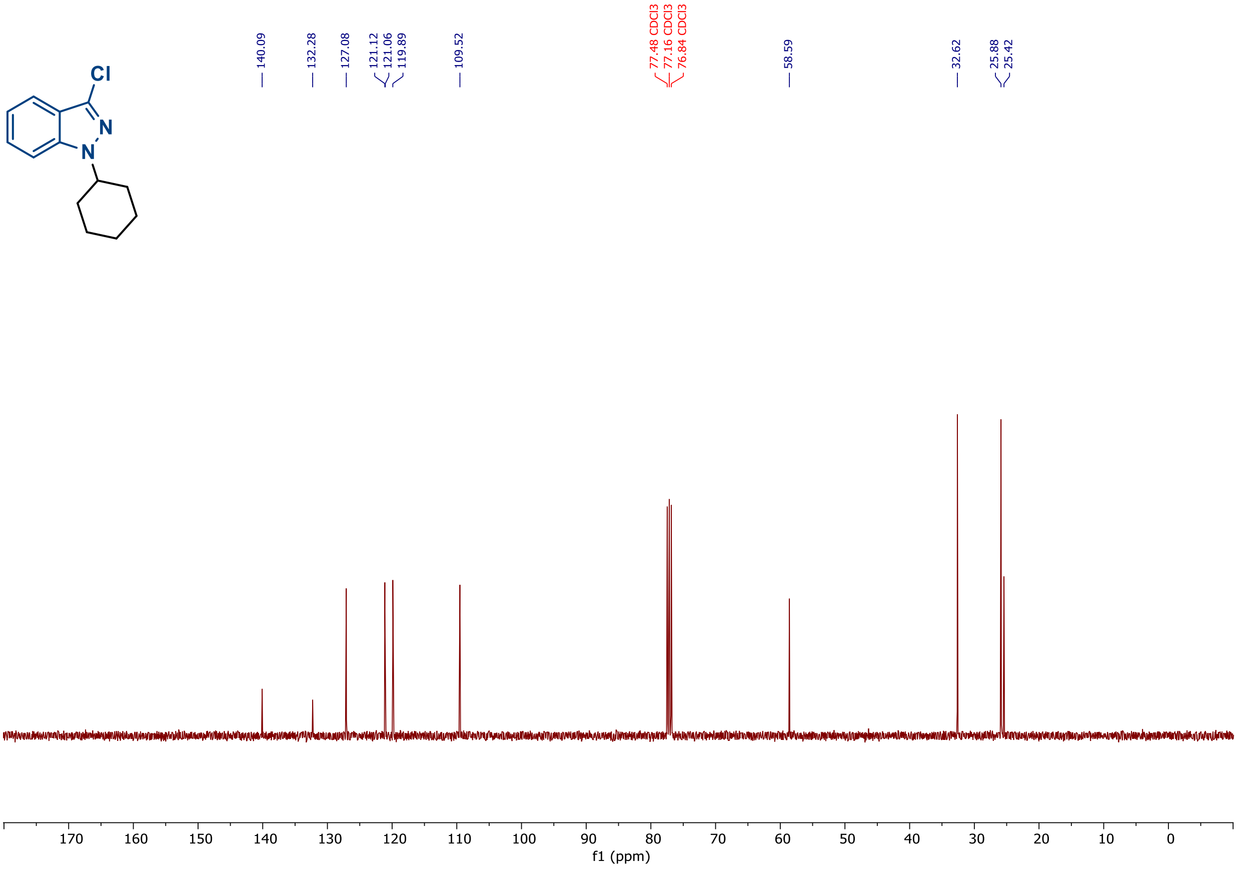

^{1}H NMR (400 MHz, $CDCl_3$) of **7** (*see procedure*)

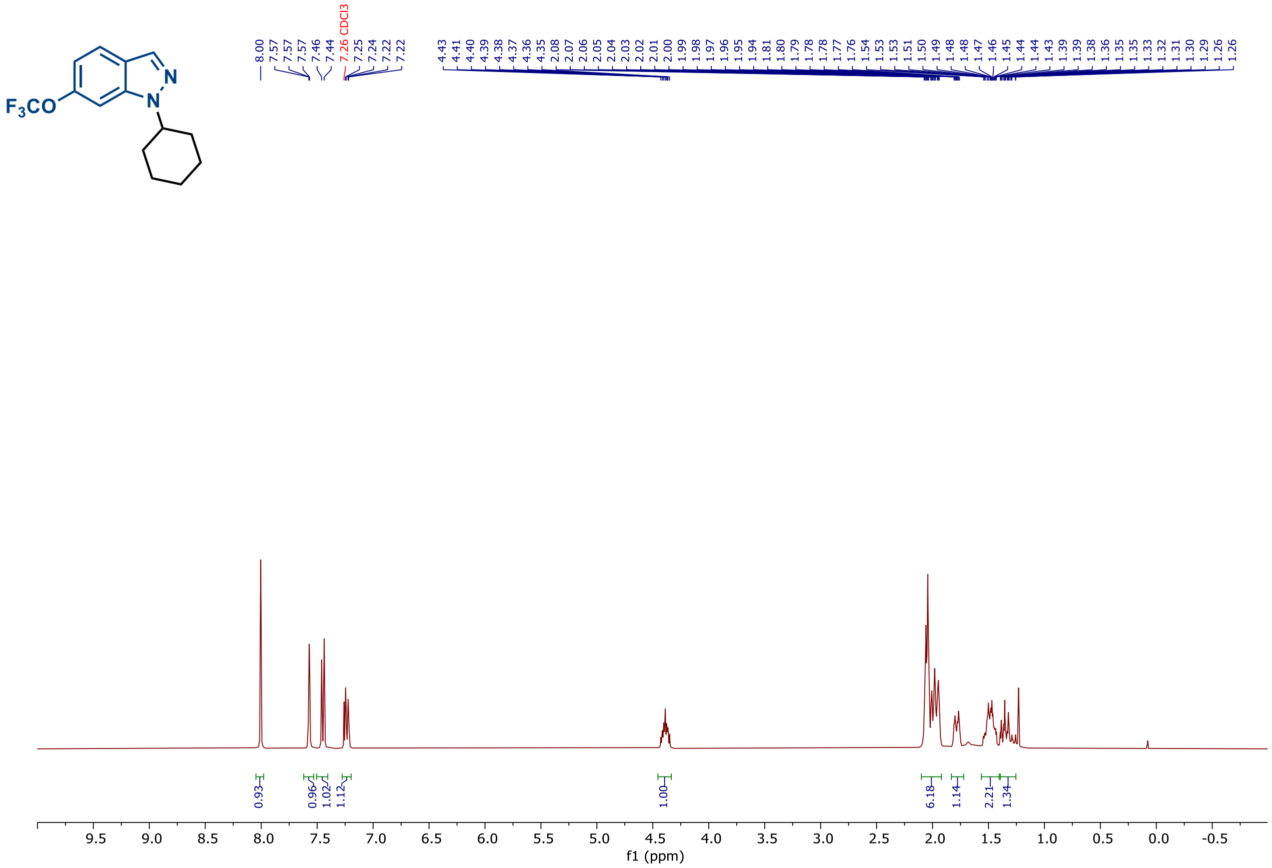

^{13}C NMR (101 MHz, $CDCl_3$) of **7**

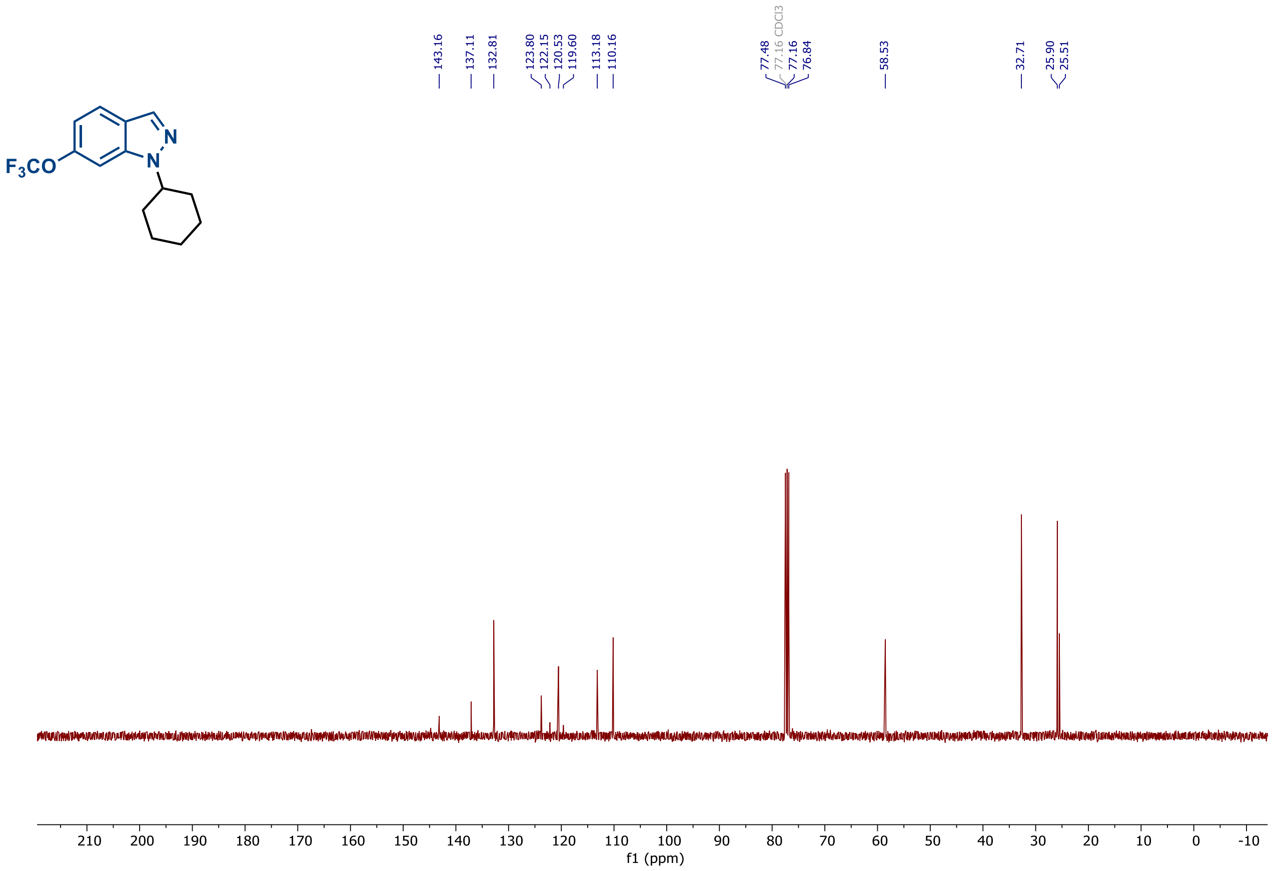

^{19}F NMR (376 MHz, $CDCl_3$) of **7**

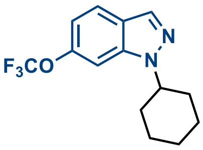

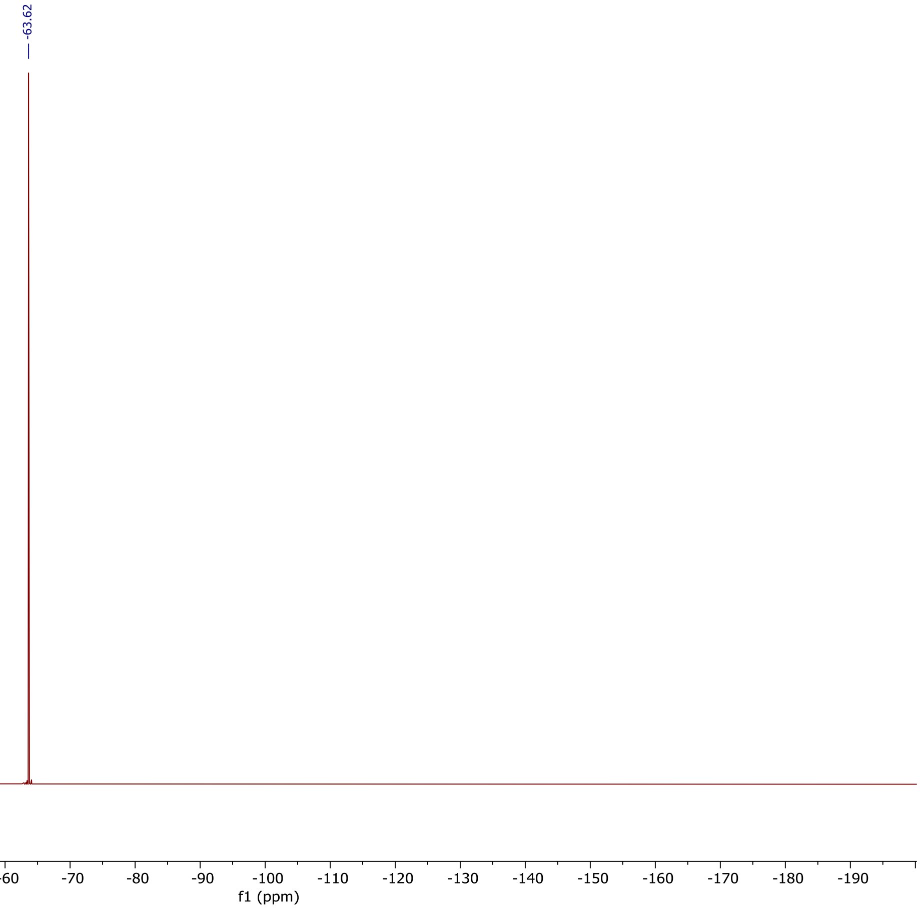

^{1}H NMR (400 MHz, $CDCl_3$) of **8** (*see procedure*)

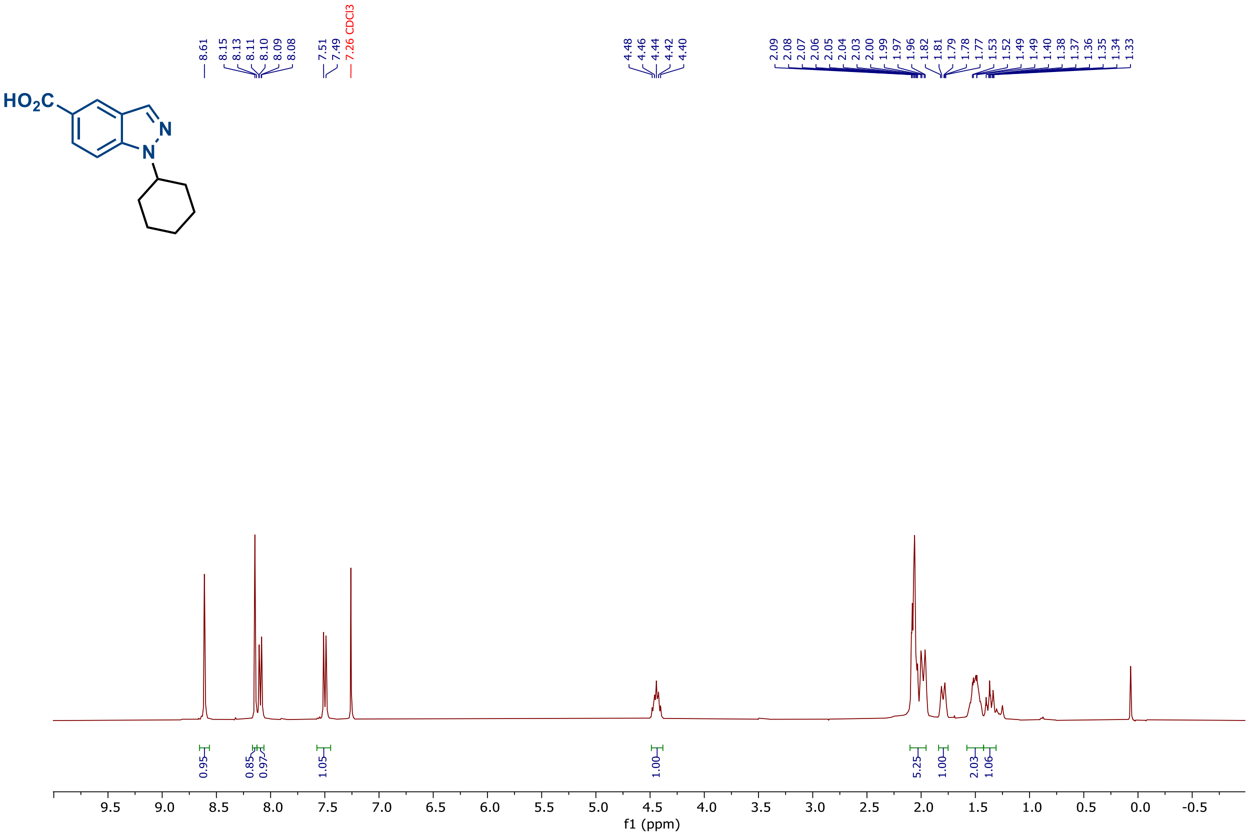

^{13}C NMR (101 MHz, $CDCl_3$) of **8**

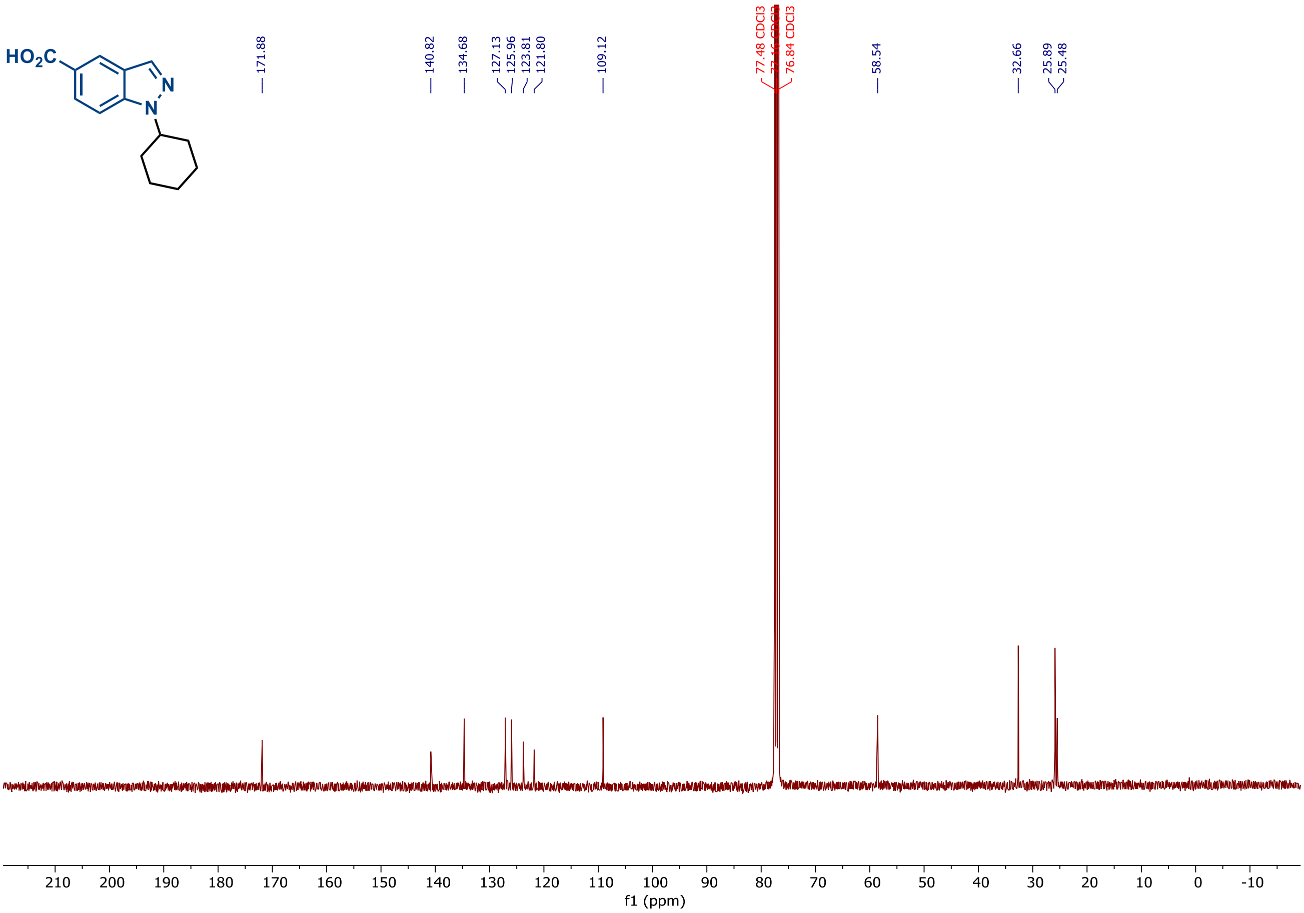

^{1}H NMR (400 MHz, $CDCl_3$) of **9** (see procedure)

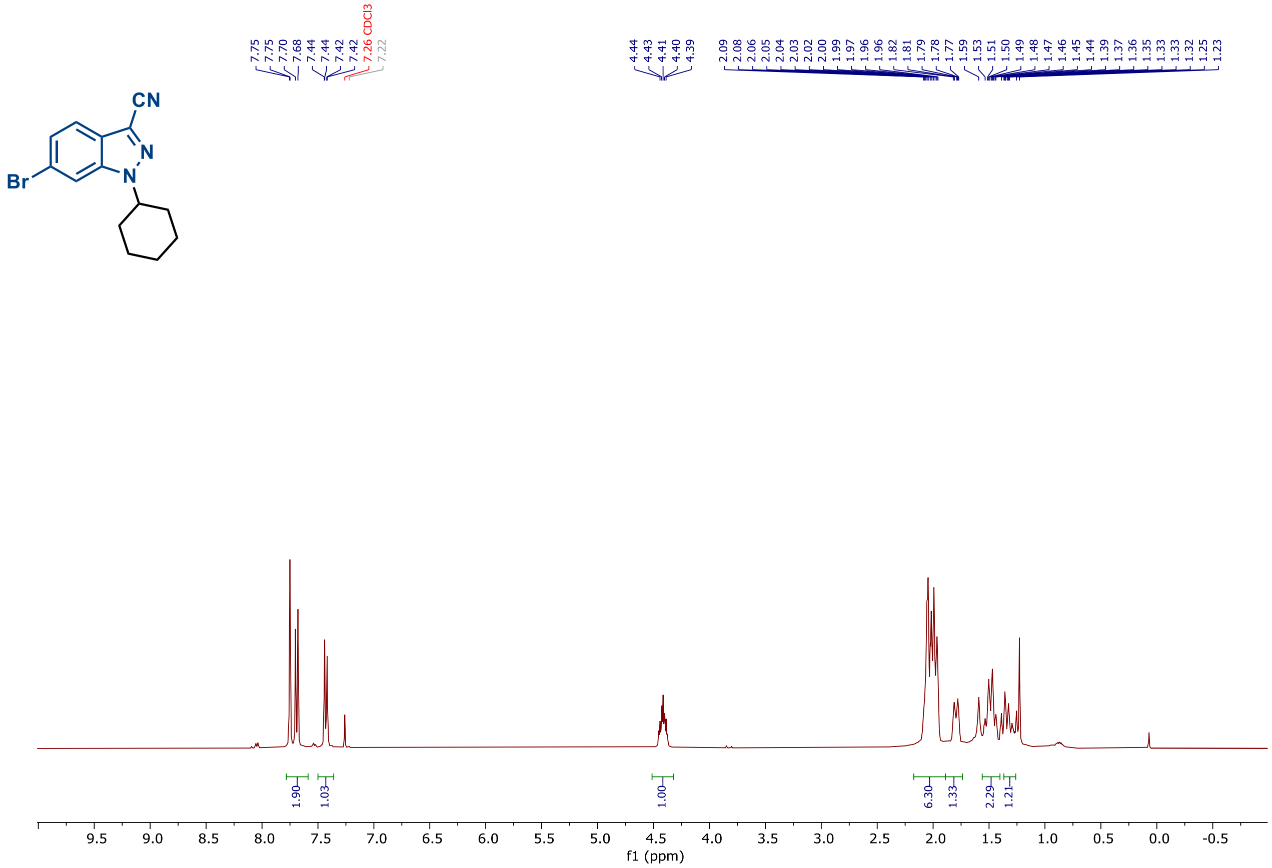

^{13}C NMR (101 MHz, $CDCl_3$) of **9**

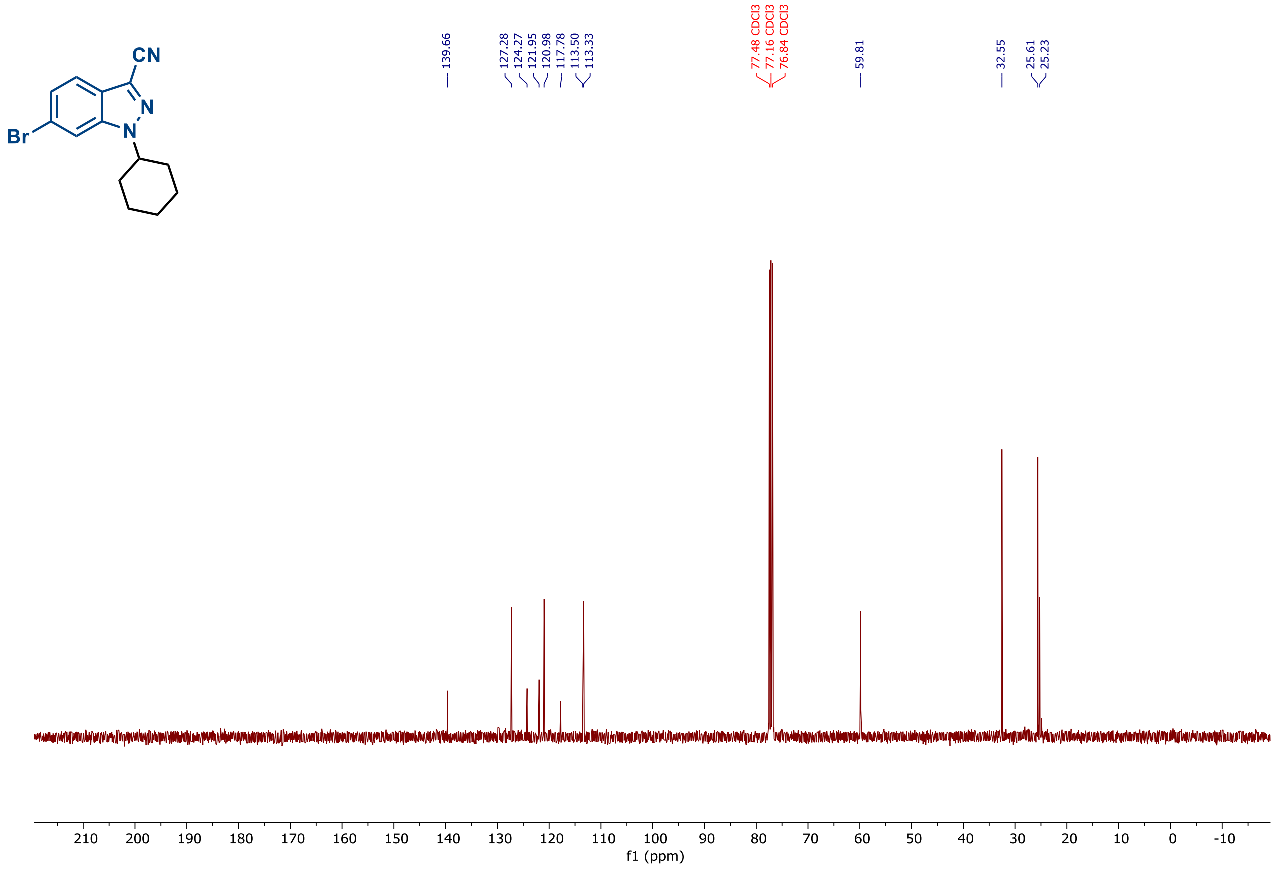

^{1}H NMR (400 MHz, $CDCl_3$) of **10** (*see procedure*)

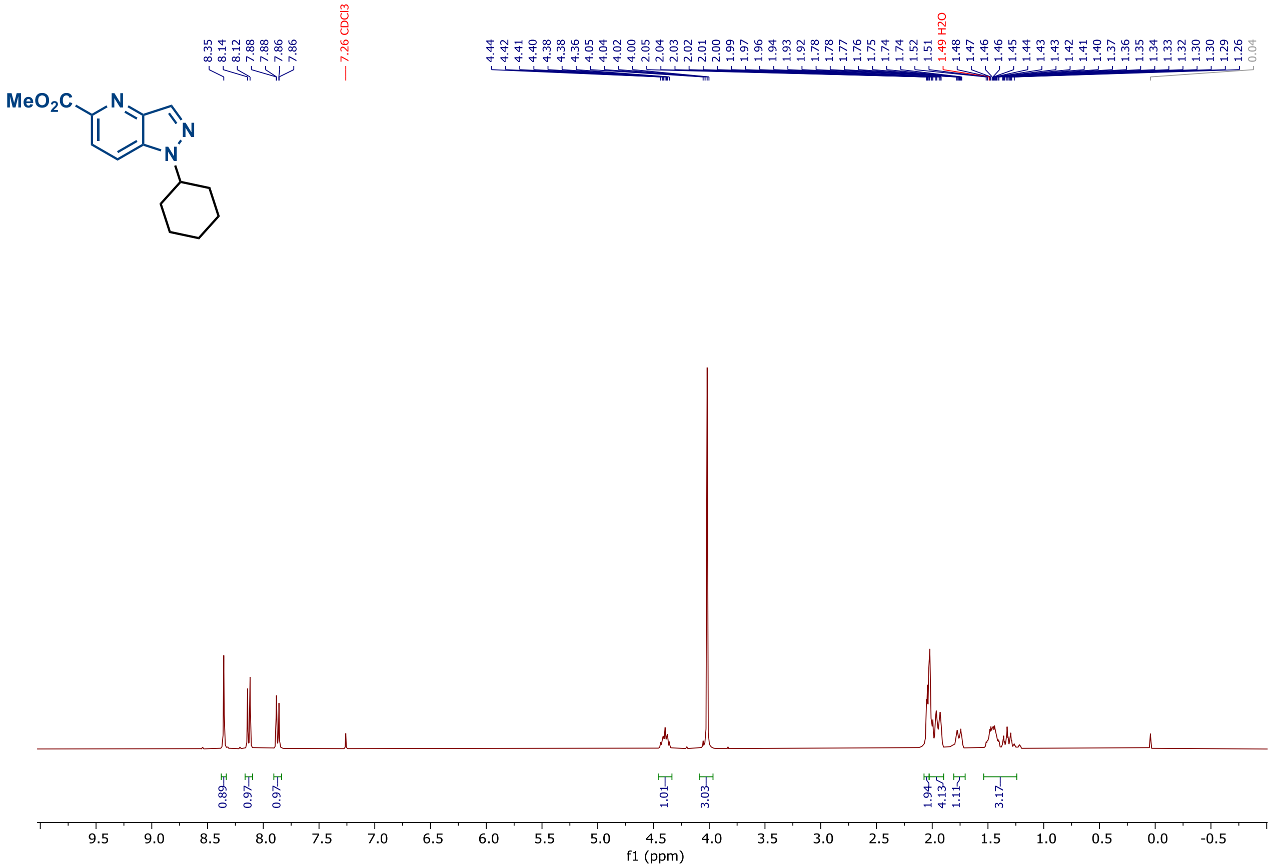

^{13}C NMR (101 MHz, $CDCl_3$) of **10**

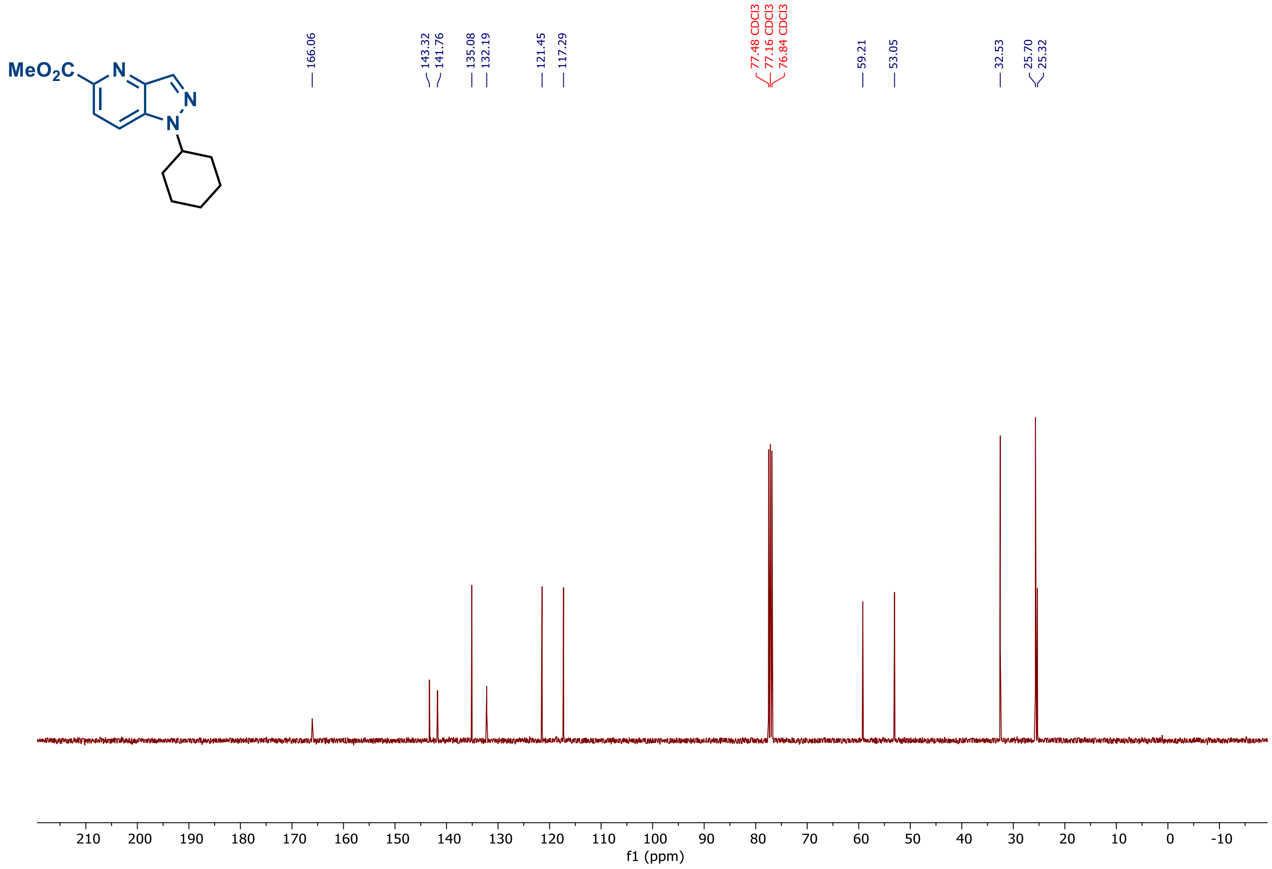

^{1}H NMR (400 MHz, $CDCl_3$) of **11** (*see procedure*)

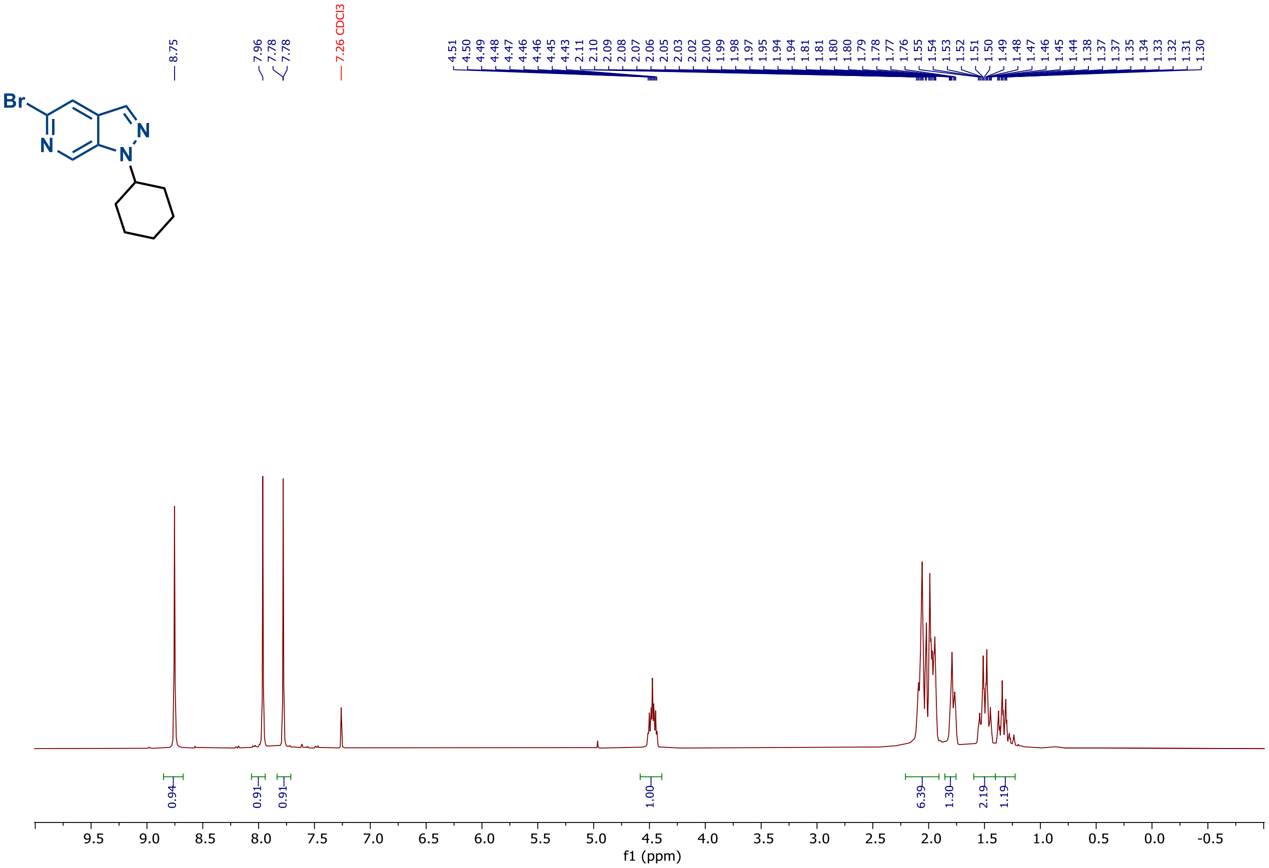

^{13}C NMR (101 MHz, $CDCl_3$) of **11**

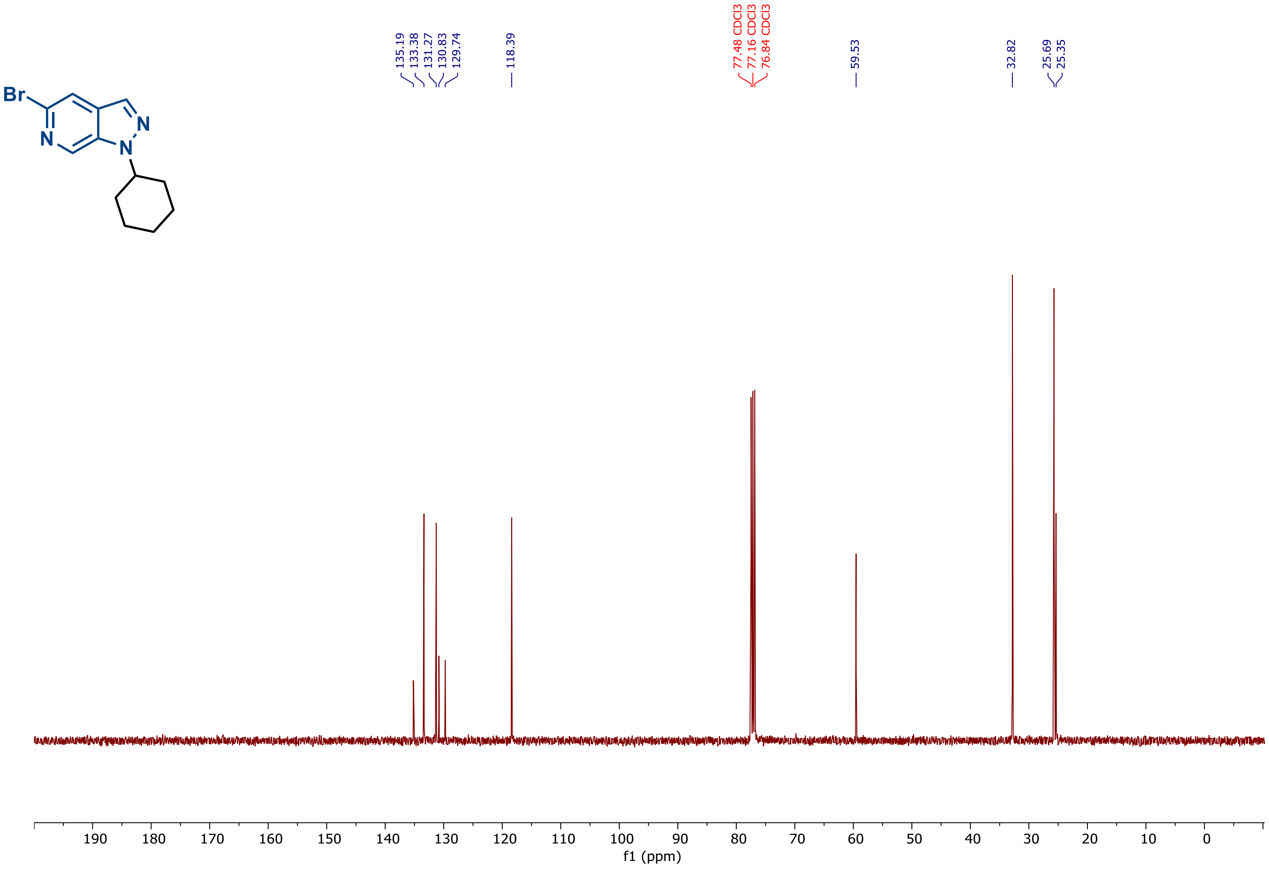

^{1}H NMR (400 MHz, $CDCl_3$) of **12** (*see procedure*)

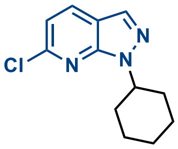

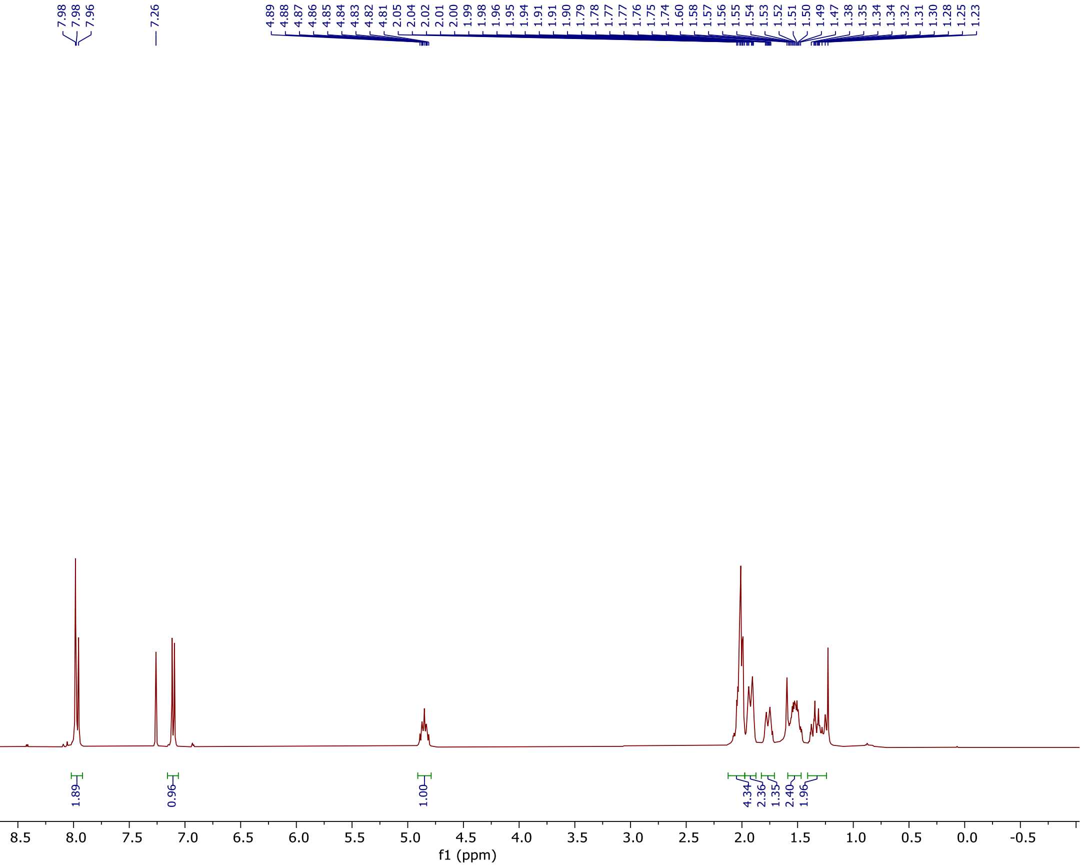

^{13}C NMR (101 MHz, $CDCl_3$) of **12**

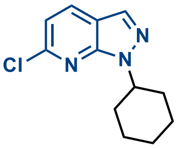

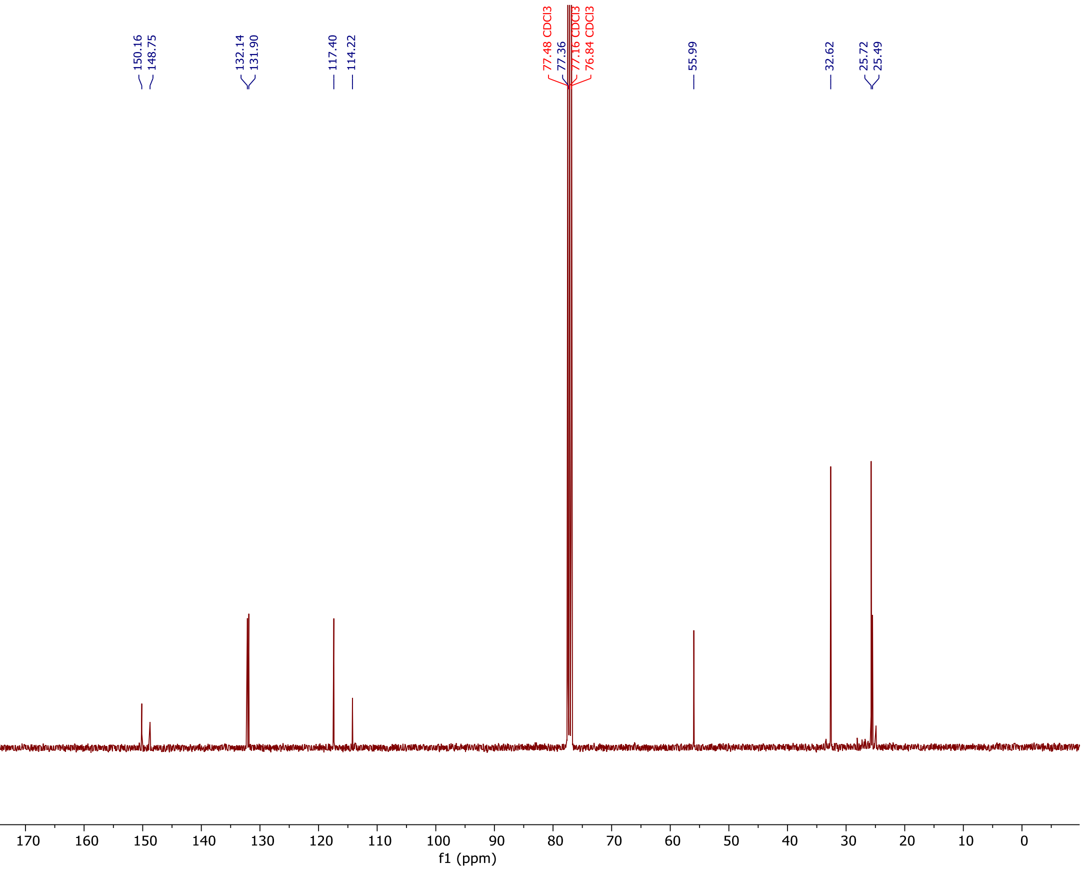

^{1}H NMR (400 MHz, $CDCl_3$) of **13** (*see procedure*)

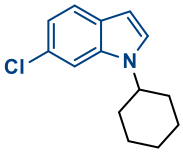

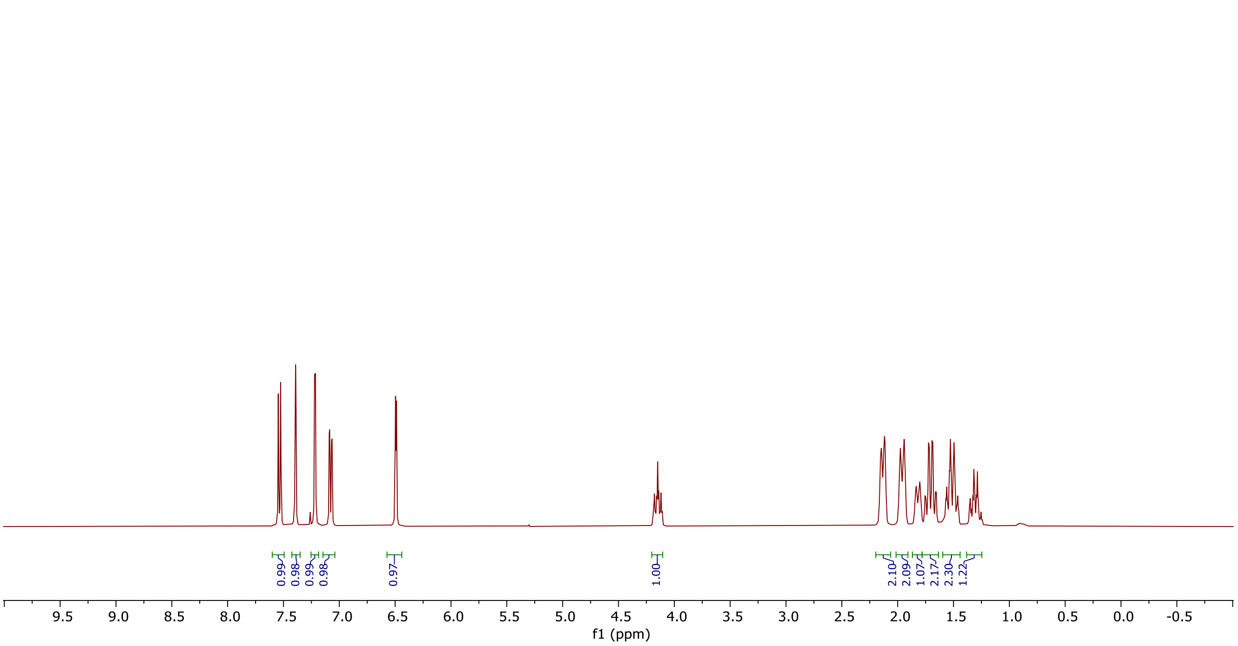

^{13}C NMR (101 MHz, $CDCl_3$) of **13**

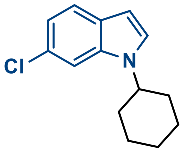

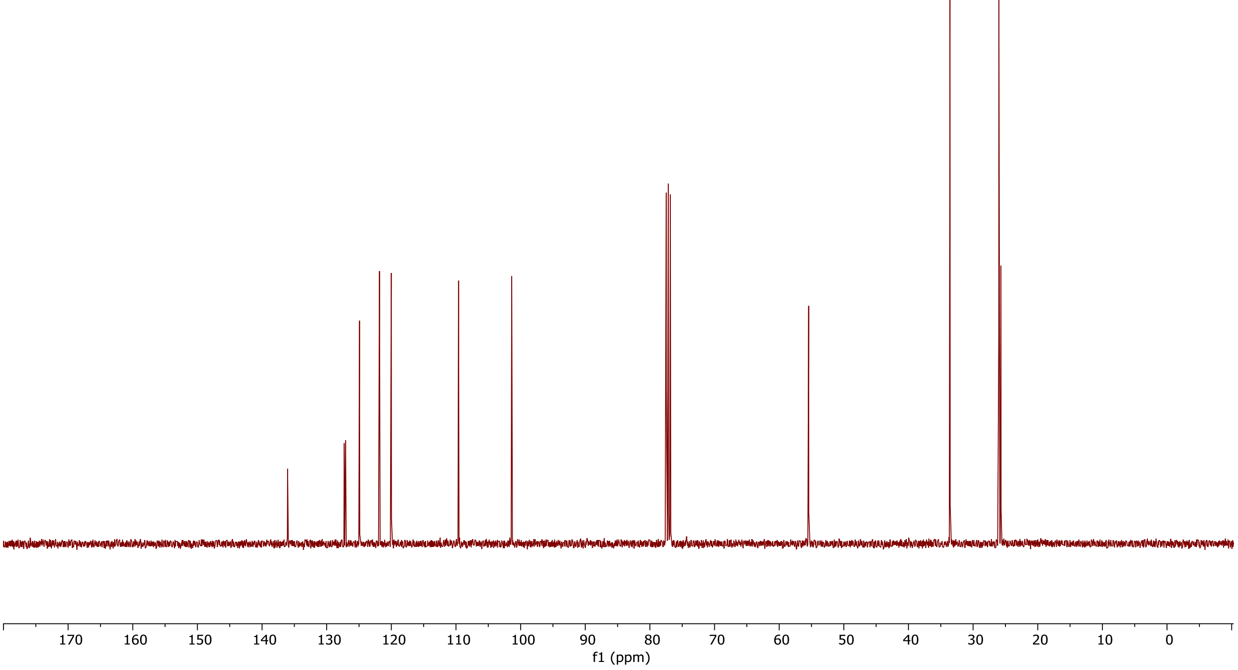

^{1}H NMR (400 MHz, $CDCl_3$) of **14** (*see procedure*)

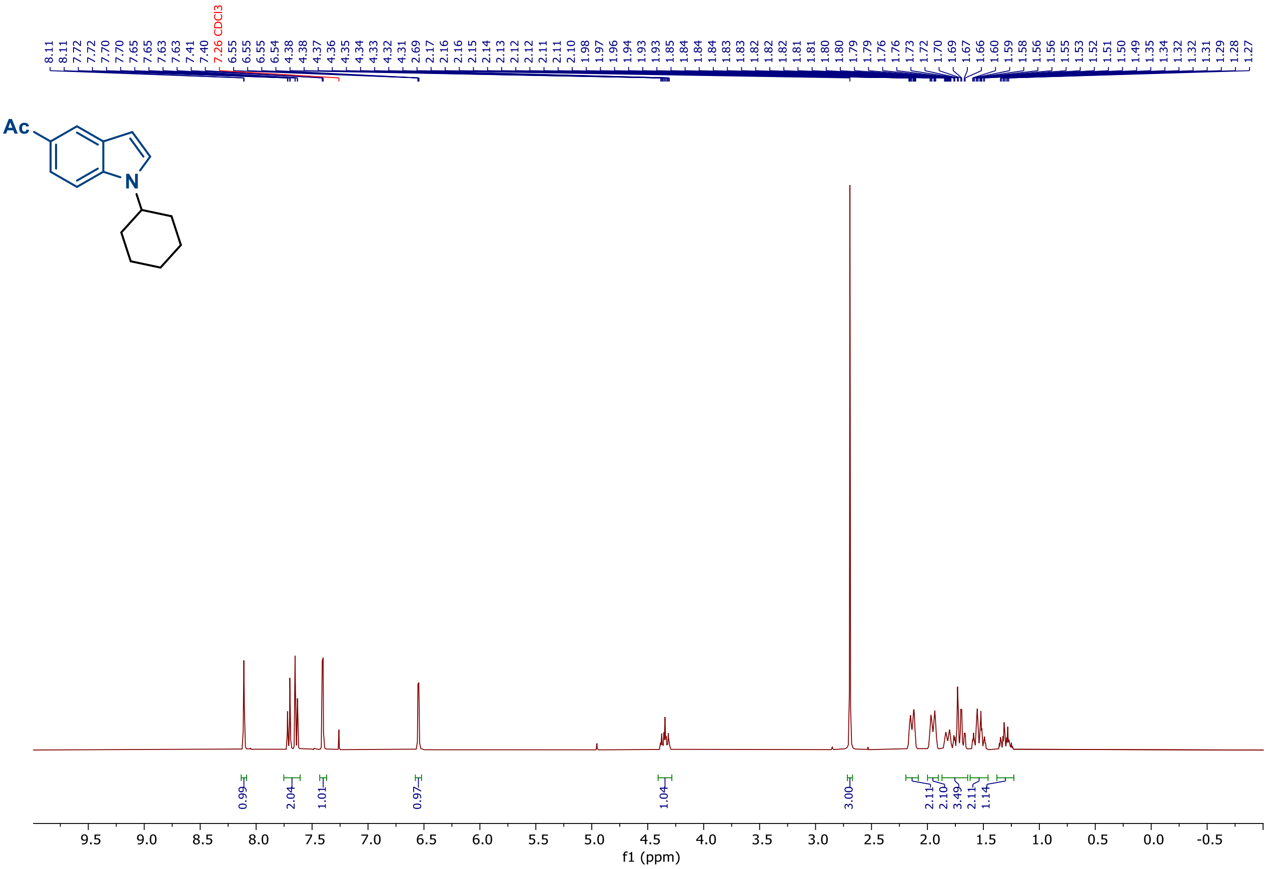

^{13}C NMR (101 MHz, $CDCl_3$) of **14**

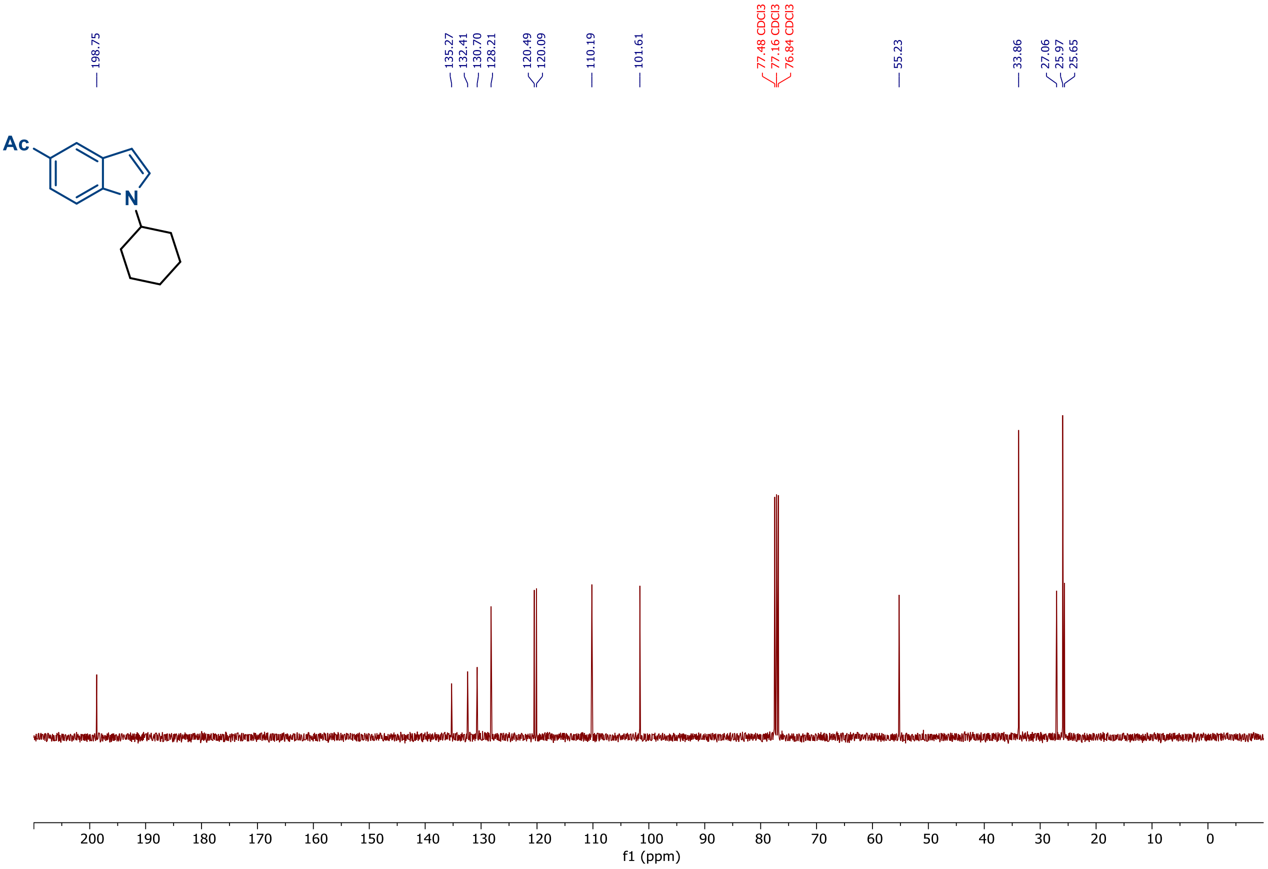

^{1}H NMR (400 MHz, $CDCl_3$) of **15** (*see procedure*)

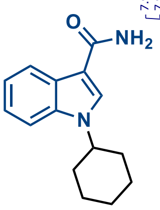

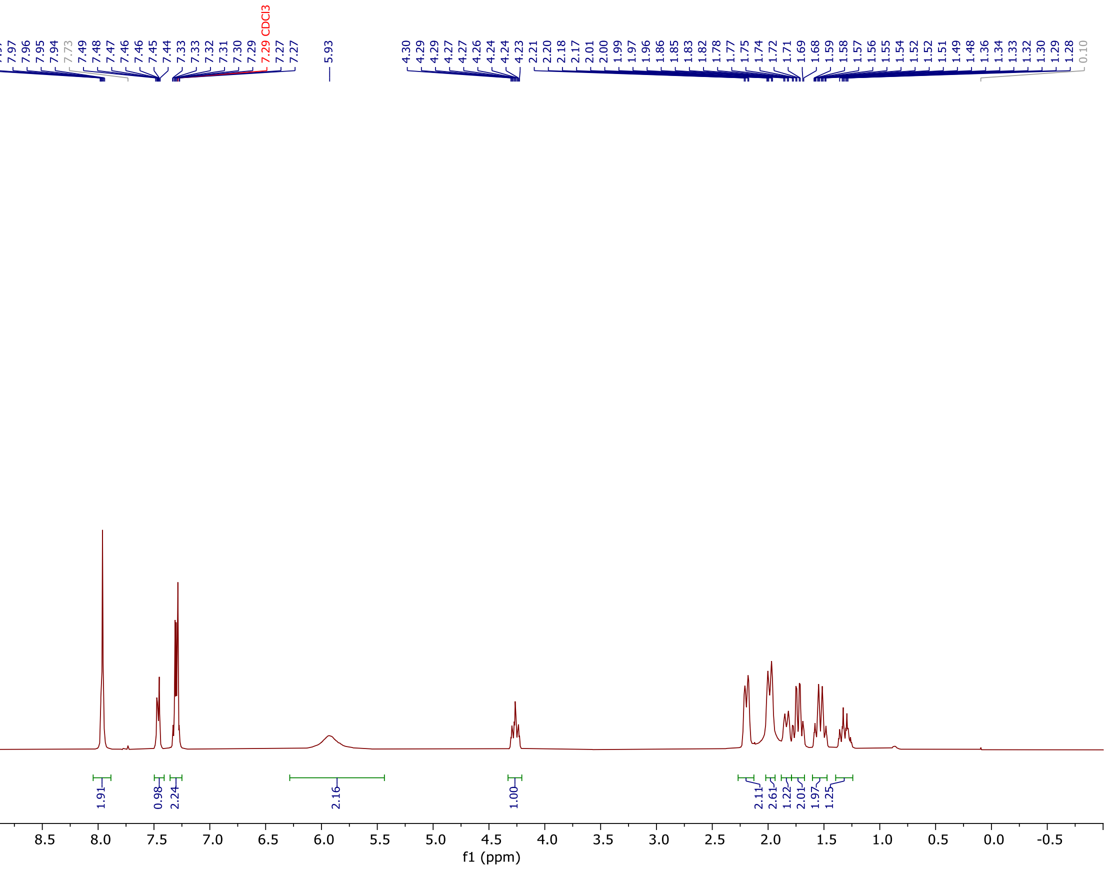

^{13}C NMR (101 MHz, $CDCl_3$) of **15**

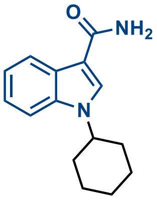

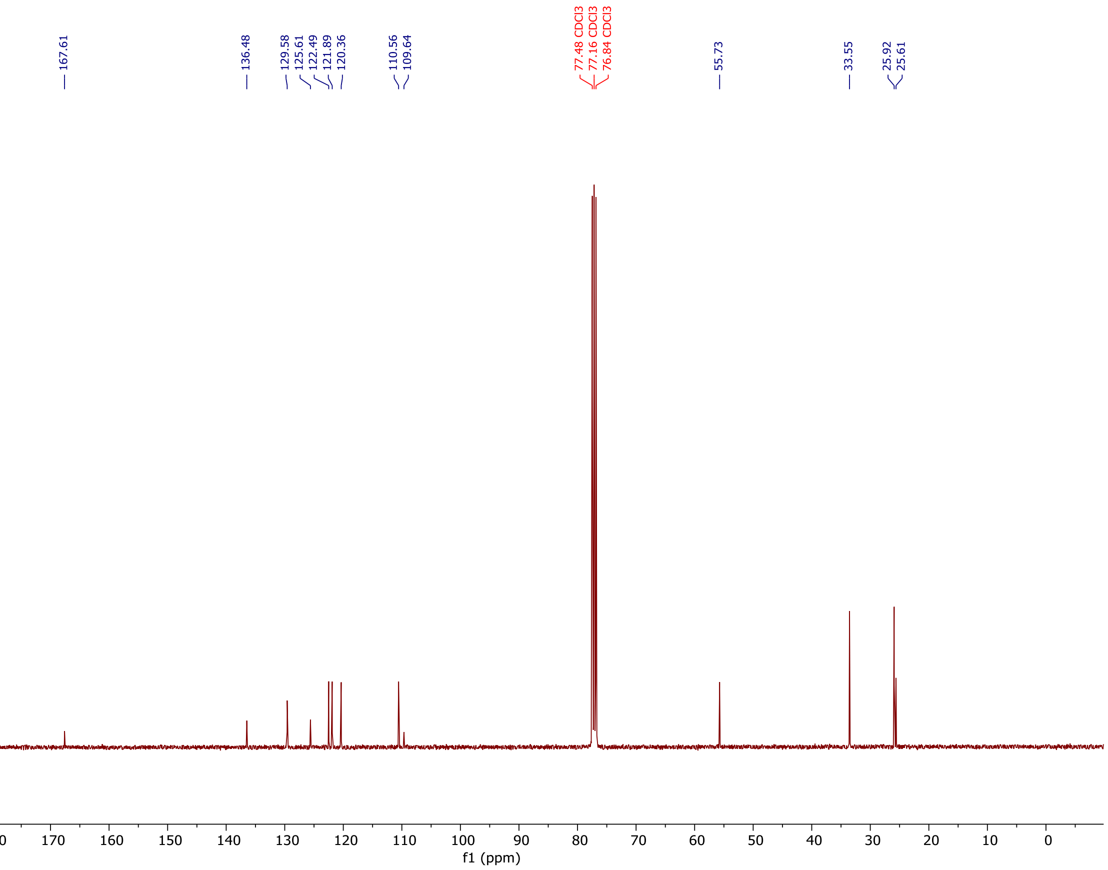

^{1}H NMR (400 MHz, $CDCl_3$) of **16** (*see procedure*)

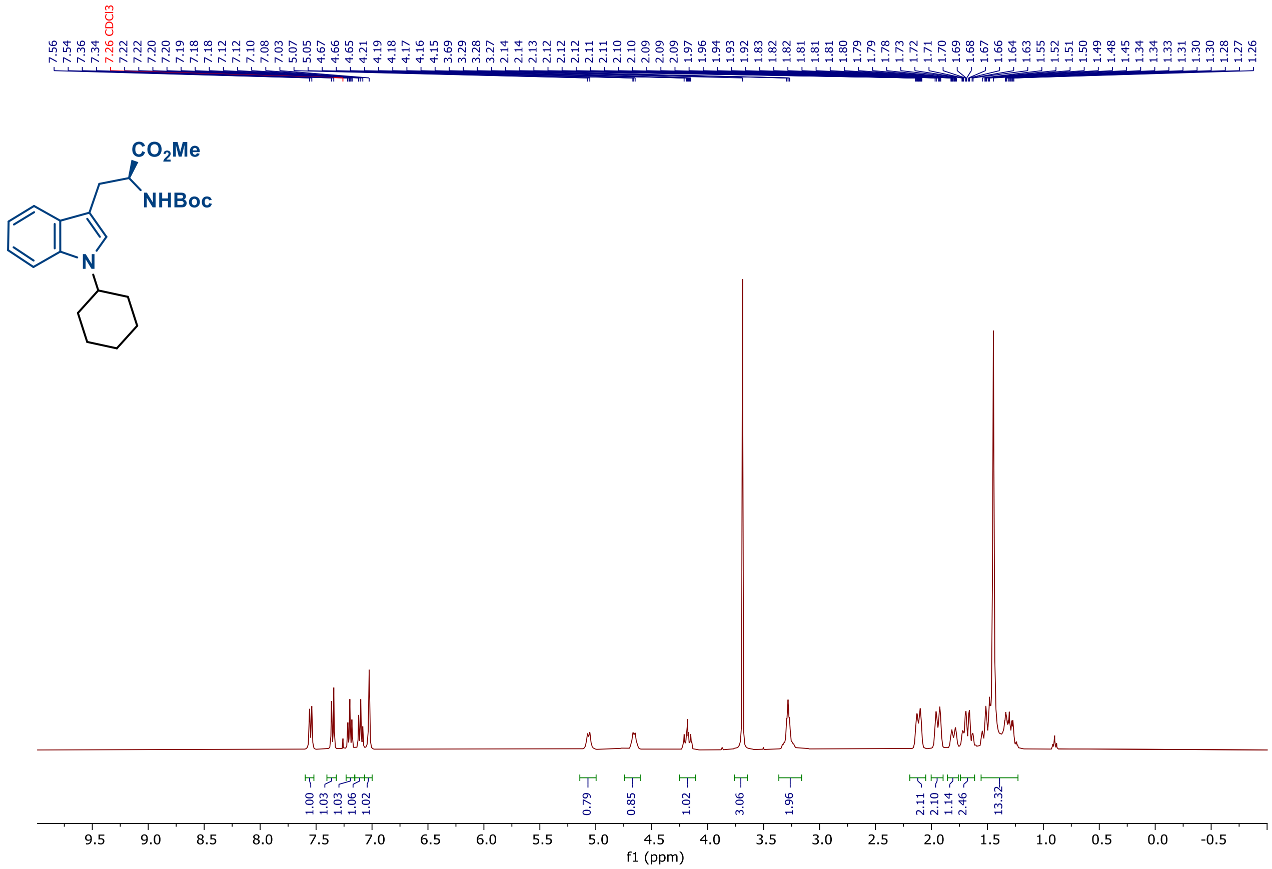

^{13}C NMR (101 MHz, $CDCl_3$) of **16**

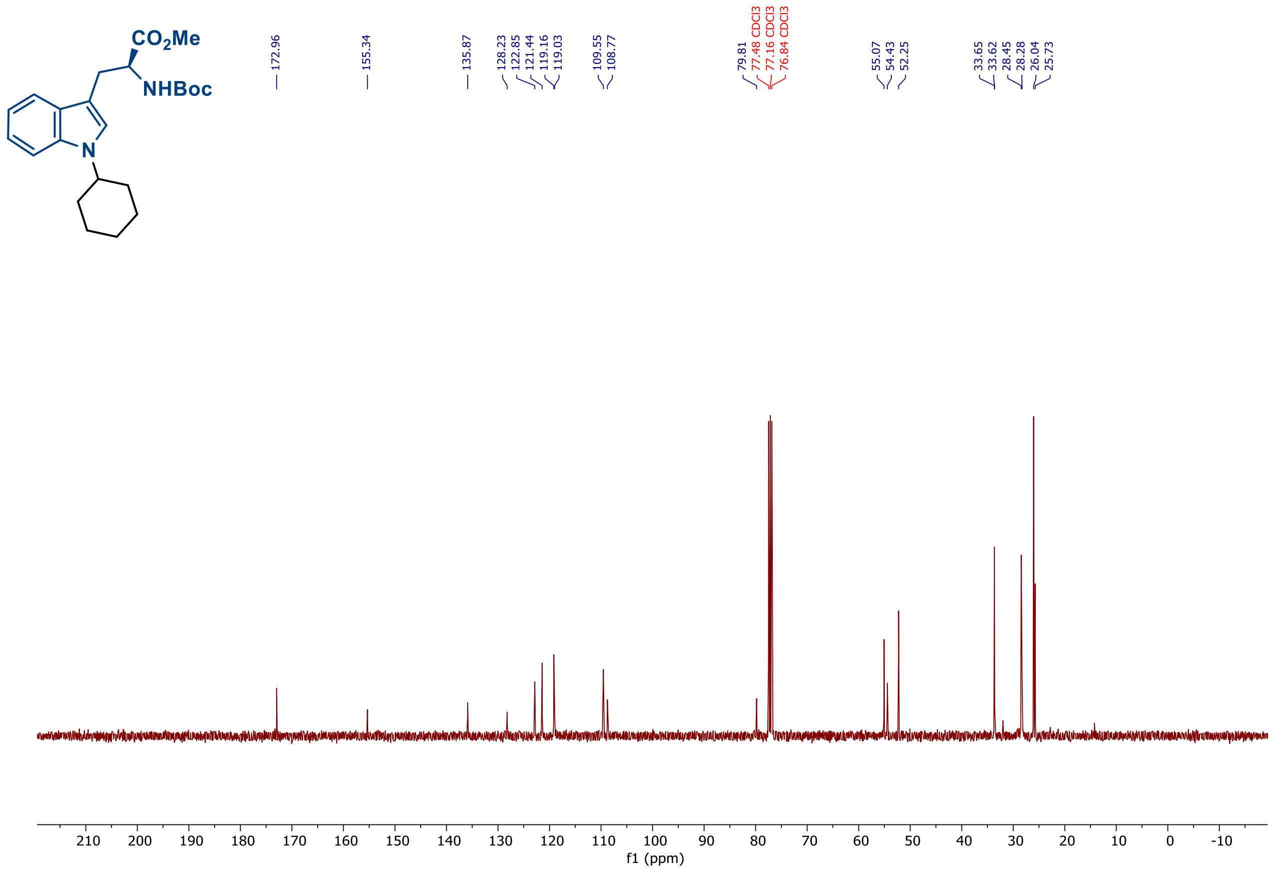

^1H NMR (400 MHz, $CDCl_3$) of **17** (see procedure)

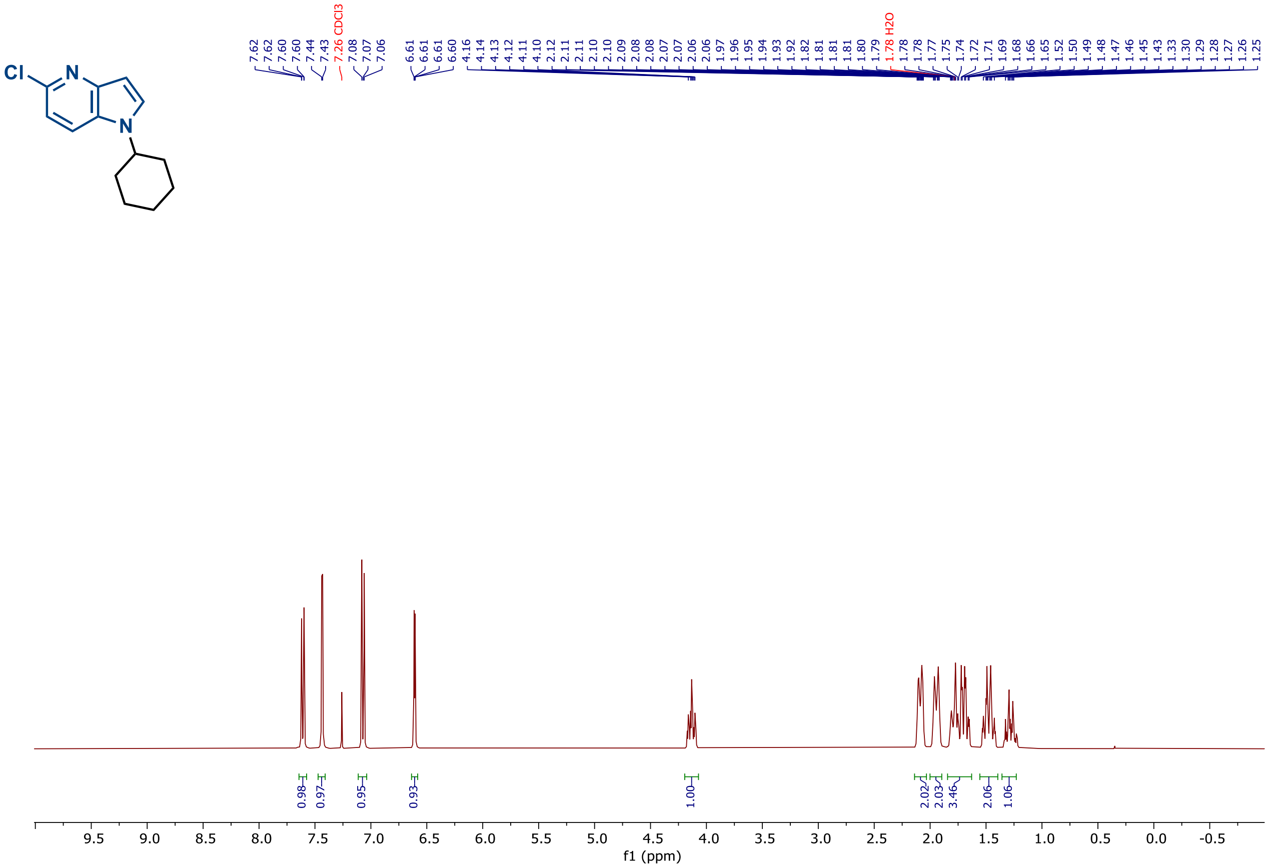

^{13}C NMR (101 MHz, $CDCl_3$) of **17**

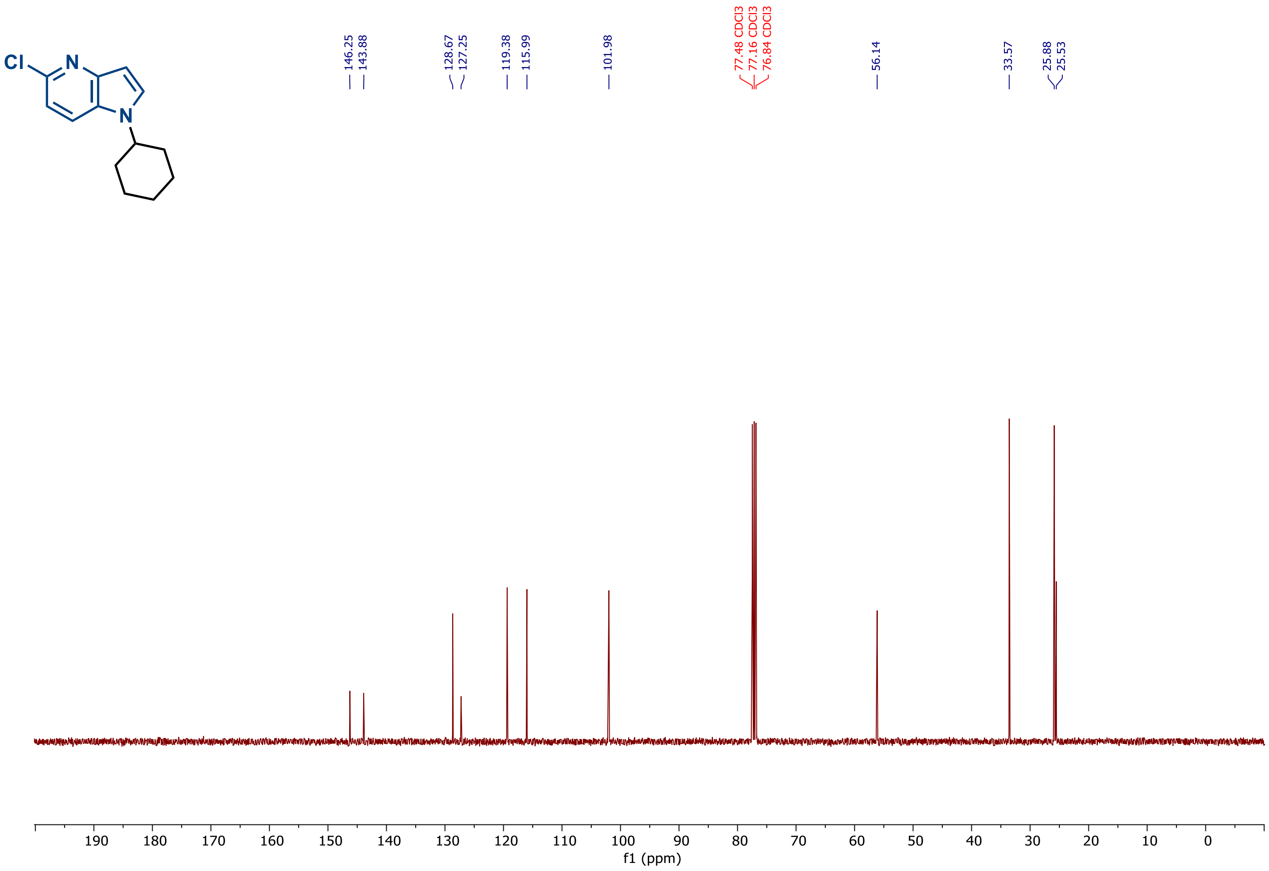

^{1}H NMR (400 MHz, $CDCl_3$) of **18** (*see procedure*)

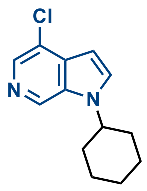

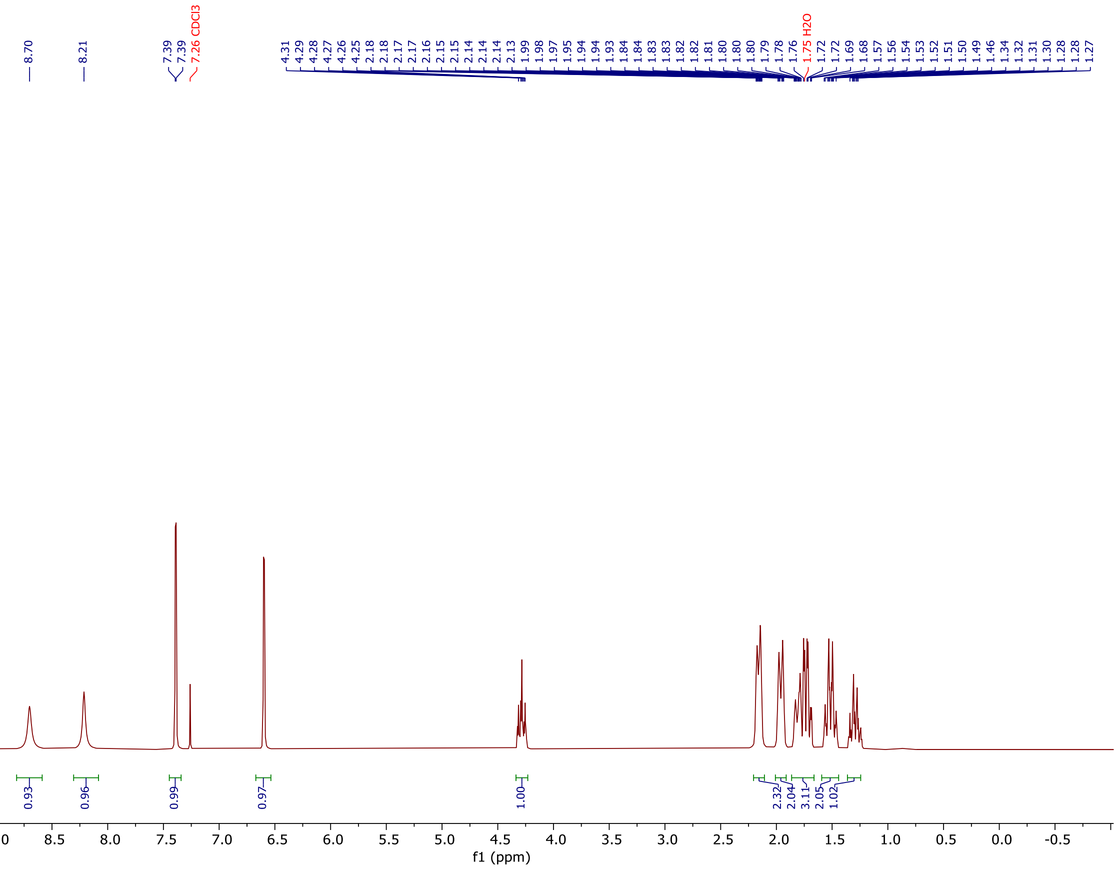

^{13}C NMR (101 MHz, $CDCl_3$) of **18**

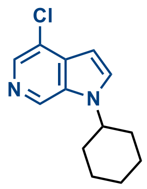

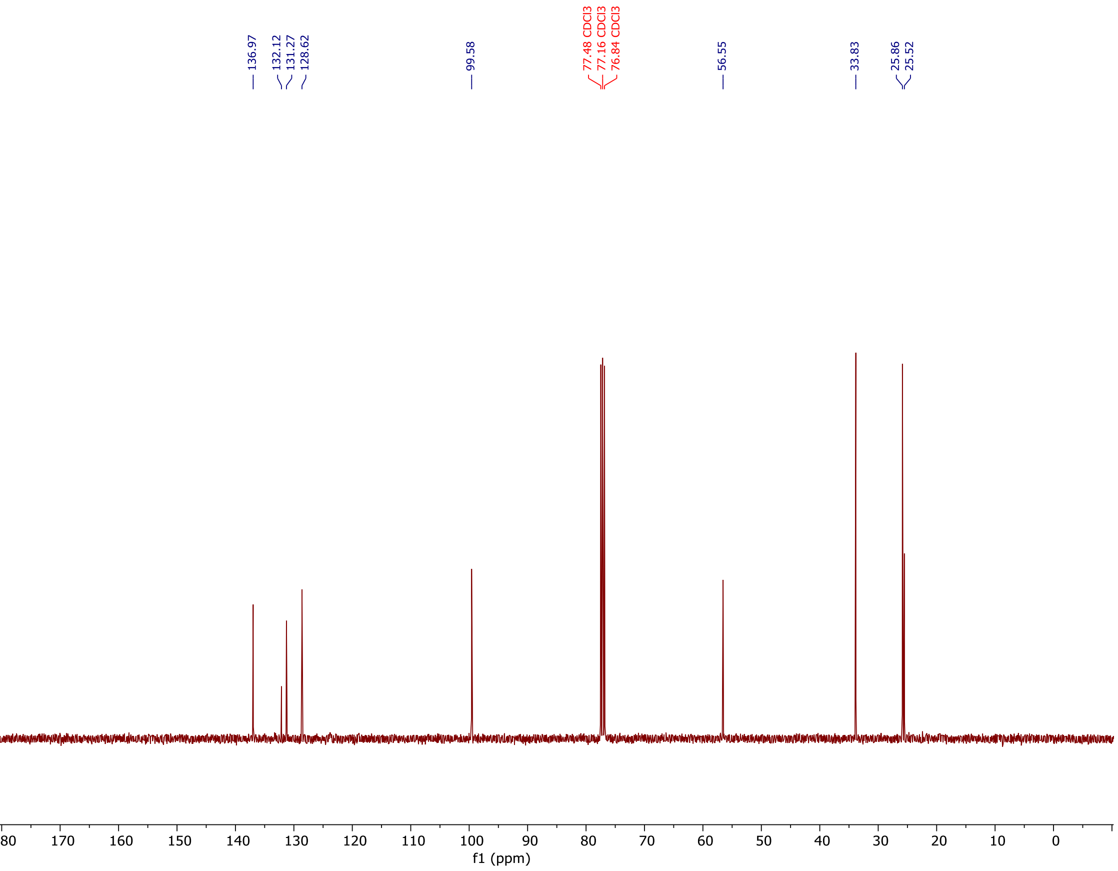

^{1}H NMR (400 MHz, $CDCl_3$) of **19** (<u>*see procedure*</u>)

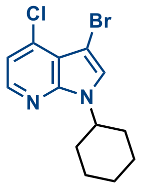

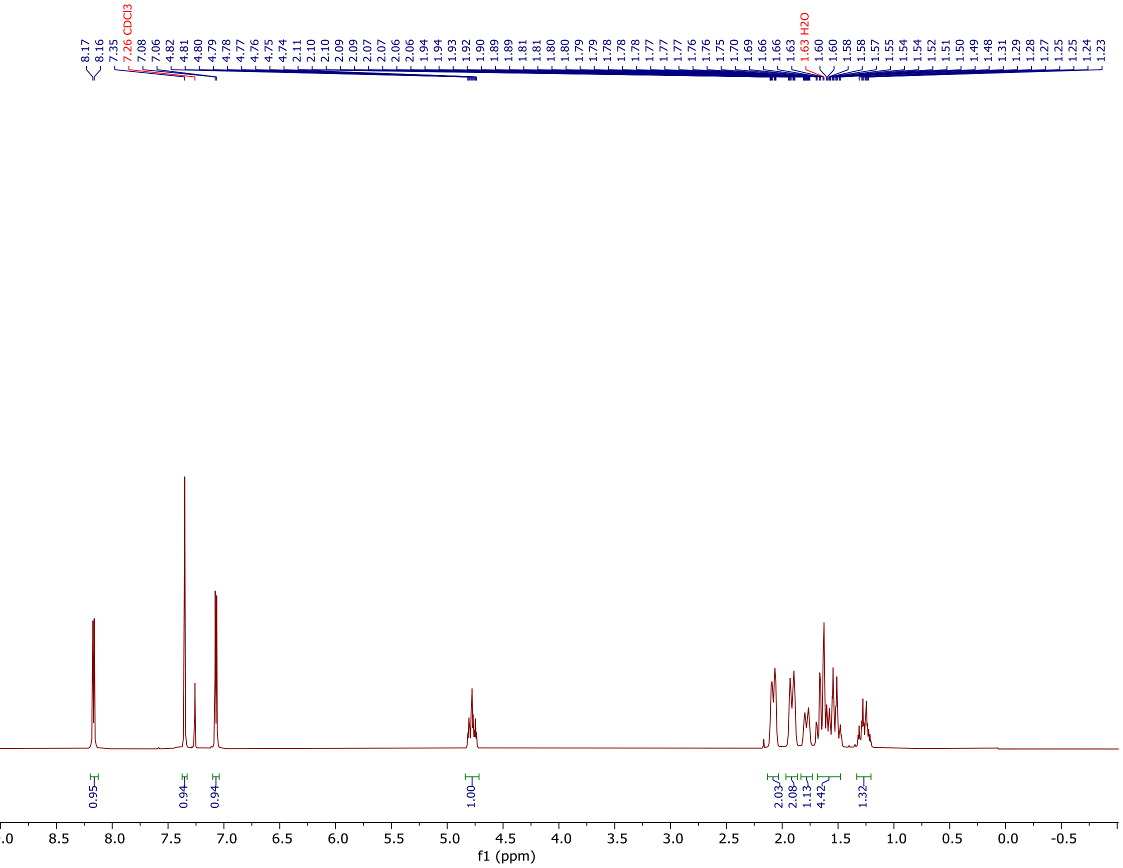

^{13}C NMR (101 MHz, $CDCl_3$) of **19**

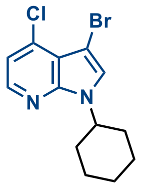

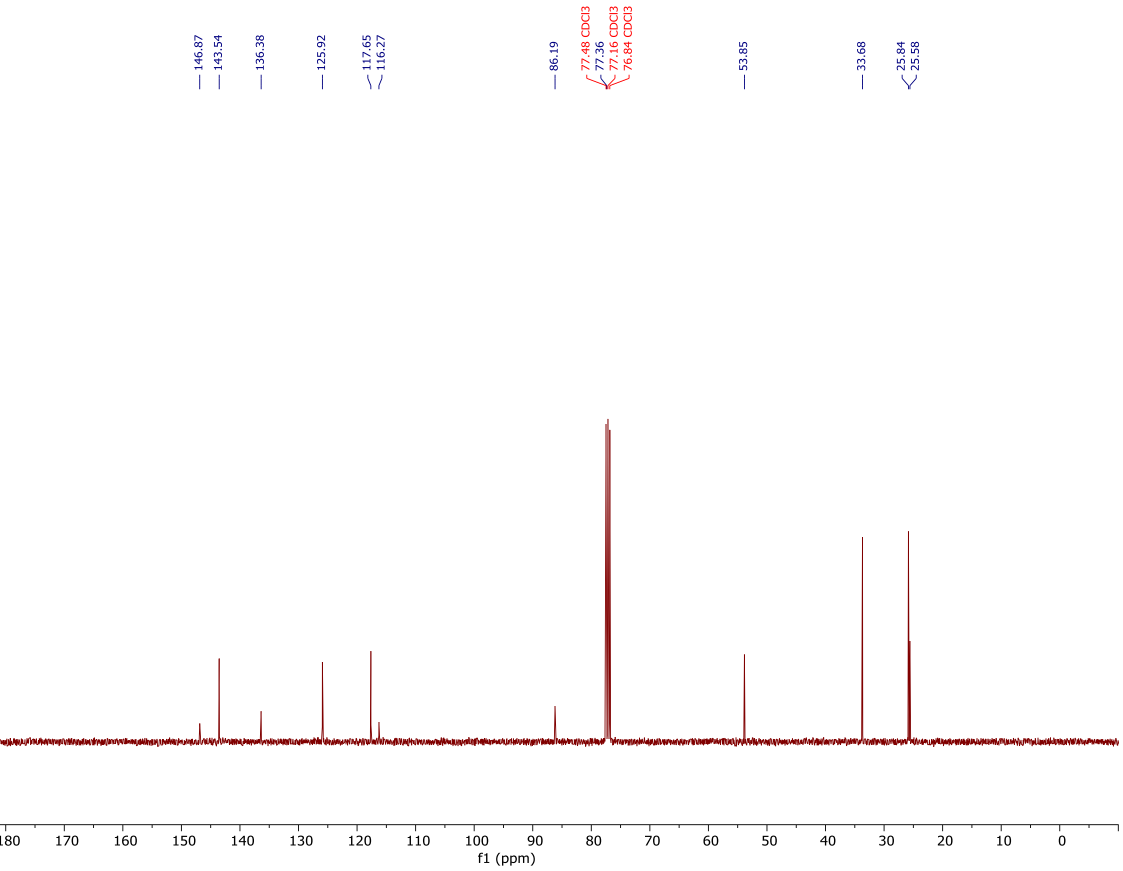

^{1}H NMR (400 MHz, $CDCl_3$) of **20** (see procedure)

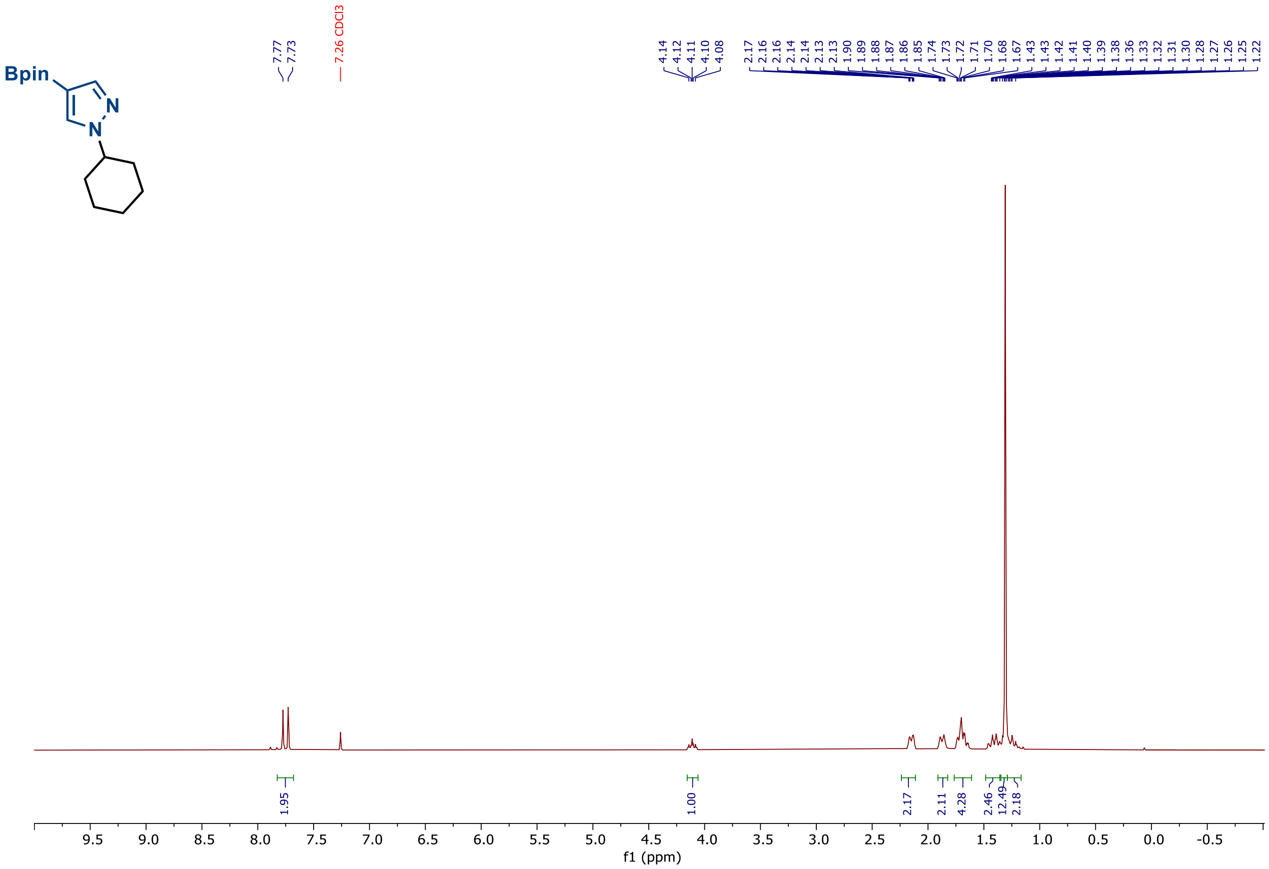

^{13}C NMR (101 MHz, $CDCl_3$) of **20**

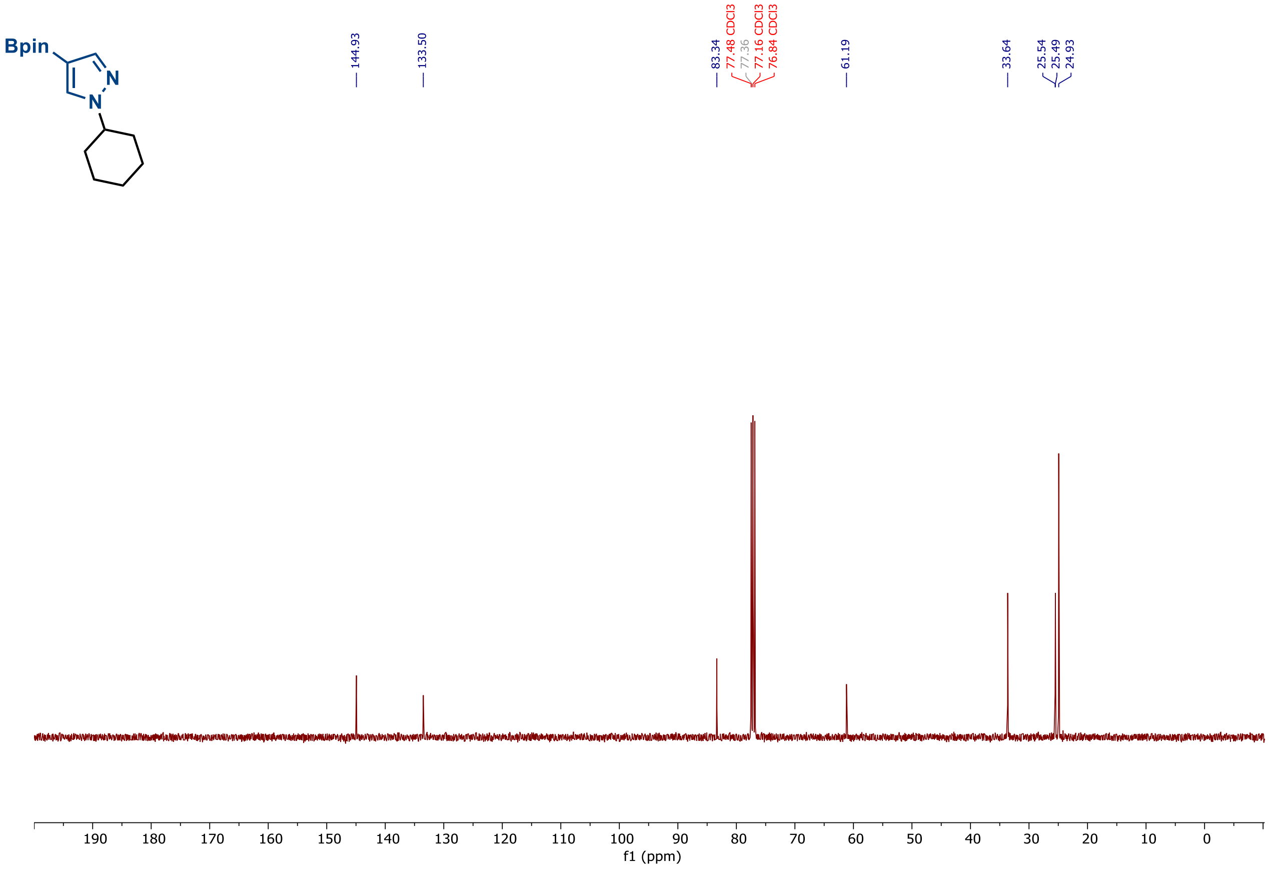

^{11}B NMR (128 MHz, $CDCl_3$) of **20**

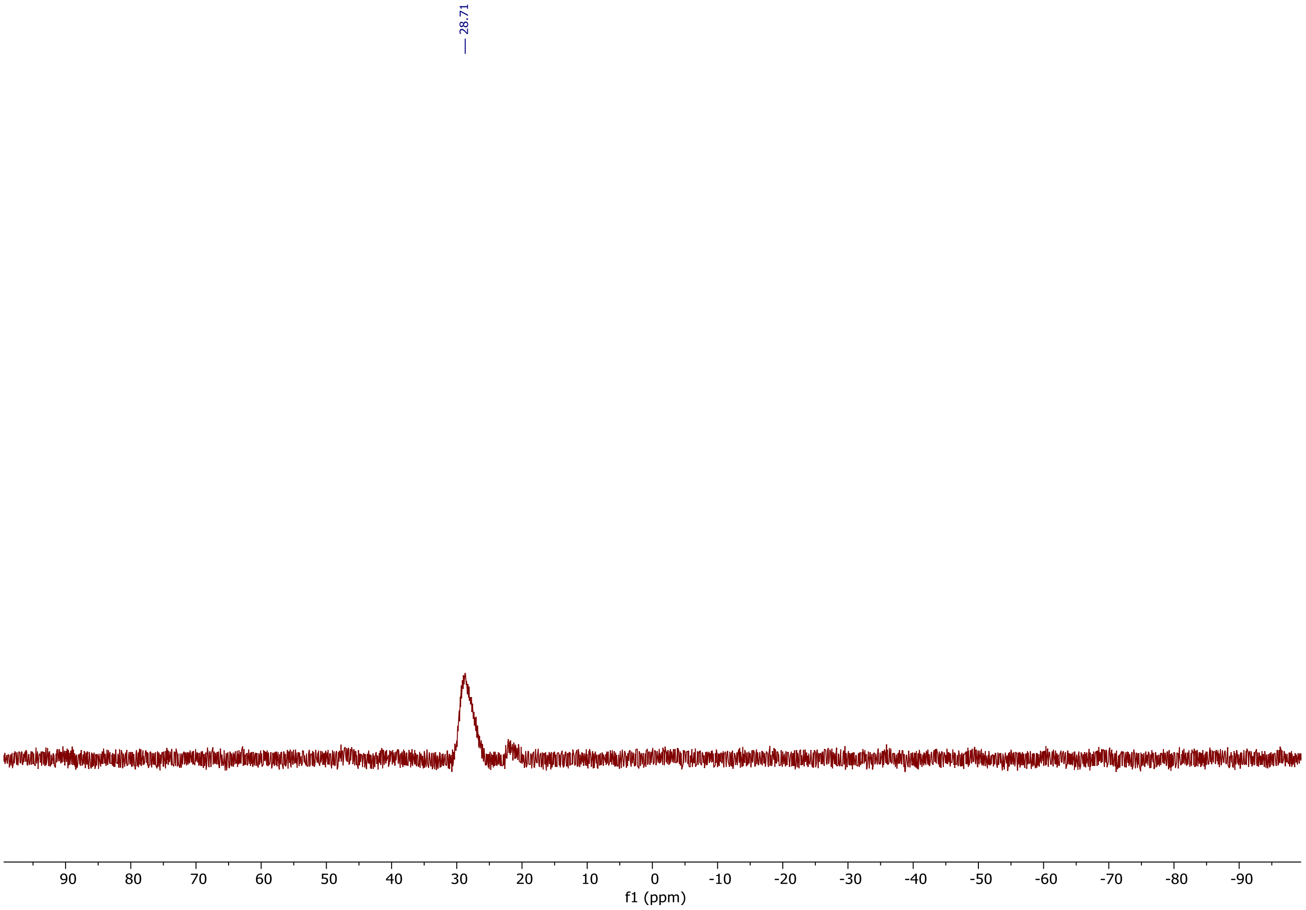

[1] H NMR (400 MHz, $CDCl_3$) of **21** (*see procedure*)

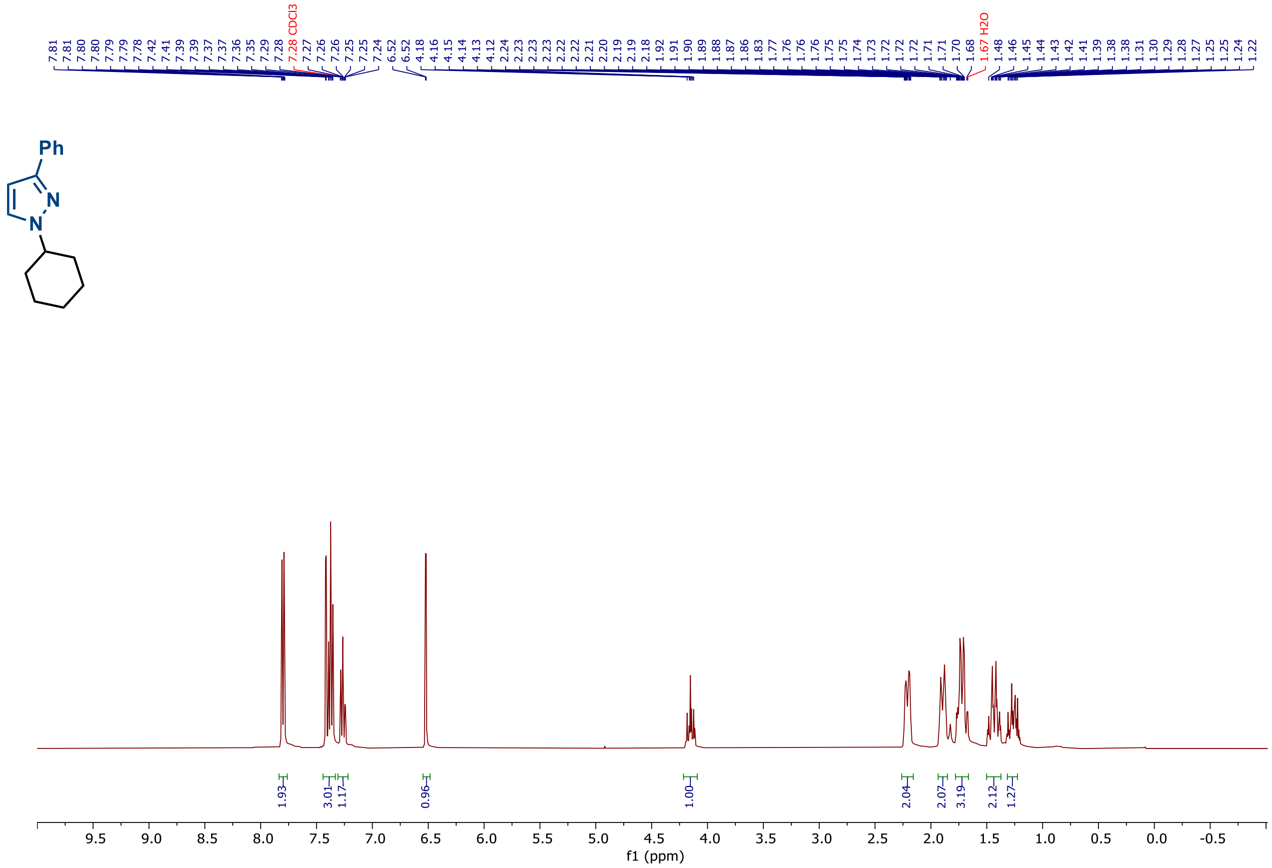

[13] C NMR (101 MHz, $CDCl_3$) of **21**

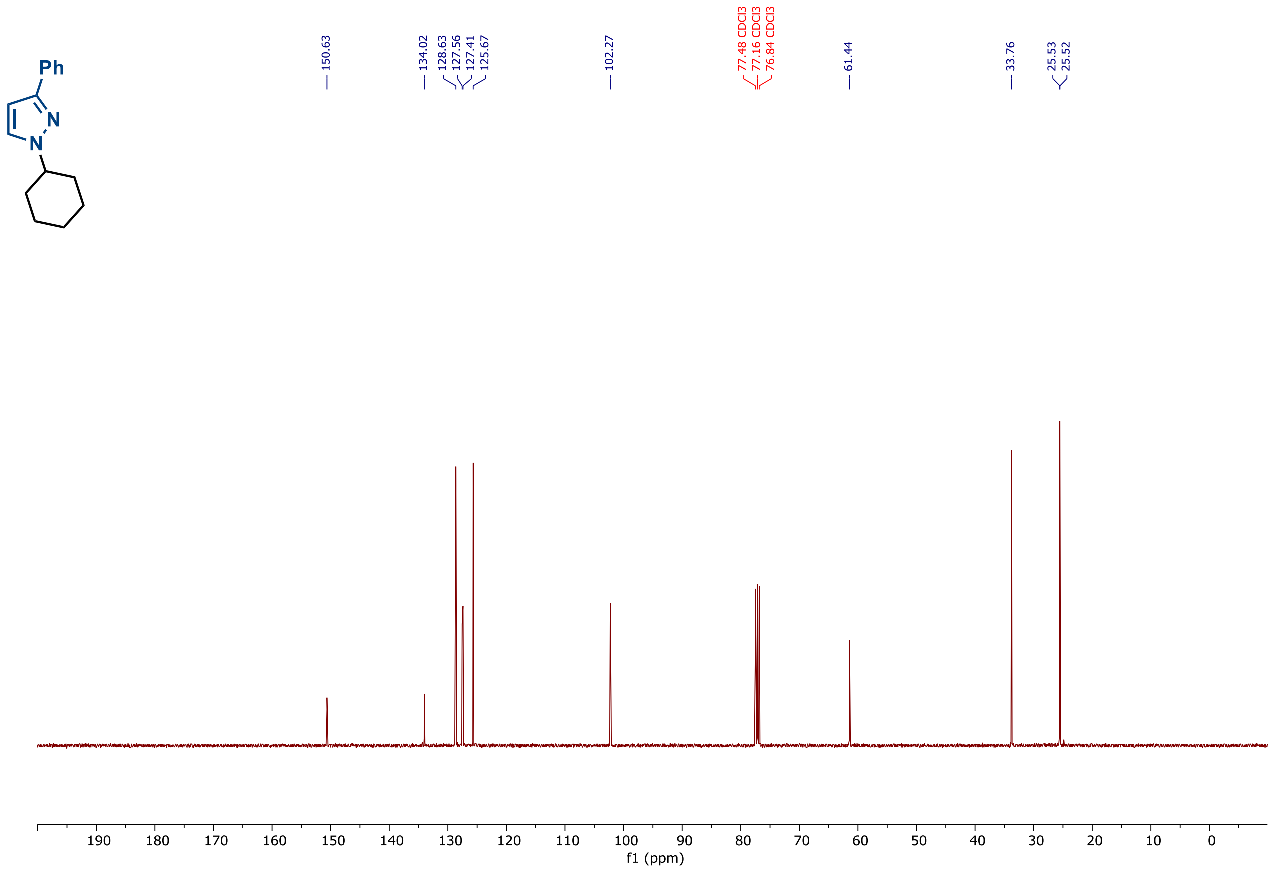

^{1}H NMR (400 MHz, $CDCl_3$) of **22** (*see procedure*)

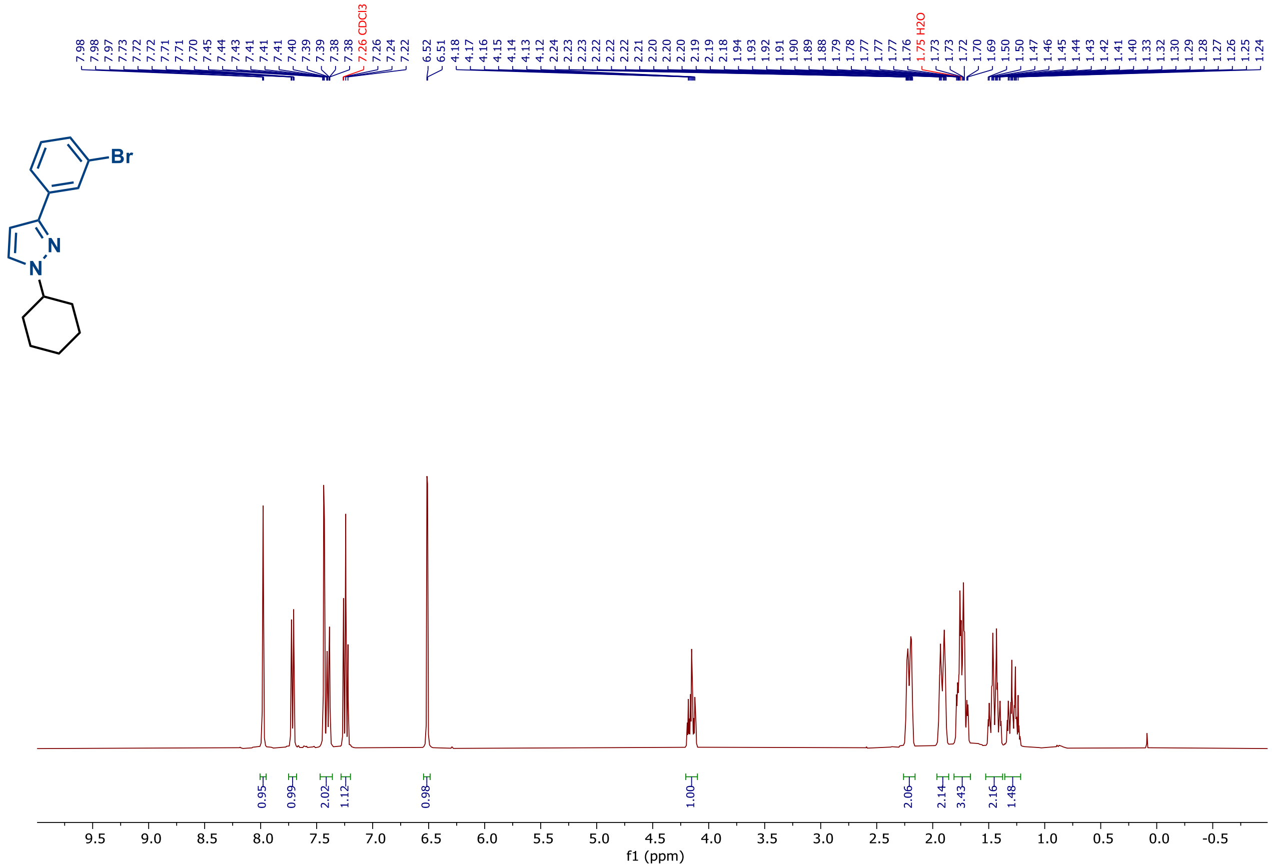

^{13}C NMR (101 MHz, $CDCl_3$) of **22**

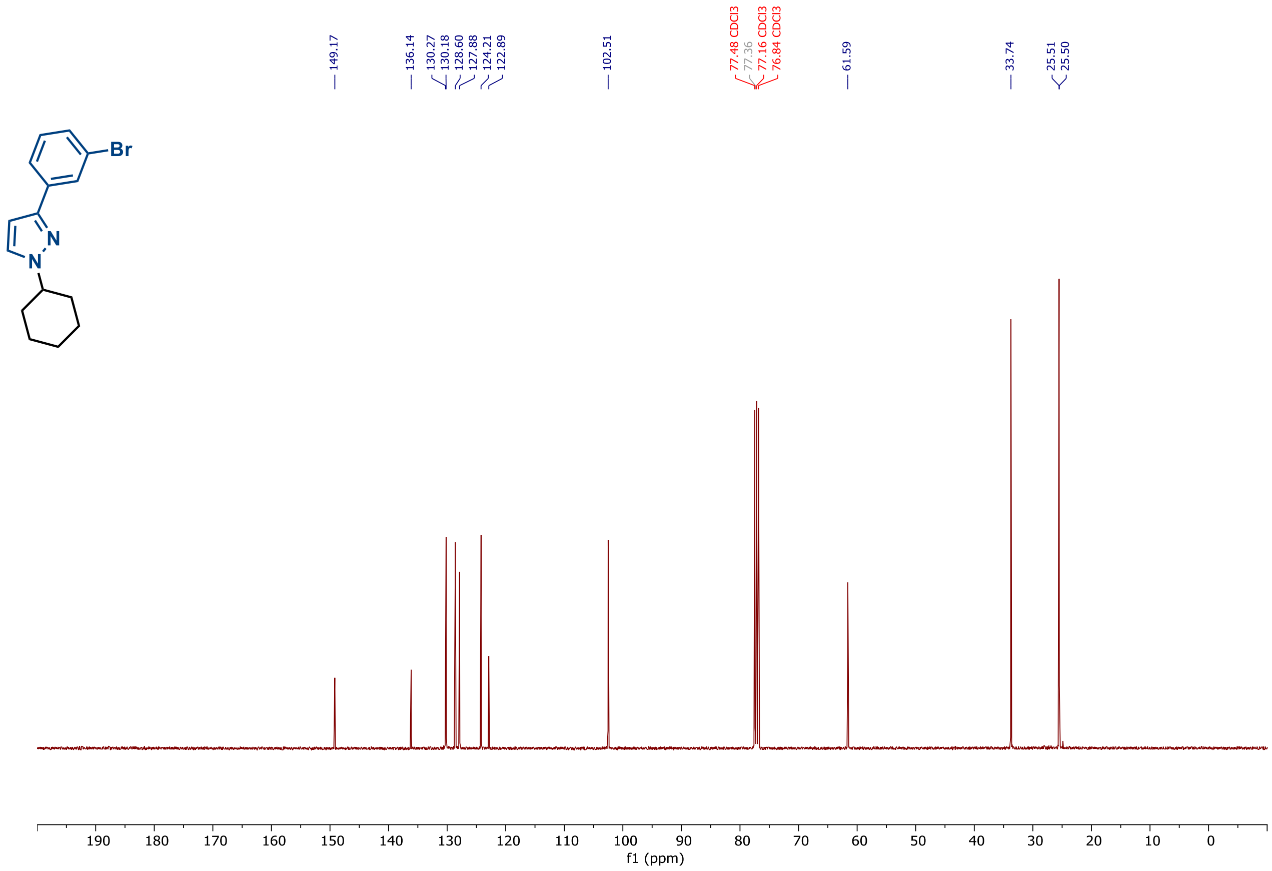

^{1}H NMR (400 MHz, $CDCl_3$) of **23** (see procedure)

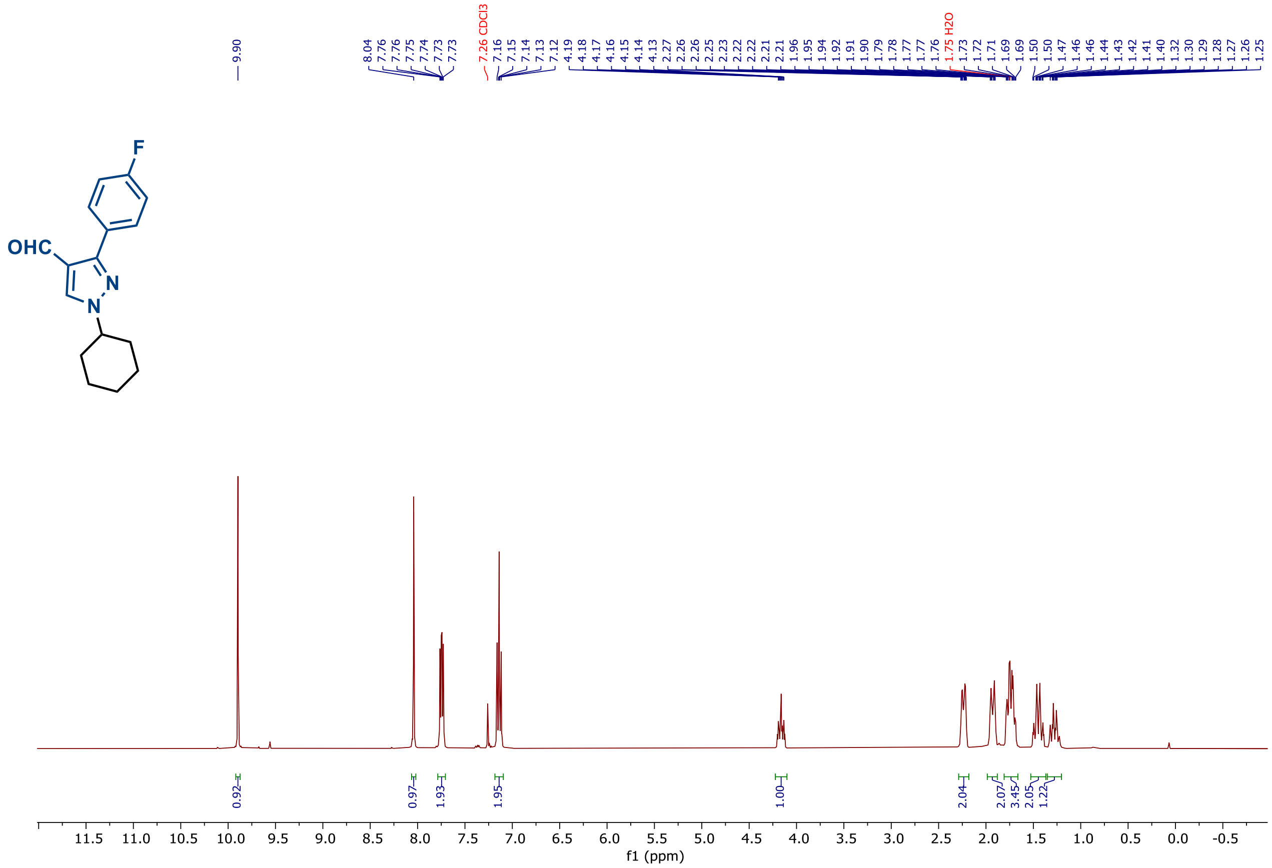

^{13}C NMR (101 MHz, $CDCl_3$) of **23**

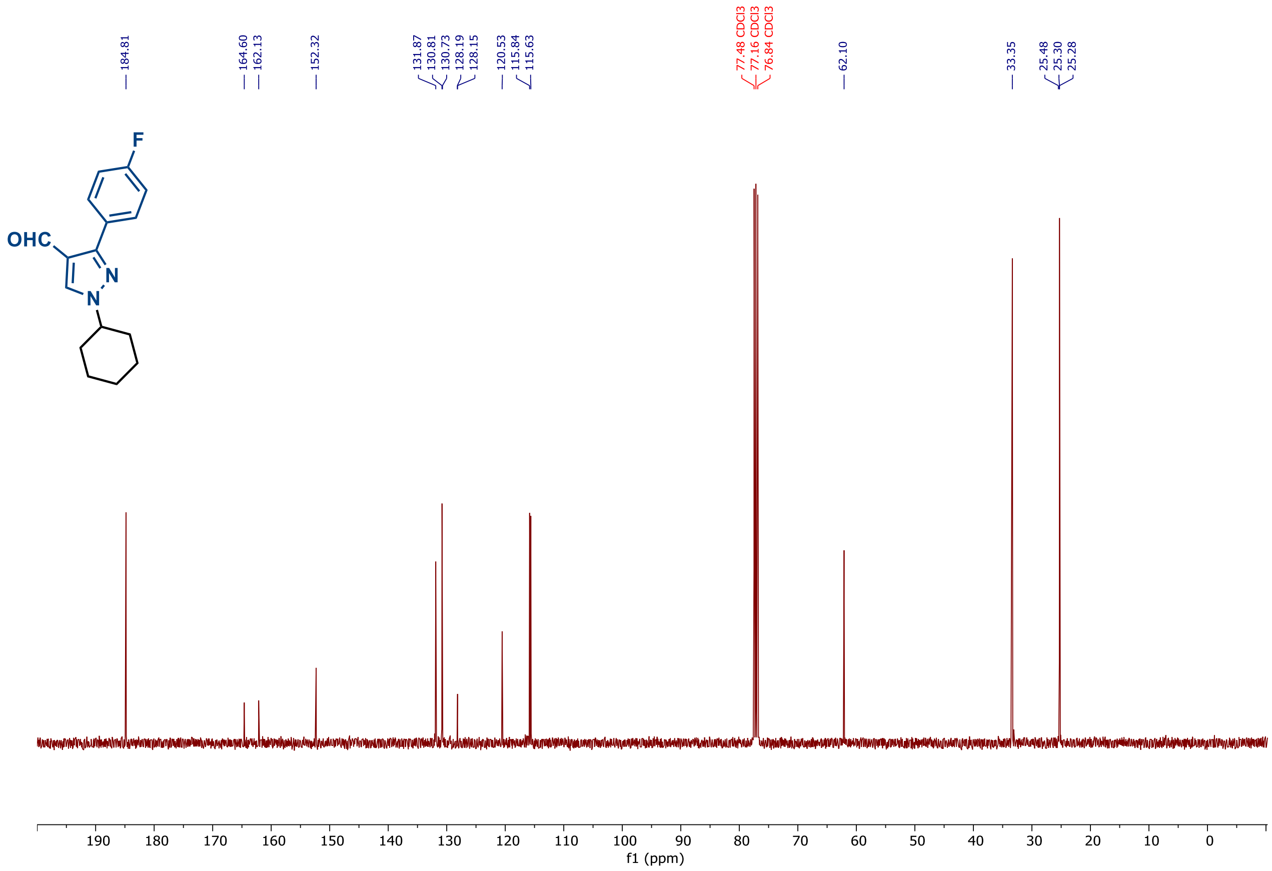

^{19}F NMR (376 MHz, $CDCl_3$) of **23**

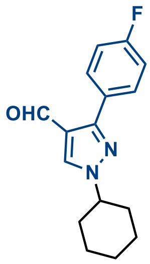

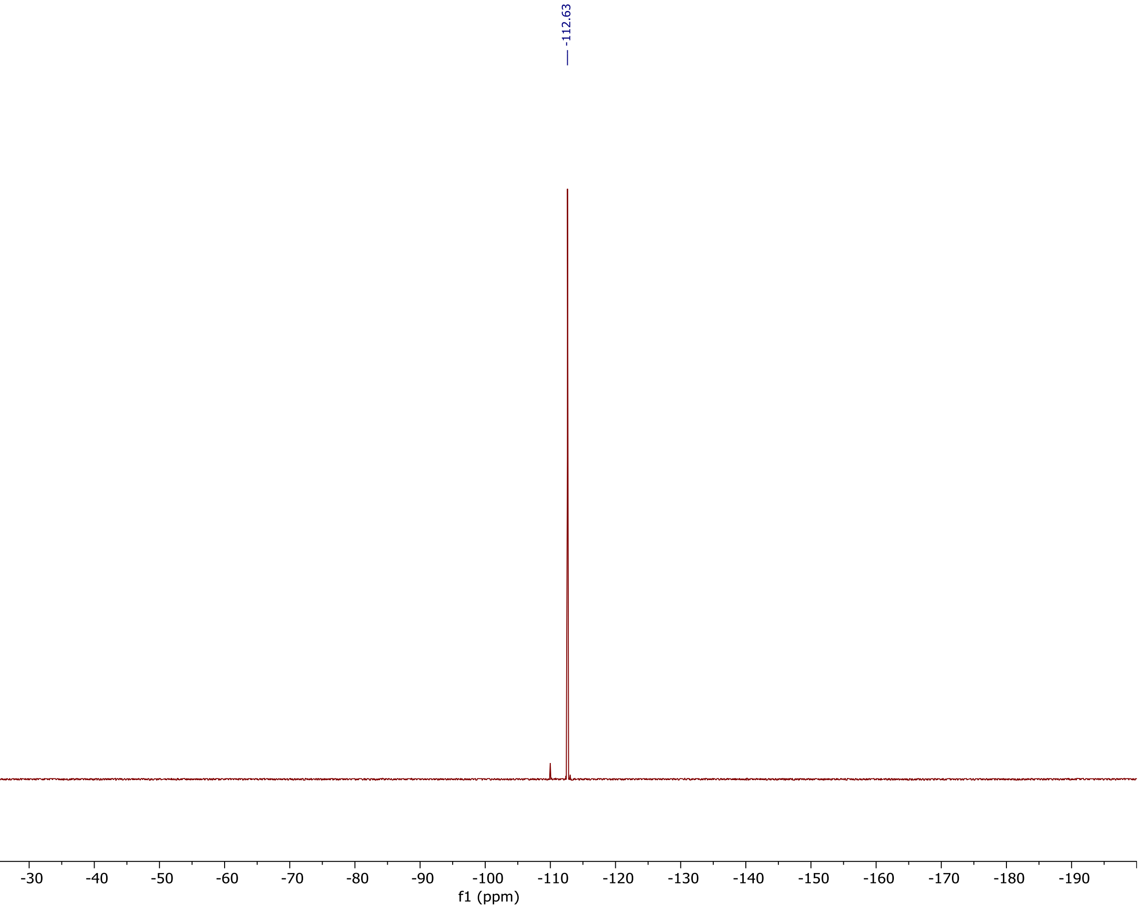

^{1}H NMR (400 MHz, $CDCl_3$) of **24** (*see procedure*)

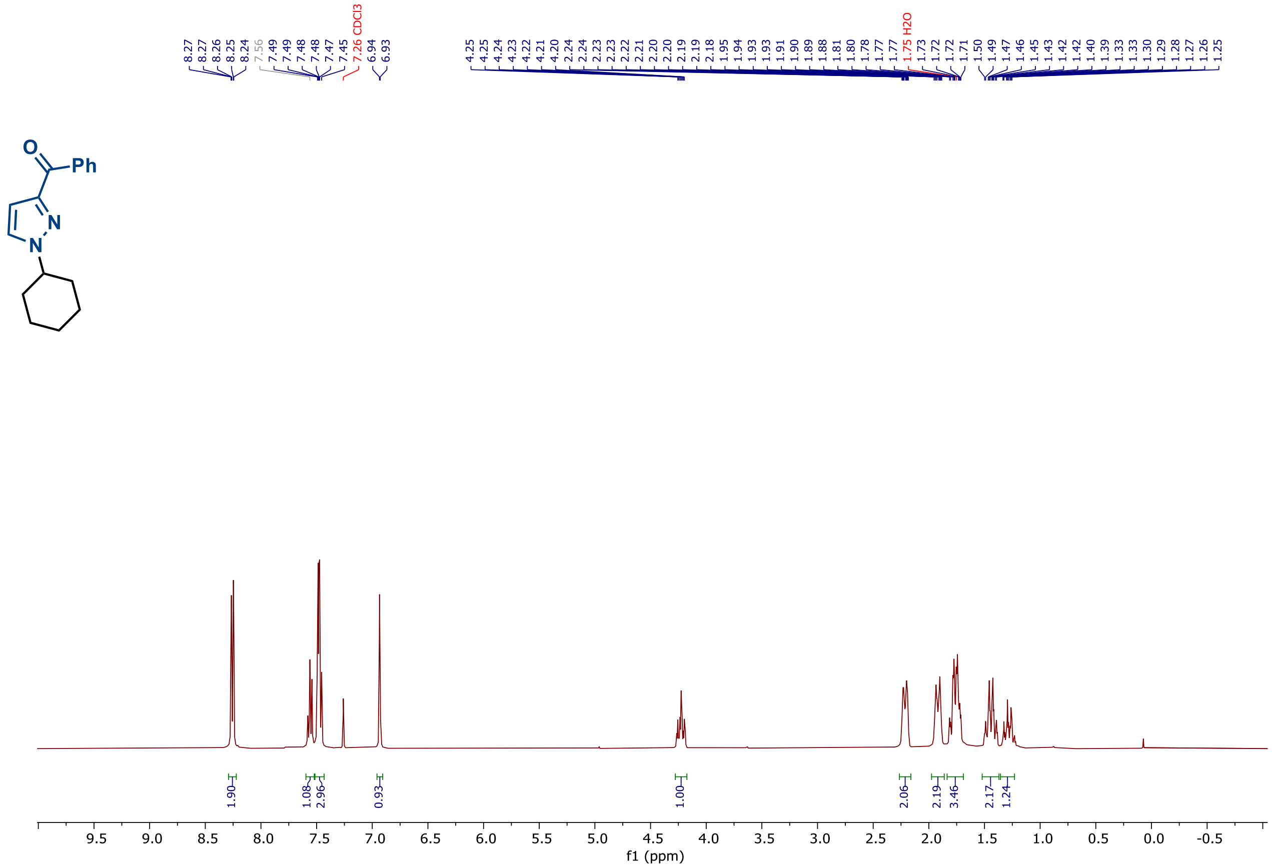

^{13}C NMR (101 MHz, $CDCl_3$) of **24**

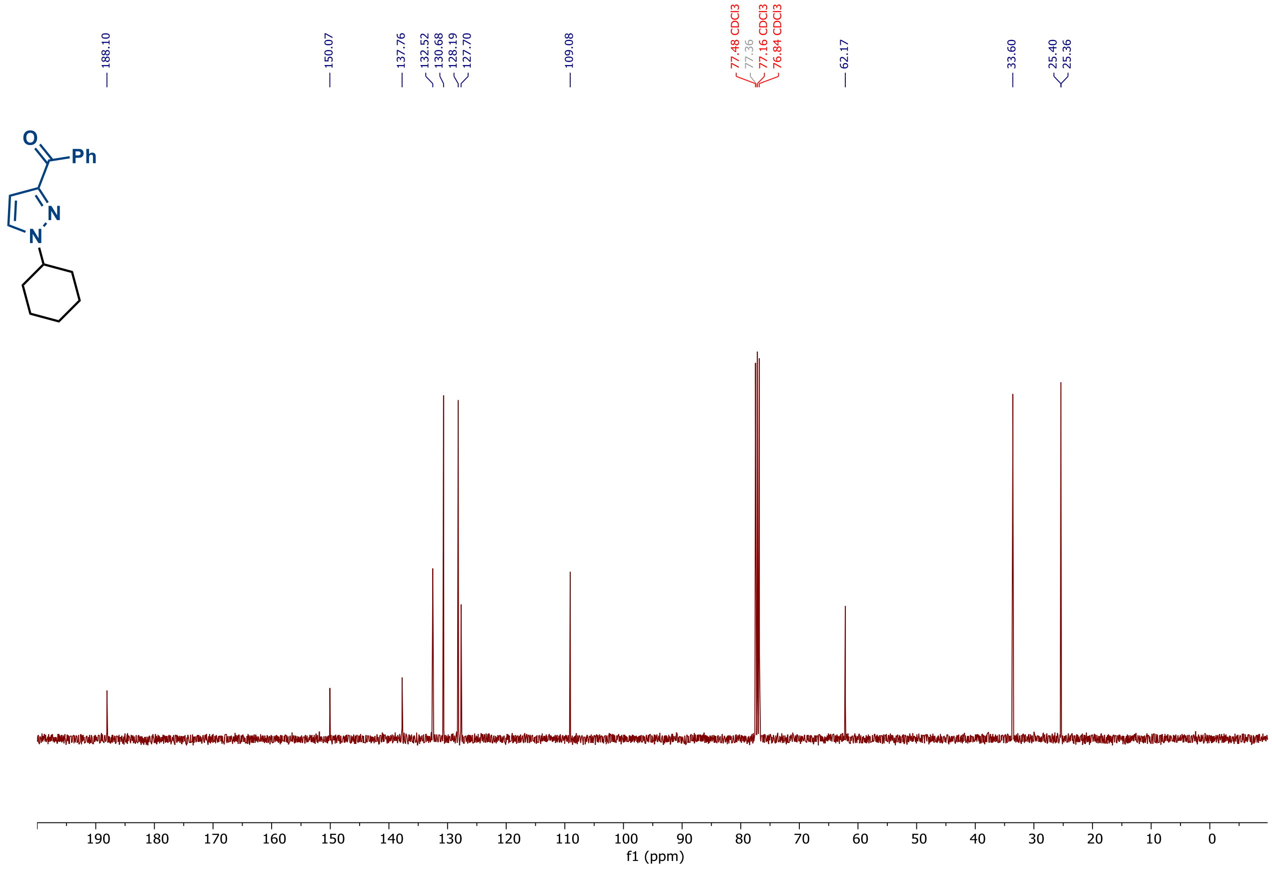

^{1}H NMR (400 MHz, $CDCl_3$) of **25** (see procedure)

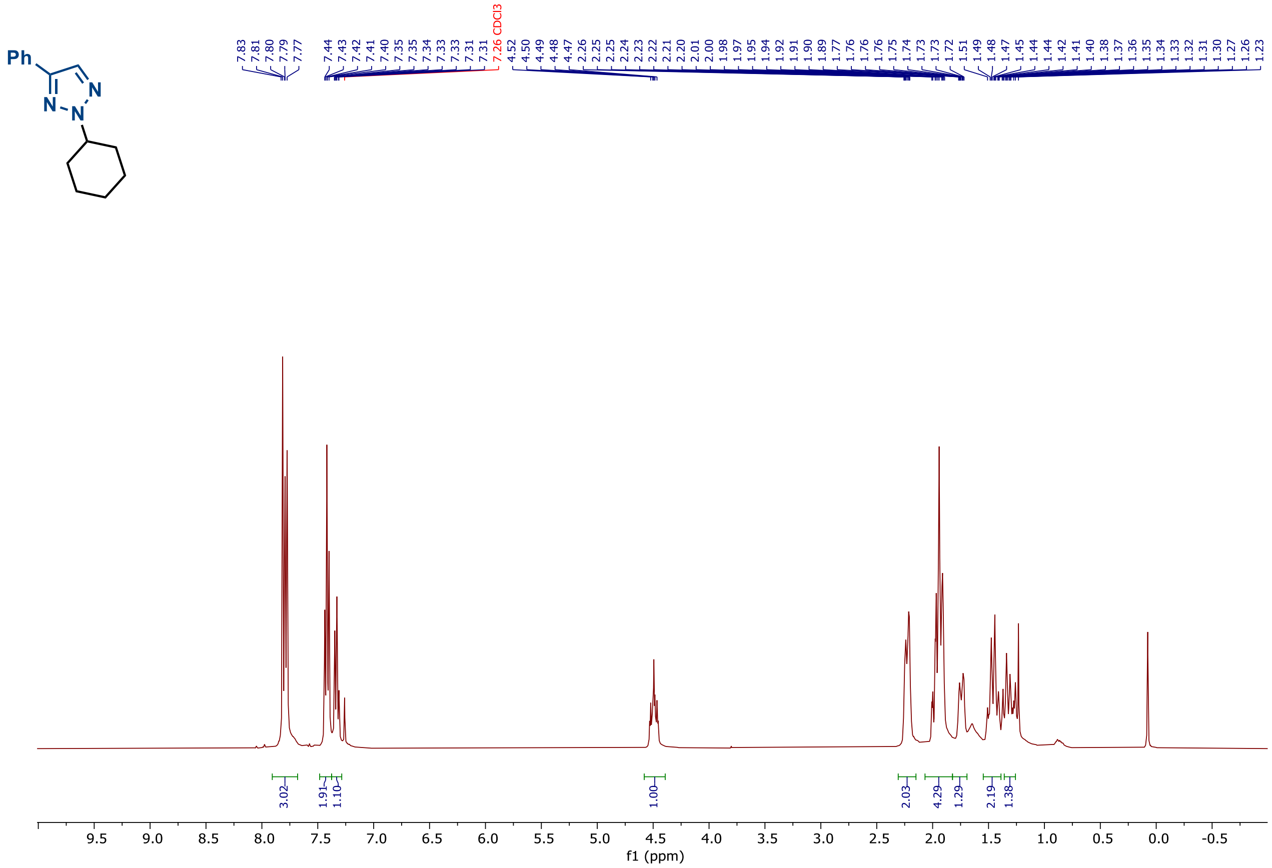

^{13}C NMR (101 MHz, $CDCl_3$) of **25**

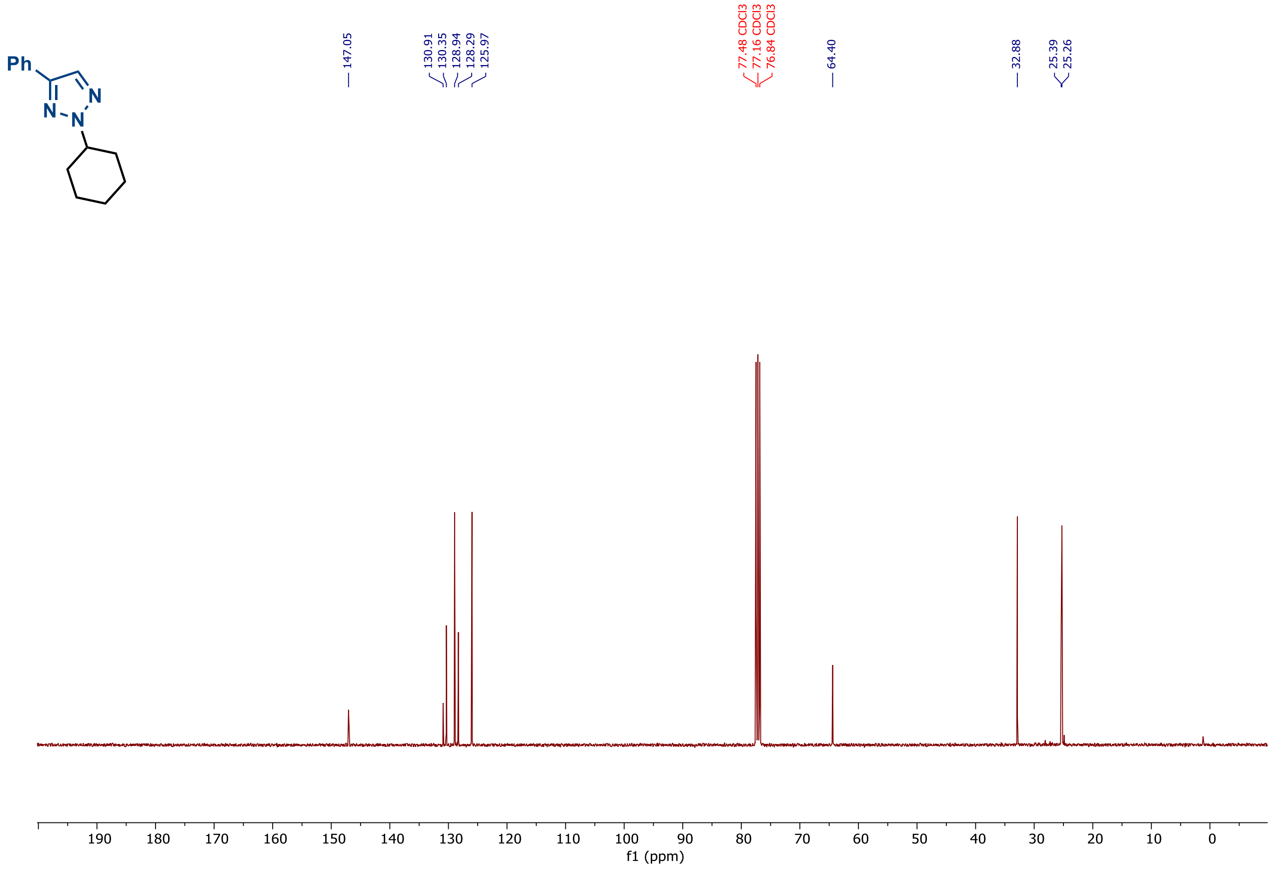

^{1}H NMR (400 MHz, CDCl3) of **26** (see procedure)

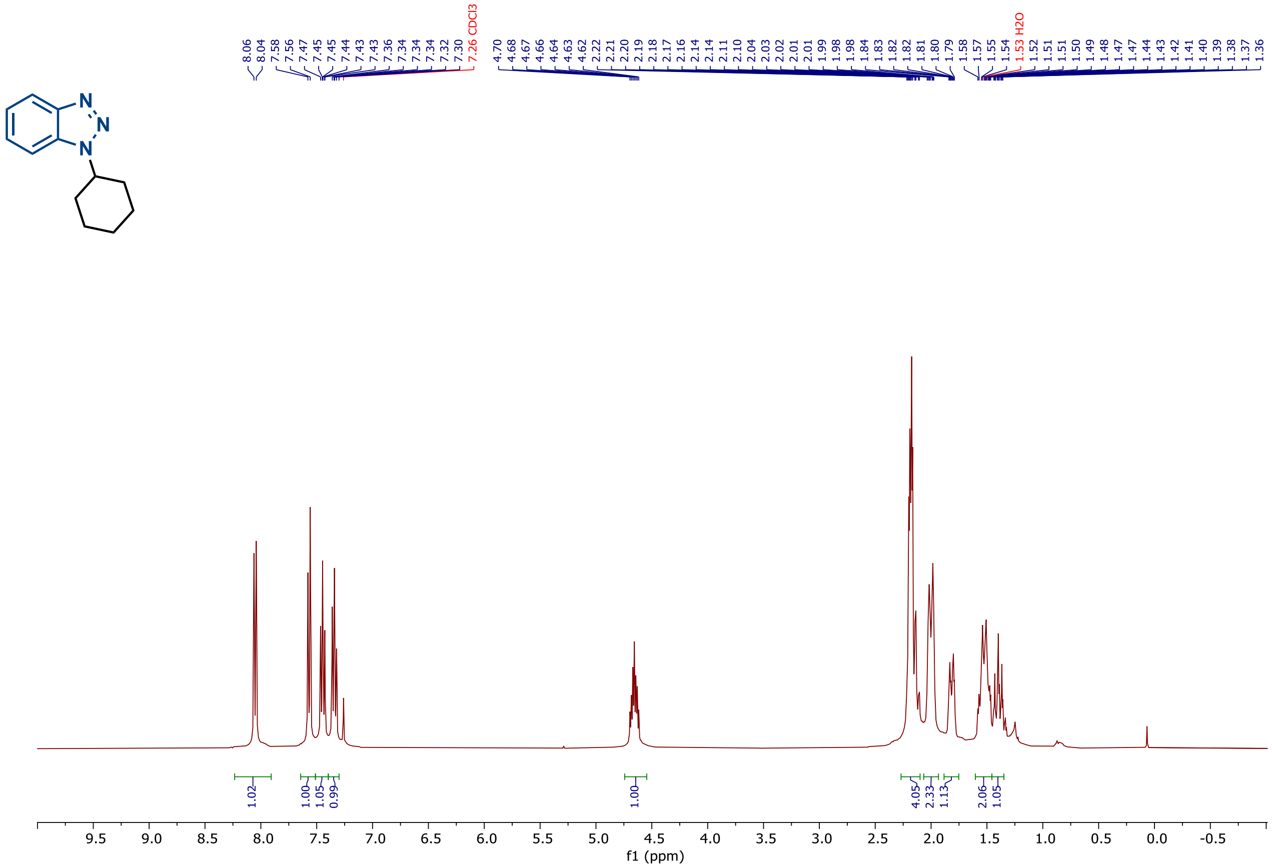

^{13}C NMR (101 MHz, CDCl3) of **26**

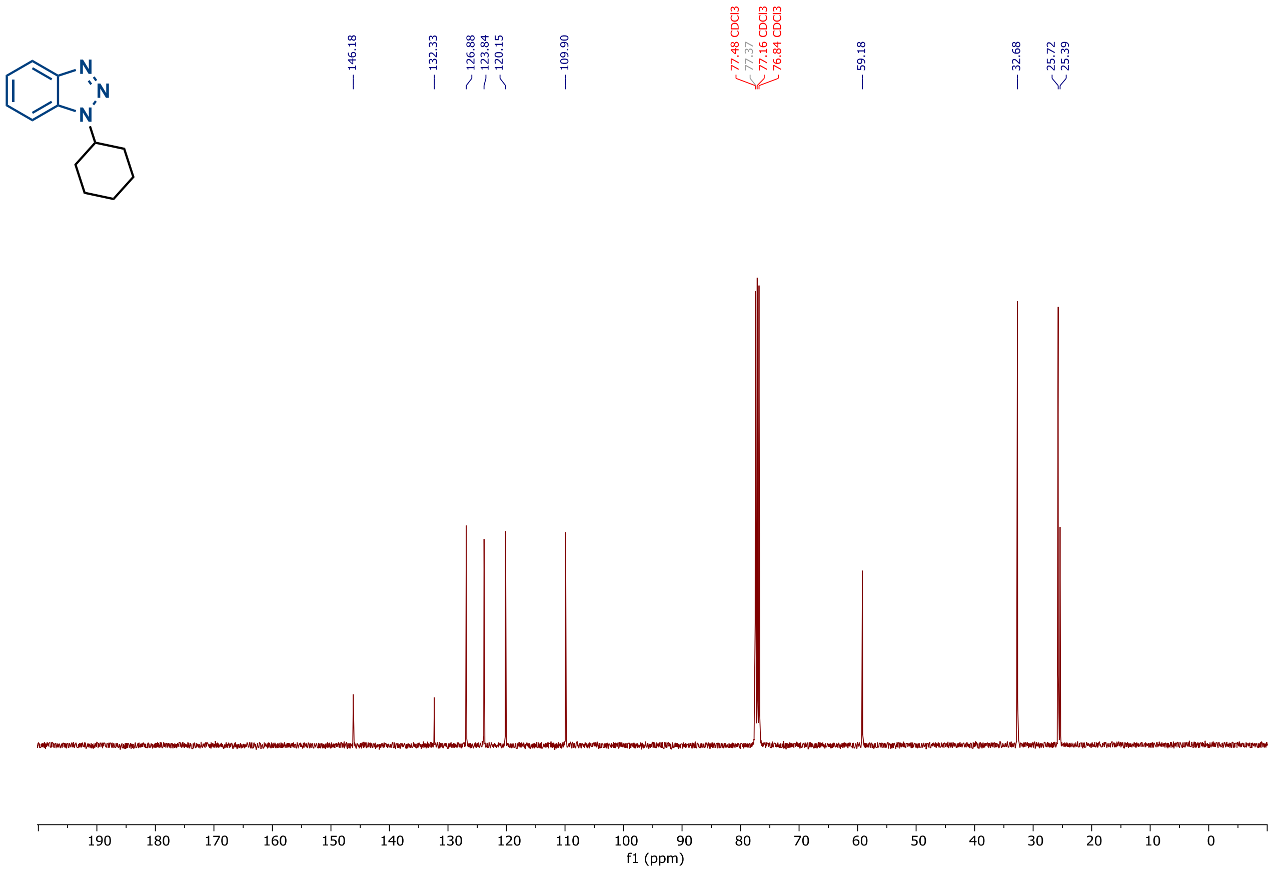

^{1}H NMR (400 MHz, $CDCl_3$) of **27** (*see procedure*)

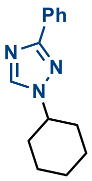

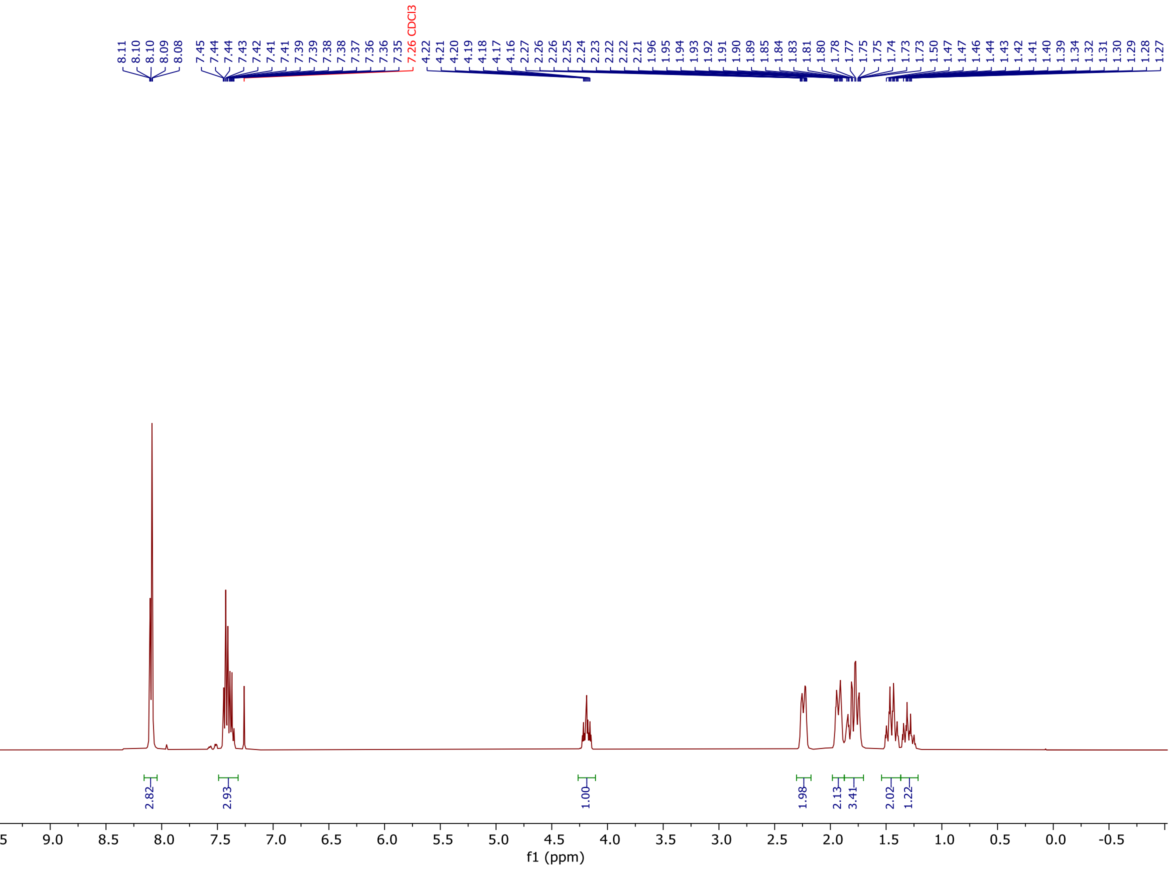

^{13}C NMR (101 MHz, $CDCl_3$) of **27**

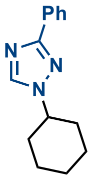

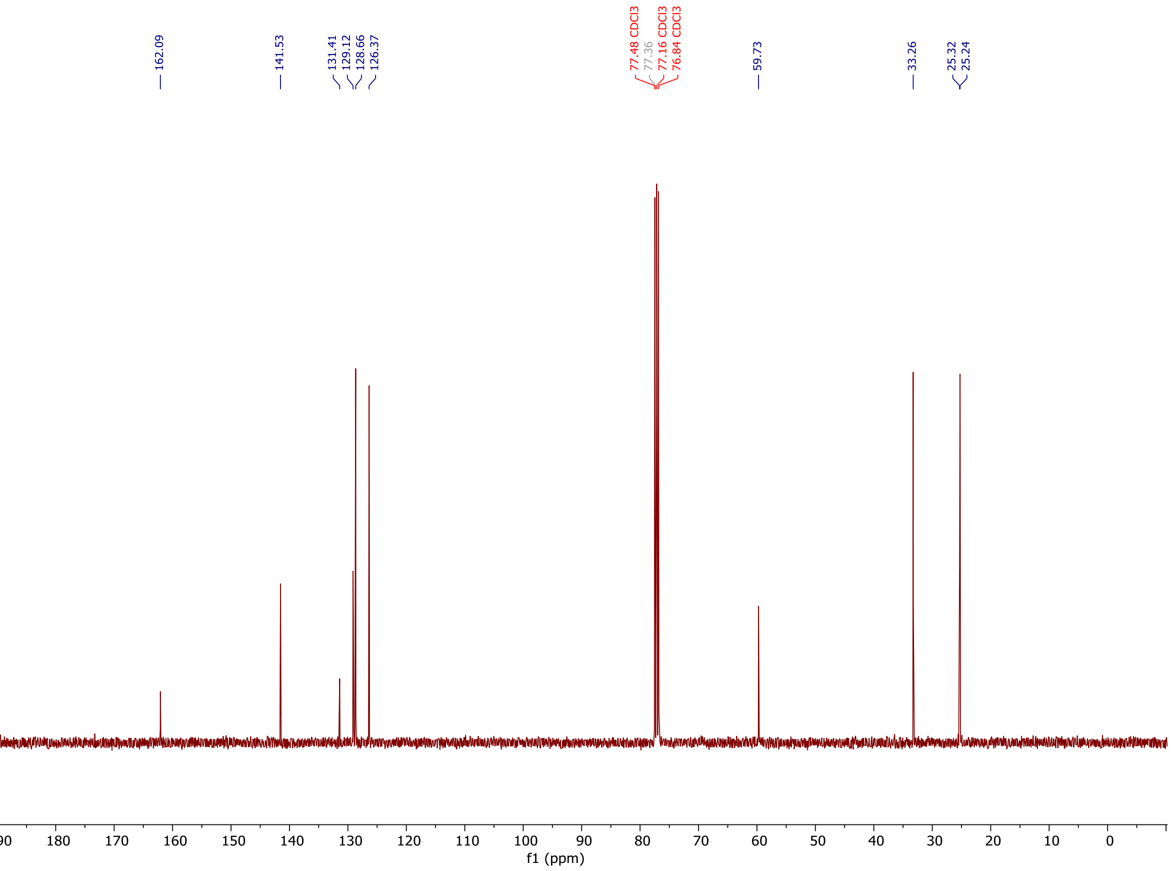

^{1}H NMR (400 MHz, $CDCl_3$) of **28** (*see procedure*)

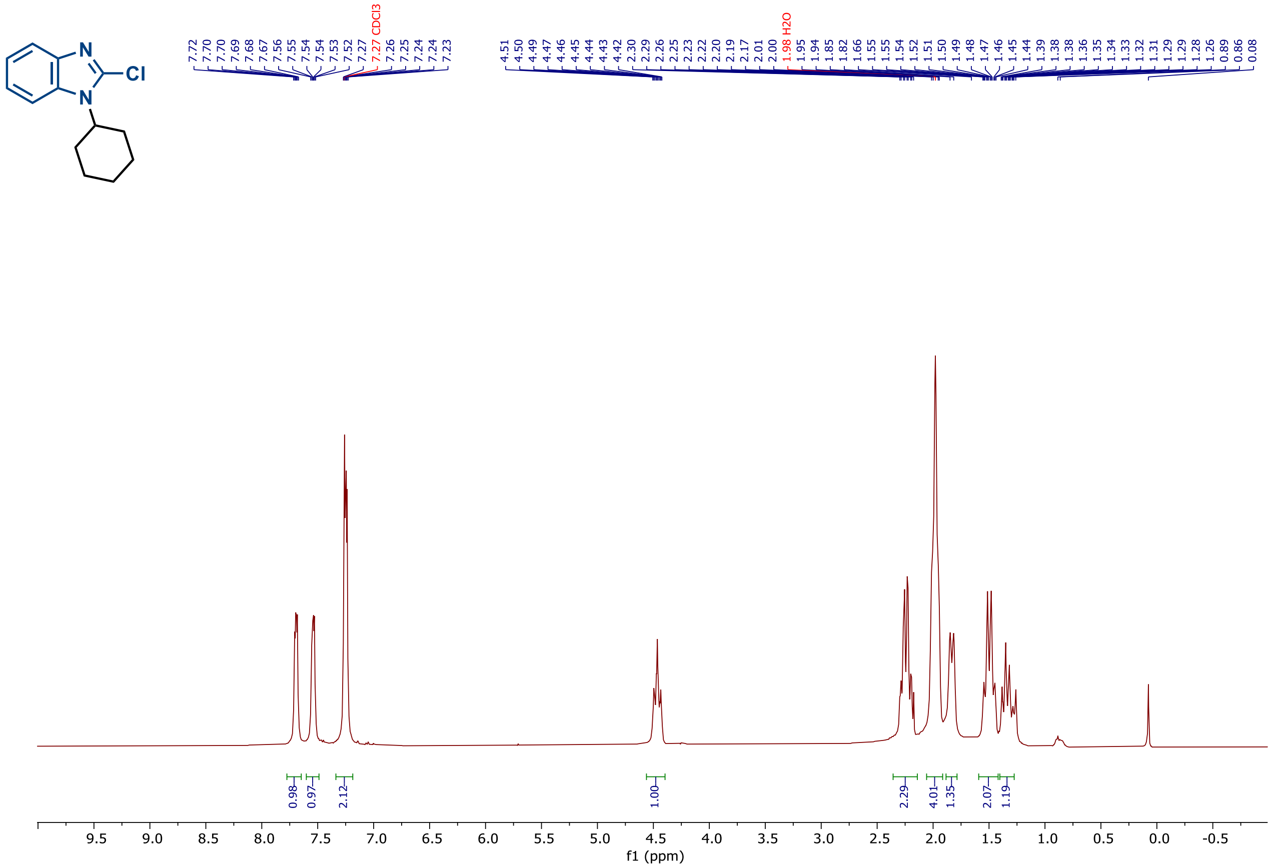

^{13}C NMR (101 MHz, $CDCl_3$) of **28**

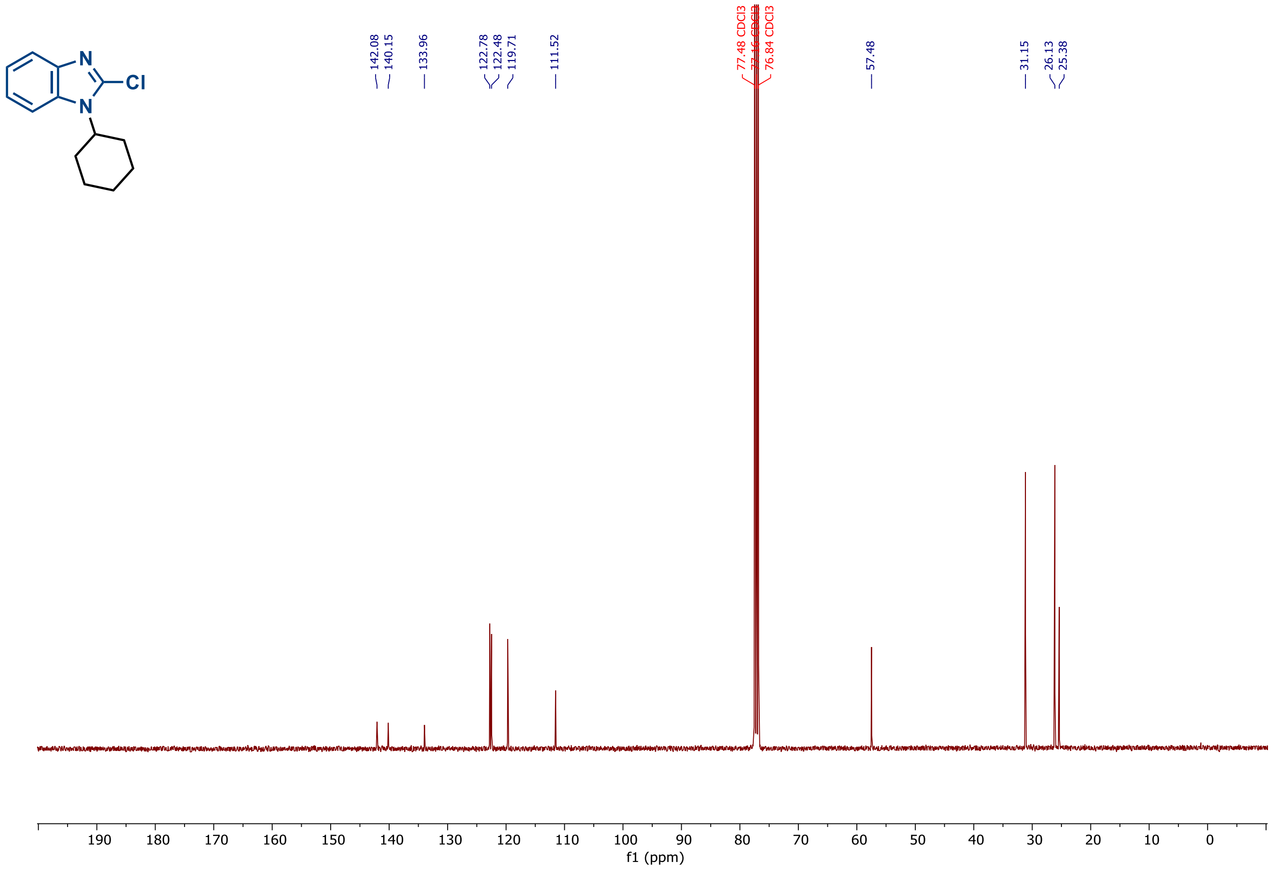

^{1}H NMR (400 MHz, $CDCl_3$) of **29** (<u>*see procedure*</u>)

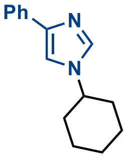

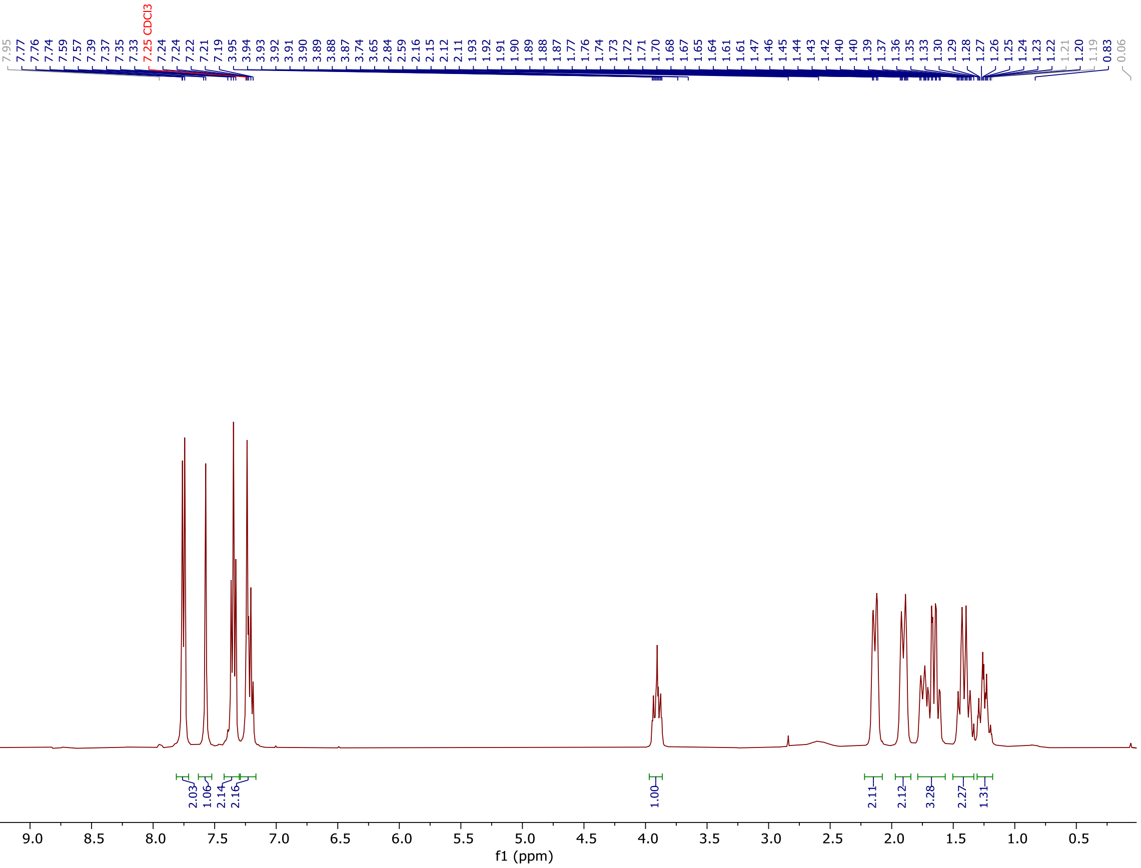

^{13}C NMR (101 MHz, $CDCl_3$) of **29**

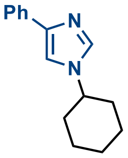

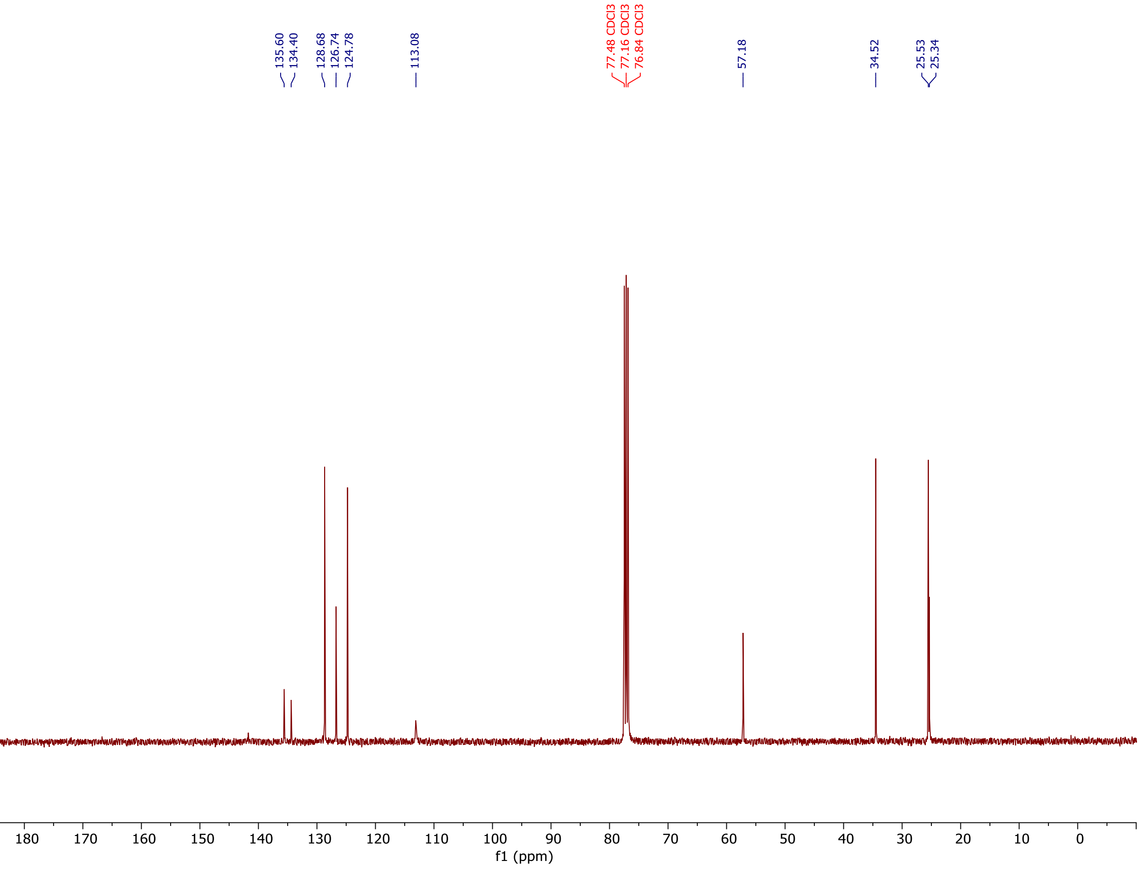

^{1}H NMR (400 MHz, $CDCl_3$) of **30** (*see procedure*)

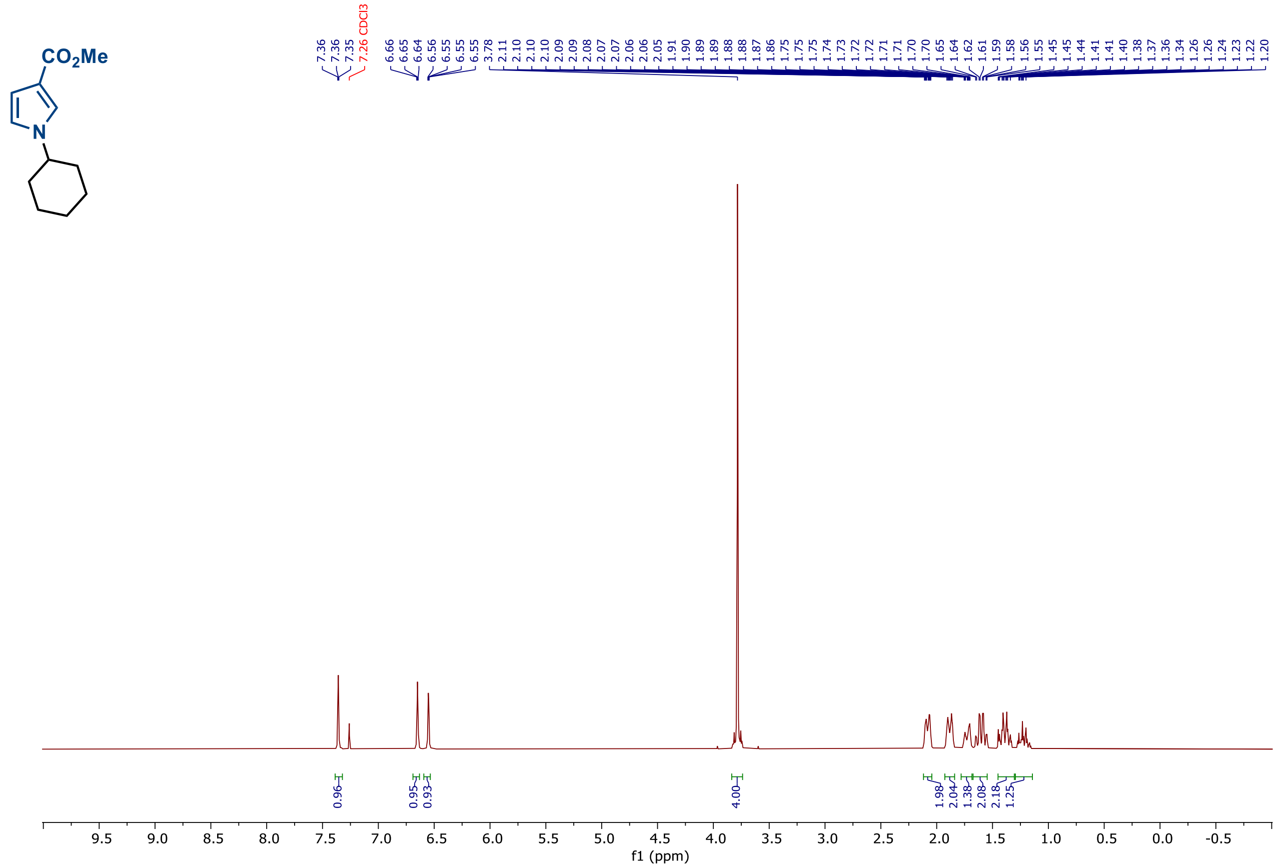

^{13}C NMR (101 MHz, $CDCl_3$) of **30**

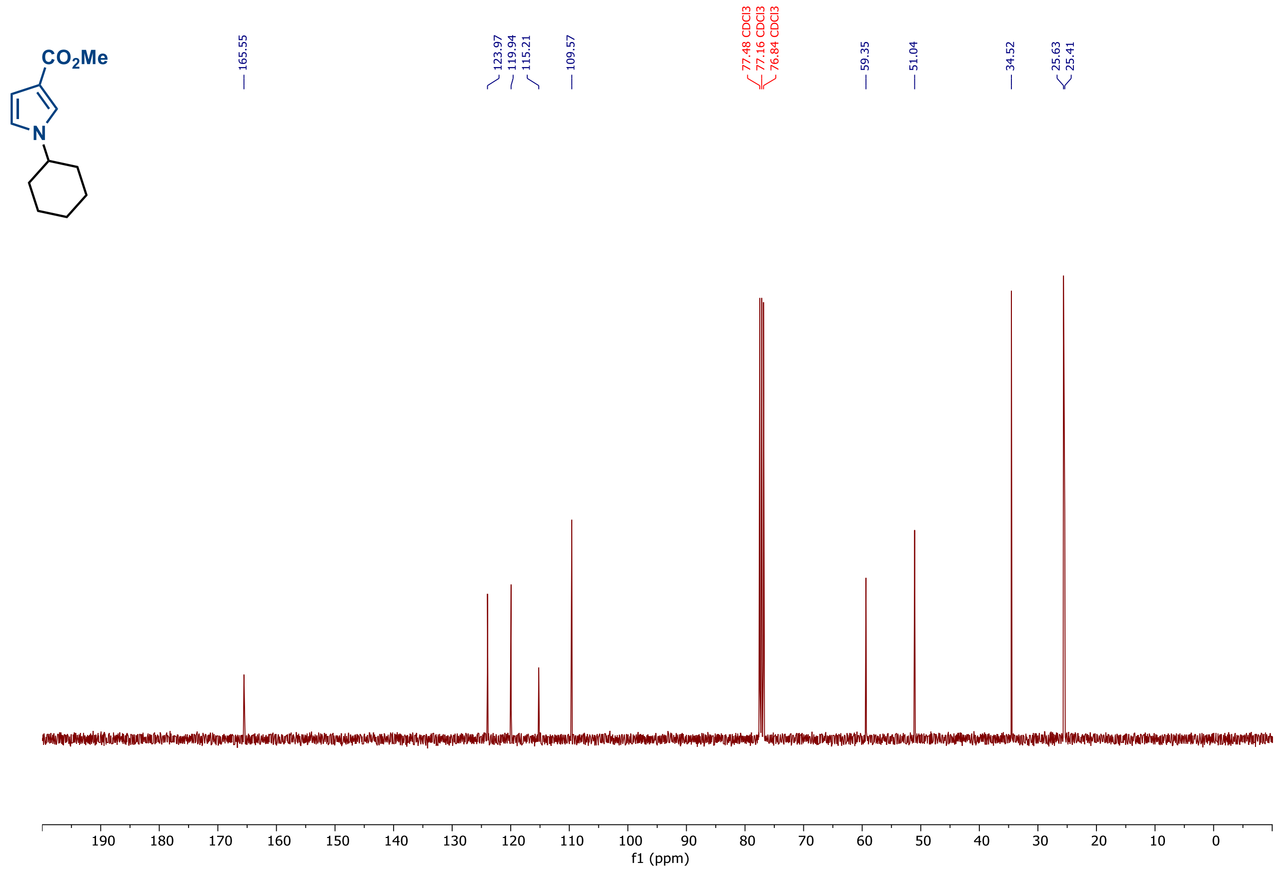

^{1}H NMR (400 MHz, $CDCl_3$) of **31** (*see procedure*)

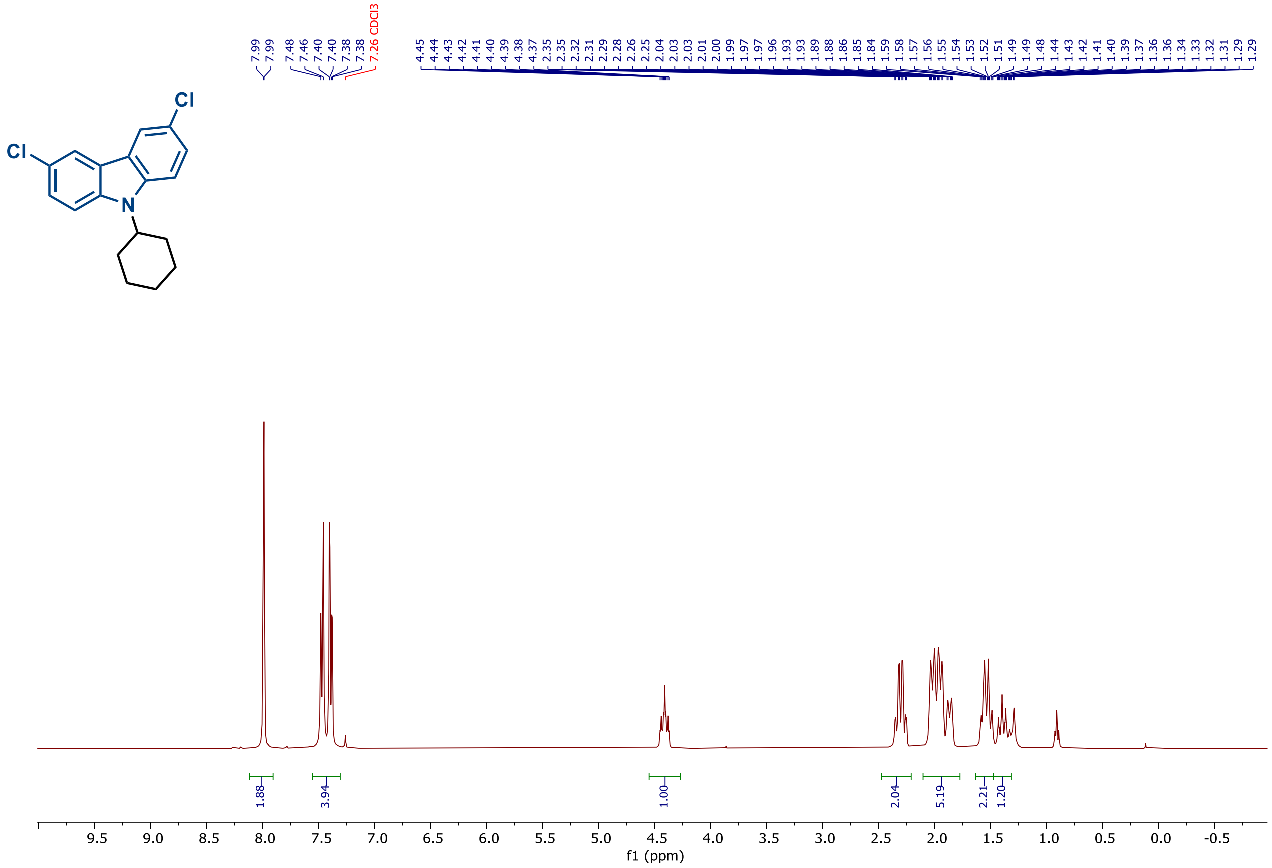

^{13}C NMR (101 MHz, $CDCl_3$) of **31**

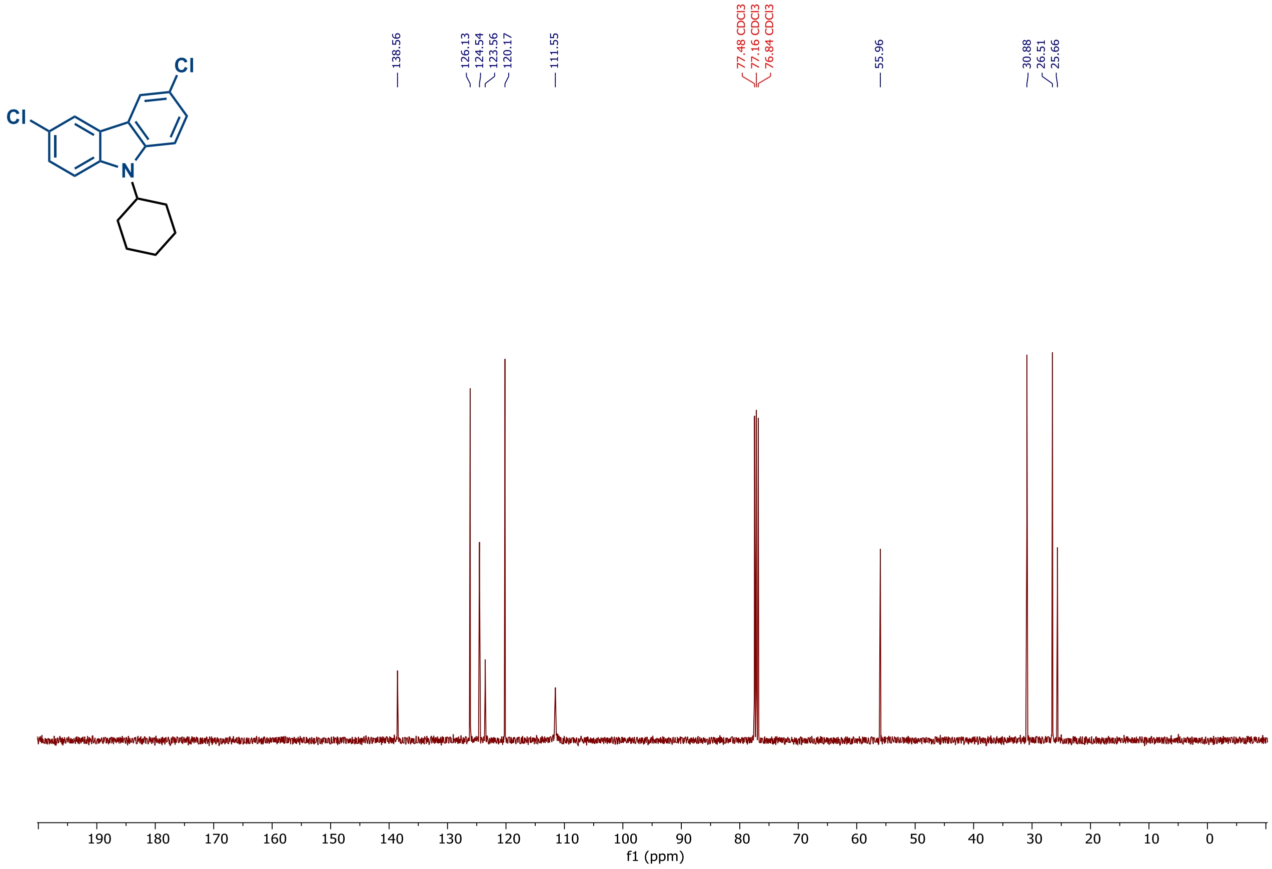

^{1}H NMR (400 MHz, $CDCl_3$) of **32** (*see procedure*)

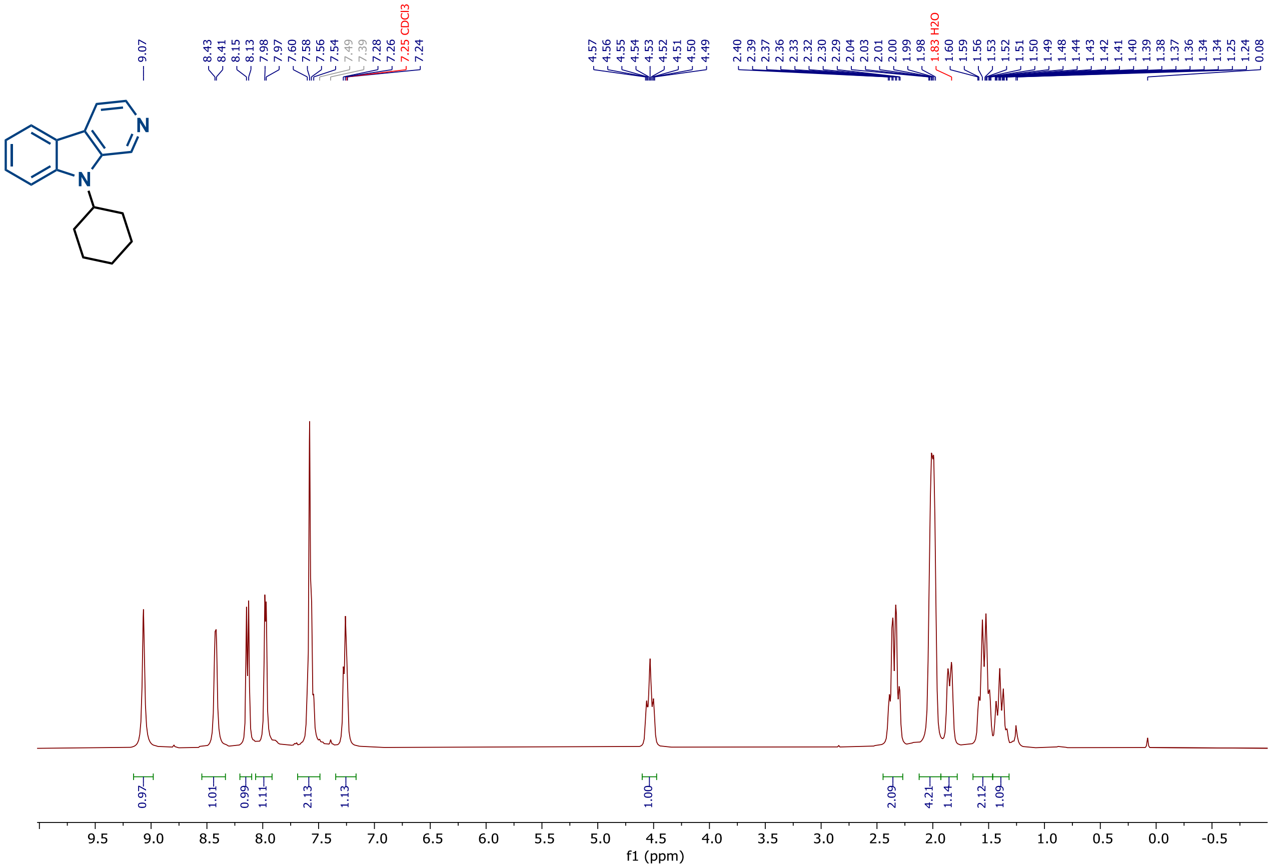

^{13}C NMR (101 MHz, $CDCl_3$) of **32**

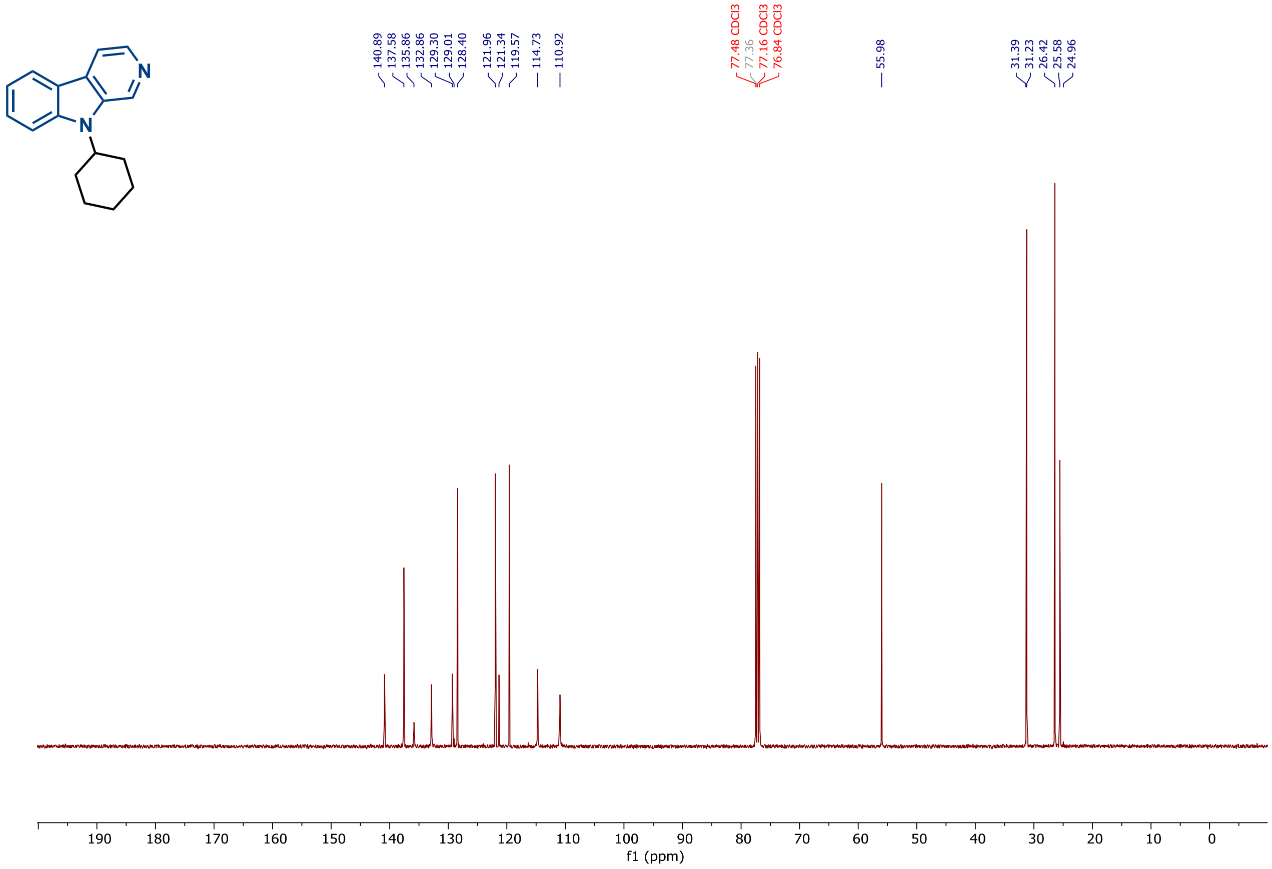

^{1}H NMR (400 MHz, $CDCl_3$) of **33** (*see procedure*)

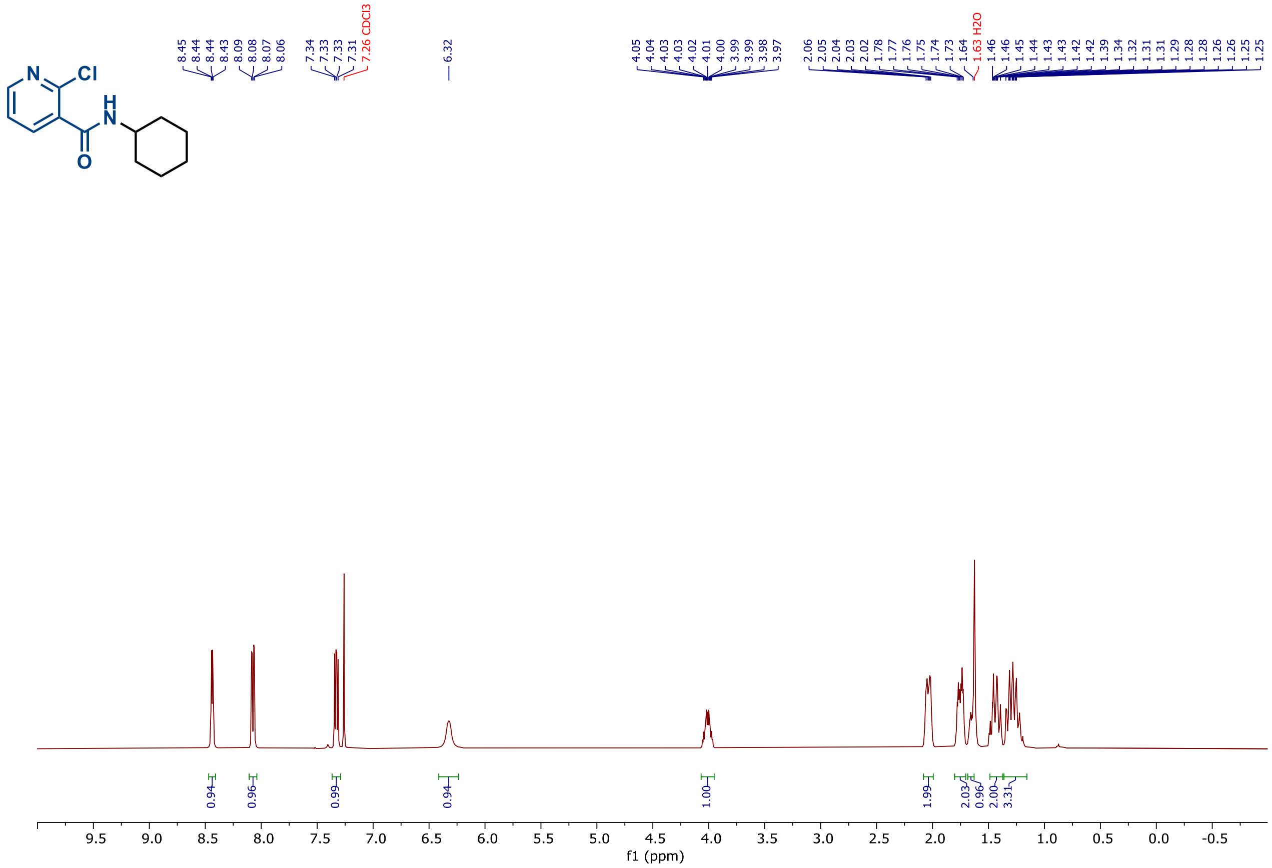

^{13}C NMR (101 MHz, $CDCl_3$) of **33**

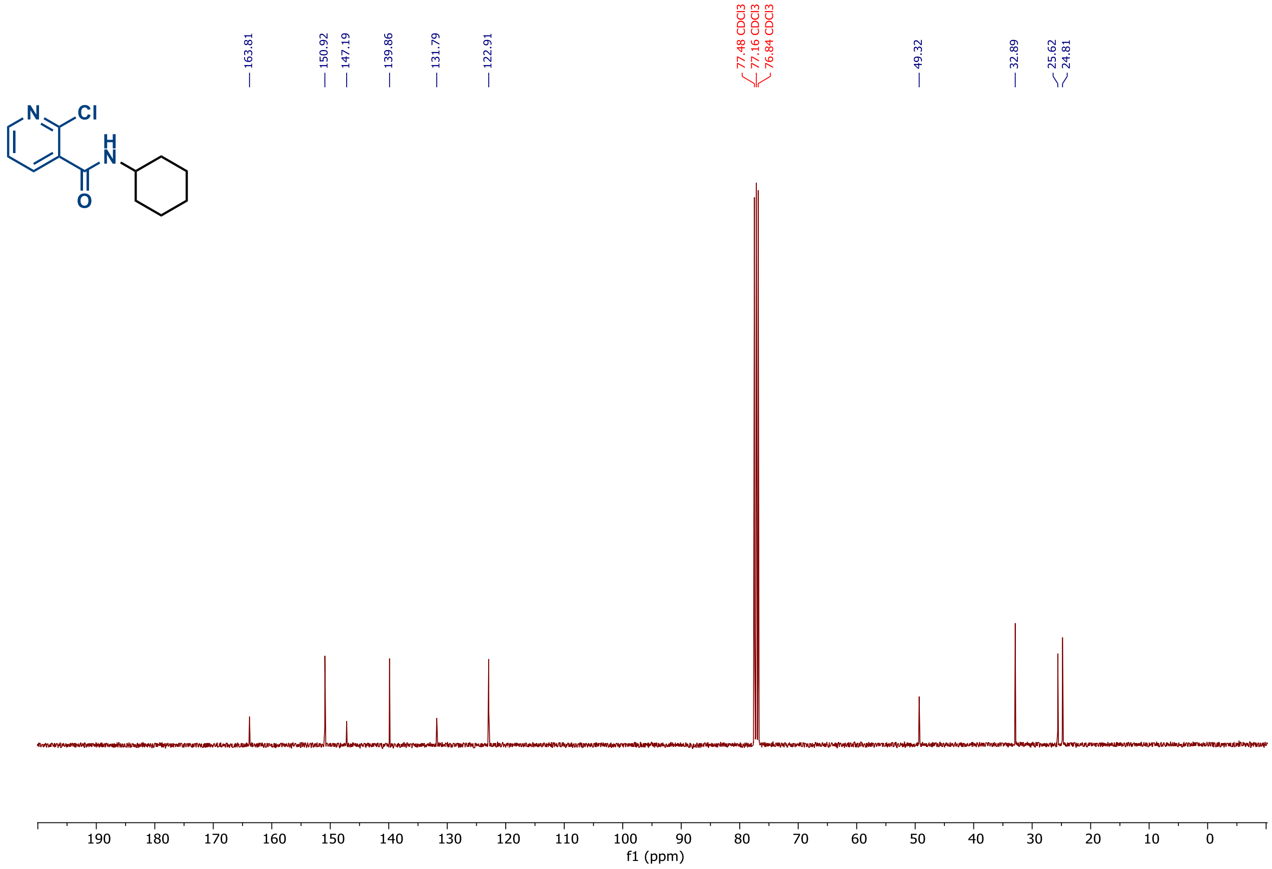

^{1}H NMR (400 MHz, CDCl$_3$) of **34** (see procedure)

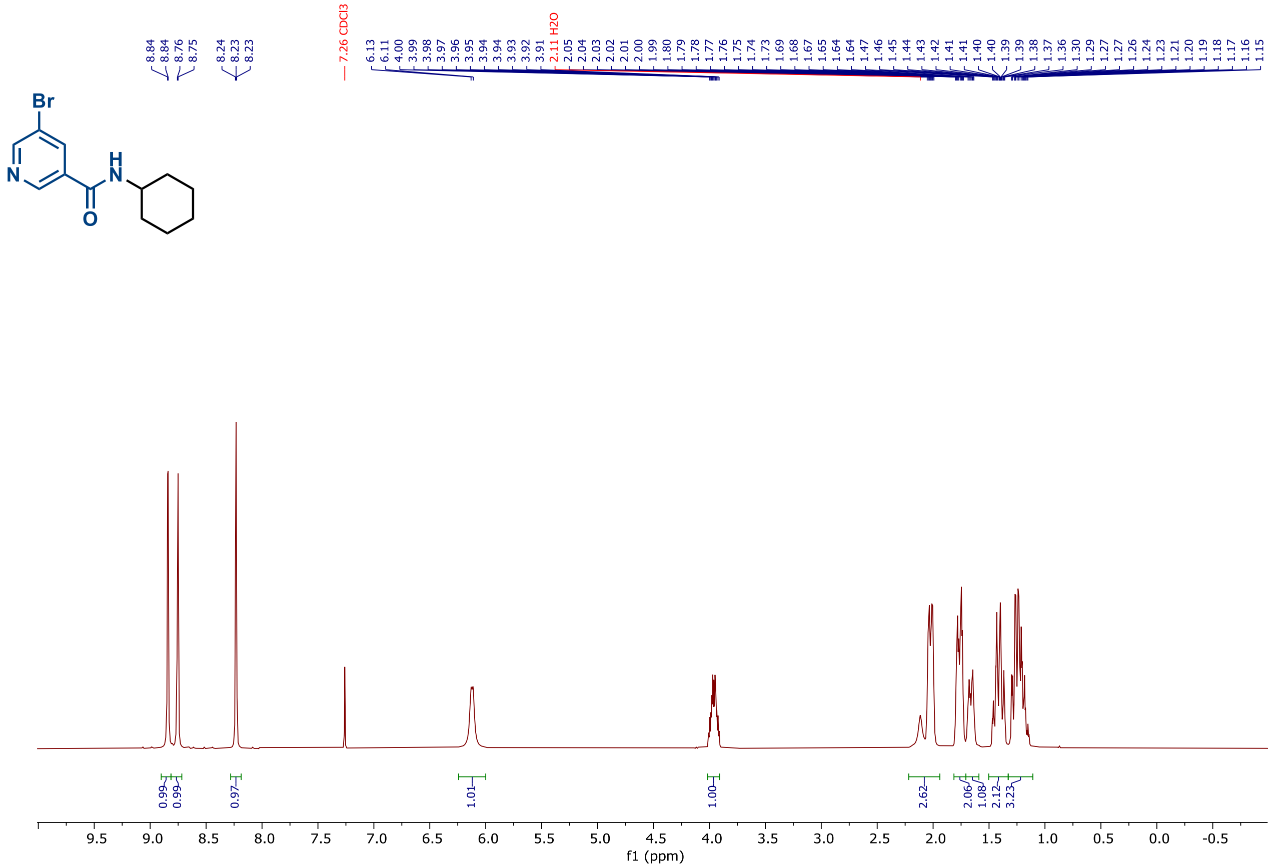

^{13}C NMR (101 MHz, CDCl$_3$) of **34**

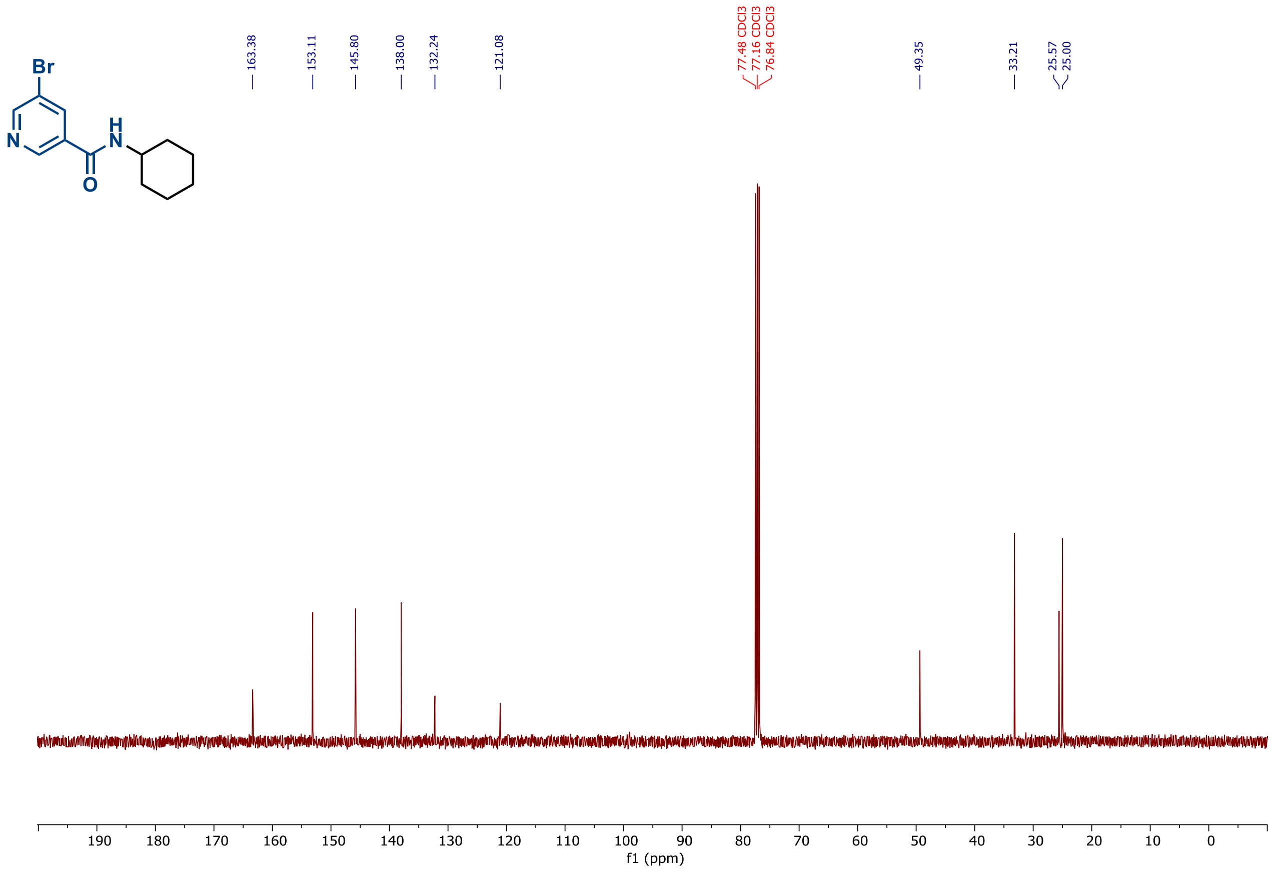

^1H NMR (400 MHz, $CDCl_3$) of **35** (see procedure)

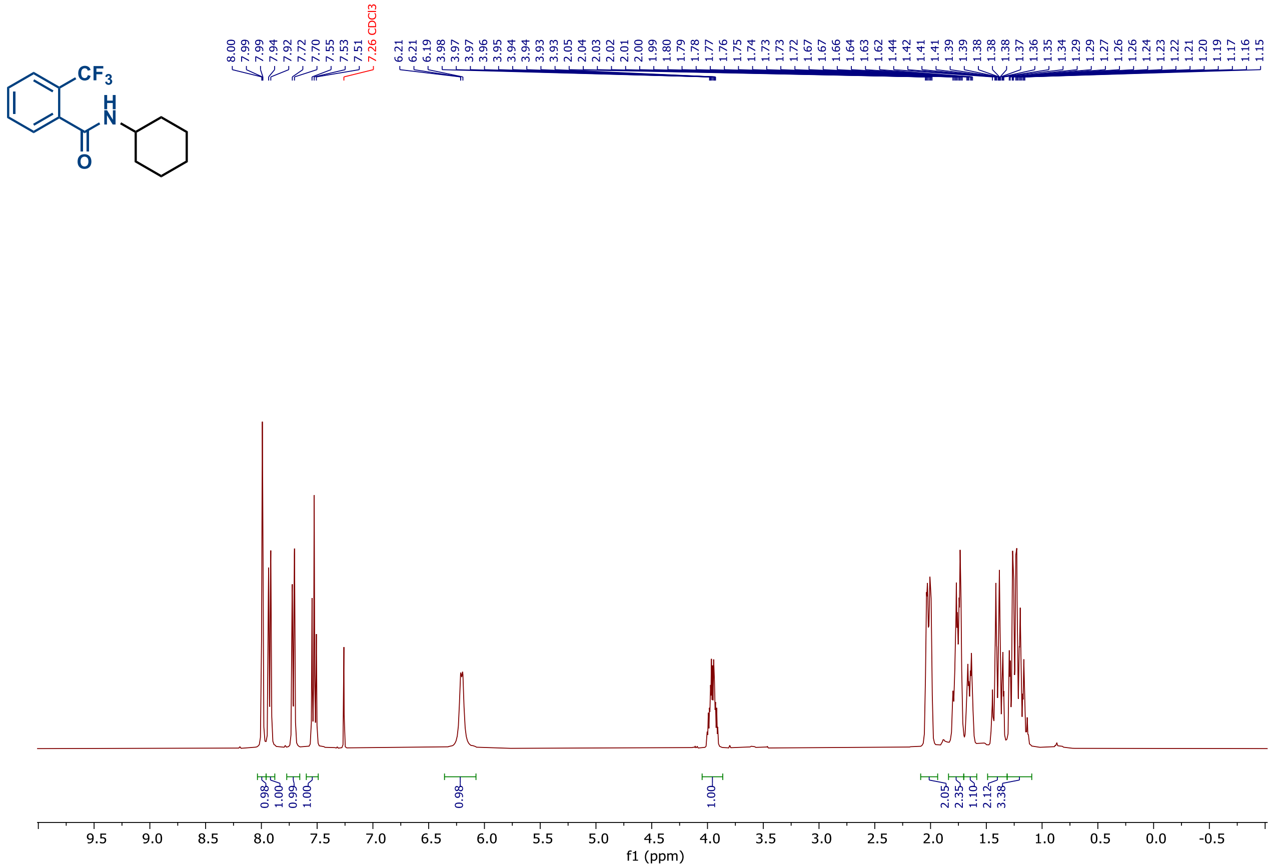

^{13}C NMR (101 MHz, $CDCl_3$) of **35**

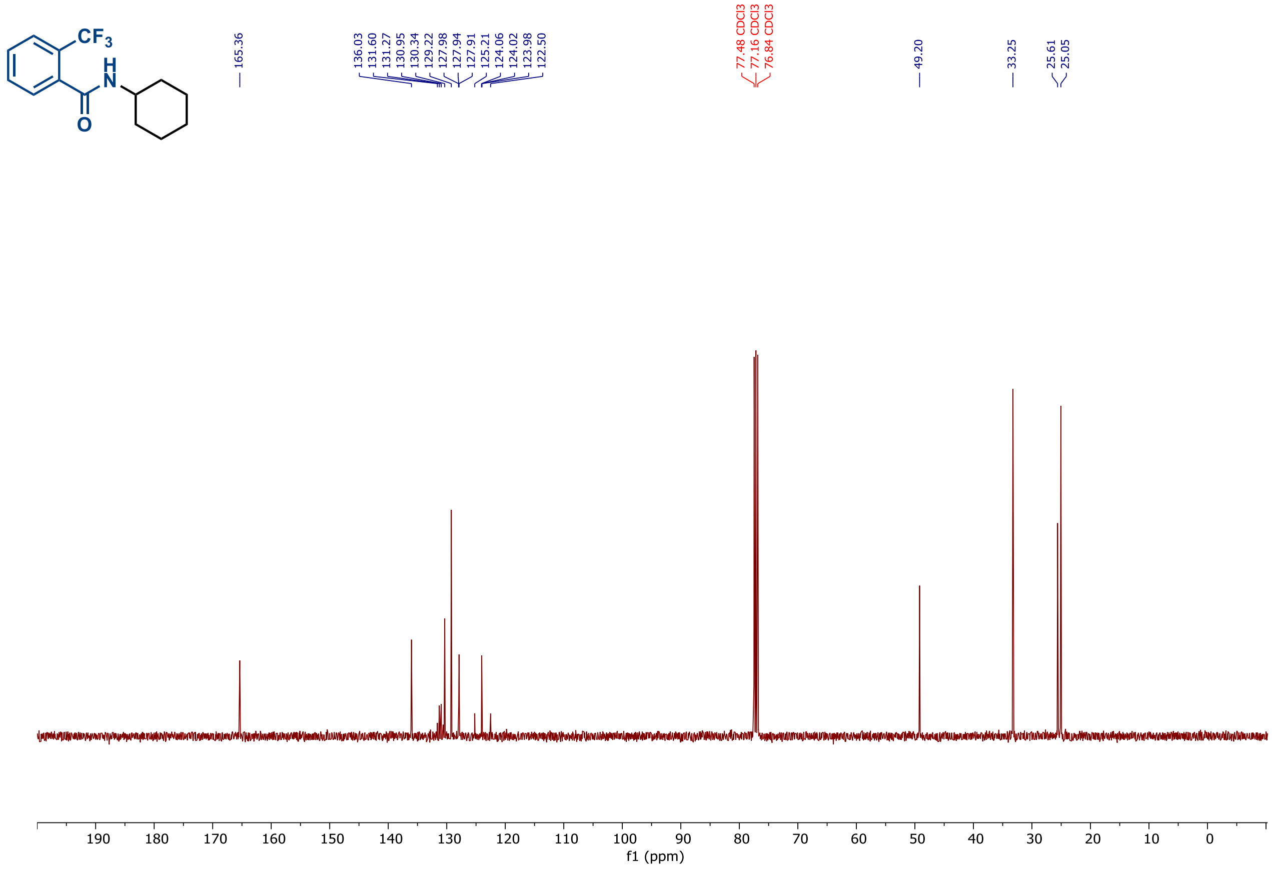

^{19}F NMR (376 MHz, $CDCl_3$) of **35**

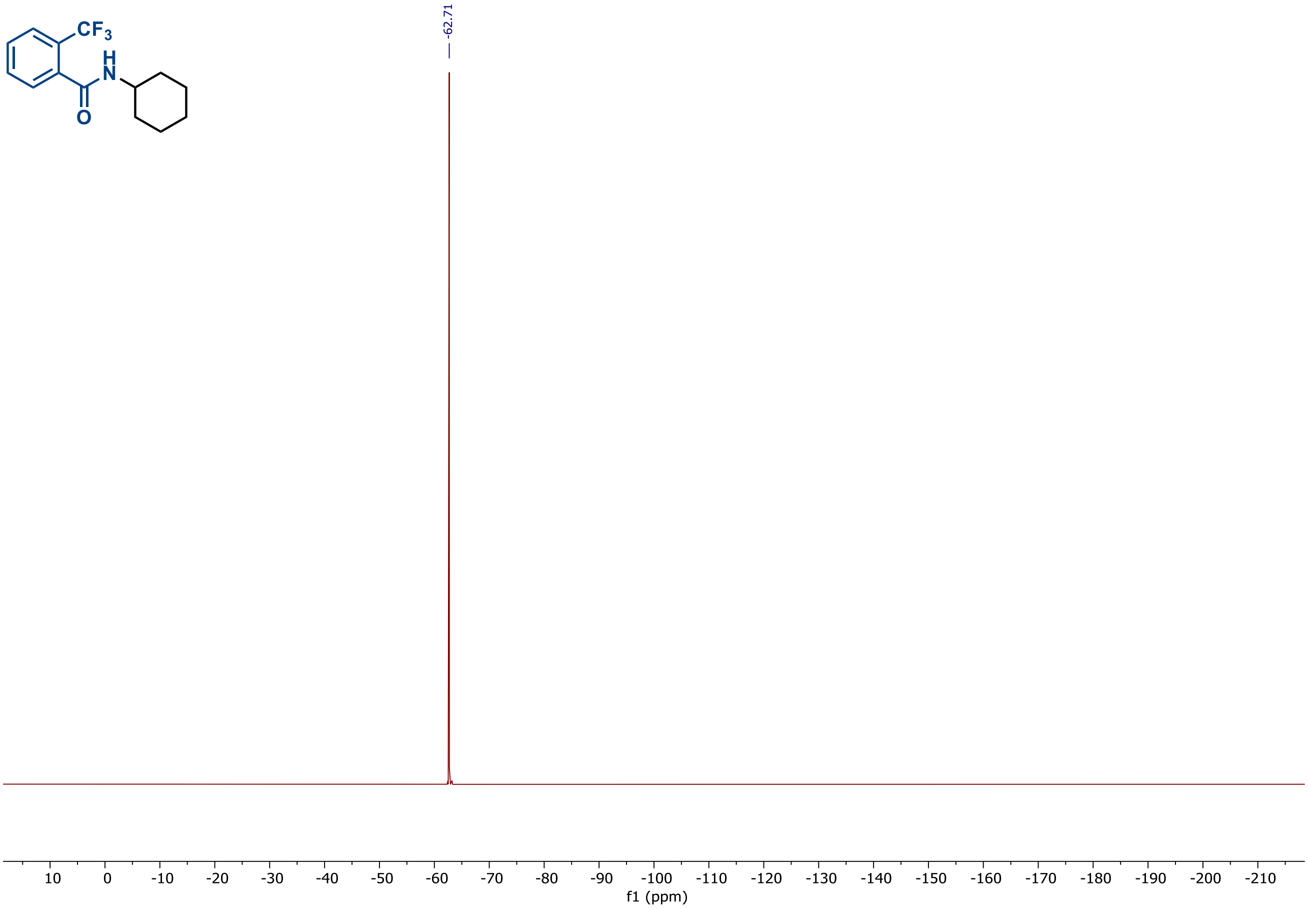

^{1}H NMR (400 MHz, $CDCl_3$) of **36** (*see procedure*)

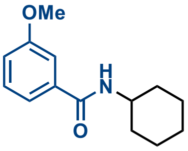

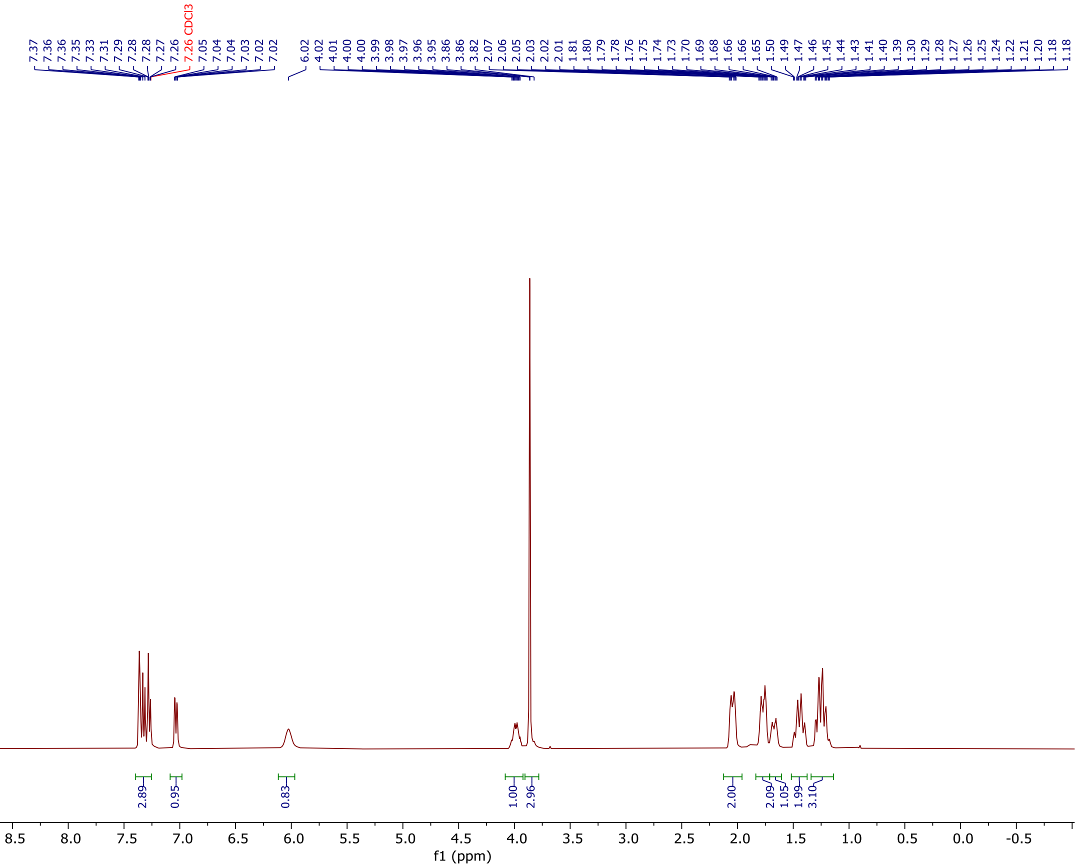

^{13}C NMR (101 MHz, $CDCl_3$) of **36**

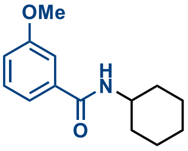

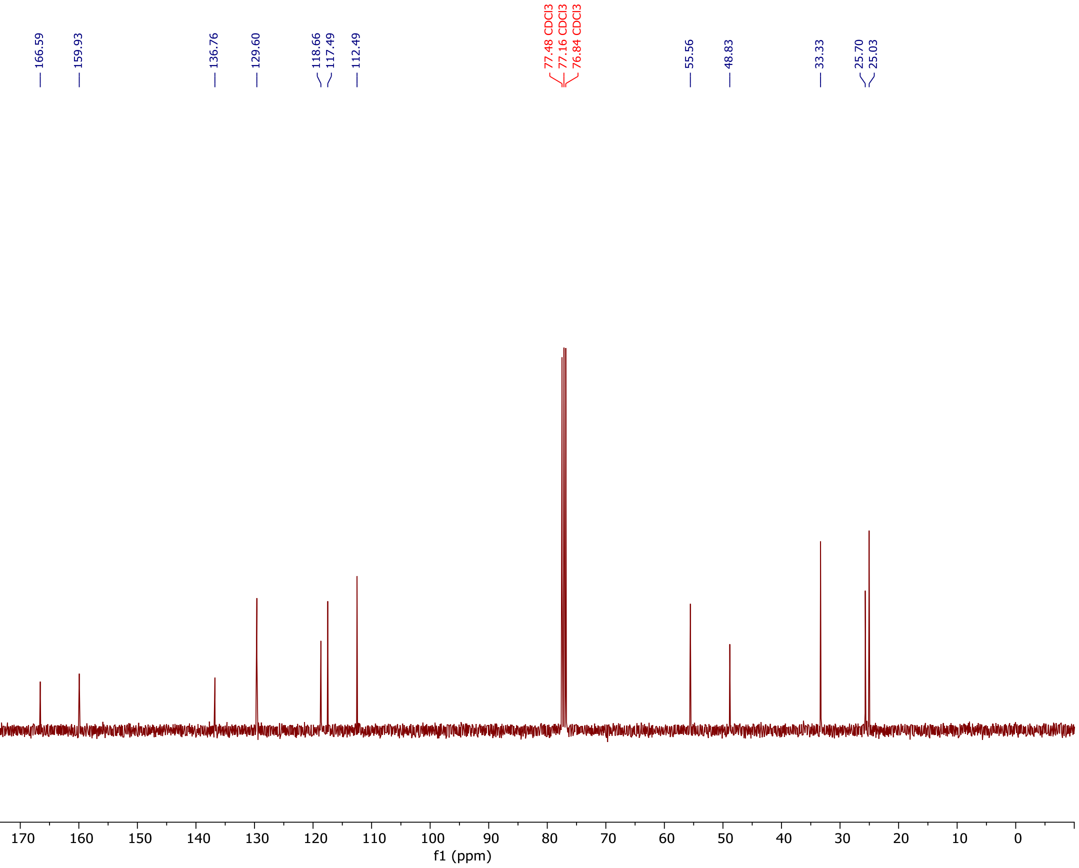

^{1}H NMR (400 MHz, $CDCl_3$) of **37** (*see procedure*)

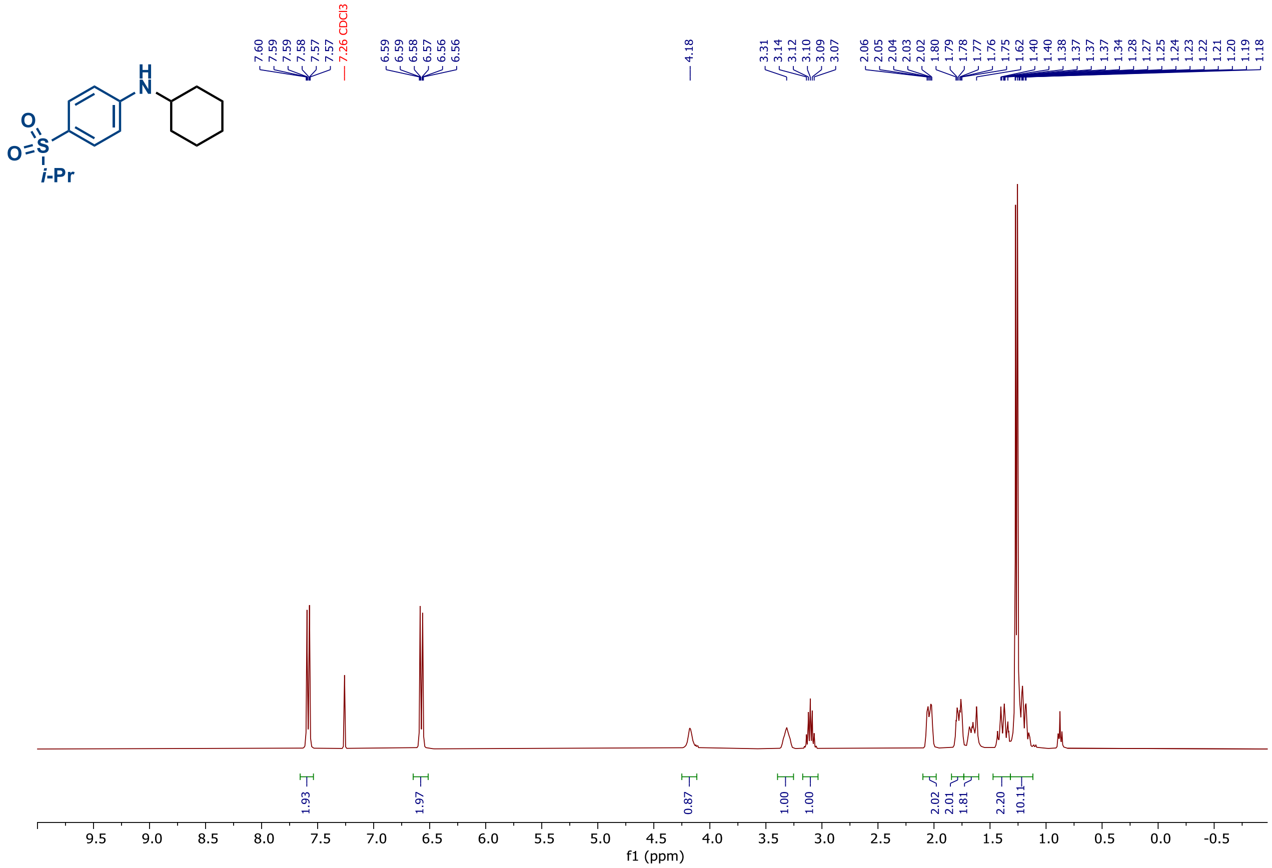

^{13}C NMR (101 MHz, $CDCl_3$) of **37**

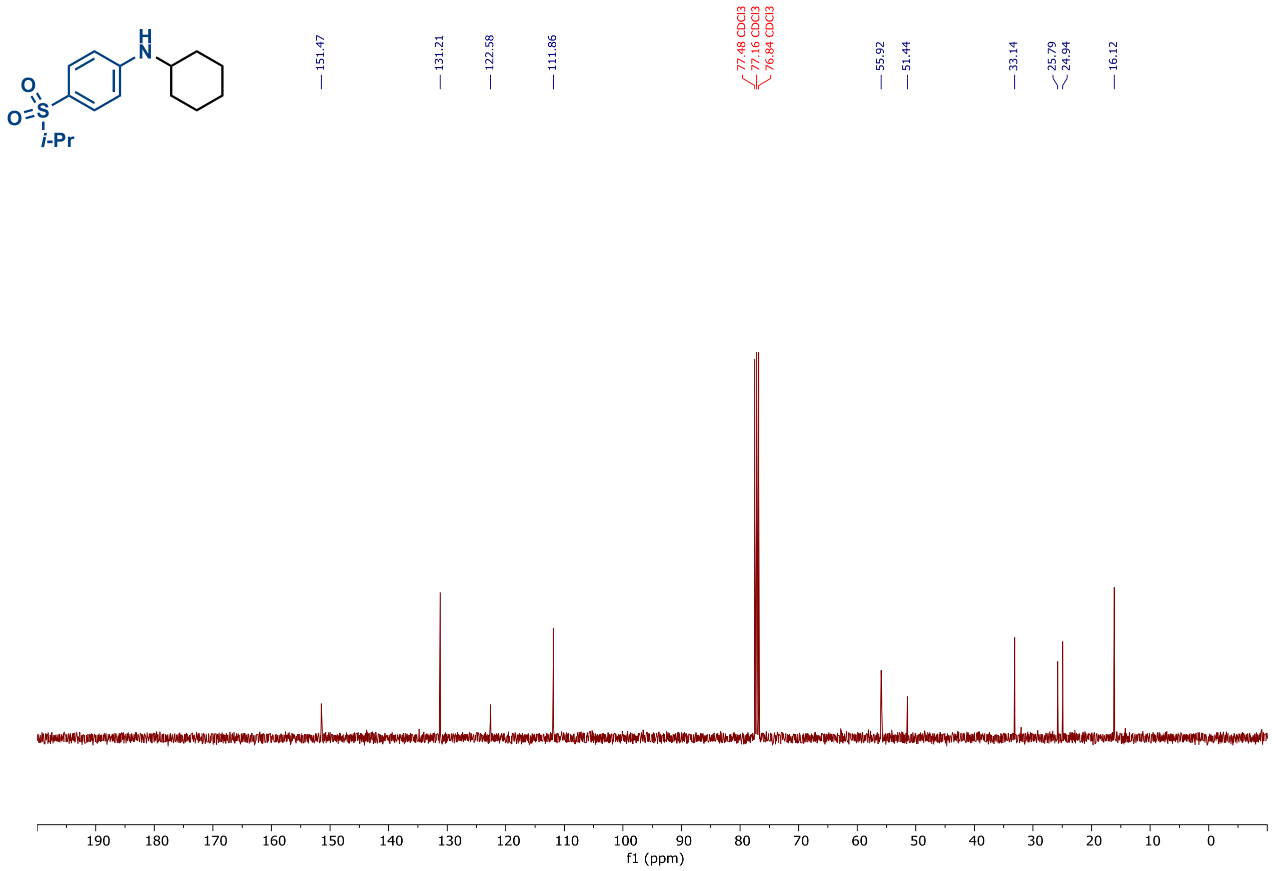

^{1}H NMR (400 MHz, $CDCl_3$) of **38** (*see procedure*)

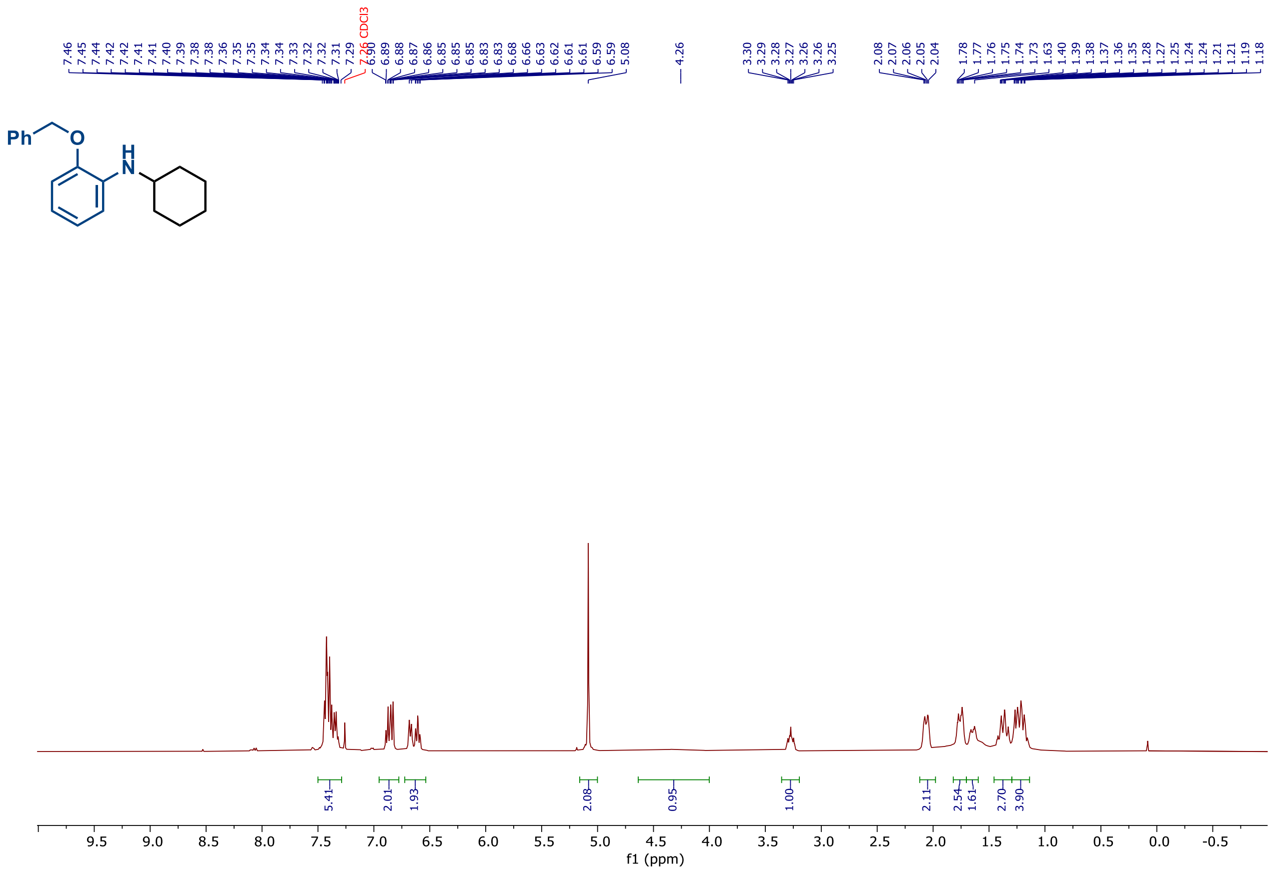

^{13}C NMR (101 MHz, $CDCl_3$) of **38**

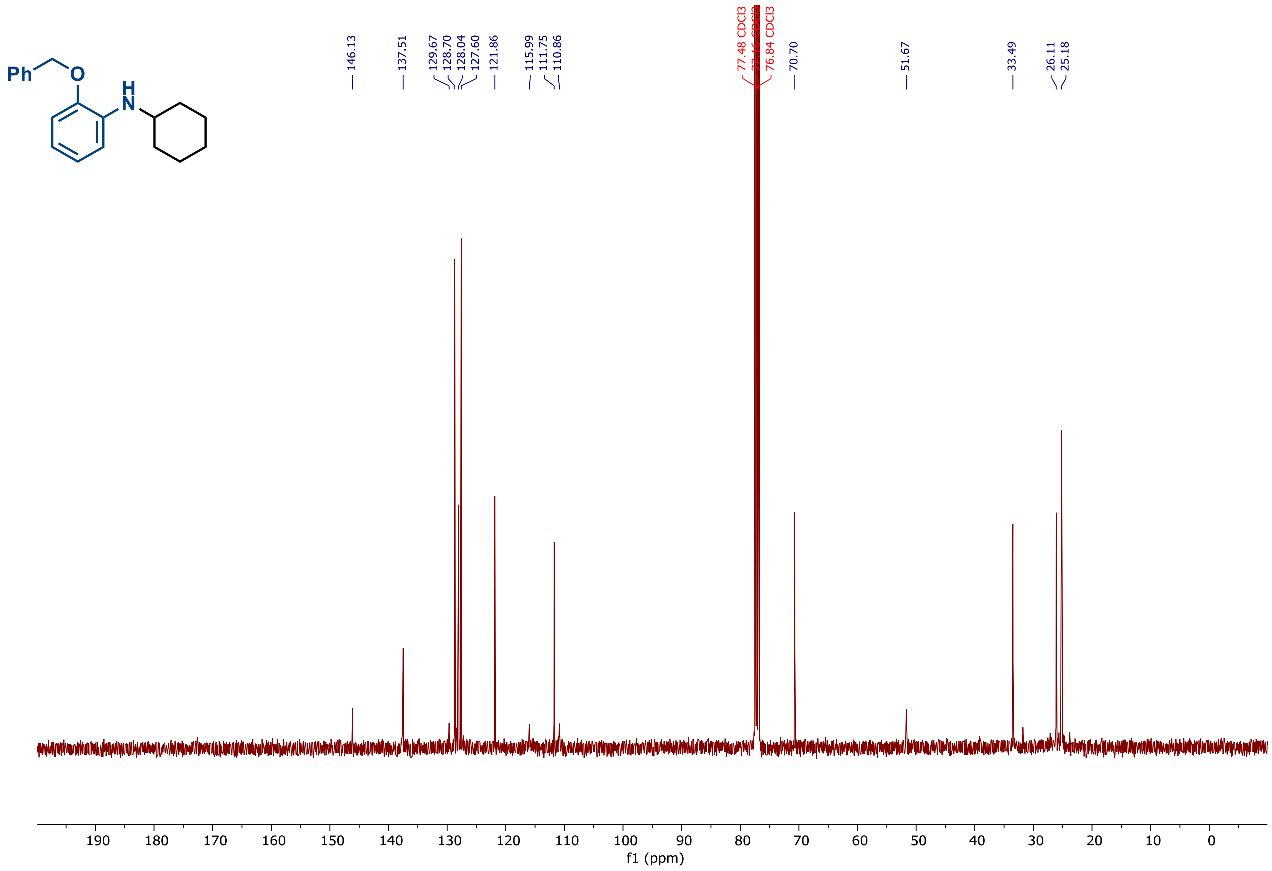

^{1}H NMR (400 MHz, $CDCl_3$) of **39** (<u>*see procedure*</u>)

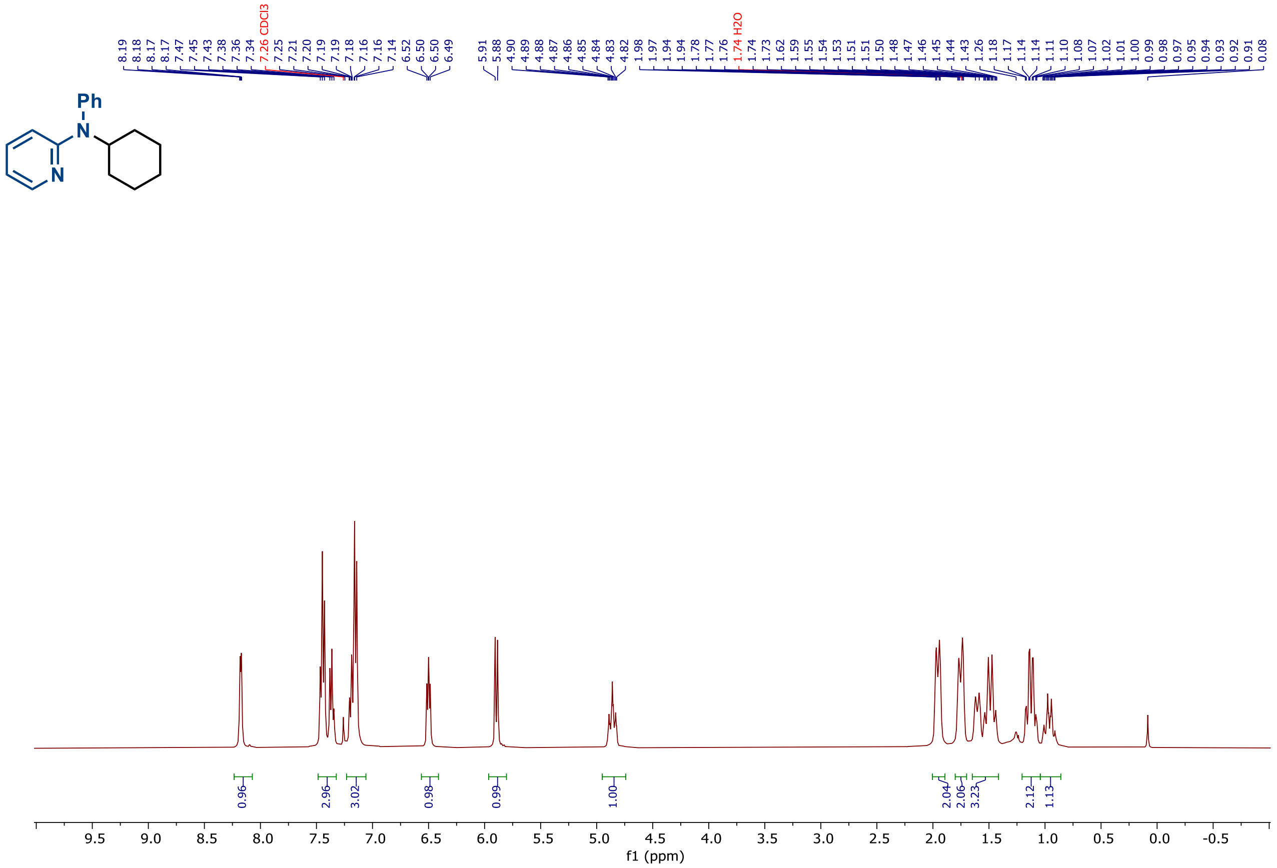

^{13}C NMR (101 MHz, $CDCl_3$) of **39**

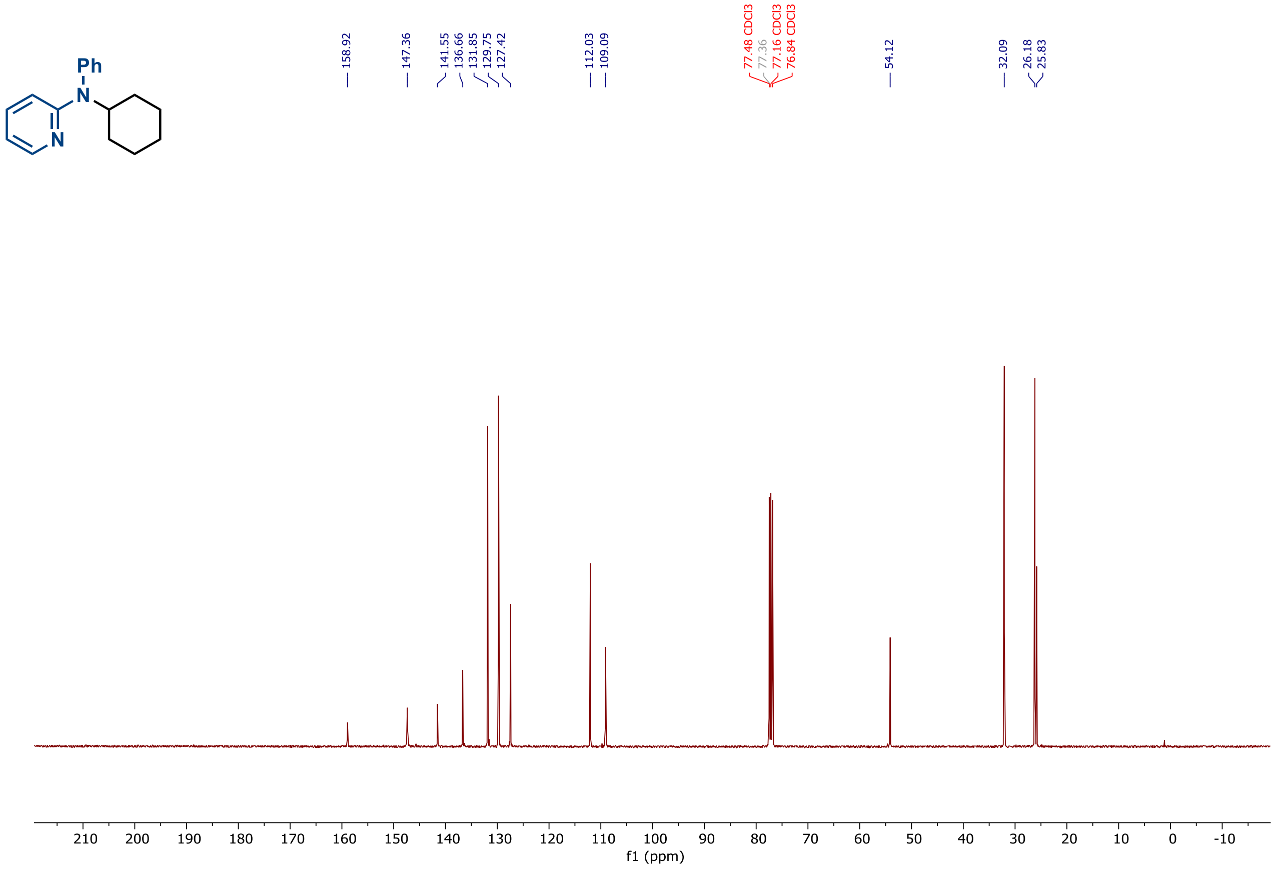

^{1}H NMR (400 MHz, $CDCl_3$) of **40** (*see procedure*)

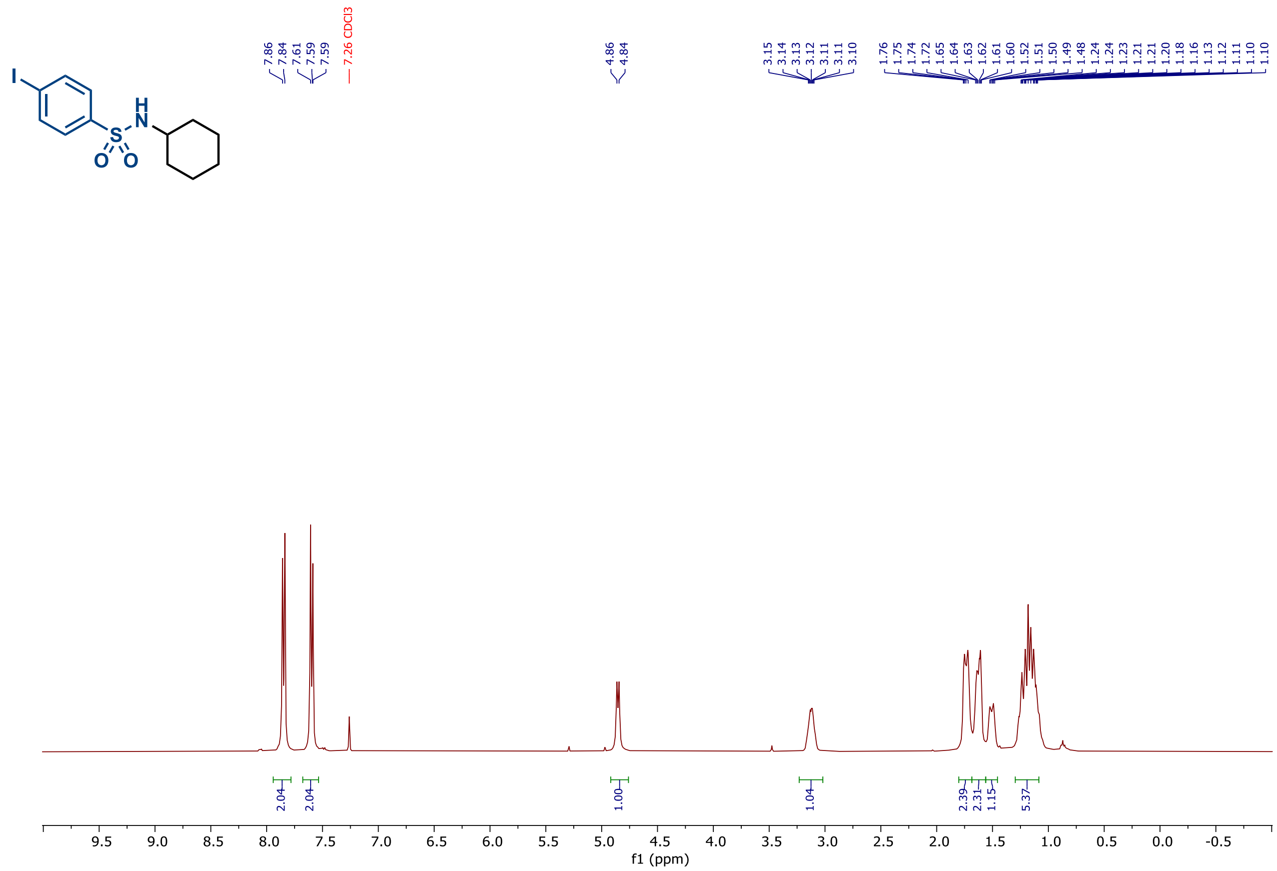

^{13}C NMR (101 MHz, $CDCl_3$) of **40**

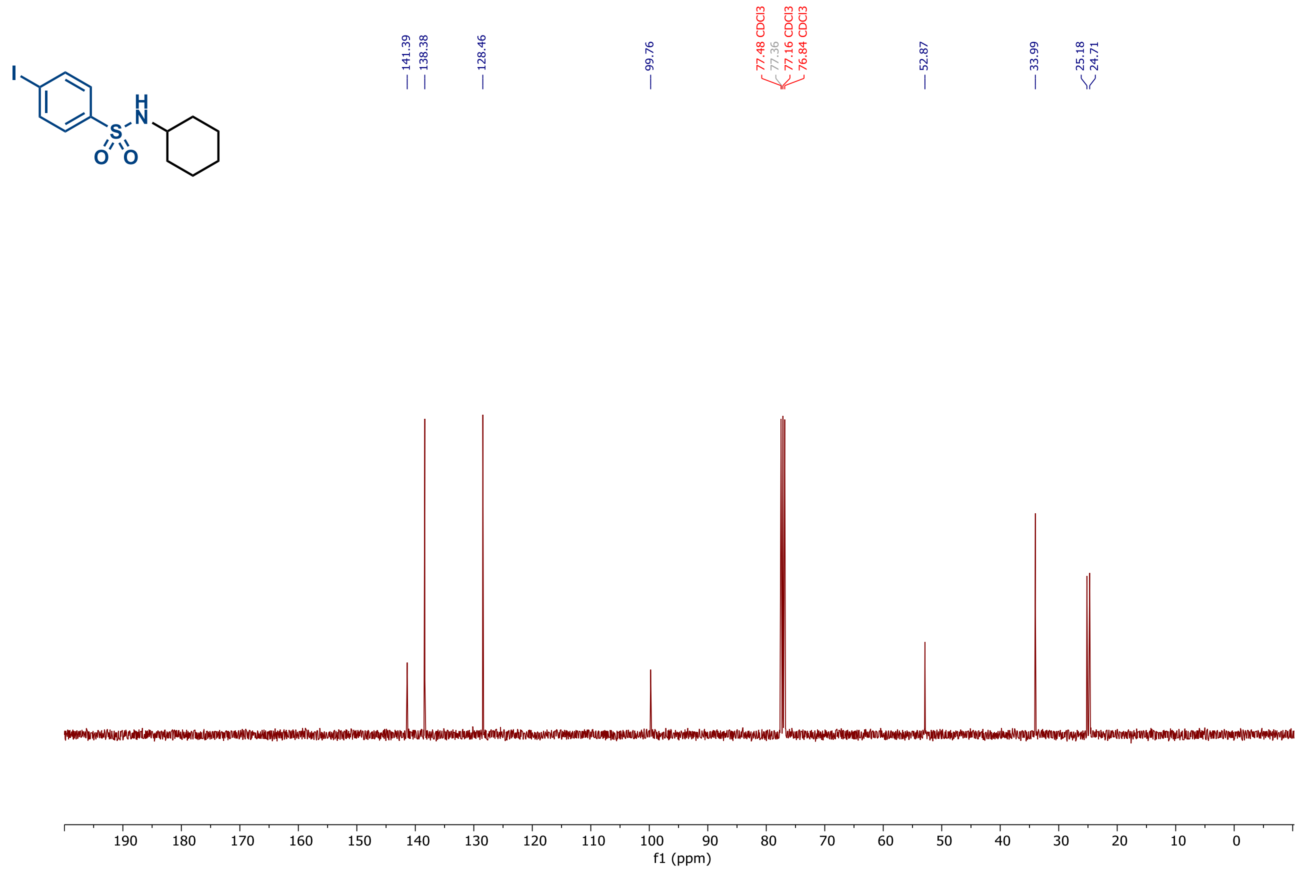

^{1}H NMR (400 MHz, $CDCl_3$) of **41** (*see procedure*)

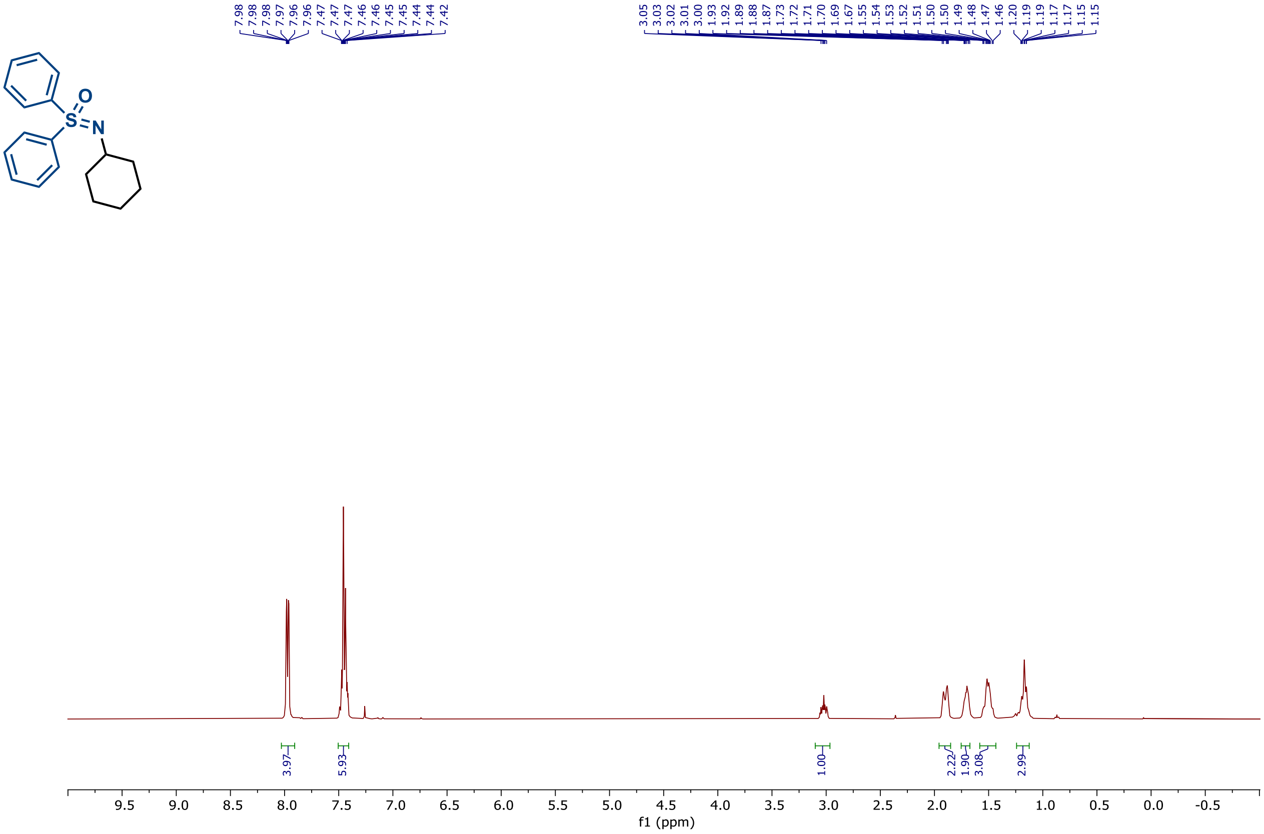

^{13}C NMR (101 MHz, $CDCl_3$) of **41**

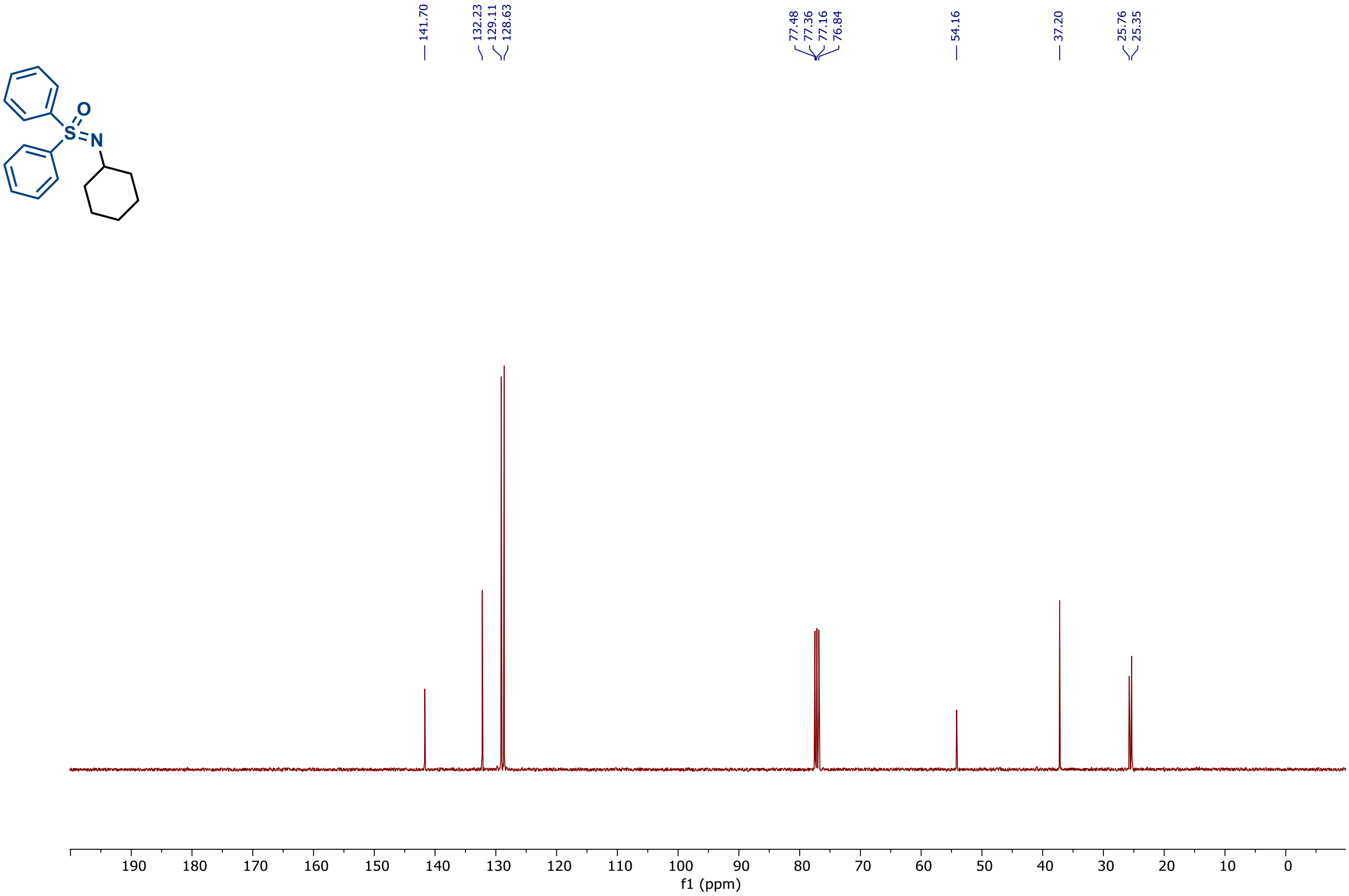

^{1}H NMR (400 MHz, $CDCl_3$) of **42** (see procedure)

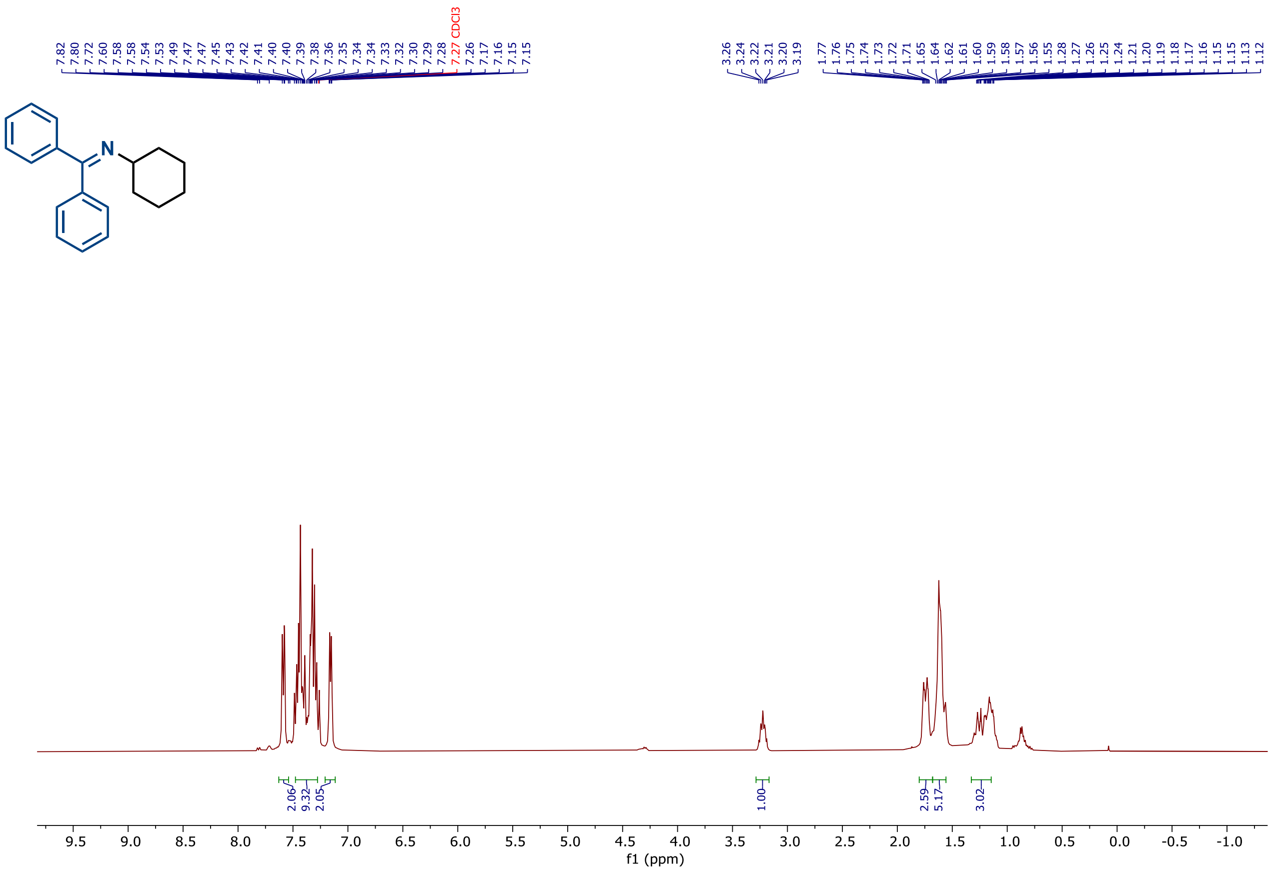

^{13}C NMR (101 MHz, $CDCl_3$) of **42**

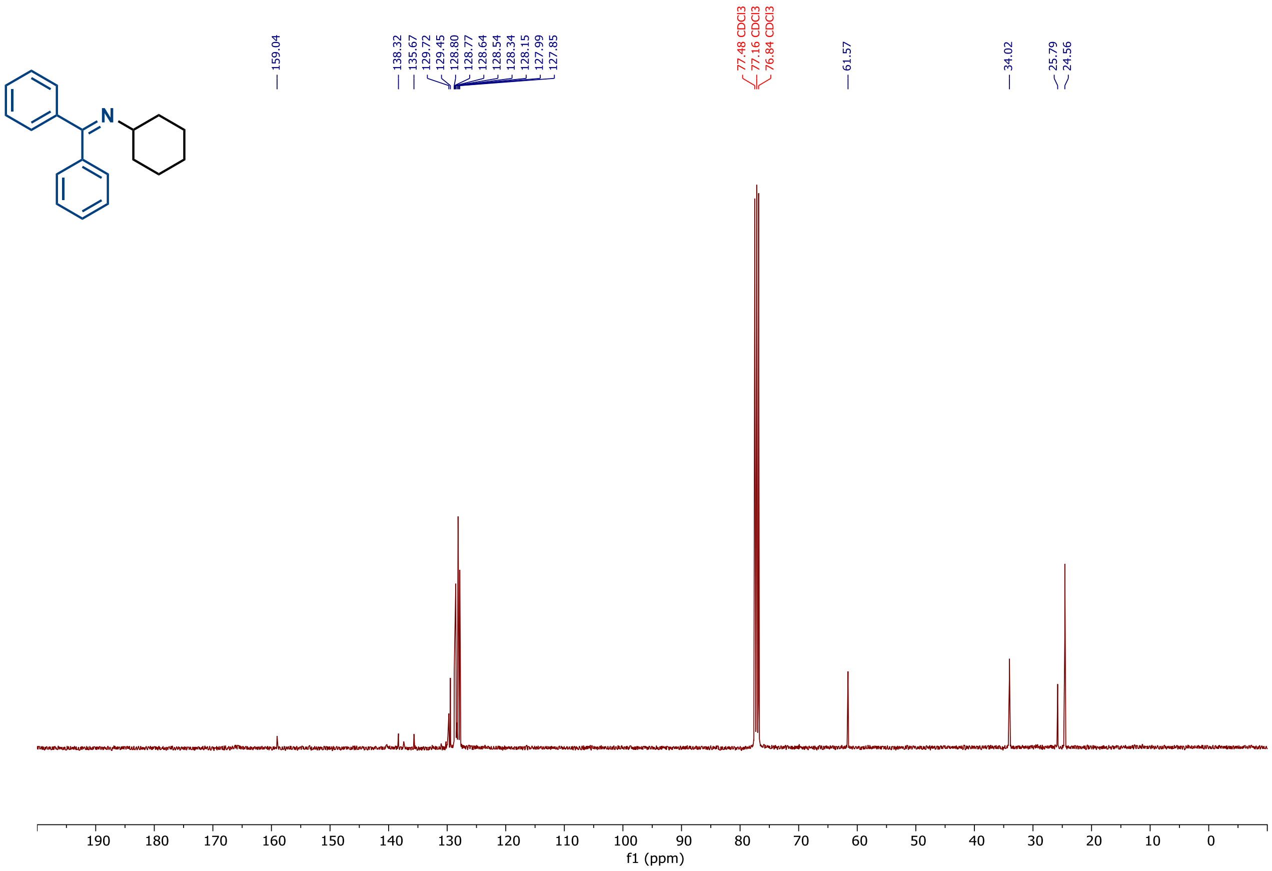

^{1}H NMR (400 MHz, $CDCl_3$) of **43** (see procedure)

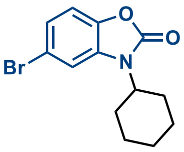

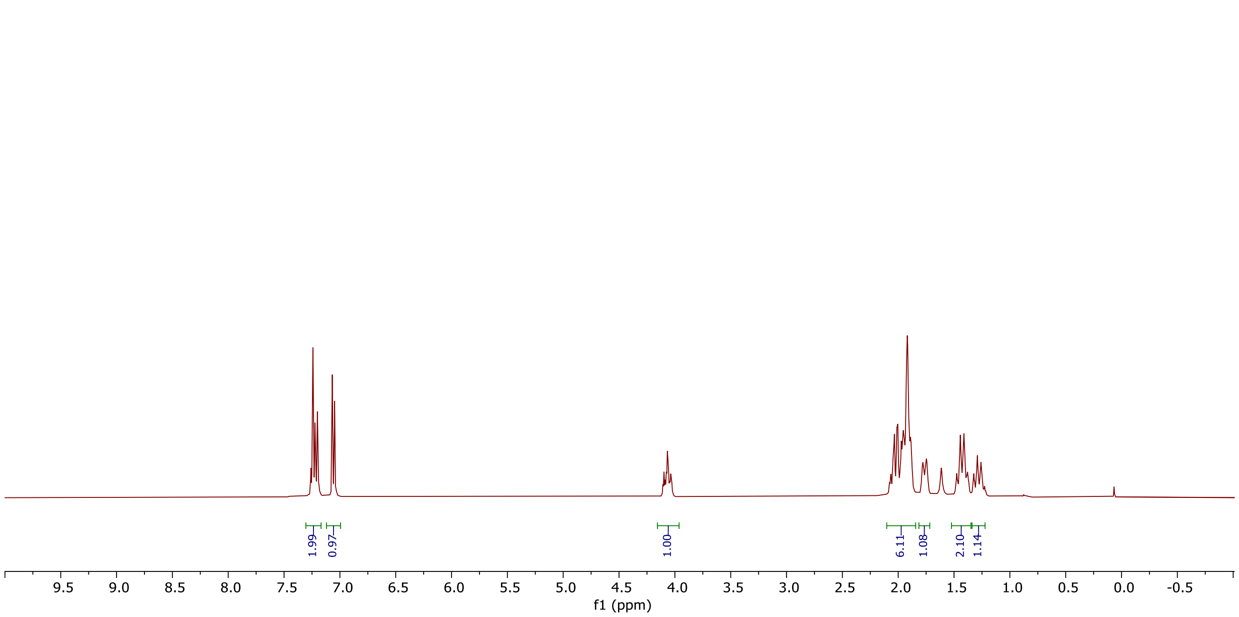

^{13}C NMR (101 MHz, $CDCl_3$) of **43**

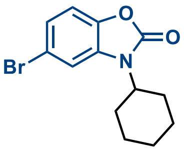

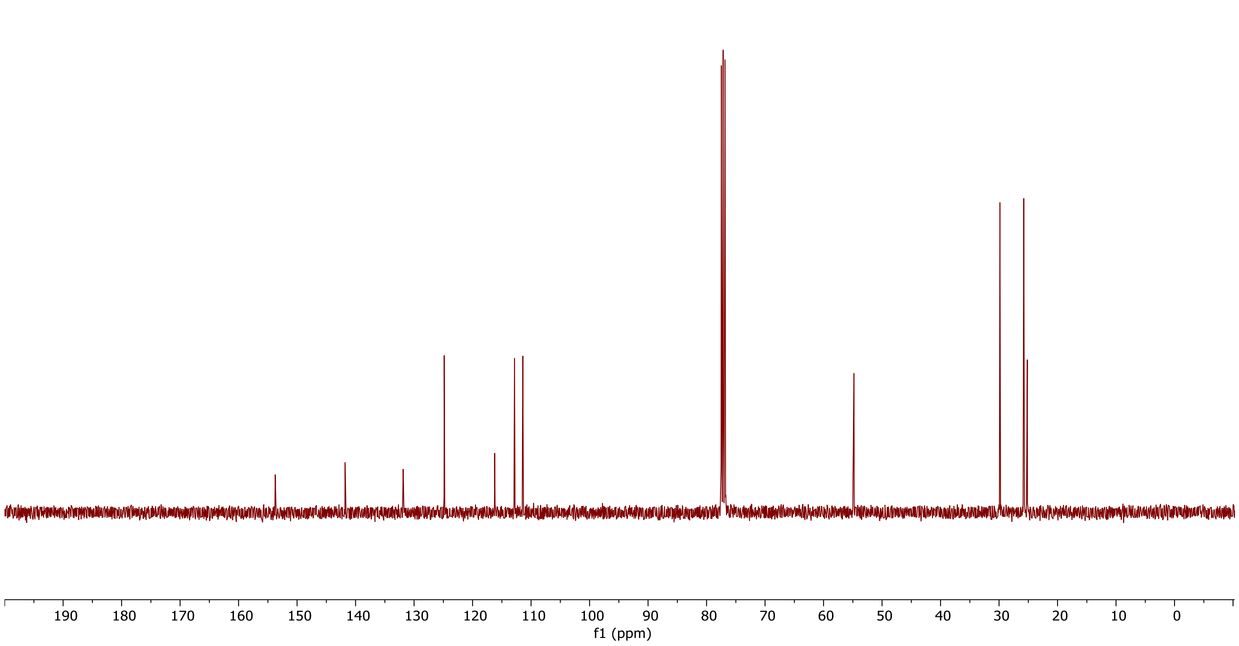

^{1}H NMR (400 MHz, $CDCl_3$) of **44** (*see procedure*)

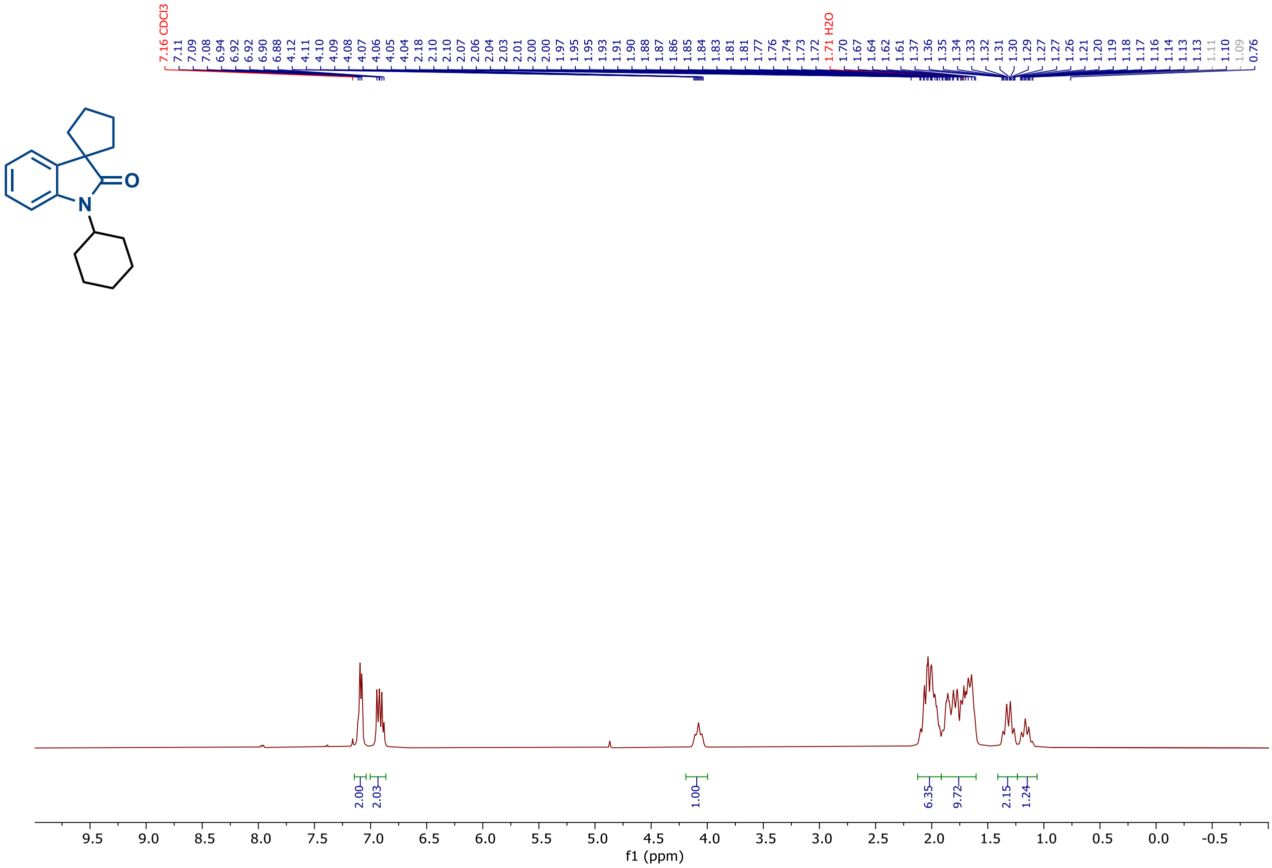

^{13}C NMR (101 MHz, $CDCl_3$) of **44**

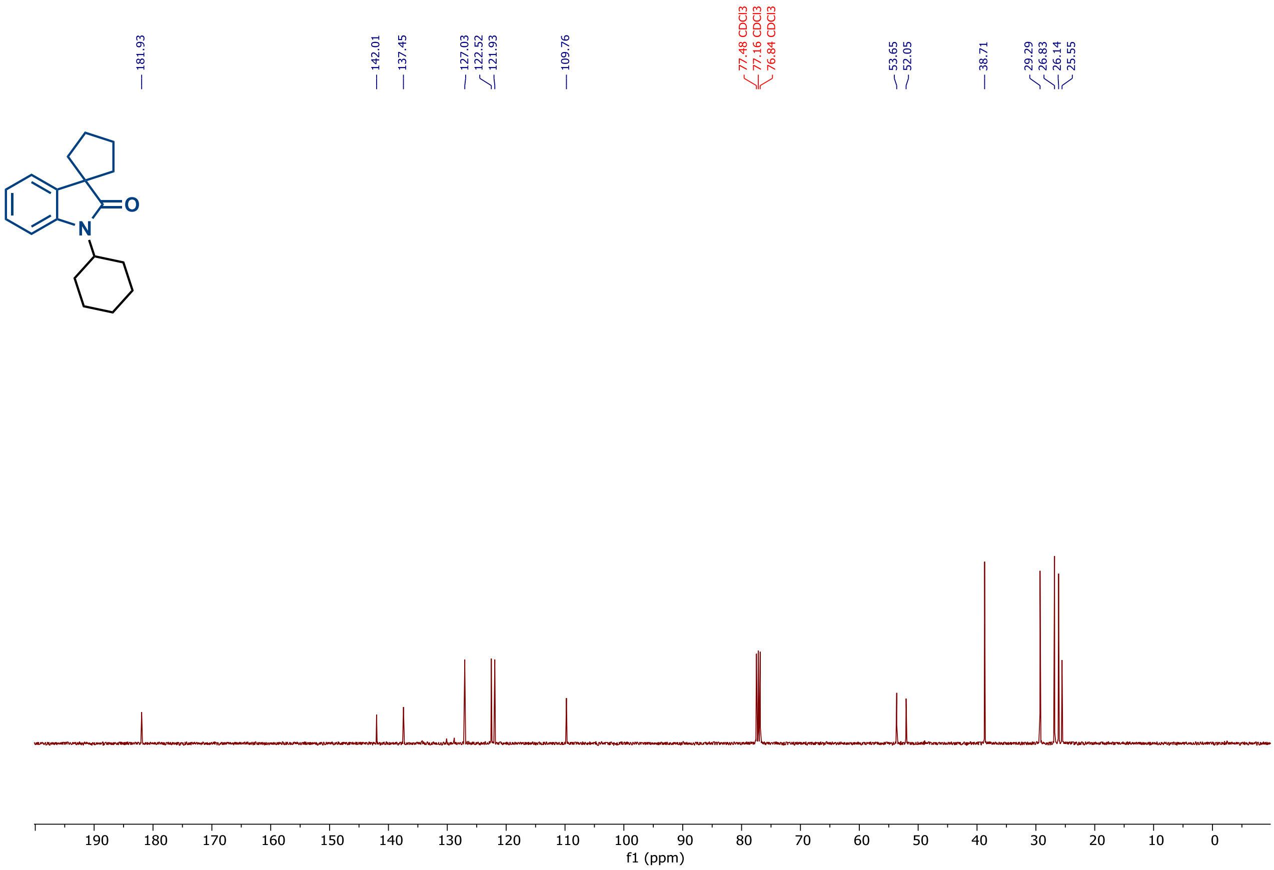

^{1}H NMR (400 MHz, $CDCl_3$) of **45** (see procedure)

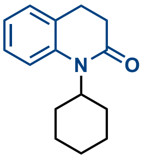

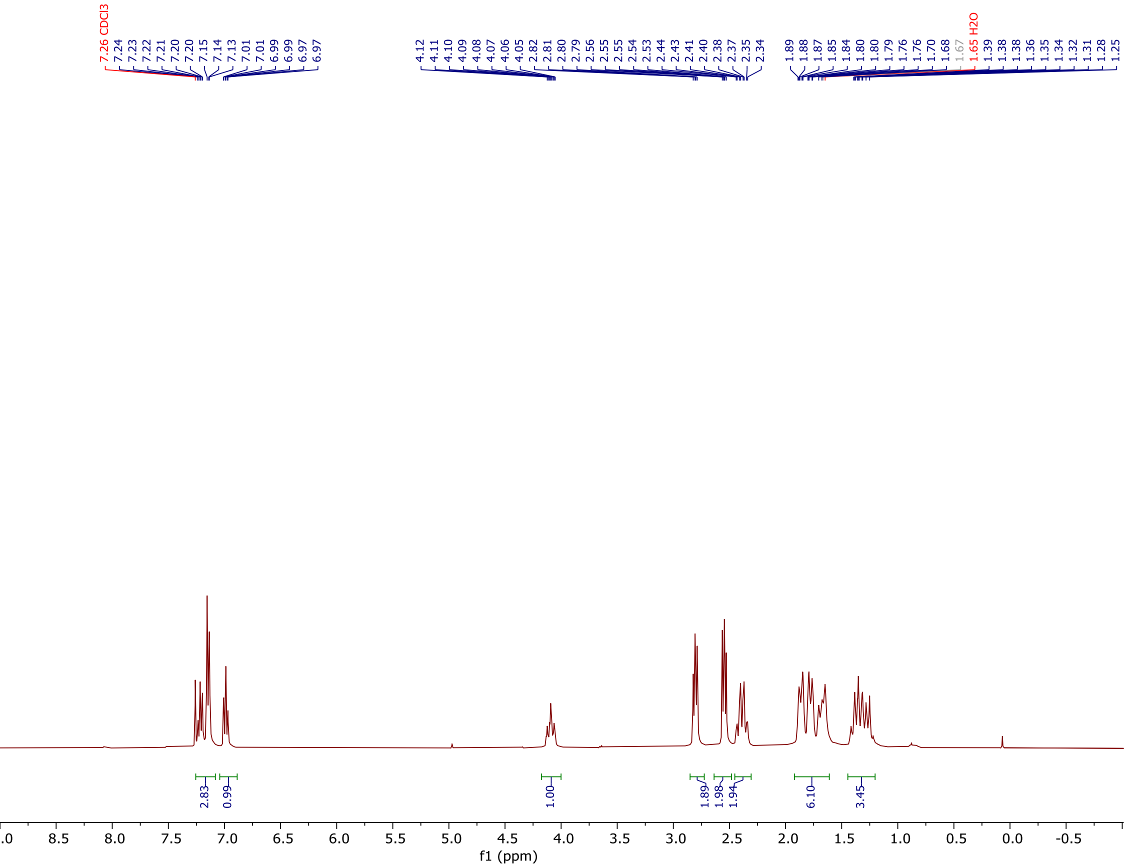

^{13}C NMR (101 MHz, $CDCl_3$) of **45**

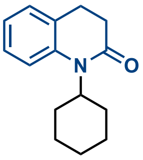

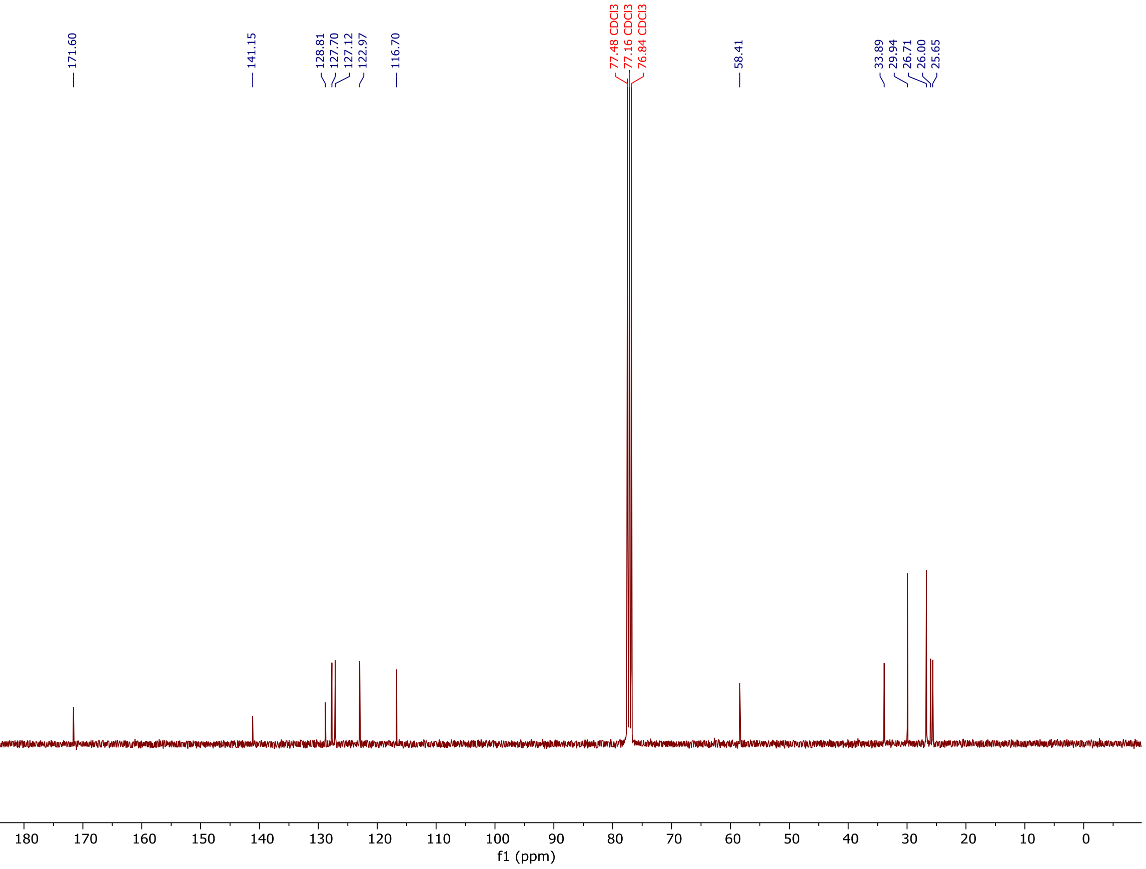

^{1}H NMR (400 MHz, $CDCl_3$) of **46** (see procedure)

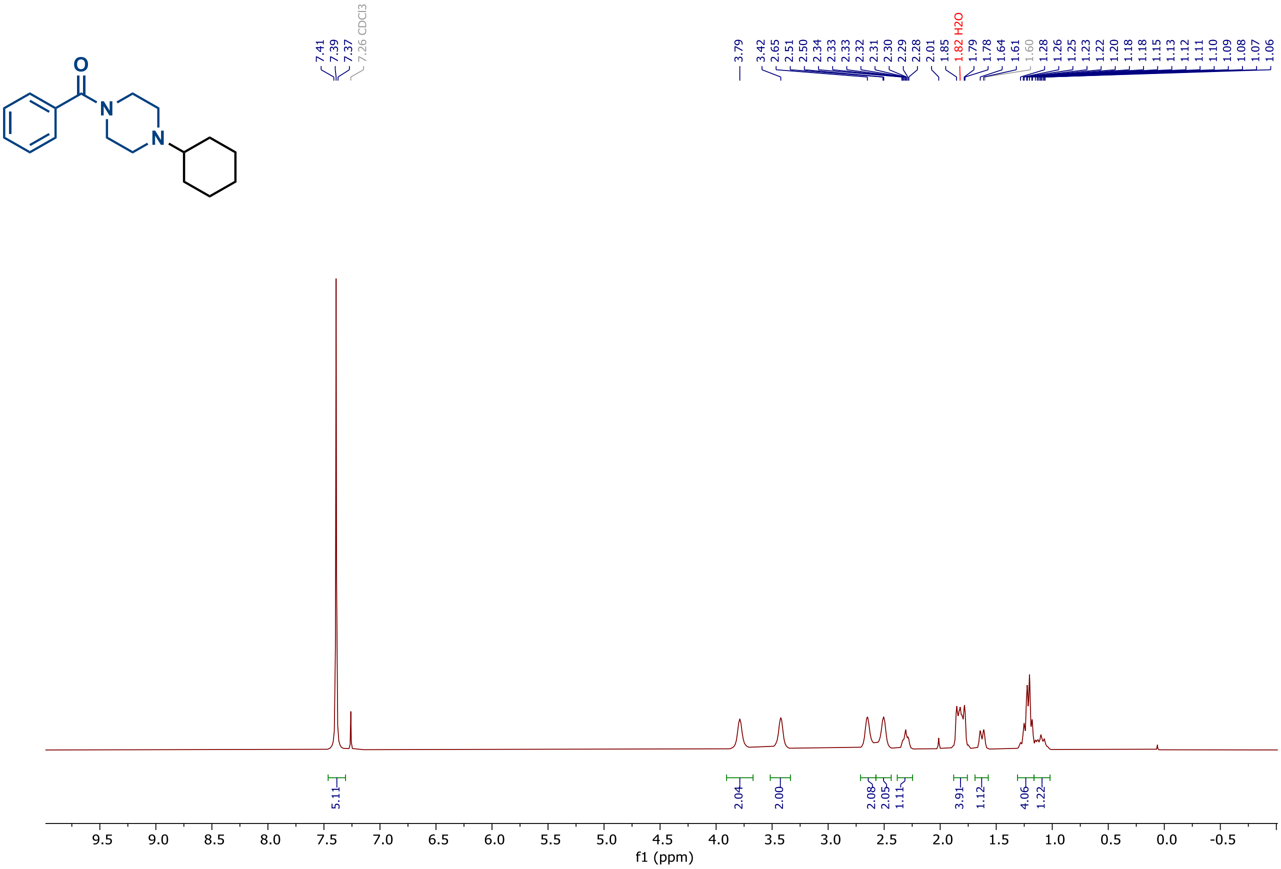

^{13}C NMR (101 MHz, $CDCl_3$) of **46**

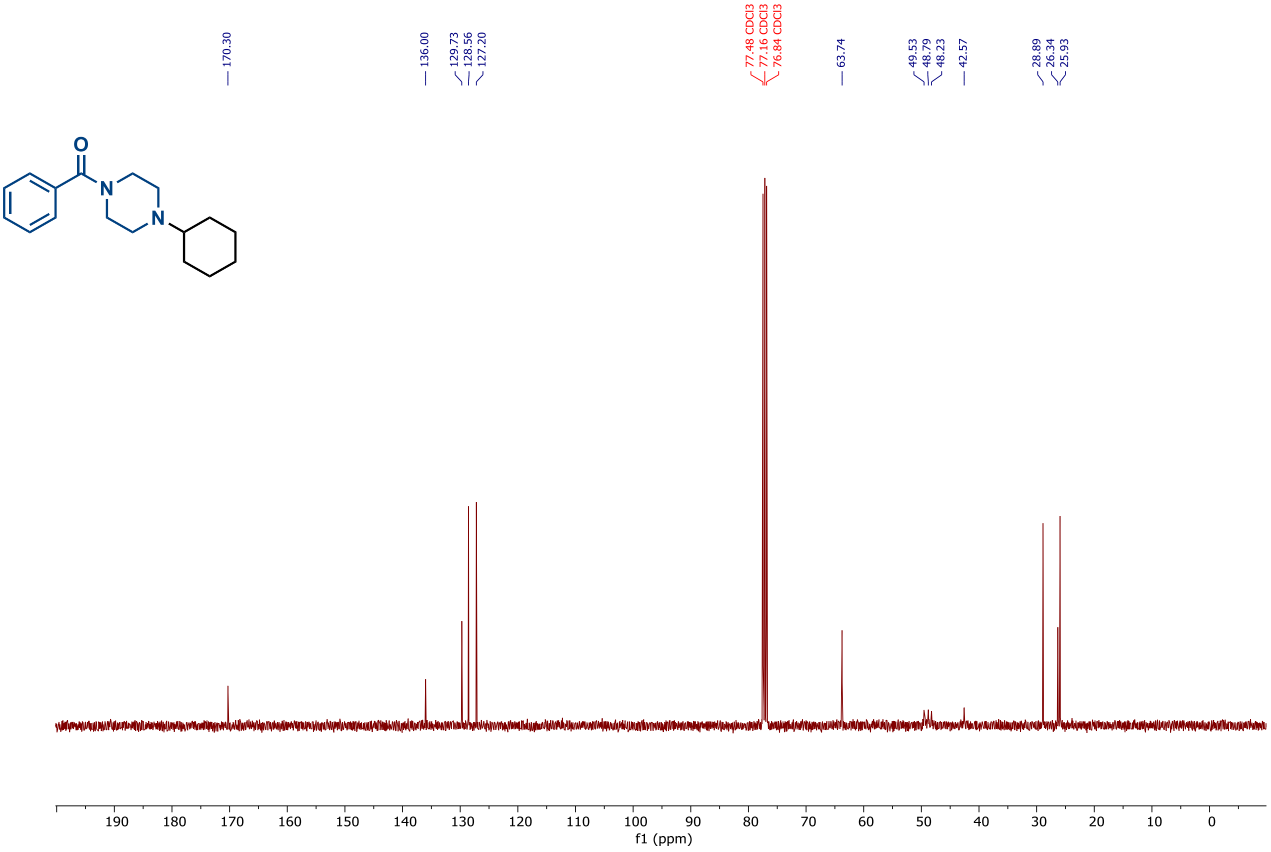

^{1}H NMR (400 MHz, $CDCl_3$) of **47** (*see procedure*)

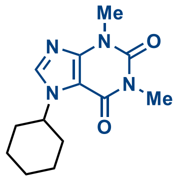

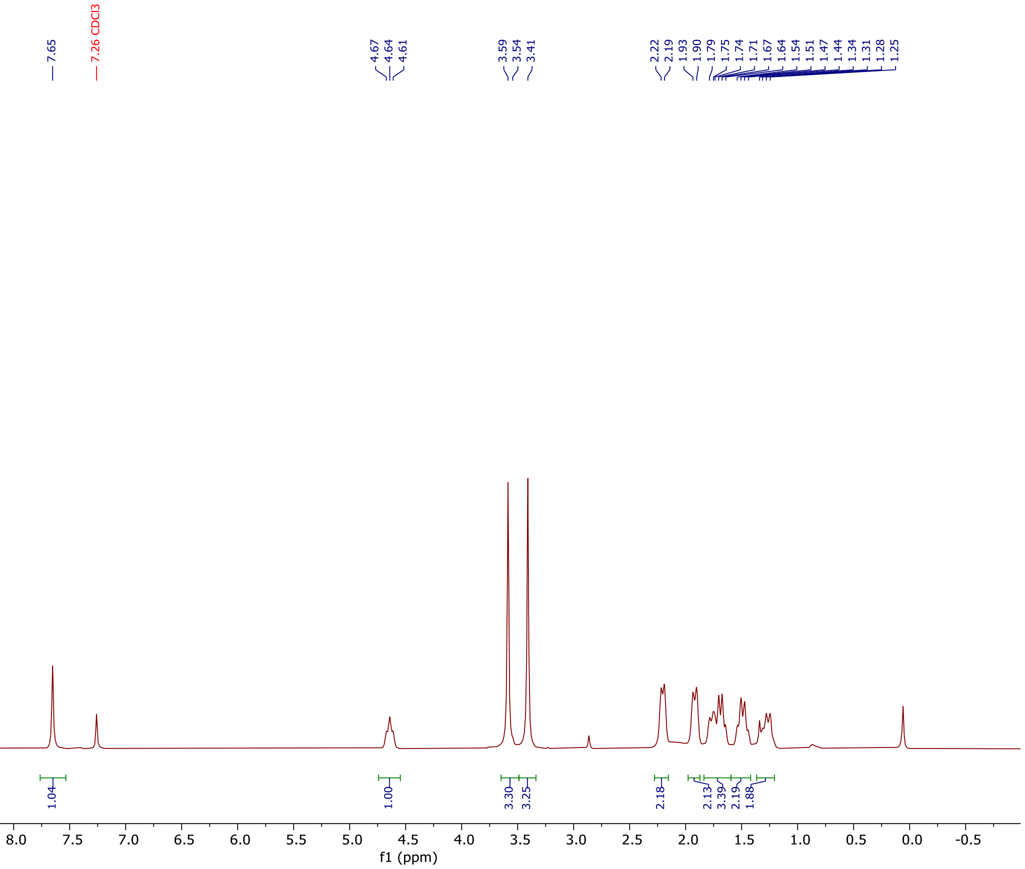

^{13}C NMR (101 MHz, $CDCl_3$) of **47**

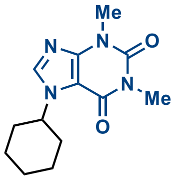

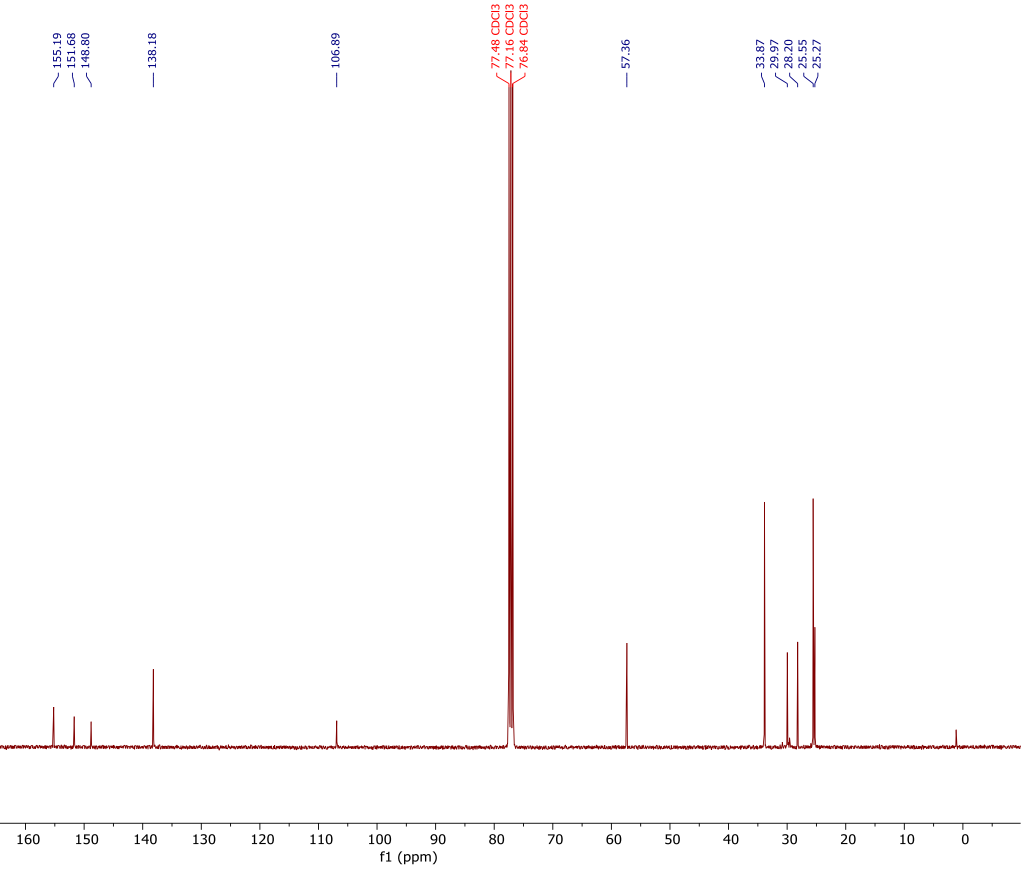

^{1}H NMR (400 MHz, CDCl3) of **48** (*see procedure*)

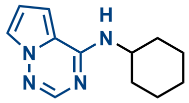

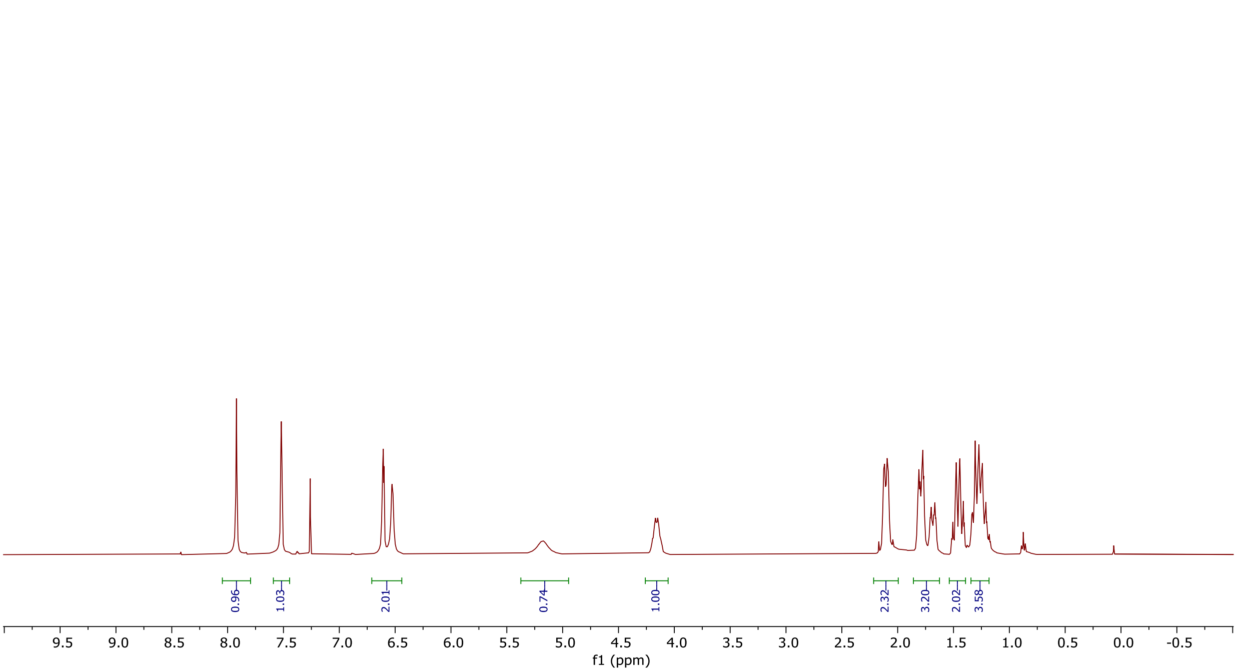

^{13}C NMR (101 MHz, CDCl3) of **48**

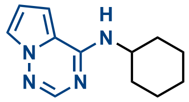

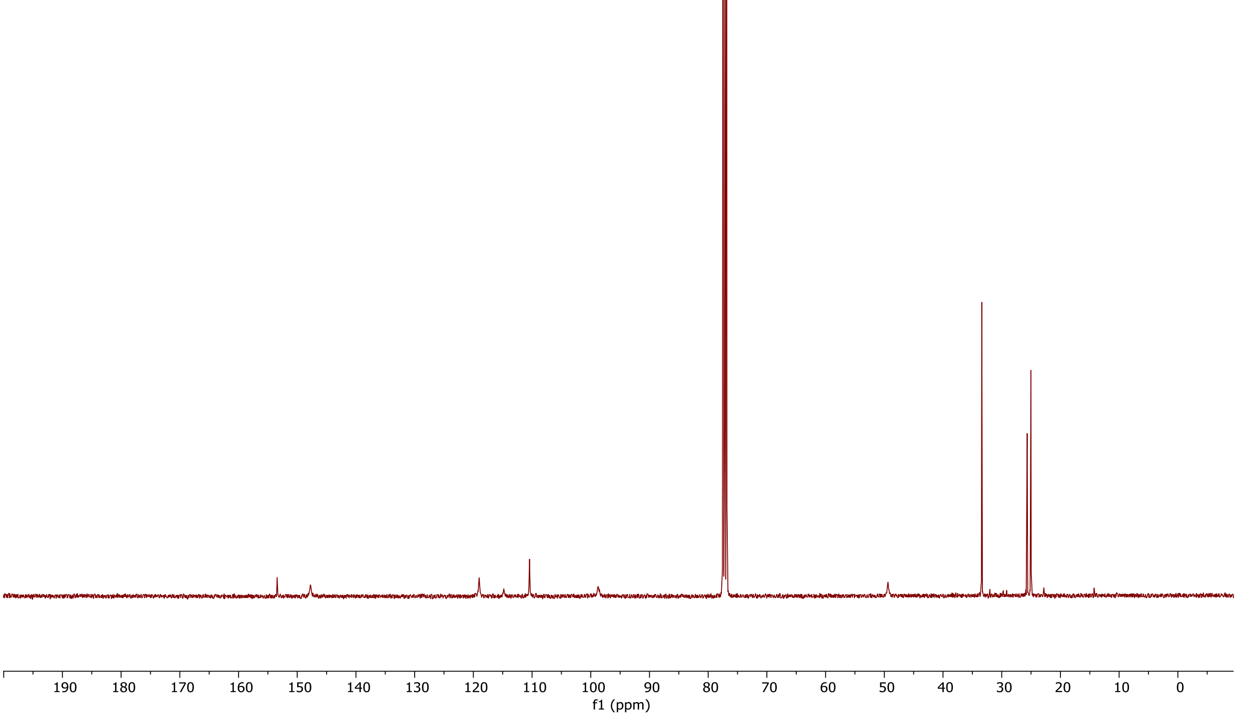

^{1}H NMR (400 MHz, $CDCl_3$) of **49** (*see procedure*)

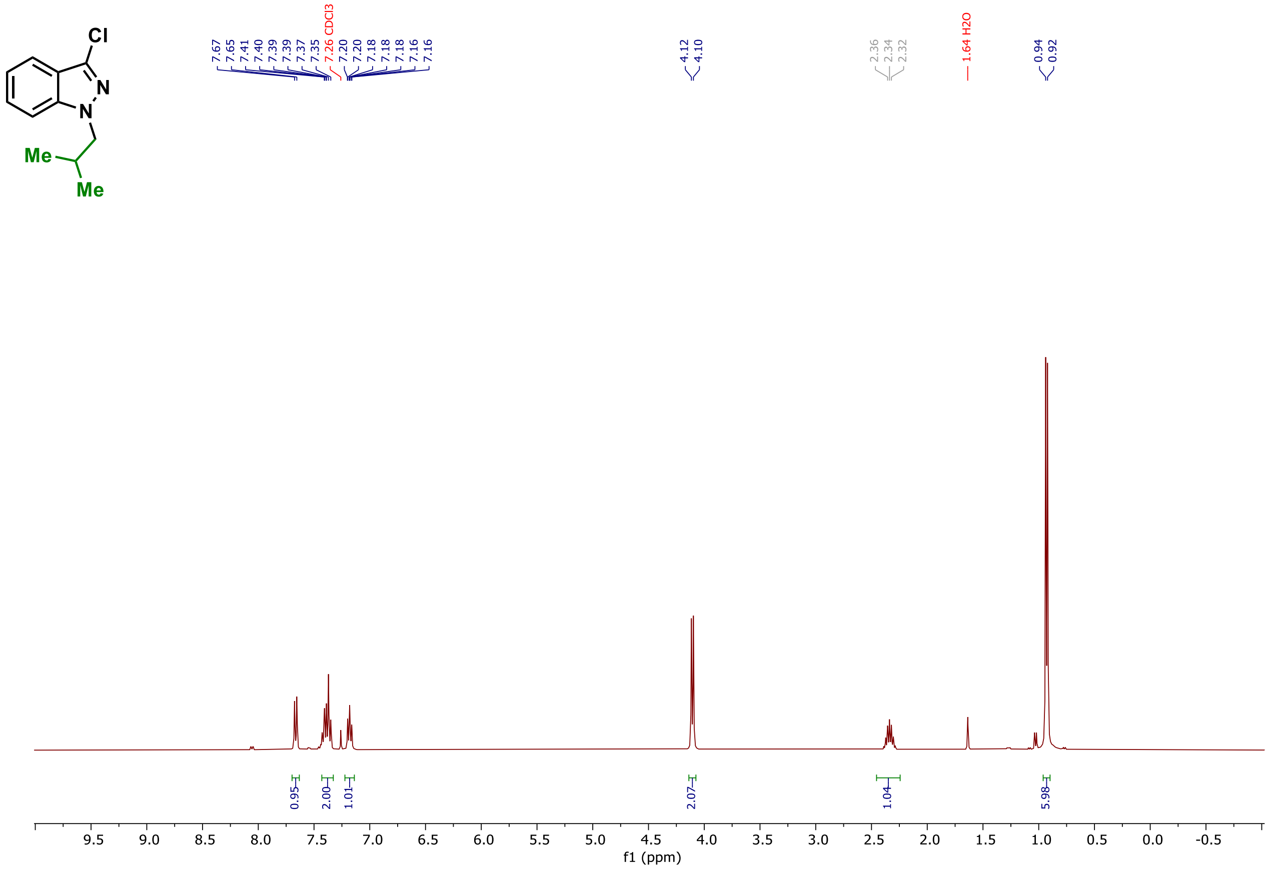

^{13}C NMR (101 MHz, $CDCl_3$) of **49**

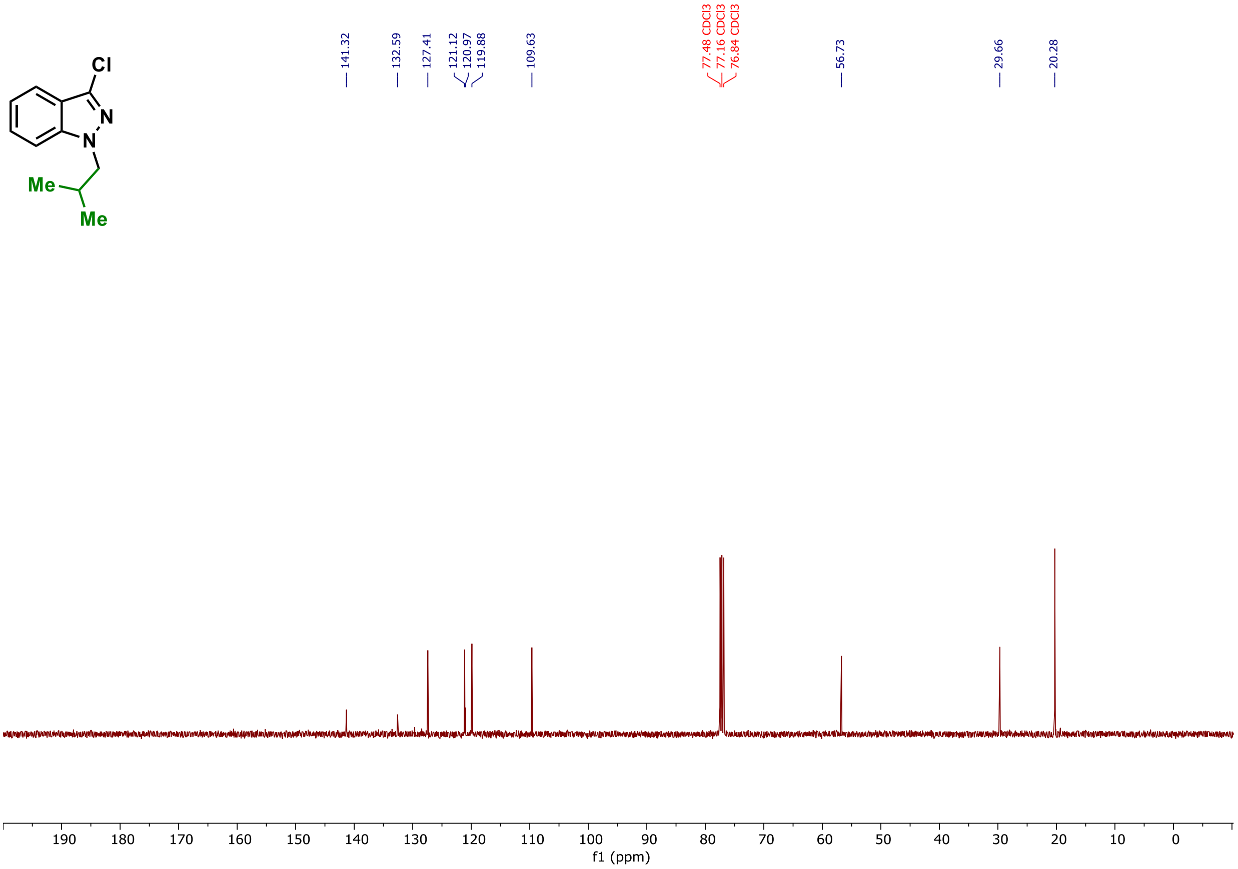

^{1}H NMR (400 MHz, $CDCl_3$) of **50** (*see procedure*)

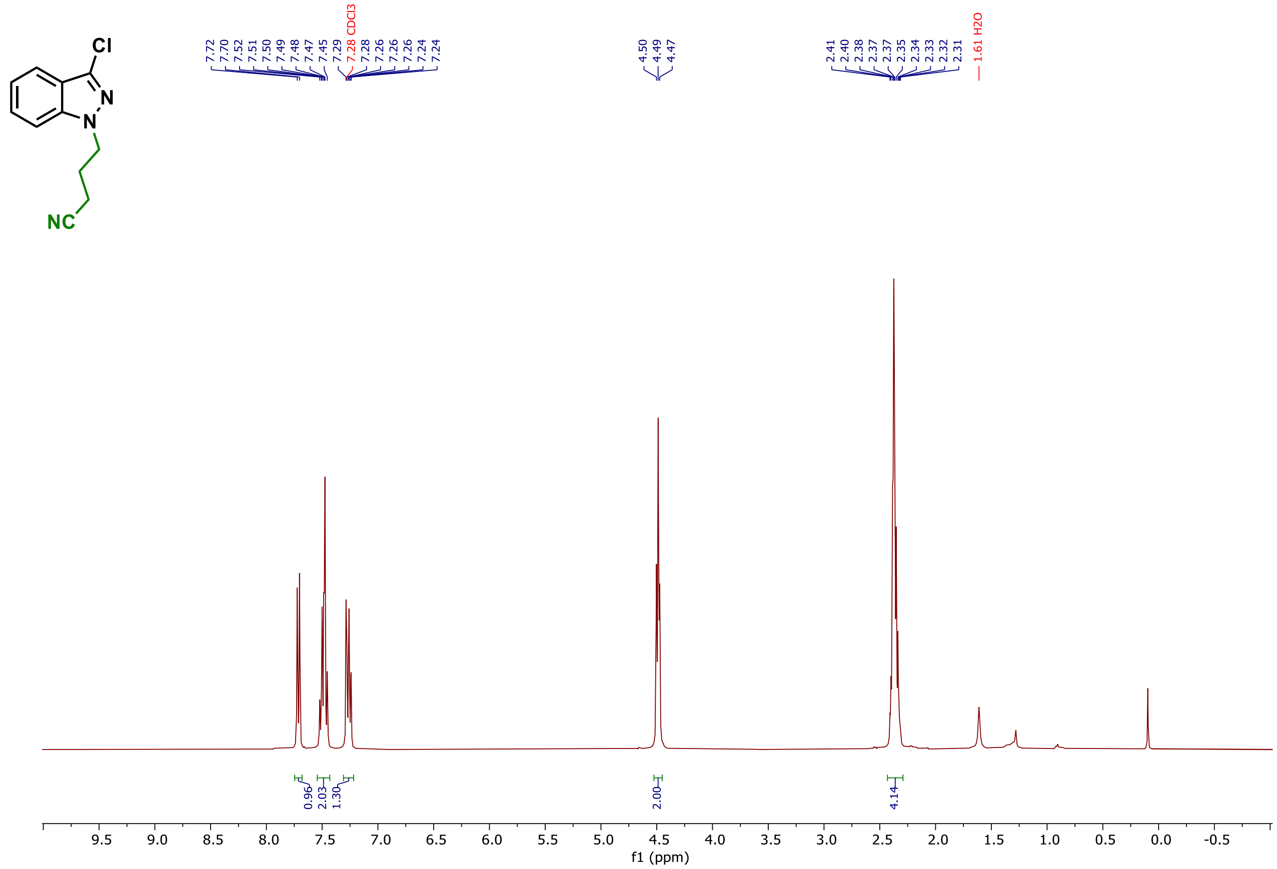

^{13}C NMR (101 MHz, $CDCl_3$) of **50**

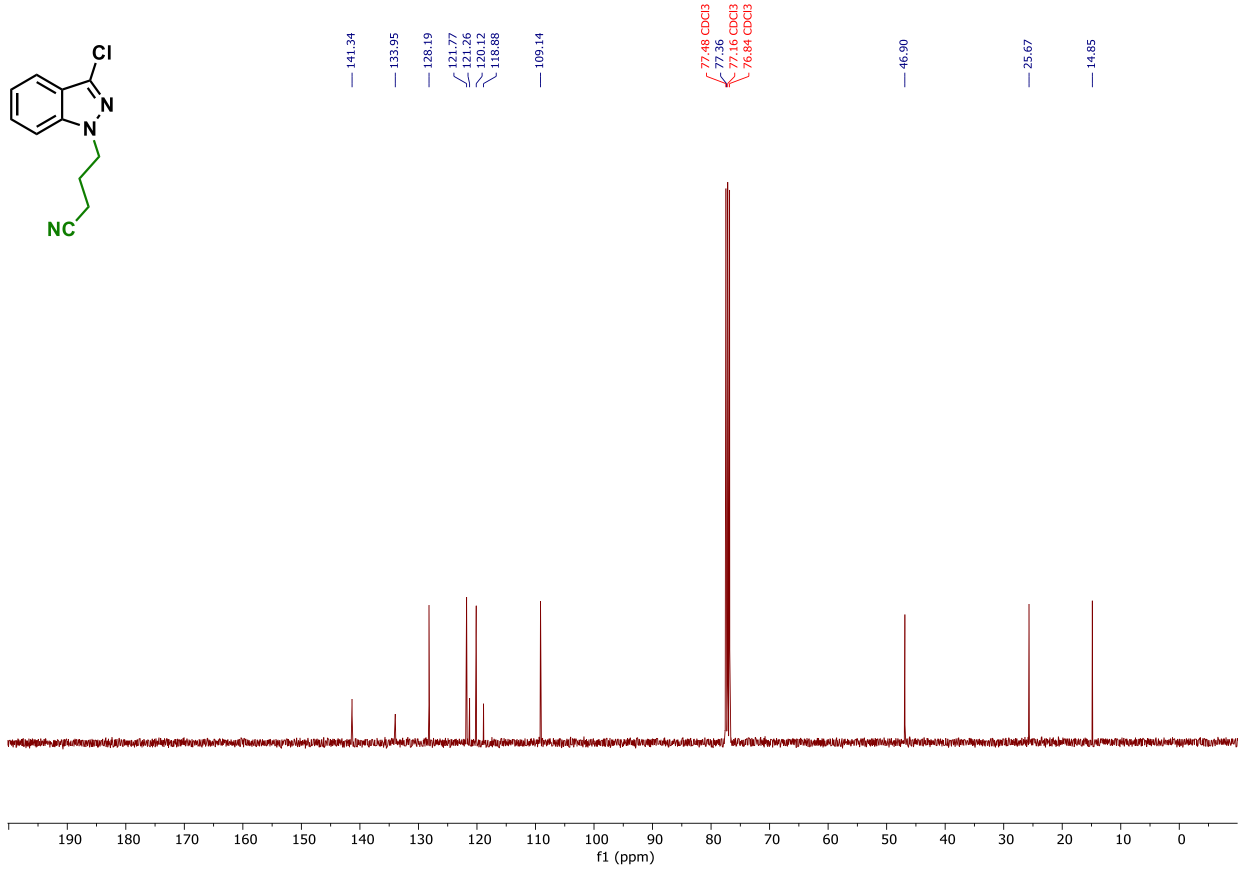

^{1}H NMR (400 MHz, $CDCl_3$) of **51** (*see procedure*)

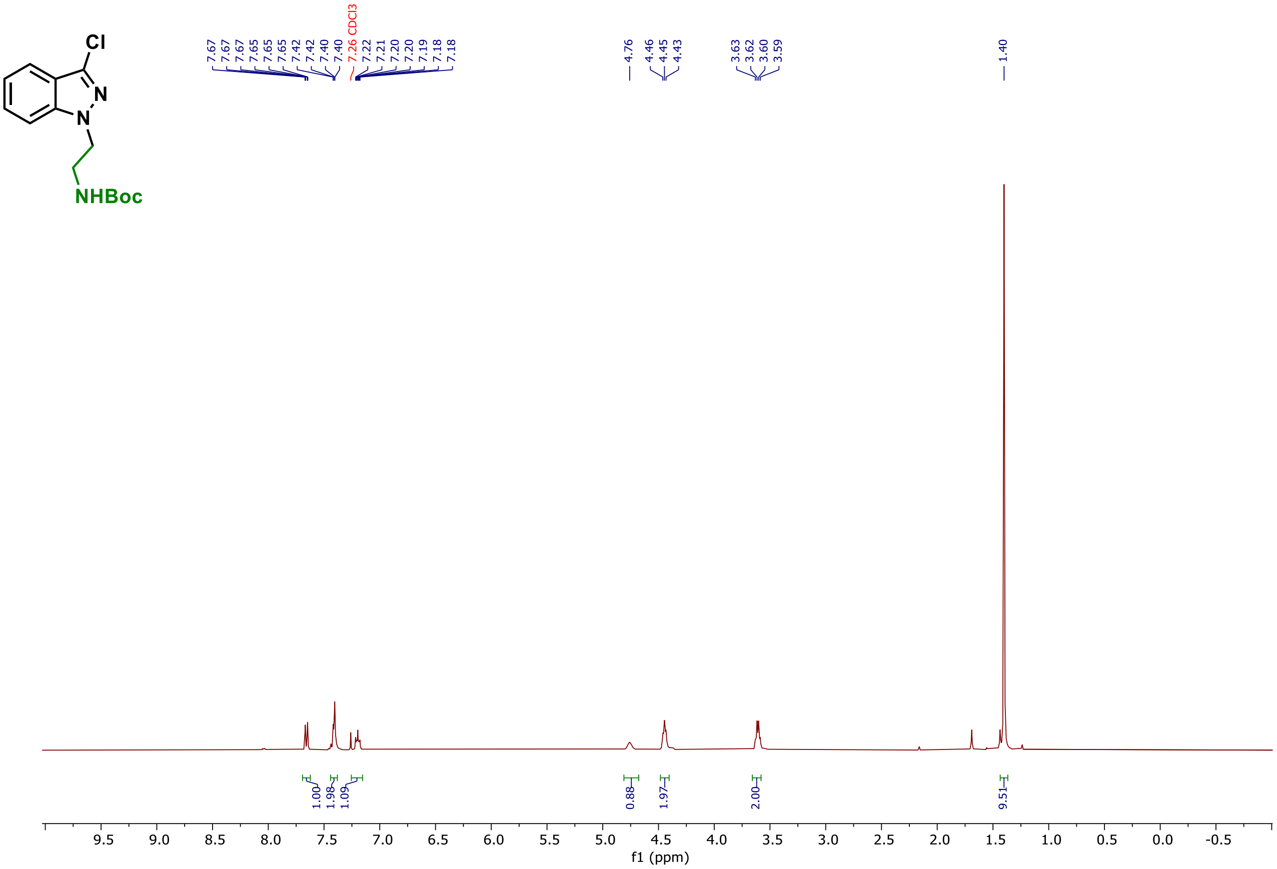

^{13}C NMR (101 MHz, $CDCl_3$) of **51**

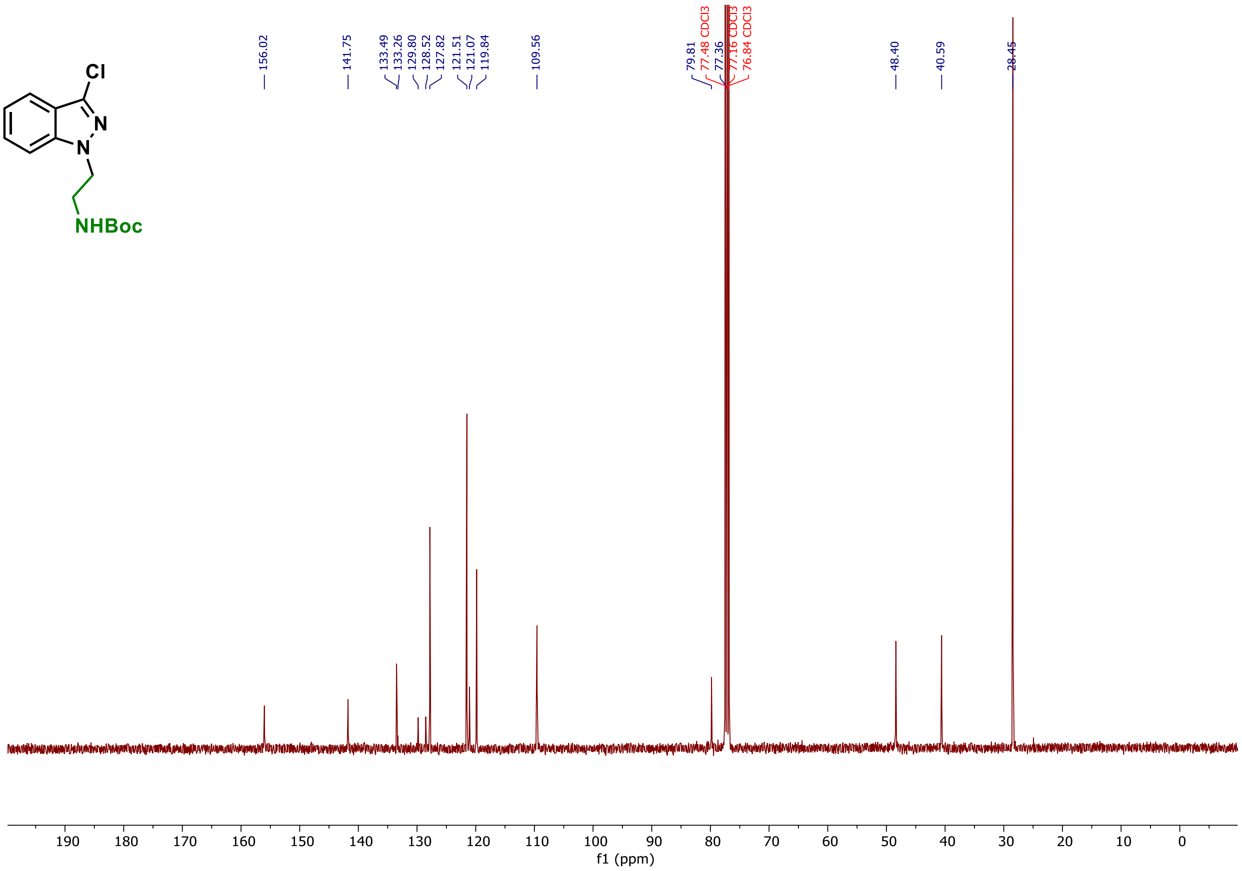

^{1}H NMR (400 MHz, CDCl3) of **52** (*see procedure*)

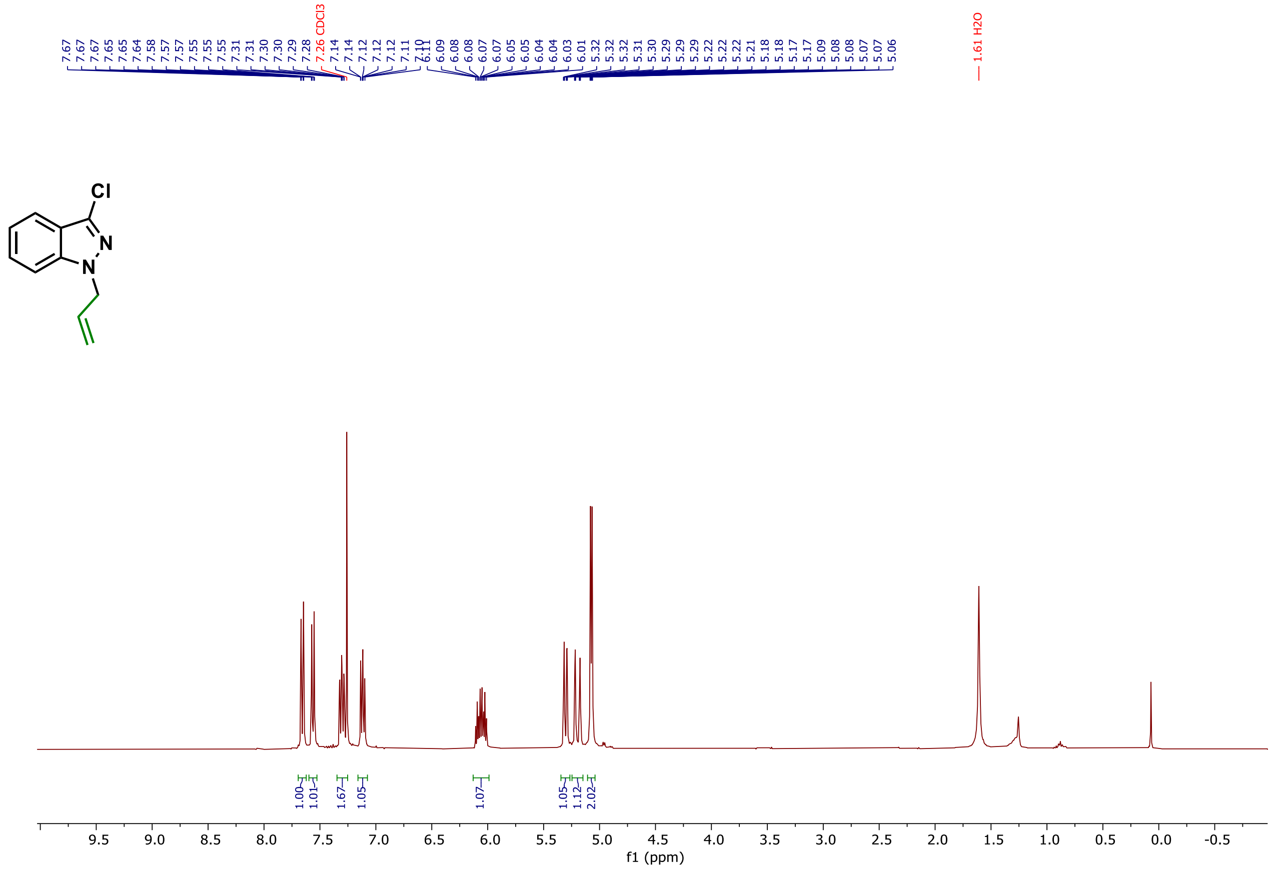

^{13}C NMR (101 MHz, CDCl3) of **52**

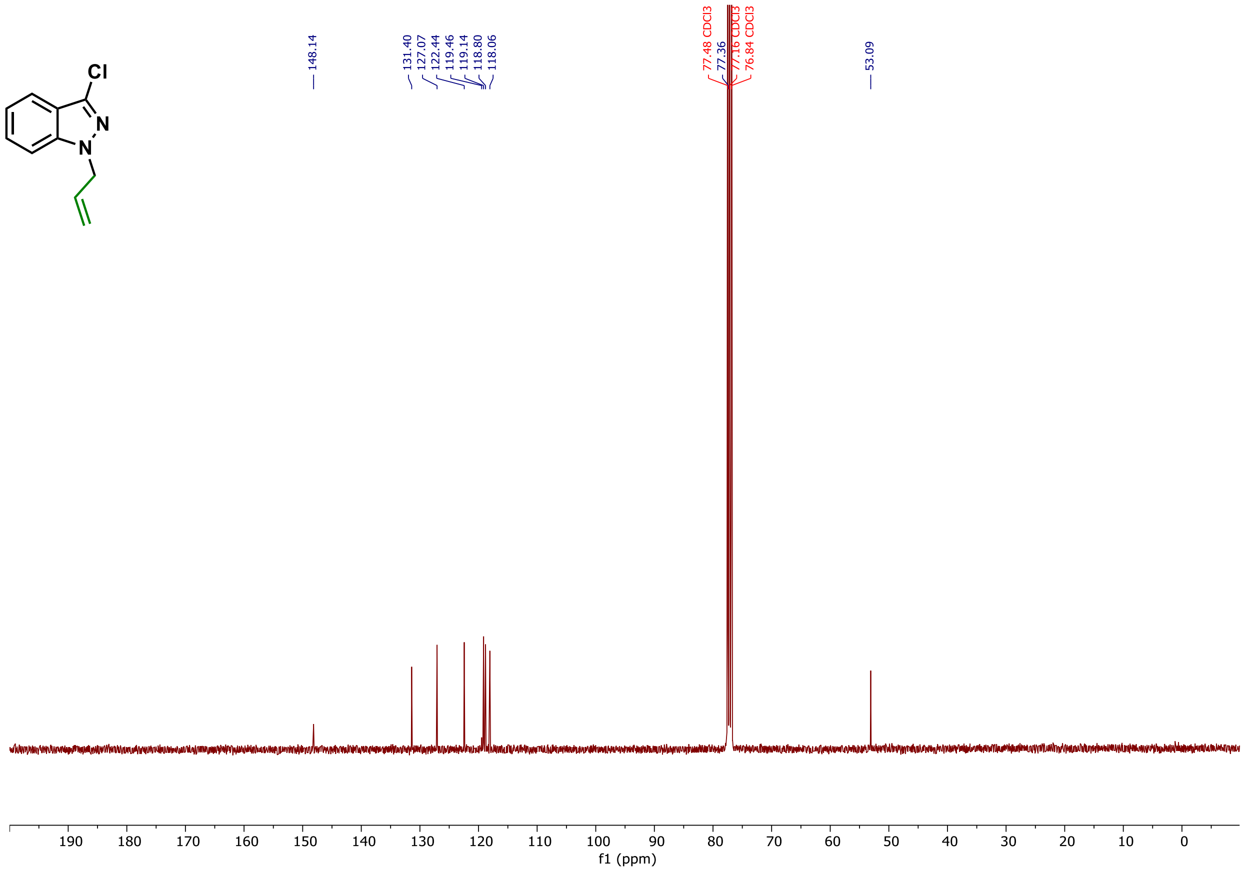

^{1}H NMR (400 MHz, $CDCl_3$) of **53** (*see procedure*)

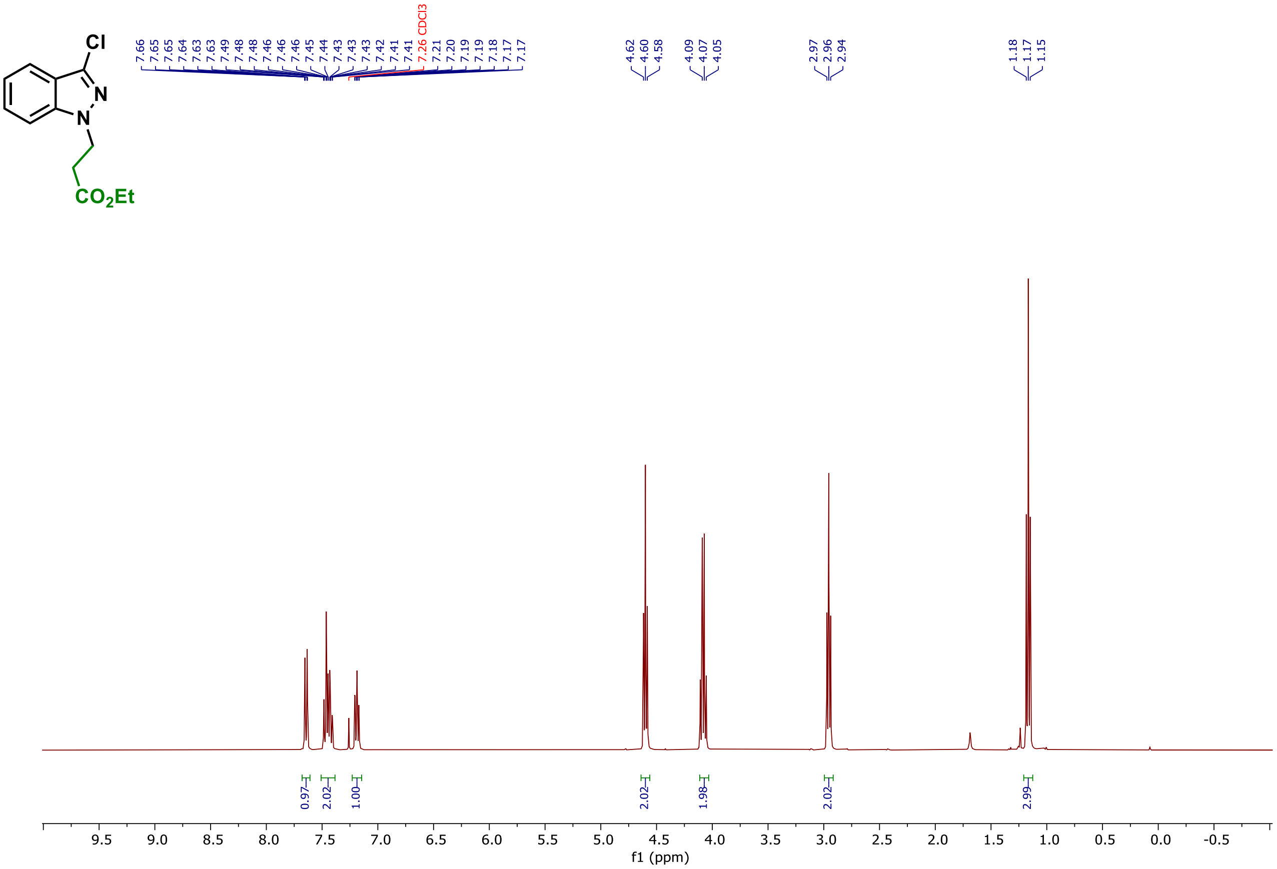

^{13}C NMR (101 MHz, $CDCl_3$) of **53**

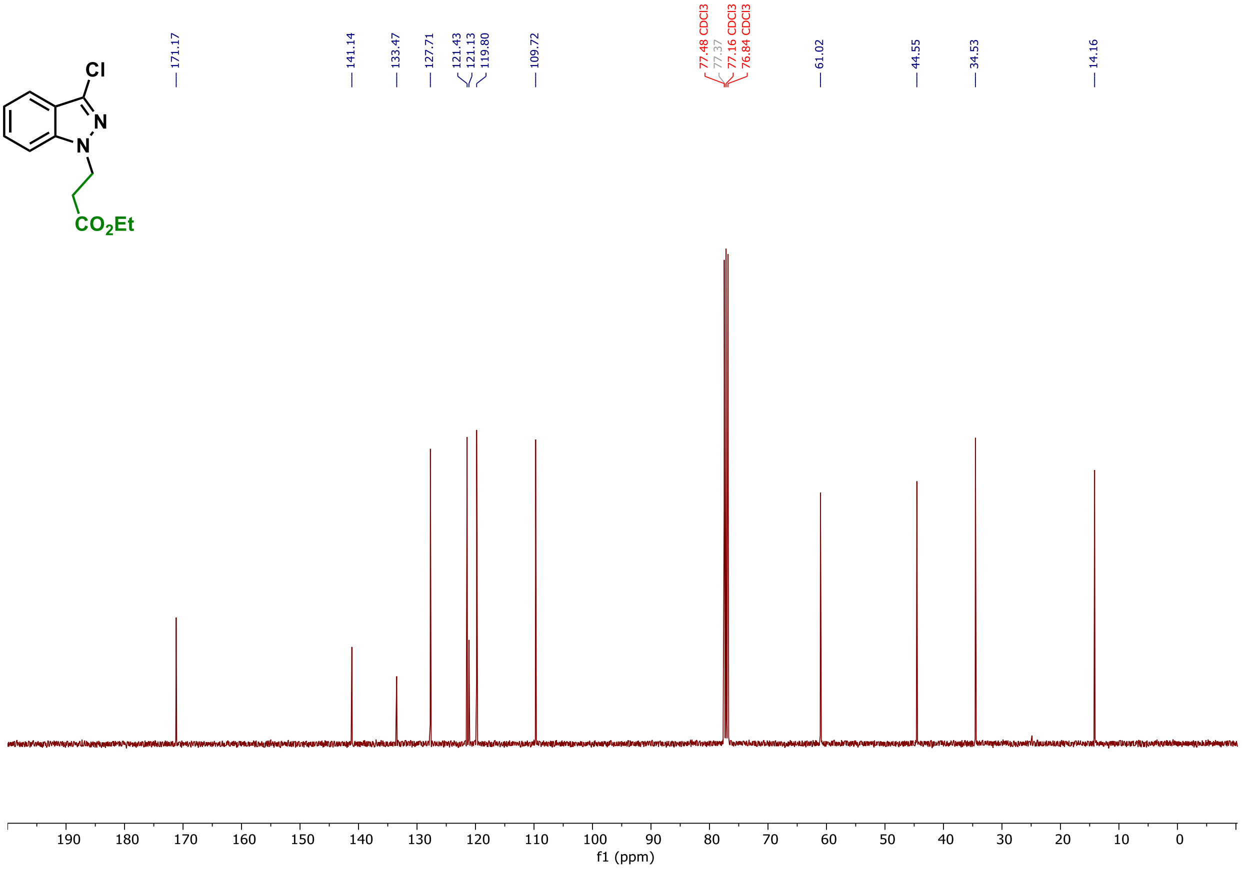

^{1}H NMR (400 MHz, $CDCl_3$) of **54** (*see procedure*)

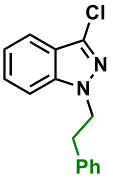

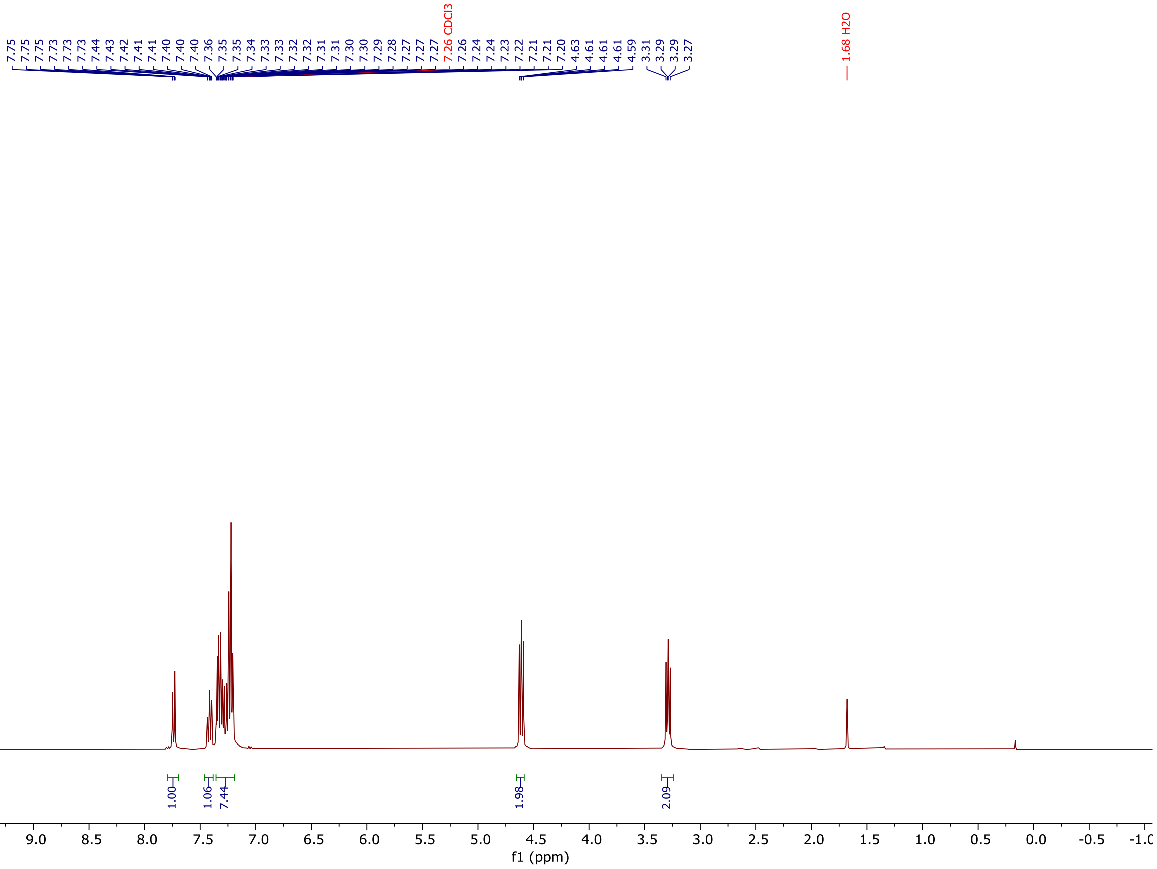

^{13}C NMR (101 MHz, $CDCl_3$) of **54**

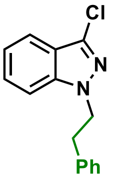

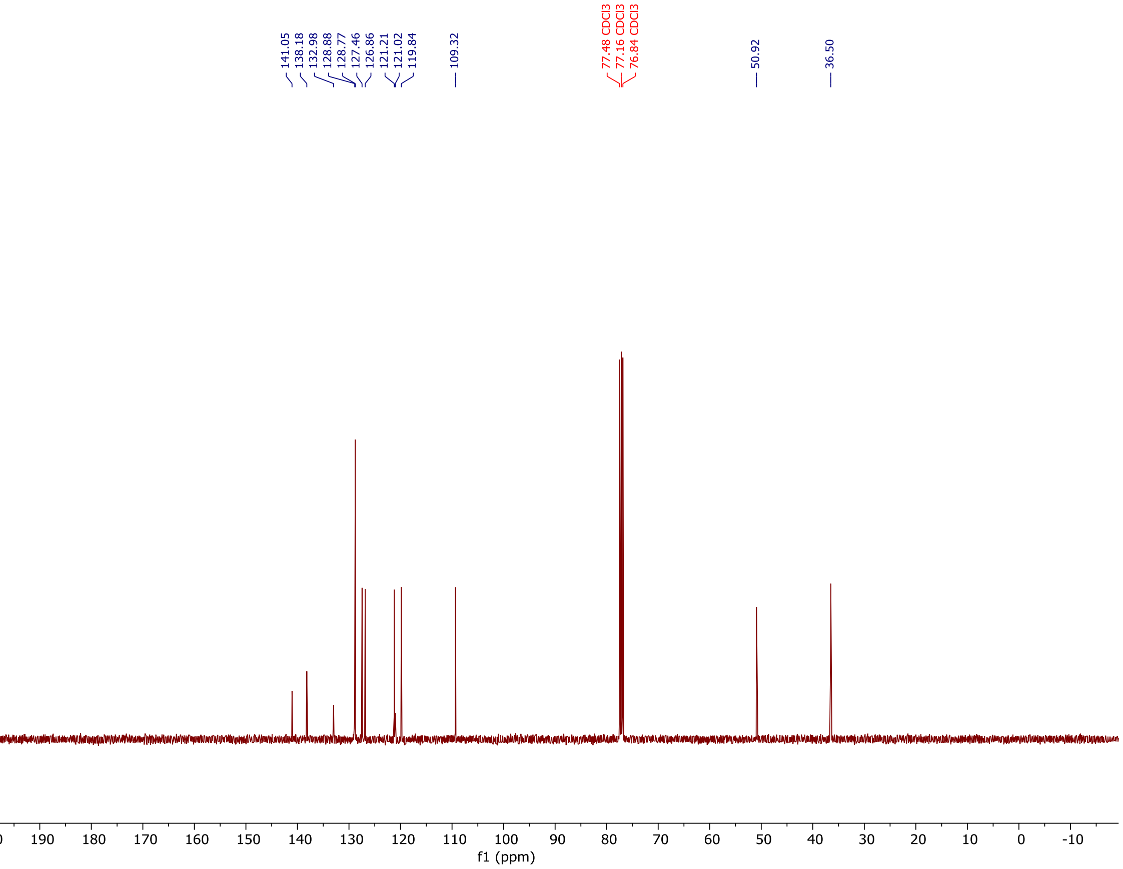

^{1}H NMR (400 MHz, $CDCl_3$) of **55** (*see procedure*)

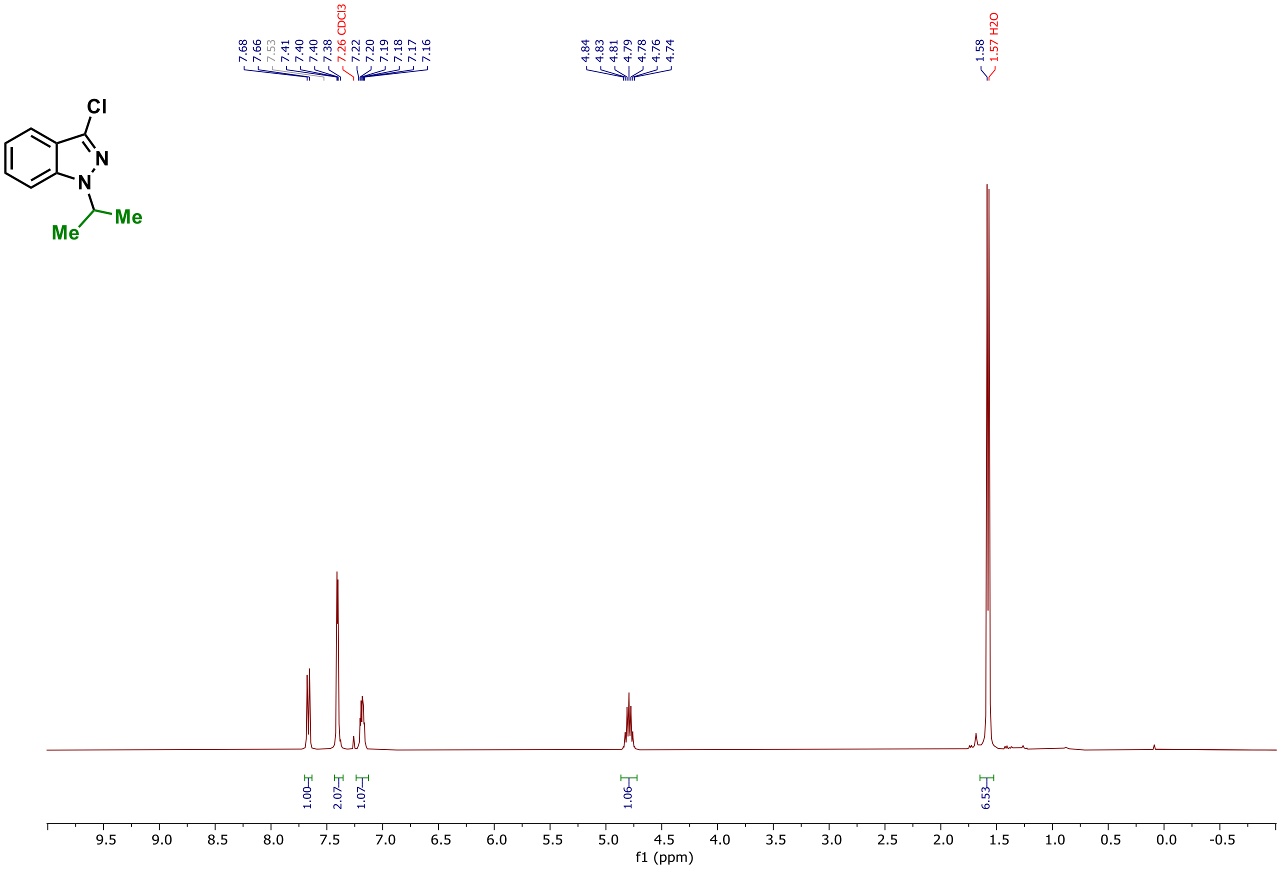

^{13}C NMR (101 MHz, $CDCl_3$) of **55**

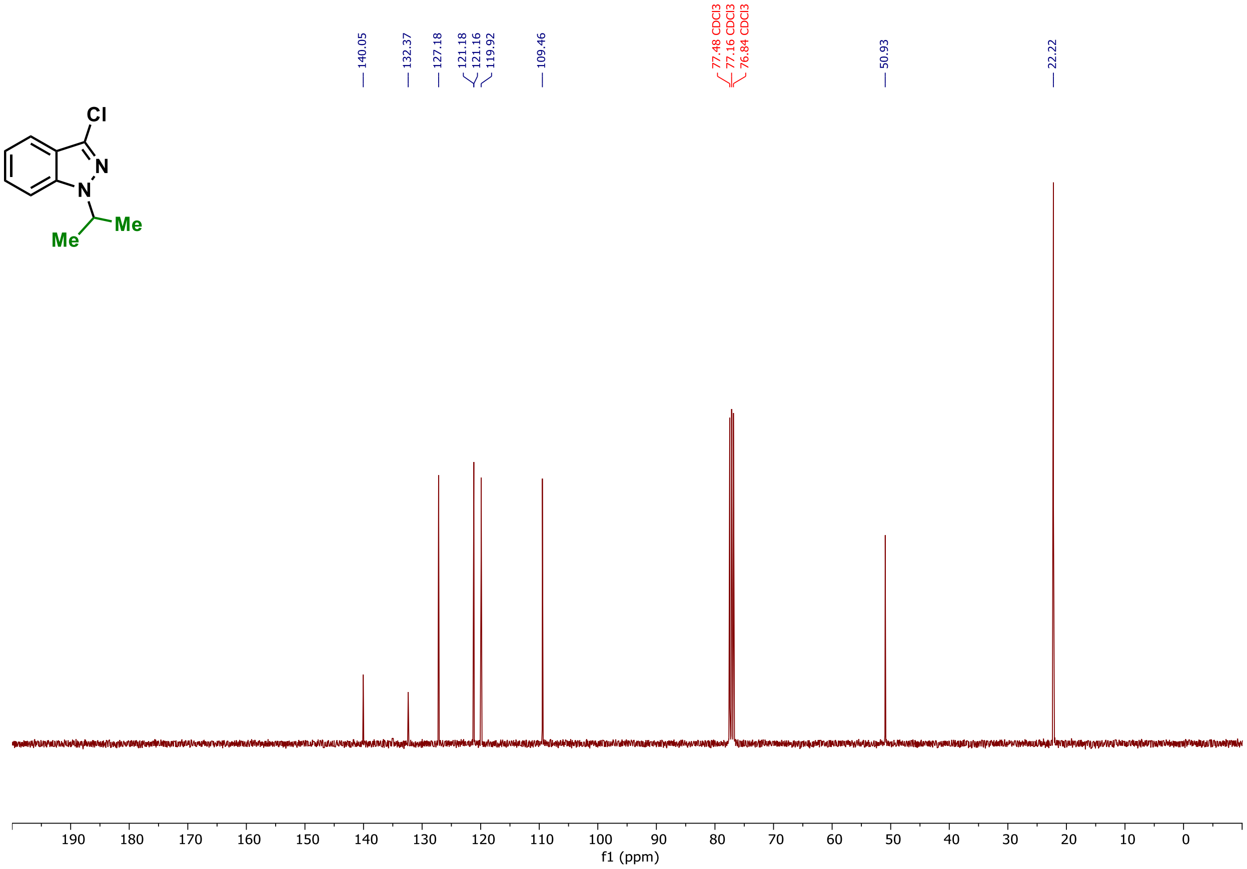

^{1}H NMR (400 MHz, $CDCl_3$) of **56** (*see procedure*)

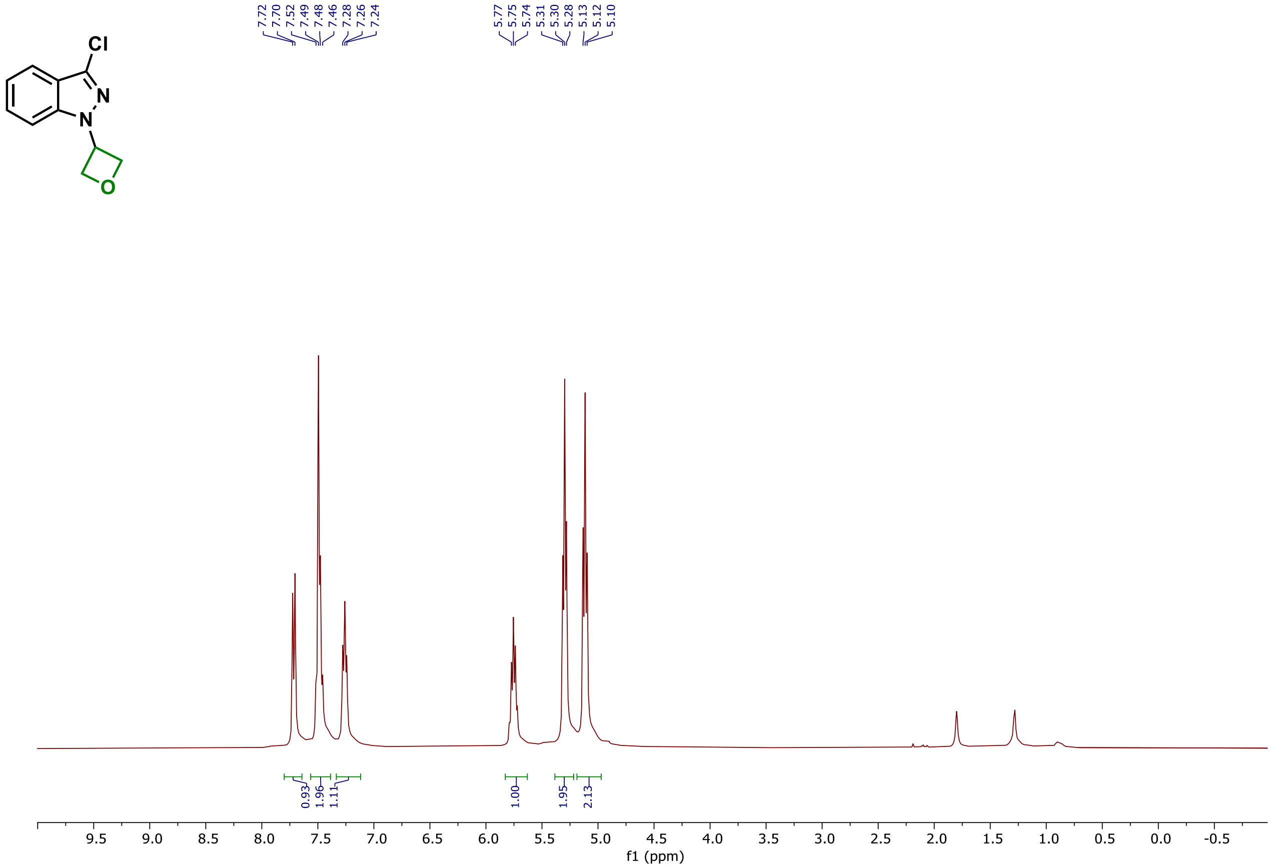

^{13}C NMR (101 MHz, $CDCl_3$) of **56**

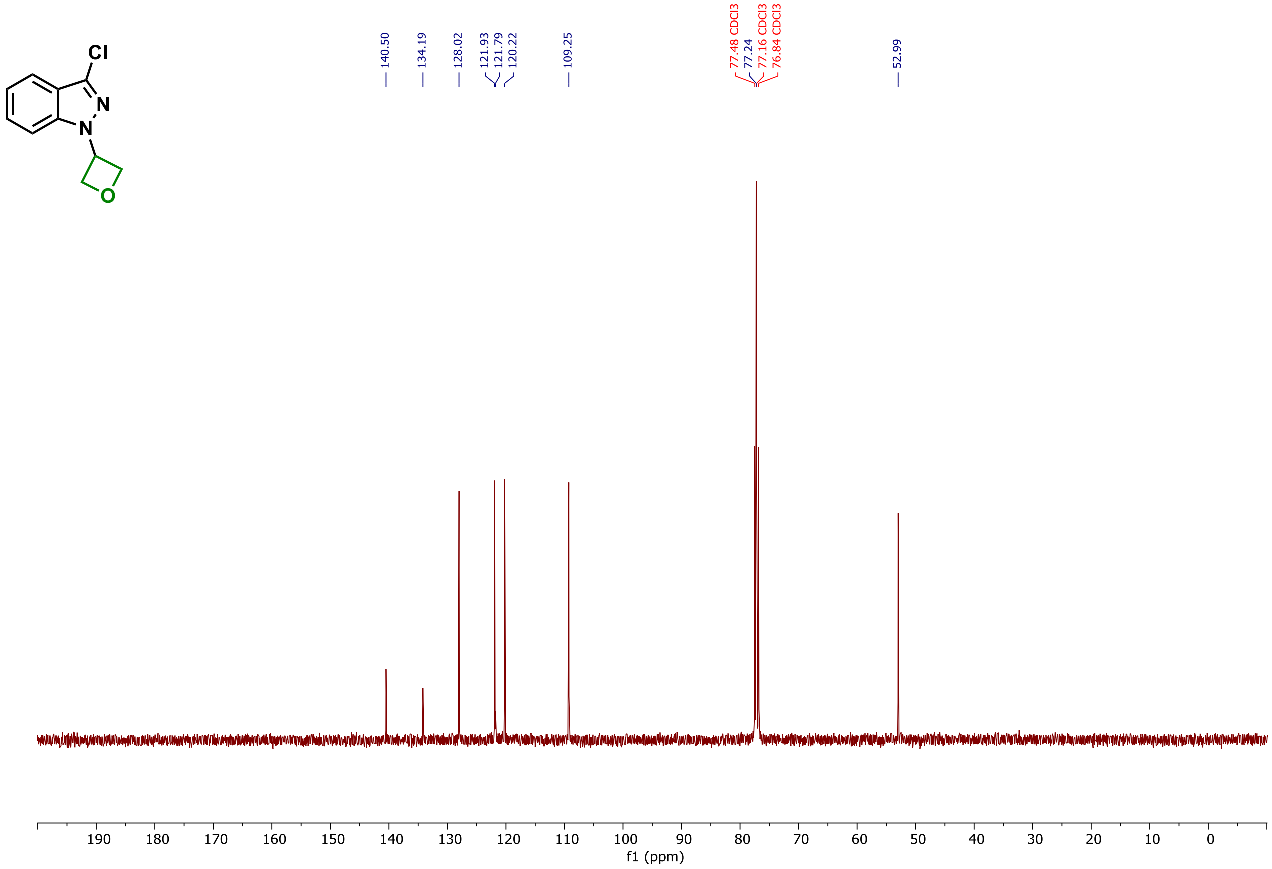

^{1}H NMR (400 MHz, $CDCl_3$) of **S57** (*see procedure*)

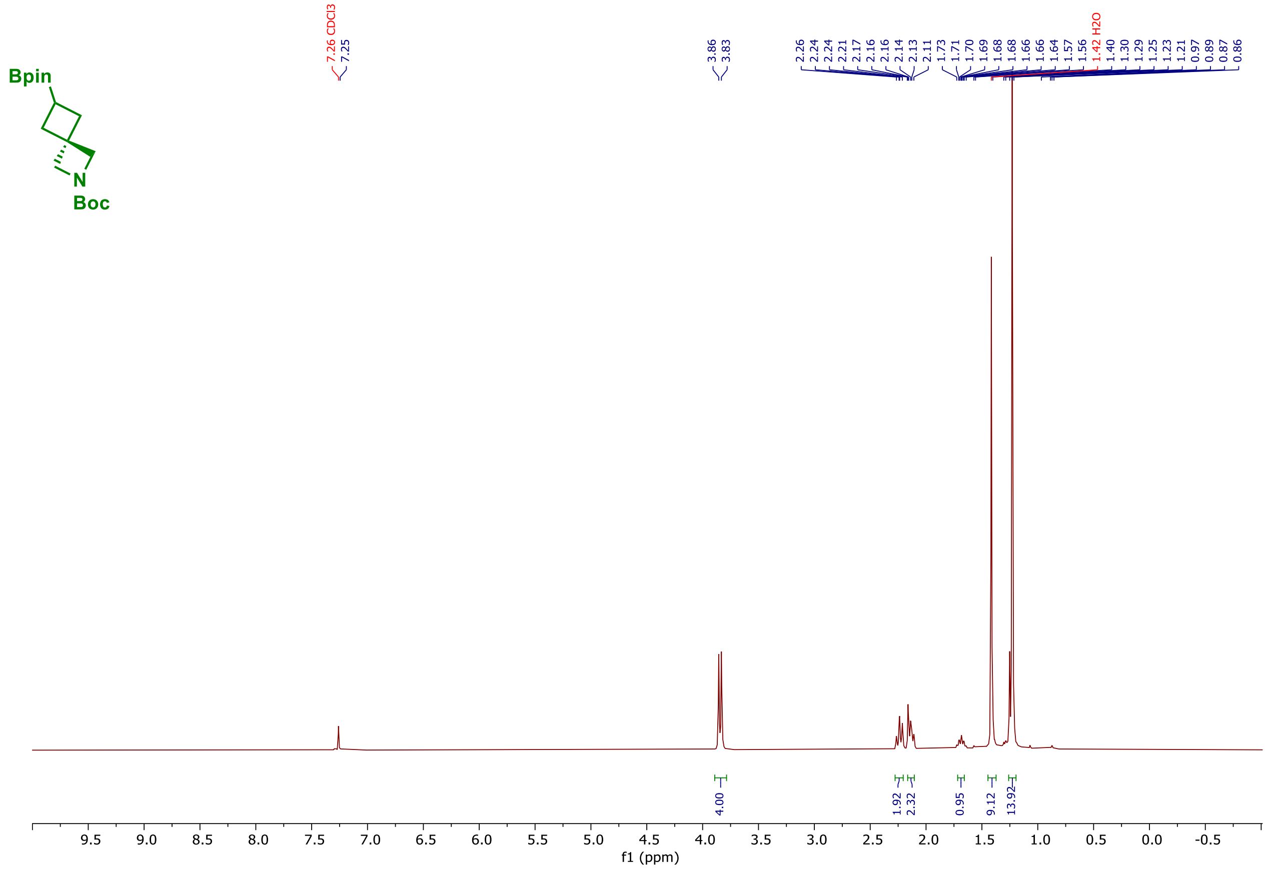

^{13}C NMR (101 MHz, $CDCl_3$) of **S57**

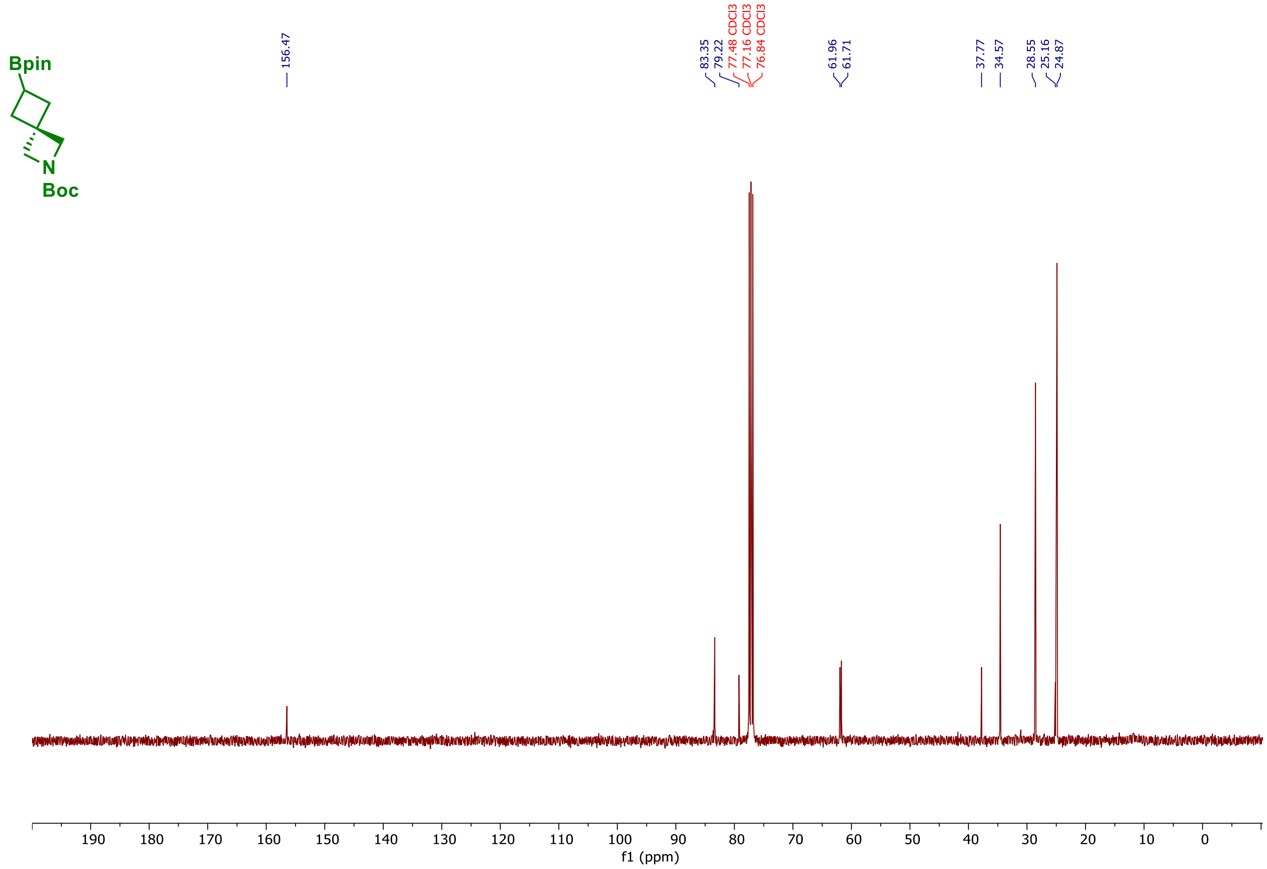

^{11}B NMR (128 MHz, $CDCl_3$) of **S57**

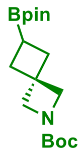

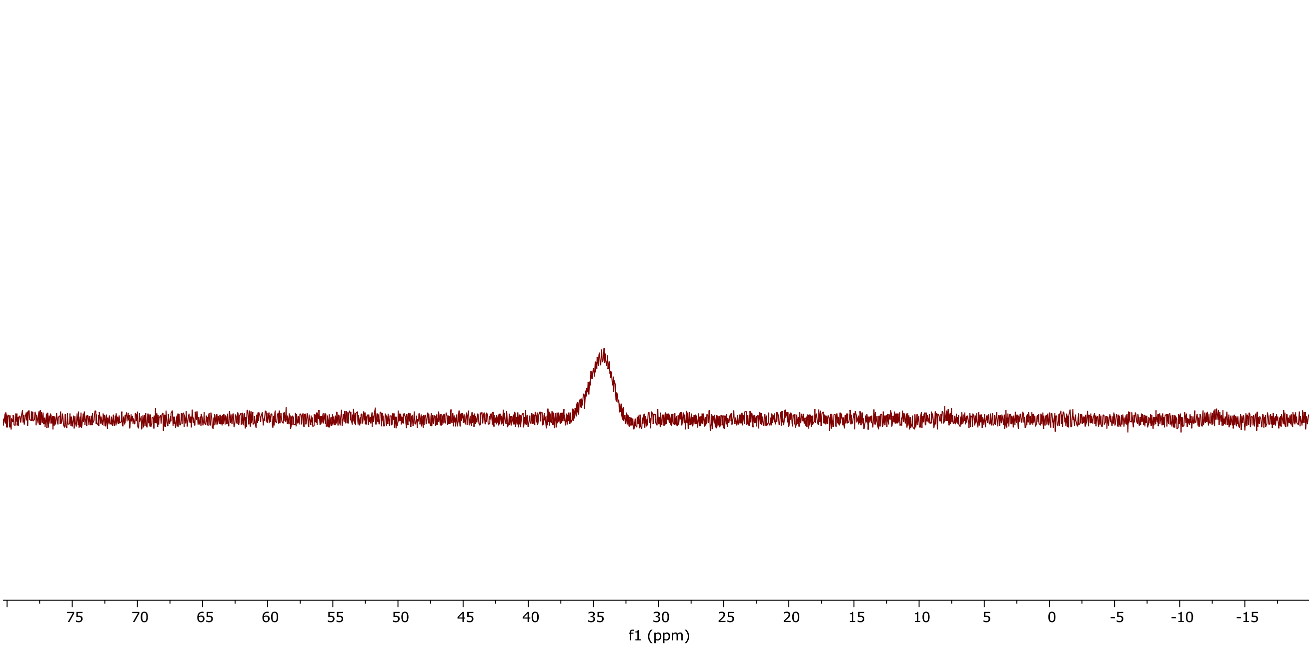

^{1}H NMR (400 MHz, $CDCl_3$) of **57** (*see procedure*)

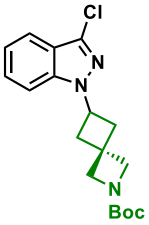

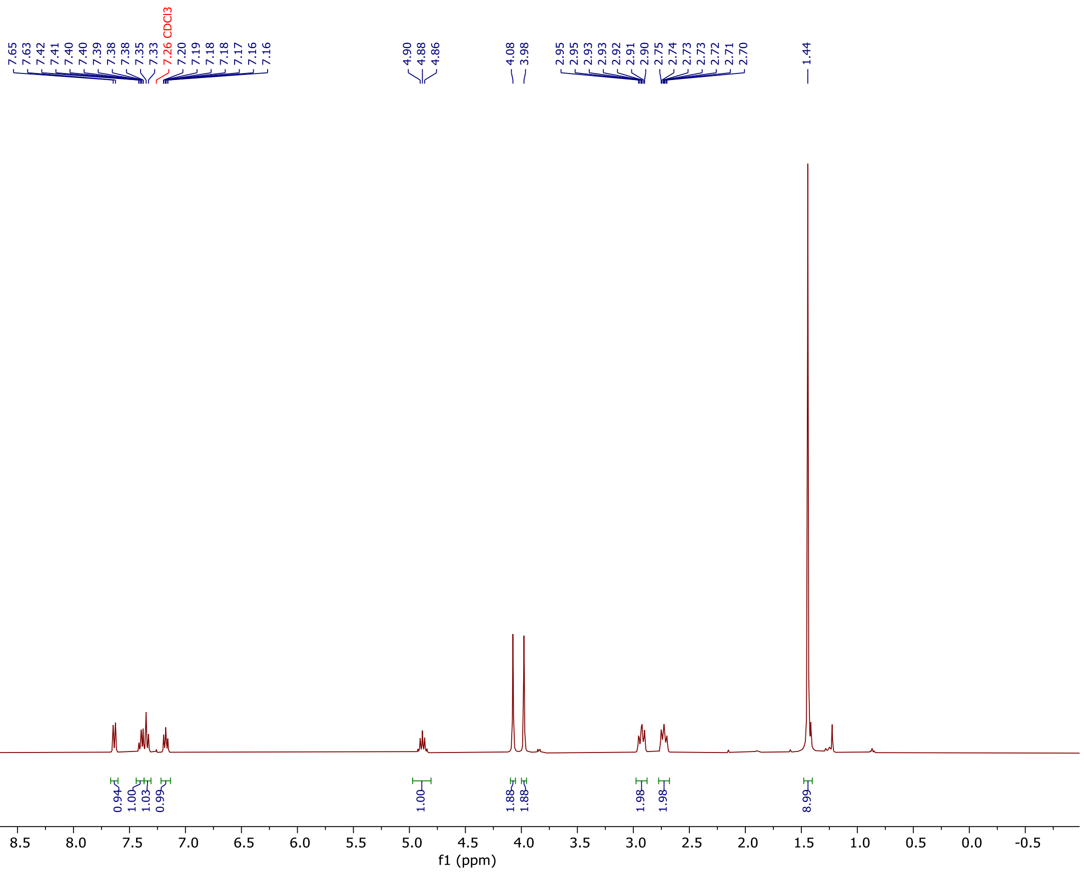

^{13}C NMR (101 MHz, $CDCl_3$) of **57**

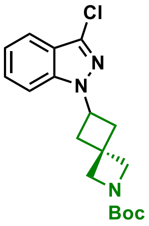

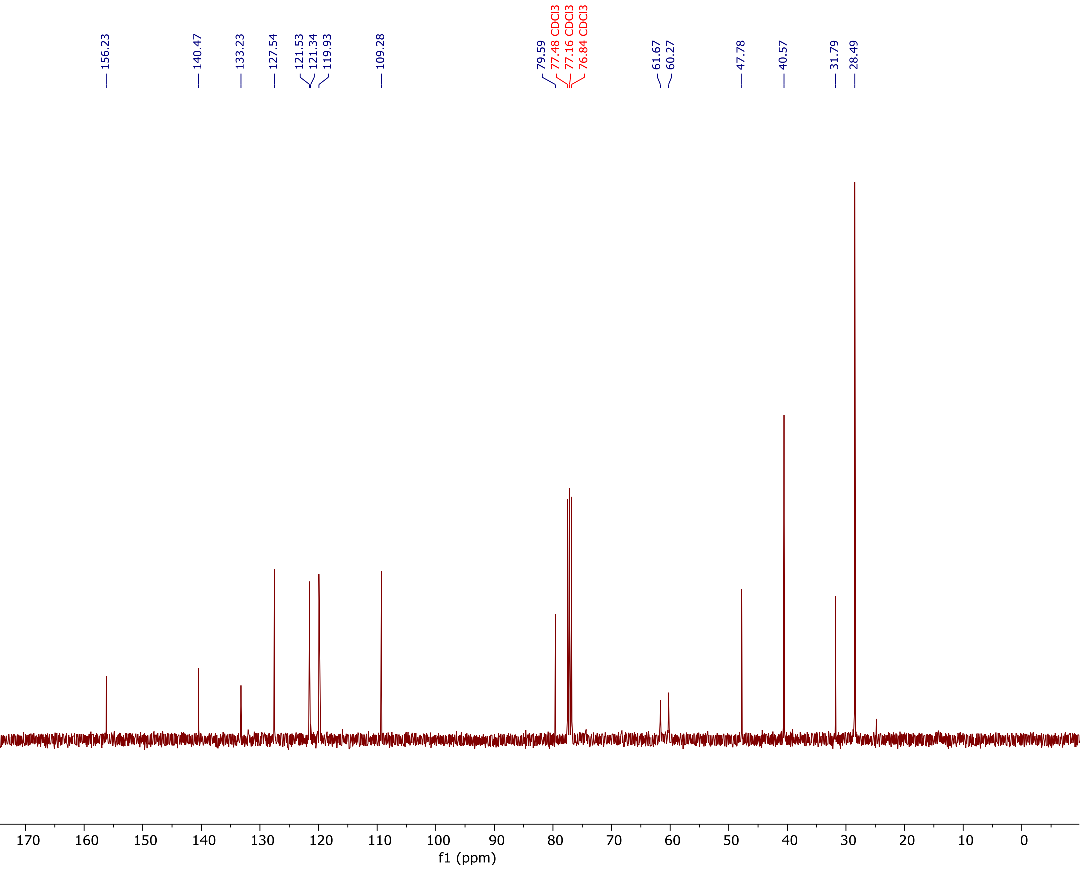

^{1}H NMR (400 MHz, $CDCl_3$) of **58** (*see procedure*)

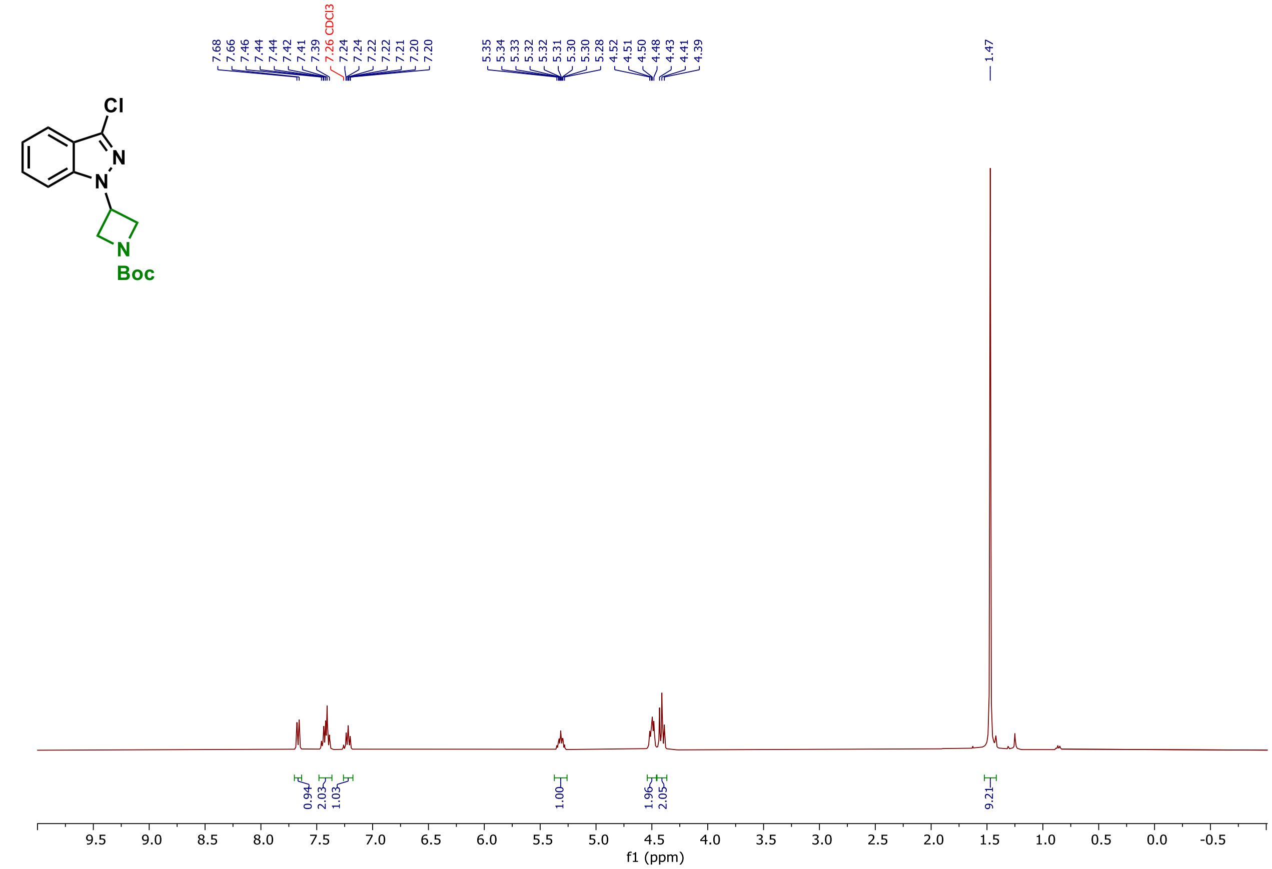

^{13}C NMR (101 MHz, $CDCl_3$) of **58**

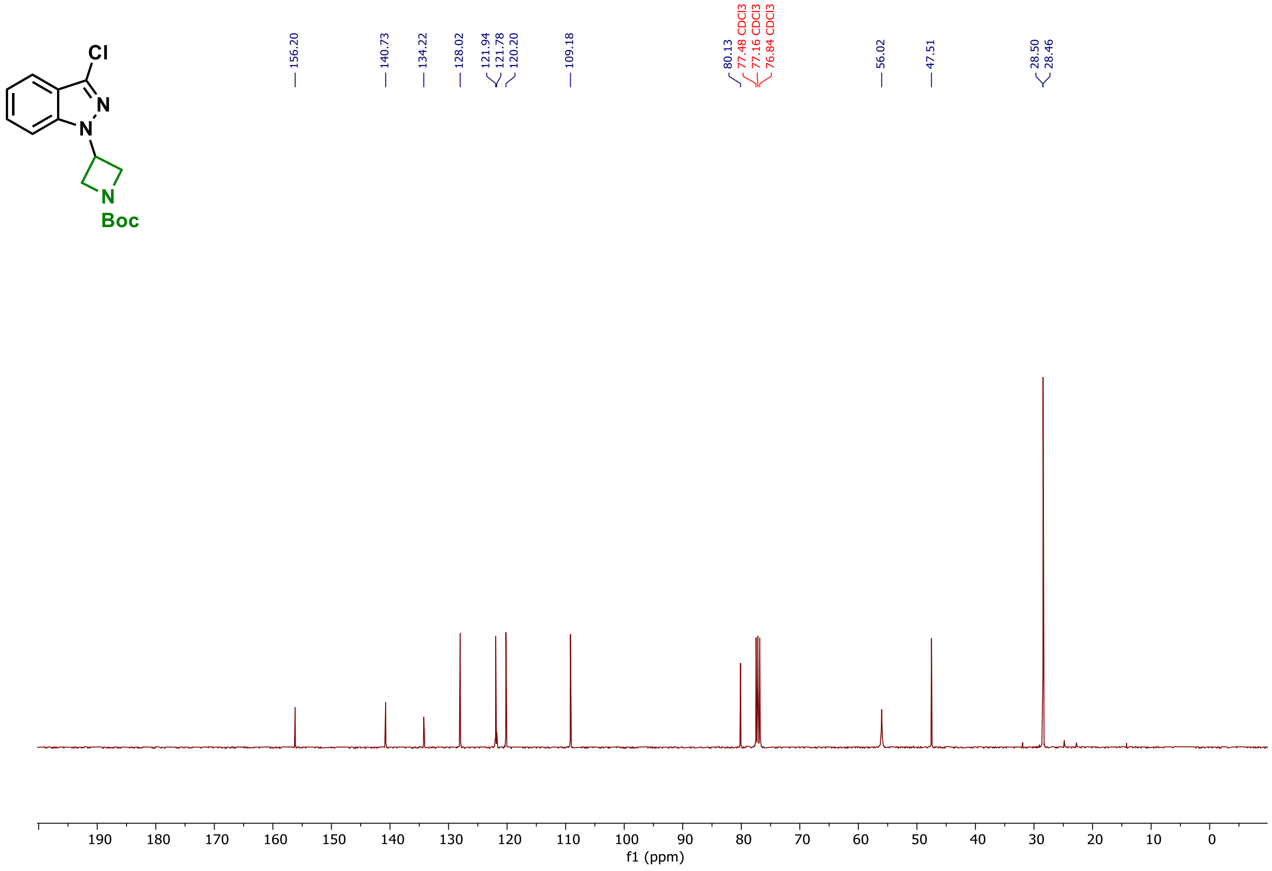

^{1}H NMR (400 MHz, $CDCl_3$) of **59** (see procedure)

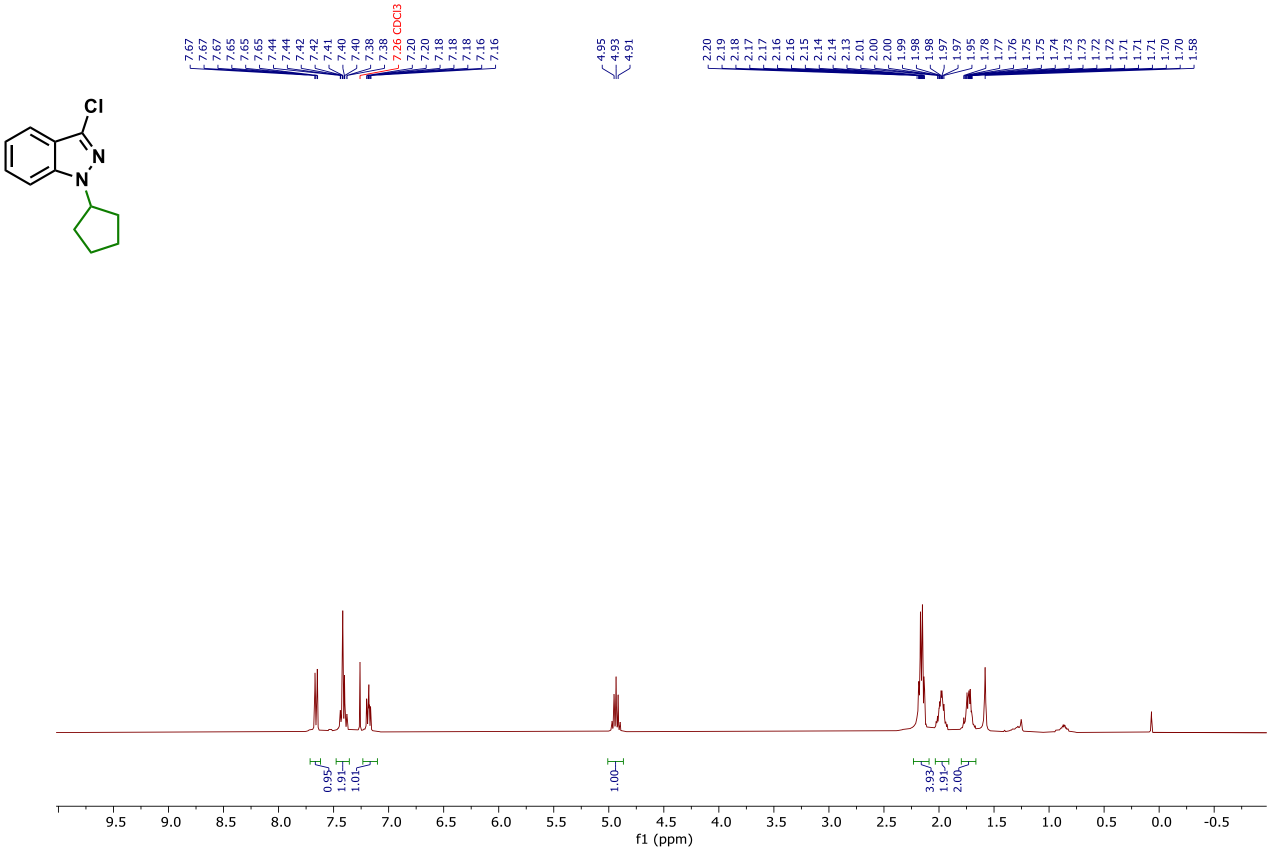

^{13}C NMR (101 MHz, $CDCl_3$) of **59**

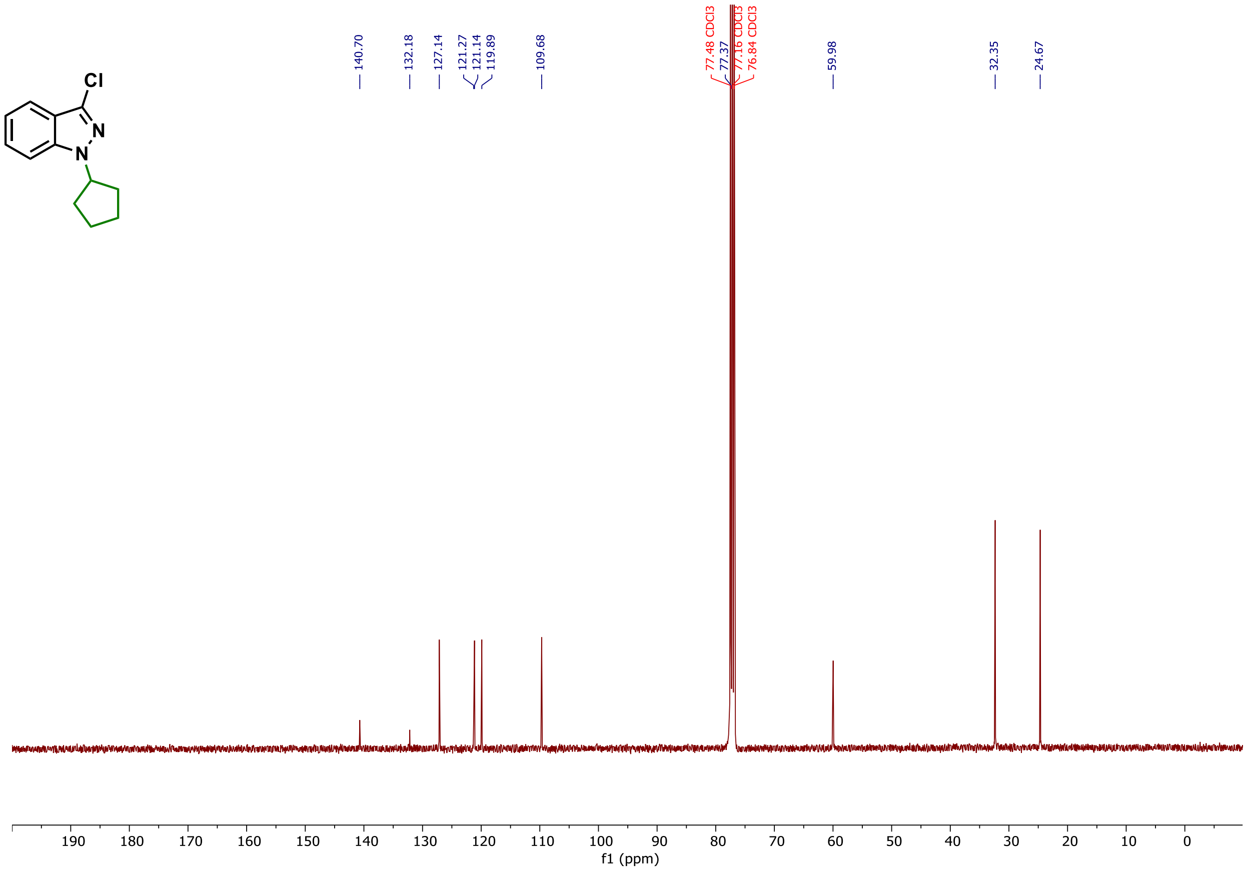

^{1}H NMR (400 MHz, $CDCl_3$) of **60** (*see procedure*)

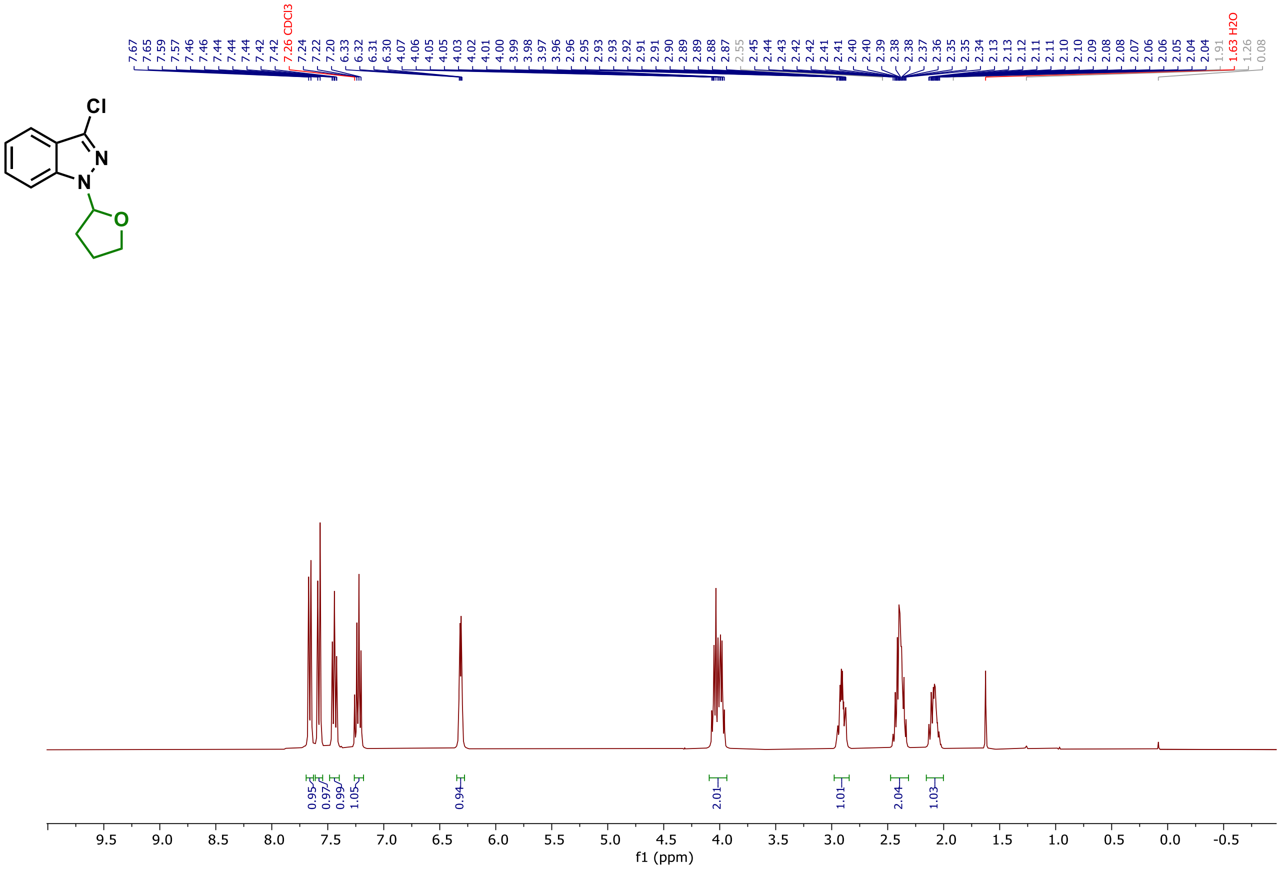

^{13}C NMR (101 MHz, $CDCl_3$) of **60**

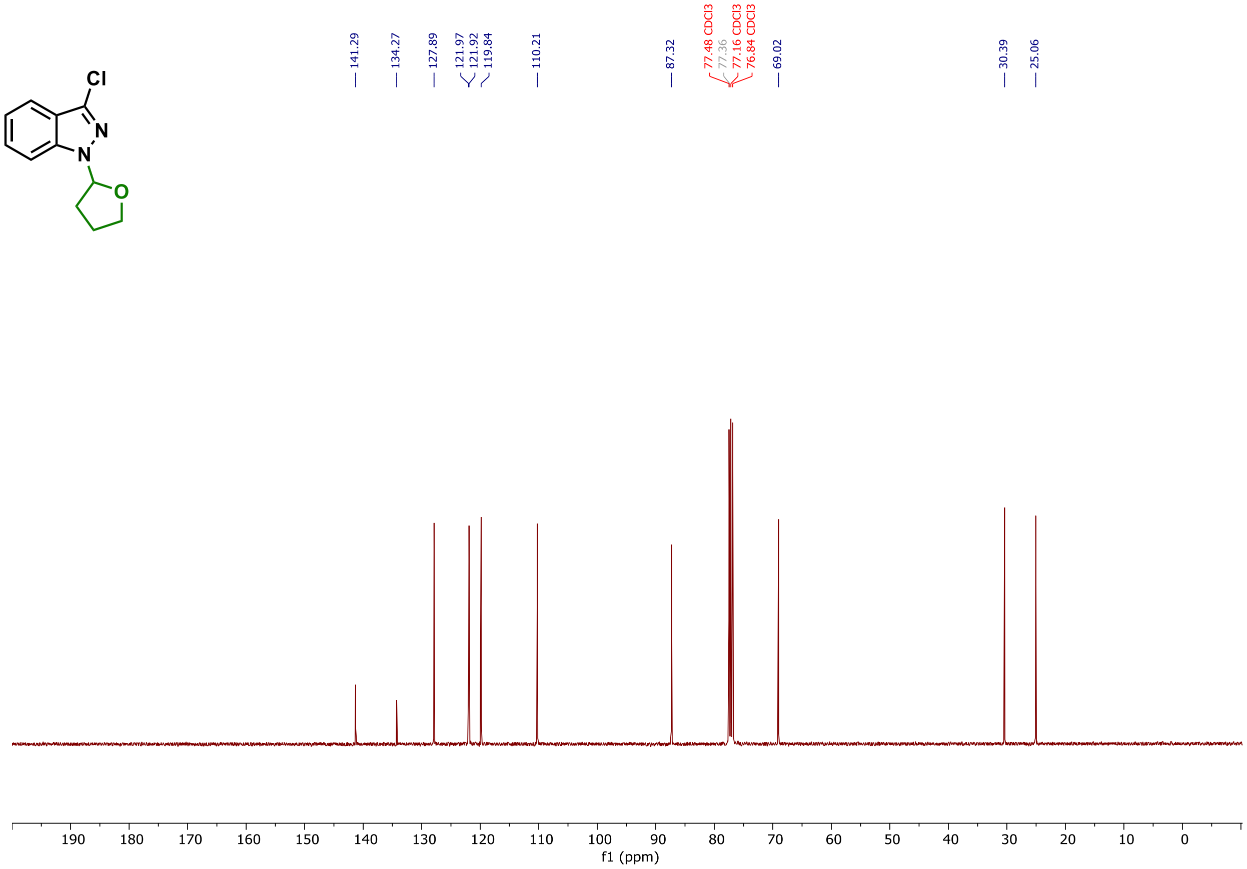

^{1}H NMR (400 MHz, $CDCl_3$) of **61** (*see procedure*)

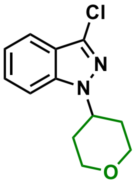

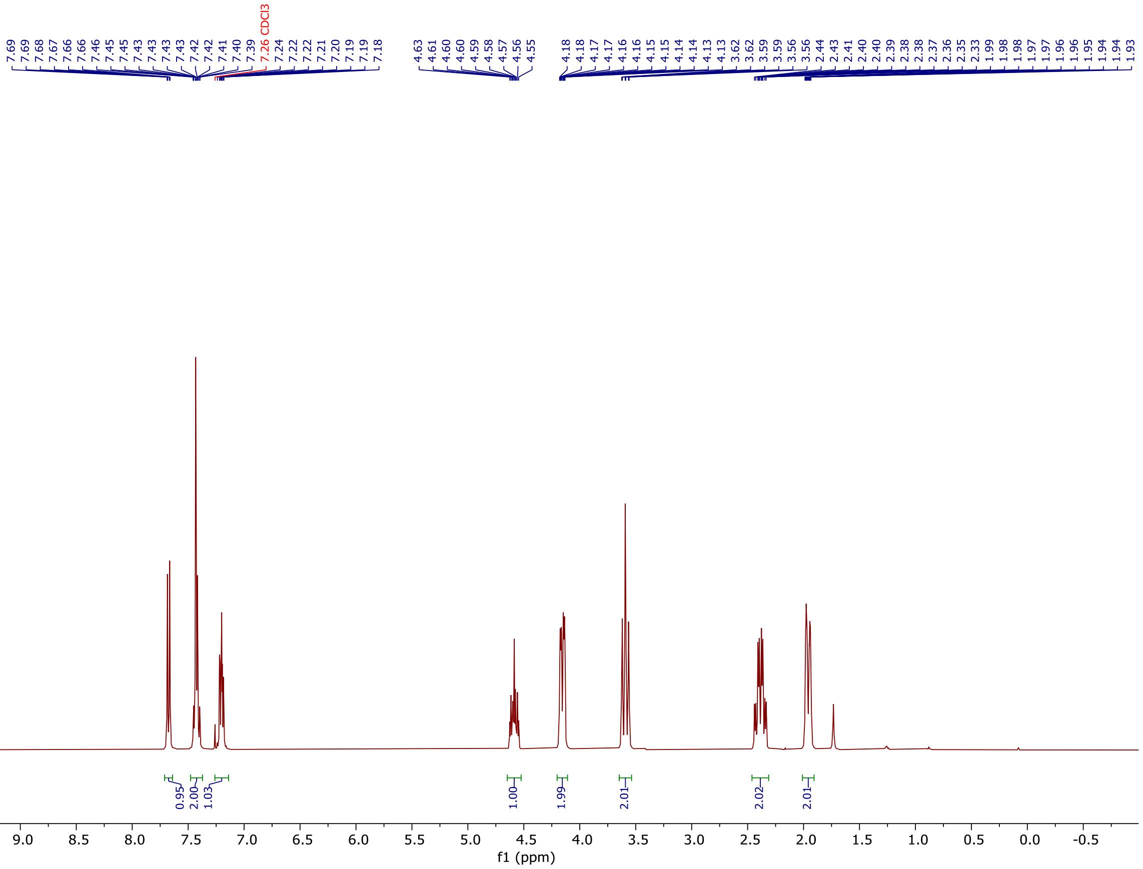

^{13}C NMR (101 MHz, $CDCl_3$) of **61**

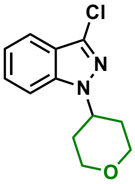

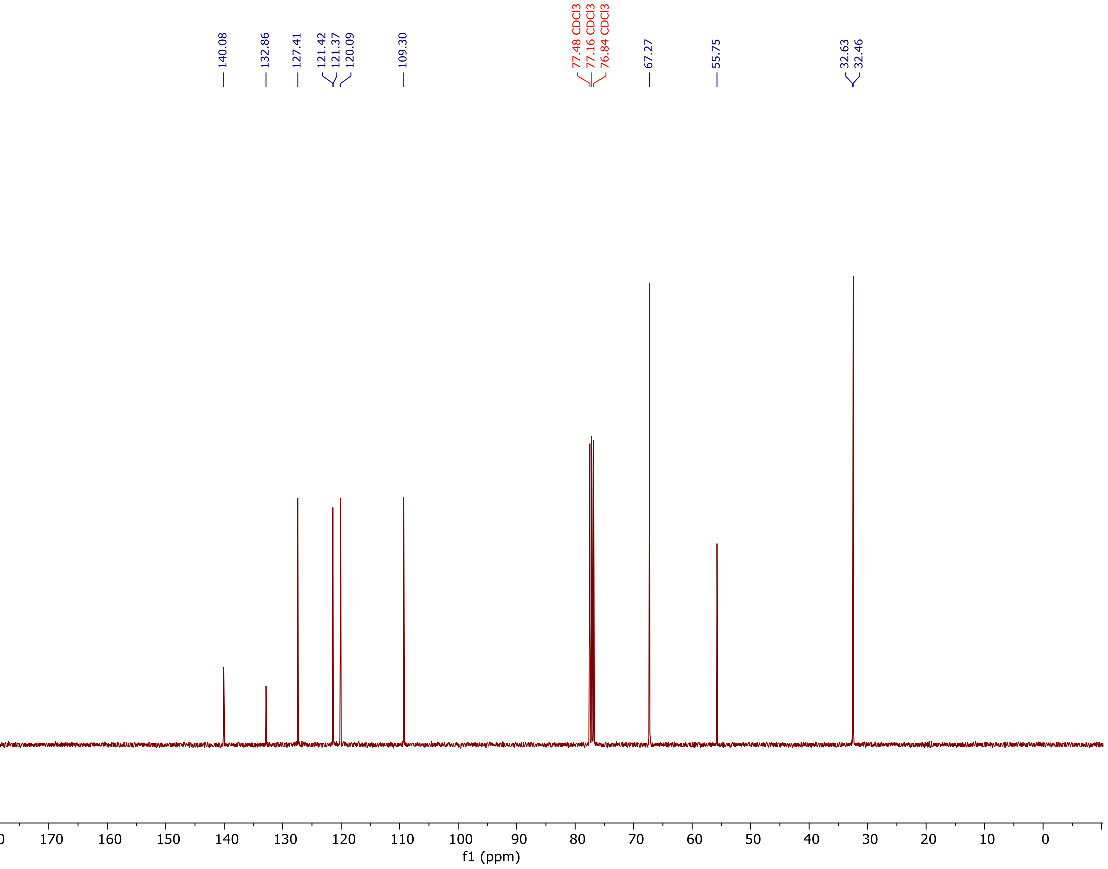

^{1}H NMR (400 MHz, $CDCl_3$) of **62** (*see procedure*)

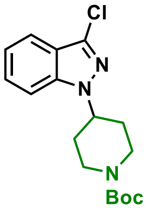

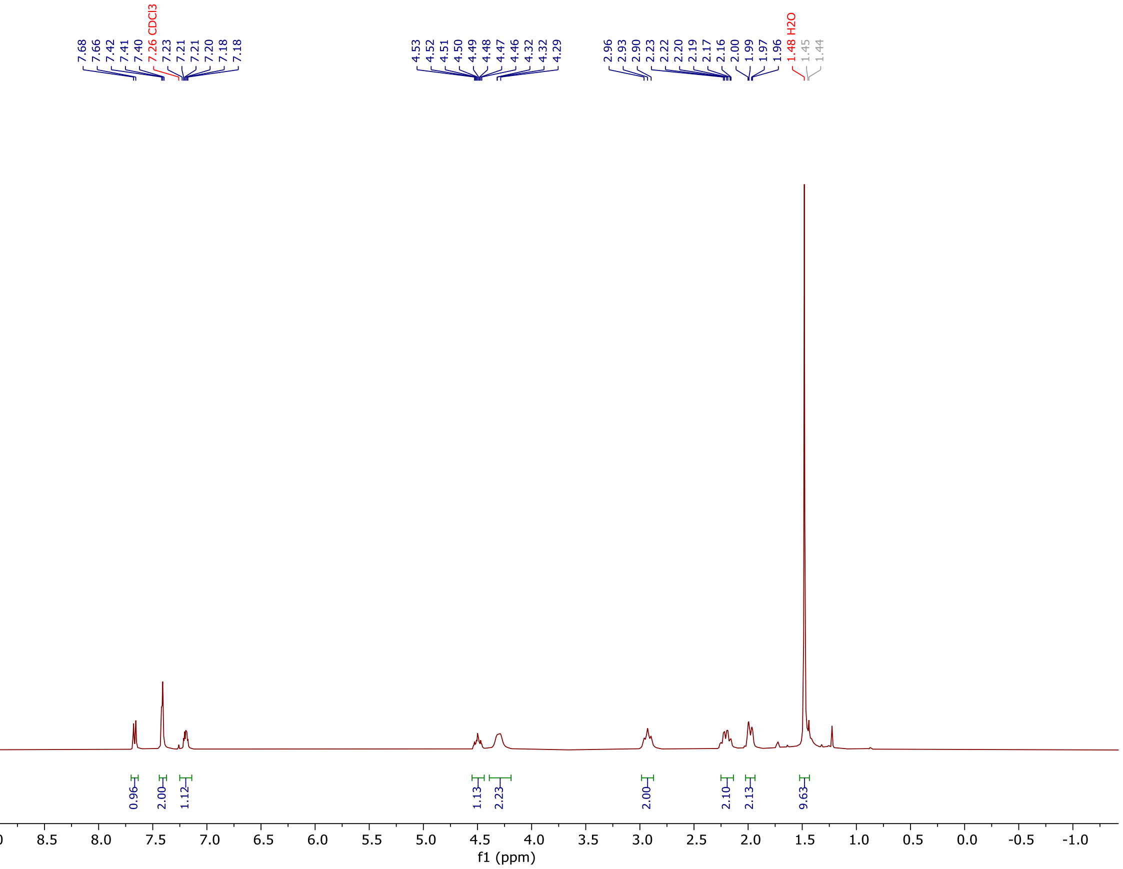

^{13}C NMR (101 MHz, $CDCl_3$) of **62**

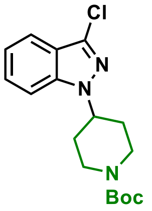

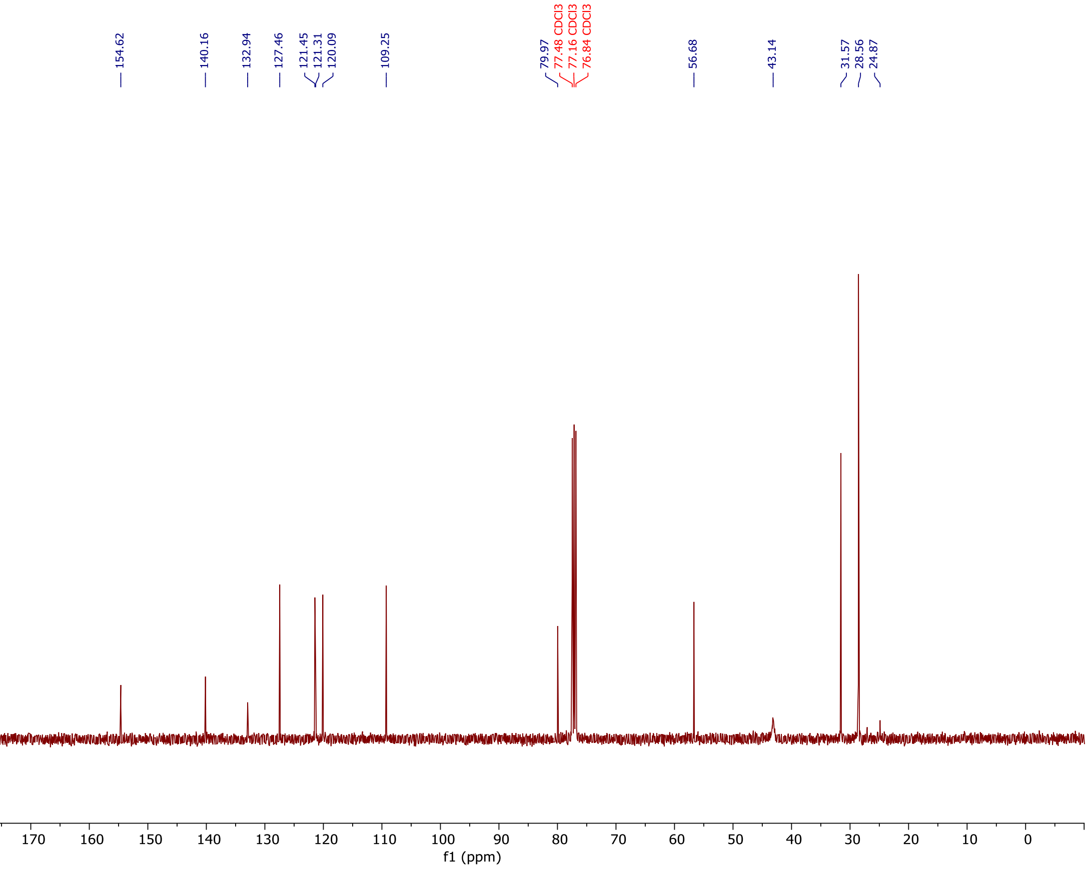

^{1}H NMR (400 MHz, $CDCl_3$) of **63** (*see procedure*)

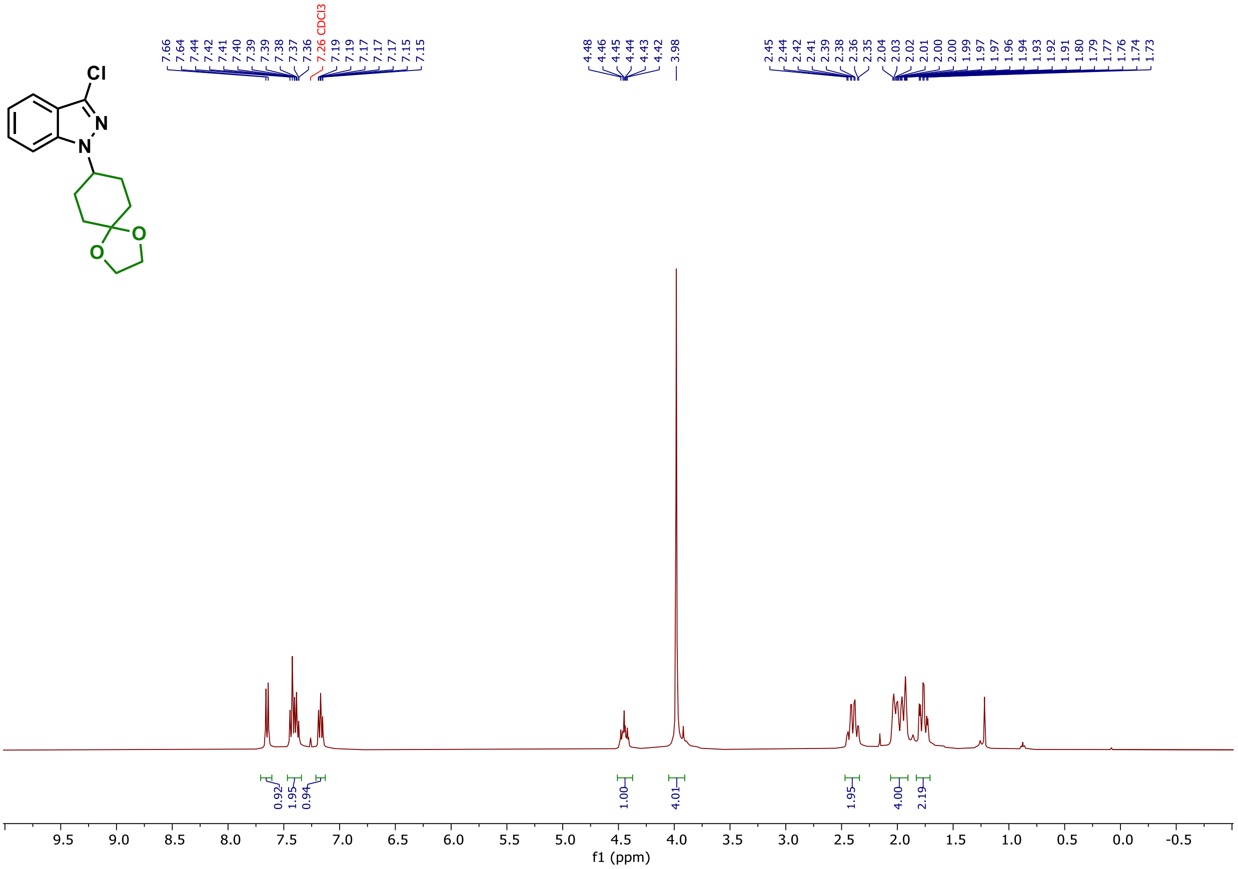

^{13}C NMR (101 MHz, $CDCl_3$) of **63**

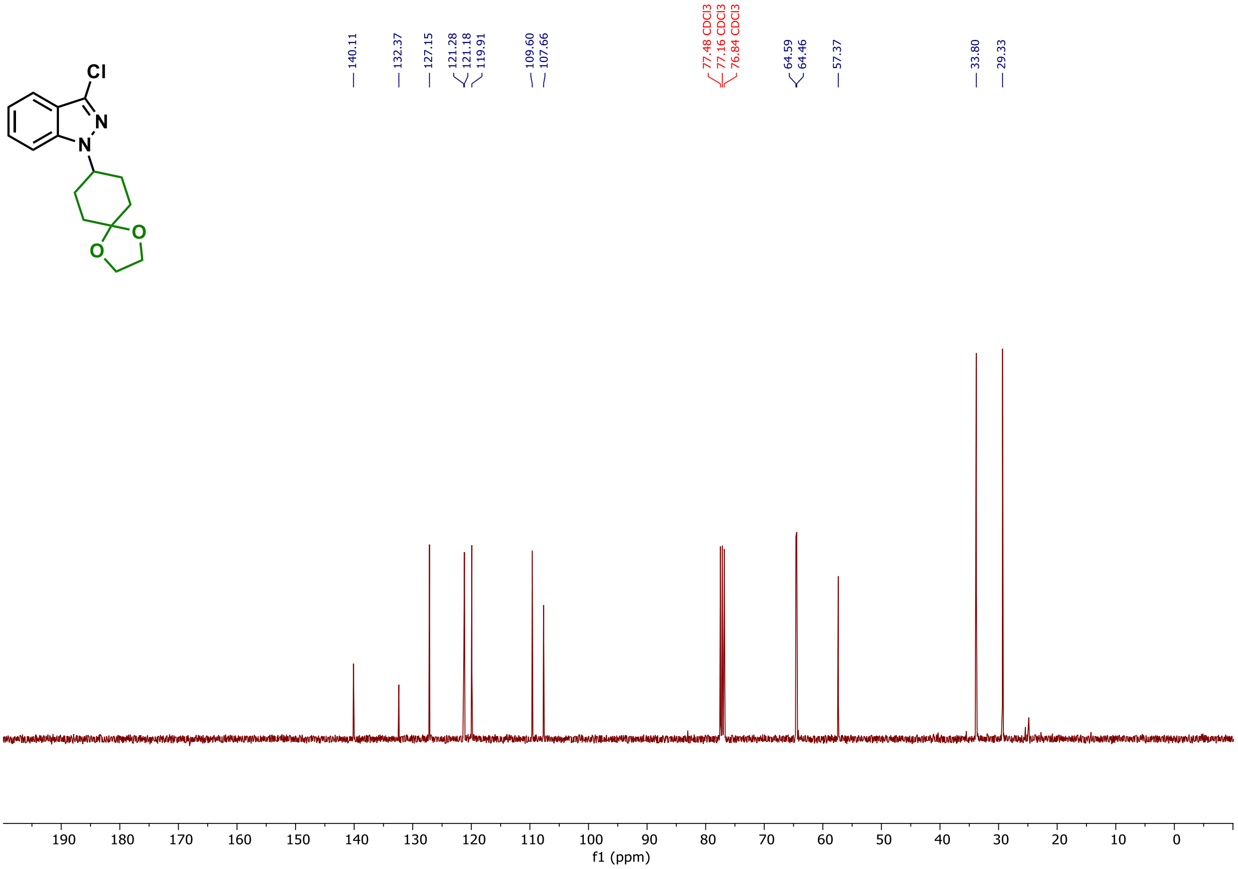

^{1}H NMR (400 MHz, $CDCl_3$) of **64** (*see procedure*)

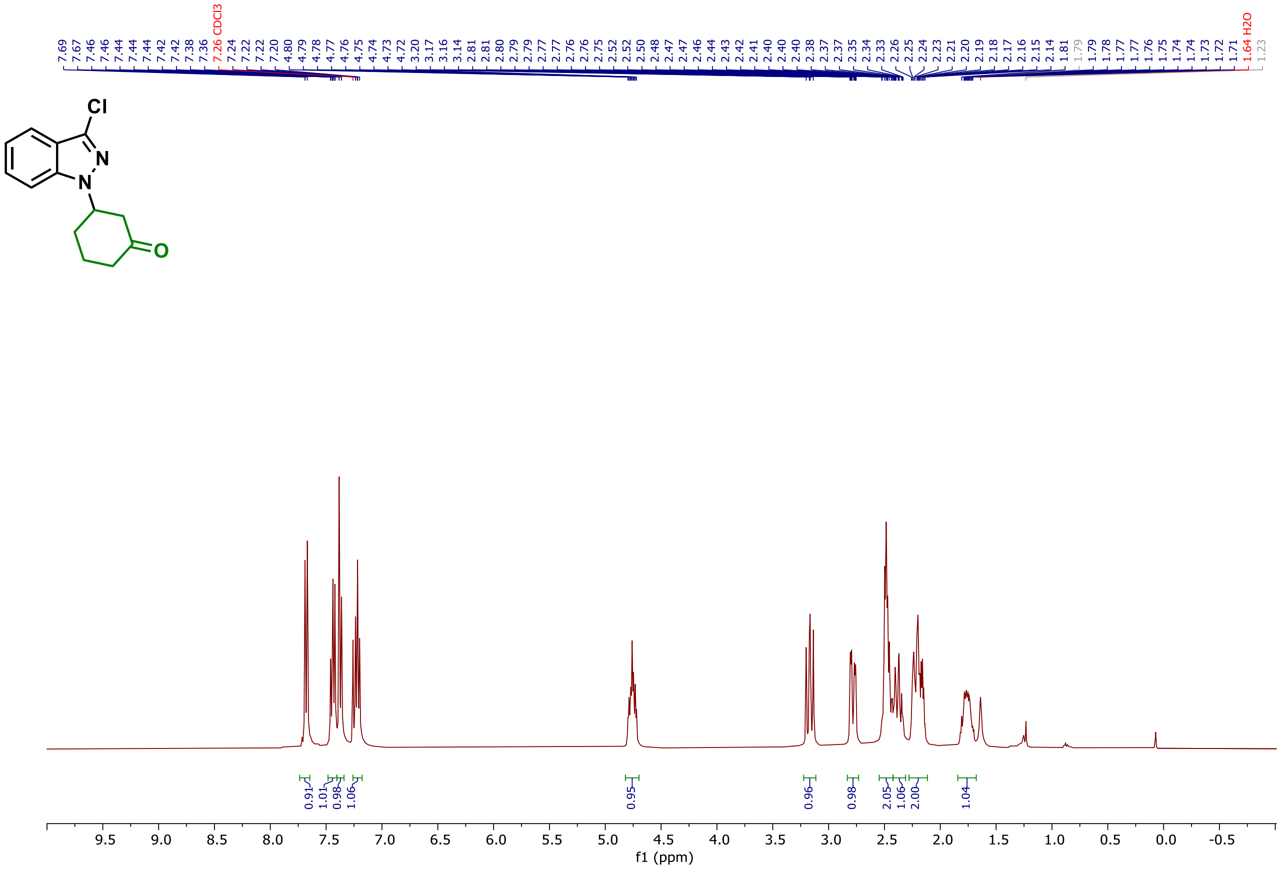

^{13}C NMR (101 MHz, $CDCl_3$) of **64**

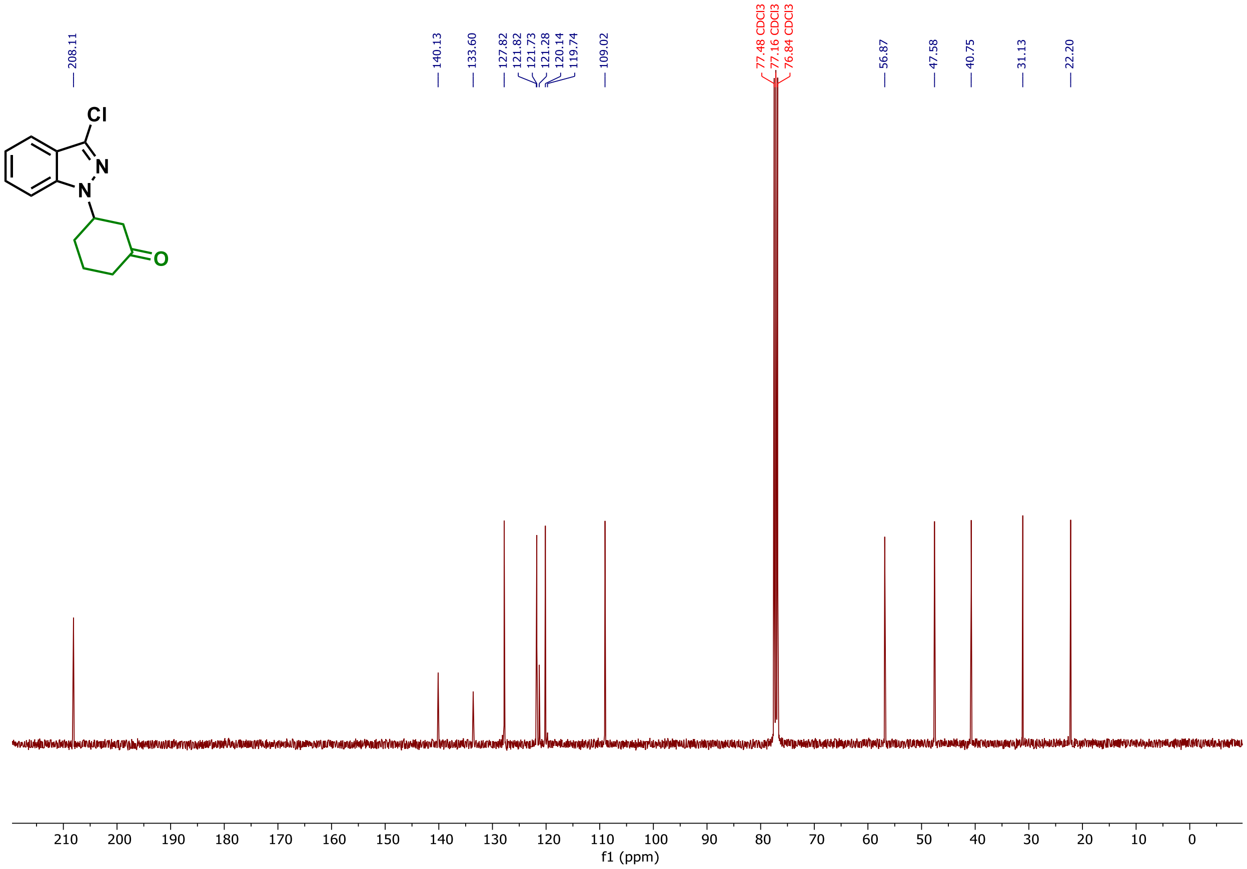

^{1}H NMR (400 MHz, $CDCl_3$) of **65** (see procedure)

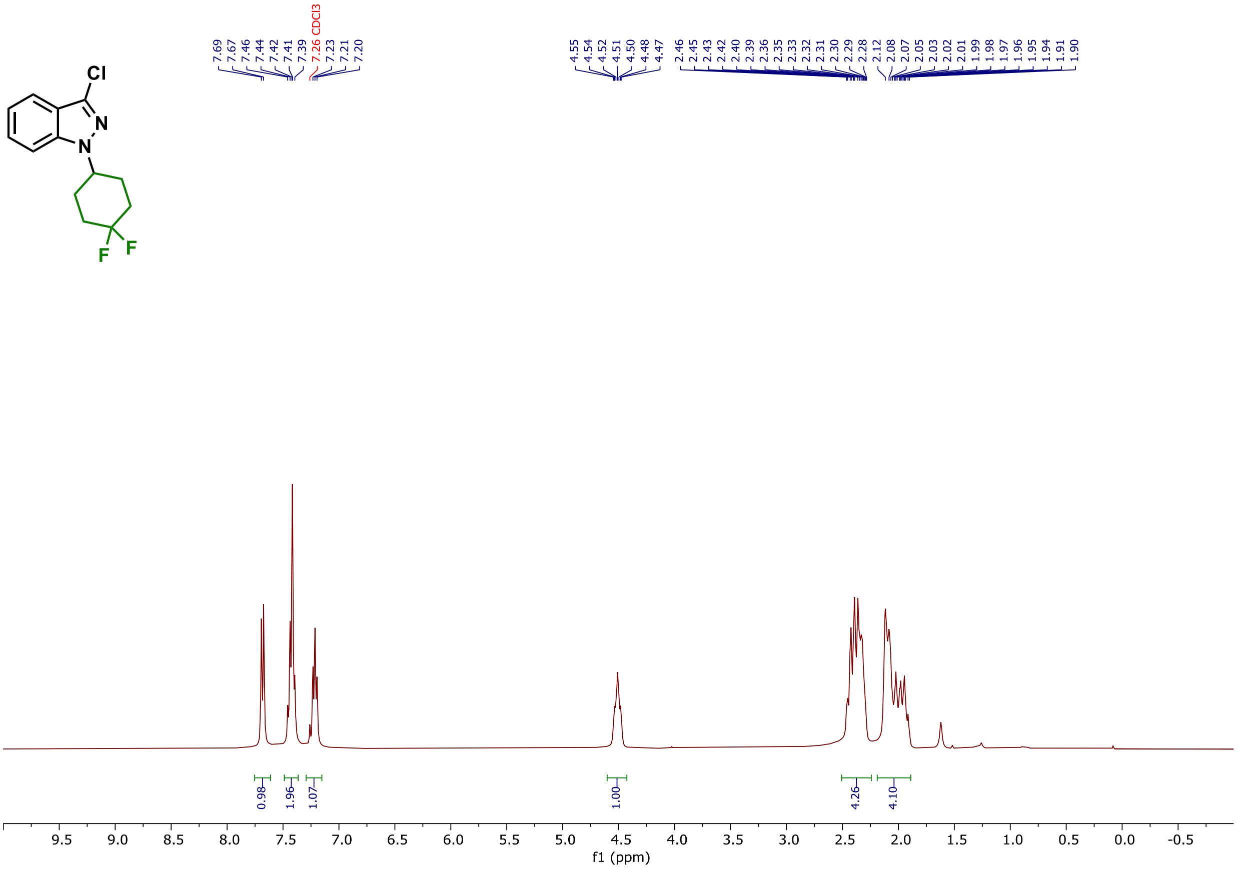

^{13}C NMR (101 MHz, $CDCl_3$) of **65**

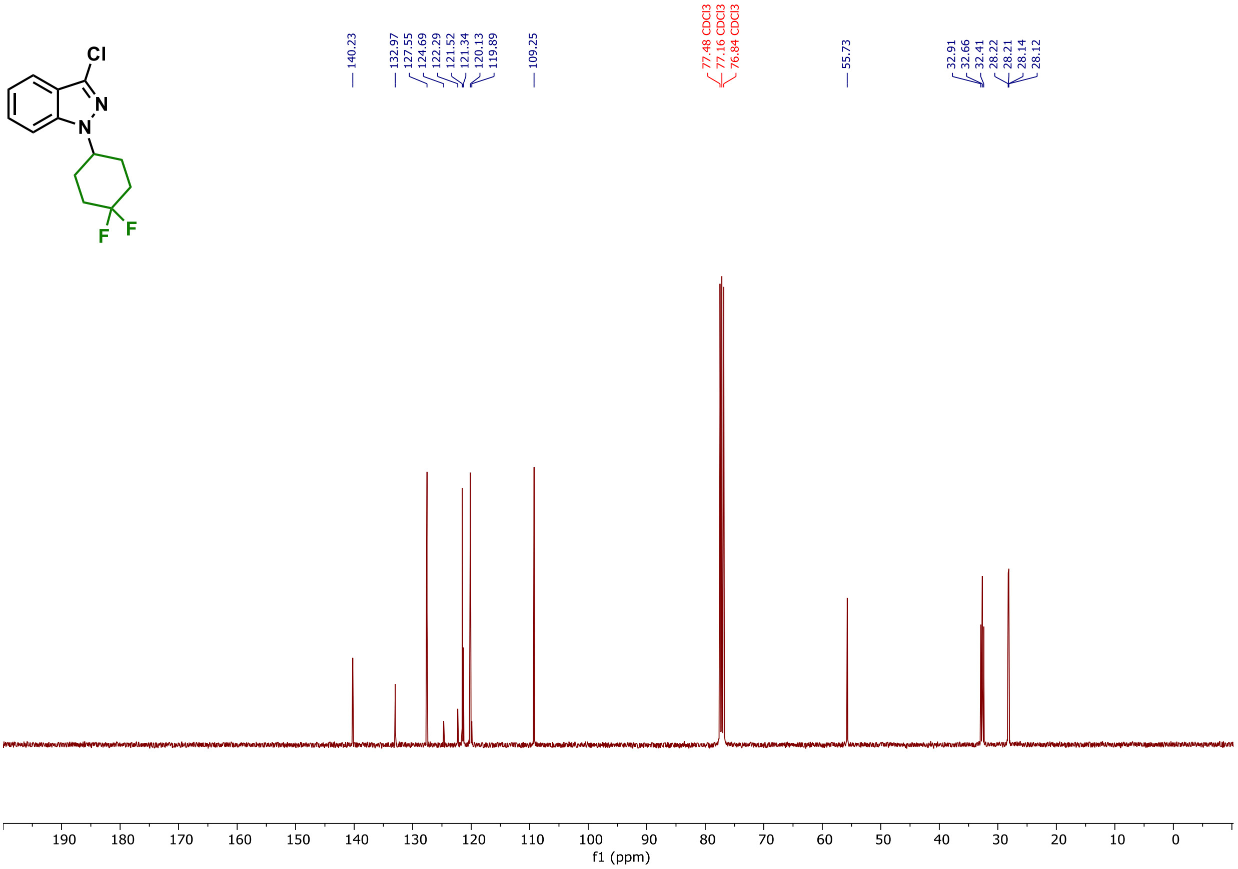

^{19}F NMR (376 MHz, $CDCl_3$) of **65**

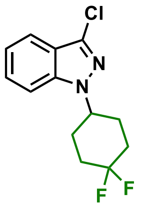

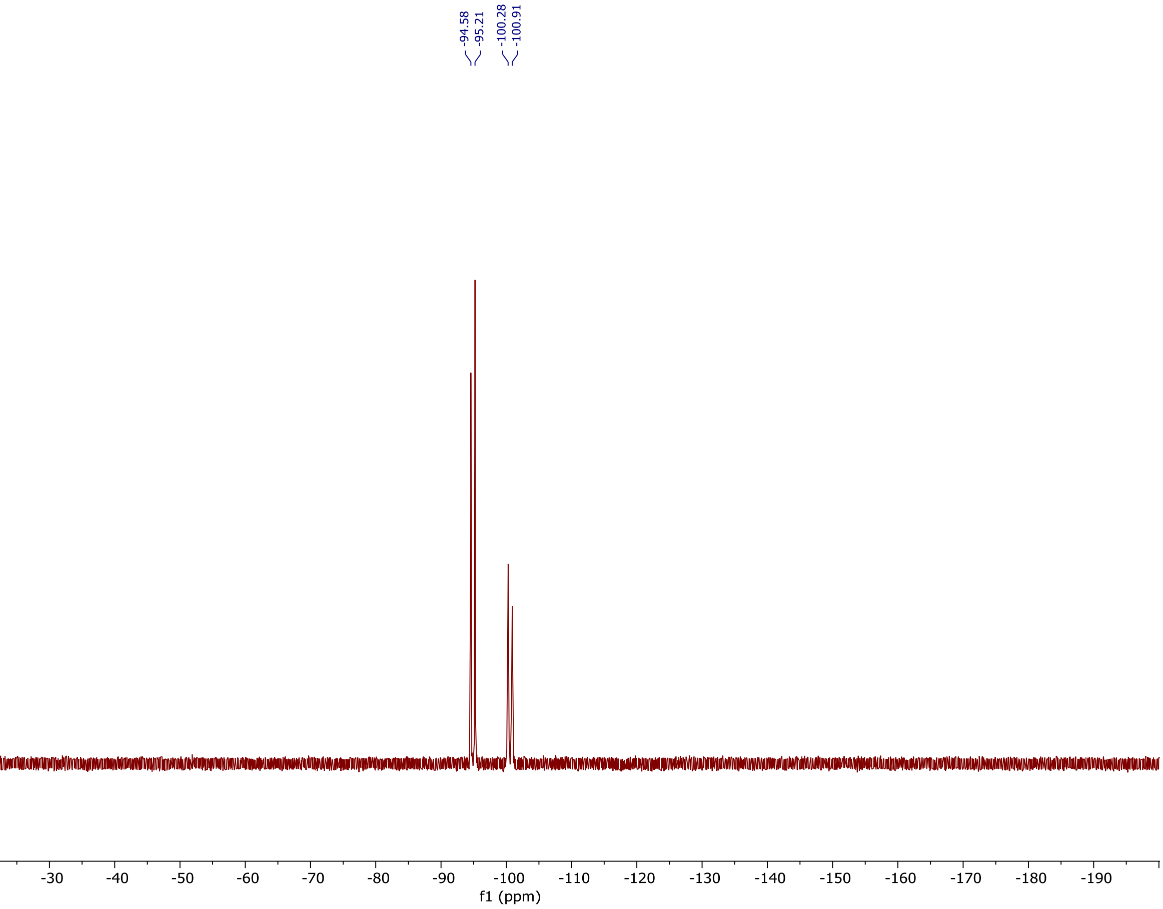

^{1}H NMR (400 MHz, $CDCl_3$) of **66** (see procedure)

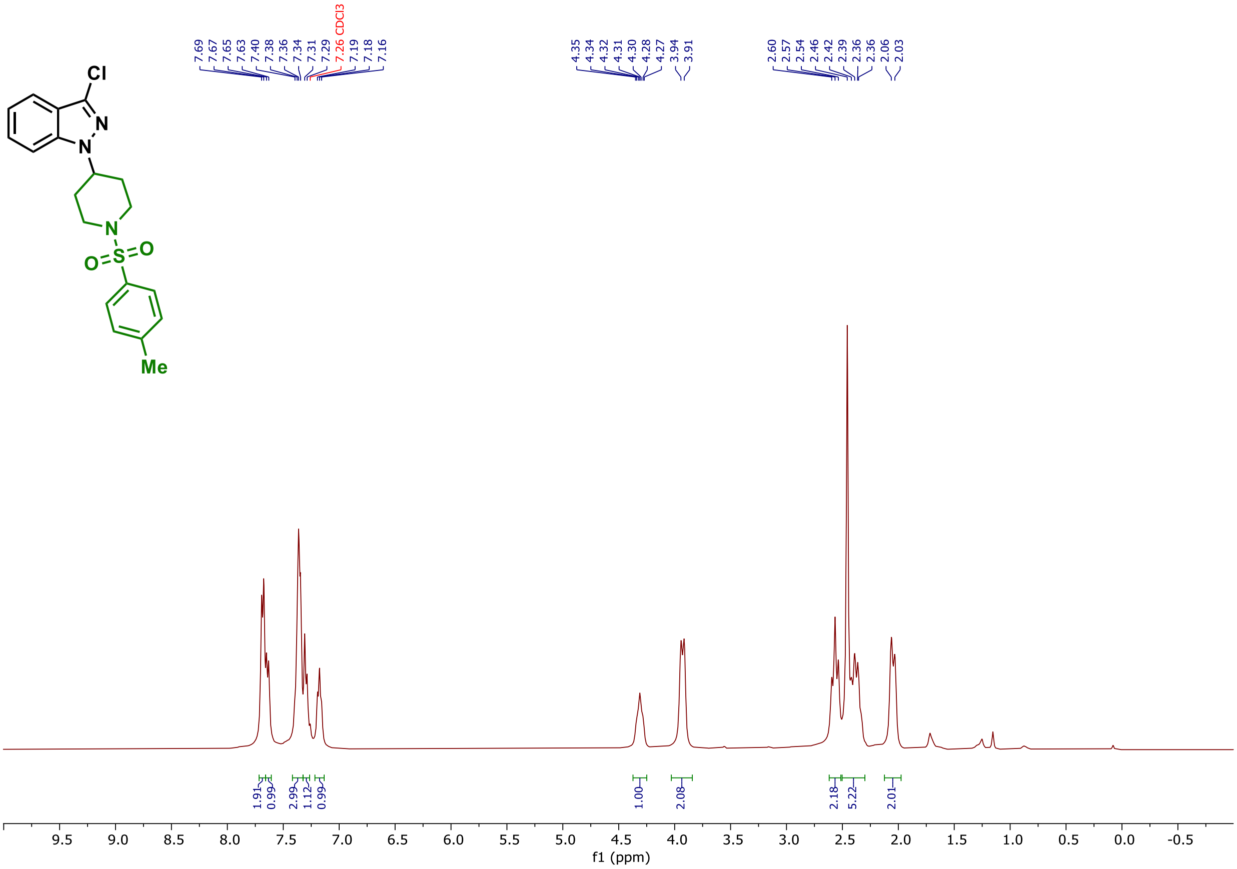

^{13}C NMR (101 MHz, $CDCl_3$) of **66**

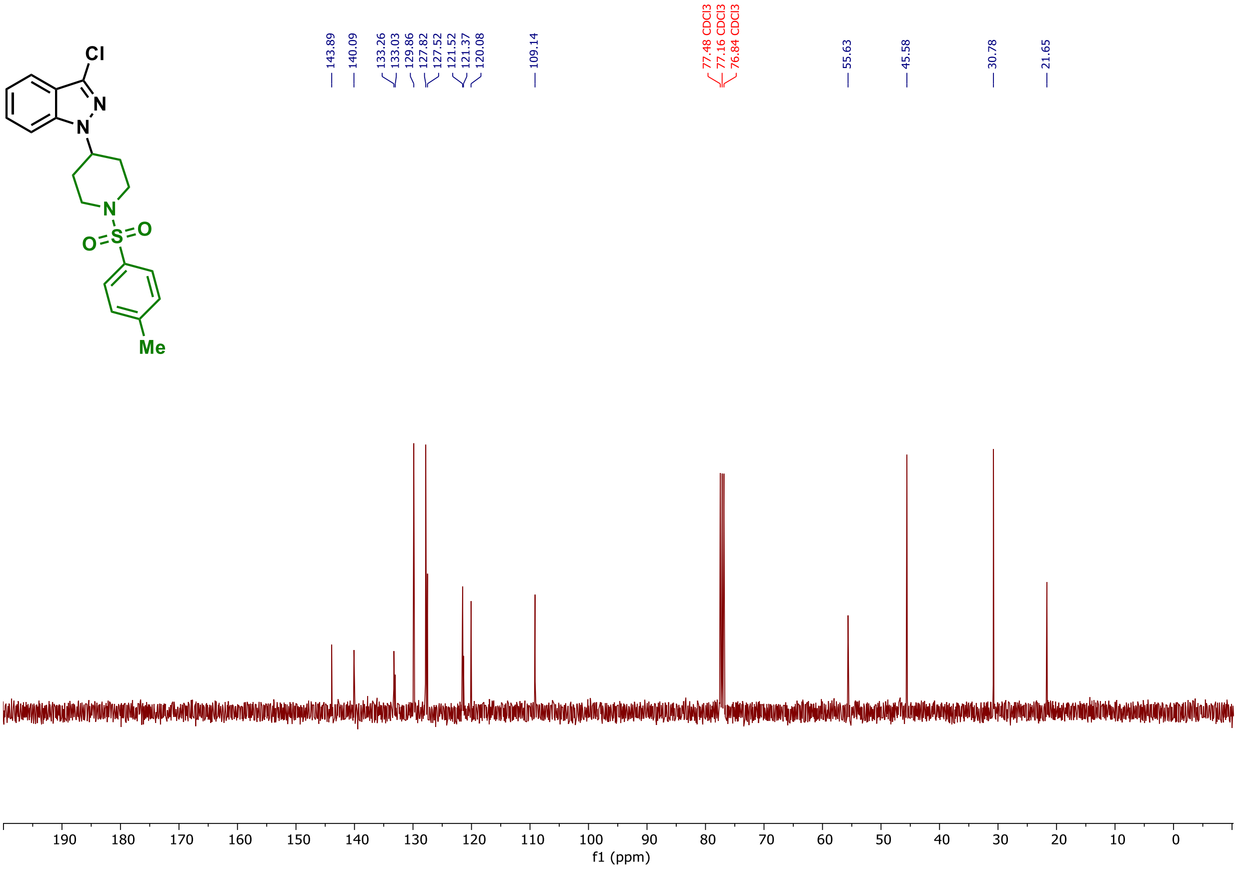

^{1}H NMR (400 MHz, $CDCl_3$) of **67** (*see procedure*)

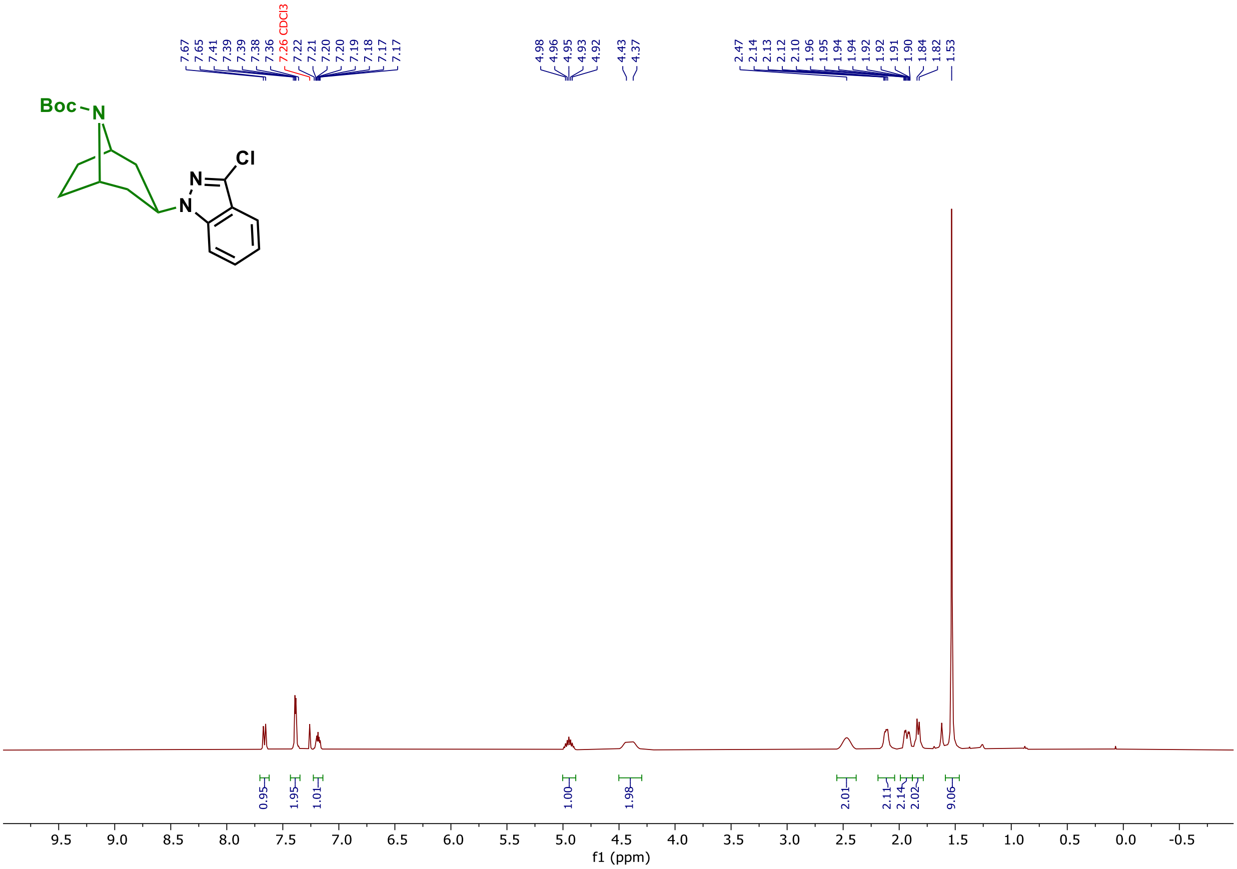

^{13}C NMR (101 MHz, $CDCl_3$) of **67**

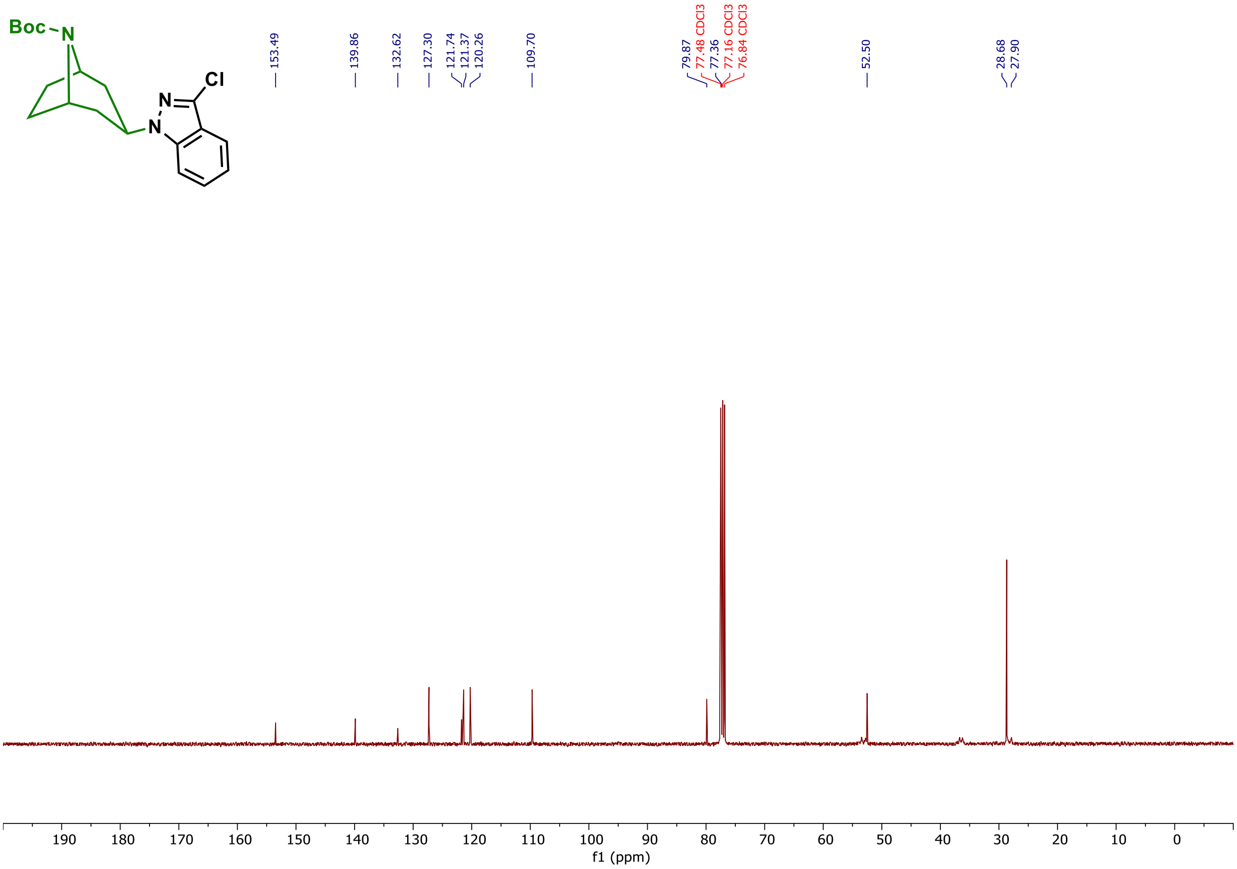

^{1}H NMR (400 MHz, $CDCl_3$) of **68** (*see procedure*)

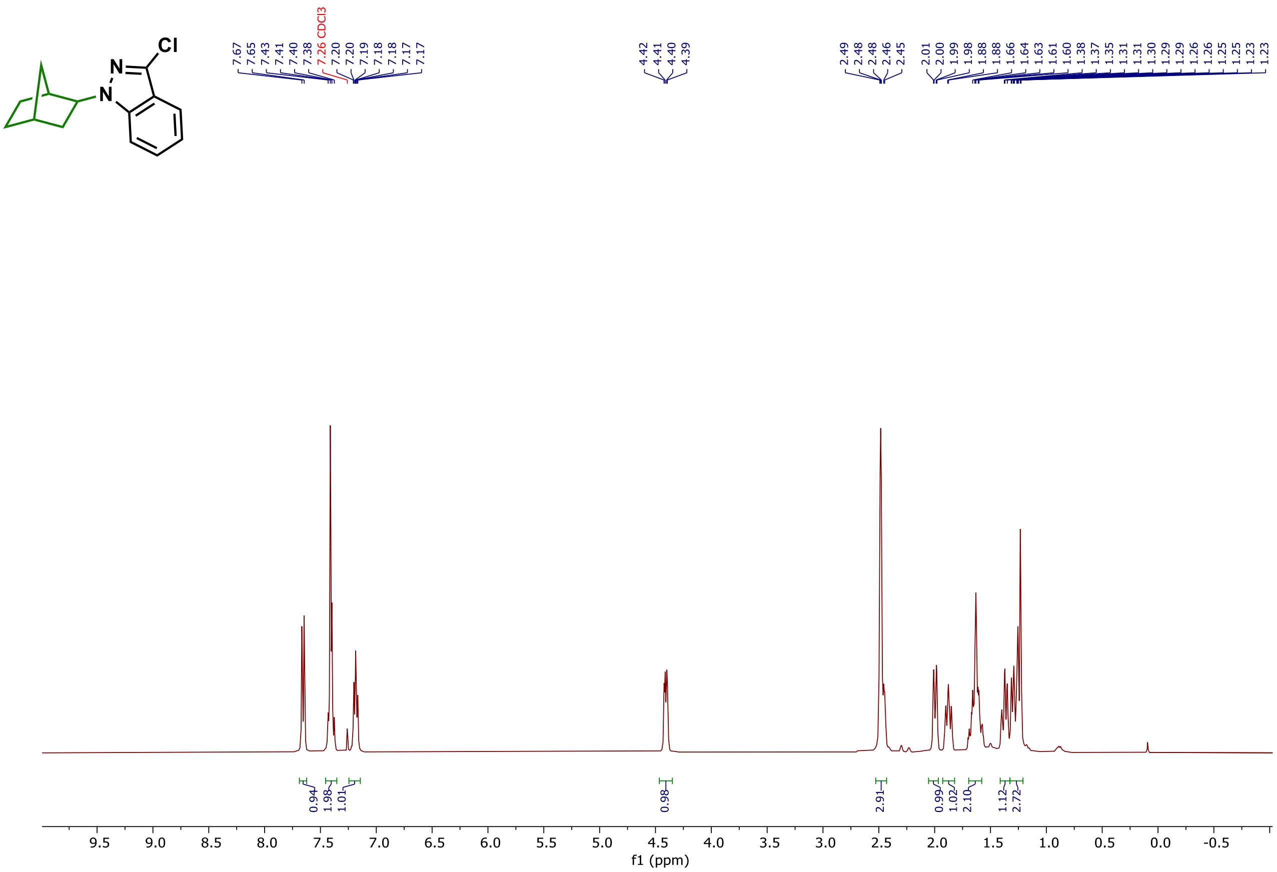

^{13}C NMR (101 MHz, $CDCl_3$) of **68**

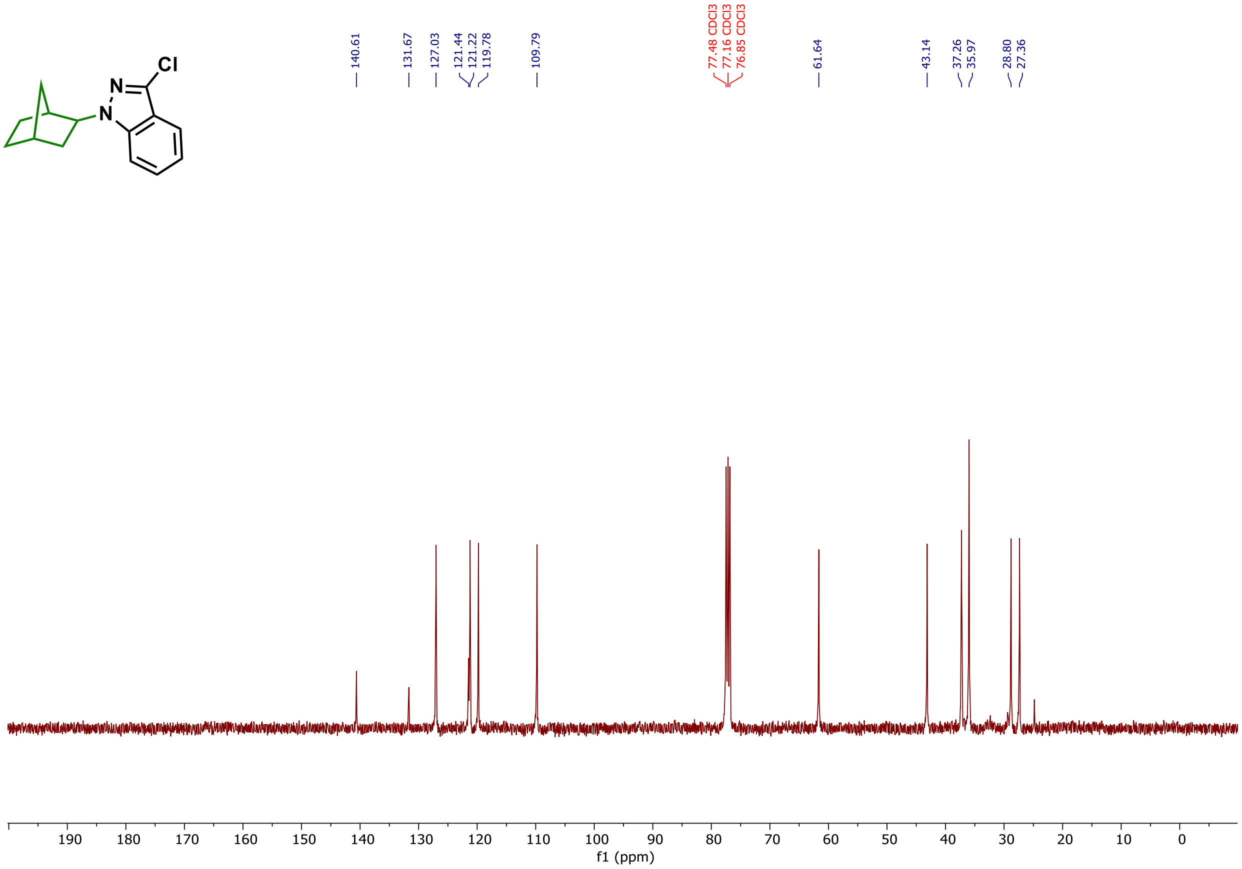

^{1}H NMR (400 MHz, $CDCl_3$) of **69** (*see procedure*)

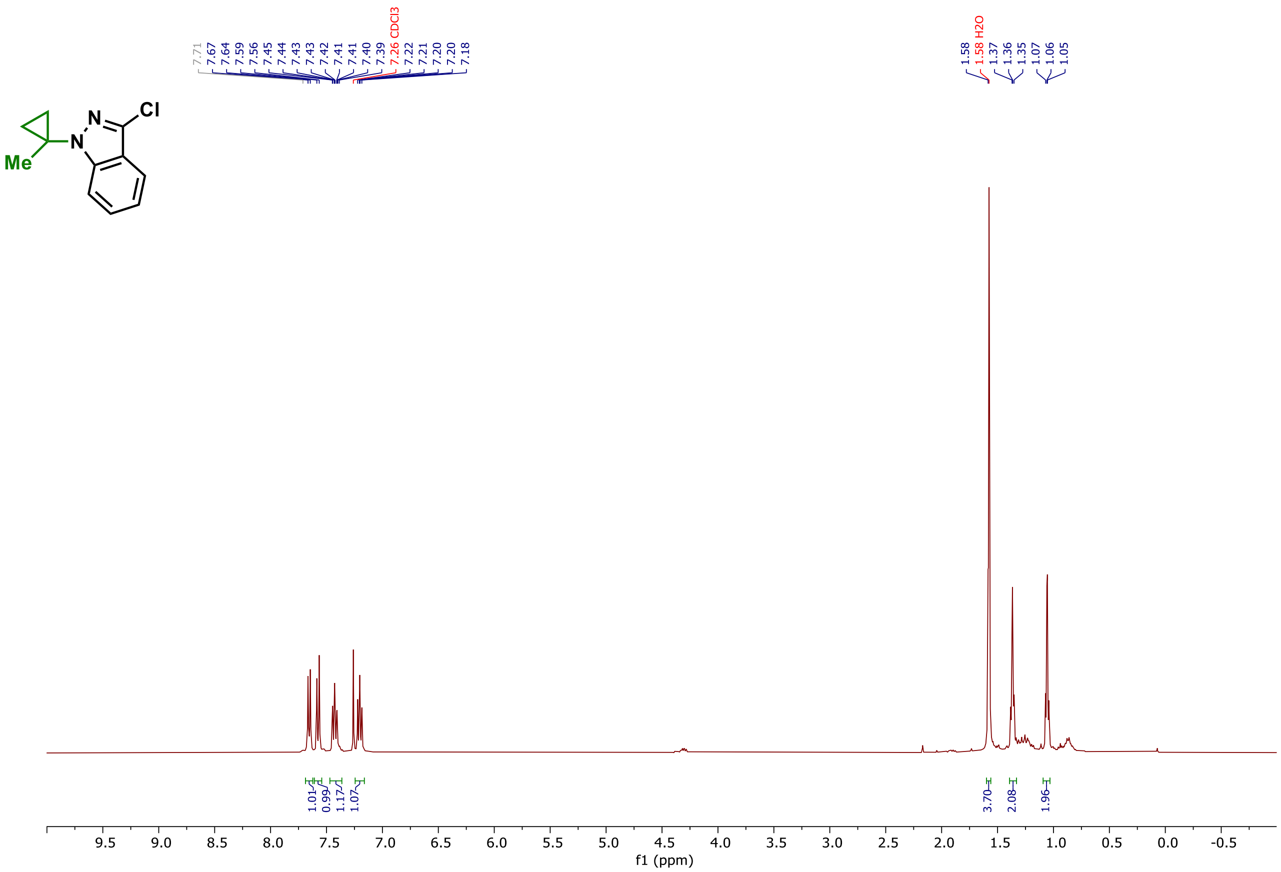

^{13}C NMR (101 MHz, $CDCl_3$) of **69**

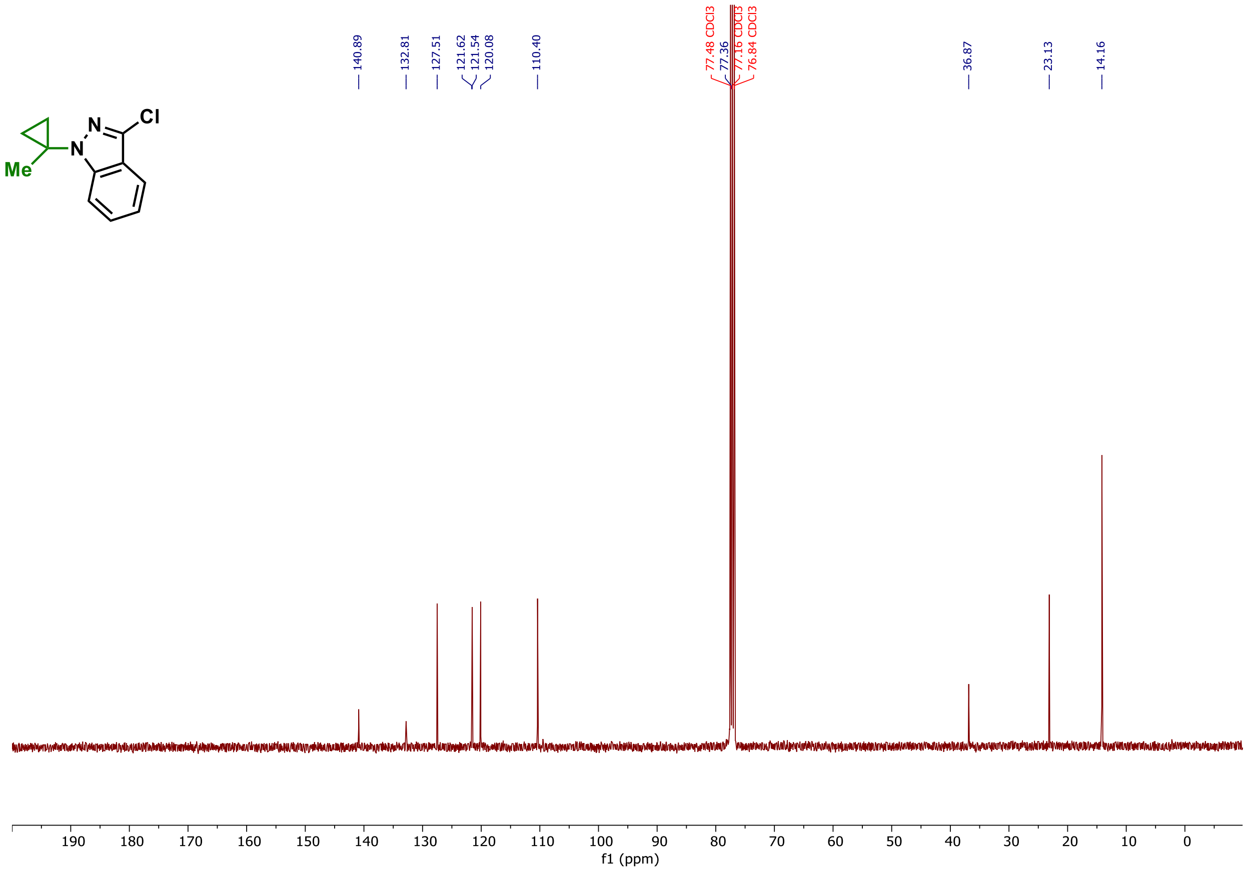

^{1}H NMR (400 MHz, $CDCl_3$) of **70** (see procedure)

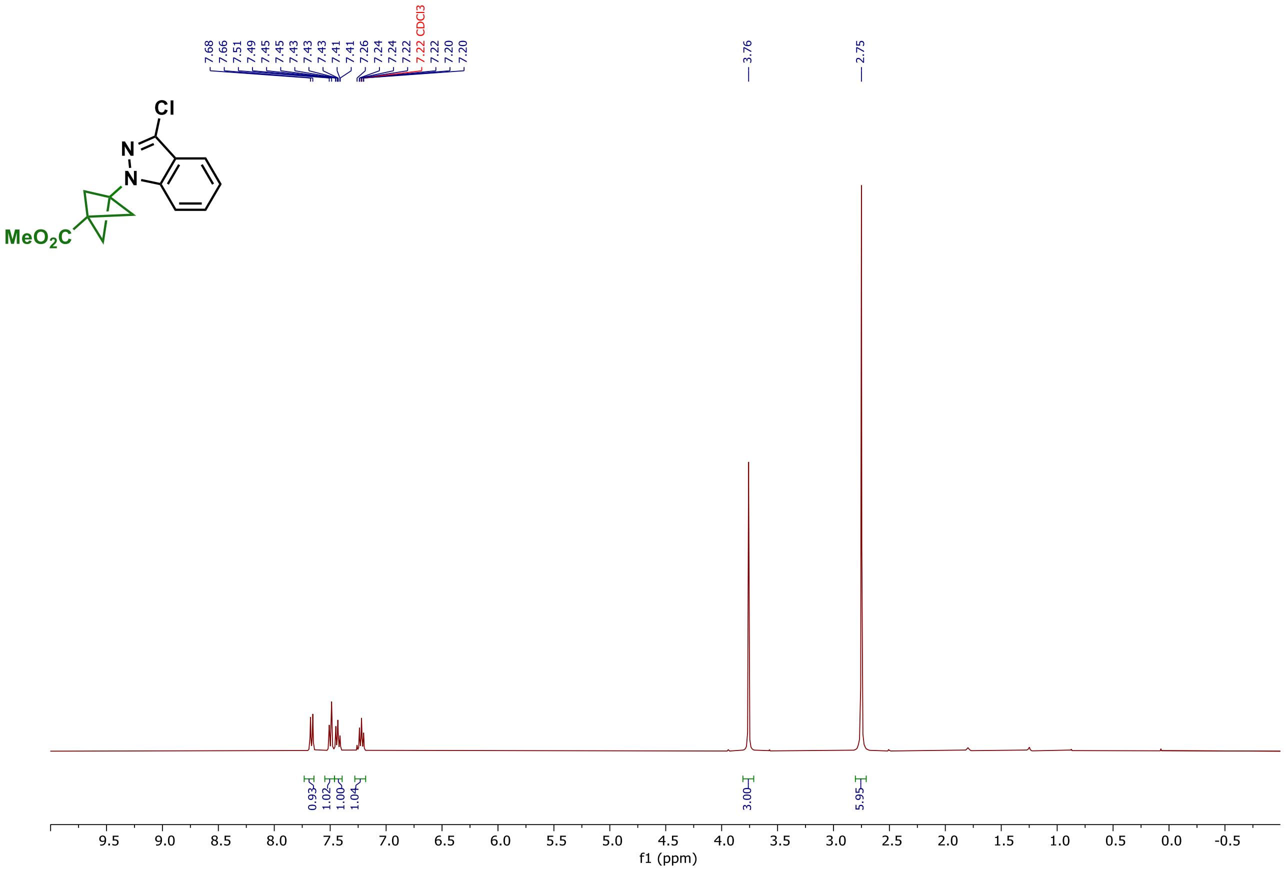

^{13}C NMR (101 MHz, $CDCl_3$) of **70**

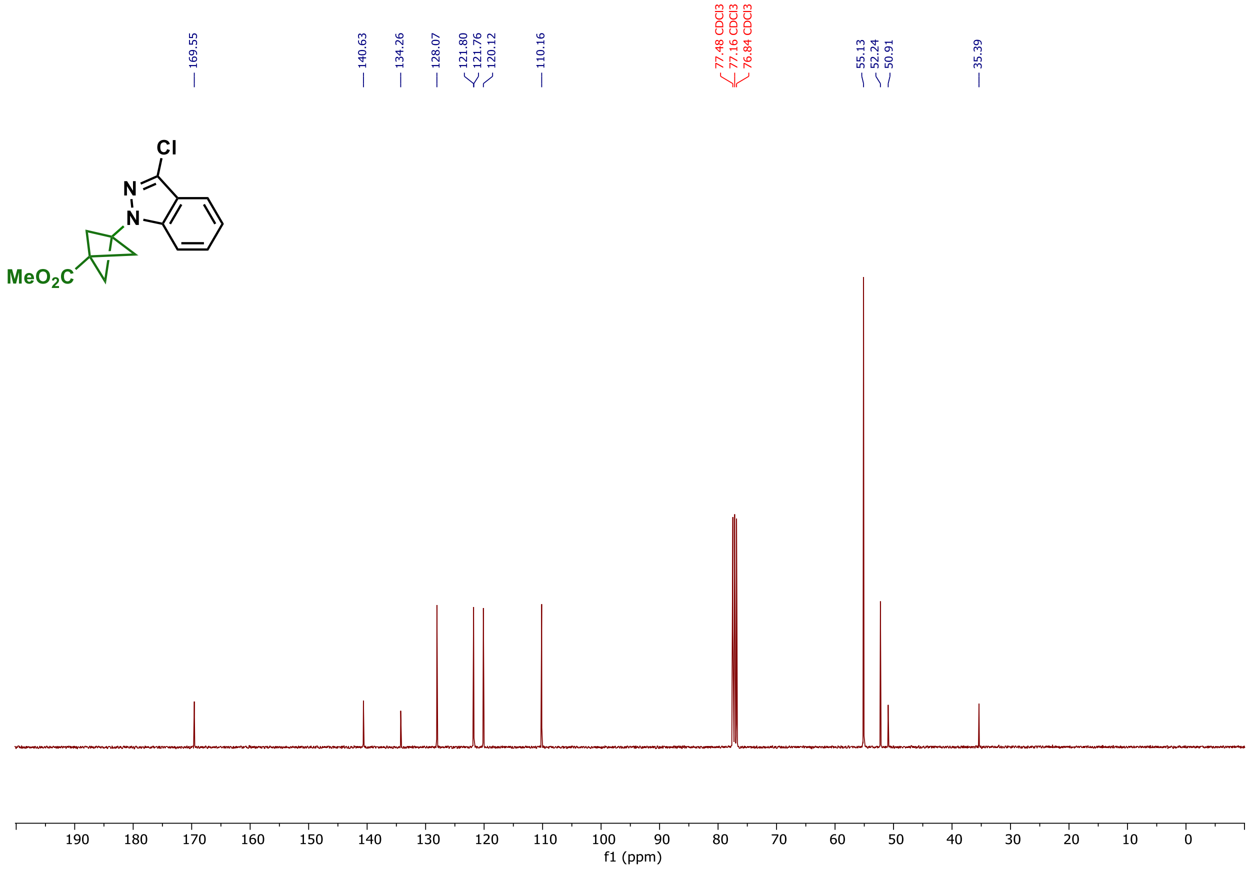

^1H NMR (400 MHz, $CDCl_3$) of **71** (*see procedure*)

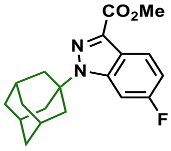

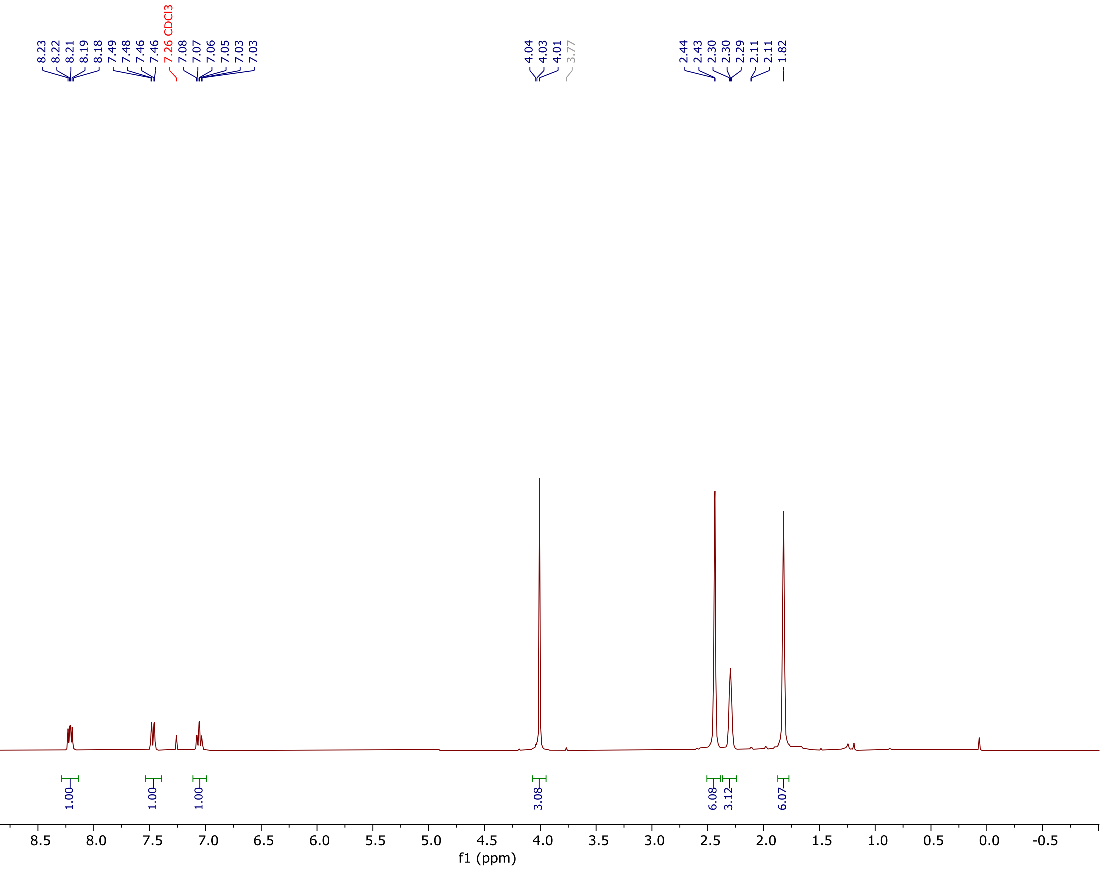

^{13}C NMR (101 MHz, $CDCl_3$) of **71**

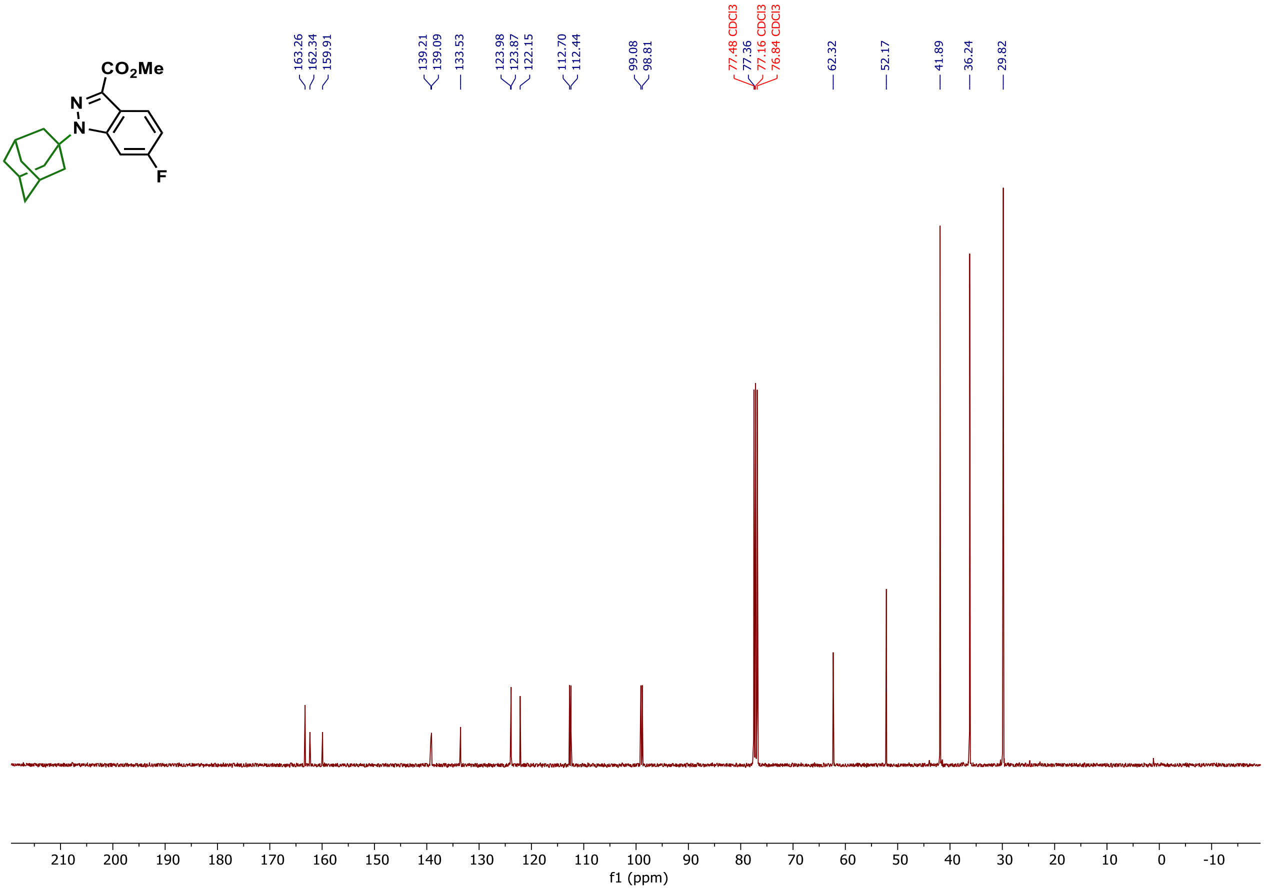

^{19}F NMR (376 MHz, $CDCl_3$) of **71**

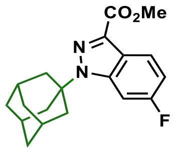

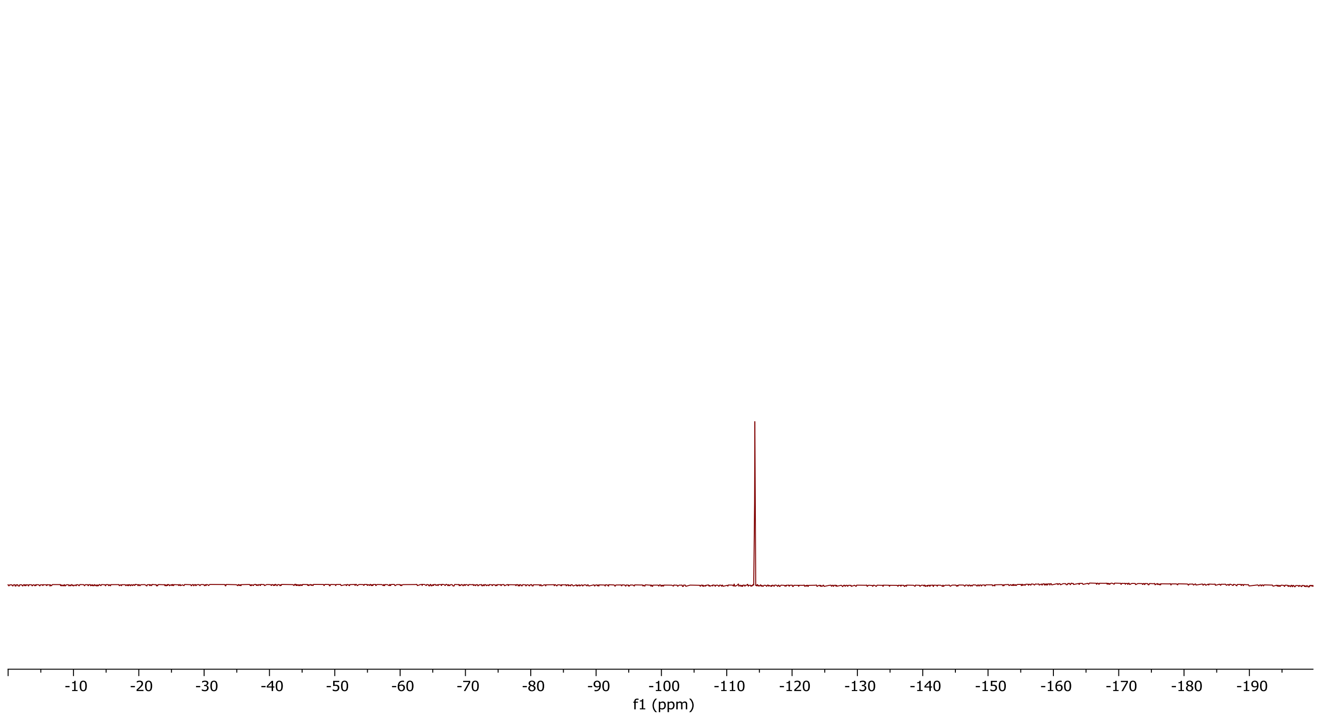

^{1}H NMR (400 MHz, CD_3Cl) of **72** (*see procedure*)

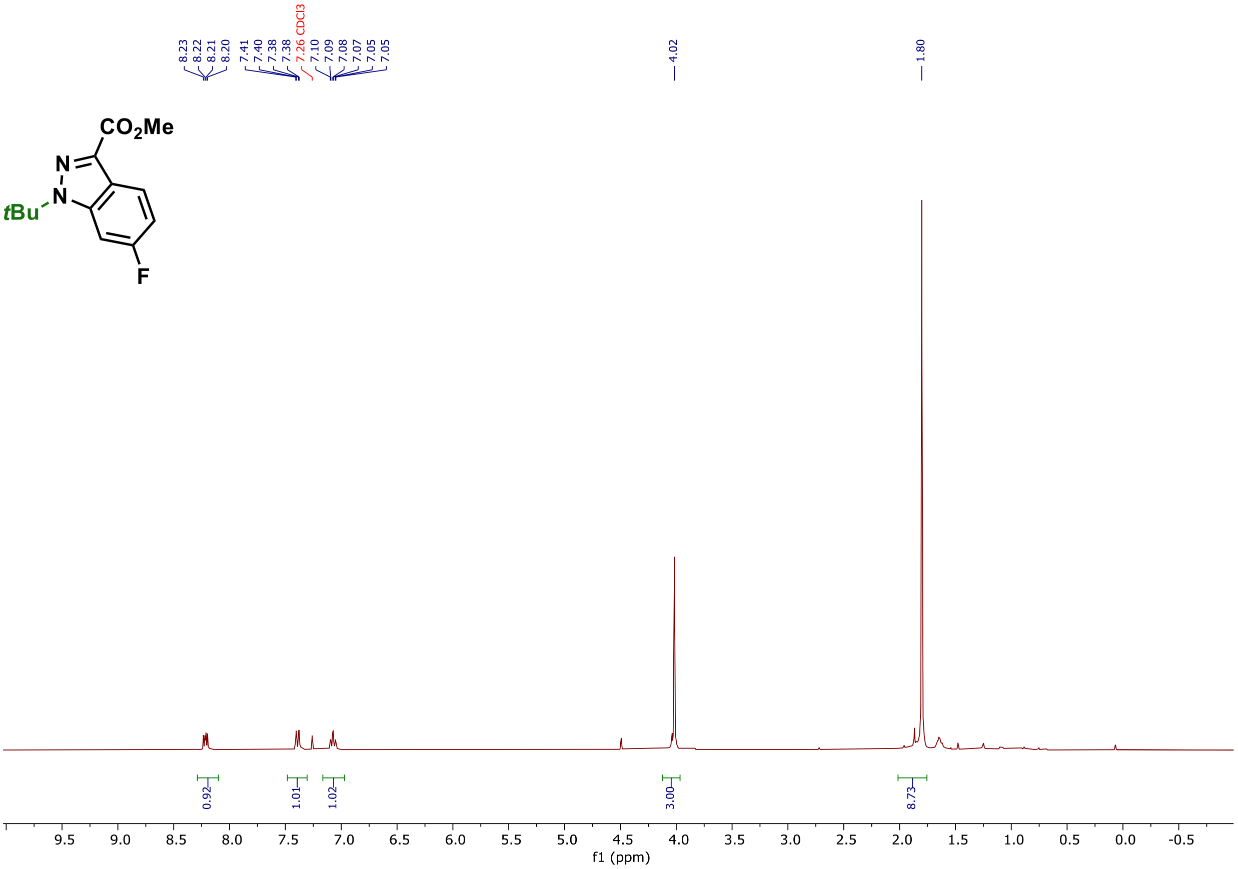

^{13}C NMR (101 MHz, $CDCl_3$) of **72**

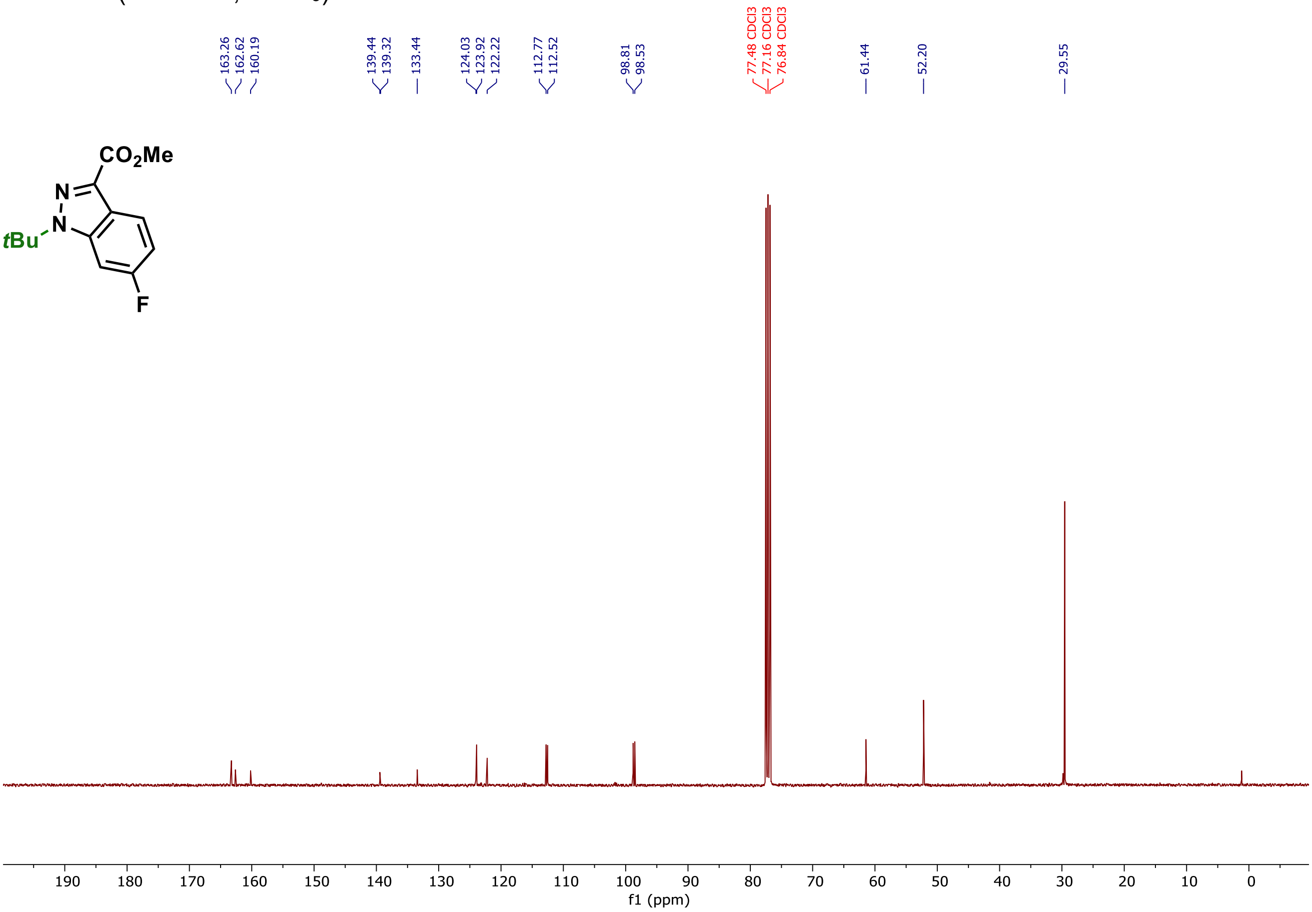

^{19}F NMR (376 MHz, $CDCl_3$) of **72**

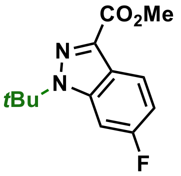

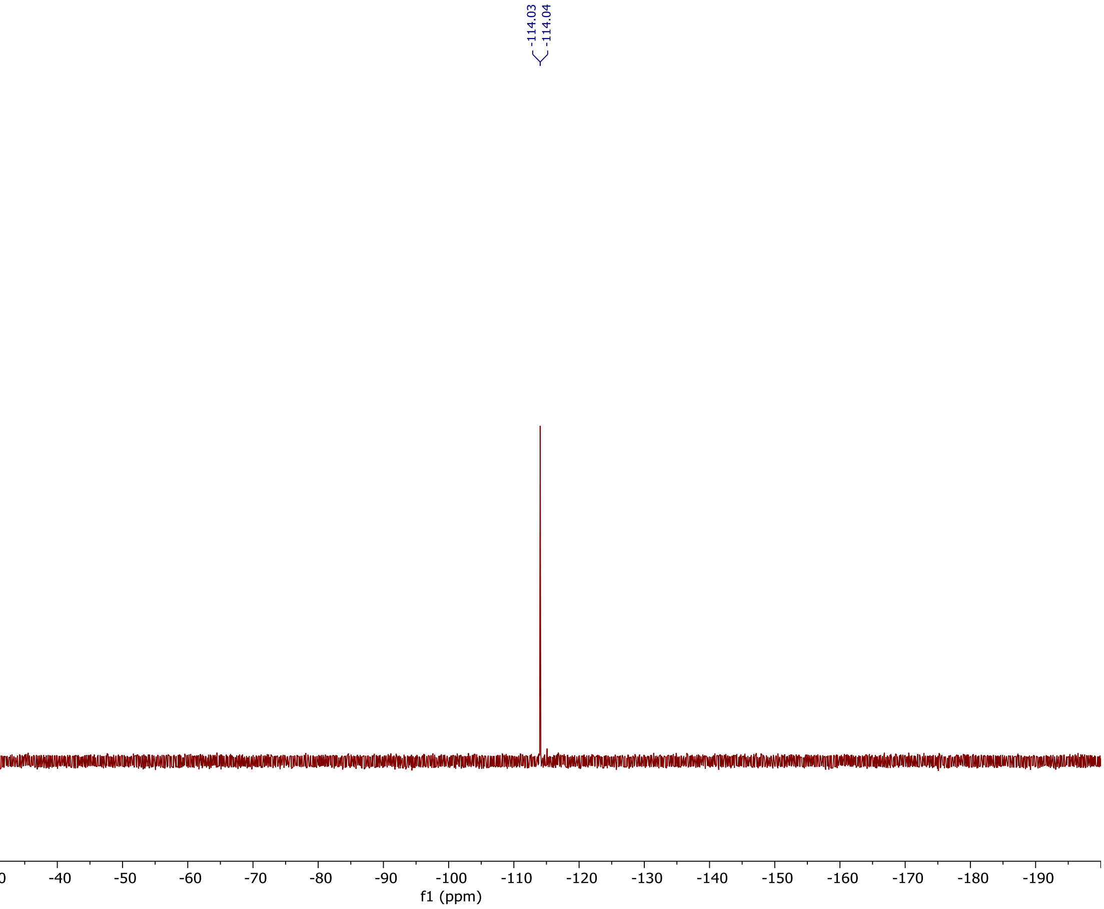

^{1}H NMR (400 MHz, *d*$_6$-DMSO) of **73** (*see procedure*)

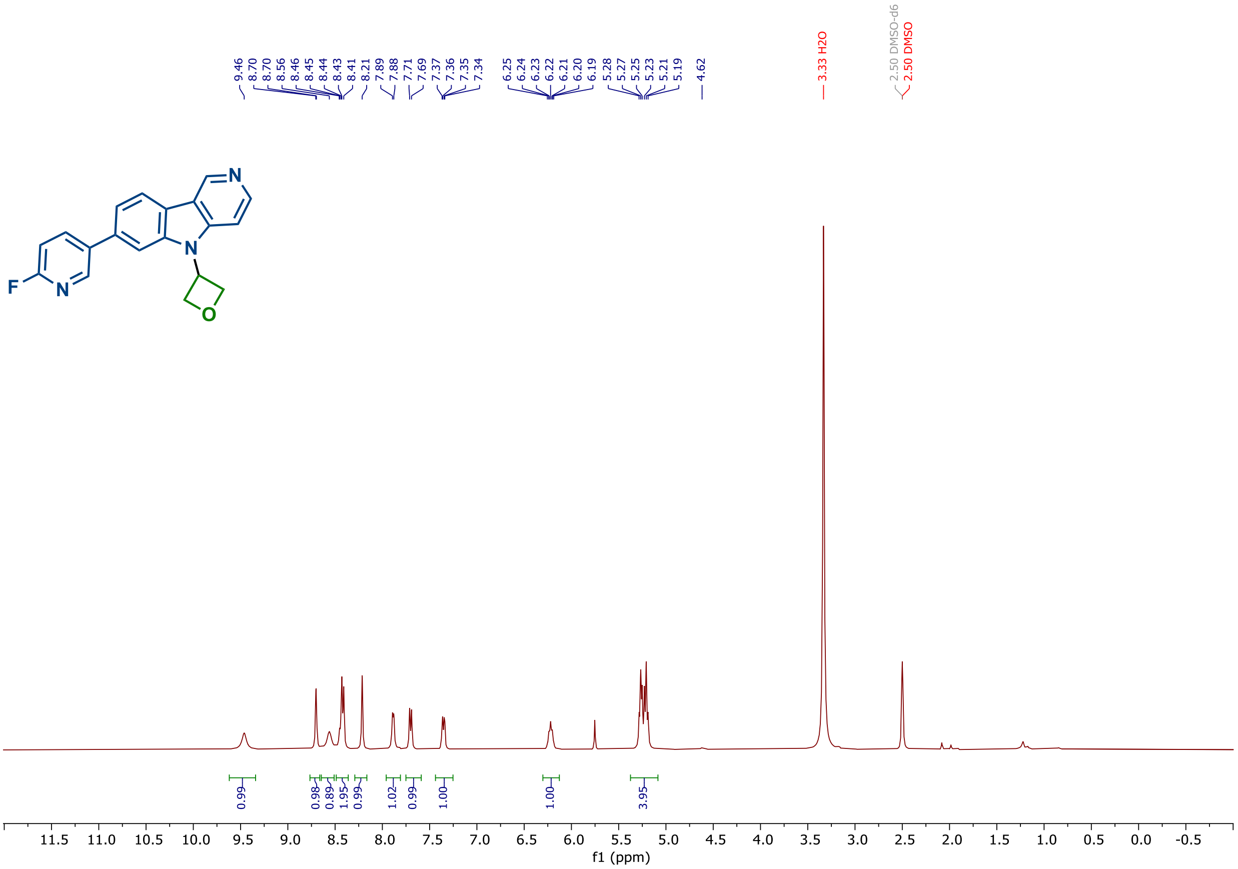

^{13}C NMR (101 MHz, *d*$_6$-DMSO) of **73**

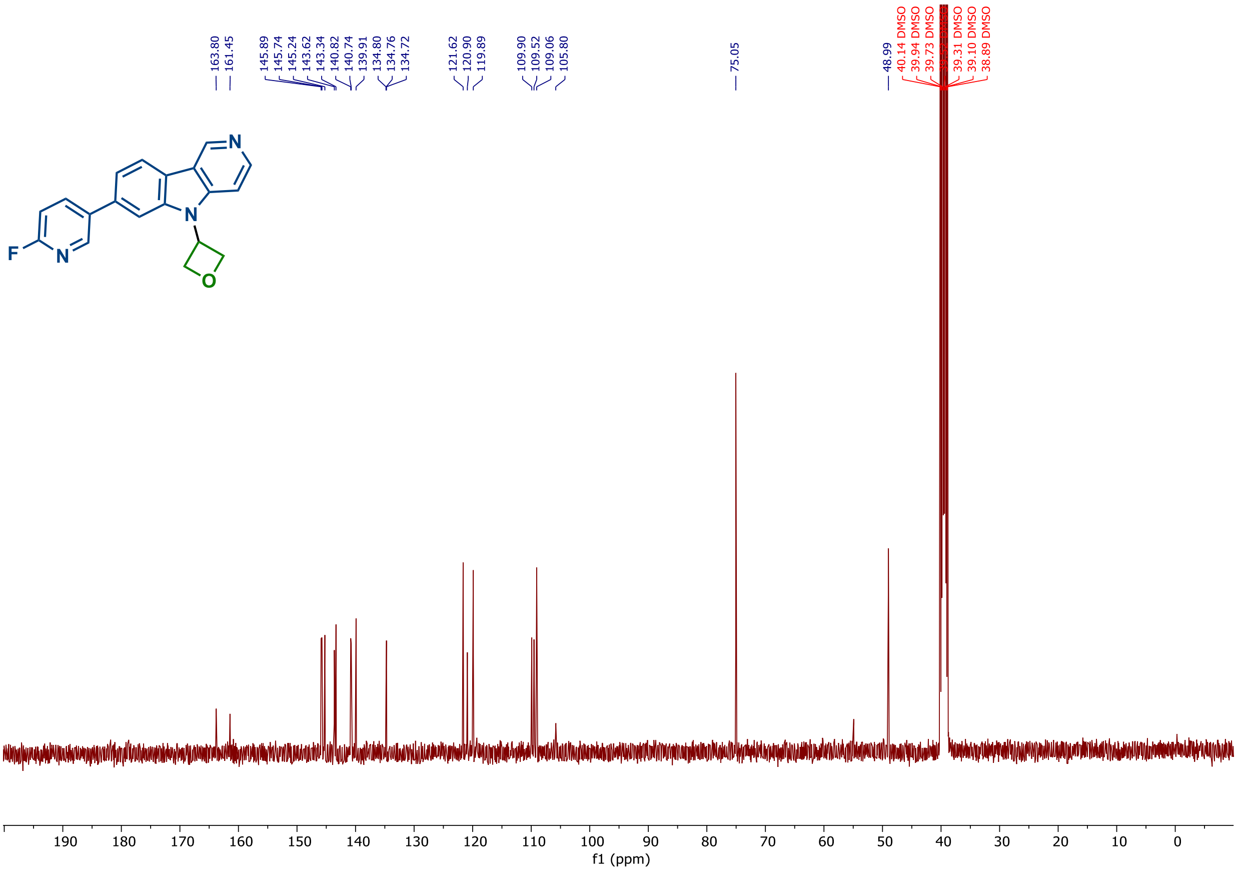

^{13}C NMR (101 MHz, $CDCl_3$) of **73**

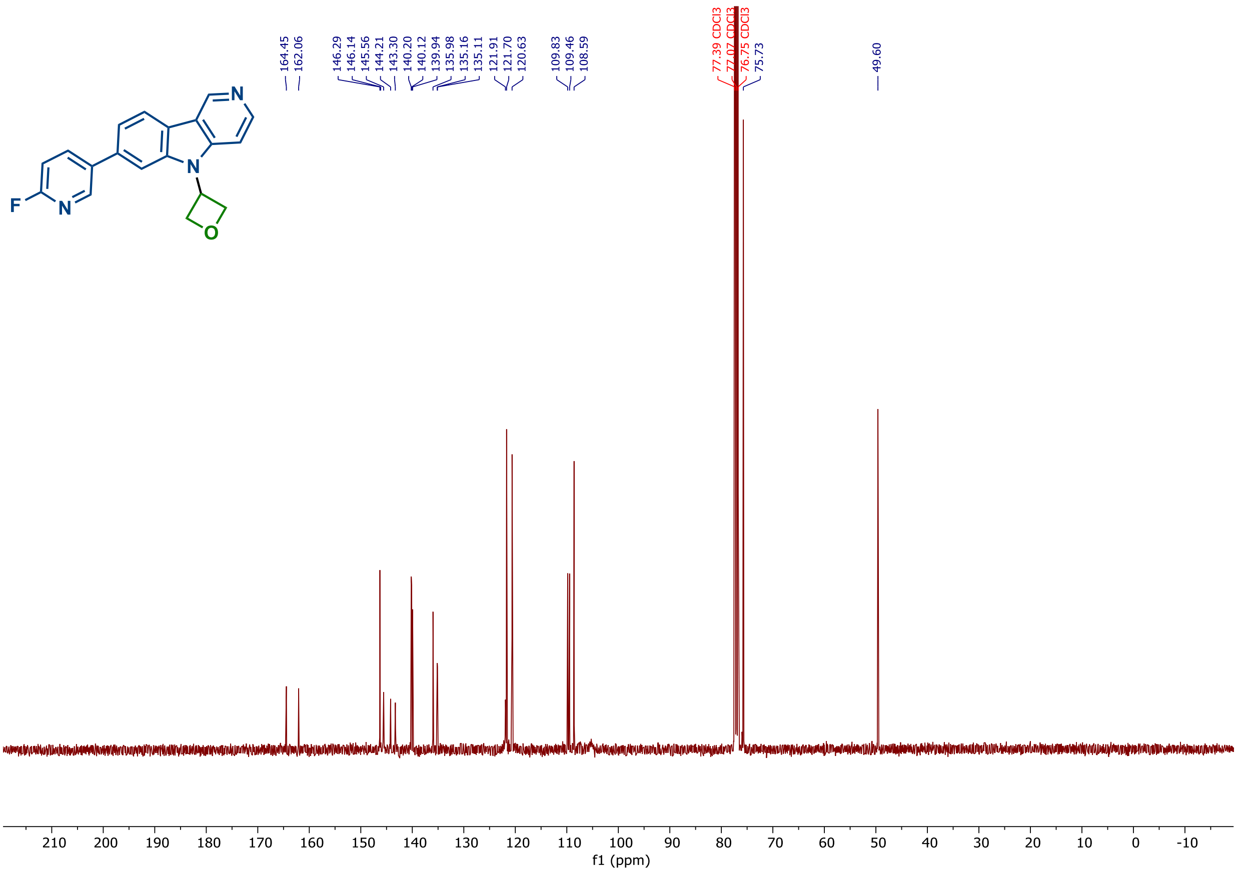

^{19}F NMR (376 MHz, $CDCl_3$) of **73**

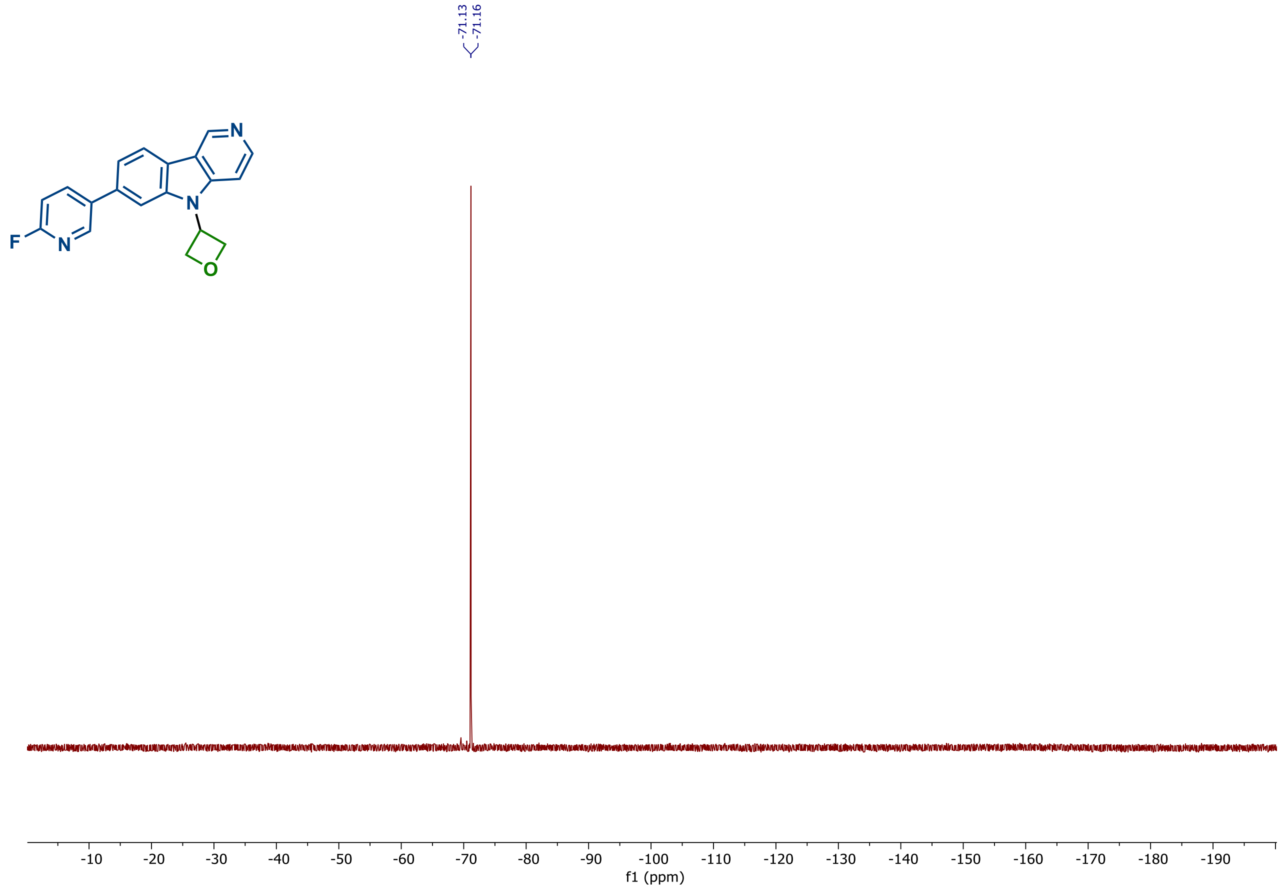

^{1}H NMR (400 MHz, $CDCl_3$) of **74** (*see procedure*)

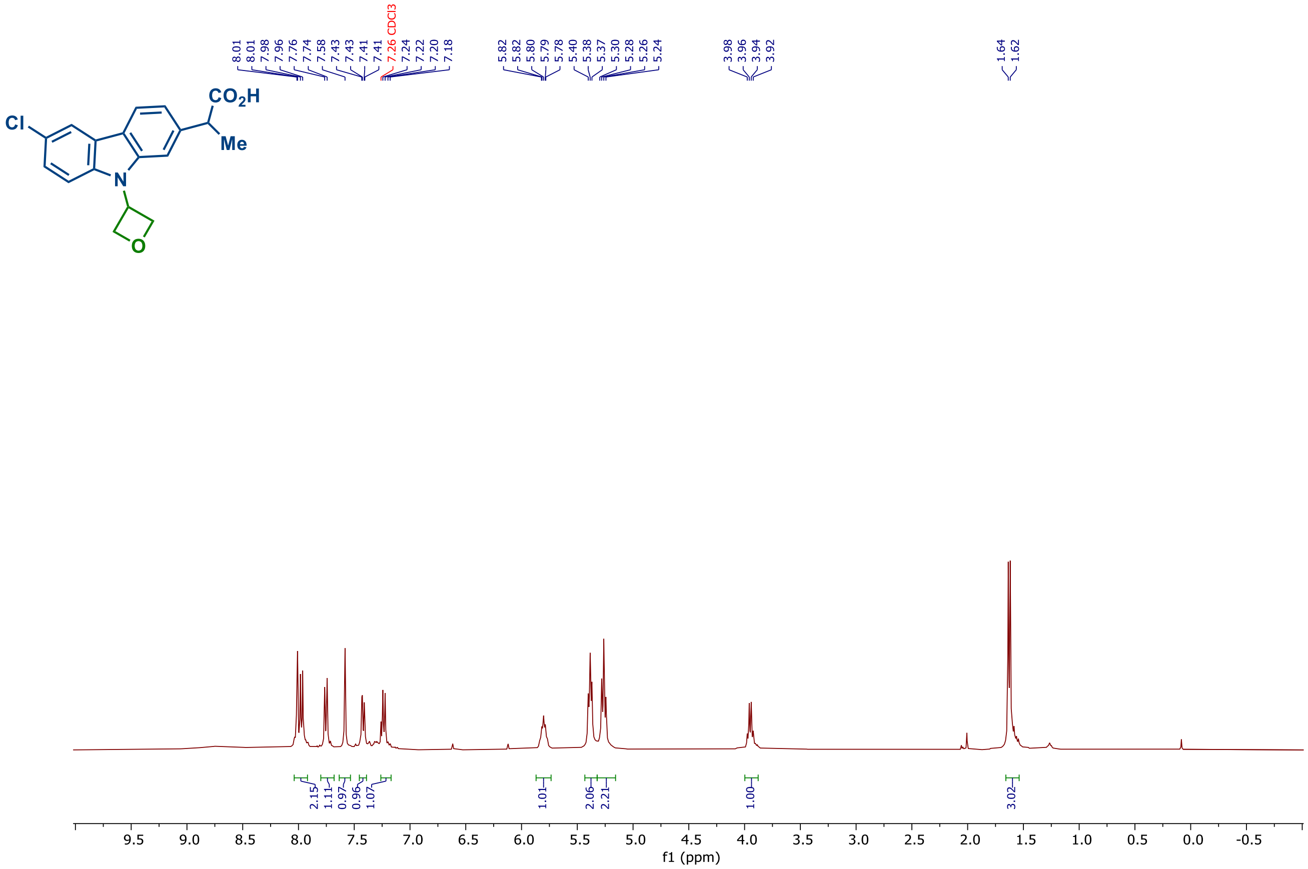

^{13}C NMR (101 MHz, $CDCl_3$) of **74**

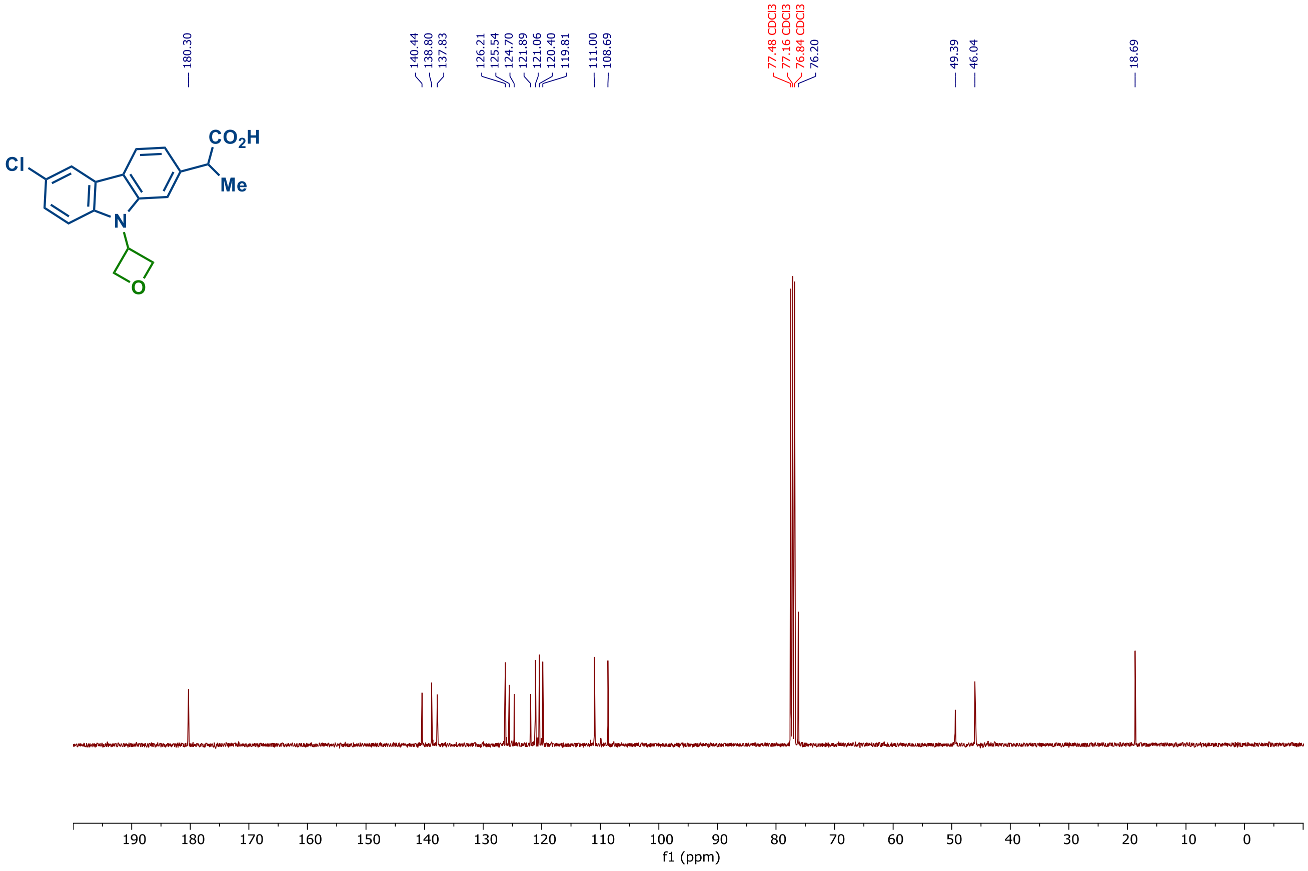

^{1}H NMR (400 MHz, $CDCl_3$) of **75** (see procedure)

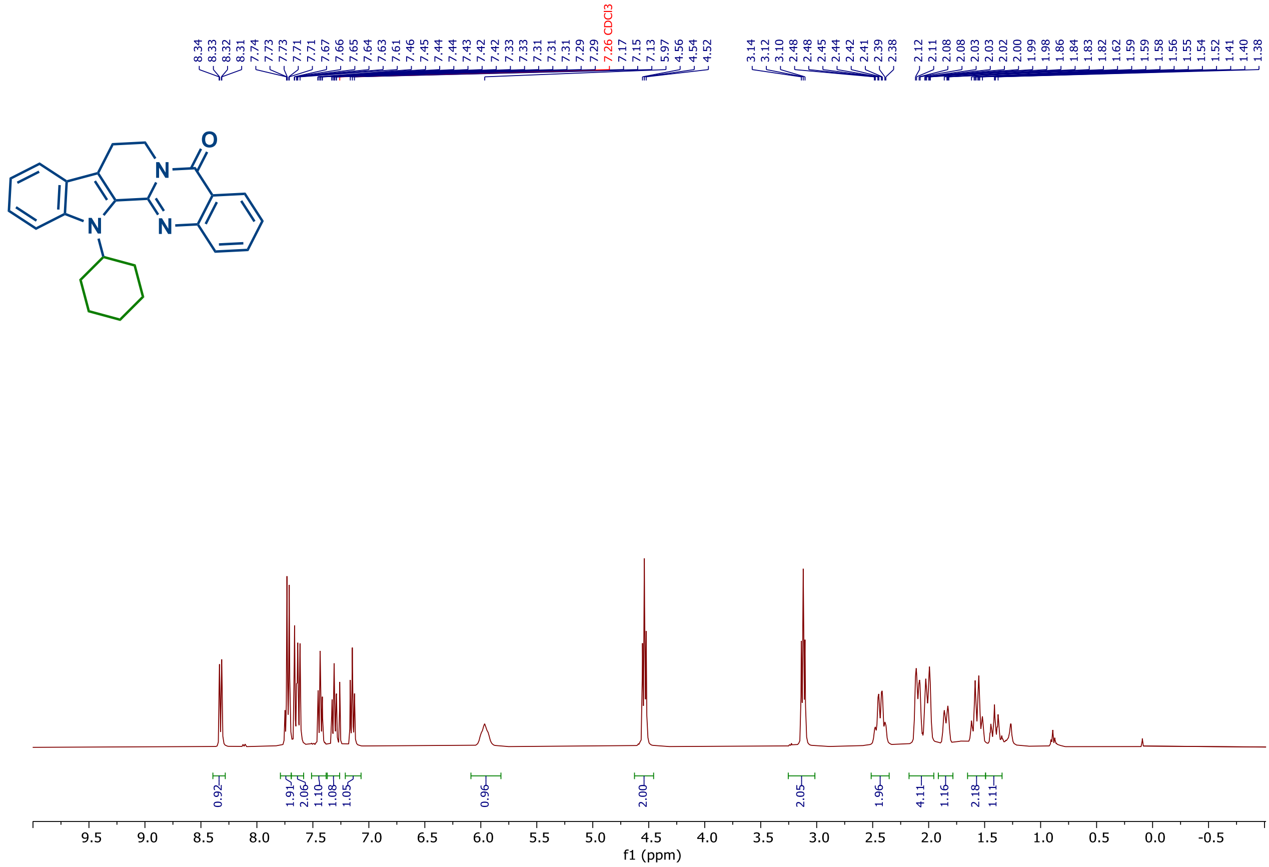

^{13}C NMR (101 MHz, $CDCl_3$) of **75**

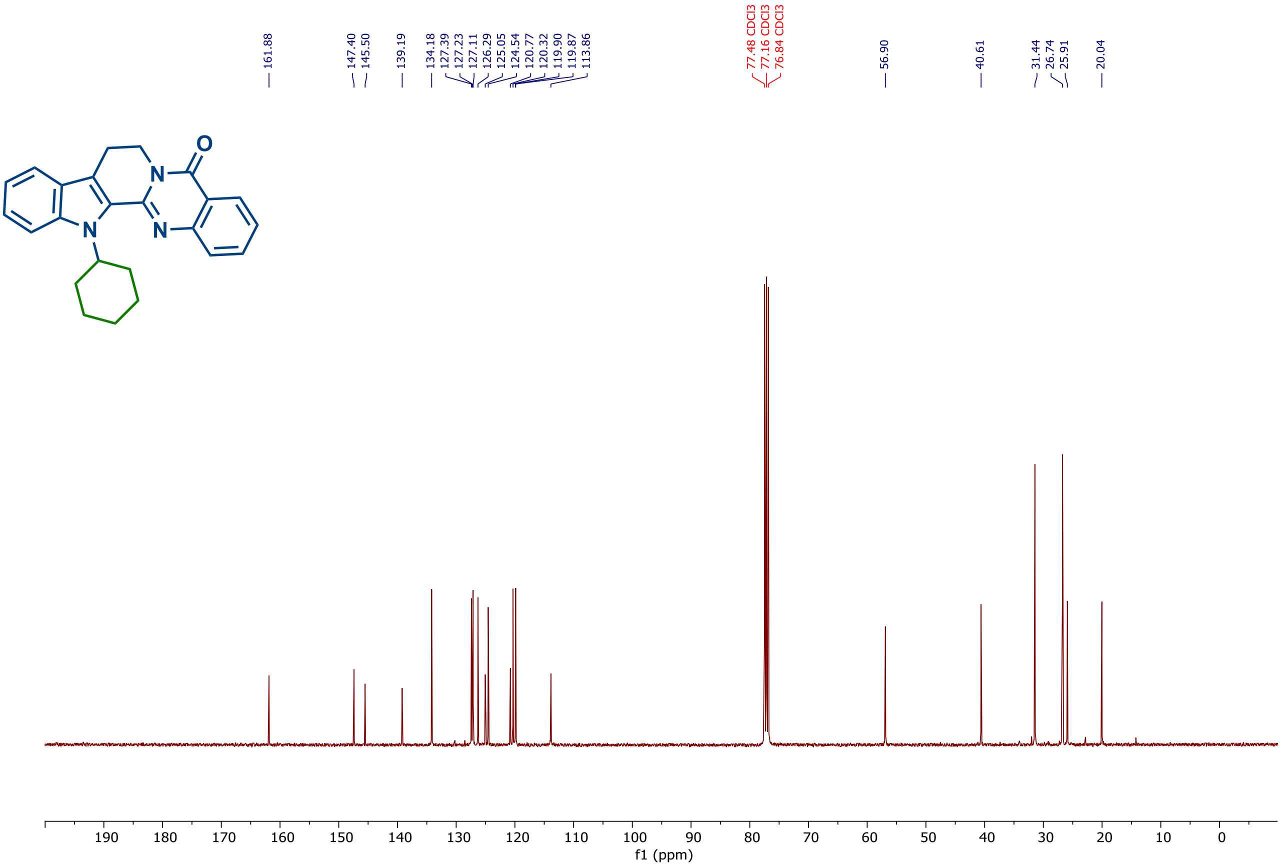

^{1}H NMR (400 MHz, CD_3OD) of **76** (*see procedure*)

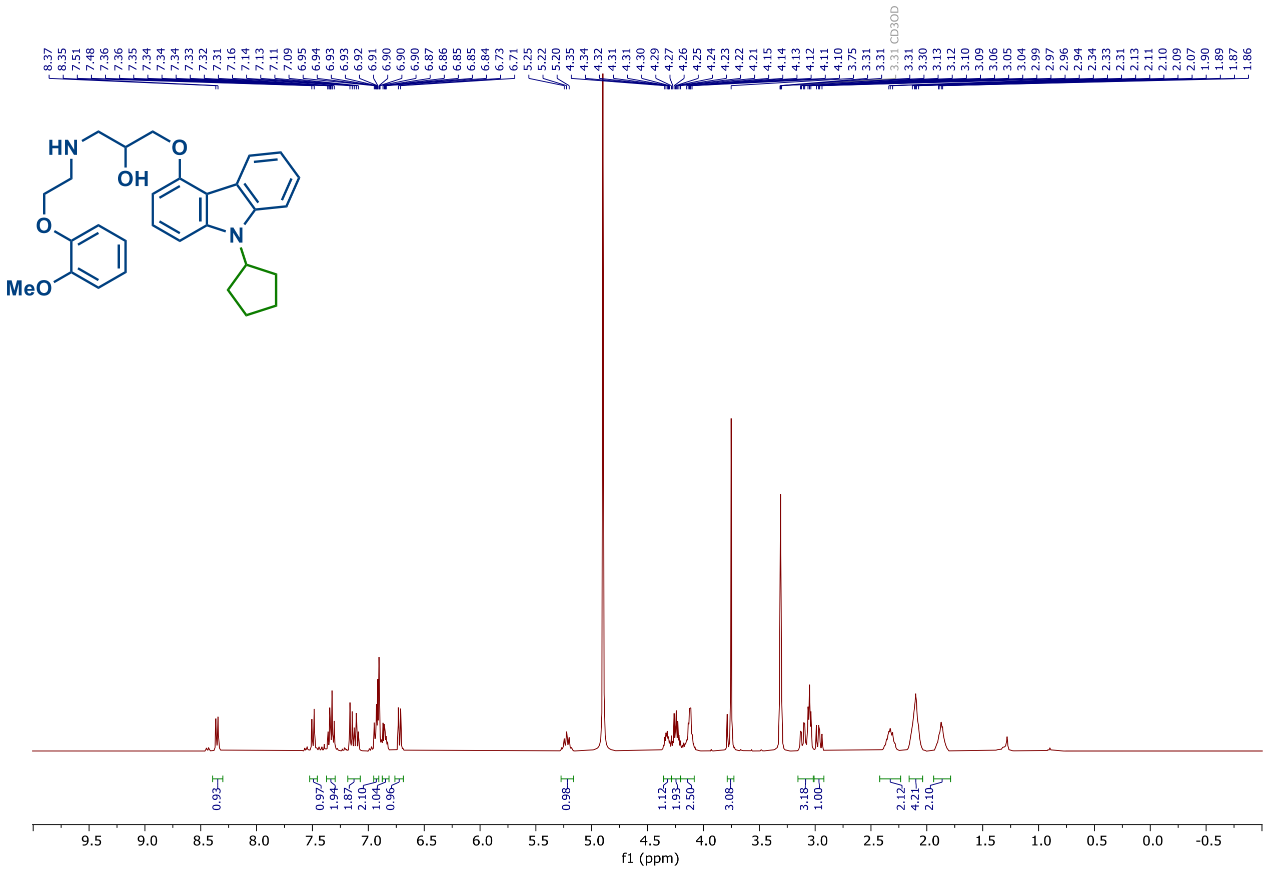

^{13}C NMR (101 MHz, CD_3OD) of **76**

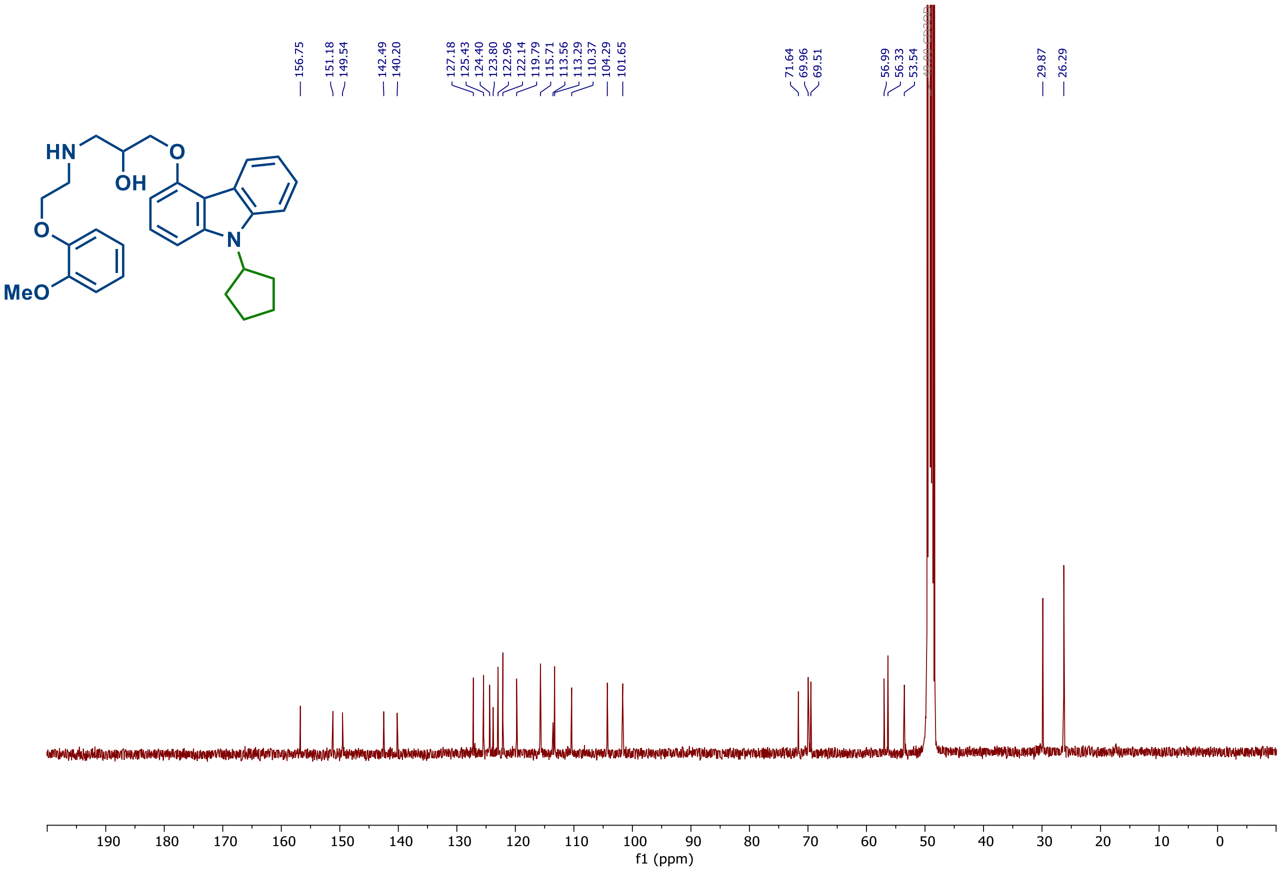

^{1}H NMR (400 MHz, $CDCl_3$) of **77** (*see procedure*)

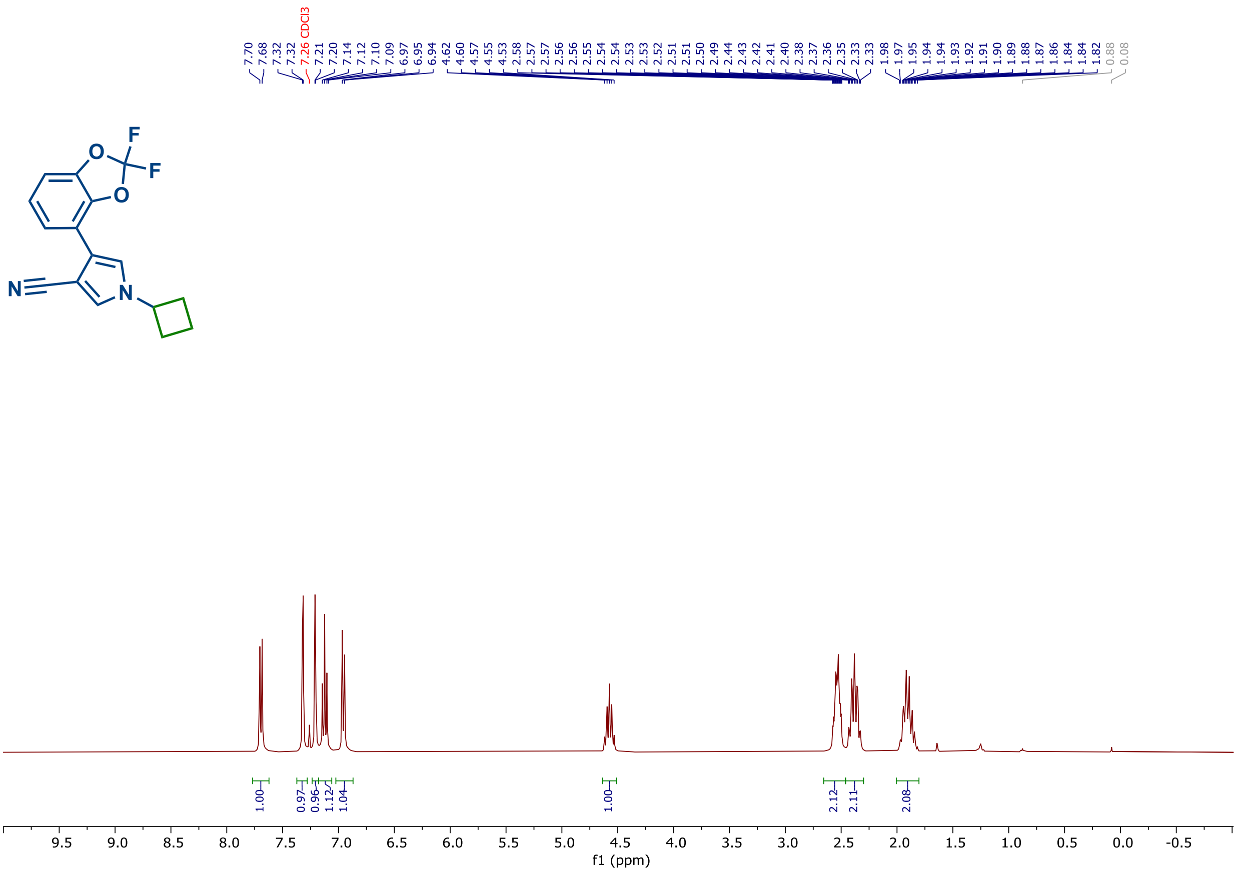

^{13}C NMR (101 MHz, $CDCl_3$) of **77**

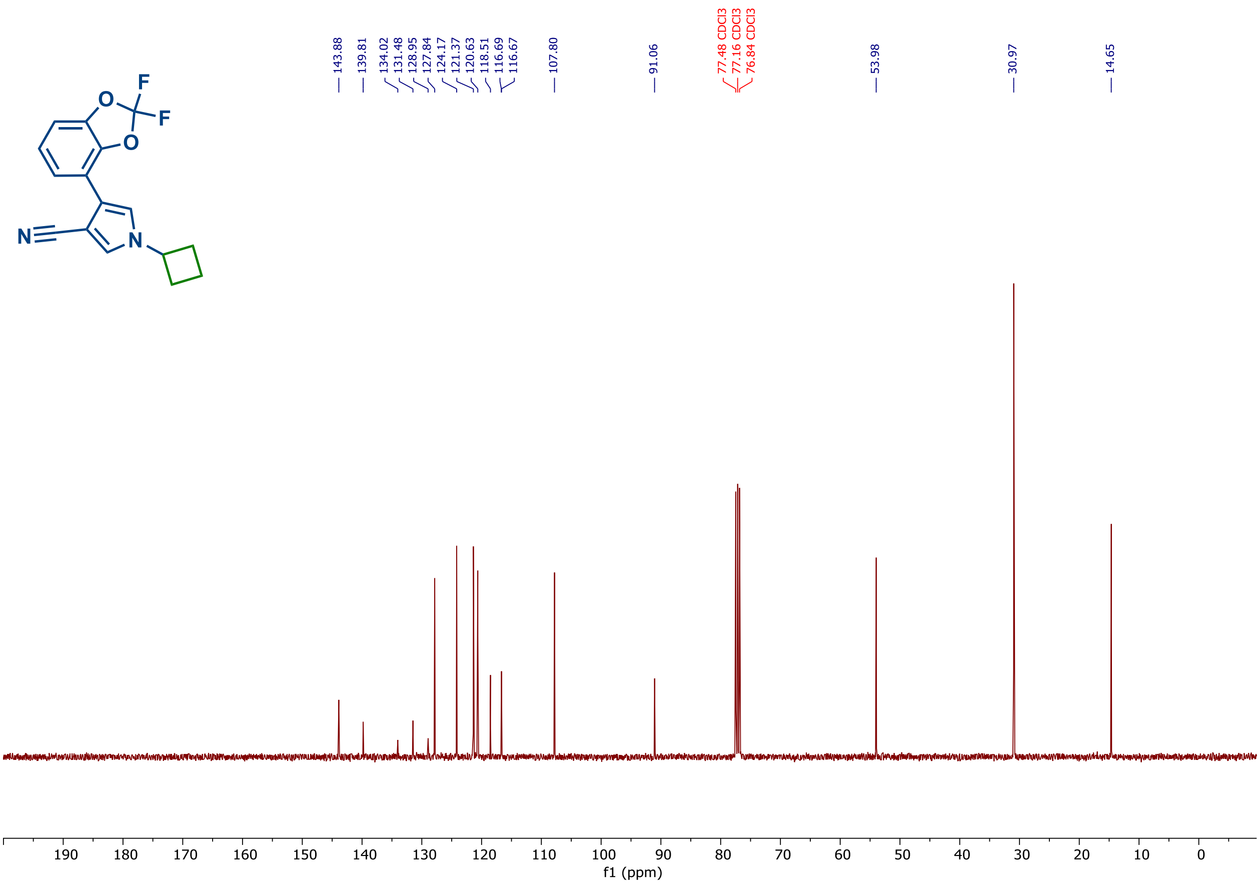

^{19}F NMR (376 MHz, $CDCl_3$) of **77**

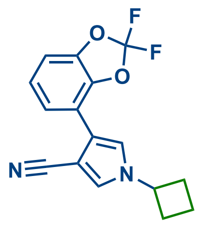

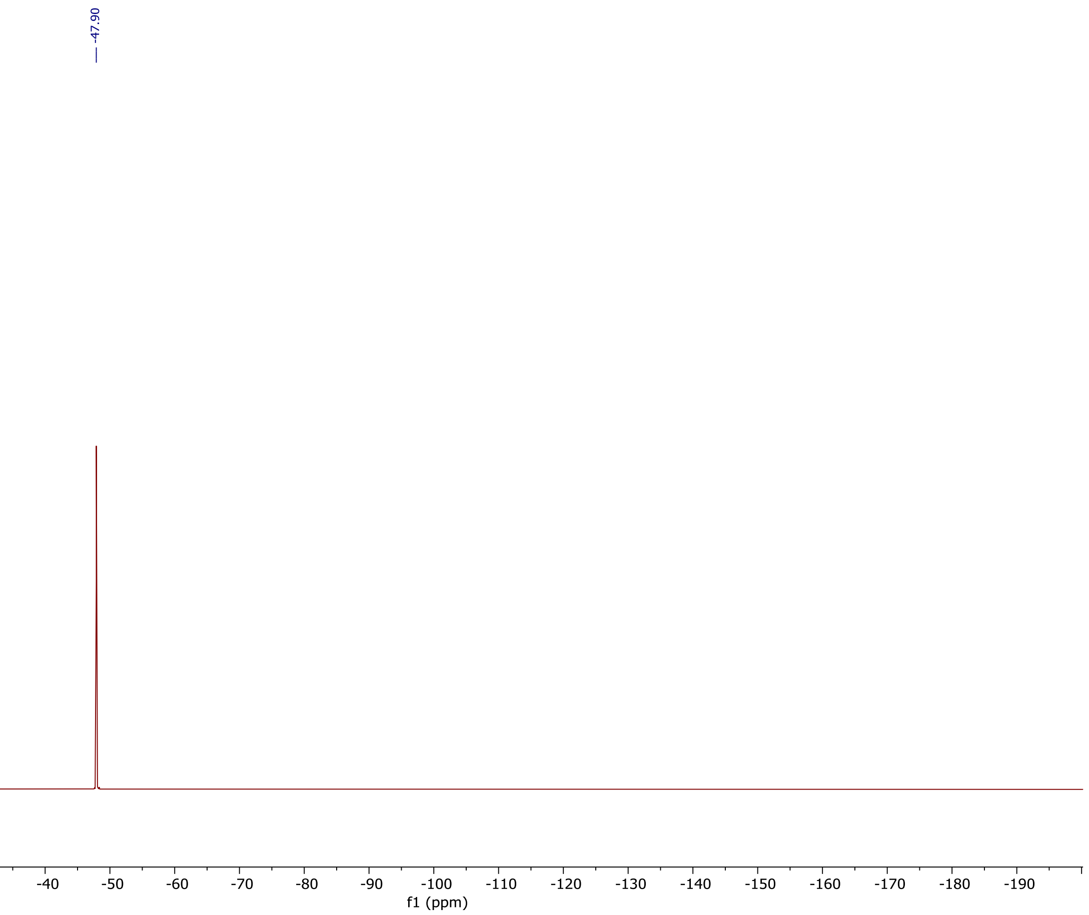

^{1}H NMR (400 MHz, $CDCl_3$) of **78** (*see procedure*)

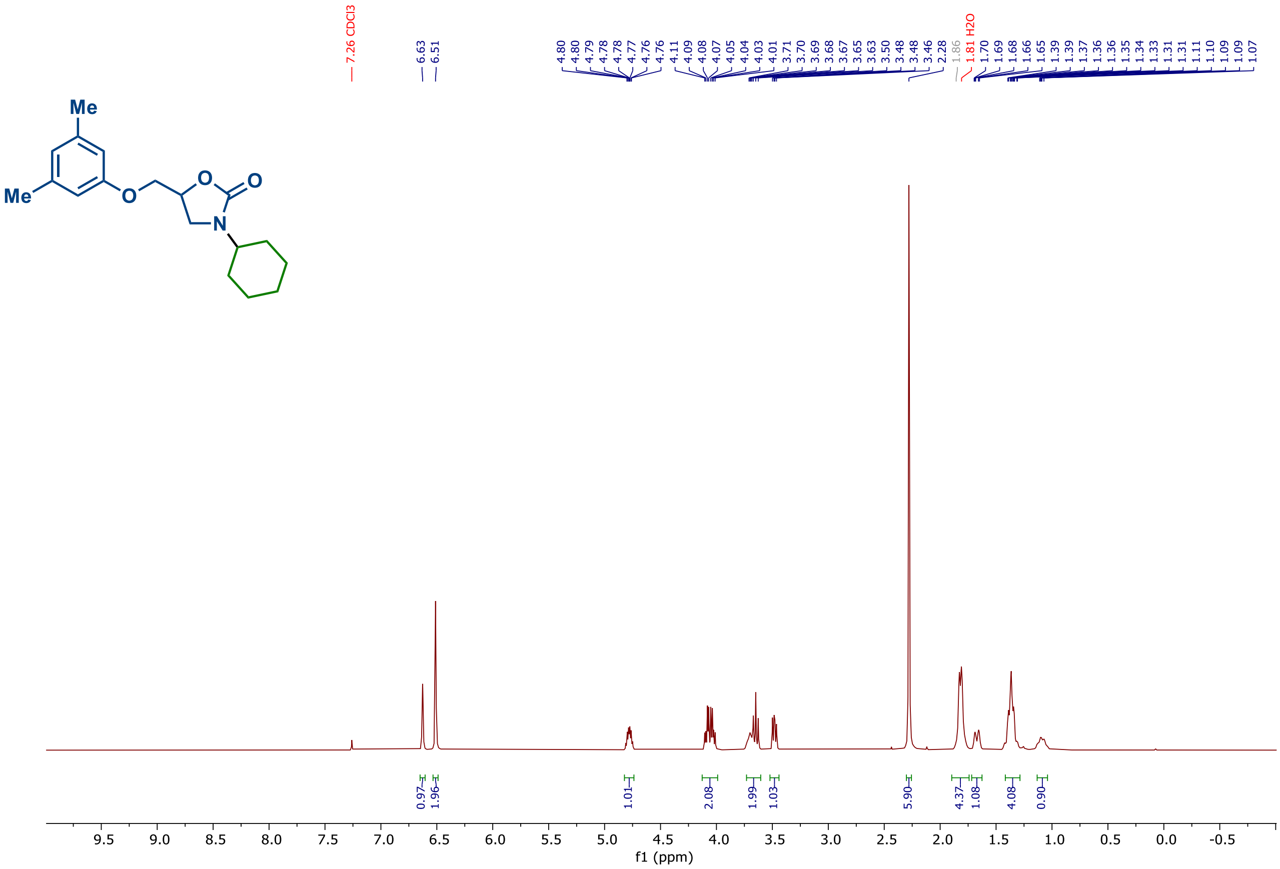

^{13}C NMR (101 MHz, $CDCl_3$) of **78**

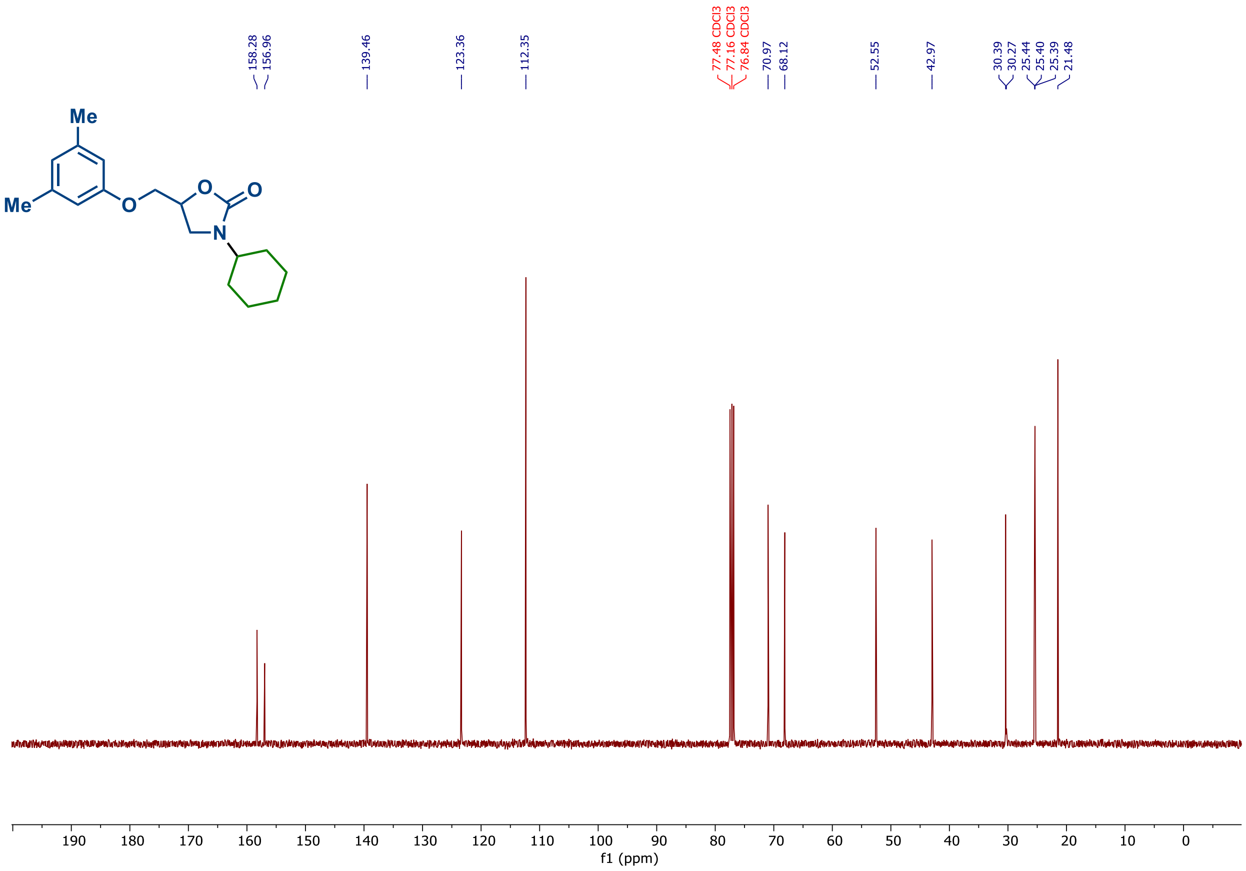

^{1}H NMR (400 MHz, $CDCl_3$) of **79** (*see procedure*)

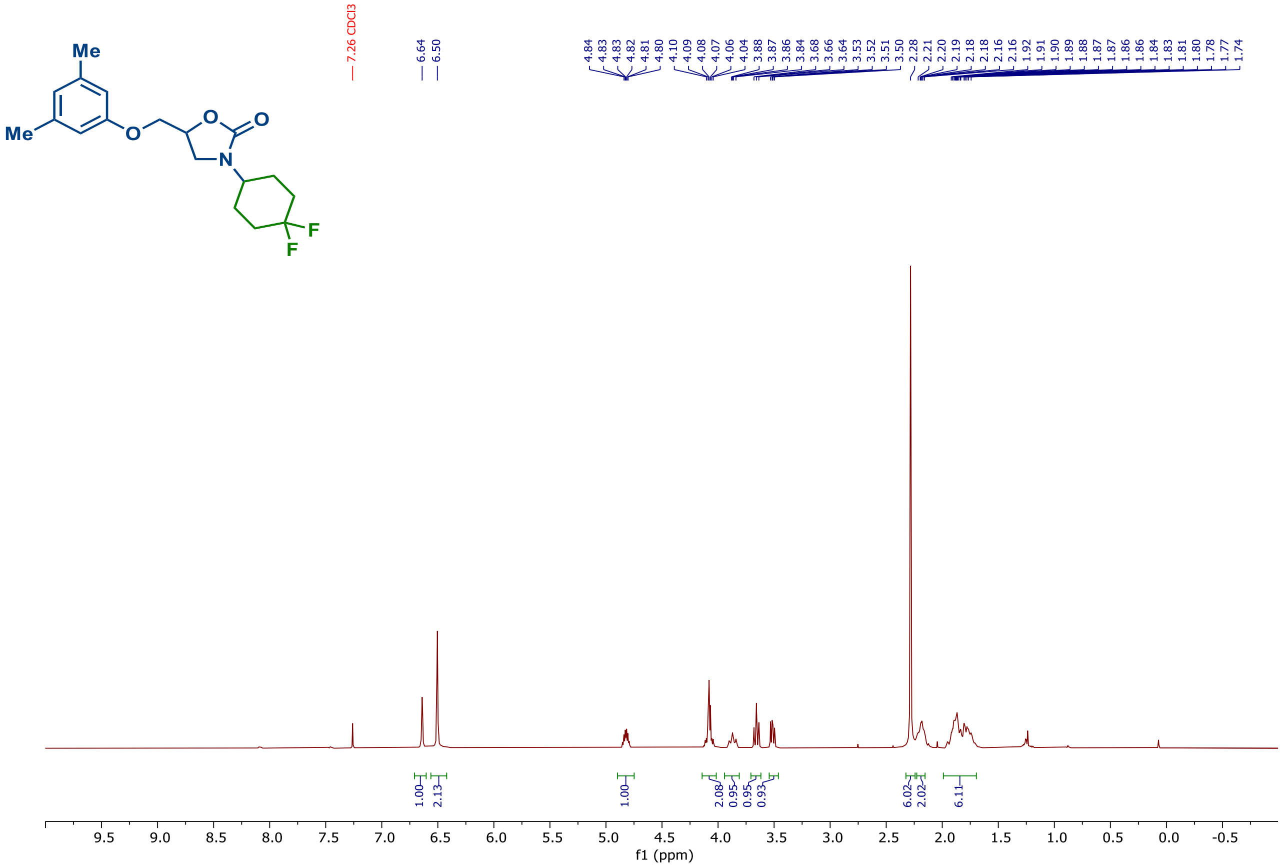

^{13}C NMR (101 MHz, $CDCl_3$) of **79**

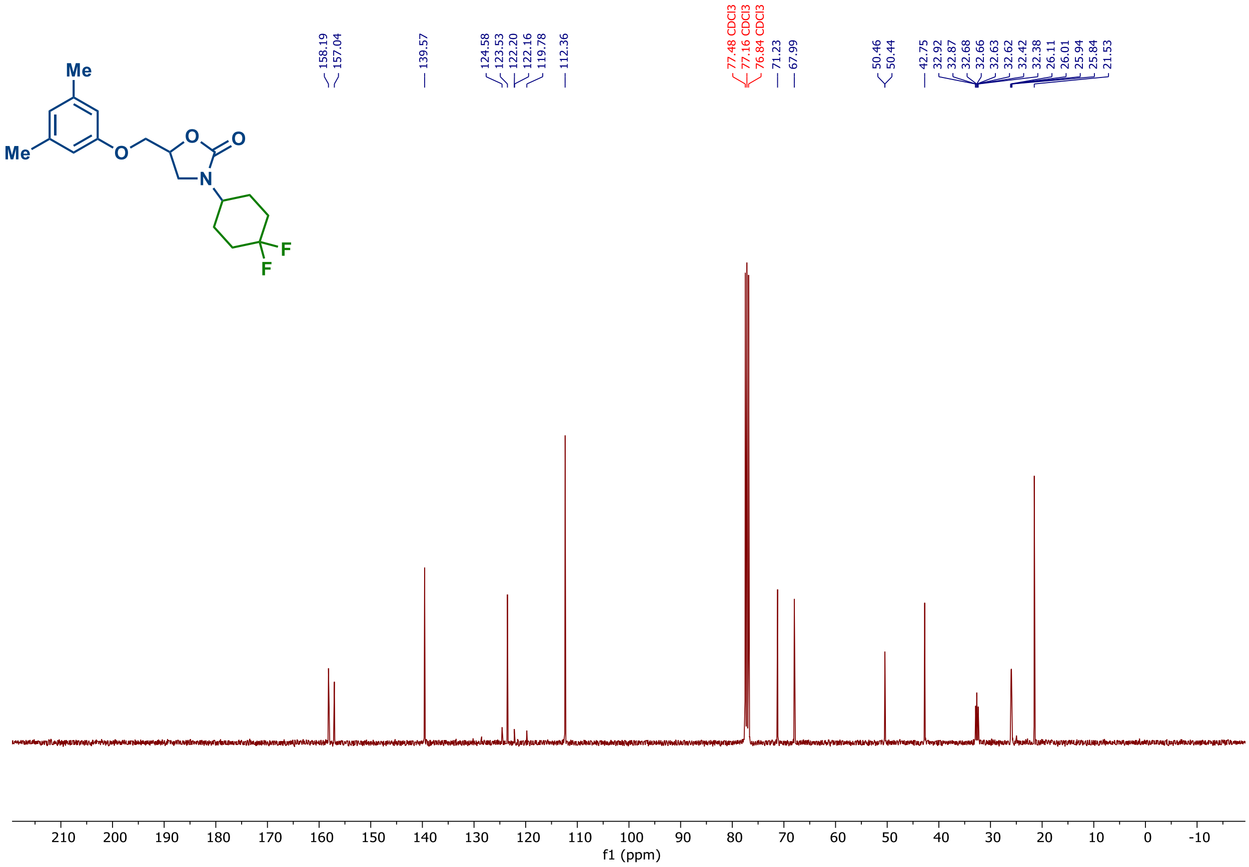

^{19}F NMR (376 MHz, $CDCl_3$) of **79**

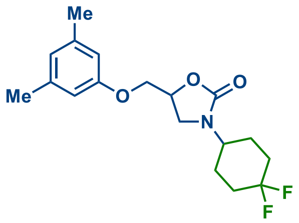

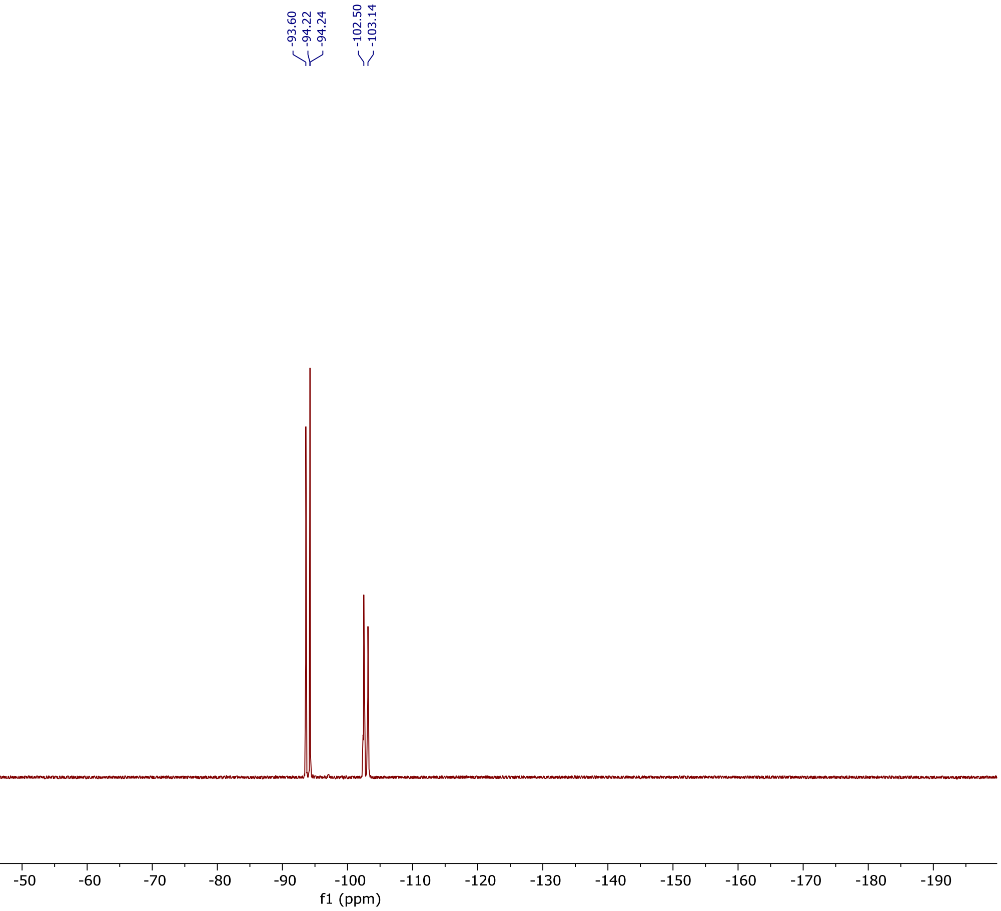

^{1}H NMR (400 MHz, $CDCl_3$) of **80** (see procedure)

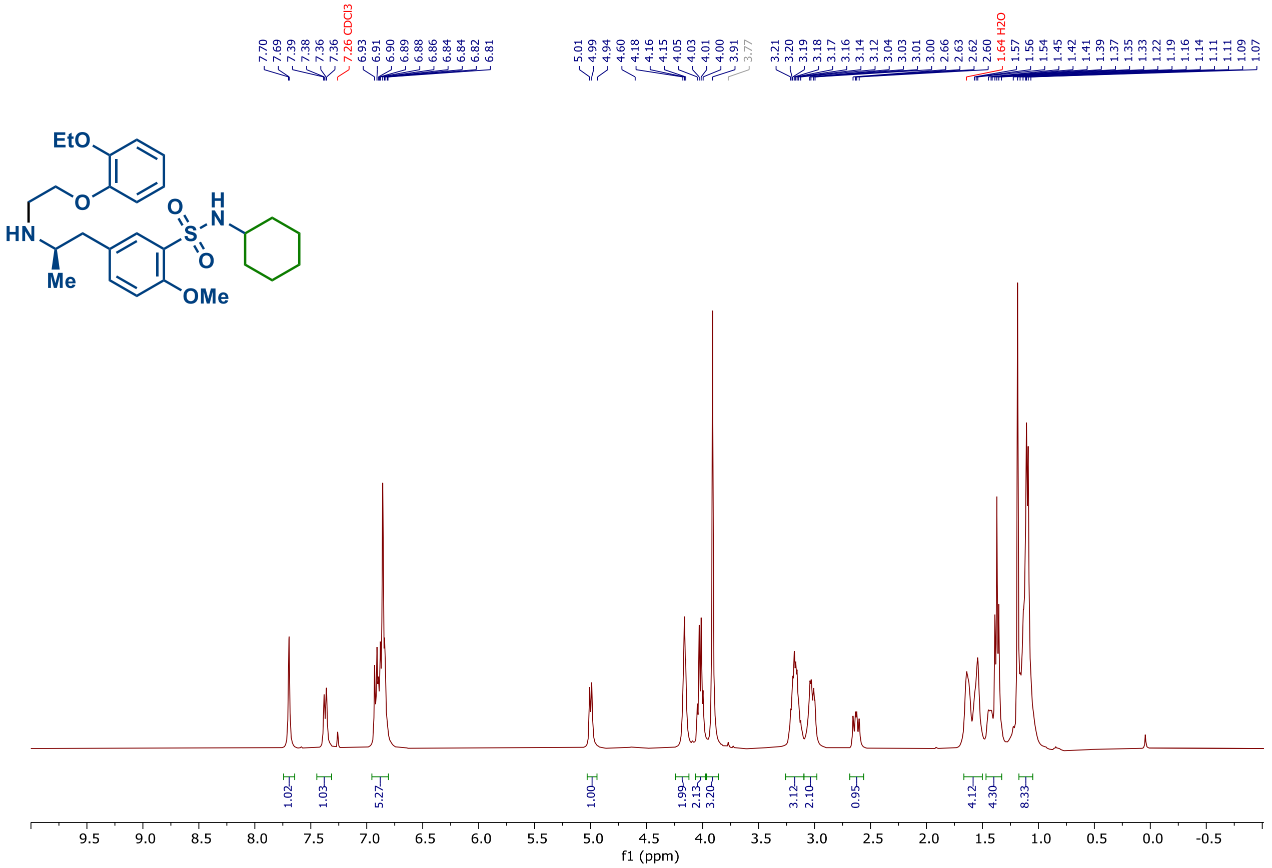

^{13}C NMR (101 MHz, $CDCl_3$) of **80**

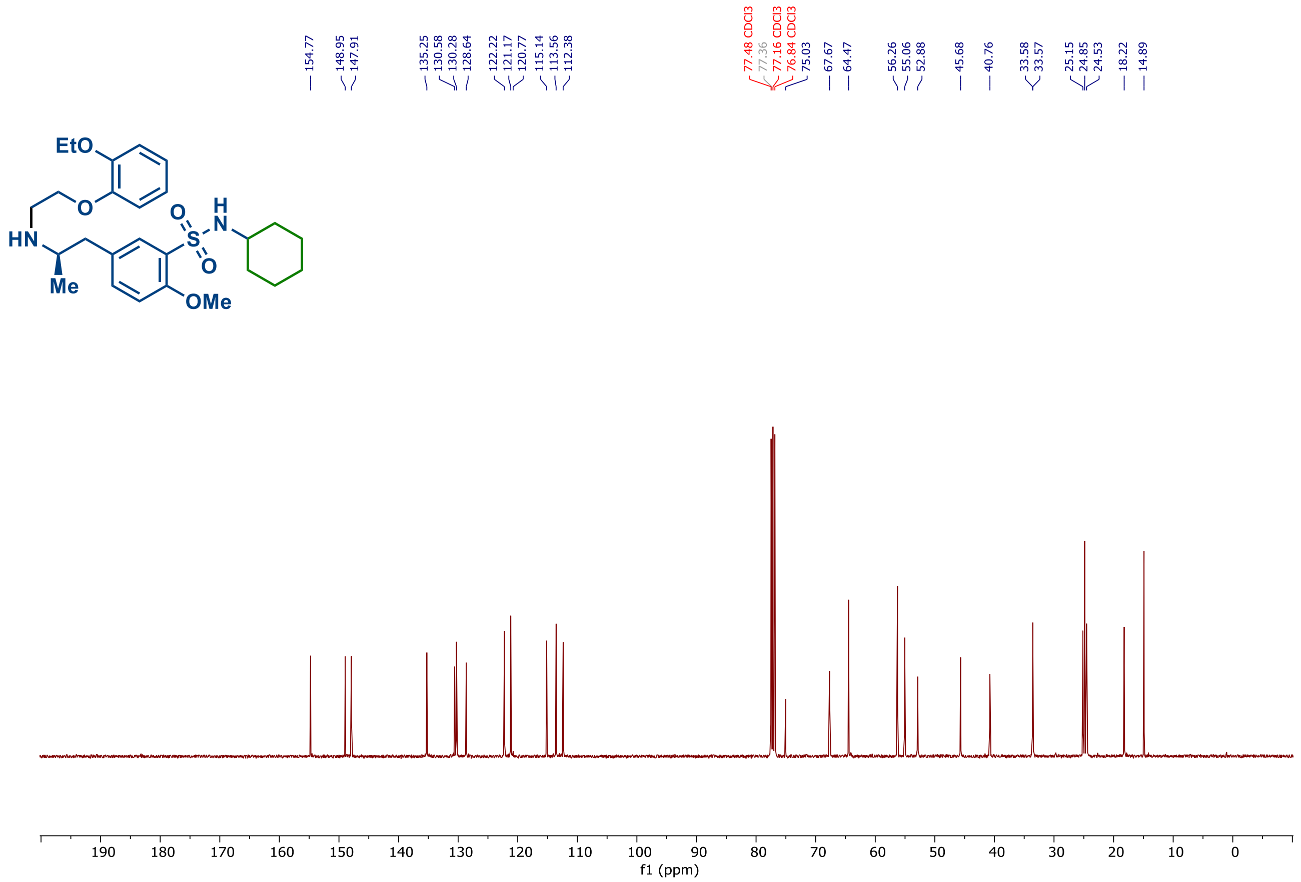

^{1}H NMR (400 MHz, $CDCl_3$) of **81** (*see procedure*)

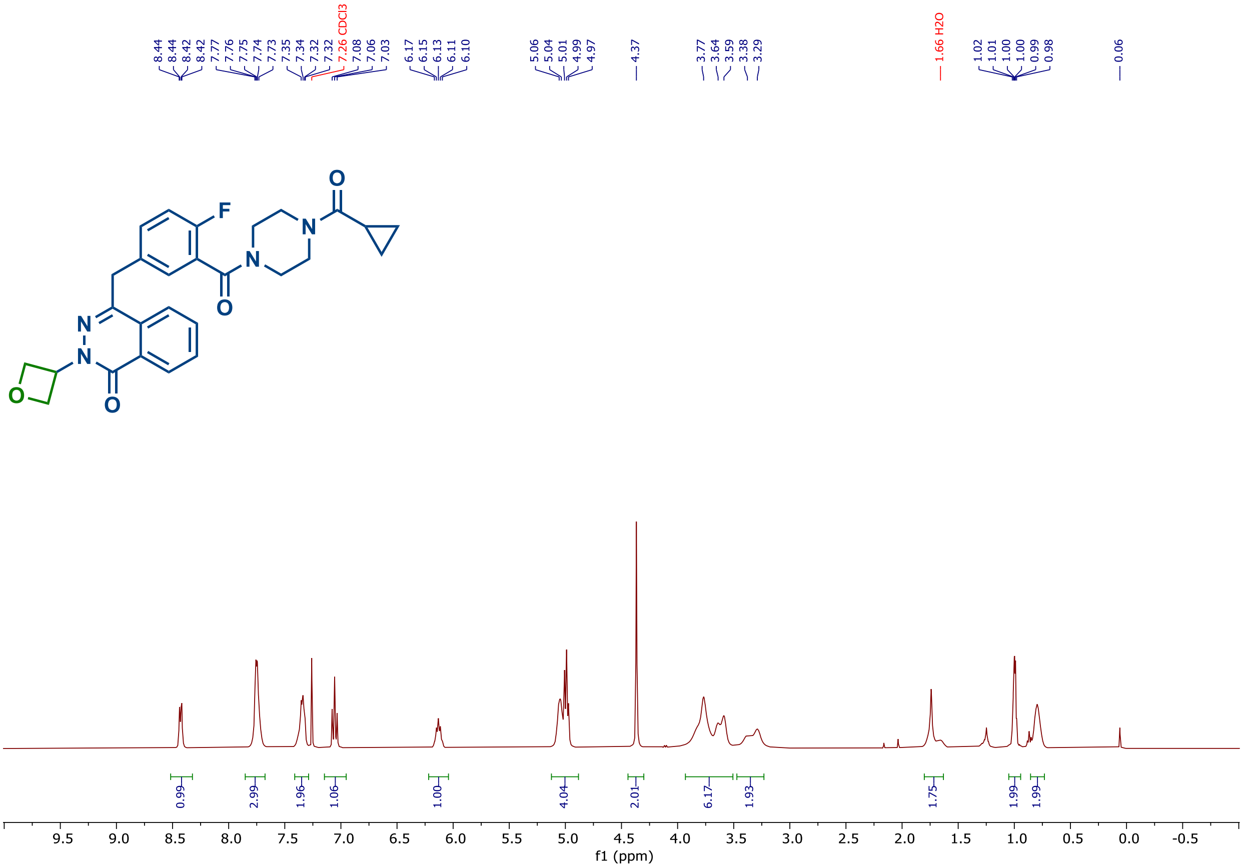

^{13}C NMR (101 MHz, $CDCl_3$) of **81**

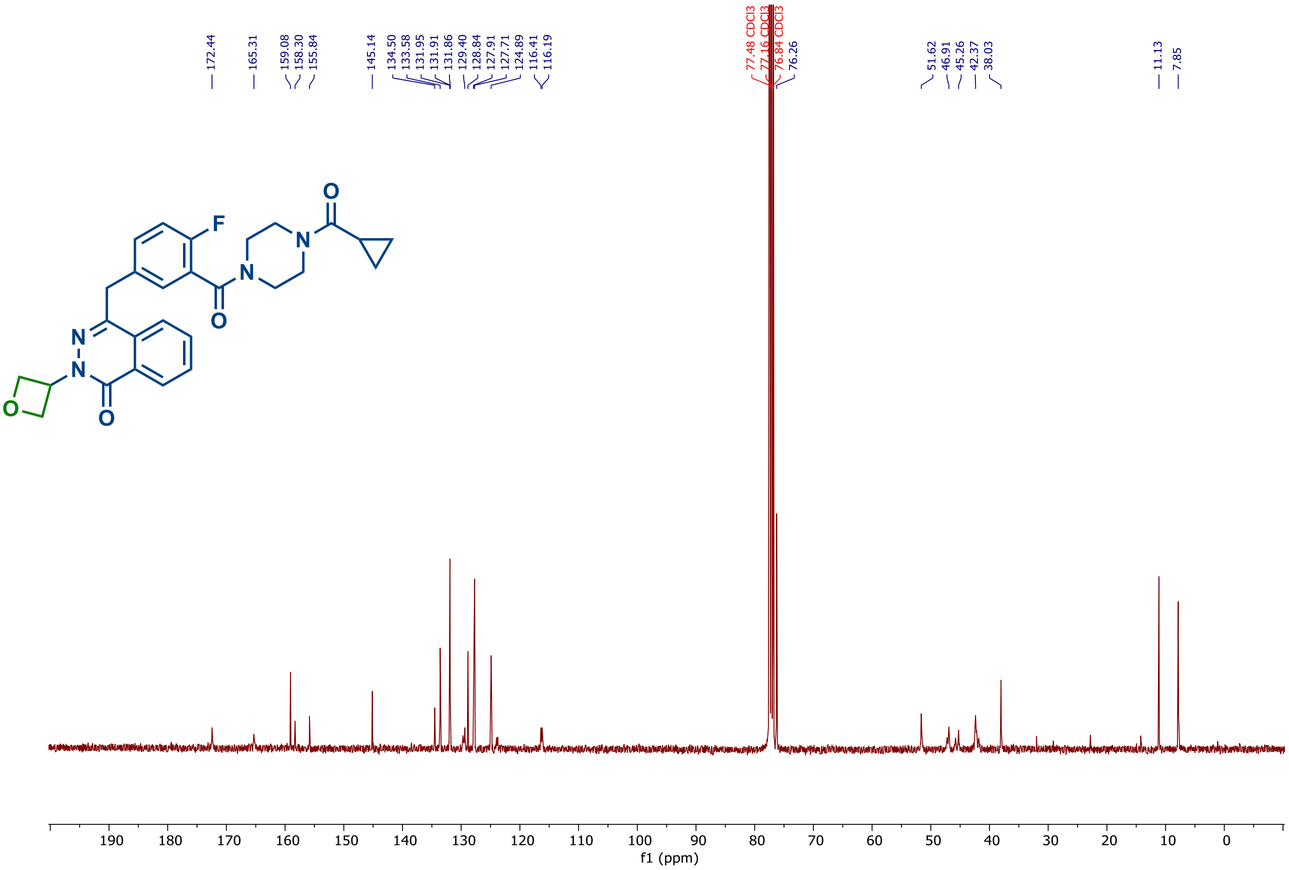

^{19}F NMR (376 MHz, $CDCl_3$) of **81**

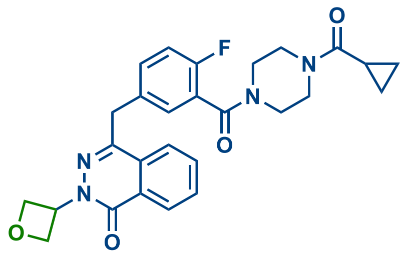

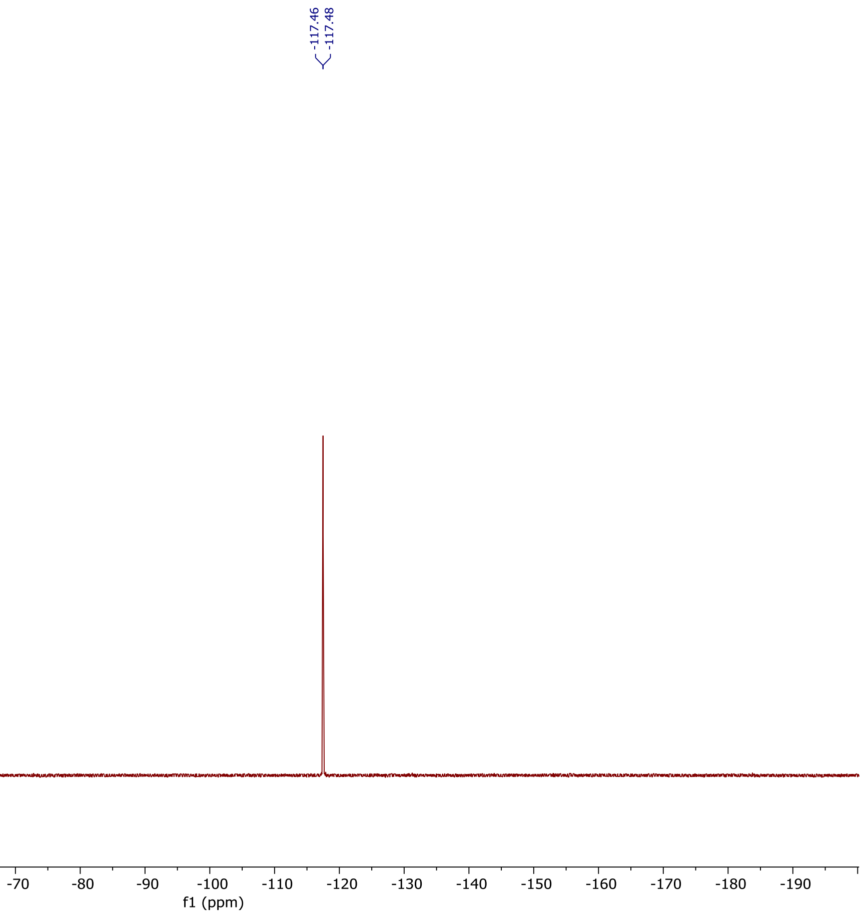

^{1}H NMR (400 MHz, CD_3OD) of **82** (see procedure)

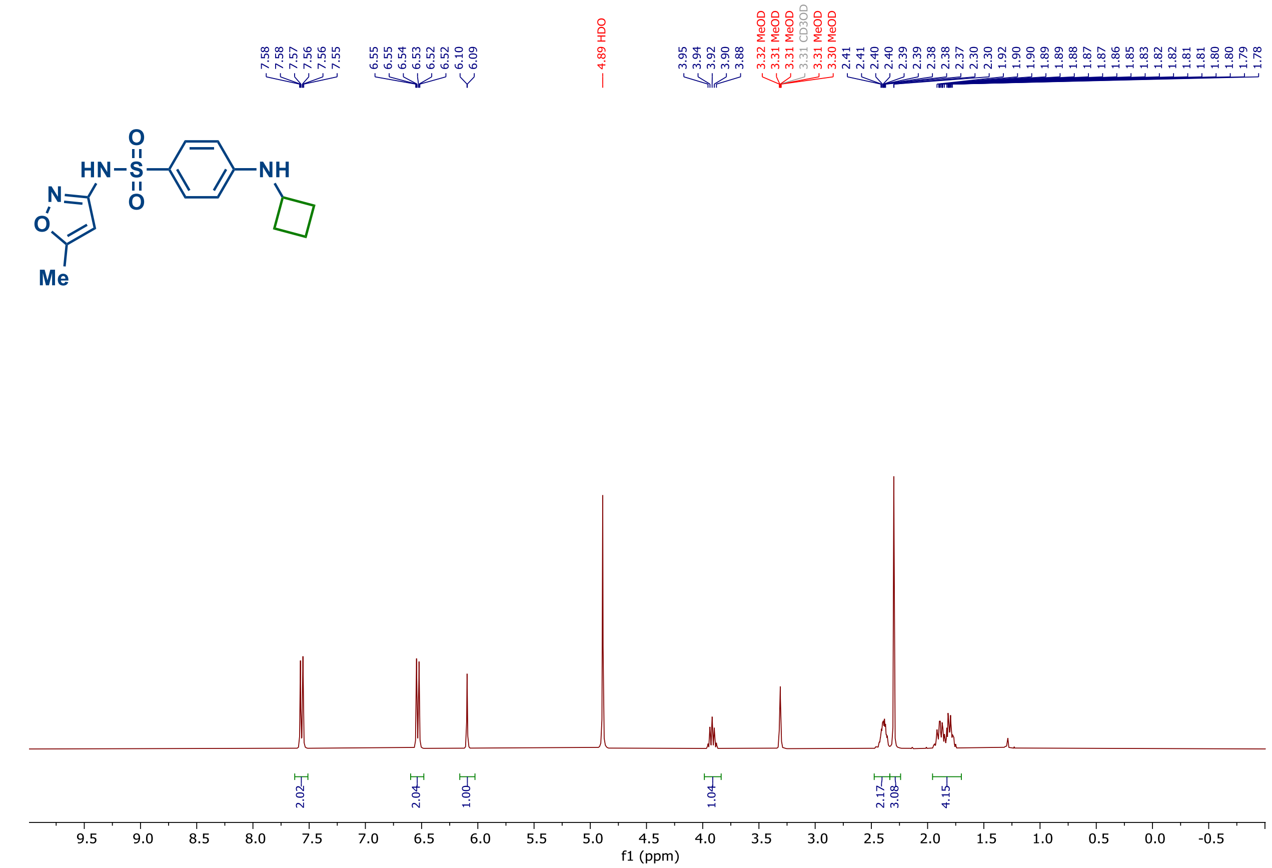

^{13}C NMR (101 MHz, CD_3OD) of **82**

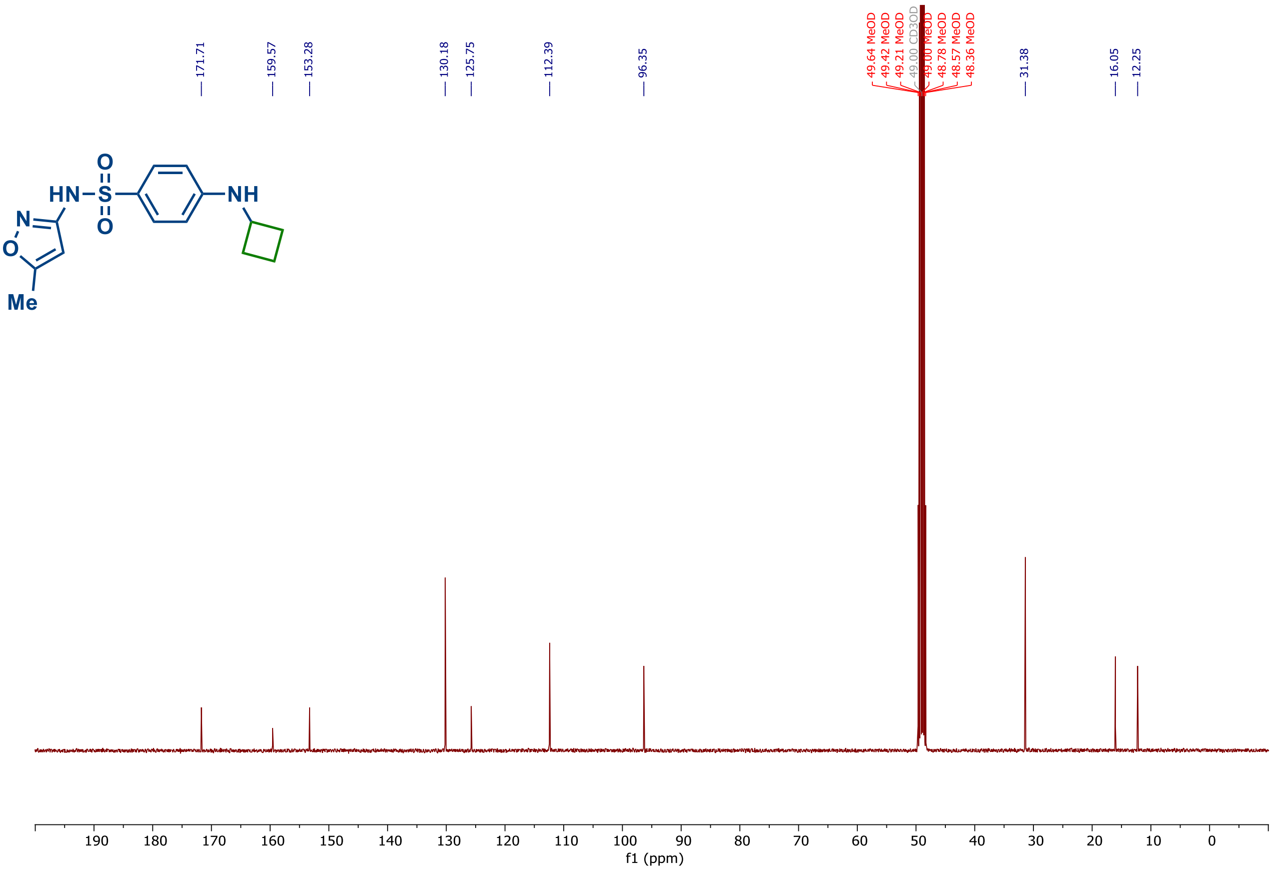

^{1}H NMR (400 MHz, $CDCl_3$) of **83** (*see procedure*)

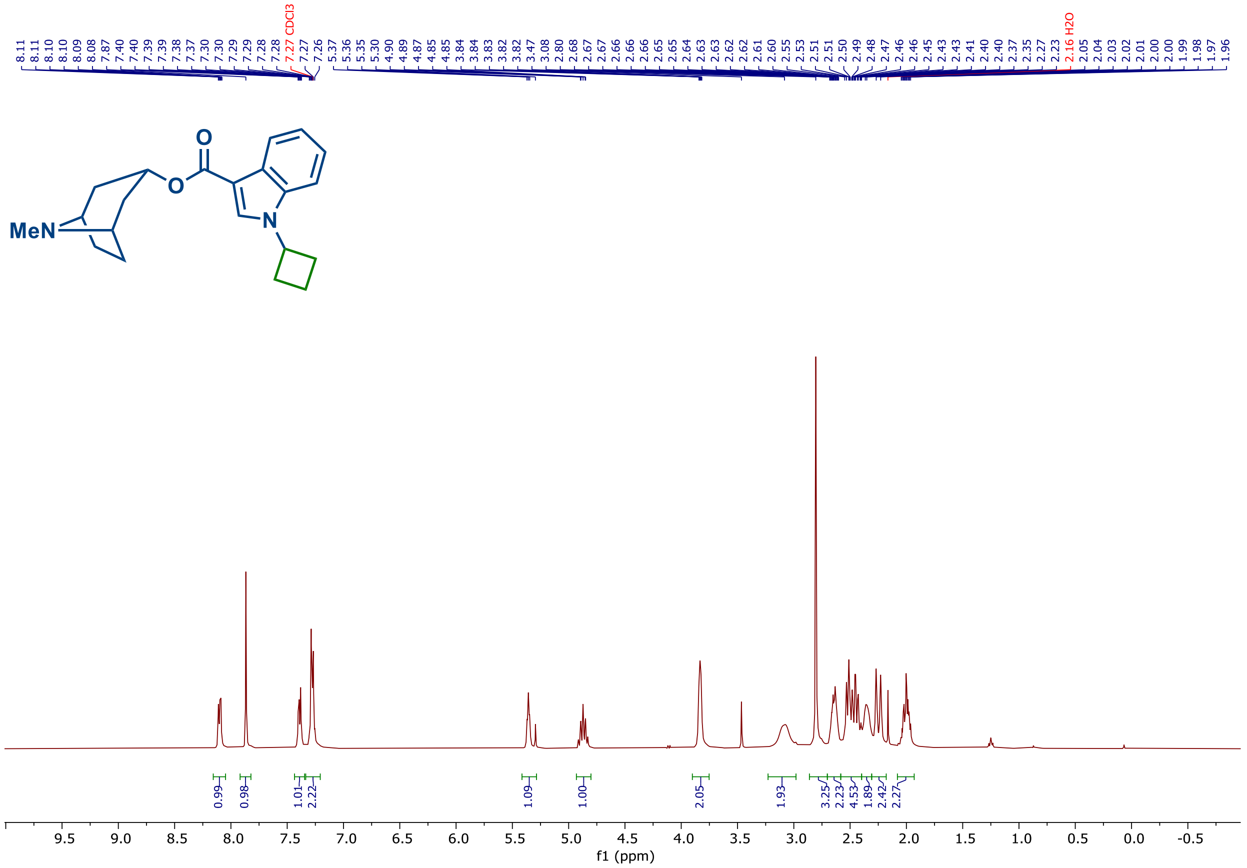

^{13}C NMR (101 MHz, $CDCl_3$) of **83**

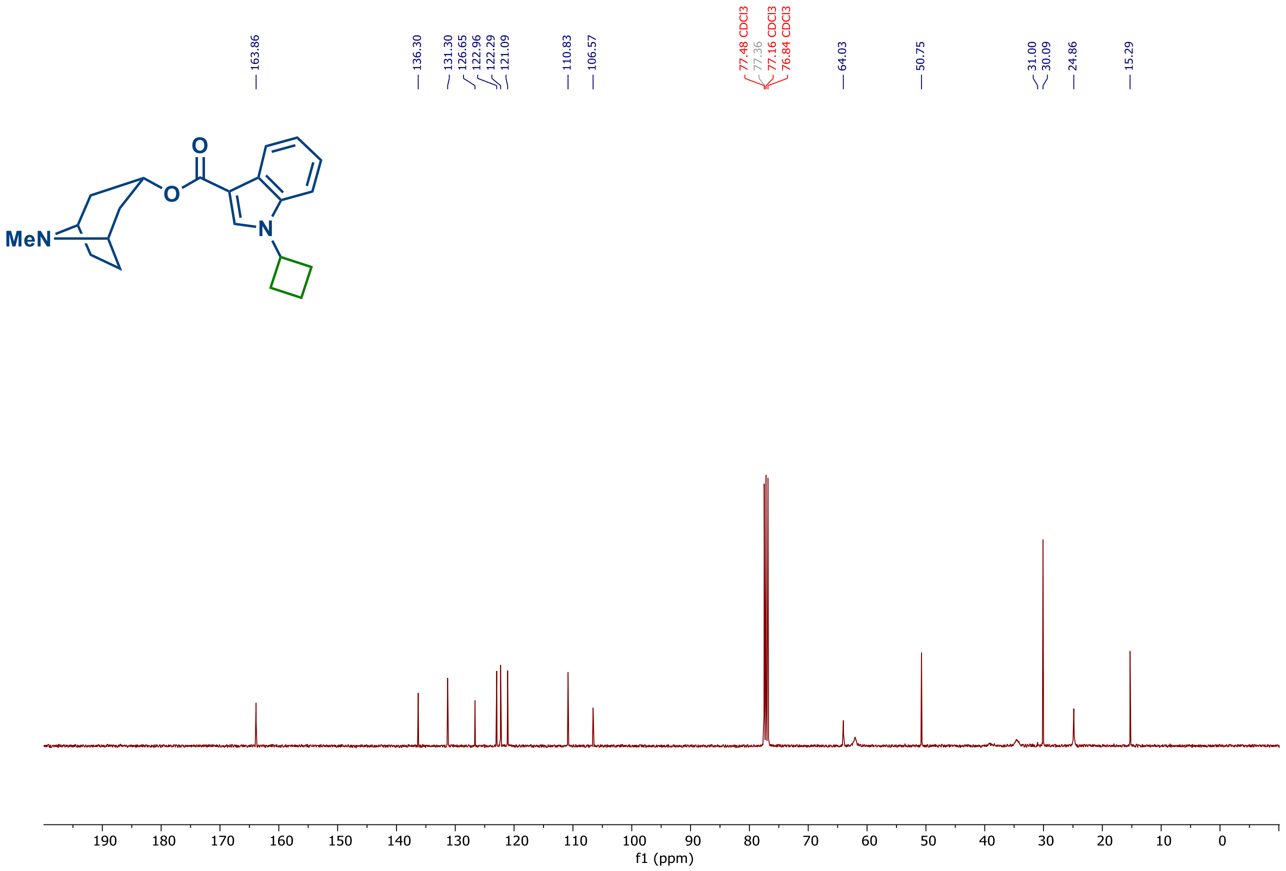

^{1}H NMR (400 MHz, $CDCl_3$) of **84** (*see procedure*)

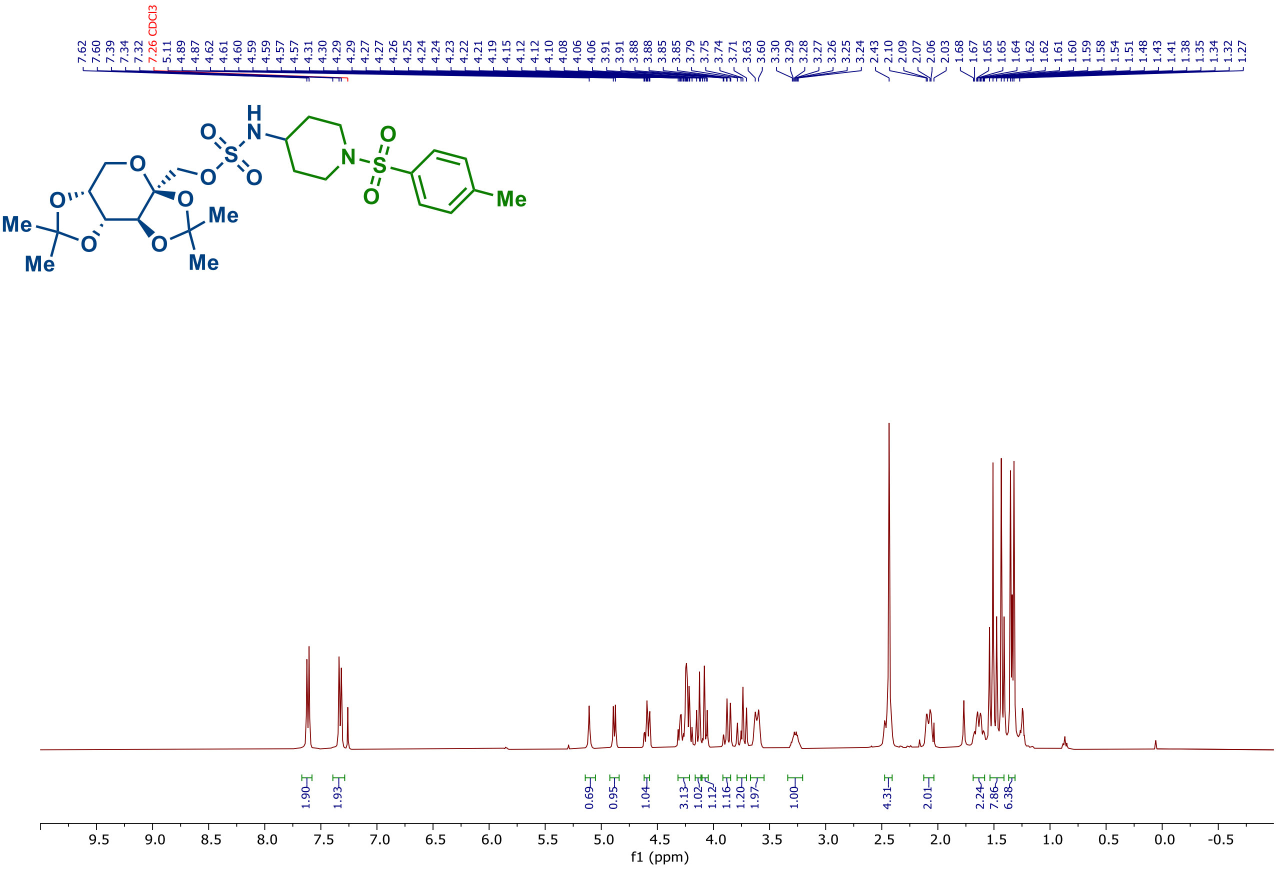

^{13}C NMR (101 MHz, $CDCl_3$) of **84**

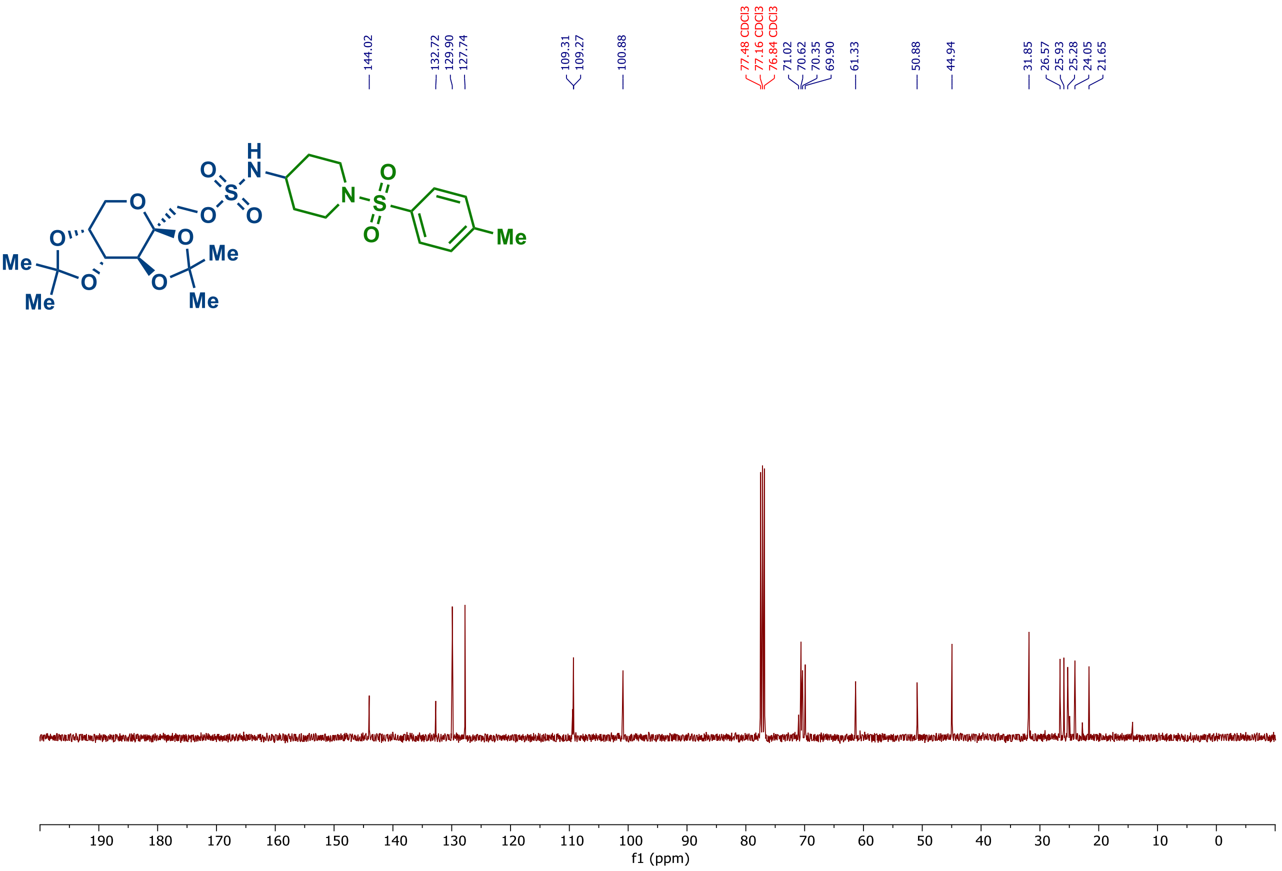

^{1}H NMR (400 MHz, $CDCl_3$) of **85** (*see procedure*)

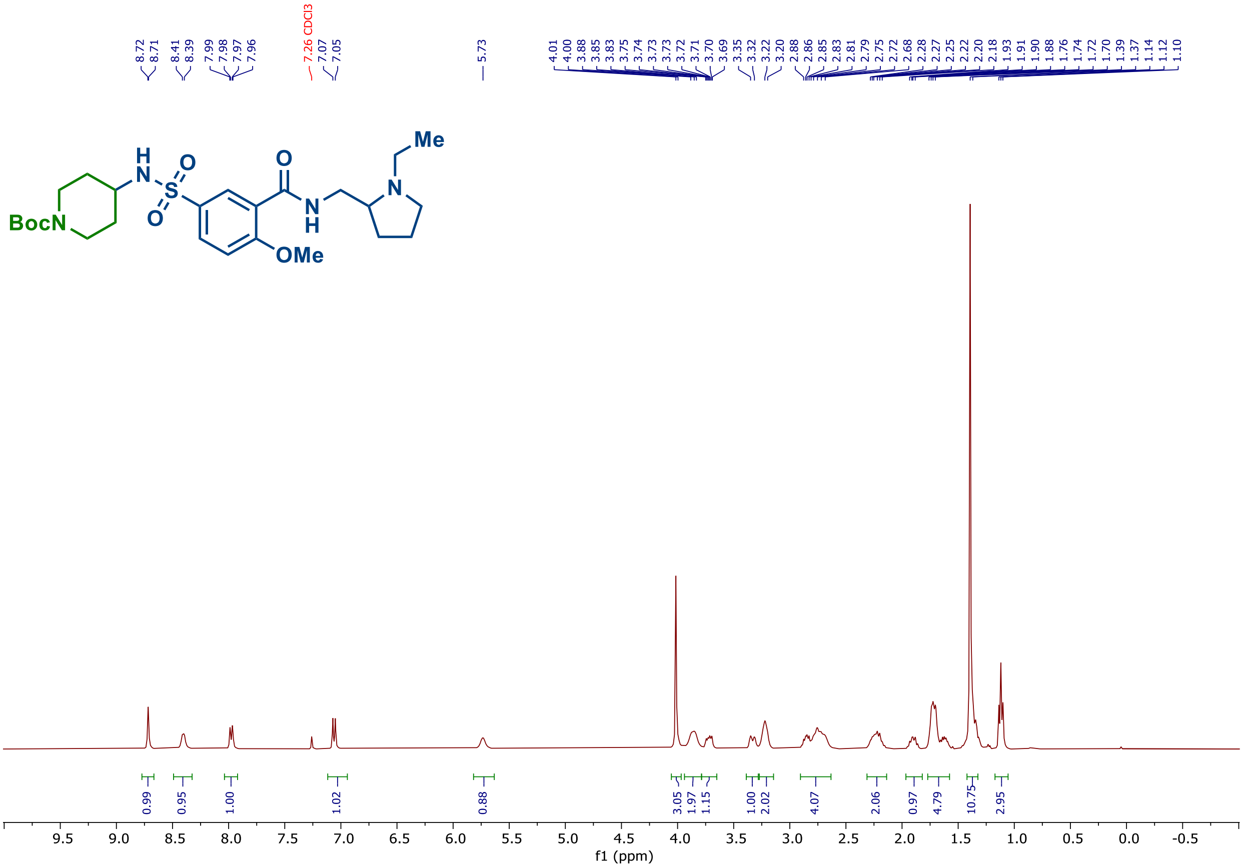

^{13}C NMR (101 MHz, $CDCl_3$) of **85**

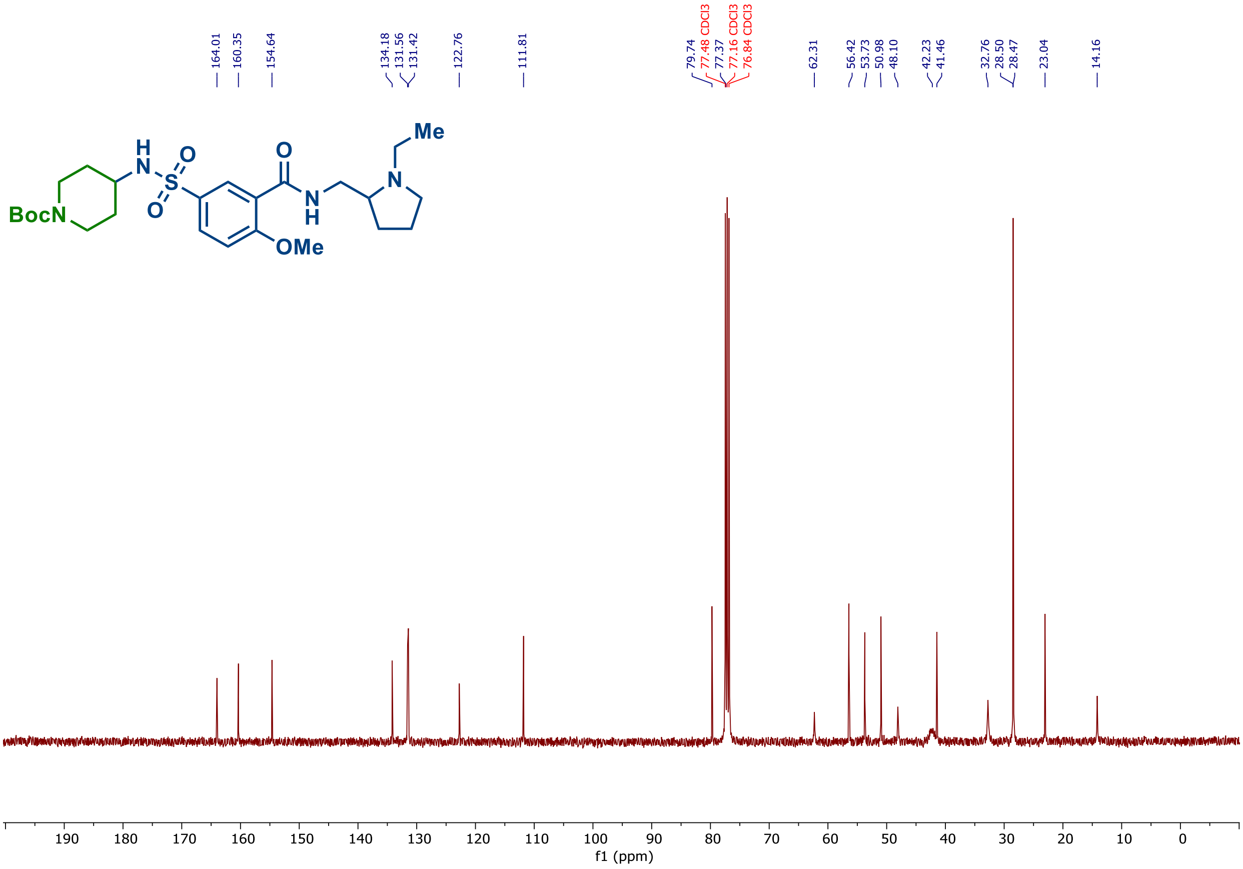

^{1}H NMR (400 MHz, $CDCl_3$) of **86** (*see procedure*)

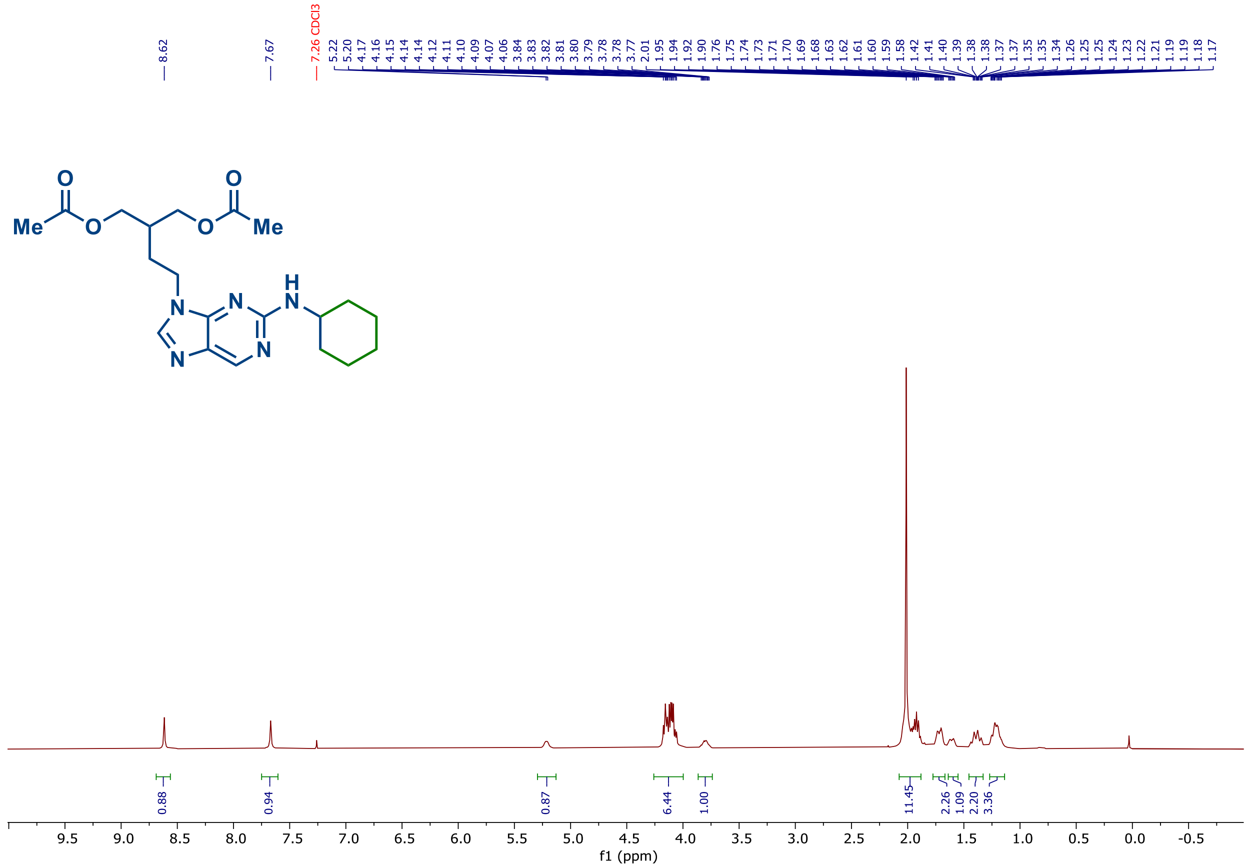

^{13}C NMR (101 MHz, $CDCl_3$) of **86**

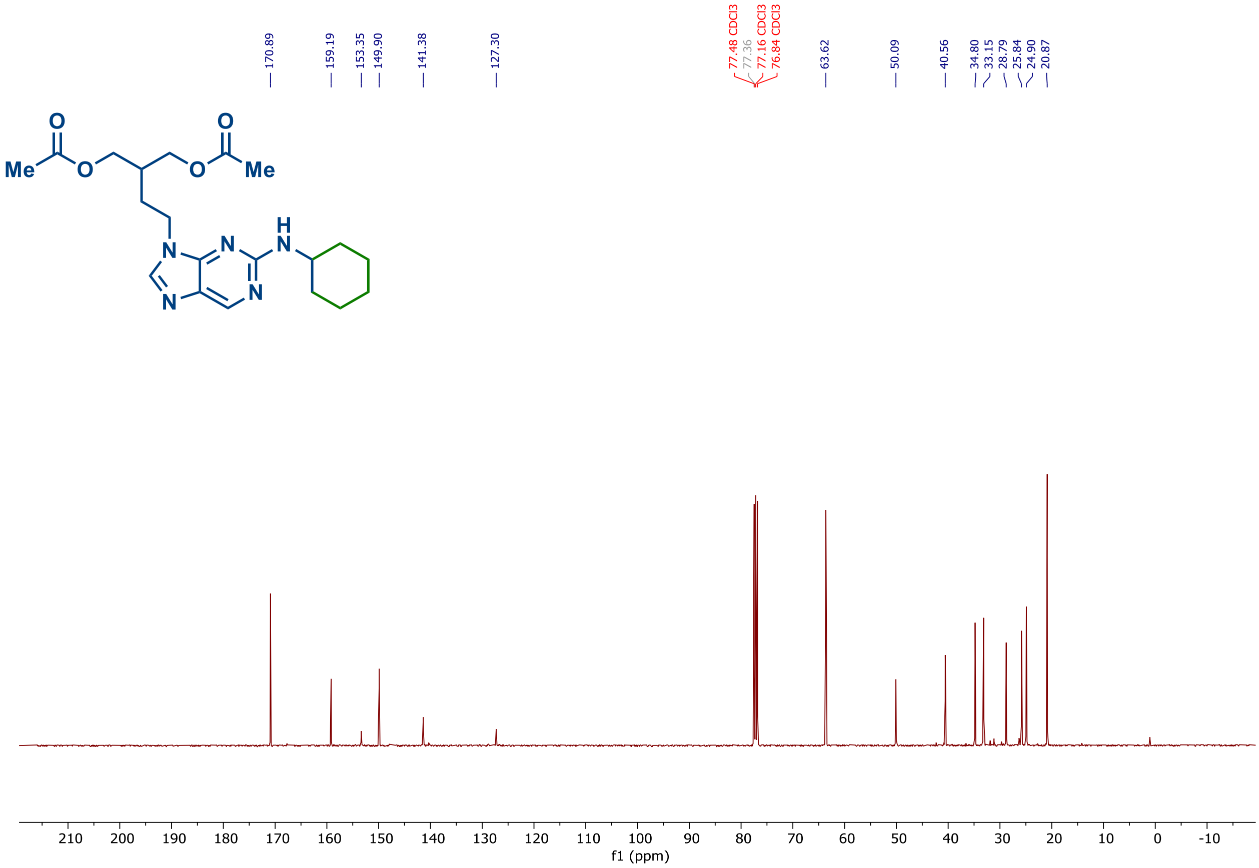

^{1}H NMR (400 MHz, CD_3CN) of **87** (*see procedure*)

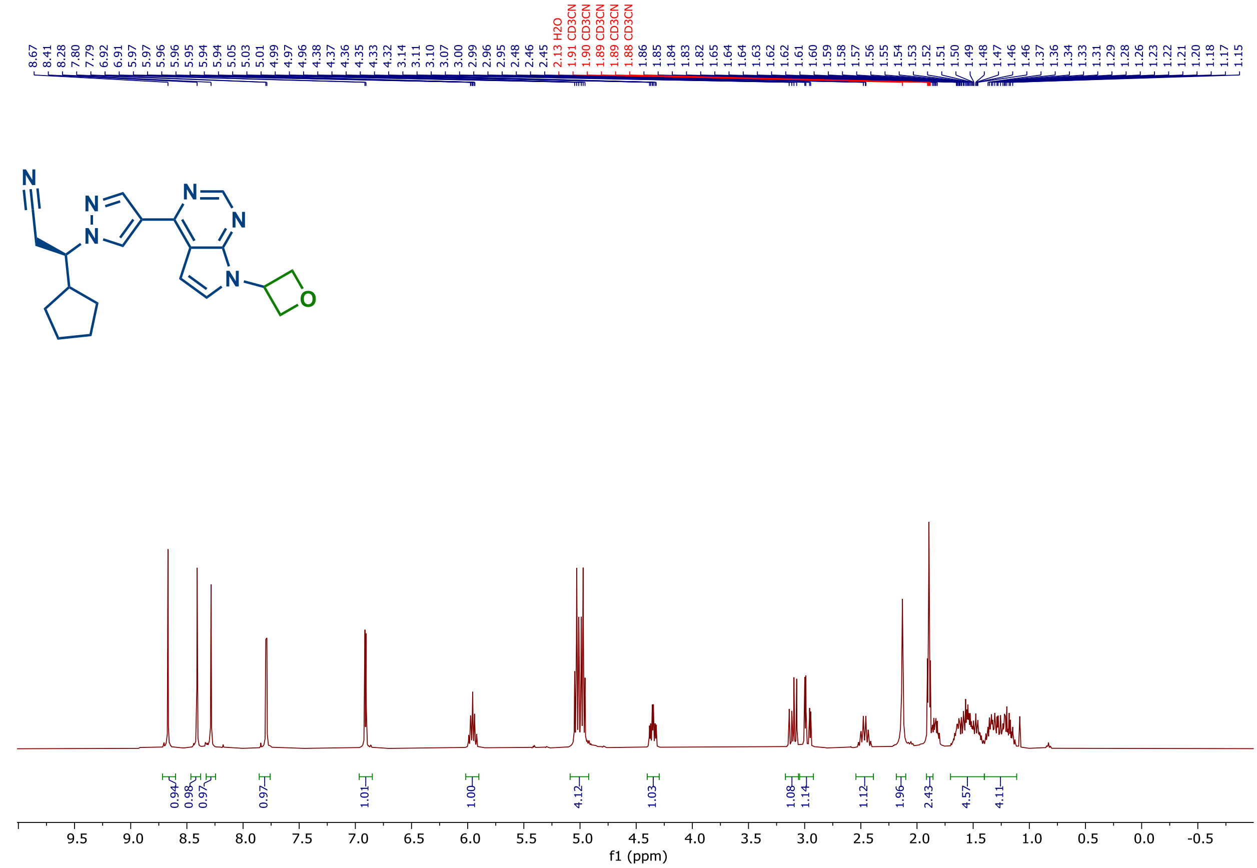

^{13}C NMR (101 MHz, CD_3CN) of **87**

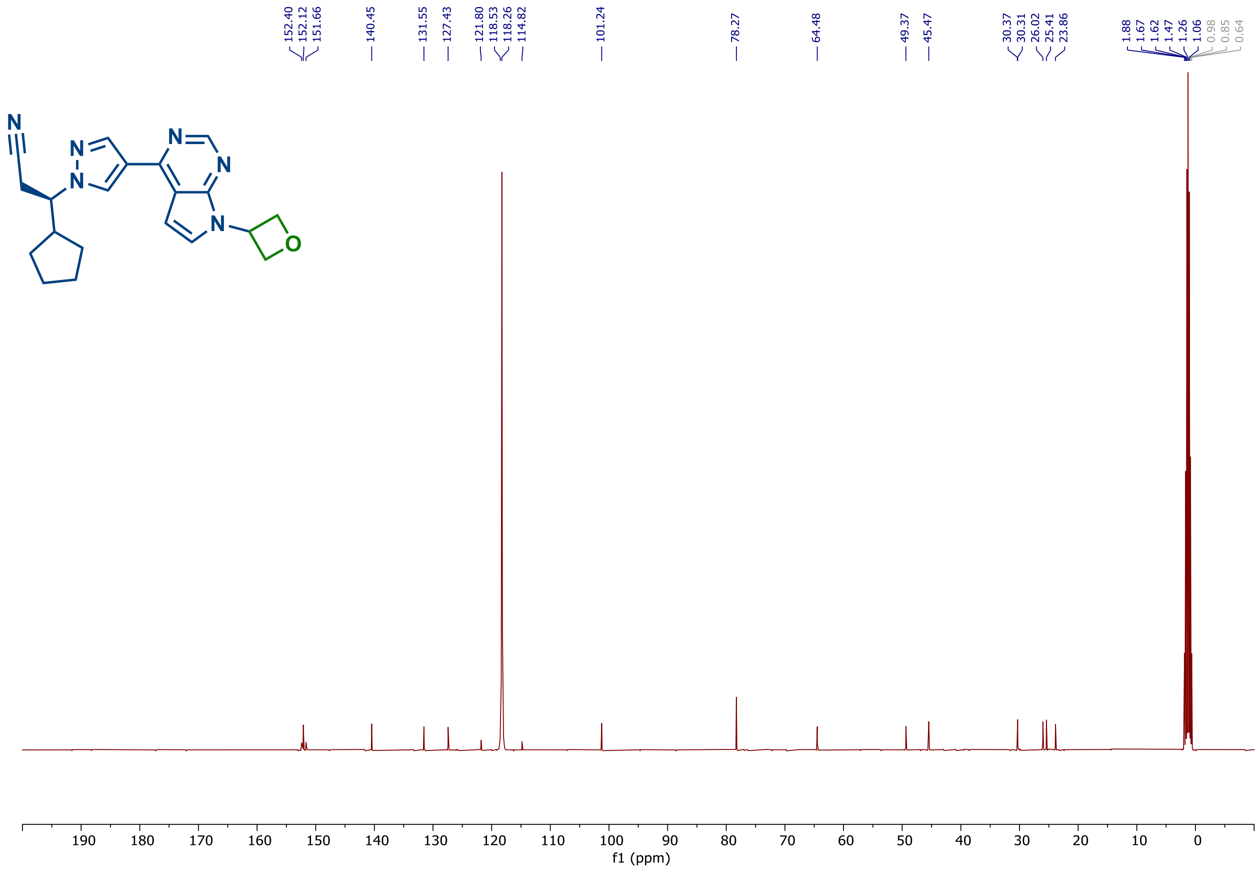

^{1}H NMR (400 MHz, CD_3CN) of **88** (*see procedure*)

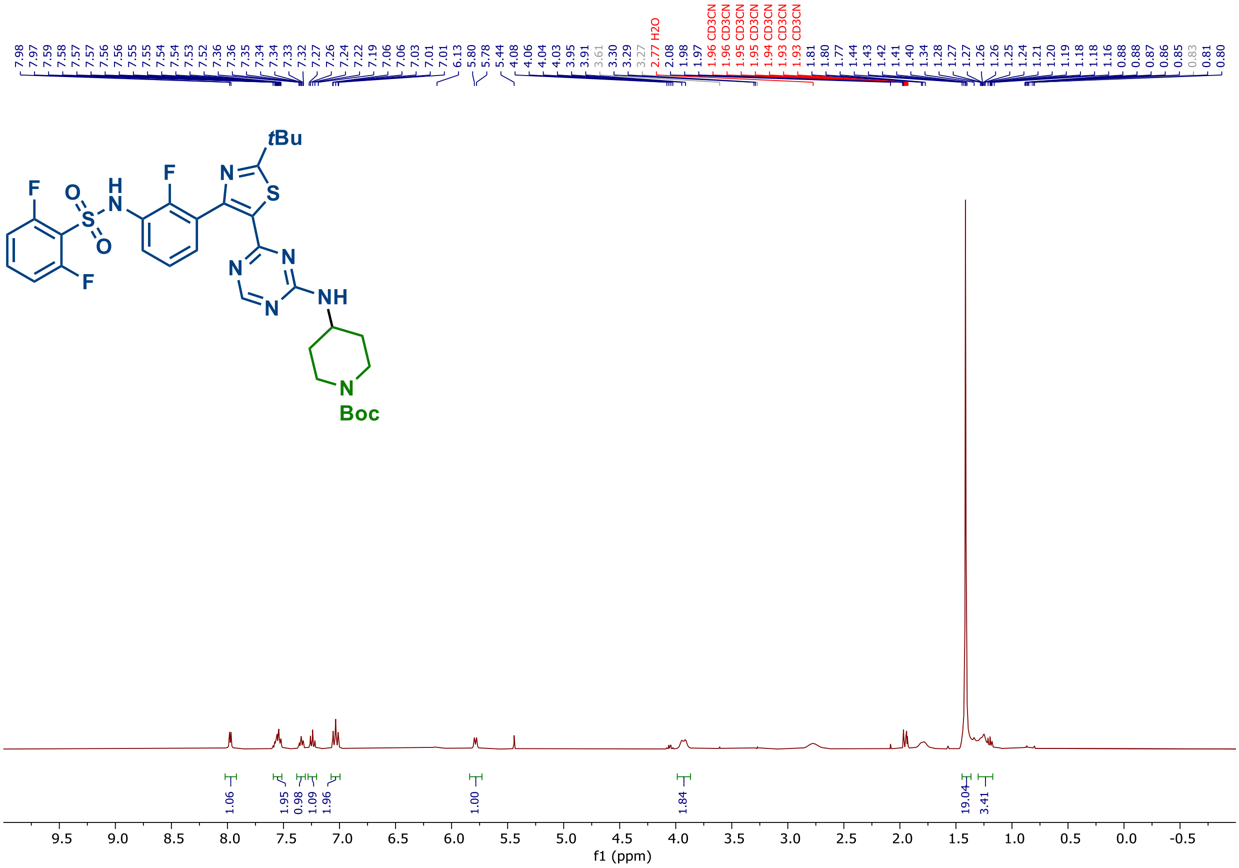

^{13}C NMR (101 MHz, CD_3CN) of **88**

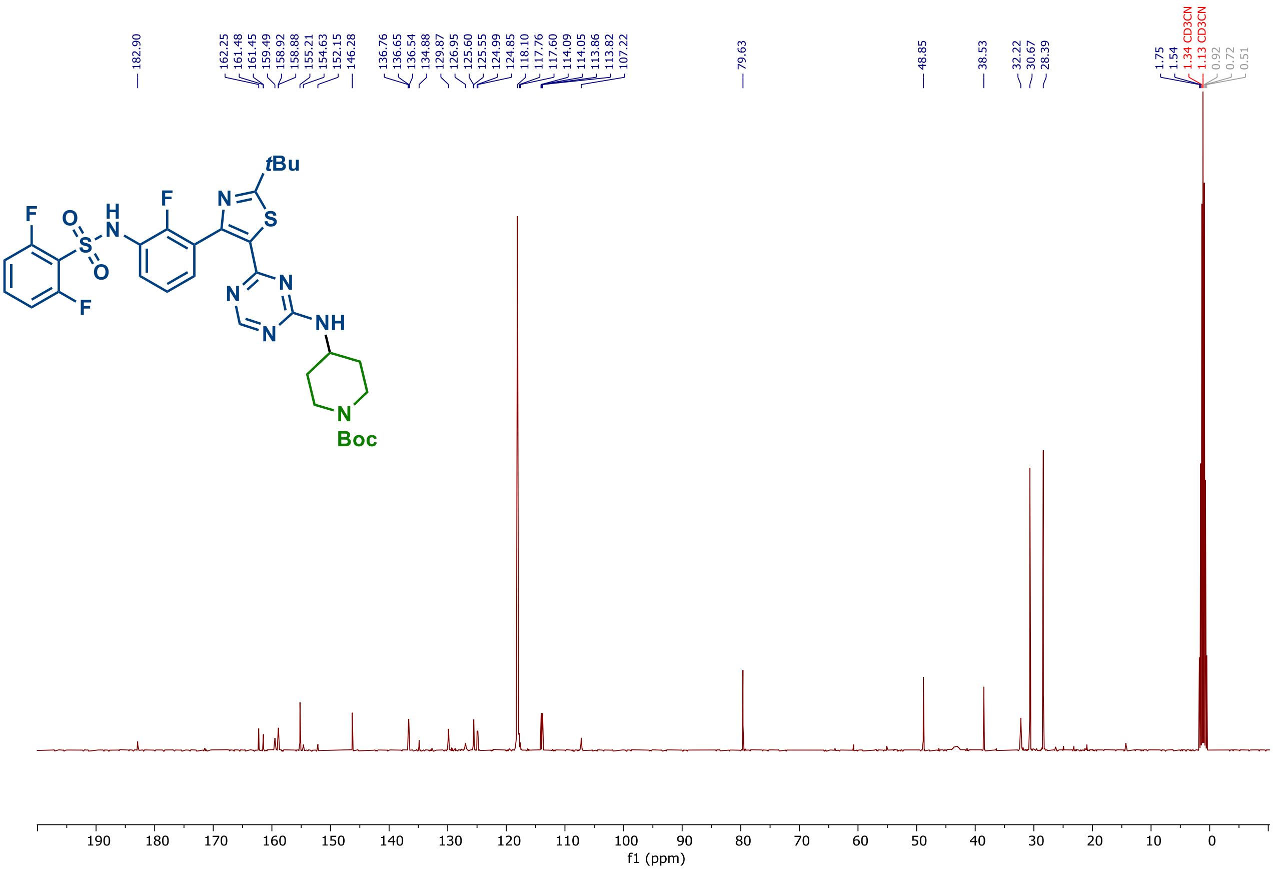

^{19}F NMR (376 MHz, CD_3CN) of **88**

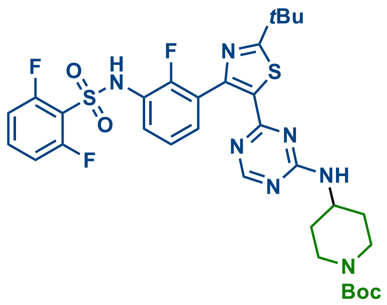

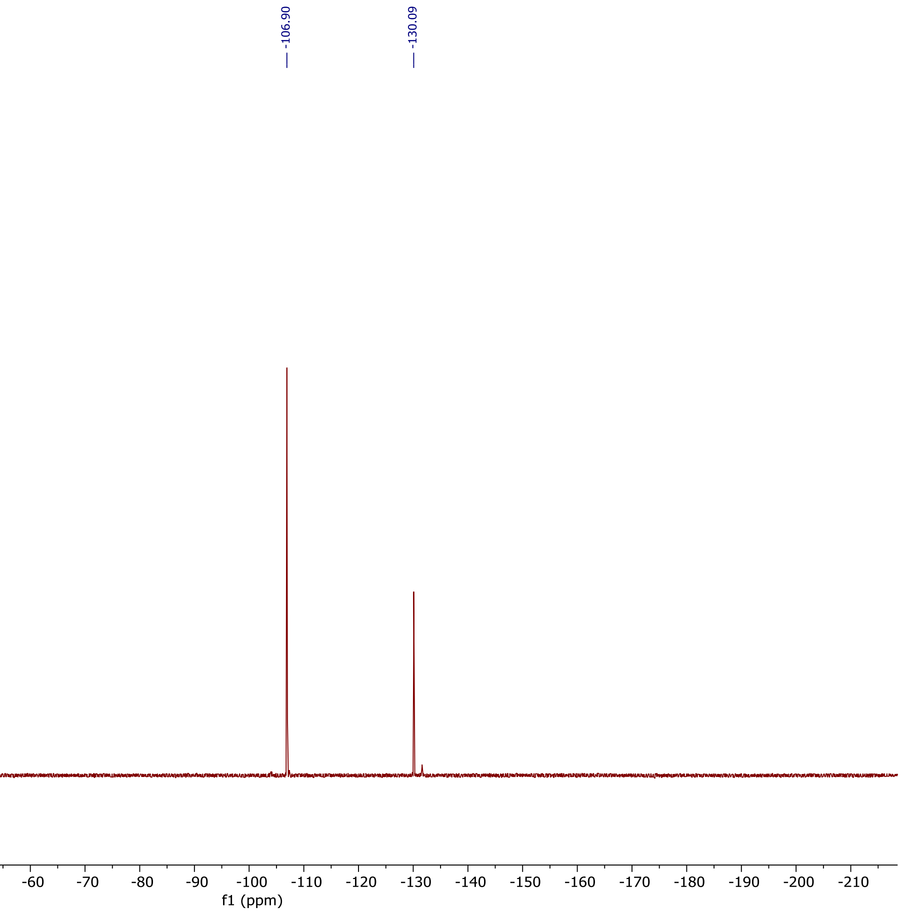

^{1}H NMR (400 MHz, d_6-DMSO) of **89** (*see procedure*)

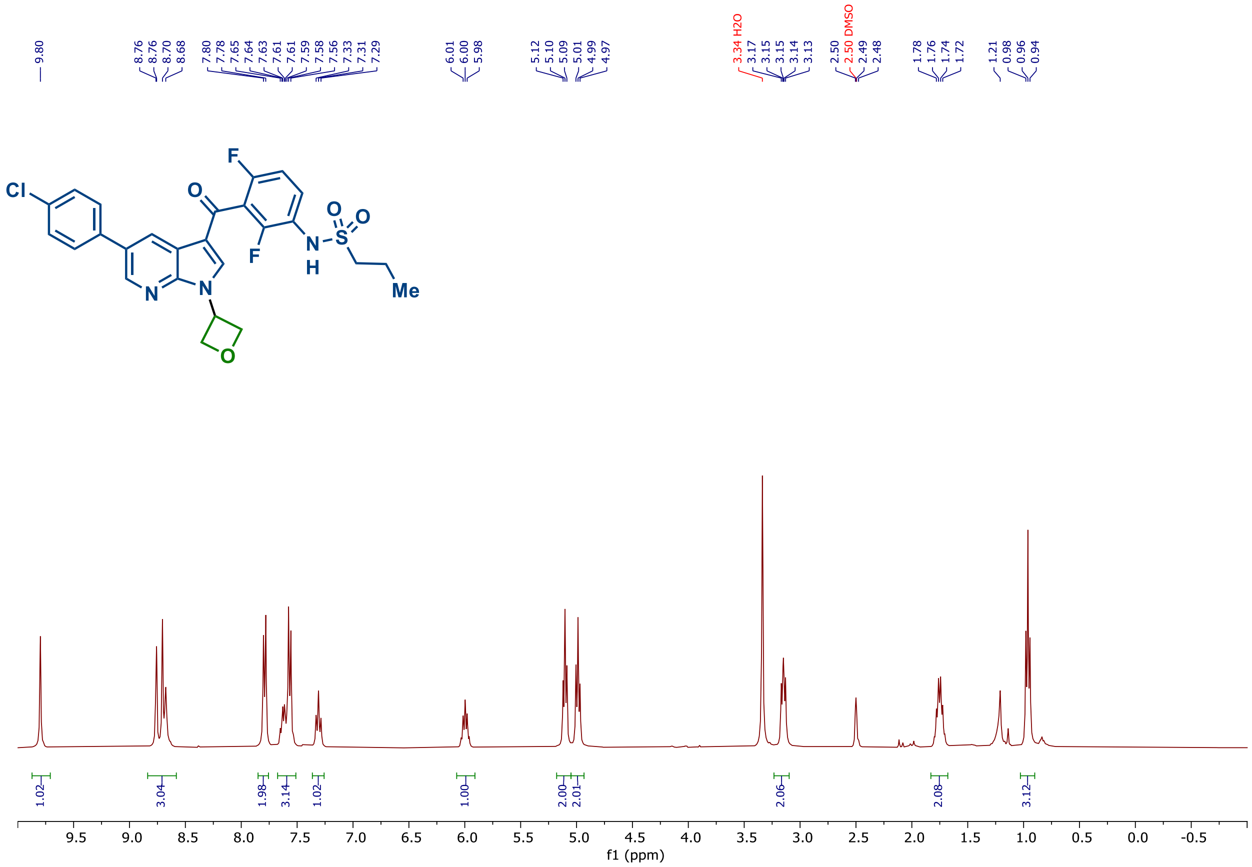

^{13}C NMR (101 MHz, d_6-DMSO) of **89**

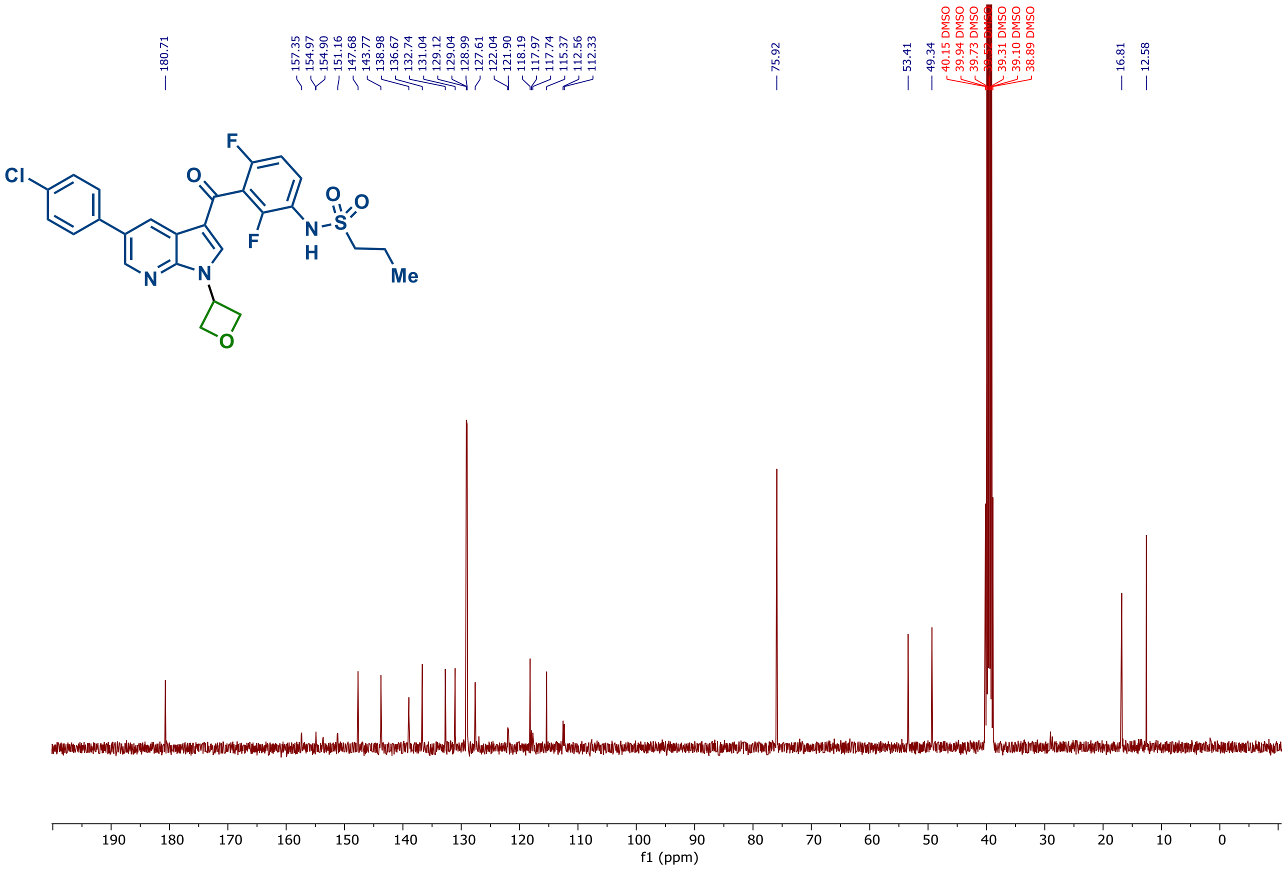

^{19}F NMR (376 MHz, d_6-DMSO) of **89**

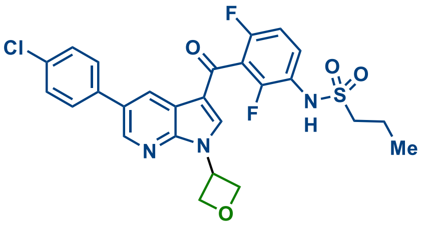

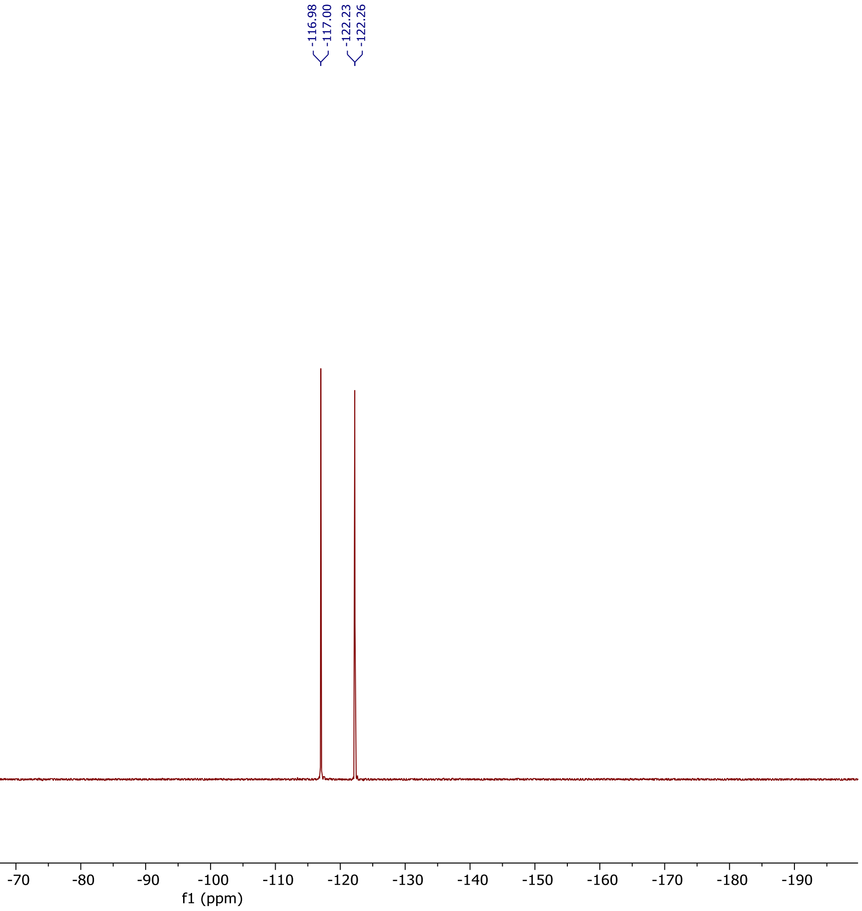

^{1}H NMR (400 MHz, $CDCl_3$) of **90** (*see procedure*)

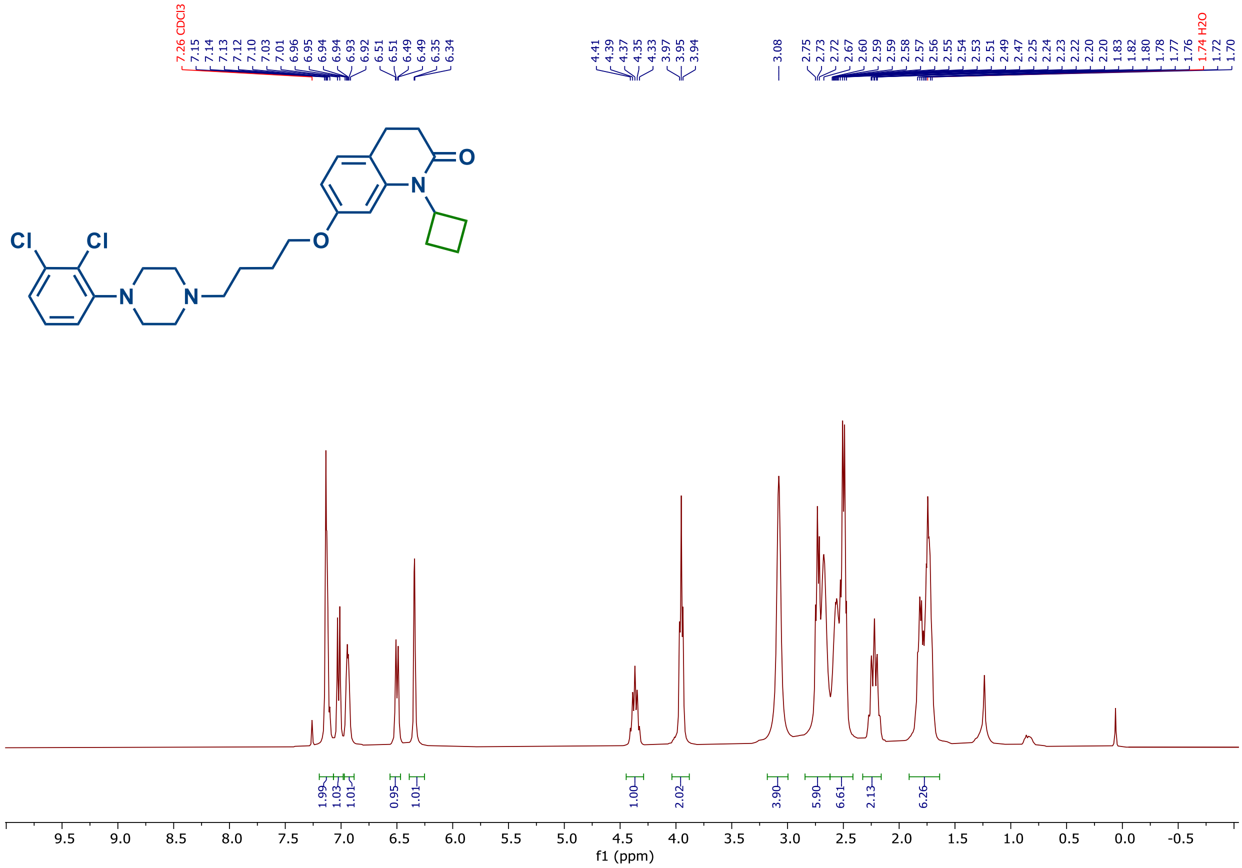

^{13}C NMR (101 MHz, $CDCl_3$) of **90**

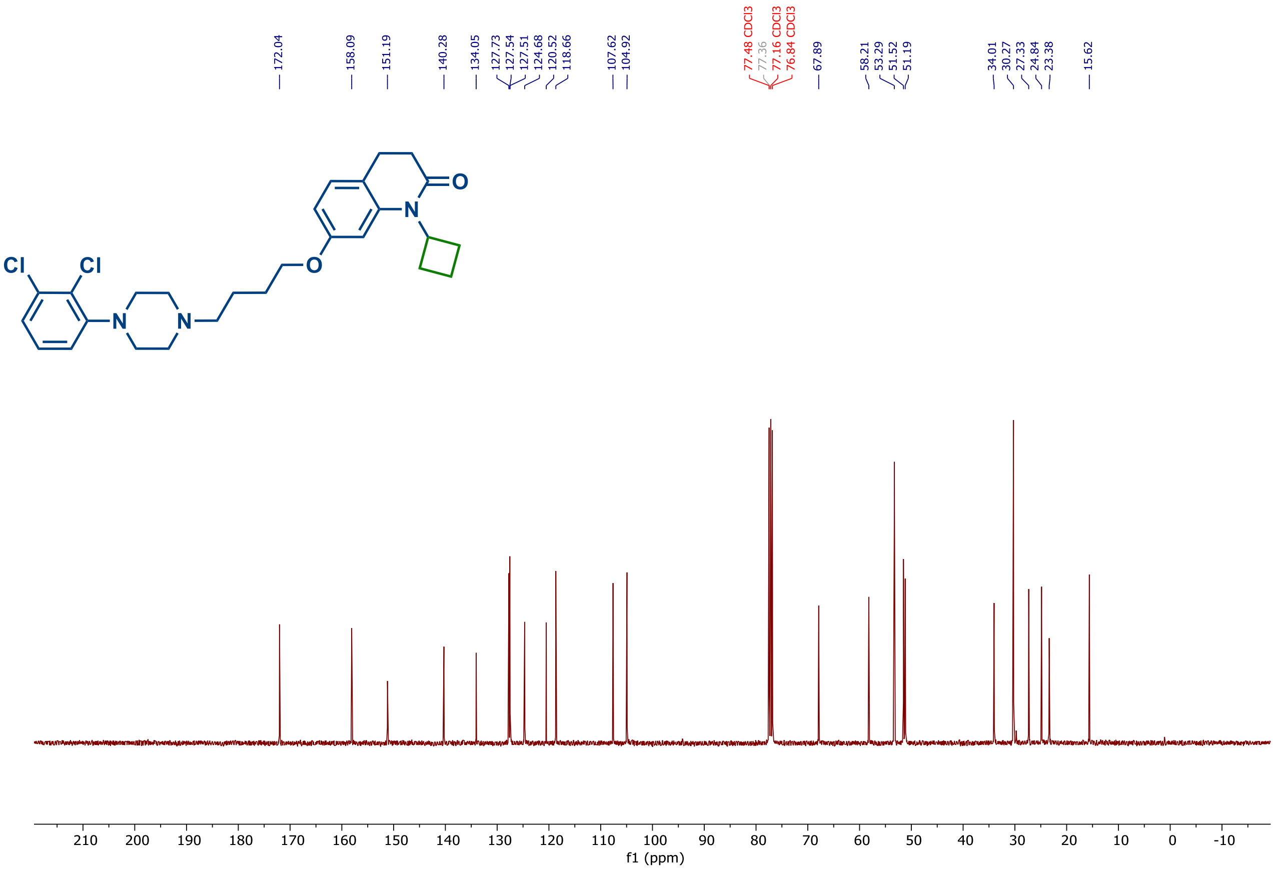

^{1}H NMR (400 MHz, $CDCl_3$) of **91** (*see procedure*)

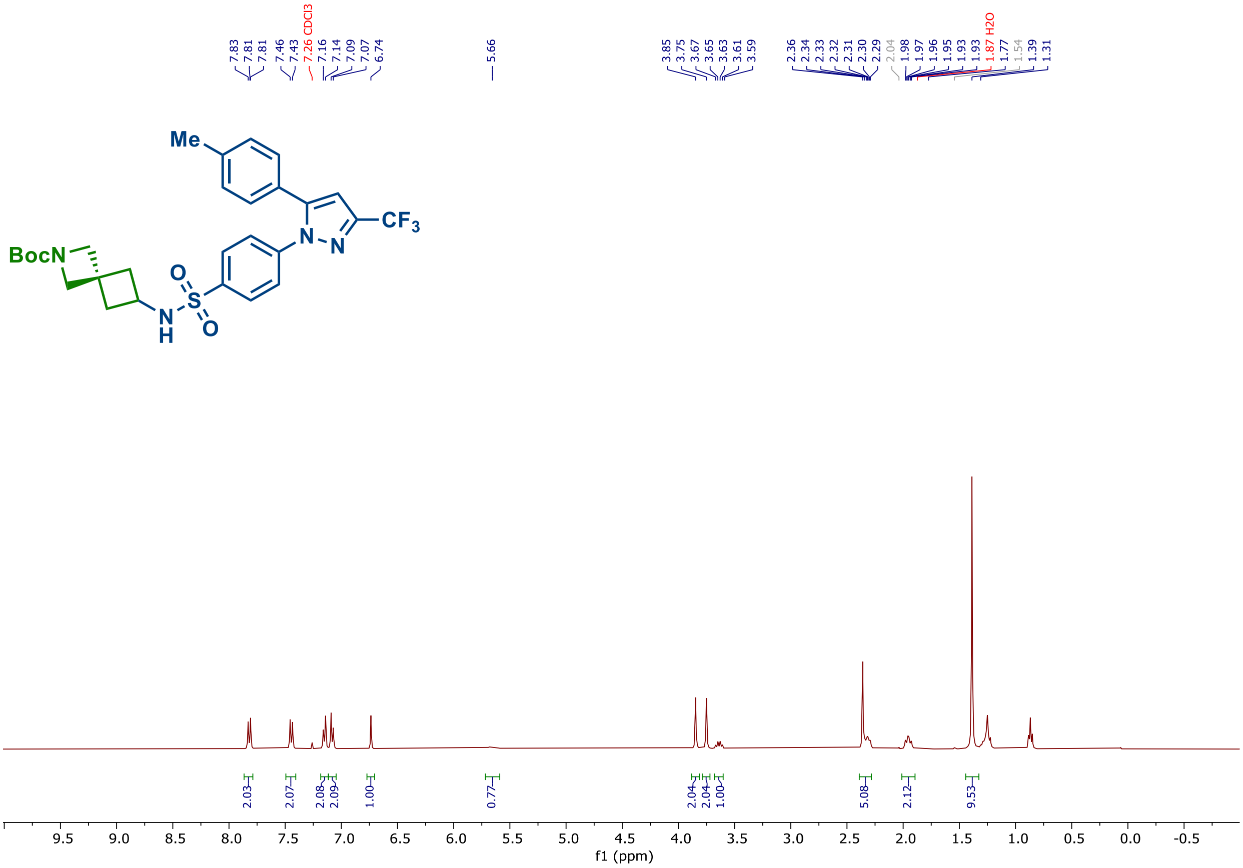

^{13}C NMR (101 MHz, $CDCl_3$) of **91**

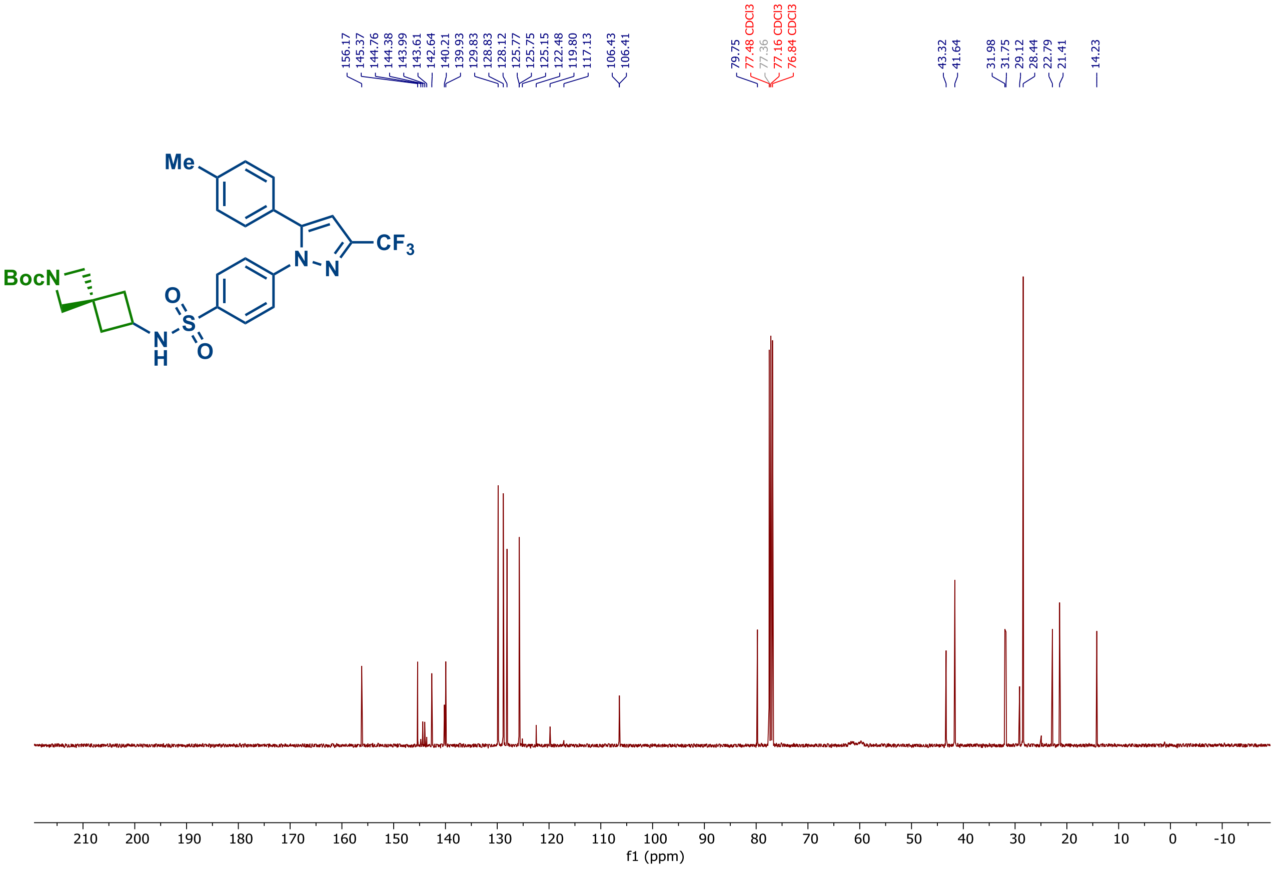

^{19}F NMR (376 MHz, $CDCl_3$) of **91**

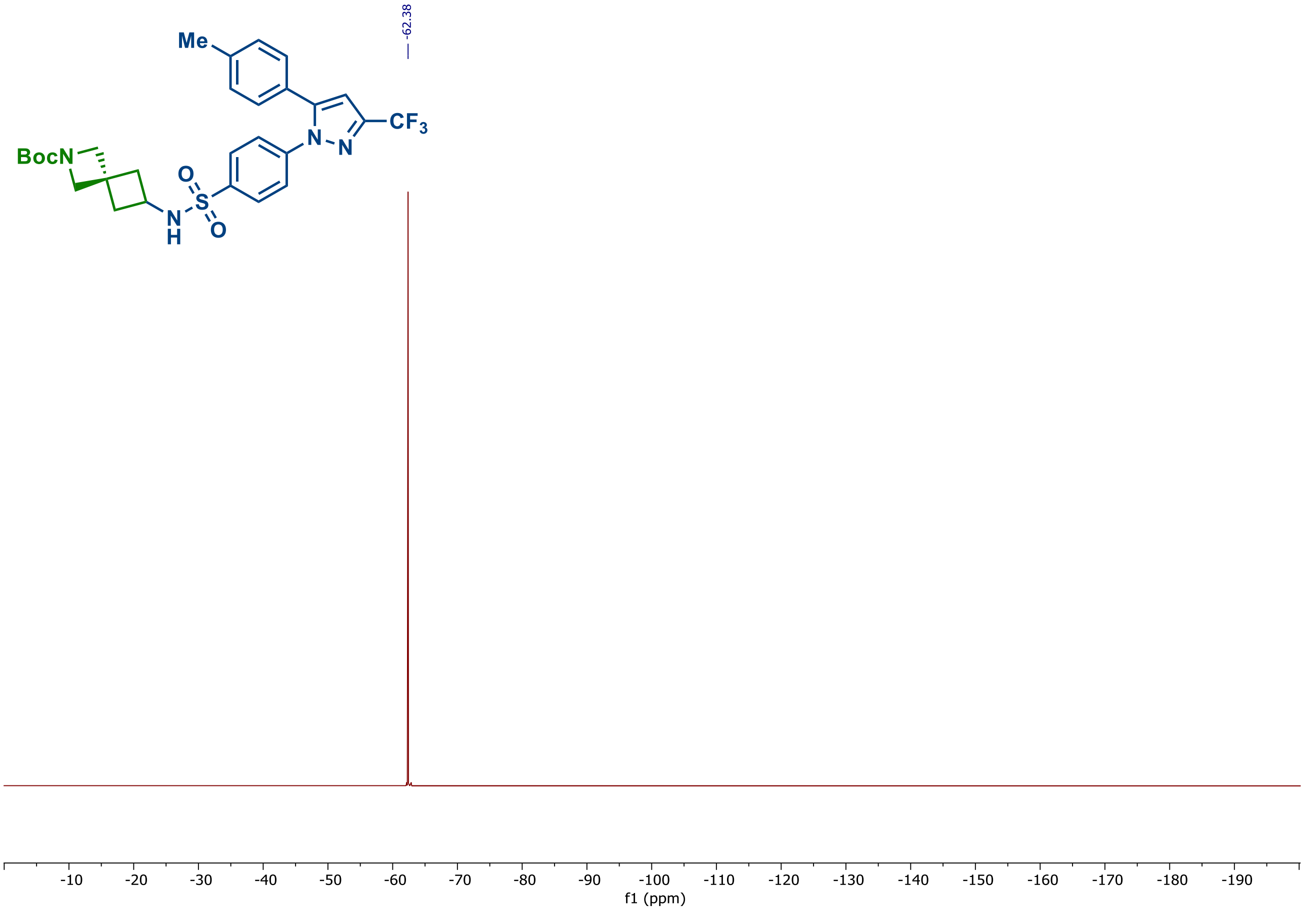

^{1}H NMR (400 MHz, acetic-*d*4) of **92** (*see procedure*)

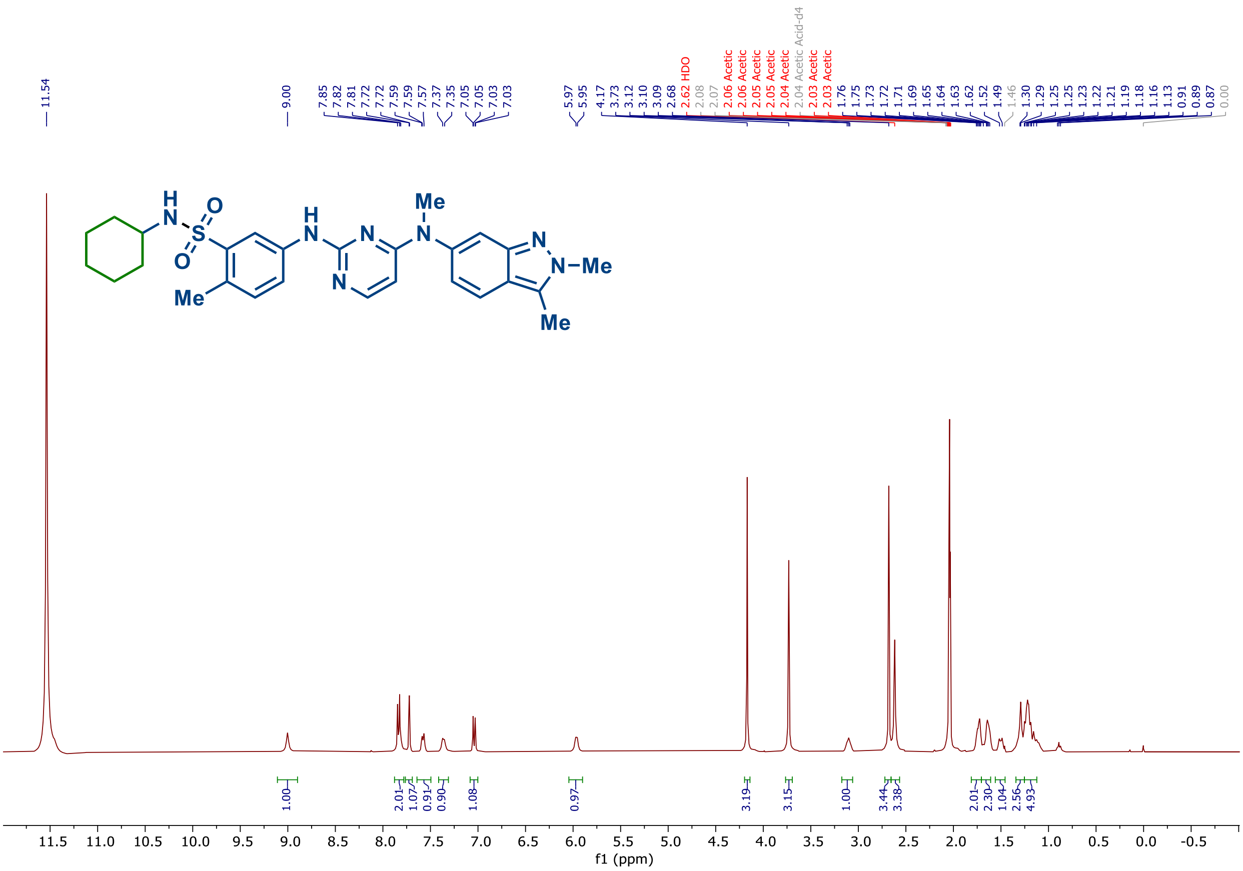

^{13}C NMR (101 MHz, acetic-*d*4) of **92**

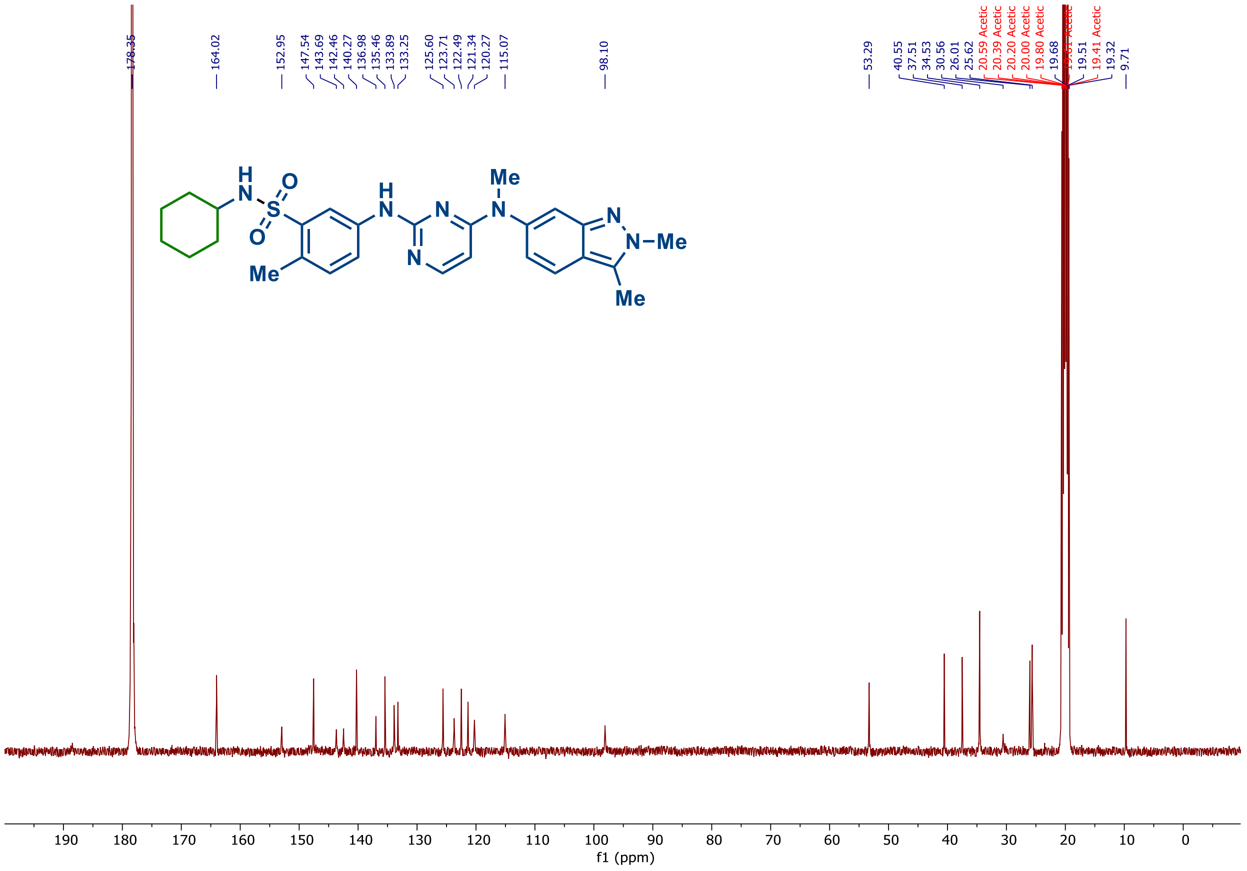

^{1}H NMR (400 MHz, $CDCl_3$) of **93** (*see procedure*)

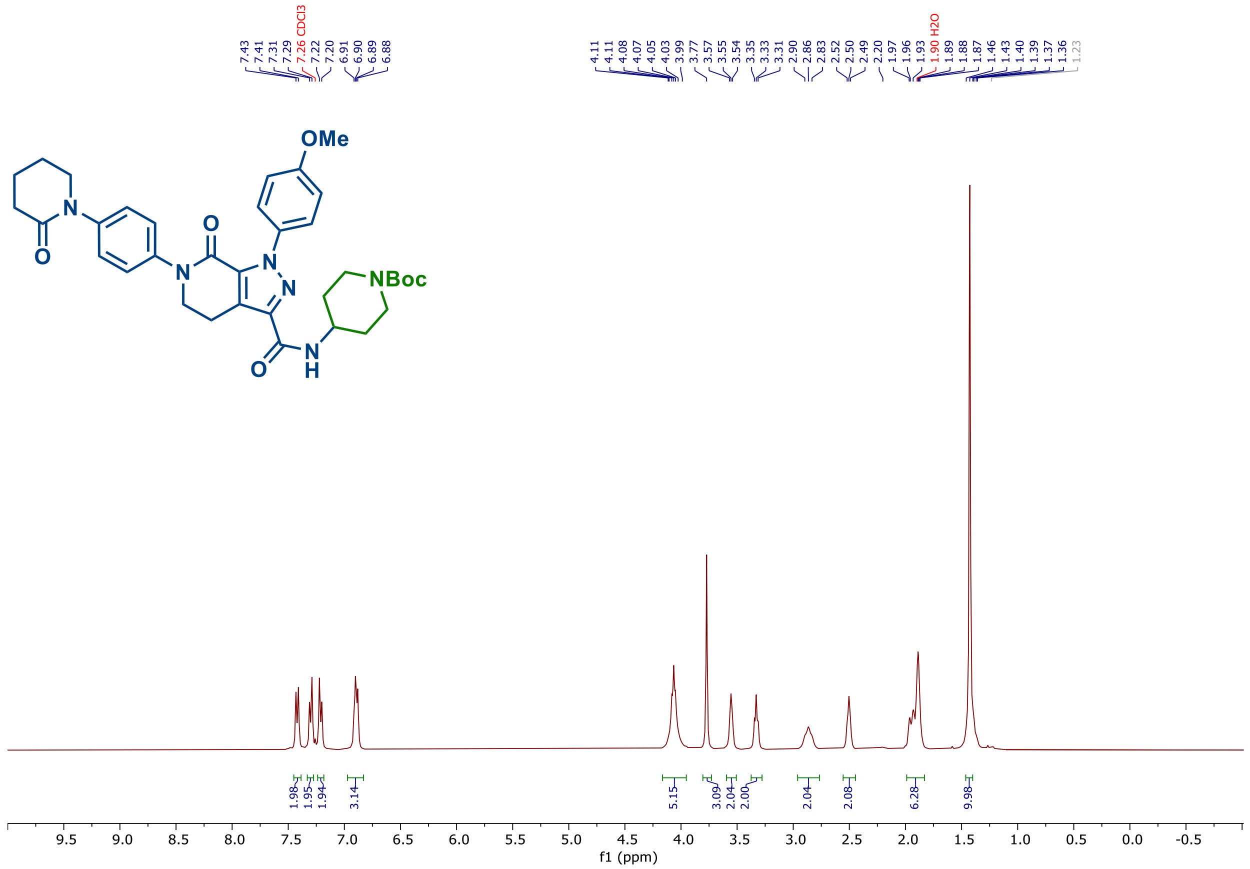

^{13}C NMR (101 MHz, $CDCl_3$) of **93**

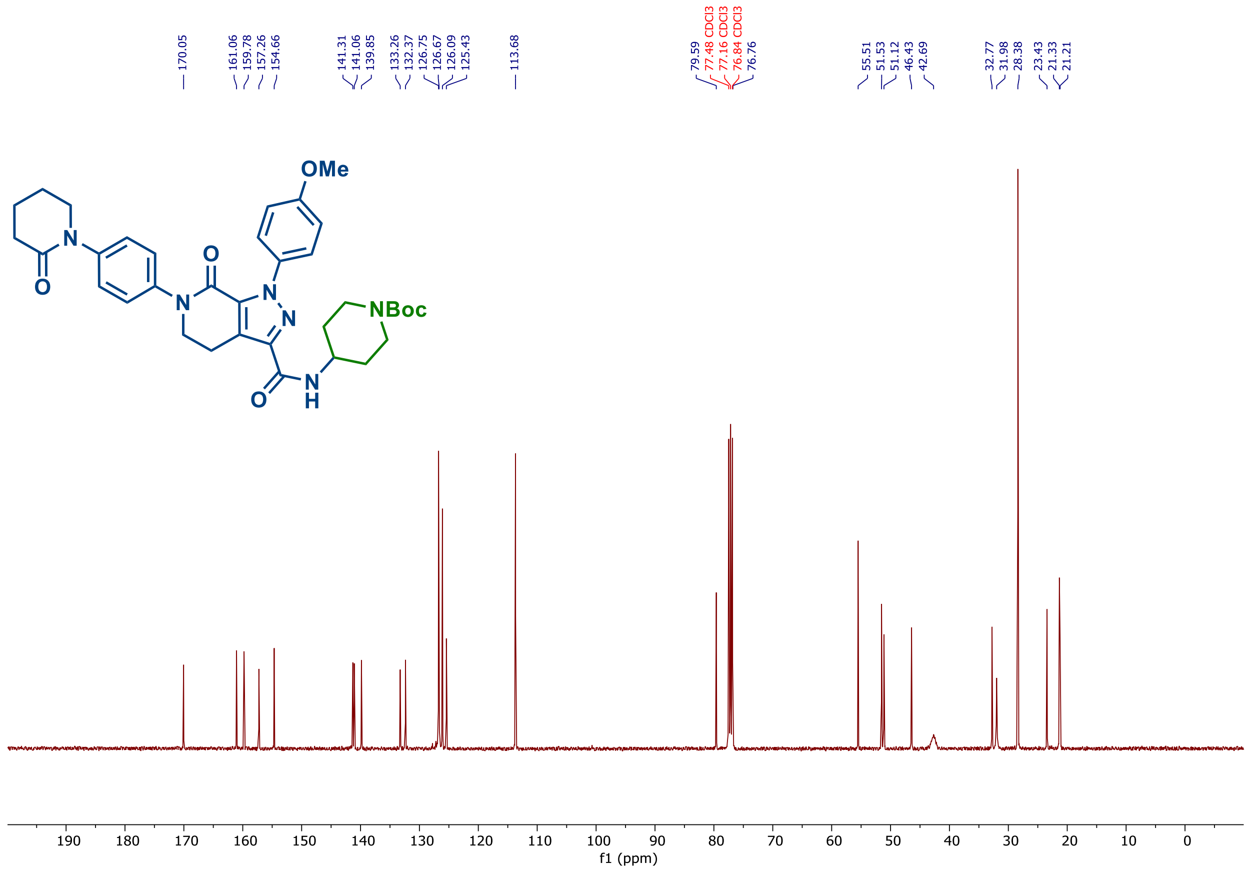

^{1}H NMR (400 MHz, $CDCl_3$) of **94** (*see procedure*)

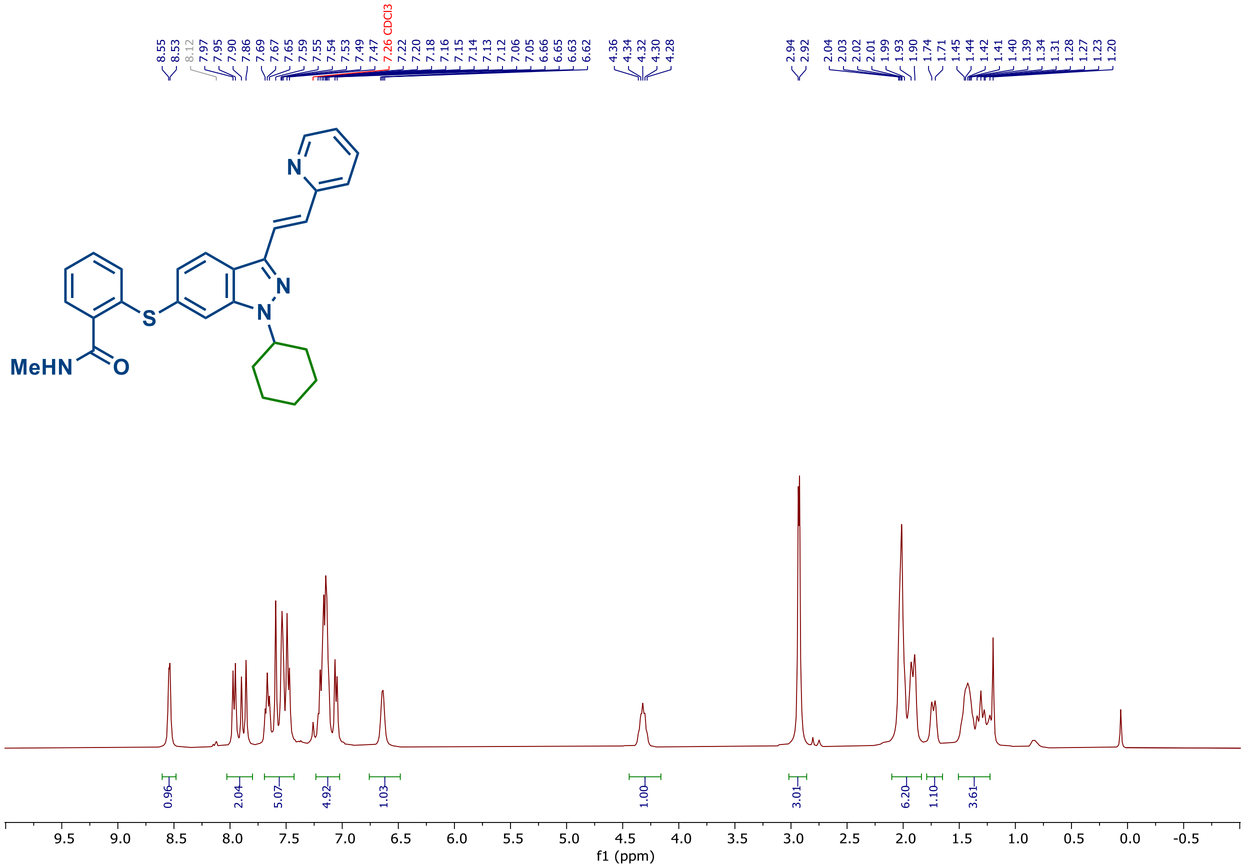

^{13}C NMR (101 MHz, $CDCl_3$) of **94**

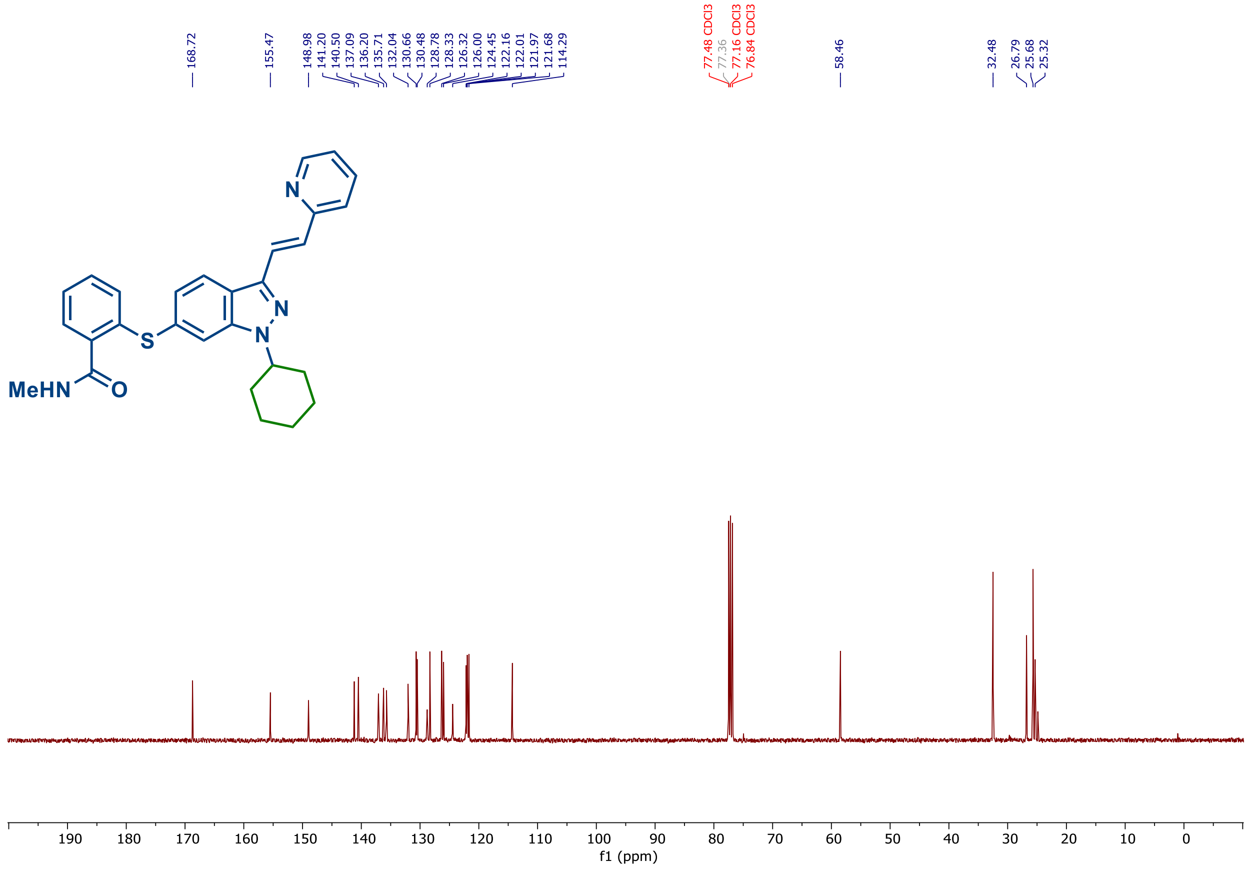

^{1}H NMR (400 MHz, $CDCl_3$) of **95** (*see procedure*)

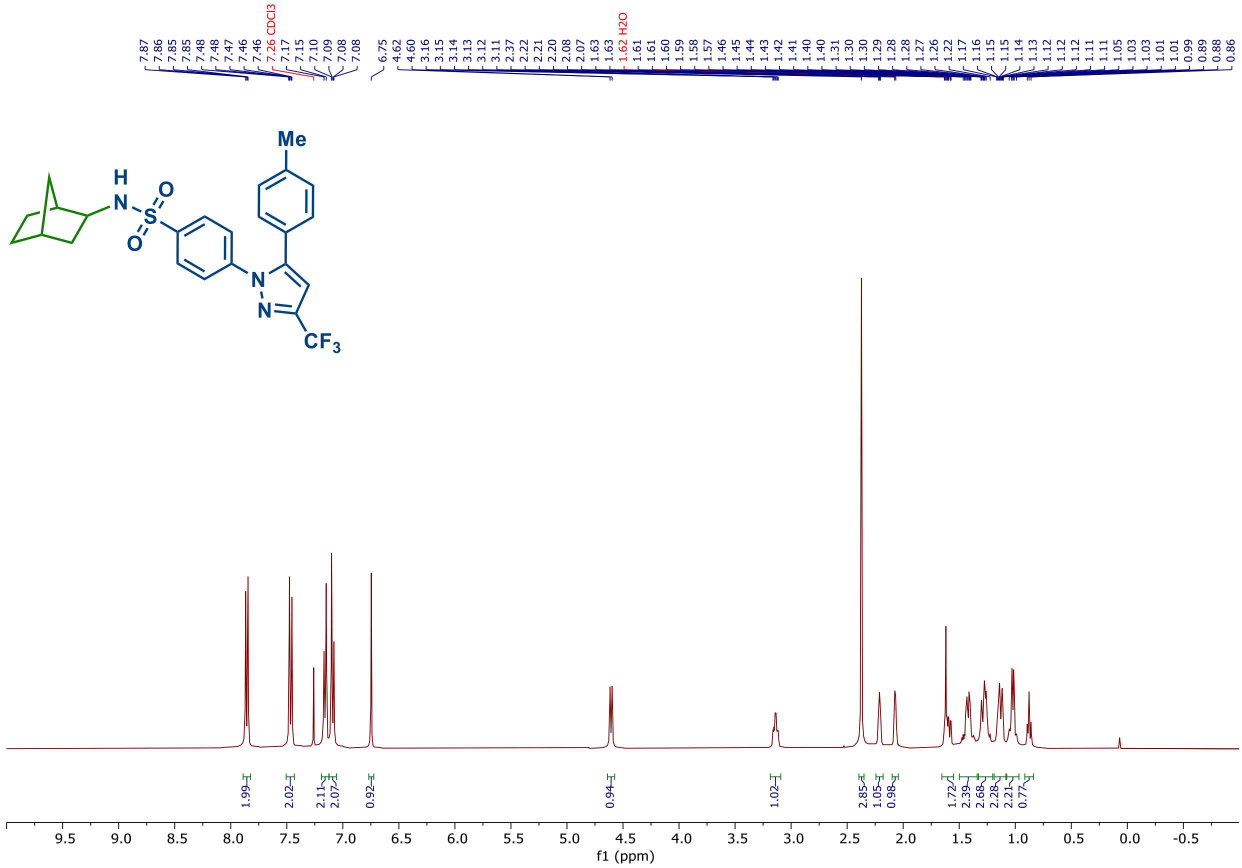

^{13}C NMR (101 MHz, $CDCl_3$) of **95**

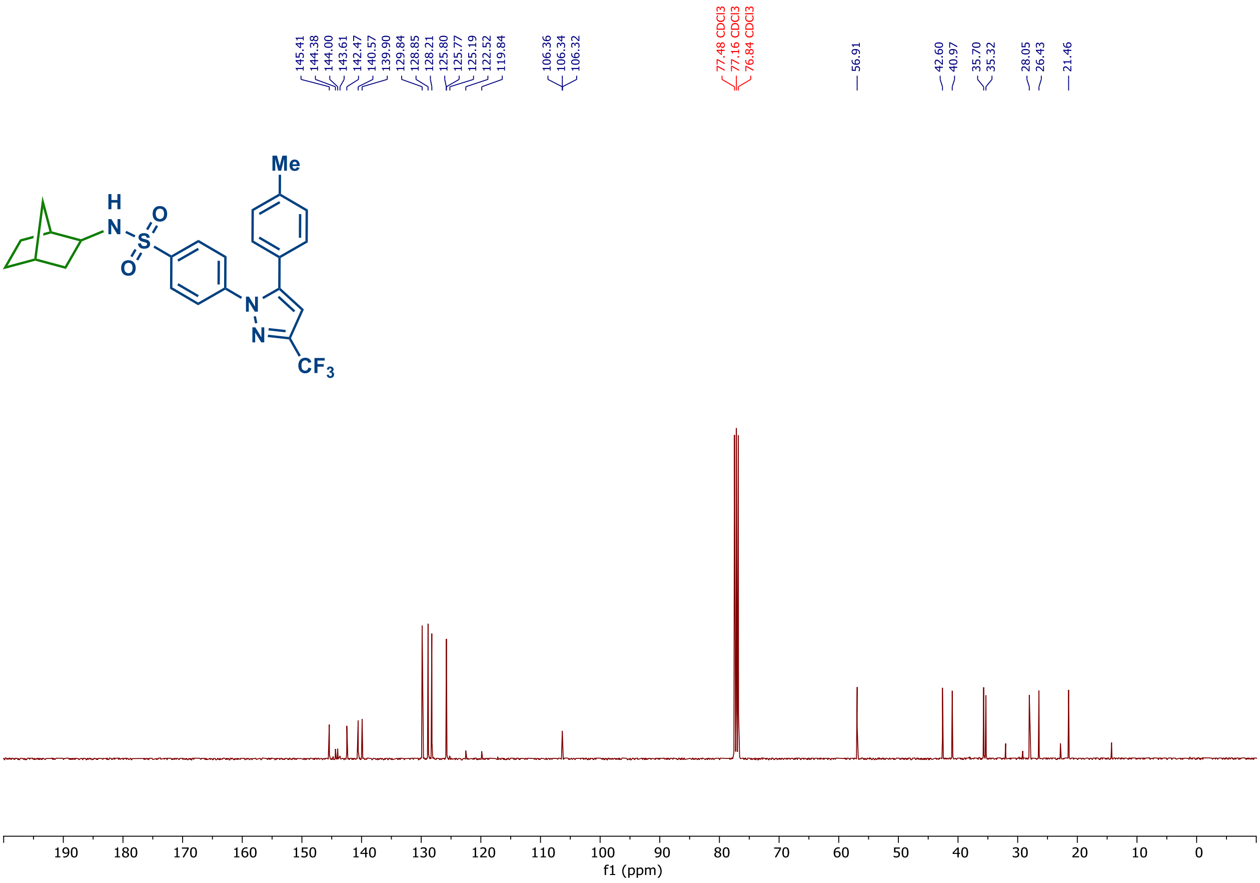

^{19}F NMR (376 MHz, $CDCl_3$) of **95**

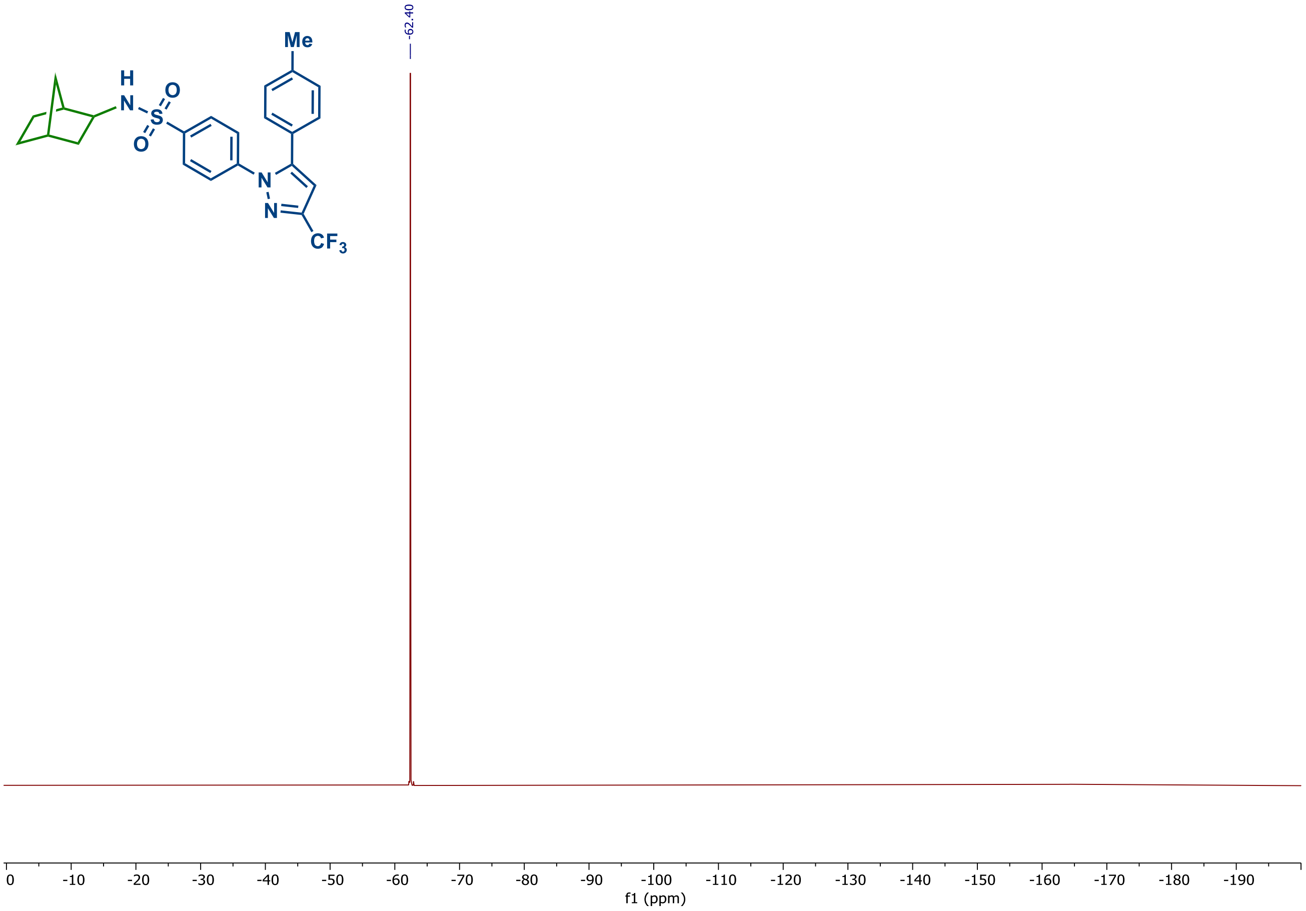

^{1}H NMR (400 MHz, $CDCl_3$) of **96** (*see procedure*)

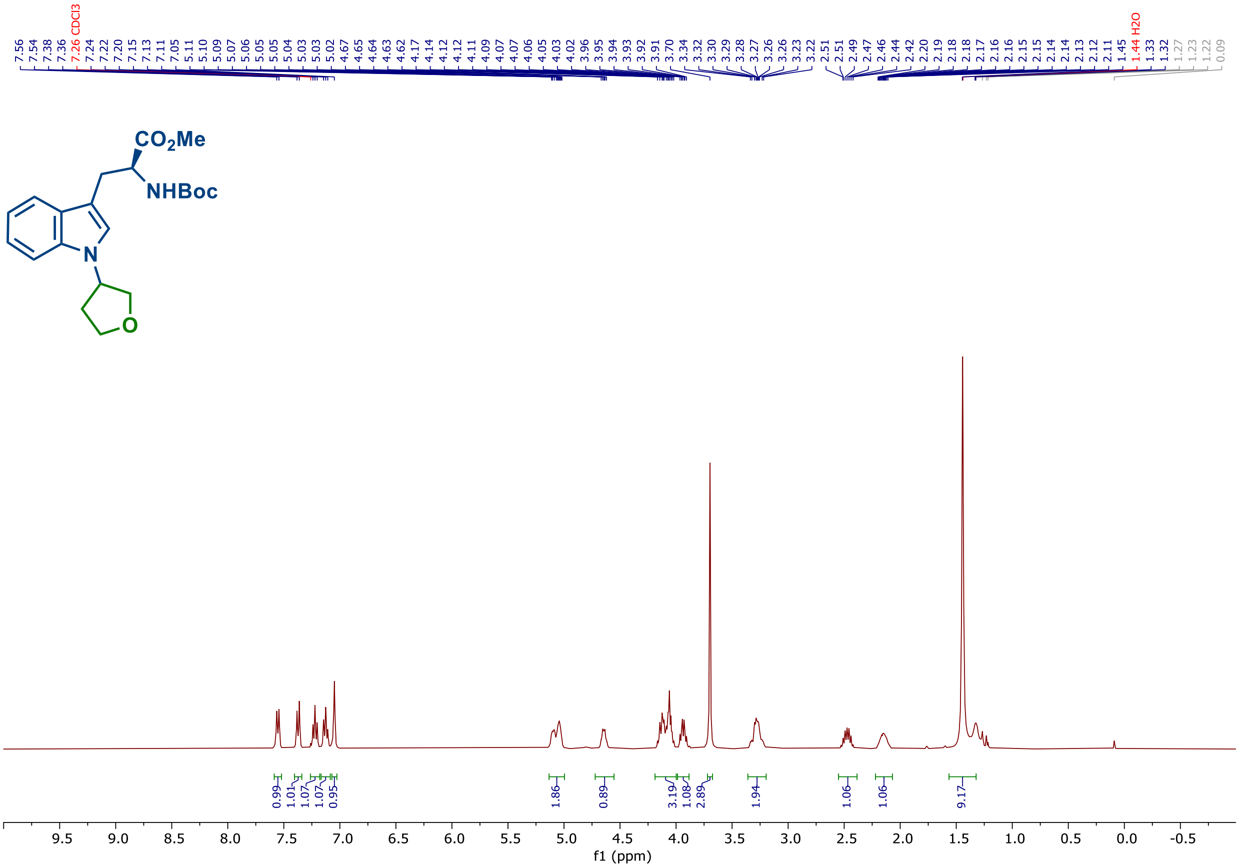

^{13}C NMR (101 MHz, $CDCl_3$) of **96**

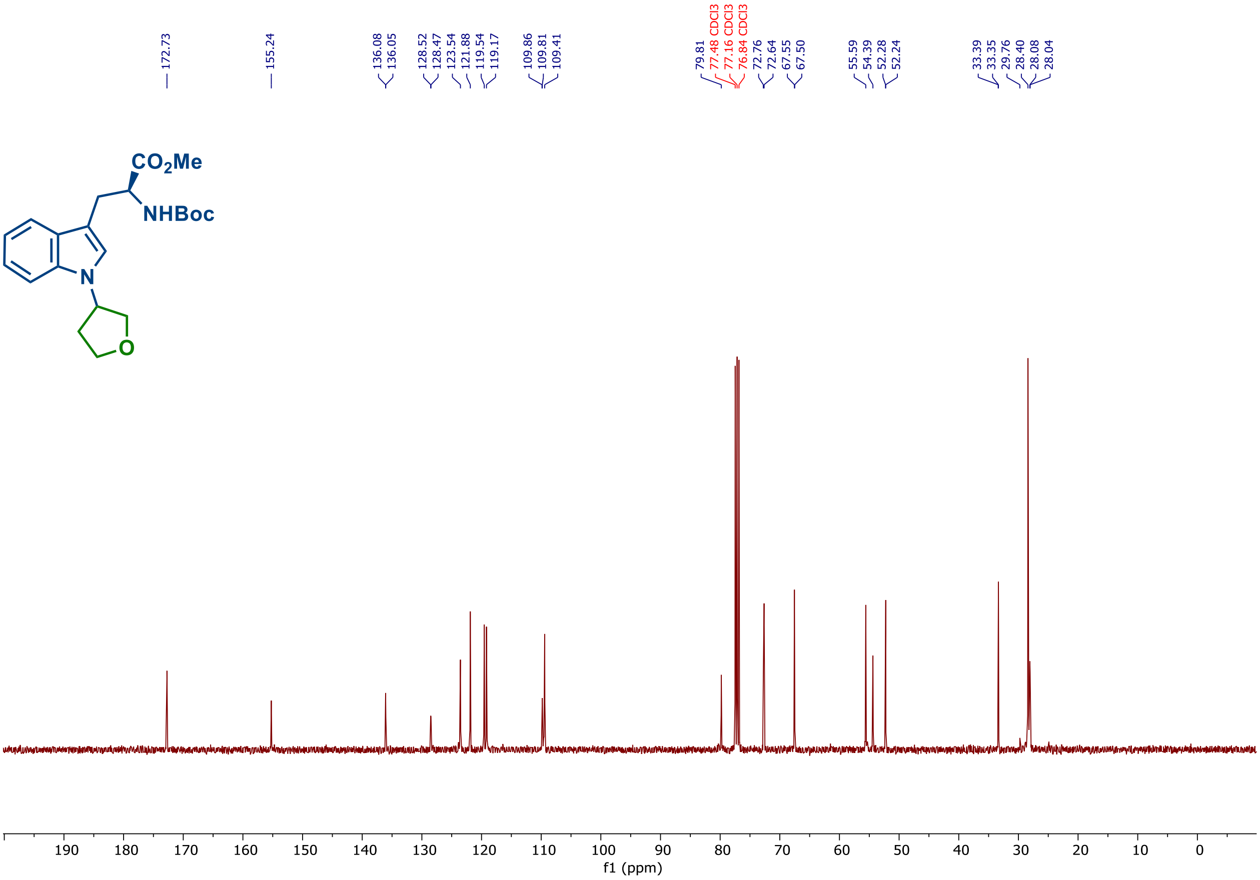

^{1}H NMR (400 MHz, $CDCl_3$) of **97** (*see procedure*)

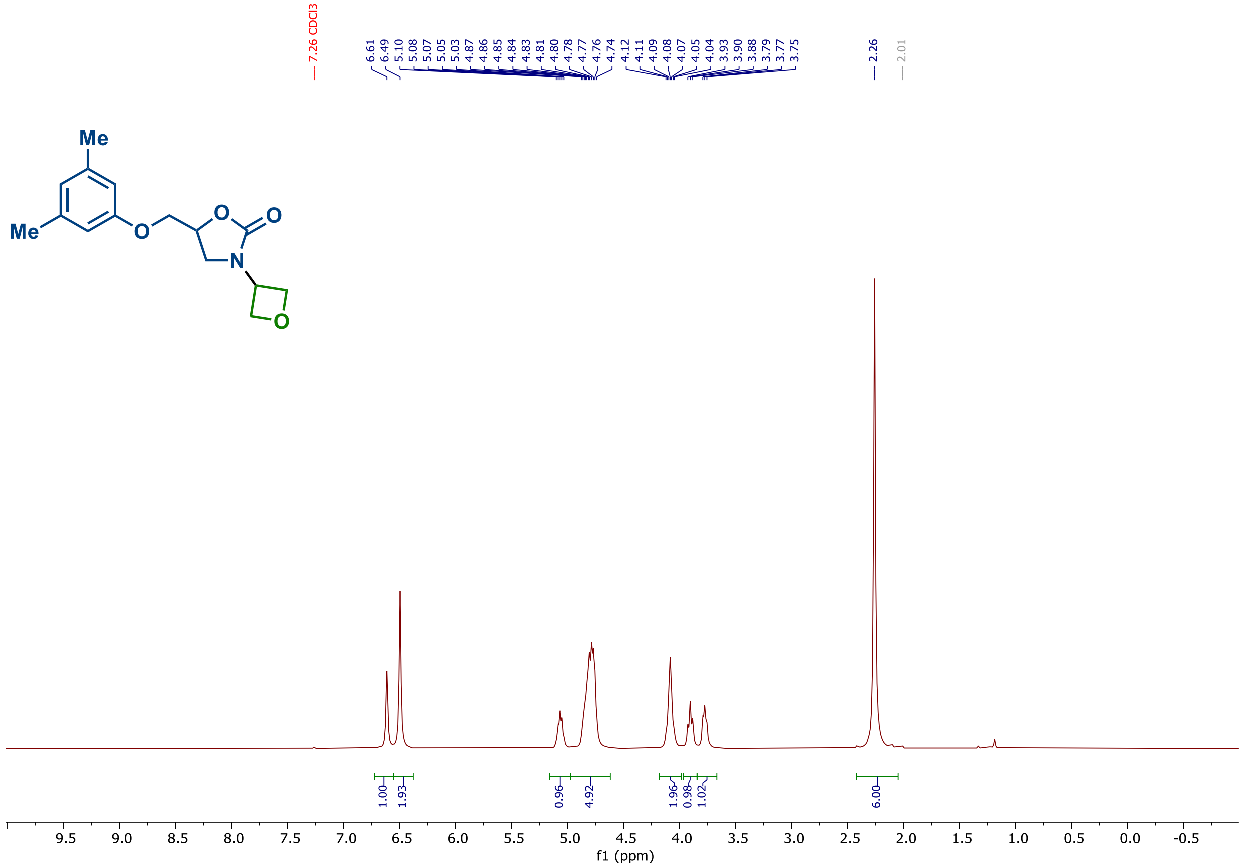

^{13}C NMR (101 MHz, $CDCl_3$) of **97**

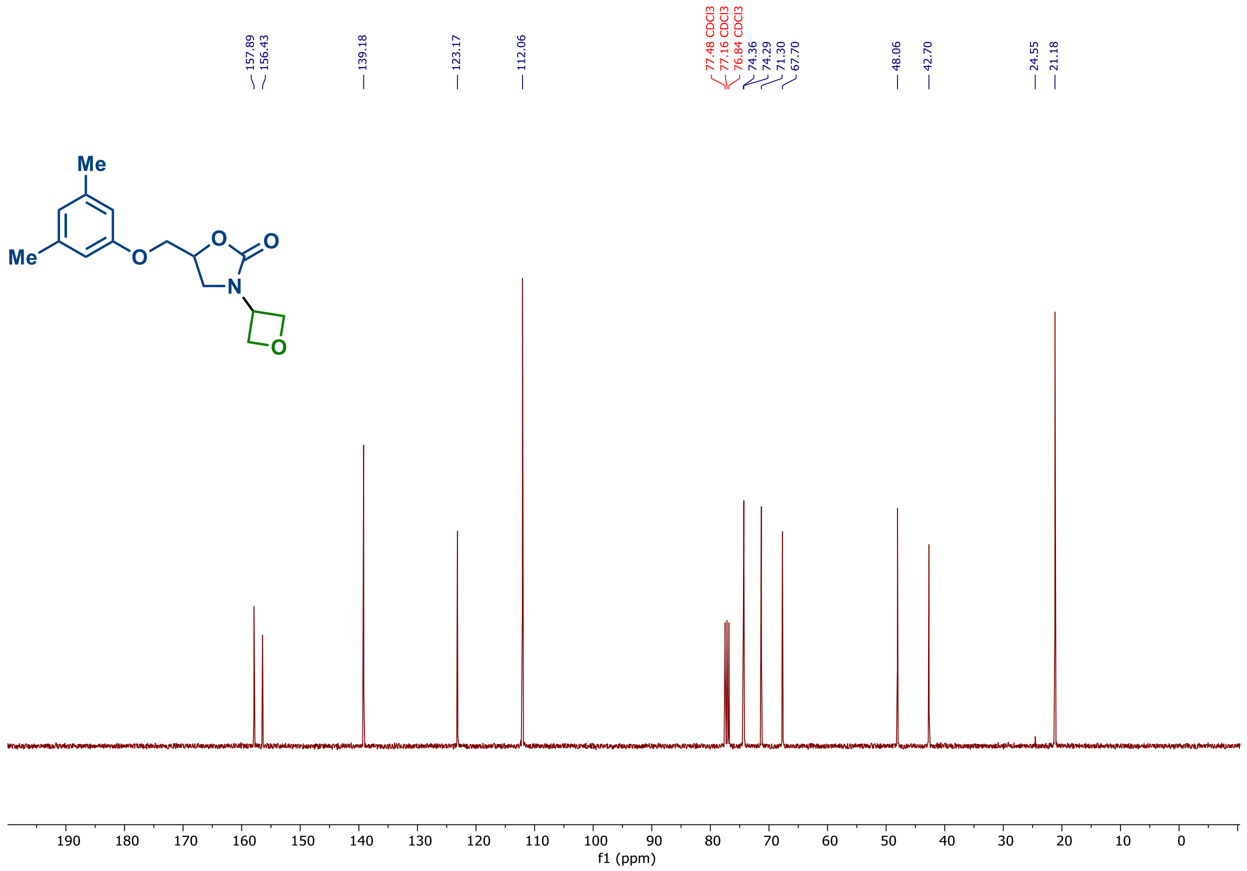

^{1}H NMR (400 MHz, $CDCl_3$) of **98** (*see procedure*)

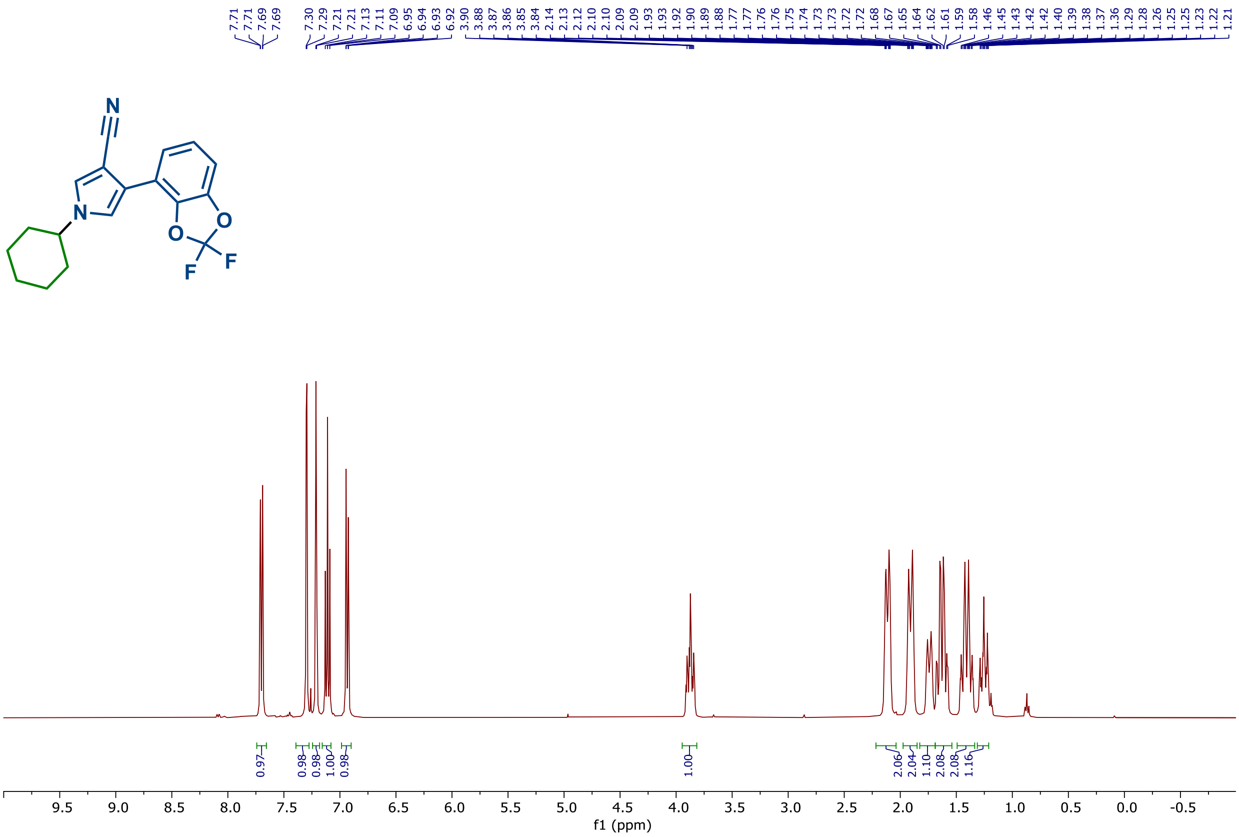

^{13}C NMR (101 MHz, $CDCl_3$) of **98**

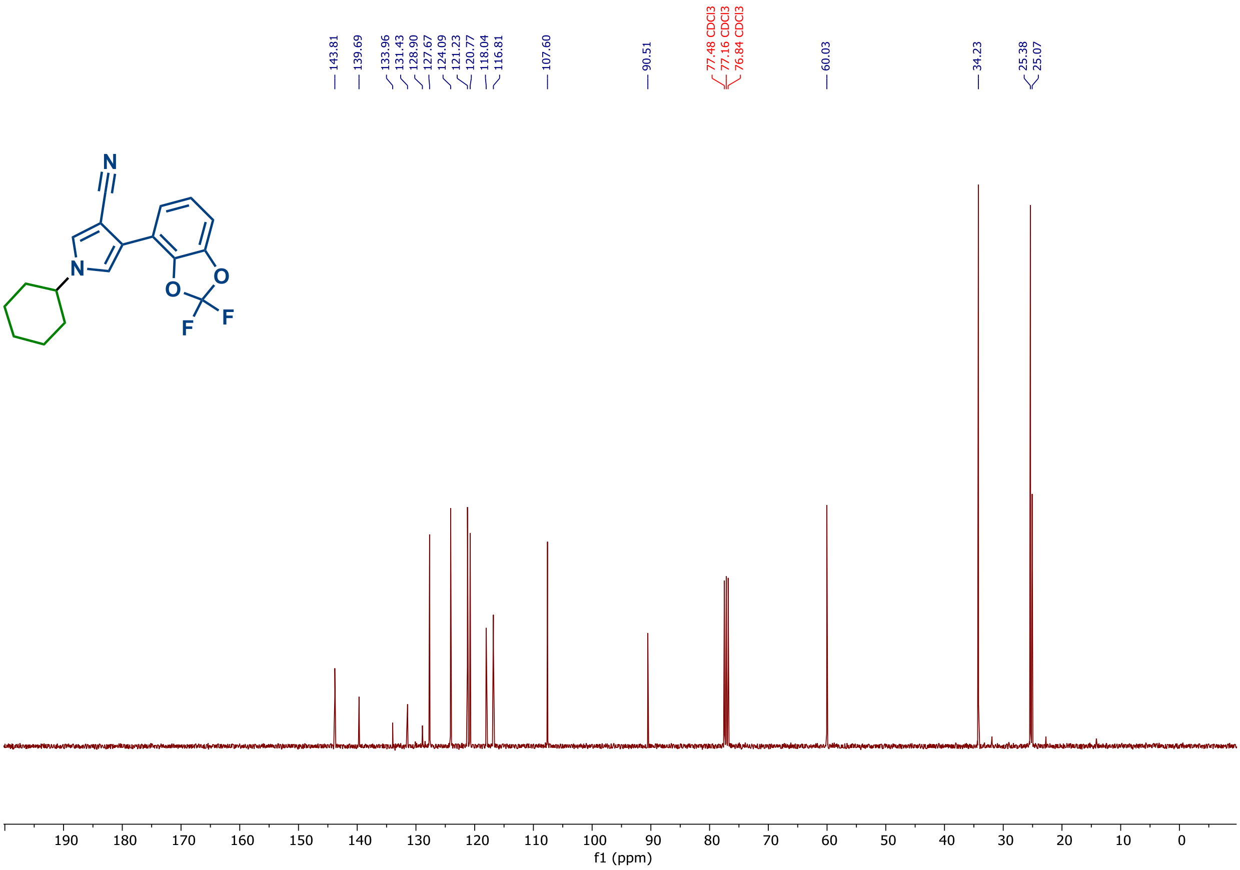

^{19}F NMR (376 MHz, $CDCl_3$) of **98**

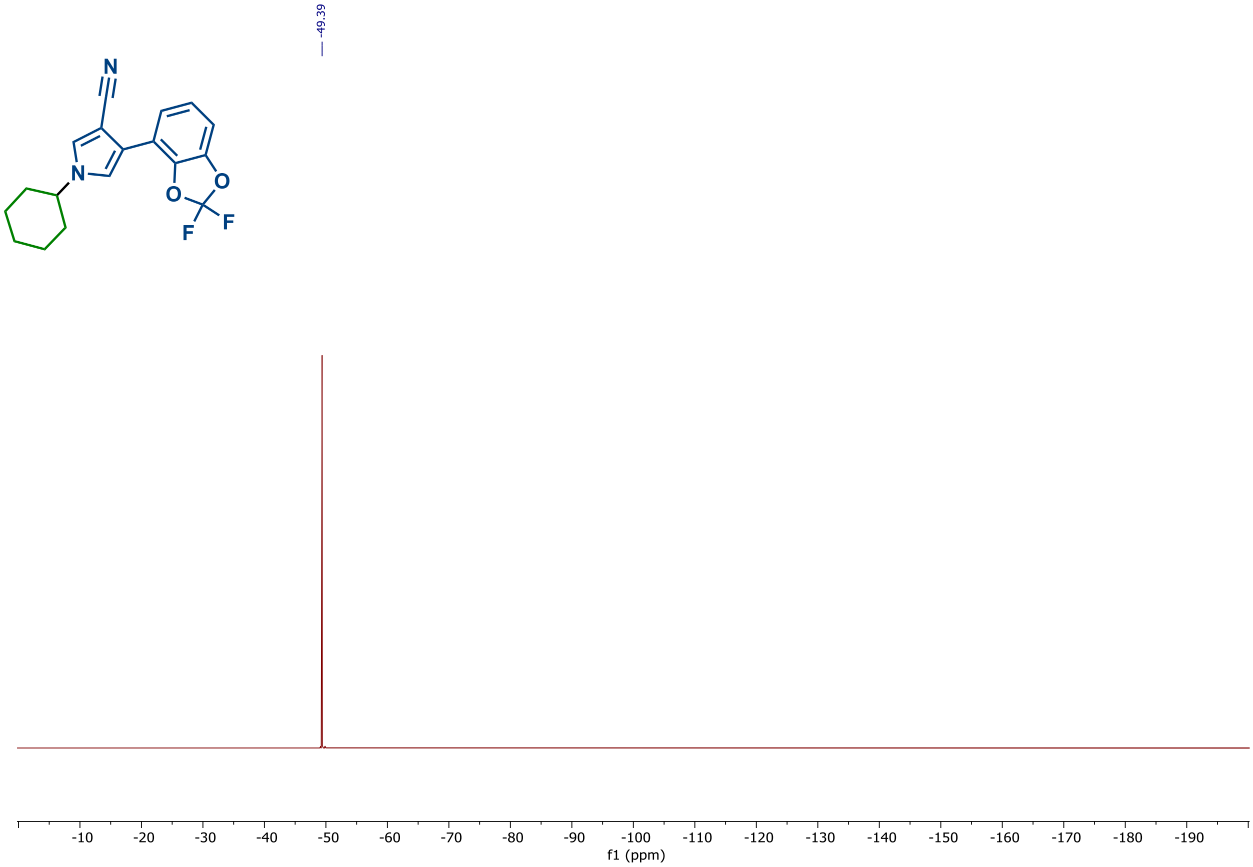

^{1}H NMR (400 MHz, $CDCl_3$) of **99** (*see procedure*)

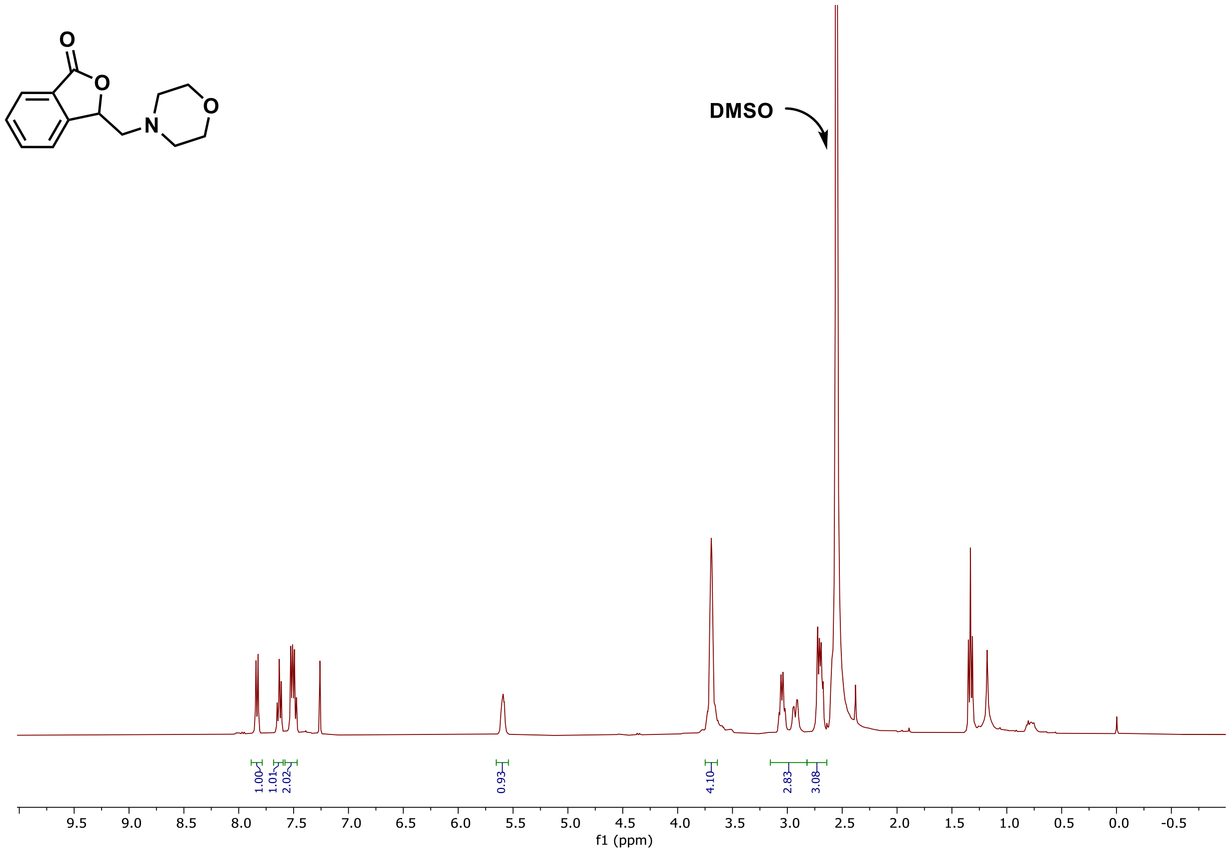

^{1}H NMR (400 MHz, $CDCl_3$) of **100** (*see procedure*)

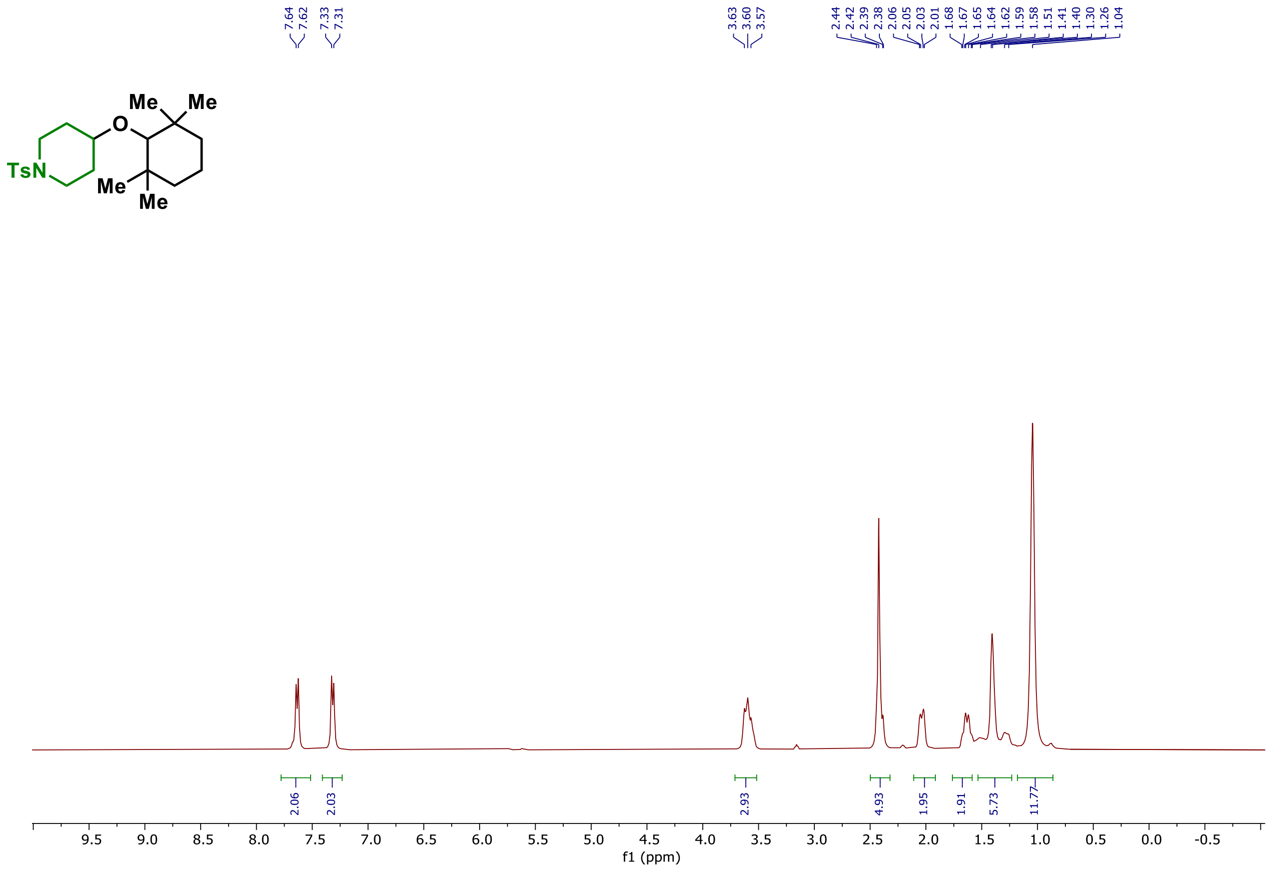

^{13}C NMR (101 MHz, $CDCl_3$) of **100**

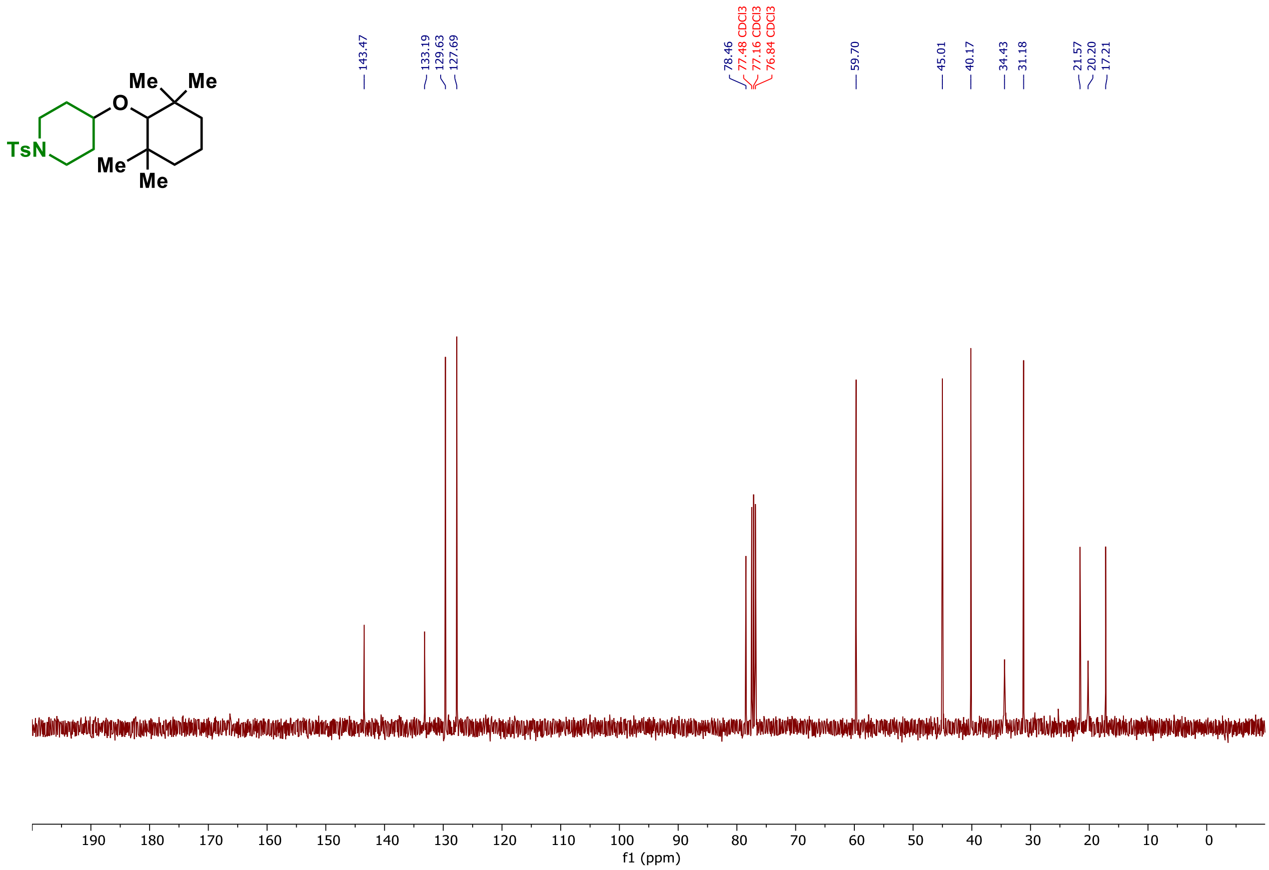

^{1}H NMR (400 MHz, $CDCl_3$) of **101a** (see procedure)

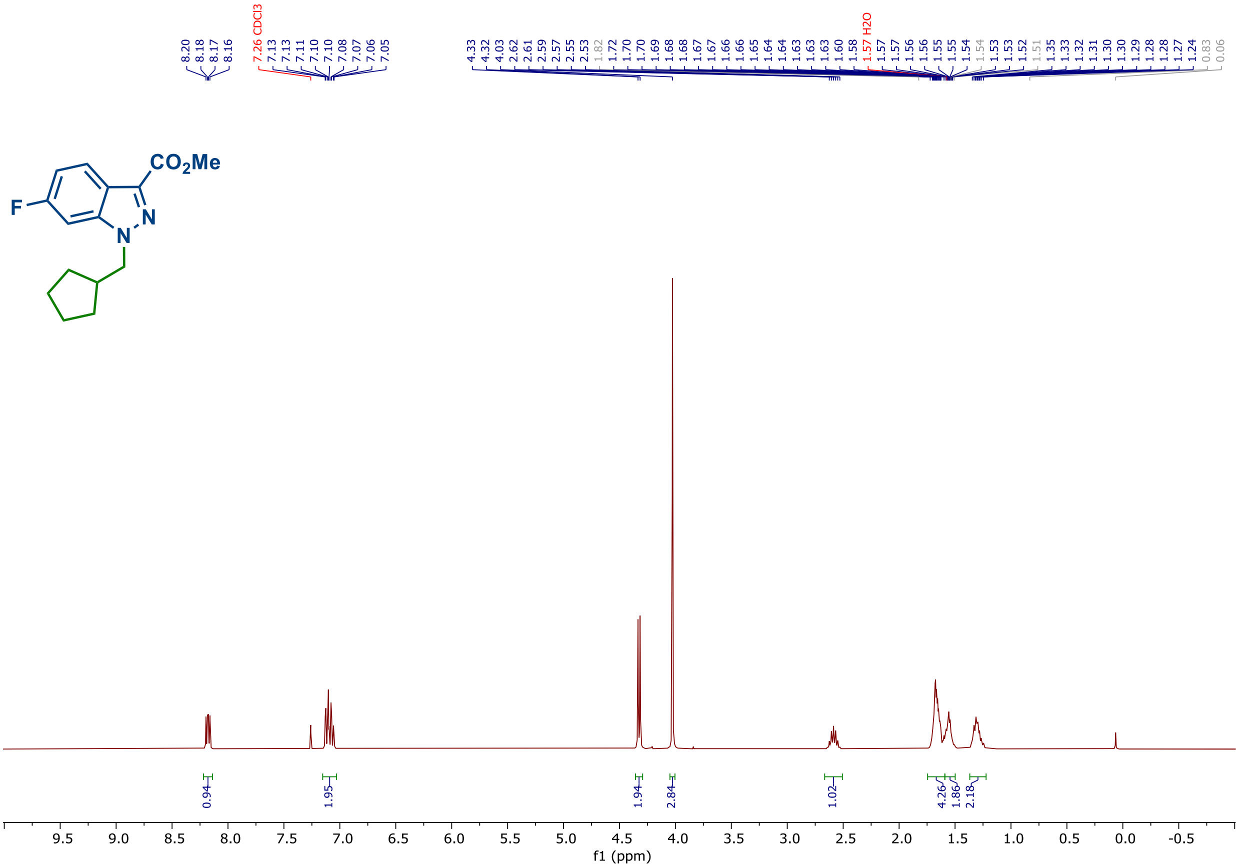

^{13}C NMR (101 MHz, $CDCl_3$) of **101a**

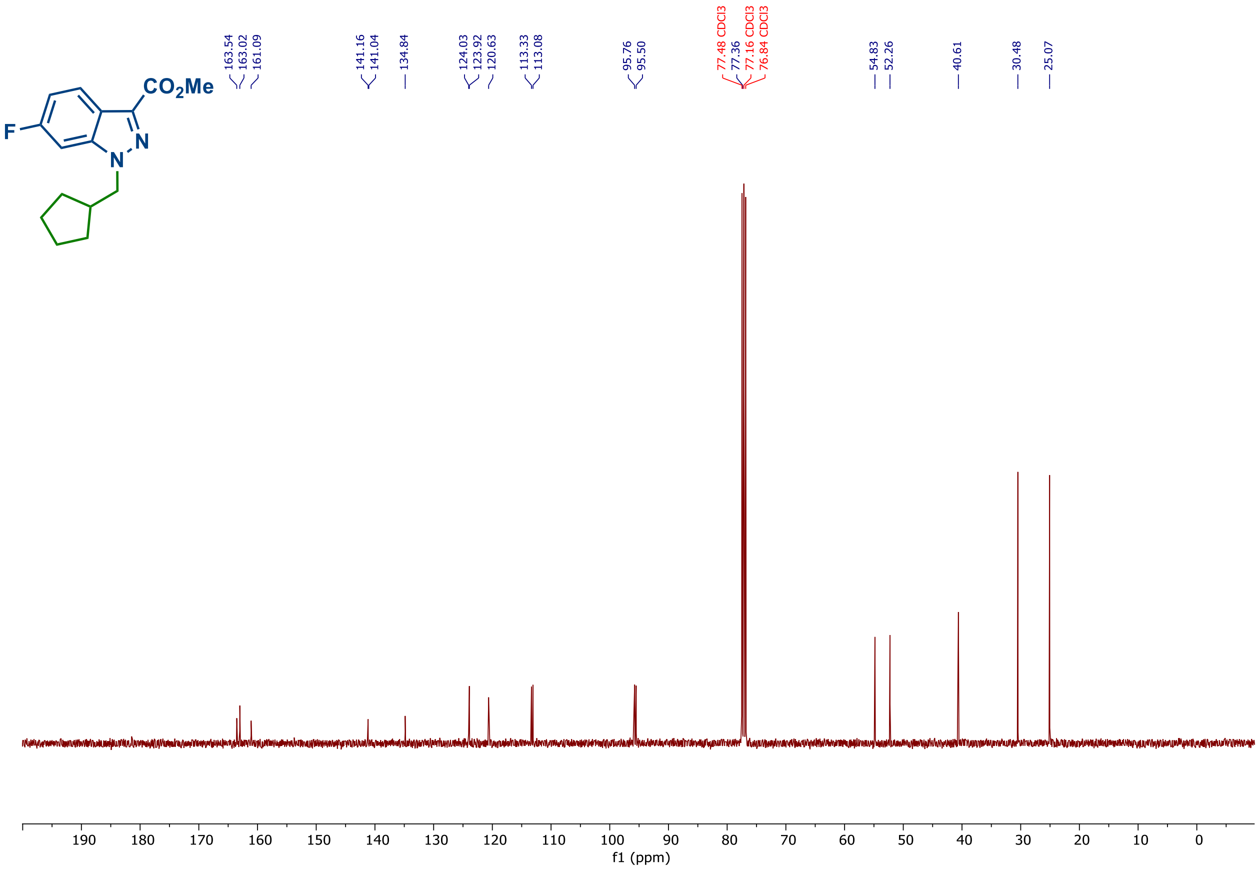

^{19}F NMR (376 MHz, $CDCl_3$) of **101a**

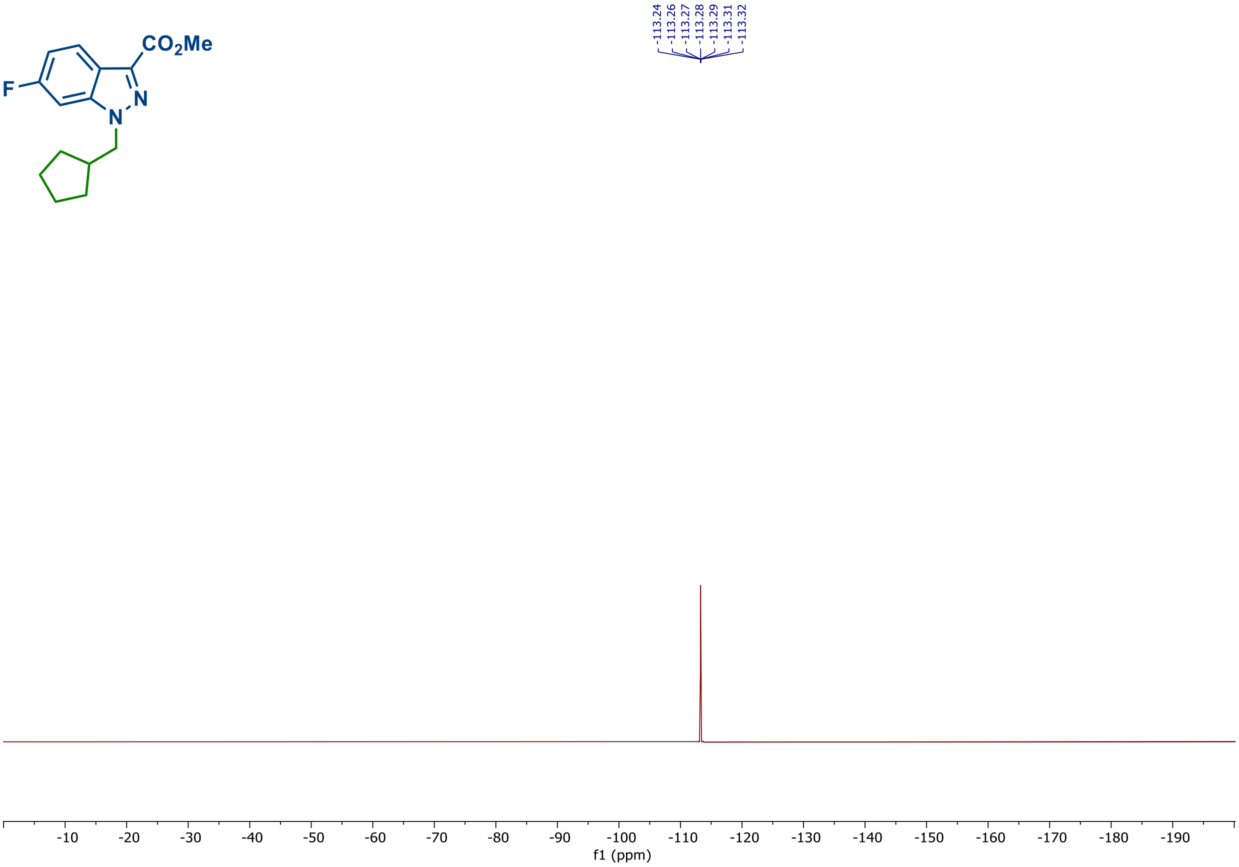

^{1}H NMR (400 MHz, $CDCl_3$) of **101a** and **101b** (*see procedure*)

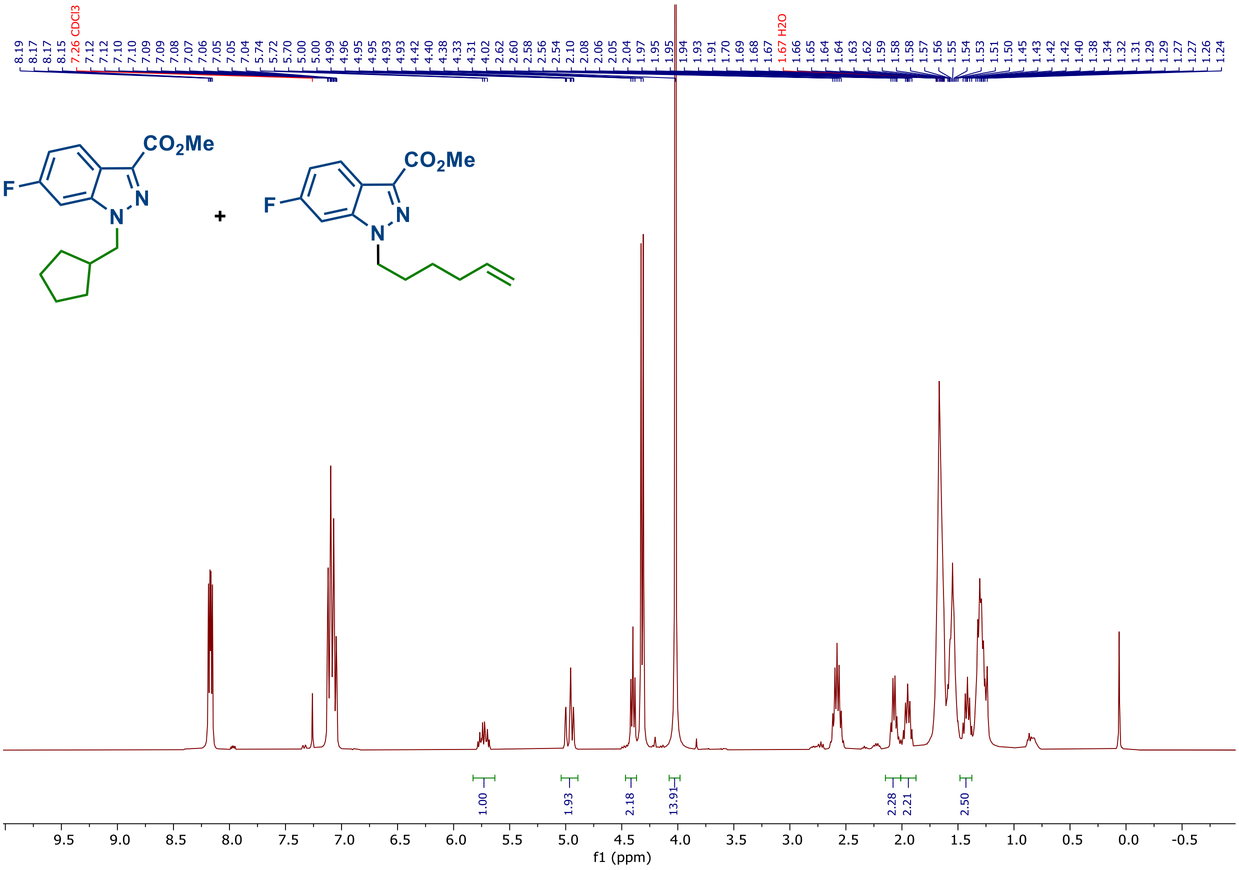

^{13}C NMR (101 MHz, $CDCl_3$) of **101a** and **101b**

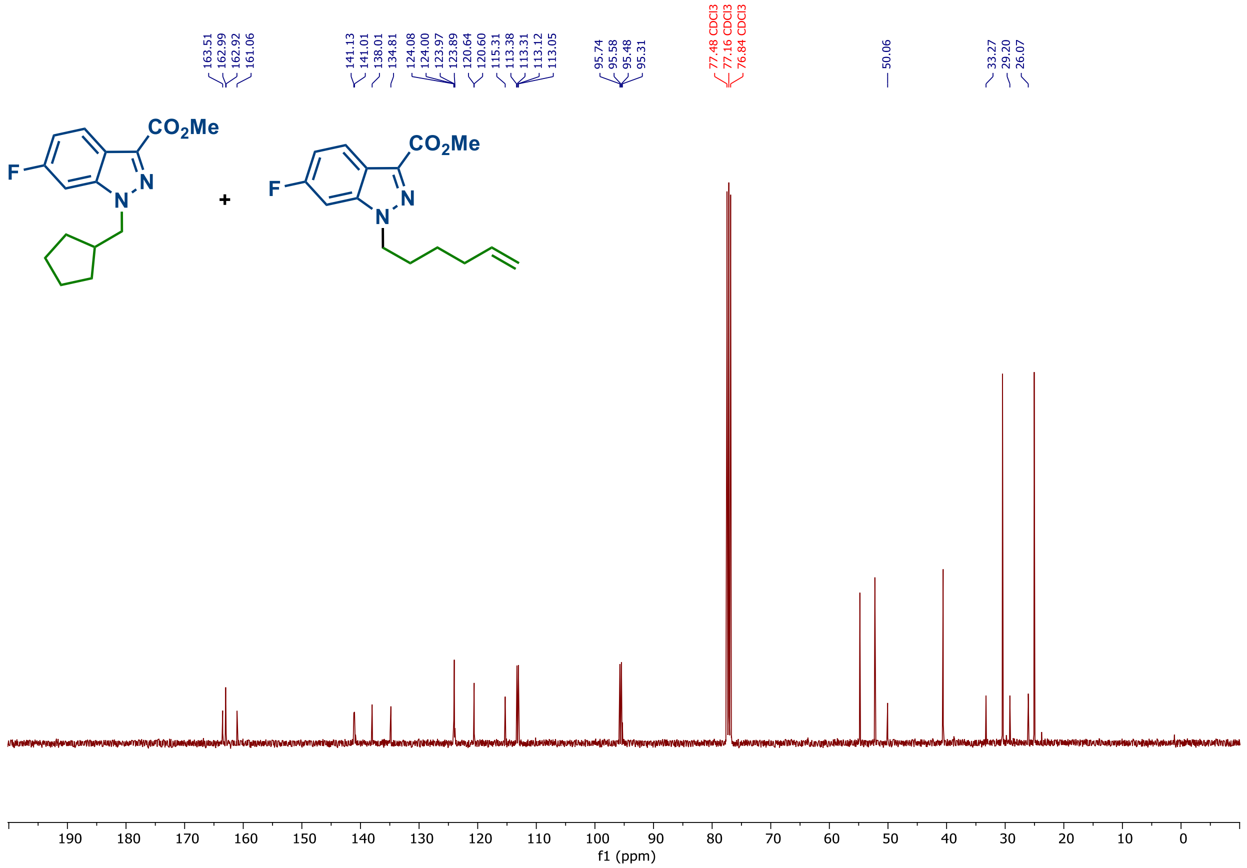

^{19}F NMR (376 MHz, $CDCl_3$) of **101a** and **101b**

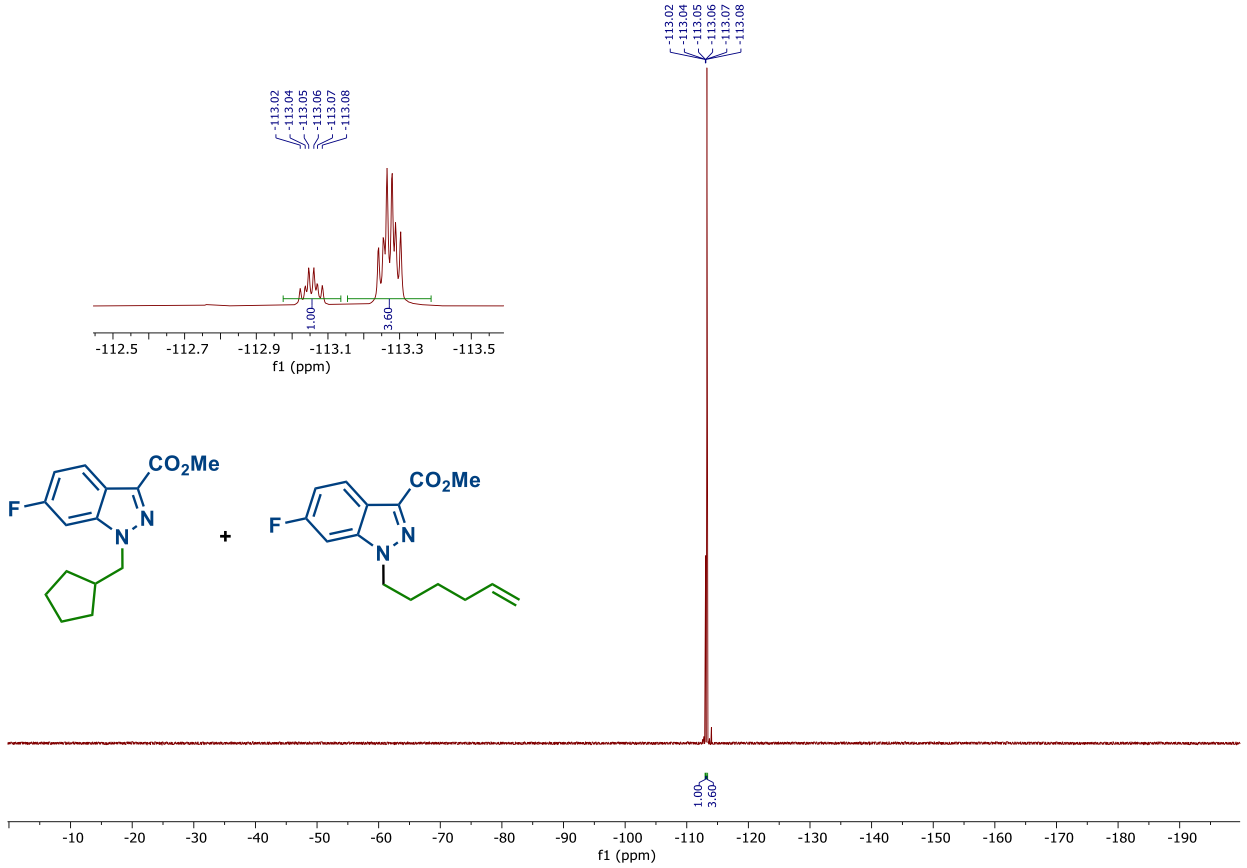

7. REFERENCES

1) Y. Liang, X. Zhang, D. W. C. MacMillan, *Nature* **2018**, *559*, 83–88.
2) A. G. Steinig, M. J. Mulvihill, J. Wang, D. S. Werner, Q. Weng, J. Kan, H. Coate, X. Chen, US20090197862 A1, **2009**.
3) J. Hood, S. K. C. Kumar, WO2013040215 A1, **2013**.
4) B. L. Tran, B. Li, M. Driess, J. F. Hartwig, *J. Am. Chem. Soc.* **2014**, *136*, 2555–2563.
5) X.-Y. Lv, R. Abrams, R. Martin, *Angew. Chem. Int. Ed.* **2023**, *62*, e202217386.
6) H. Keum, H. Jung, J. Jeong, D. Kim, S. Chang, *Angew. Chem. Int. Ed.* **2021**, *60*, 25235–25240.
7) T. Alam, A. Rakshit, H. N. Dhara, A. Palai, B. K. Patel, *Org. Lett.* **2022**, *24*, 6619–6624.
8) B. Górski, A.-L. Barthelemy, J. J. Douglas, F. Juliá, D. Leonori, *Nat. Catal.* **2021**, *4*, 623–630.
9) X. Han, W. Yue, Z. Wang, H. Xu, M. Yang, J. Zhu, *Org. Lett.* **2024**, *26*, 9305–9310.
10) N. W. Dow, A. Cabré, D. W. C. MacMillan, *Chem* **2021**, *7*, 1827–1842.
11) S. Bénard, L. Neuville, J. Zhu, *Chem. Commun.* **2010**, *46*, 3393–3395.
12) E. Racine, F. Monnier, J.-P. Vorsb, M. Taillefer, *Chem. Commun.* **2013**, *49*, 7412–7414.
13) F. M. Dennis, A. R. Arenas, G. Rodgers, M. Shanmugam, J. A. Andrews, S. L. Peralta-Arriaga, B. M. Partridge, *Chem. Eur. J.* **2024**, *30*, e202303636.
14) B. N. Hemric, K. Shen, Q. Wang, *J. Am. Chem. Soc.* **2016**, *138*, 5813–5816.
15) E. A. Romero, J. L. Peltier, R. Jazzara, G. Bertrand, *Chem. Commun.* **2016**, *52*, 10563.
16) H. Zhao, A. J. McMillan, T. Constantin, R. C. Mykura, F. Juliá, D. Leonori, *J. Am. Chem. Soc.* **2021**, *143*, 14806–14813.
17) M. Rueda-Becerril, C. C. Sazepin, J. C. T. Leung, T. Okbinoglu, P. Kennepohl, J.-F. Paquin, G. M. Sammis, *J. Am. Chem. Soc.* **2012**, *134*, 4026–4029.
18) H. Tian, W. Xu, Y. Liu, Q. Wang, *Org. Lett.* **2020**, *22*, 5005–5008.
